# The forest of knowledge under global change

**DOI:** 10.1038/s41586-026-10741-y

**Published:** 2026-07-08

**Authors:** Rodrigo Cámara-Leret, Patrick R. Roehrdanz, Jordi Bascompte

**Affiliations:** 1 Department of Systematic and Evolutionary Botany, University of Zurich, Zurich, Switzerland; 2 Moore Center for Science, Conservation International, Arlington, VA USA; 3 Department of Evolutionary Biology and Environmental Studies, University of Zurich, Zurich, Switzerland

**Keywords:** Tropical ecology, Ecosystem services, Environmental health, Climate-change ecology

## Abstract

Amazonia harbours more than 10% of the terrestrial biodiversity of the Earth^1^ and more than 400 Indigenous groups^2^. So far, however, no study has assessed how climate change and the loss of Indigenous languages may simultaneously impact its biological and cultural heritage. Here, to bridge this gap, we first assembled a database of 90,536 reports from 700 references to understand the societal benefits that native plants provide across all countries of the Amazon basin. We found that humans utilize 5,796 native plant species, which amounts to one-third of the known Amazon vascular seed plant flora. Next, analysing 8,429 species distribution models across three future climate scenarios (SSP1–2.6, SSP3–7.0 and SSP5–8.5), we show that climate change will produce a greater reduction in the ranges of utilized than of non-utilized species by 2060–2080. Locally, Indigenous cultures may lose an average of 28–34% of their utilized plant species and 18–23% of their associated services from climate change. Regionally, the loss of threatened Indigenous languages may result in a 26% reduction in the Amazonian knowledge pool. Overall, our results point to the strong climate and language vulnerability of Amazonian biocultural heritage. At the same time, these results—together with our publicly available dataset—may serve to guide biocultural restoration and reverse the growing global change effects on ecosystems and cultural traditions.

## Abstract (web summary)

The biocultural heritage of the Amazon, its plant species, plant services and cultures are at risk from climate change.

## Main

**Fig. 1:**
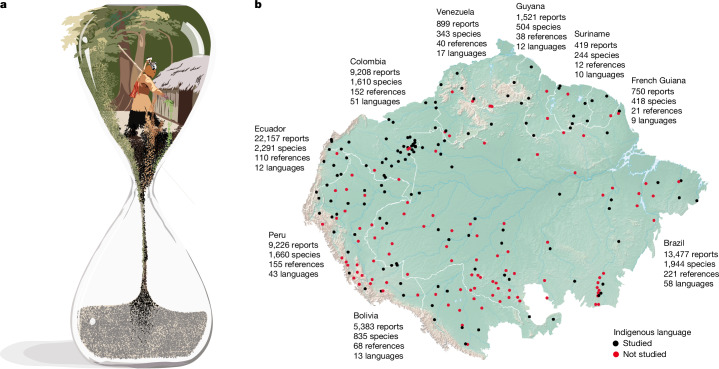
Linking biological and cultural heritage to study climate change impacts over time. **a**, Our analogy of a sand clock for a particular culture in Amazonia, containing knowledge of plant species (biological heritage represented by green grains) and plant services (cultural heritage represented by black or brown grains). In this paper, we assessed to what degree the biocultural heritage in this sand clock will be eroded by climate change by 2060–2080. In our drawings of sand clocks in Fig. 3, this is quantified by the volume of sand grains occupying the lower globe. **b**, Data on the benefits provided by plants to people compiled in this study from 700 references, 9 countries and 156 Indigenous languages of the Amazon basin. Illustration in (**a**) created by Inés Cámara Leret.

Biologists call Amazonia the ‘Earth’s lungs’—for it harbours around 40% of the remaining tropical forests of the world^3^. Anthropologists call it a ‘living library’—for the sophisticated knowledge of its Indigenous and local people^4^. So far, however, no interdisciplinary effort has been made to link ecological data with Indigenous knowledge to quantify the climate and language change exposure of Amazonian biological and cultural heritage. On the one hand, ecologists assessing the impacts of climate change have emphasized two scales: ecosystems (that is, forest–savannah transitions^5^) and organisms (for example, animals^6^ and plants^7^). On the other hand, social scientists have addressed the importance of Indigenous and local knowledge for climate change mitigation^8,9^, but have not assessed the fate of the species that matter to people. As a result of these separate investigations, our understanding of the climate and language change vulnerability of the unique biocultural heritage of Amazonia—of its plant species, plant services and cultures—remains incipient (Fig. 1a).

## Amazonian plants utilized by people

Humans first reached Amazonia approximately 13,000 years ago^10^ and initiated a process of biodiscovery that lasts to this day^11^. Understanding how Amazonian societies utilize and manage local plant resources has been a major focus of scientific research, with notable contributions by Marcgrave and Piso (seventeenth century), Aublet (eighteenth century), von Humboldt, von Martius and Spruce (nineteenth century) and Schultes (twentieth century), among others. These efforts have shown that Amazonia is an important centre for plant domestication^12^, that its Indigenous communities are intimately familiar with the identity and properties of thousands of edible and medicinal species^13^, and that its flora is the most diverse in the world^14,15^. So far, however, no synthesis on the interactions between people and plants exists across the basin, rendering its Indigenous and local knowledge and the contributions of the Amazonian flora to the world largely underappreciated. Furthermore, the scattered nature of previous research makes its access by local communities difficult, limiting the value of historical works for cultural revitalization efforts. Together, knowledge gaps and lack of access to information hinder biocultural conservation efforts on plants that matter to people.

To assess to what degree climate and language change may simultaneously impact the biological and cultural heritage of Amazonia, we started by compiling 90,536 reports of services provided by Amazonian plants to people from 700 references published between 1504 and 2023 (Fig. 1b and Supplementary Table 1). We found that Amazonian societies use at least 5,796 plant species, which represents a twofold increase in the number of species relative to previous reviews^16^. Of these species, 5,679 are vascular seed plant species and 117 are ferns. This amounts to 36% of the currently known seed plant flora in Amazonia, which we estimated at 15,585 species (see Methods; Supplementary Table 2). Utilized species belong to 195 plant families and 1,284 genera. The five families with the greatest number of utilized species are: Fabaceae (662 species), Rubiaceae (339 species), Melastomataceae (214 species), Annonaceae (182 species) and Lauraceae (176 species). The top five genera for utilized species are *Miconia* (125 species), *Piper* (109 species), *Inga* (92 species), *Pouteria* (71 species) and *Solanum* (63 species). The five most frequently cited species are all palms: *Bactris gasipaes* (cited in 178 references and 58 Indigenous groups), *Oenocarpus bataua* (176 references and 60 Indigenous groups), *Mauritia flexuosa* (165 references and 66 Indigenous groups), *Euterpe precatoria* (148 references and 65 Indigenous groups) and *Iriartea deltoidea* (120 references and 40 Indigenous groups). Human use of neotropical palms dates to at least 14,700 bc and became particularly widespread after 9,000 bc (ref. ^17^). Today, palms remain central to food security and cultural practices in Amazonia^18^.

**Extended Data Fig. 1:**
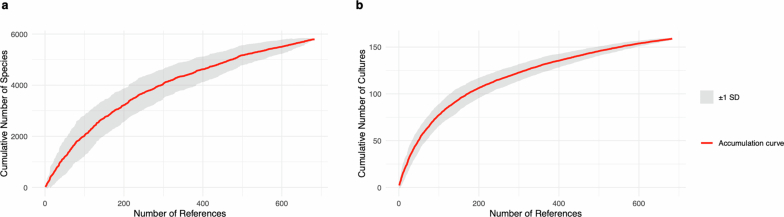
Ethnobotanical documentation shows no evidence of saturation in Amazonia. a, Cumulative number of species vs number of references. b, Cumulative number of cultures vs number of references. Shaded bands indicate ±1 standard deviation from randomized accumulation order.

Amazonian ethnobotanists have centred their efforts on Indigenous peoples (55% of reports). Indigenous cultures report four times more utilized species than non-Indigenous cultures (4,305 versus 1,012 species, respectively). Of the 400 Indigenous cultures recognized in Amazonia^2^, the analysed literature contains information for 243 (48 extinct) that speak 156 living languages (55% threatened). Five cultures harbour 47% of all use reports (Kichwa, 17%; Waorani, 11%; Secoya, 8%; Cofán, 7%; and Shuar, 4%). Of note, 74% of plant services are associated with a single culture and are thus strongly localized. Most culturally unique services are from references published in the twenty-first and twentieth centuries (69% and 24%, respectively) and 60% are exclusive to languages that are threatened according to Ethnologue (99% according to Glottolog). This suggests that many unique services reflect documentation of vulnerable cultural knowledge rather than exclusively historical practices. Research on Amazonian plants is biased to their medicinal properties. Medicinal uses have nearly three times the number of reports as human food uses (33,245, versus 12,333), twice as many species (3,862 versus 1,804) and 57% of all unique reports. Overall, the most prolific Amazonian ethnobotanists were the late Richard E. Schultes—who over five decades recorded 999 utilized species—and the still active Carlos Cerón (1,061 species). Ecuador is the best-documented country, with almost twice the number of literature reports recorded for Brazil, the next best-studied country. Still, despite centuries of research, documentation efforts have not saturated in Amazonia: more utilized species remain to be added to the scientific record and 50% of Amazonian Indigenous groups are missing in the literature (Extended Data Fig. 1). As most Amazonian languages are threatened and most knowledge is linguistically unique, substantial amounts of knowledge on the richest flora of the world could be irretrievably lost unless oral transmission is sustained or documentation efforts increase.

## Regional climate change impacts

**Fig. 2:**
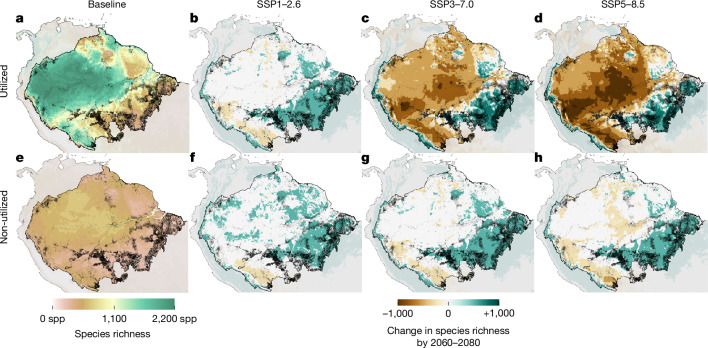
Climate change impacts will be more severe for plant species used by people. **a**–**h**, Baseline (**a**,**e**) and future change predictions (**b**–**d**,**f**–**h**) in the spatial distribution of utilized (**a**–**d**) and non-utilized (**e**–**h**) plant species richness. Future change predictions show the median across five GCMs for the period 2060–2080 and three SSPs: SSP1–2.6, a policy scenario that achieves climate targets by mid-century (**b**,**f**); SSP3–7.0, a scenario with minimal action on climate (**c**,**g**); and SSP5–8.5, a worst-case no-policy scenario with no action on climate mitigation (**d**,**h**). Plant range models selected for mapping had a minimum of 20 unique occurrence records after cleaning and spatial thinning and a continuous Boyce index value > 0.5.

**Extended Data Fig. 2:**
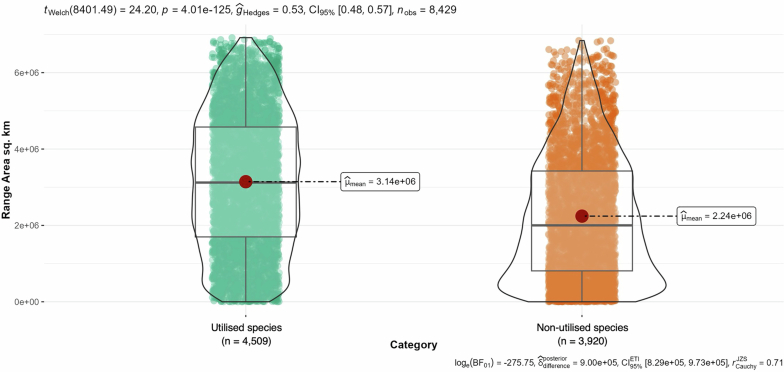
Utilised plant species (left) have larger baseline ranges than non-utilised species (right). Boxplots show the median (center line), interquartile range (box; 25^th^–75^th^ percentiles), and whiskers extending to 1.5× the interquartile range; points beyond this range are shown as outliers. Statistical comparisons between groups were performed using two-sided Student’s t-tests. Results were consistent with nonparametric Wilcoxon rank-sum tests. No adjustment for multiple comparisons was applied.

**Extended Data Fig. 3:**
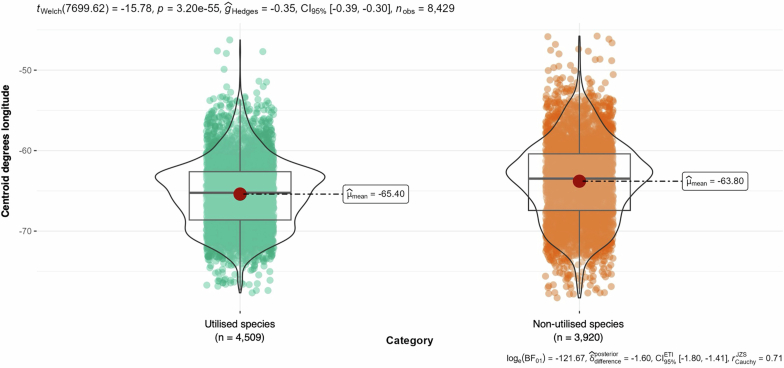
Utilised plant species (left) have significantly different centroid longitudes than non-utilised species (right). Boxplots show the median (center line), interquartile range (box; 25^th^–75^th^ percentiles), and whiskers extending to 1.5× the interquartile range; points beyond this range are shown as outliers. Statistical comparisons between groups were performed using two-sided Student’s t-tests. Results were consistent with nonparametric Wilcoxon rank-sum tests. No adjustment for multiple comparisons was applied.

**Extended Data Fig. 4:**
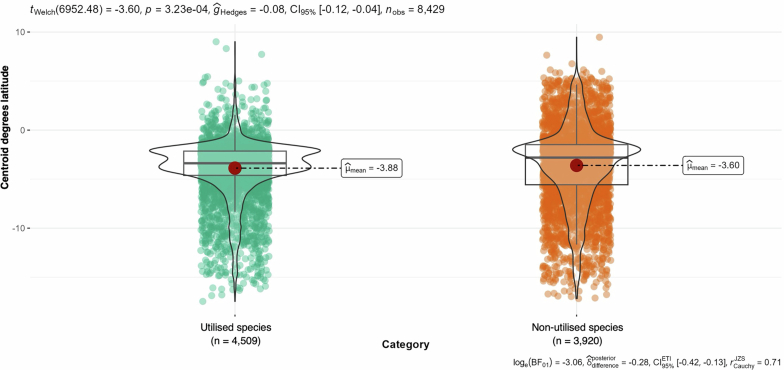
Utilised plant species (left) have significantly different centroid latitudes than non-utilised species (right). Boxplots show the median (center line), interquartile range (box; 25^th^–75^th^ percentiles), and whiskers extending to 1.5× the interquartile range; points beyond this range are shown as outliers. Statistical comparisons between groups were performed using two-sided Student’s t-tests. Results were consistent with nonparametric Wilcoxon rank-sum tests. No adjustment for multiple comparisons was applied.

**Extended Data Fig. 5:**
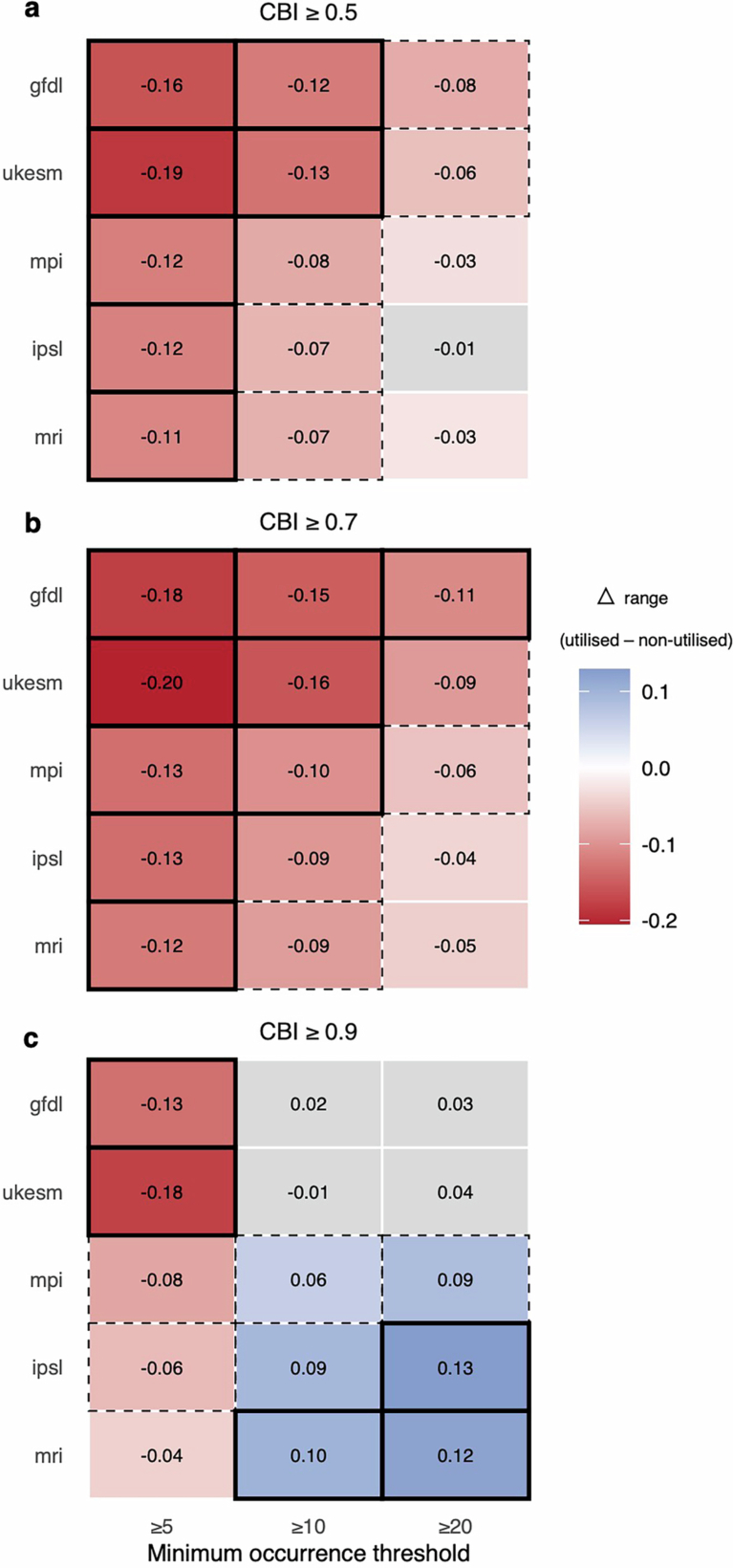
Differences in proportional future range change between utilised vs non-utilised species. Mean differences in proportional future range change (Δ = utilised − non-utilised species) averaged across SSPs. Results are shown for three Continuous Boyce Index thresholds: a, CBI ≥ 0.5. b, CBI ≥ 0.7. c, CBI ≥ 0.9. Columns represents different occurrence thresholds (≥ 5, ≥10, ≥20). Rows show values for five general circulation models (GCM) sorted from top to bottom according to their ISIMIP3 priority ranking: gfdl, ukesm, mpi, ipsl, and mri. Colors indicate the direction and magnitude of change, with red tones denoting larger range contractions in utilised species relative to non-utilised species, and blue tones indicating smaller range contractions in utilised species relative to non-utilised species. Grey tiles denote non-significant differences based on bootstrap confidence intervals across SSPs. Numeric labels report mean change values between utilised and non-utilised species for each model–threshold combination. Solid and dashed outlines highlight strong ((|Δ| ≥0.10)) and moderate ((|Δ| ≥0.05)) effect sizes, respectively.

**Extended Data Fig. 6:**
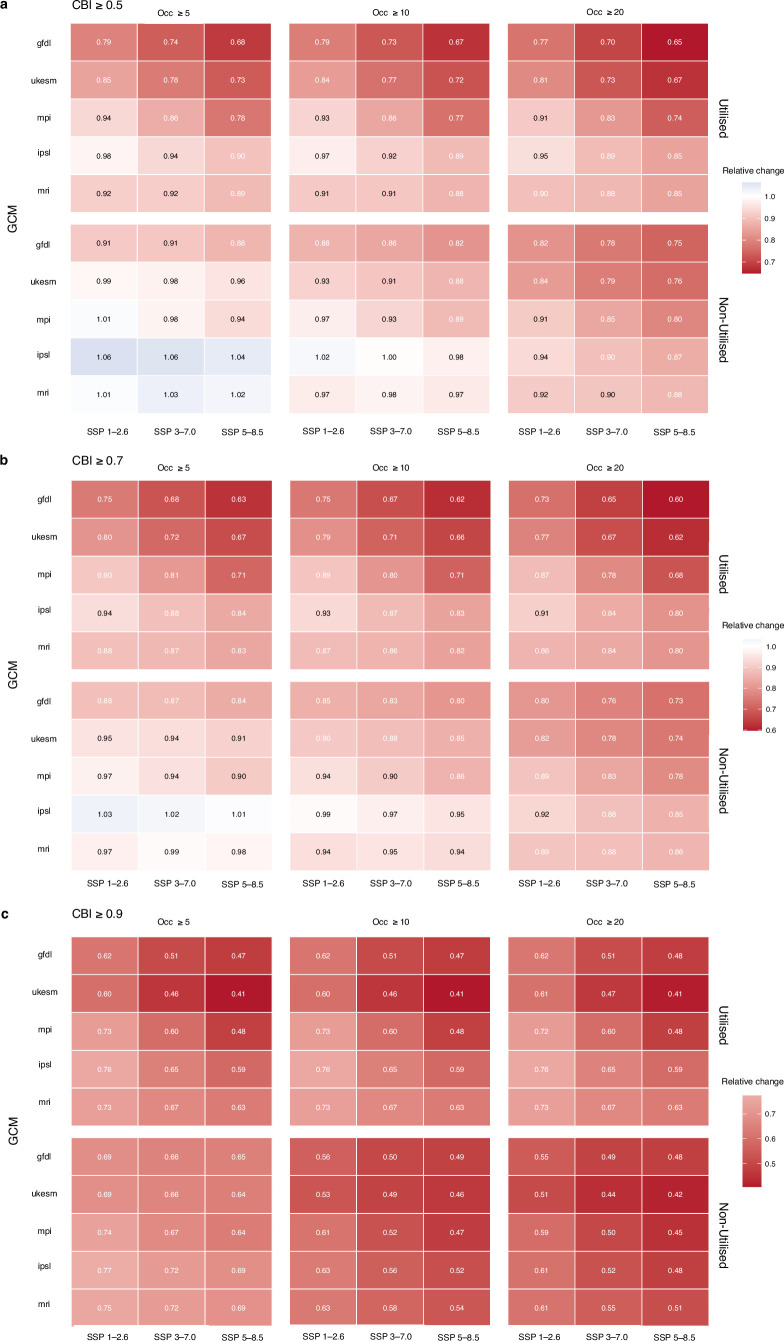
Sensitivity of utilised and non-utilised species’ projected relative range changes to thresholds in general circulation models, emission scenarios, Continuous Boyce Index, and occurrences. Heat maps show the mean relative change in species’ projected range size, calculated as the ratio of future to current range area, across three emissions scenarios (SSP1–2.6, SSP3–7.0 and SSP5–8.5) and five general circulation models (GCM: gfdl, ukesm, mpi, ipsl, and mri). GCMs are ordered from top to bottom according to their ISIMIP3 priority ranking. Values are averaged across species and shown separately for utilised and non-utilised species (rows), and for three minimum occurrence thresholds used to define species’ current distributions (≥5, ≥10, ≥20; columns). Panels correspond to alternative climatic suitability cut-offs used to define suitable habitat: (a) CBI ≥ 0.5; (b) CBI ≥ 0.7; (c) CBI ≥ 0.9. Colors indicate relative range change: blue denoting gains, red denoting losses, and white indicating no net change (value = 1). Numeric labels report mean relative change for each model–scenario combination (e.g., 0.75 indicates an average range contraction of 25%, whereas 1.25 indicates an average expansion of 25%).

To assess to what degree climate change may impact the geographical range of plant species that matter to people, we built species distribution models (SDMs; Supplementary Note 1) for 4,509 utilized species (78% of the species in our database) and for 3,920 non-utilized species (39% of the non-utilized species in the Amazon flora). We found two significant differences in their current distribution ranges. First, utilized plants have larger baseline ranges than non-utilized plants (*P* = 4.01 × 10^−125^; Extended Data Fig. 2). Assessing whether the larger baseline ranges of utilized plants are a legacy of historical human management or whether humans preferentially targeted species with naturally larger ranges would require data that are currently unavailable^19,20^. Second, utilized plants have centroids that significantly differ in longitude (*P* = 3.20 × 10^−55^) and latitude (*P* = 3.23 × 10^−4^) from those of non-utilized plants (Extended Data Figs. 3 and 4). Next, we compared mean changes between utilized and non-utilized species by projecting SDMs for 2060–2080 under five general circulation models (GCMs; gfdl, ukesm, mpi, ipsl and mri) and three shared socioeconomic pathways (SSPs; 1–2.6, 3–7.0 and 5–8.5). Across most SSPs and GCMs, plant species associated with human use are projected to experience systematically greater range contractions than non-utilized species (Fig. 2 and Extended Data Figs. 5 and 6). Together, these results demonstrate that focusing specifically on utilized plants is crucial. Although macroecological studies often analyse all plants together, our findings show that climate change will impact species that are important to people in distinct and potentially more severe ways.

**Extended Data Fig. 7:**
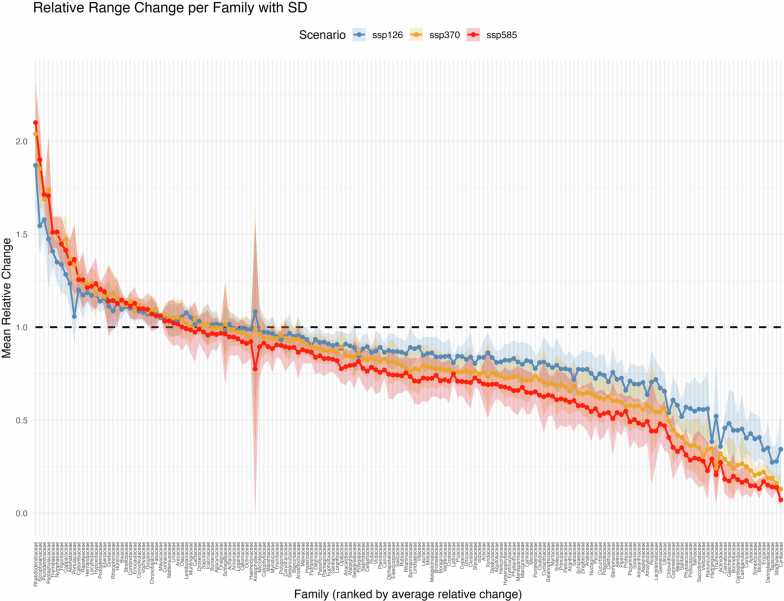
Family-level patterns in projected range change for Amazonian utilised plant species. Mean relative change in range area (future/baseline) for utilised plant species, aggregated at the family level and averaged across general circulation models (GCMs). Families are ranked along the x-axis by their average relative change across all scenarios. Coloured lines and points show mean relative range change under three future climate scenarios (SSP1–2.6, SSP3–7.0 and SSP5–8.5), while shaded bands indicate ±1 s.d. across GCMs. The dashed black line shows the baseline (present climate), corresponding to a relative change of 1 for all species. Sample sizes (n) indicate the number of species per family included in the analysis: Achariaceae (n = 7), Acanthaceae (n = 22), Actinidiaceae (n = 2), Alismataceae (n = 1), Amaranthaceae (n = 8), Amaryllidaceae (n = 4), Anacardiaceae (n = 19), Anisophylleaceae (n = 1), Annonaceae (n = 146), Apiaceae (n = 1), Apocynaceae (n = 133), Aquifoliaceae (n = 6), Araceae (n = 117), Araliaceae (n = 11), Aristolochiaceae (n = 13), Aspleniaceae (n = 9), Arecaceae (n = 124), Asteraceae (n = 57), Balanophoraceae (n = 3), Begoniaceae (n = 6), Bignoniaceae (n = 88), Bixaceae (n = 5), Bonnetiaceae (n = 2), Boraginaceae (n = 25), Bromeliaceae (n = 22), Burmanniaceae (n = 2), Burseraceae (n = 61), Cabombaceae (n = 1), Calophyllaceae (n = 17), Campanulaceae (n = 3), Cannabaceae (n = 3), Cannaceae (n = 2), Capparaceae (n = 3), Cardiopteridaceae (n = 2), Caricaceae (n = 4), Caryocaraceae (n = 11), Celastraceae (n = 28), Chloranthaceae (n = 2), Chrysobalanaceae (n = 71), Cleomaceae (n = 3), Clethraceae (n = 1), Clusiaceae (n = 60), Combretaceae (n = 27), Commelinaceae (n = 11), Connaraceae (n = 16), Convolvulaceae (n = 18), Costaceae (n = 15), Cucurbitaceae (n = 24), Cyperaceae (n = 33), Cyrillaceae (n = 1), Dennstaedtiaceae (n = 1), Dichapetalaceae (n = 10), Dilleniaceae (n = 19), Droseraceae (n = 1), Ebenaceae (n = 1), Elaeocarpaceae (n = 20), Eriocaulaceae (n = 3), Ericaceae (n = 2), Erythroxylaceae (n = 15), Euphorbiaceae (n = 105), Fabaceae (n = 483), Gentianaceae (n = 21), Gesneriaceae (n = 39), Gleicheniaceae (n = 2), Gnetaceae (n = 5), Goupiaceae (n = 1), Haemodoraceae (n = 2), Heliconiaceae (n = 25), Hernandiaceae (n = 4), Humiriaceae (n = 16), Hydrangeaceae (n = 1), Hypericaceae (n = 16), Hymenophyllaceae (n = 7), Hypoxidaceae (n = 1), Icacinaceae (n = 3), Iridaceae (n = 2), Lacistemataceae (n = 4), Lamiaceae (n = 35), Lauraceae (n = 132), Lecythidaceae (n = 63), Lentibulariaceae (n = 3), Linaceae (n = 3), Lindsaeaceae (n = 2), Loganiaceae (n = 29), Loranthaceae (n = 16), Lythraceae (n = 7), Malpighiaceae (n = 59), Malvaceae (n = 114), Marantaceae (n = 30), Marcgraviaceae (n = 9), Mayacaceae (n = 1), Melastomataceae (n = 174), Meliaceae (n = 48), Menispermaceae (n = 34), Metteniusaceae (n = 10), Microteaceae (n = 1), Monimiaceae (n = 3), Moraceae (n = 104), Muntingiaceae (n = 1), Myristicaceae (n = 42), Myrtaceae (n = 44), Nymphaeaceae (n = 4), Ochnaceae (n = 22), Olacaceae (n = 18), Onagraceae (n = 10), Opiliaceae (n = 2), Oxalidaceae (n = 6), Passifloraceae (n = 38), Pentaphylacaceae (n = 3), Peraceae (n = 6), Petiveriaceae (n = 5), Phyllanthaceae (n = 17), Phytolaccaceae (n = 3), Picramniaceae (n = 7), Picrodendraceae (n = 1), Piperaceae (n = 79), Plantaginaceae (n = 3), Poaceae (n = 70), Podocarpaceae (n = 3), Podostemaceae (n = 2), Polygonaceae (n = 21), Polypodiaceae (n = 29), Pontederiaceae (n = 3), Portulacaceae (n = 3), Primulaceae (n = 23), Proteaceae (n = 5), Putranjivaceae (n = 2), Ranunculaceae (n = 1), Rapateaceae (n = 4), Rhabdodendraceae (n = 1), Rhamnaceae (n = 13), Rhizophoraceae (n = 4), Rosaceae (n = 6), Rubiaceae (n = 257), Rutaceae (n = 31), Sabiaceae (n = 4), Saccolomataceae (n = 1), Salicaceae (n = 36), Santalaceae (n = 8), Sapindaceae (n = 94), Sapotaceae (n = 43), Schizaeaceae (n = 3), Schlegeliaceae (n = 2), Scrophulariaceae (n = 1), Selaginellaceae (n = 12), Simaroubaceae (n = 9), Siparunaceae (n = 18), Smilacaceae (n = 7), Solanaceae (n = 90), Staphyleaceae (n = 1), Stemonuraceae (n = 1), Strelitziaceae (n = 1), Styracaceae (n = 4), Symplocaceae (n = 1), Talinaceae (n = 1), Tapisciaceae (n = 1), Thurniaceae (n = 1), Thymelaeaceae (n = 5), Triuridaceae (n = 1), Trigoniaceae (n = 2), Urticaceae (n = 57), Ulmaceae (n = 2), Verbenaceae (n = 17), Violaceae (n = 16), Vitaceae (n = 7), Vochysiaceae (n = 40), Xyridaceae (n = 3), and Zingiberaceae (n = 10).

**Extended Data Fig. 8:**
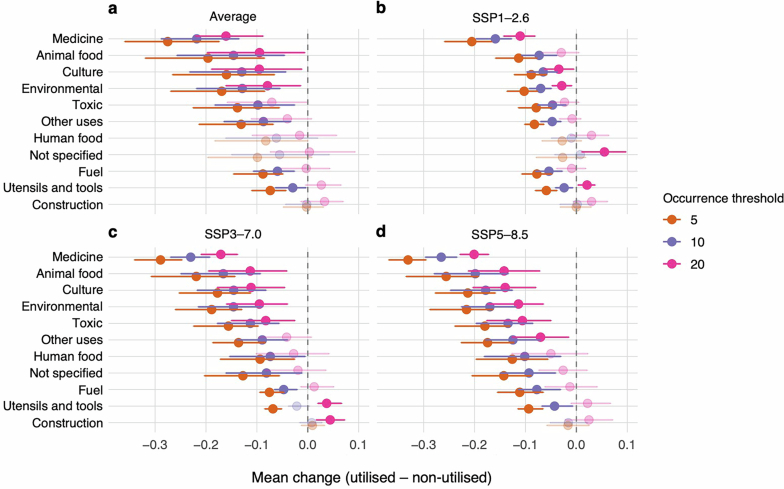
Projected climate-driven range changes of utilised plants by use category. Mean changes in projected range size (2060–2080 vs present) between non-utilised (n = 3,920) and utilized species (n = 4,509) grouped by use category. a, average across SSPs. b, SSP1–2.6. c, SSP3–7.0. d, SSP5–8.5. Results are shown for a Continuous Boyce index (CBI)** ≥** 0.7 and for different occurrence thresholds: ≥ 5 (orange), ≥ 10 (purple), and ≥ 20 (pink). Points indicate mean change per use category; error bars are 95% bootstrap confidence intervals. Non-significant changes are semi-transparent; significant changes are opaque. Categories are ordered by mean decrease, with the most negatively affected at the top (e.g., Medicine). Sample sizes (n = number of species) refer to the number of biologically independent species included after filtering for CBI** ≥** 0.7 and each occurrence threshold. For occurrence ≥ 5, sample sizes per use category were: Medicine, n = 1238; AnimalFood, n = 555; Culture, n = 445; Environmental, n = 208; Toxic, n = 173; OtherUses, n = 27; HumanFood, n = 558; NotSpecified, n = 6; Fuel, n = 356; UtensilsAndTools, n = 448; Construction, n = 555. See Extended Data Table 1 for sample sizes in the other occurrence thresholds).

To understand how climate change may differentially impact the societal benefits derived from utilized plants, we examined projected range changes across plant lineages, use categories and gradients of cultural breadth (see Methods). Across plant lineages, projected range contractions are expected for 72% of plant families and 68% of genera. However, some utilized families and genera depart from this overall trend (Extended Data Fig. 7 and Supplementary Fig. 1), indicating taxonomic heterogeneity in climate change impacts across the tree of life. Across plant use categories, significant declines are predicted for most uses, with species used for medicine particularly affected (Extended Data Fig. 8). This underscores the potential for climate change to disproportionately threaten species underpinning human health systems. Finally, across gradients of cultural breadth, species supporting human cultural diversity are projected to experience range contractions regardless of whether their uses are unique to a single culture (*n* = 3,038), shared among a limited number of cultures (*n* = 2,544) or widespread across multiple cultures (*n* = 221; Supplementary Table 3). By highlighting the vulnerability of medicinal species and the widespread exposure of culturally important plants to climate-driven range loss, our study underscores the importance of integrating human needs into conservation planning. Greater attention to utilized species is therefore essential not only for biodiversity and ecosystem integrity but also for sustaining the cultural practices through which these species are maintained.

## Local climate change impacts

**Fig. 3:**
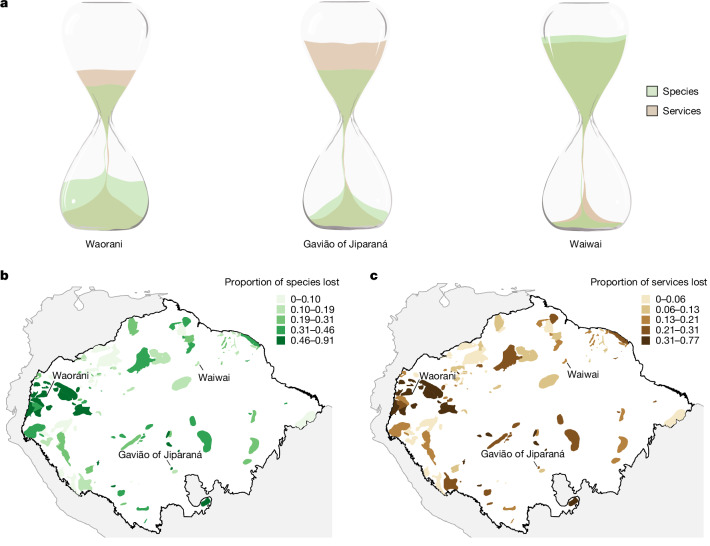
Climate change threatens cultural heritage. **a**, Sand clocks show the predicted local extinction of plant services owing to the local extinction of plant species from climate change. Three examples are shown to illustrate that local extinction of plant species and services varies across cultures. **b**,**c**, Proportion of plant species (**b**) or plant services (**c**) that are predicted to be lost within 82 Amazonian language polygons (sensu Ethnologue^22^) owing to climate change by 2060–2080. Illustration in (**a**) created by Inés Cámara Leret.

Having assessed regional climate change effects on utilized species across taxonomic lineages, use categories and cultural breadth classes, we then examined its effects at the local scale that matters to particular Indigenous cultures^21^. Most cultures speak a distinct language and inhabit a particular territory, which linguists have mapped as language polygons^22^. Because many Indigenous languages are ethnobotanically under-documented, here we only focused on 82 languages with at least 10 reported useful species in the literature (range of 10–1,197 species, mean of 130 and s.d. of 201). For each language, we built an Indigenous knowledge network^23^ to relate individual plant species (nodes in one set) to particular services (nodes on the other set) based on the knowledge (links) held by speakers of that language (see Methods). Next, we quantified the climate change exposure of each Indigenous knowledge network by first calculating the proportion of utilized species predicted to be lost within each language polygon for the period 2060–2080. We found that, on average, projected local extinctions of plant species increase with climate severity, from 28% (range of 25–32%, s.d. of 3%) under SSP1–2.6, to 30% (range of 25–35%, s.d. of 5%) under SSP3–7.0, and 34% (range of 27–39%, s.d. of 6%) under SSP5–8.5. We then evaluated how these projected species losses translate into reductions in plant services across each network. Plant service losses track species trends, rising from 18% to 23% across the same scenarios (Fig. 3). The proportion of plant services lost will vary among cultures, even for cultures losing a similar fraction of species. Such loss is uncorrelated to the geographical longitude of languages, indicating that the biocultural impacts of climate change will be felt across the entire Amazon basin. Still, regions with threatened languages are projected to experience higher losses of plant species (mean ± s.d.: 39% ± 27% versus 25% ± 20%, Wilcoxon *W* = 863, *P* = 0.0043) and services (27% ± 20% versus 17% ± 15%, *W* = 824, *P* = 0.0018), highlighting the vulnerability of these cultures to climate change.

## Language change impacts

So far, we have focused on the biocultural loss triggered by the extinction of plants, but the extinction of culture also matters. Indeed, cultural loss may undermine Indigenous knowledge networks at least as fast as biological loss^23^. Specifically, language extinction may contribute to the loss of medicinal knowledge, as much of this knowledge is associated with a single language^24^. Although plant use knowledge may, in some cases, persist across languages or cultural contexts, we assumed a worst-case scenario in which language loss corresponds to knowledge loss. To assess how language extinction may impact Amazonian knowledge, we first built what we refer to as the Indigenous knowledge metaweb^23^ (that is, the aggregated network resulting from adding all Indigenous and non-Indigenous knowledge networks, which represents the total knowledge about the services provided by all plant species in the study area). We then obtained linguistic threat information for all the languages in our sample from Ethnologue—the most comprehensive catalogue of the languages of the world^22^—and estimated the effect of language loss on the erosion of the Indigenous knowledge metaweb. From the 76,390 literature reports of native plant species, 57% are from 156 Indigenous languages, of which 56% are threatened. By contrast, the remaining 44% arise from reports from non-specified languages whose threat status is unknown (82% of the 44%) or from non-Indigenous languages (for example, 11% Portuguese and 7% Spanish). Accordingly, if threatened Indigenous languages vanish by the end of the century as predicted by linguists^22^, the Amazonian metaweb would shrink by 26%. And yet, this estimate is conservative for at least three reasons. First, our sample includes nearly one-third of Amazonian languages. Because most of the non-sampled cultures speak threatened languages, including those in the analyses would further shrink the metaweb. Second, our threat classification based on the Ethnologue conservatively classifies 56% of Indigenous languages in our sample as threatened, compared with 97% by Glottolog^25^. Third, we classified all reports from non-specified languages as not threatened, but most Amazonian languages are threatened. Thus, had we classified non-specified language reports as belonging to threatened languages, the metaweb would shrink by 61%.

## Safeguarding the forest of knowledge

Here we have shown that the ethnobotanical knowledge of Amazonian peoples spans at least 5,796 native plant species, that utilized species may follow different climate change trajectories than the rest of plant species, and that 28–34% of utilized species and 18–23% of their associated services may become locally extinct by 2060–2080. This massive climate reshuffling of plants and associated services will have critical repercussions for Amazonian societies. On the one hand, our findings are a conservative best-case scenario, as we do not consider synergies with other global change drivers such as deforestation, fire or mining—three major threats to the biocultural heritage of Amazonia^26–28^. We also did not consider extreme events triggered by climate change, which can alter phenological cycles and productivity well before local extinction occurs. For example, the 2024 extreme drought led to losses in 2025 for the Brazil nut (*Bertholletia excelsa*) harvest, with basin-wide effects on food security from the Karipuna Indigenous land in Rondônia to the Cajarí Extractivist reserve in Amapá^29^. In addition, vegetation types in Amazonia can vary over just a few kilometres—from floodplain to terra firma to white sand forests—but we did not include vegetation type in SDM buffers as no basin-wide vegetation map yet exists. Incorporating such data would probably yield more conservative estimates by constraining species to specific habitats and thus strengthen the overall trend reported here. On the other hand, our findings did not consider microclimatic data, which are currently unavailable across Amazonia. In temperate systems, incorporating microclimate has been shown to both amplify and reduce the disequilibrium between community responses and macroclimate change^30^. Moreover, macroclimate-based SDMs can overestimate the potential latitudinal range limits of temperate species, producing wider niches for minimum and mean temperatures and narrower niches for maximum temperatures^31^. This suggests that projections based solely on macroclimate may overestimate exposure to warming, particularly for understorey species. And yet, studies in tropical lowland forests have indicated that climate change is advancing faster than canopy species can probably track and that acclimation seems improbable^32^. As a result, the effect of fine-scale microclimatic variation on long-term persistence of forest understorey species remains uncertain. Although the relative differences between utilized and non-utilized species are probably robust to algorithm choice, we note that reliance on a single algorithm (Maxent) introduces uncertainty in the magnitude of projected range shifts^33^. Finally, our predicted range changes also do not account for human management. Although human-assisted translocations may mitigate the impacts of global change for a small subset of species (for example, edible palms), such interventions are unlikely for most species, including thousands of shade-loving forest trees and many poorly studied herbs and lianas.

Hundreds of cultures and their languages have disappeared owing to post-contact disease epidemics, slavery and violence^34^. As a result, their knowledge on the use and management of the most biodiverse forest of the world has been forever lost. All cultures with extinct languages in our review have superficial documentation. Today, as languages continue to erode^35^, much of the recorded knowledge will not be passed to the next generation, limiting the future wellbeing of the inhabitants of the region. Our results strongly hint to the possibility that the climate tipping point for Amazonia^36^ will not only impact ecosystem and biological diversity but also interact with language threat and cascade across the unique cultural heritage of the biome. Our publicly available dataset of more than 76,000 literature reports, in turn, represents a critical resource for scientists, policy makers, non-governmental organizations, and Indigenous and local communities restoring biocultural heritage in the Amazon (http://www.the-forest-of-knowledge.com). Although our analysis captures the breadth of documented biocultural knowledge, it does not distinguish between historically documented and currently practiced uses and therefore should not necessarily be taken as a measure of present-day societal benefits. Future work integrating temporal persistence and cultural continuity with the ecological traits of species will be key to assessing human relevance under global change.

Our results can inform policy in at least three ways. First, our predictions of climate and language change effects on both biological and cultural heritage call for a better coupling of these dimensions in science and policy. Thus, addressing the entire socioenvironmental sphere, rather than its parts in isolation, is necessary. One way to implement this is via multidisciplinary and intercultural efforts such as the Science Panel for the Amazon, whose main role is to integrate Indigenous and local knowledge with Western science to achieve evidence-based solutions for sustainable development. Second, our finding that climate change is all-enveloping and threatens all cultures calls for Pan-Amazonian solutions. A shared and long-term vision for effective cooperation and sustained common action among Amazonian states exists (that is, the Amazon Cooperation Treaty Organization), but its implementation remains challenging. This is especially critical in remote areas where Indigenous and local communities are most vulnerable to illegal actors that trespass institutional and security voids. Finally, retaining primary tropical forests and their carbon offset benefits requires long-term financial, environmental and social commitments from parties to the Intergovernmental Panel on Climate Change. Our synthesis of thousands of edible and medicinal species can catalyse enterprises that foster a more diverse Amazonian ‘sociobioeconomy’ beyond monocultures, that is not exclusively commercial, but rather strengthens cultural foundations too. Emphasis on the ‘socio’ in sociobioeconomy—as articulated in the Pan-Amazonian Pact for Climate—will be key to fostering initiatives that genuinely respect Indigenous and local knowledge, align with community aspirations and safeguard local wellbeing in accordance with principles of justice, equity and diversity.

## Methods

### Study area

We delimited our study area of the Amazon basin following the biogeographical limits proposed by the Amazon Network of Georeferenced Socio-Environmental Information (RAISG)^37^.

### Plants used in Amazonia

To understand how climate change may affect the biocultural heritage of Amazonia, we first compiled bibliographic information on the plants recorded as utilized by Indigenous and non-Indigenous local communities (Supplementary Table 1). We gathered five different types of information: (1) regional compilations for the Amazon basin^16^, Northwest Amazonia^13^ and the Guianas^38^; (2) country-level compilations, which exist for Ecuador^39^, Peru^40^ and Brazil^41^; (3) monographs on individual Indigenous groups (for example, refs. ^42–68^); (4) specific plant services, such as food (for example, refs. ^69–71^) and medicine (for example, refs. ^72–79^); and (5) early historical accounts, going back to the sixteenth century (for example, refs. ^80–84^). We defined a ‘use report’ as the concatenation of a plant species with plant part, use category and use subcategory (for example, *Clusia schultesii* Maguire + entire leaf + medicine + skin and subcutaneous tissue). We classified each use report as either ‘unique’ or ‘shared’ based on the cultural groups that cited it. Use reports linked to a single language or a single specified cultural group were considered unique, whereas those cited by multiple languages or groups—including cases labelled ‘not specified, but more than one’, or ‘not specified’ occurring across multiple countries or references—were classified as shared. Together, our review represents the most comprehensive ethnobotanical assessment in Amazonia, covering 50% of its Indigenous cultures. As more field ethnobotanical studies are conducted, the number of studied cultures will continue to increase, as will the number of utilized species, because the ethnobotany of many Amazonian Indigenous groups remains superficially studied^85^. To address the latter, we focused our local-scale analyses on only the best-studied cultures (see below).

### Harmonization of scientific names

Three steps were followed to harmonize the scientific names of utilized species and to confirm their native status in Amazonia: first, a checklist of Amazonian vascular seed plants was built (hereafter ‘Amazon checklist’) by combining the tree species of ter Steege et al.^15^ and the non-tree species of Cardoso et al.^14^ (‘tree.10 cm.DBH = no’). After removing duplicates (*n* = 1,123), there were 16,219 vascular seed plant species. These were reviewed using the R package rWCVP (v1.2.4)^86^, yielding 14,293 accepted names. Second, all non-accepted names from the rWCVP analysis (16,219 − 14,293 = 1,926; that is, illegitimate, invalid, misapplied, orthographic, synonym, unplaced and not available (NA)) were checked against Plants of the World Online (POWO; https://www.plantsoftheworldonline.org) and the online Catalogue of Vascular Plants of the Americas (VPA)^87^. This verification resulted in two lists, depending on the online synonymy portal that was consulted: POWO (15,638 species) or VPA (15,806 species). Third, as the Amazon checklist only includes seed plants, the utilized species list (hereafter ‘ethnobotany list’) was filtered for seed plants. This filtered list was then compared with the POWO (89% match rate) and VPA (90%) lists. All non-matching names were double checked by consulting online type specimens at JSTOR Global Plants (https://plants.jstor.org), specimen occurrences at the Global Biodiversity Information Facility (GBIF; https://www.gbif.org) and synonymy in the VPA and POWO online portals. Most of the species missed in the ethnobotany list (82 of 87 species) were added to it, except for five non-native species: *Colocasia esculenta* (L.) Schott, *Cyperus rotundus* L., *Nymphoides indica* (L.) Kuntze, *Portulaca oleracea* L. and *Psidium guajava* L. Of the names in the ethnobotany list not listed in the Amazon checklist, 407 were native species missed by the Amazon checklist (and added to it), 153 were synonymy variants present in both lists and 145 were non-native species (removed from the ethnobotany list). Ultimately, the Amazon seed plant checklist (Supplementary Table 2) had 15,585 or 15,749 species according to POWO and VPA, respectively, whereas the utilized seed plant list had 5,679 species. These totals were used to calculate the proportion of utilized species in the Amazon seed plant flora. Non-seed plant names were checked against World Ferns, the most up-to-date synonymic checklist of the ferns and lycophytes of the world^88^.

### Indigenous language names

Indigenous group names and their spoken languages and geographical coordinates were verified using Ethnologue^22^ and Glottolog^25^. Classification of language endangerment followed the Ethnologue and was compared with Glottolog as the latter tends to classify more languages as endangered. The Ethnologue classifies language endangerment based on the Expanded Graded Intergenerational Disruption Scale^89^. The languages in our sample were classified into 11 of the 13 levels of the Expanded Graded Intergenerational Disruption Scale: national, wider communication, educational, developing, vigorous, threatened, shifting, moribund, nearly extinct, dormant and extinct. Of these levels, the first five are not endangered. The Glottolog uses the agglomerated endangerment status (derived from The Catalog of Endangered Languages, UNESCO Atlas of the World’s Languages in Danger and Ethnologue) and has six levels: not endangered, threatened, shifting, moribund, nearly extinct and extinct.

### Species range models

All SDMs were implemented through the R package wallace 2 (ref. ^90^) (Supplementary Note 1). We obtained primary species occurrence records through automated programmatic queries to the Botanical Information and Ecology Network (BIEN) using the R packages BIEN (v1.2.8)^91^ and spocc (v1.2.4)^92^, which provides a unified interface to major biodiversity repositories including BIEN, GBIF, VertNet, Ocean Biodiversity Information System (OBIS) and others. For each species in the study list, we executed a standardized observation record retrieval routine in wallace 2, specifying BIEN as the source database. BIEN aggregates curated plant occurrence records from herbaria, plot networks and digitization initiatives worldwide, and supports taxonomically resolved queries suitable for large-scale biodiversity analyses^92^. Returned records were parsed and cleaned automatically by the wallace 2 internal cleaning step, which removes malformed coordinates, impossible values and duplicated entries. For transparency and reproducibility, all cleaned occurrence tables were exported as comma-separated values files and stored by species. SDMs were produced for species with more than ten unique occurrence records in the list of Amazonian useful plants. In addition, modelled plant species with more than 20% of the total modelled range in the Amazon basin were selected as the control group^25^. The final number of species used in this analysis was 4,509 Amazonian utilized plant species and 3,920 control group species, for a combined total of 8,429 species.

We used a subset of CHELSA baseline bioclimatic variables at 5 arcminute (approximately 10 km) spatial resolution as predictors (bio1–mean annual temperature, bio2–temperature seasonality, bio5–maximum annual monthly temperature, bio6–minimum annual monthly temperature, bio12–mean annual precipitation and bio15–precipitation seasonality)^93^. For each species, environmental values were extracted at occurrence locations and duplicated or NA-containing environmental records were removed before modelling. Soil variables used in species distribution models were depth to bedrock, pH, clay proportion, silt proportion and bulk density. All soil-related variables were obtained from SoilGrids (v1.0)^94^. Variables with multiple strata available were the mean of the top 1 m (strata 1–4). Soil variables were included as it has been shown that climate change analyses that do not incorporate soil variability can misrepresent edaphic specialists^95^.

All future environmental variables were obtained for three SSPs that explore possible changes in future energy use, greenhouse gas emissions and temperature^96^. Following the recommendation of ref. ^96^, we not only focused on extreme outcomes but also on more plausible outcomes. Thus, we selected the following SSPs: (1) SSP1.2–6, likely, modest mitigation; (2) SSP3–7.0, unlikely, average no-policy; and (3) SSP5–8.5, highly unlikely, worst-case no-policy. We have presented the results for five GCMs and follow the ISIMIP3 priority ranking (GFDL-ESM4 > UKESM1-0-LL > MPI-ESM1-2-HR > IPSL-CM6A-LR > MRI-ESM2-0), as defined in the ISIMIP3 simulation protocol^97^.

To reduce the effects of spatial sampling bias, occurrence points were spatially thinned at a minimum nearest-neighbour distance of 10 km using the R package spThin^98^. Species-specific background regions were constructed using a 10° buffer around the thinned occurrences, following a point-buffer approach. Environmental layers were masked to this extent, and 10,000 background points were randomly sampled from the masked region. Environmental predictors were subsequently extracted at all background locations and appended to a metadata table for model fitting. To evaluate model performance while accounting for spatial autocorrelation, we used spatial block partitioning. Blocks were generated with an aggregation factor of 2 applied to the masked environmental data. Both occurrence and background points were assigned to partitions for use in cross-validation.

We generated SDMs via the ENMeval v2.0 framework^99^ implemented in wallace 2. Models were fit with the maxnet algorithm with no clamping^100,101^. We tuned models using combinations of linear and quadratic feature classes and regularization multipliers of 1–3. For each species, all parameter combinations were evaluated using spatial block cross-validation. The optimal model was selected based on the highest continuous Boyce index (CBI) value from the test data^102^. The selected model was projected onto current climatic conditions using the cloglog output. To create binary suitable–unsuitable maps, we calculated pixel-level suitability at occurrence locations and applied the 10th percentile training presence threshold. To omit degraded areas from potential areas of distribution, we excluded sites having a human modification index^103^ value greater than 0.1.

We projected the best-performing model to future climate conditions using all GCMs available in CHELSA under SSP1–2.6, SSP3–7.0 and SSP5–8.5 coupled SSP and representative concentration pathway scenarios for the period 2060–2080 (ref. ^93^). Environmental predictors for future scenarios were identical in variable set and naming convention to the baseline models. Model transfers to future climate scenarios were performed with cloglog output and clamping disabled. Transferred predictions were also thresholded to binary maps using the 10th percentile training presence value derived from current-climate predictions to ensure comparability.

### Regional climate change effects on utilized versus non-utilized plant species

To assess how climate change may impact utilized versus non-utilized plant species, we first built SDMs for 4,509 utilized and 3,920 non-utilized species, respectively (see the section ‘Species range models’ above). We compared changes in the average range of utilized versus non-utilized species between the present and 2060–2080 across five GCMs and three SSPs (SSP1–2.6, SSP3–7.0 and SSP5–8.5). For each species, proportional changes were averaged across SSPs, and differences between utilized and non-utilized groups were computed for each model and filtering threshold. For all use categories, the same procedure was applied to quantify differences in mean range change between utilized and non-utilized species within each category. Uncertainty was assessed using non-parametric bootstrap resampling (1,000 iterations), separately for each occurrence and CBI threshold combination, generating 95% confidence intervals. Differences were considered non-significant when confidence intervals overlapped zero, and multiple thresholds of occurrence number (≥5, ≥10 and ≥20) and CBI (≥0.5, ≥0.7 and ≥0.9) were explored to assess robustness.

We summarized projected range changes of utilized plant species across plant lineages (at the family and genus levels) across climate scenarios (Extended Data Fig. 7 and Supplementary Fig. 1). For each genus, we calculated the mean relative range change and standard deviation across GCMs. Relative range change was defined as the projected range size under a future climate scenario divided by the baseline range for the same taxon, averaged across species and GCMs. A value of 1 indicates no change, values below 1 indicate contraction, and values above 1 indicate expansion relative to the baseline. To visualize these patterns, families were plotted in alphabetical order, with genera on the *x* axis and mean relative range change on the *y* axis. Points represent the mean change per genus under each scenario (SSP1–2.6, SSP3–7.0 and SSP5–8.5), and semi-transparent error bars indicate ±1 s.d. across GCMs.

To determine to what degree utilized species underpin human cultural diversity, we devised three cultural breadth classes. (1) Unique species (*n* = 3,038) are those for which all documented services are exclusively cited by a single culture. (2) Shared species (*n* = 2,544) have a mix of services: some are exclusive to one culture, whereas others are cited by multiple cultures. (3) Widespread species (*n* = 221) are those for which all documented services are cited by multiple cultures. Pairwise comparisons of relative range change between species classes were performed using Wilcoxon rank-sum tests (Supplementary Table 3). This approach allowed us to assess whether species with different patterns of cultural use differ in their projected climate change vulnerability.

### Local climate change effects on Indigenous knowledge networks

To understand how climate change may affect individual cultures, we built Indigenous knowledge networks^23^ for the best-studied 82 languages that had at least 10 species reports in the literature. We modelled changes in utilized species and associated services in each network by comparing how many of the current useful species persist in the future within the Ethnologue language polygons associated to each network. This information was then used to calculate to what degree the localized loss of species by 2060–2080 will result in the loss of plant services in each local network using the R package igraph (v2.0.3)^104^. Specifically, for each language polygon, we quantified the proportion of species predicted to be lost under future climate scenarios and then updated the adjacency matrices of the knowledge networks to reflect these local extinctions. As some species may be the only species providing a given service, their loss can lead to the disappearance of services that are not redundant among species. By incorporating these future local species extinctions across language polygons, we assessed the proportion of plant services lost within each knowledge network. Next, we assessed whether predicted climate change effects on Amazonian plant species and plant services differed between languages classified as threatened or non-threatened. For each language, we calculated the mean proportion of plant species predicted to go extinct by 2060–2080 and the mean proportion of services lost (across SSPs and GCMs). Differences between threatened (*n* = 46) and non-threatened (*n* = 36) languages were tested using two-sided Wilcoxon rank-sum tests. Effect sizes are reported as mean ± s.d., along with the Wilcoxon test statistic (*W*) and associated *P* value.

### Indigenous knowledge metaweb

To assess the affects of language extinction across Amazonia, we first built an Indigenous knowledge metaweb that contains the total pool of knowledge in our database. The metaweb included 5,796 species, 717 plant services and 36,504 interactions. Using language threat data from Ethnologue (see above), we calculated the proportion of the metaweb that would remain after all threatened languages were lost. Under this scenario, the metaweb would only retain knowledge exclusively documented in non-threatened Indigenous languages, non-Indigenous languages (Portuguese and Spanish) and in non-specified Indigenous languages, which are assumed to be non-threatened. However, as this assumption is conservative, we repeated the analysis classifying all non-specified Indigenous languages as threatened.

### Reporting summary

Further information on research design is available in the Nature Portfolio Reporting Summary linked to this article.

## Online content

Any methods, additional references, Nature Portfolio reporting summaries, source data, extended data, supplementary information, acknowledgements, peer review information; details of author contributions and competing interests; and statements of data and code availability are available at 10.1038/s41586-026-10741-y.

## Supplementary information

Supplementary Information Supplementary Note 1 containing the ODMAP description of Species Distribution Modelling workflow.

Reporting Summary

Supplementary Figure 1 Genus-level patterns in projected range change for Amazonian utilised plant species. Mean relative change in Amazonian range area (future/baseline) for utilised plant species, aggregated to the genus level and shown by family. Points show genus means under three future climate scenarios (SSP1–2.6, SSP3–7.0 and SSP5–8.5), with error bars indicating ±1 s.d. across general circulation models. The dashed black line shows the family-specific mean of species’ present-climate Amazonian range (averaged across general circulation models), used as the reference for calculating relative range change. Families are displayed across multiple pages, with axes scaled independently to highlight within-family variation. Sample sizes (n = number of species) per genus are shown in parentheses next to genus names.

Supplementary Table 1 List of the 700 references included in the bibliographic review.

Supplementary Table 2 Checklist of Amazonian vascular plants.

Supplementary Table 3 Pairwise Wilcoxon rank-sum tests comparing projected relative range change among culturally widespread, shared, and unique plant species across scenarios, occurrence thresholds (occ), and Continuous Boyce Index (CBI) thresholds. Reported p-values indicate no significant differences (all p > 0.05).

## Figures and Tables

**Extended Data Table 1:**
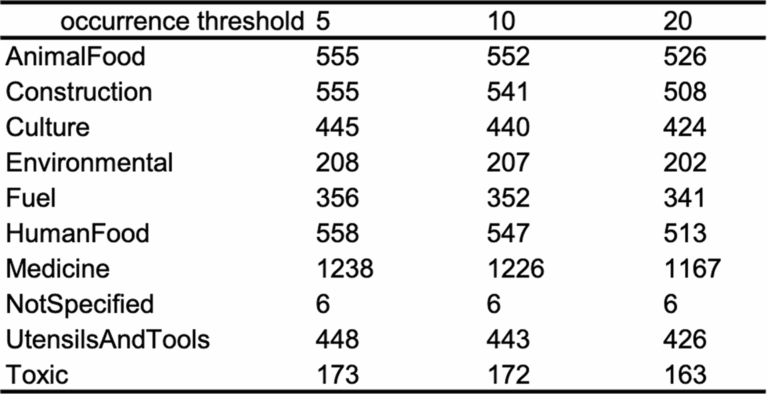
Sample sizes for projected climate-driven range changes across plant use categories

Sample sizes after filtering for Continuous Boyce index (CBI) ≥ 0.7 and different occurrence thresholds (≥ 5, ≥ 10, and ≥ 20 occurrence records). Values are shown for each plant use category. See Extended Data Fig. 8.

## Data Availability

Ethnobotanical data are available (http://www.the-forest-of-knowledge.com and on Zenodo^105^ (10.5281/zenodo.19202485)). SDMs are available on Zenodo^105^ (10.5281/zenodo.19202485). Language data are available from the Ethnologue^22^.

## Code availability

Code is available on Zenodo^105^ (10.5281/zenodo.19202485).
