# Supplementary material for: The forest of knowledge under global change
Source: Nature. 2026 Jul 8;655(8125):1212–6. doi: 10.1038/s41586-026-10741-y (PMC13421348; doi:10.1038/s41586-026-10741-y)
Supplement: Supplementary file 1 — Supplementary Note 1 containing the ODMAP description of Species Distribution Modelling workflow. [file 41586_2026_10741_MOESM1_ESM.pdf]

nature portfolio

https://doi.org/10.1038/s41586-026-10741-y

Supplementary information

The forest of knowledge under global change

In the format provided by the authors and unedited

Supplementary Note 1 | ODMAP description of Species Distribution Modelling workflow.

1. Overview

Species distribution models (SDMs) were developed to estimate present-day habitat suitability and project potential future suitability under climate change scenarios for a set of focal species – derived from vascular plant species with >20% of modelled range occurring within the Amazon Basin as in Feng et al. (*1*). Models were implemented in R using the Wallace modelling framework combined with the Maxent algorithm via the maxnet implementation. Occurrence records were obtained from the Botanical Information and Ecology Network (BIEN) database and processed to remove spatial duplicates and reduce sampling bias. Environmental predictors included climate and soil variables describing temperature, precipitation, and edaphic conditions. Models were calibrated using spatial cross-validation and evaluated using the continuous Boyce index (CBI). Final models were used to generate continuous suitability predictions and binary habitat maps under both current and projected future climate conditions.

2. Data

2.1 Response Variable (Occurrence Data)
Species occurrence records were obtained from the BIEN database using the Wallace function occs_queryDb. Occurrence datasets consisted of georeferenced presence-only observations including longitude and latitude coordinates.

Occurrence records were filtered through several steps prior to model fitting:
• Removal of duplicate raster cells so that no two records occur within the same environmental grid cell.
• Removal of records with missing environmental predictor values.
• Spatial thinning using a minimum nearest-neighbor distance of 10 km to reduce spatial sampling bias.

2.2 Environmental Predictors
Environmental predictors included climate and soil variables expected to influence species distributions.

Climate predictors:
• Annual mean temperature (bio1)
• Mean diurnal temperature range (bio2)
• Maximum temperature of warmest month (bio5)
• Minimum temperature of coldest month (bio6)
• Annual precipitation (bio12)
• Precipitation seasonality (bio15)

Soil predictors:
• Bedrock depth
• Soil pH
• Silt fraction
• Clay fraction
• Soil bulk density

All environmental predictors are 5 arc minute resolution with a WGS84 (EPSG:4326) CRS and were cropped from global extent to a bounding box that encompasses the Netotropical biogeographic realm (bounding box of Xmin = -110, Xmax = -34, Ymin = -60, Ymax = 29). Environmental predictors were stored as GeoTIFF rasters and loaded as SpatRaster objects using the R package terra.

3. Accessible Area and Background Data

3.1 Accessible Area Definition (M)
Background sampling was restricted to a species-specific accessible area representing the region hypothesized to have been accessible to the species through dispersal processes.

The accessible area was defined as 10 km buffers surrounding occurrence records. This buffered region was converted into a raster mask used to constrain background sampling.

3.2 Background Sampling
Within the accessible area mask, 10,000 background points were randomly sampled for each species. Environmental predictor values were extracted for these points and used during model calibration.

4. Model

4.1 Modelling Algorithm
Species distribution models were fitted using the Maxent algorithm implemented via the R package maxnet within the Wallace framework.

4.2 Feature Classes
Model features were restricted to Linear (L) and Quadratic (Q) feature classes.

4.3 Regularization
Regularization multipliers were tuned across values from 1 to 3 with a step size of 1 to control model complexity.

5. Partitioning and Model Evaluation

Model calibration and evaluation were performed using spatial cross-validation. Occurrence and background data were partitioned using the block method, which divides the study area into spatial quadrants to reduce spatial autocorrelation between training and testing datasets.

Model performance was evaluated using the continuous Boyce index (CBI). The optimal model configuration was selected based on the highest test CBI value.

6. Prediction

6.1 Current Climate Predictions
The selected model was used to generate spatial predictions of habitat suitability across the environmental rasters representing present-day conditions using the cloglog output transformation.

6.2 Thresholding and Binary Maps
Binary habitat suitability maps were generated using the 10th percentile training presence threshold. Additional thresholds at the 20th and 30th percentiles were also calculated for comparison.

7. Projection to Future Climate Scenarios

Models were projected to future climate conditions using environmental predictors representing multiple climate models and emissions scenarios.

General circulation models included:
- GFDL
- UKESM
- MRI
- IPSL
- MPI

Climate scenarios included:
• SSP1-2.6
• SSP3-7.0
• SSP5-8.5

8. Extrapolation and Novel Climate Conditions

Model transfer to future climates may encounter environmental conditions outside the range observed during model calibration. Clamping was disabled during prediction and projections into novel environmental conditions should therefore be interpreted cautiously. Additional analyses such as MESS mapping could be used to identify areas of extrapolation.

9. Output Products

For each species the workflow produced:
• Cleaned occurrence records
• Spatially thinned occurrence dataset
• Background sampling extent
• Fitted SDM model object
• Continuous current suitability raster
• Binary current suitability raster
• Continuous suitability projections for each future scenario
• Binary projections for each future scenario
• Threshold values used for binary classification

10. Software and Reproducibility

All analyses were conducted in R using the packages wallace, maxnet, ENMeval, terra, spocc, spThin, sf, and dismo. Raster processing and environmental data extraction were conducted using the R package terra. The workflow was implemented as a scripted pipeline enabling reproducible model generation across multiple species and climate scenarios.

References

1. Feng, X. et al. How deregulation, drought and increasing fire impact Amazonian biodiversity. *Nature* **597**, 516–521 (2021).
