# Supplementary material for: The forest of knowledge under global change
Source: Nature. 2026 Jul 8;655(8125):1212–6. doi: 10.1038/s41586-026-10741-y (PMC13421348; doi:10.1038/s41586-026-10741-y)
Supplement: Supplementary file 2 — Reporting Summary [file 41586_2026_10741_MOESM2_ESM.pdf]

Corresponding author(s): Rodrigo Cámara-Leret

Last updated by author(s): May 14, 2026

Reporting Summary

Nature Portfolio wishes to improve the reproducibility of the work that we publish. This form provides structure for consistency and transparency in reporting. For further information on Nature Portfolio policies, see our Editorial Policies and the Editorial Policy Checklist.

Statistics

For all statistical analyses, confirm that the following items are present in the figure legend, table legend, main text, or Methods section.

n/a	Confirmed	
☐	☒	The exact sample size (*n*) for each experimental group/condition, given as a discrete number and unit of measurement
☒	☐	A statement on whether measurements were taken from distinct samples or whether the same sample was measured repeatedly
☐	☒	The statistical test(s) used AND whether they are one- or two-sided<br>*Only common tests should be described solely by name; describe more complex techniques in the Methods section.*
☒	☐	A description of all covariates tested
☒	☐	A description of any assumptions or corrections, such as tests of normality and adjustment for multiple comparisons
☐	☒	A full description of the statistical parameters including central tendency (e.g. means) or other basic estimates (e.g. regression coefficient) AND variation (e.g. standard deviation) or associated estimates of uncertainty (e.g. confidence intervals)
☐	☒	For null hypothesis testing, the test statistic (e.g. F, t, r) with confidence intervals, effect sizes, degrees of freedom and P value noted<br>*Give P values as exact values whenever suitable.*
☒	☐	For Bayesian analysis, information on the choice of priors and Markov chain Monte Carlo settings
☒	☐	For hierarchical and complex designs, identification of the appropriate level for tests and full reporting of outcomes
☒	☐	Estimates of effect sizes (e.g. Cohen's *d*, Pearson's *r*), indicating how they were calculated

Our web collection on statistics for biologists contains articles on many of the points above.

Software and code

Policy information about availability of computer code

Data collection	n/a
Data analysis	n/a

For manuscripts utilizing custom algorithms or software that are central to the research but not yet described in published literature, software must be made available to editors and reviewers. We strongly encourage code deposition in a community repository (e.g. GitHub). See the Nature Portfolio guidelines for submitting code & software for further information.

Data

Policy information about availability of data

All manuscripts must include a data availability statement. This statement should provide the following information, where applicable:

- Accession codes, unique identifiers, or web links for publicly available datasets
- A description of any restrictions on data availability
- For clinical datasets or third party data, please ensure that the statement adheres to our policy

Ethnobotanical data is available in the website http://www.the-forest-of-knowledge.com and Zenodo: https://doi.org/10.5281/zenodo.19202485. SDMs are available in Zenodo: https://doi.org/10.5281/zenodo.19202485. Language data are available from the Ethnologue. Code is available in Zenodo: https://doi.org/10.5281/zenodo.19202485.

Research involving human participants, their data, or biological material

Policy information about studies with human participants or human data. See also policy information about sex, gender (identity/presentation), and sexual orientation and race, ethnicity and racism.

Reporting on sex and gender	n/a
Reporting on race, ethnicity, or other socially relevant groupings	n/a
Population characteristics	n/a
Recruitment	n/a
Ethics oversight	n/a

Note that full information on the approval of the study protocol must also be provided in the manuscript.

Field-specific reporting

Please select the one below that is the best fit for your research. If you are not sure, read the appropriate sections before making your selection.

☐ Life sciences ☐ Behavioural & social sciences ☒ Ecological, evolutionary & environmental sciences

For a reference copy of the document with all sections, see nature.com/documents/nr-reporting-summary-flat.pdf

Ecological, evolutionary & environmental sciences study design

All studies must disclose on these points even when the disclosure is negative.

Study description

This study consists of four parts.

1) Amazonian plants utilized by people. A bibliographic review (1504–2023) of 90,536 plant use reports from 700 references was made to understand the societal benefits that native vascular plants provide across all countries of the Amazon basin.
We then calculated the proportion of the Amazon flora that is known to Indigenous and local people by building a checklist to the Amazon flora—integrating two published checklists of tree species and non-tree species, harmonizing their scientific names (using the R package rWCVP ver. 1.2.4). We further added 407 native species missed by the previous Amazon checklists, but which were reported by ethnobotanical studies; we confirmed their presence after double-checking type specimens at JSTOR Global Plants (https://plants.jstor.org), specimen occurrences at GBIF, and synonymy in the Vascular Plants of the Americas and the Plants of the World Online online portals).

2) Climate change impacts on plant species. We assessed to what degree climate change may impact the geographic range of plant species that matter to people vs. species that are not utilized. We built species distribution models (4509 utilized species and 3920 non-utilized species), projected them to five general circulation models (GCMs: gfdl, ukesm, mpi, ipsl, mri) and three Shared Socioeconomic Pathways (SSPs 1-2.6, 3-7.0, 5-8.5), and compared the mean change in the geographic range of utilized vs. non-utilized species by 2060–2080. We generated SDMs via the ENMeval v2.0 framework implemented in the R package 'wallace 2'.

3) Local climate change impacts Indigenous knowledge networks. For each of the 82 Indigenous languages with at least 10 utilized species in the literature, we built an Indigenous knowledge network that relates individual plant species (nodes in one set) to particular services (nodes on the other set) based on the knowledge (links) held by speakers of that language. We then quantified the climate change exposure of each Indigenous knowledge network by calculating the proportion of utilized species and services that may be driven locally extinct by 2060–2080 with the R package igraph ver. 2.0.3.

4) Language change impacts Indigenous knowledge. We first built an Indigenous knowledge metaweb (i.e., the aggregated network resulting from adding all Indigenous and non-indigenous knowledge networks, which represents all total knowledge about the services provided by all plant species in the study area). Next, we obtained linguistic threat information for all the languages in our sample (from Ethnologue) and estimated the impact of language loss on the erosion of the Indigenous knowledge metaweb.

Research sample

We focused our analysis on all the native vascular plant species and the Indigenous cultures occurring in the Amazon basin. We delimit the Amazon basin following the biogeographic limits proposed by the Amazon Network of Georeferenced Socio-Environmental Information (Rede Amazonica de Informacao Socioambiental Georreferenciada (2021), available at www.amazoniasocioambiental.org/en/maps/).

Sampling strategy

No statistical method was used for sample size calculation. The sample size in our case reflects the data that is available from public or institutional repositories for our study area.

Data collection

Plant occurrence records were gathered from the BIEN ver. 4.1 database using the R package bien ver. 1.2.8 and spocc ver. 1.2.4; Bioclimatic variables from CHELSA database at 5 arc-minute (~10 km) spatial resolution ; Soil-related variables from Soilgrids ver. 1.0. All R packages and their reference are cited in the Methods section, and the version numbers are cited in the text.

Ethnobotanical data on Amazonia's utilised plants was compiled from existing datasets, as follows:
1) Plant service records: RCL gathered five different types of information: i) regional compilations for the Amazon basin, northwest Amazonia, and the Guianas, ii) country-level compilations, iii) monographs on individual Indigenous groups, iv) specific plant services, e.g., Food, and v) early historical accounts, extending to the 16th century.
2) Plant occurrence records: PR obtained primary species occurrence records through automated programmatic queries to the Botanical Information and Ecology Network (BIEN) using the spocc R package.
3) Environmental variables: PR downloaded bioclimatic variables from CHELSA and soil variables from Soilgrids ver. 1.0.
4) Indigenous languages. RCL verified Indigenous group names and their spoken language and geographic coordinates were recorded using Ethnologue and Glottolog. RCL compiled language threat from the Ethnologue and from Glottolog.
5) Checklist of Amazonian vascular plants and harmonization of scientific names. RCL reviewed the scientific names of utilized species and confirmed their native status in Amazonia in three steps: First, a checklist of Amazonian vascular seed plants was built ("Amazon checklist", hereafter) by combining the tree species of ter Steege et al. (Ref. 15) and the non-tree species of Cardoso et al. (Ref. 14). After removing duplicates, vascular seed plant species names were reviewed using the R package rWCVP. All non-accepted names from the rWCVP analysis (i.e., Illegitimate, Invalid, Misapplied, Orthographic, Synonym, Unplaced, NA) were checked manually against Plants of the World Online (POWO) (http: //www.plantsoftheworldonline.org) and the online Catalogue of Vascular Plants of the Americas(VPA). This verification resulted in two lists, depending on the online synonymy portal that was consulted: POWO or VPA. Third, since the "Amazon checklist" only includes seed plants, the utilized species list was filtered to seed plants. This filtered list was then compared to the POWO list and VPA list. All non-matching names were double-checked by consulting online type specimens at JSTOR Global Plants (https://plants.jstor.org), specimen occurrences at GBIF (https://www.gbif.org), and synonymy in the VPA and POWO online portals.

Timing and spatial scale	Plant service records were compiled from 700 bibliographic references published between the years 1504 to 2023. For plant occurrence records, we used all collections available through time for the study area.
Data exclusions	All reports of plant species and Indigenous cultures that are not-native to the Amazon basin were excluded. We applied different quality filters to clean scientific names: we excluded fungi, lichens, algae, bryophytes, and marine species (e.g., sea grasses) Doubtful species identifications (e.g., 'cf.', 'sp. nov.', 'aff.', 'sp.') were classified to generic level. Misspelled genera were manually corrected and doubtful cases excluded. We removed all known hybrids from the analyses.
Reproducibility	As this is not an experimental study, replication was not conducted.
Randomization	Samples randomization does not apply within the context of this experimental design.
Blinding	Investigators were not blinded during data acquisition and analysis: this is not feasible within this experimental design.

Did the study involve field work? ☐ Yes ☒ No

Reporting for specific materials, systems and methods

We require information from authors about some types of materials, experimental systems and methods used in many studies. Here, indicate whether each material, system or method listed is relevant to your study. If you are not sure if a list item applies to your research, read the appropriate section before selecting a response.

Materials & experimental systems

n/a	Involved in the study
☒	☐ Antibodies
☒	☐ Eukaryotic cell lines
☒	☐ Palaeontology and archaeology
☒	☐ Animals and other organisms
☒	☐ Clinical data
☒	☐ Dual use research of concern
☒	☐ Plants

Methods

n/a	Involved in the study
☒	☐ ChIP-seq
☒	☐ Flow cytometry
☒	☐ MRI-based neuroimaging

Plants

Seed stocks	n/a
Novel plant genotypes	n/a
Authentication	n/a
