# Supplementary material for: The forest of knowledge under global change
Source: Nature. 2026 Jul 8;655(8125):1212–6. doi: 10.1038/s41586-026-10741-y (PMC13421348; doi:10.1038/s41586-026-10741-y)
Supplement: Supplementary file 3 — Genus-level patterns in projected range change for Amazonian utilised plant species. Mean relative change in Amazonian range area (future/baseline) for utilised plant species, aggregated to the genus level and shown by family. Points show genus means under three future climate scenarios (SSP1–2.6, SSP3–7.0 and SSP5–8.5), with error bars indicating ±1 s.d. across general circulation models. The dashed black line shows the family-specific mean of species’ present-climate Amazonian range (averaged across general circulation models), used as the reference for calculating relative range change. Families are displayed across multiple pages, with axes scaled independently to highlight within-family variation. Sample sizes (n = number of species) per genus are shown in parentheses next to genus names. [file 41586_2026_10741_MOESM3_ESM.pdf]

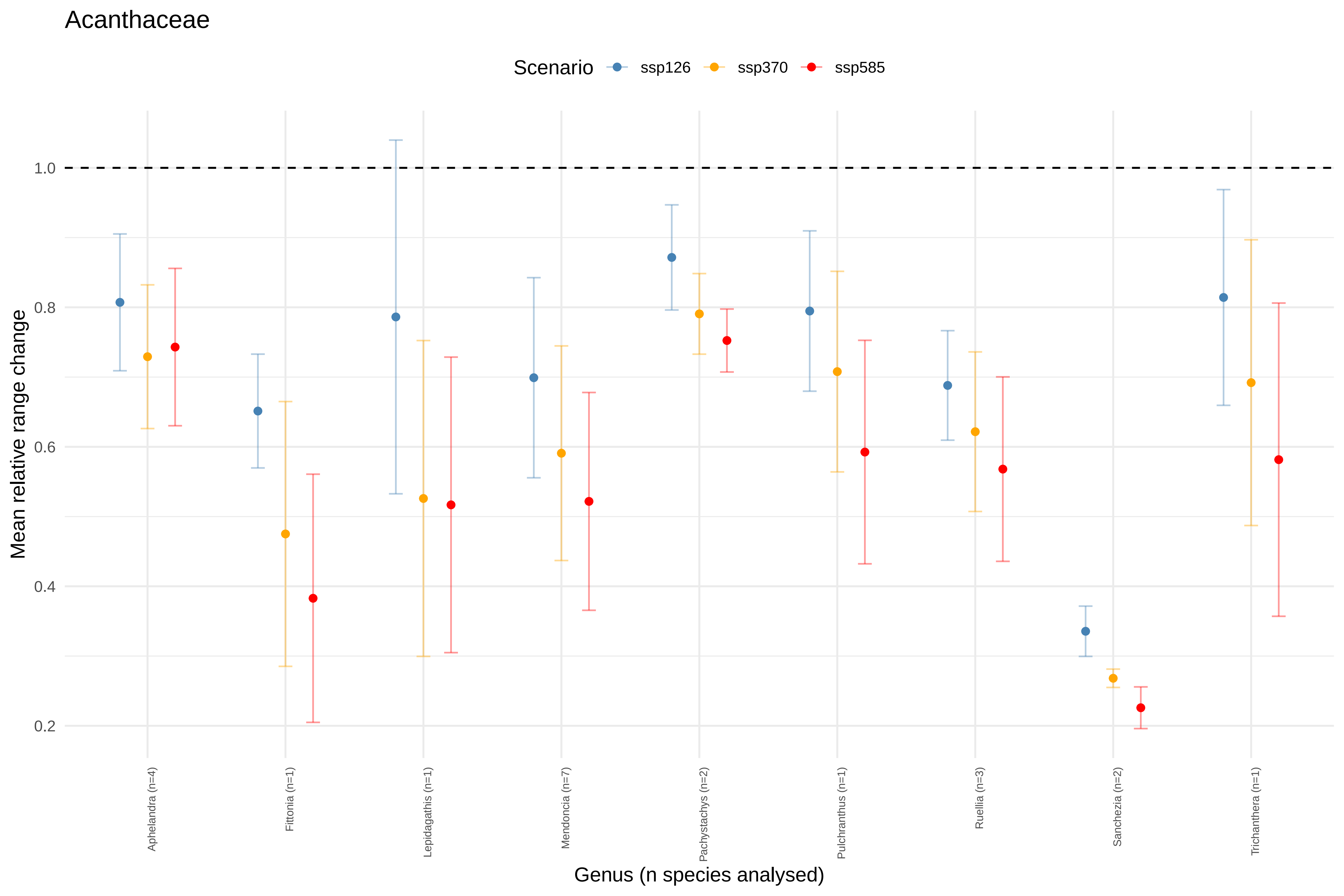
Acanthaceae
Scenario
ssp126
ssp370
ssp585
Mean relative range change
1.0
0.8
0.6
0.4
0.2
Aphelandra (n=4)
Fittonia (n=1)
Lepidagathis (n=1)
Mendoncia (n=7)
Pachystachys (n=2)
Pulchranthus (n=1)
Ruellia (n=3)
Sanchezia (n=2)
Trichanthera (n=1)
Genus (n species analysed)

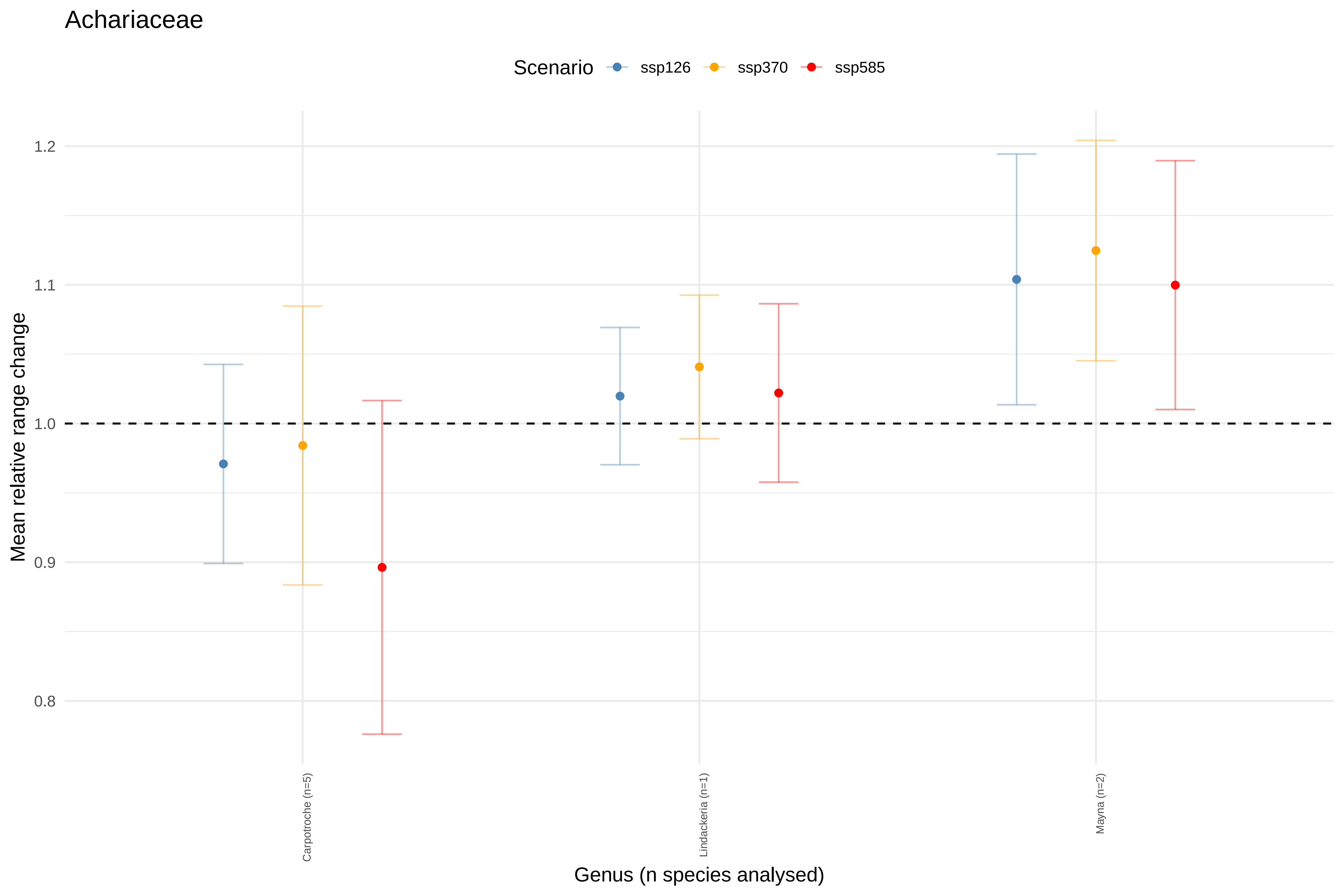

Achariaceae
Scenario
ssp126
ssp370
ssp585
Mean relative range change
1.2
1.1
1.0
0.9
0.8
Carpotroche (n=5)
Lindackeria (n=1)
Mayna (n=2)
Genus (n species analysed)

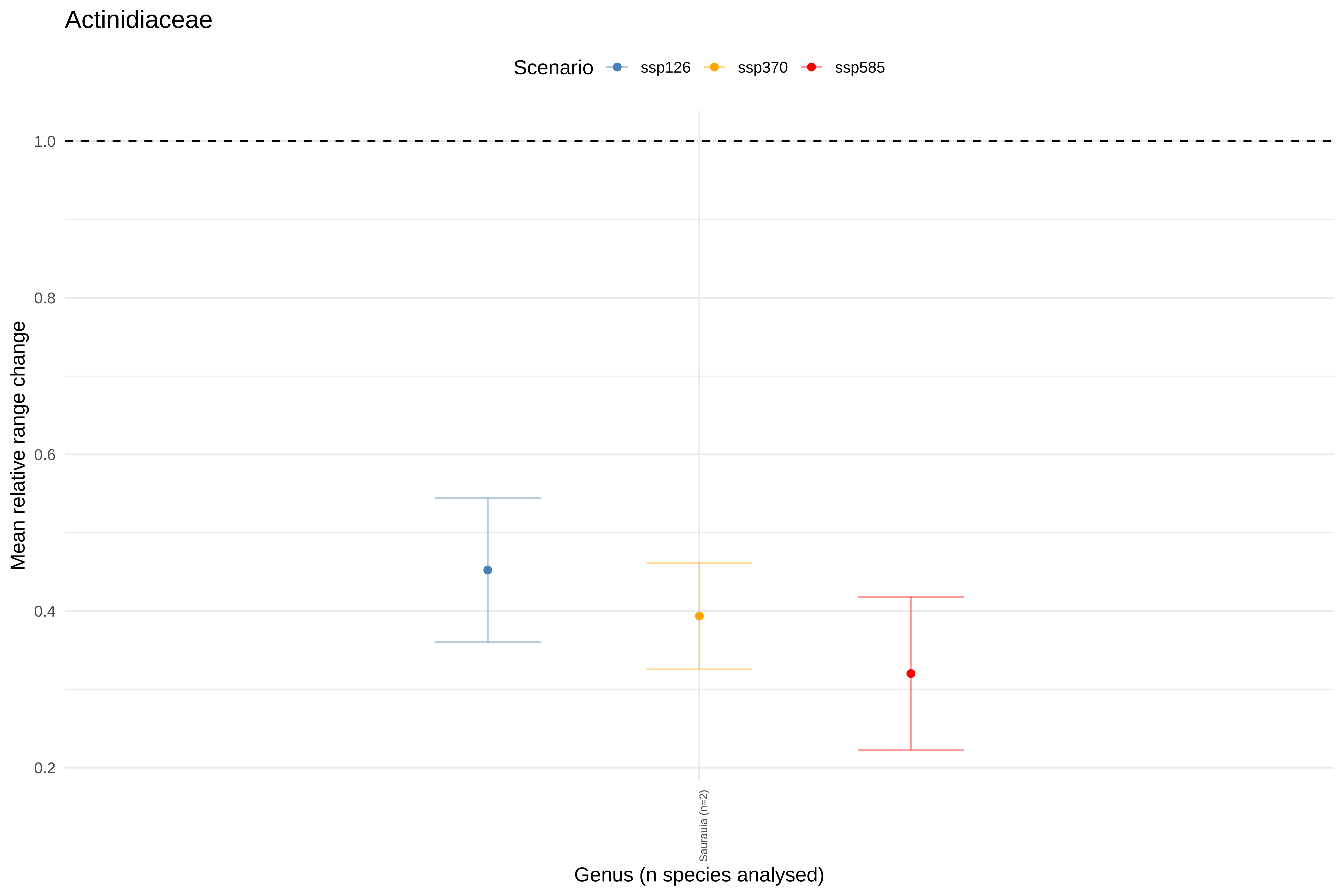

Actinidiaceae
Scenario
ssp126
ssp370
ssp585
1.0
0.8
0.6
0.4
0.2
Mean relative range change
Saurauia (n=2)
Genus (n species analysed)

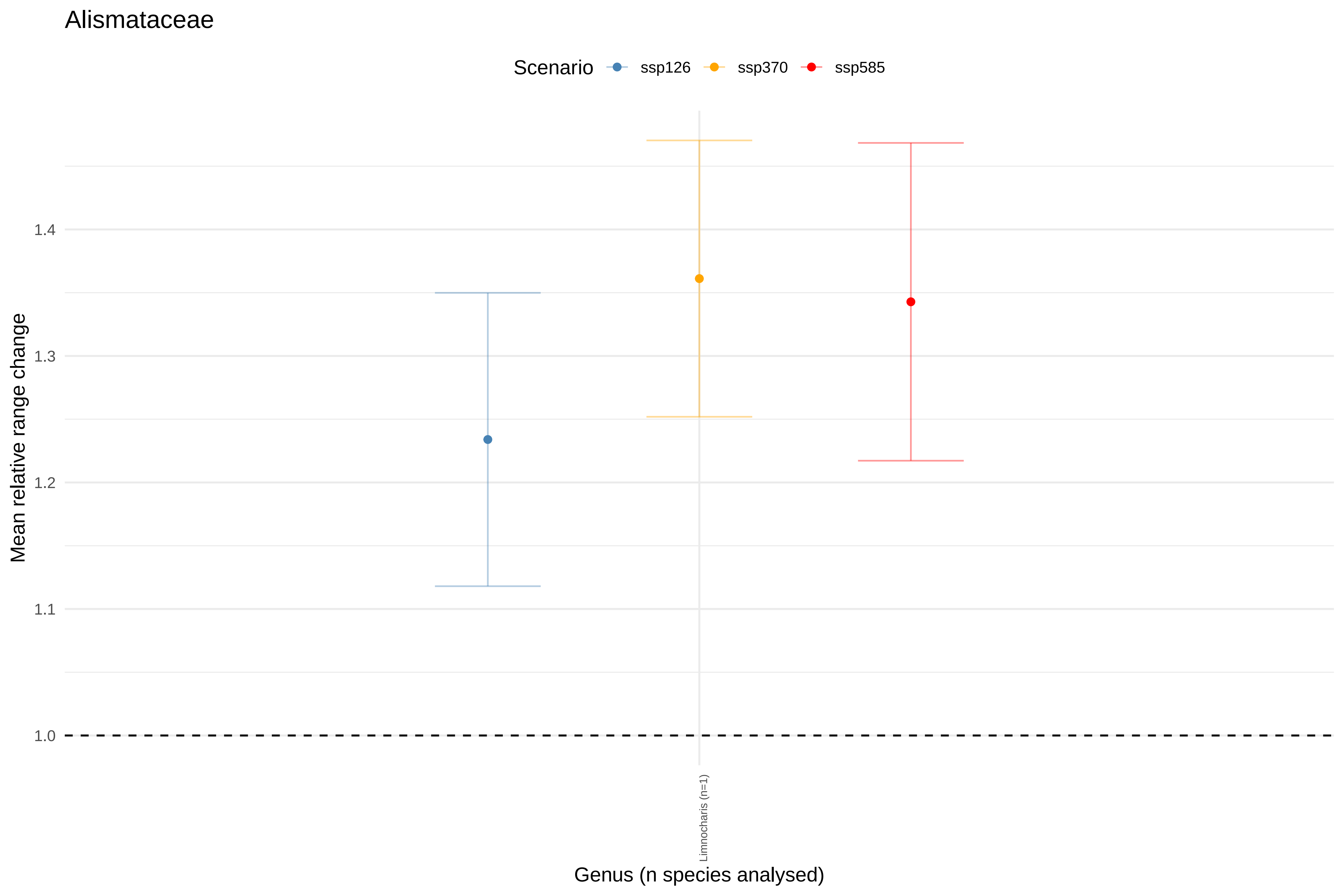

Alismataceae
Scenario
ssp126
ssp370
ssp585
Mean relative range change
1.4
1.3
1.2
1.1
1.0
Limnocharis (n=1)
Genus (n species analysed)

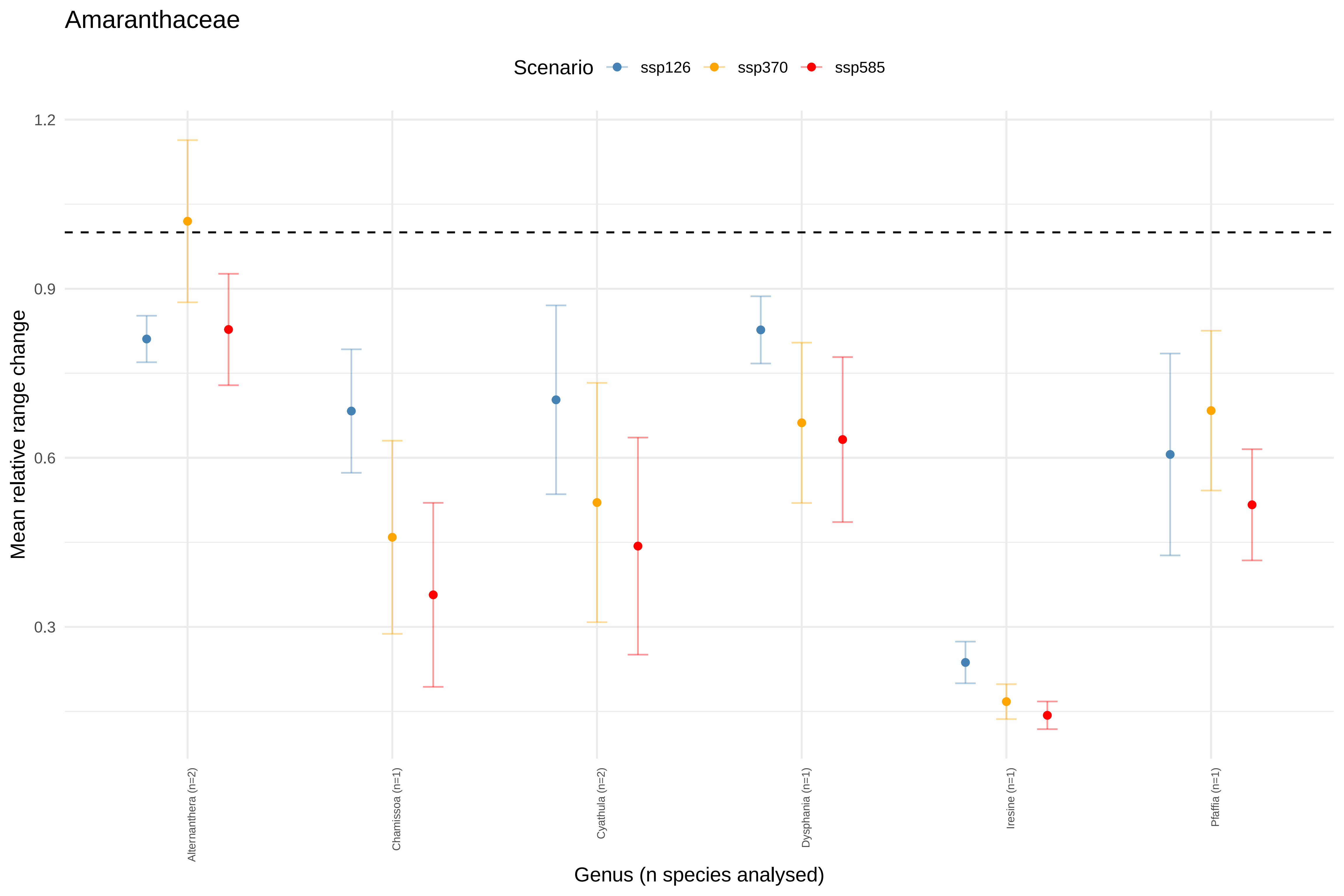

Amaranthaceae
Scenario
ssp126
ssp370
ssp585
1.2
0.9
0.6
0.3
Mean relative range change
Alternanthera (n=2)
Chamissoa (n=1)
Cyathula (n=2)
Dysphania (n=1)
Iresine (n=1)
Pfaffia (n=1)
Genus (n species analysed)

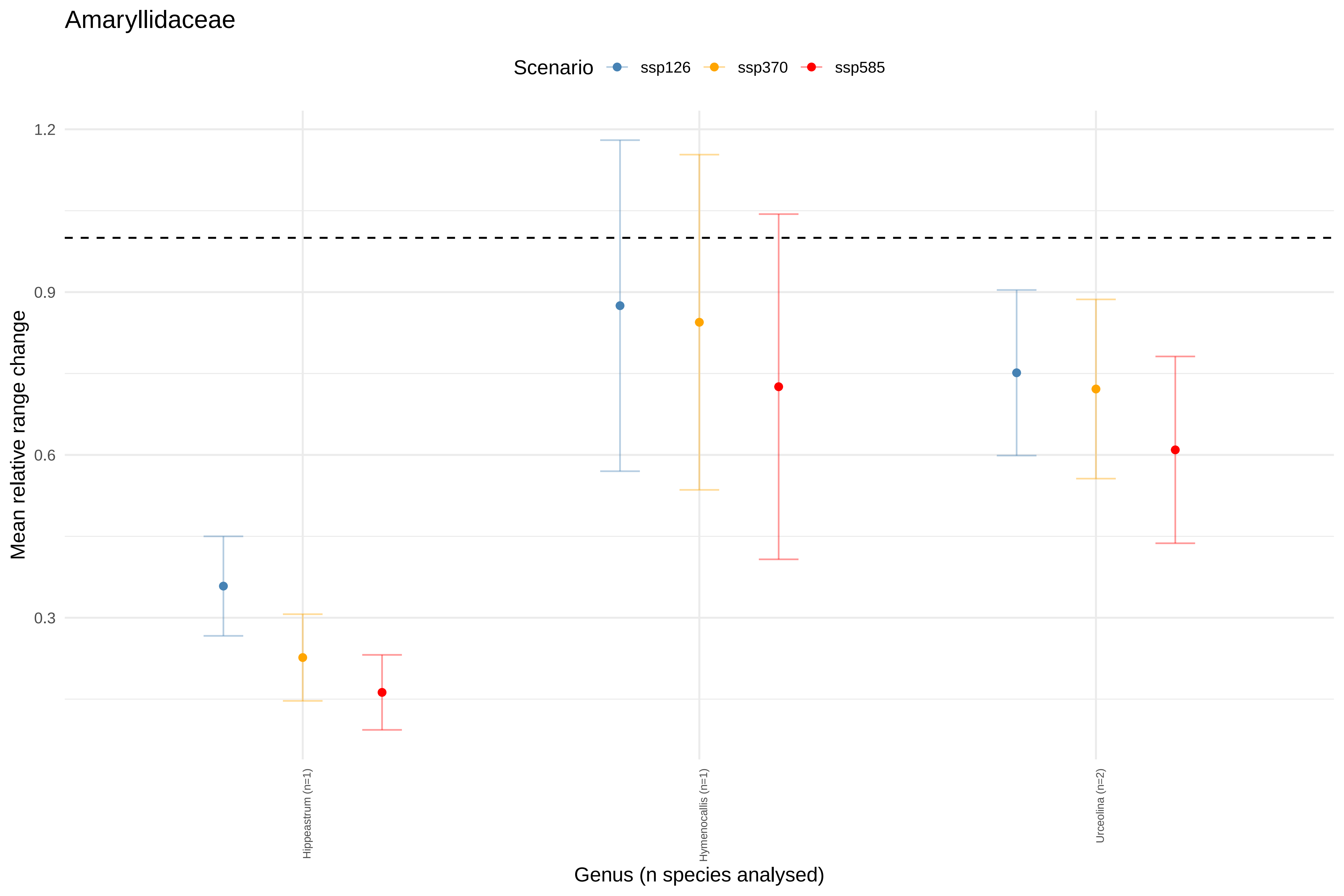
Amaryllidaceae
Scenario
ssp126
ssp370
ssp585
1.2
0.9
0.6
0.3
Mean relative range change
Hippeastrum (n=1)
Hymenocallis (n=1)
Urceolina (n=2)
Genus (n species analysed)

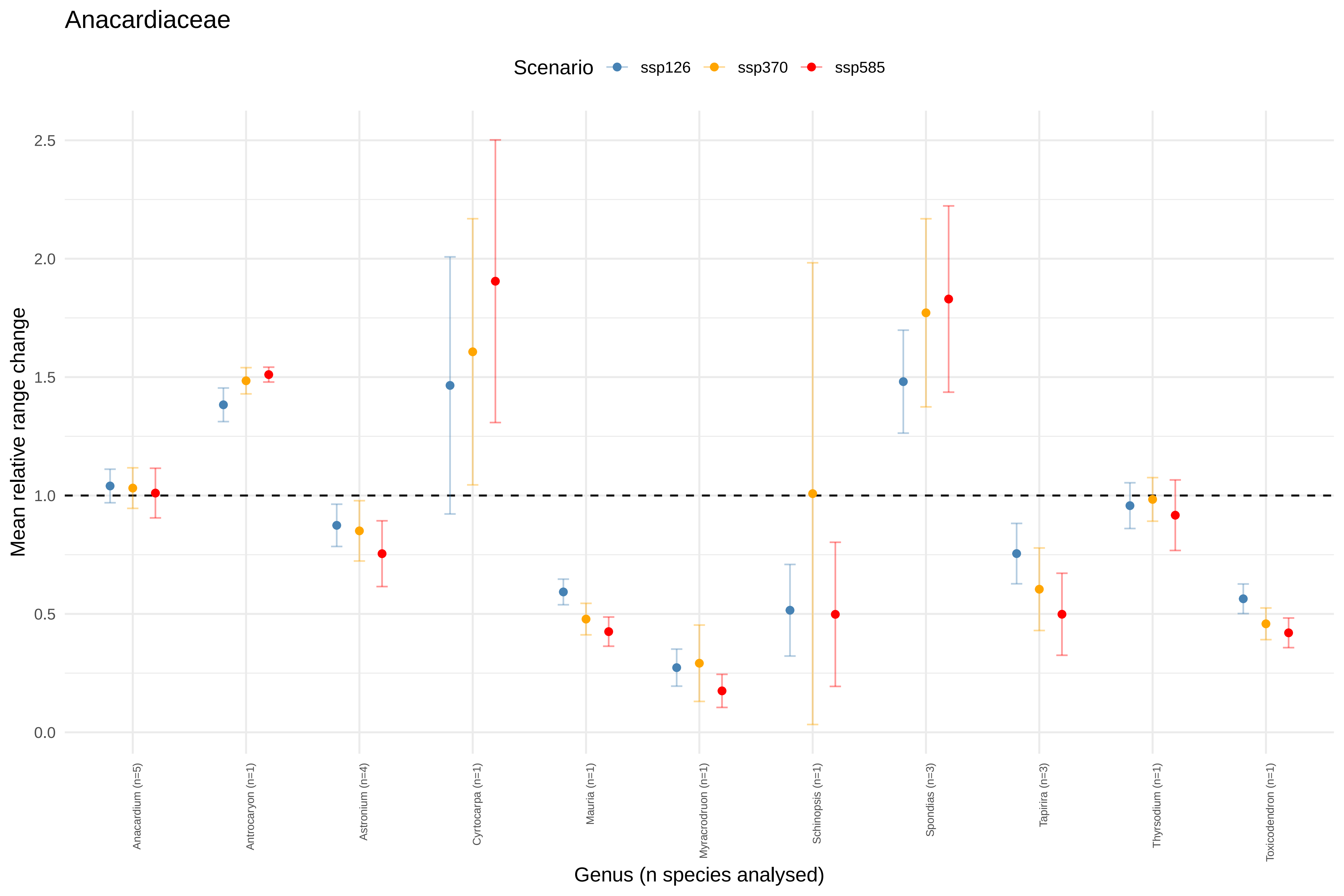
Anacardiaceae
Scenario
ssp126
ssp370
ssp585
Mean relative range change
2.5
2.0
1.5
1.0
0.5
0.0
Anacardium (n=5)
Antrocaryon (n=1)
Astronium (n=4)
Cyrtocarpa (n=1)
Mauria (n=1)
Myracrodruon (n=1)
Schinopsis (n=1)
Spondias (n=3)
Tapirira (n=3)
Thyrsodium (n=1)
Toxicodendron (n=1)
Genus (n species analysed)

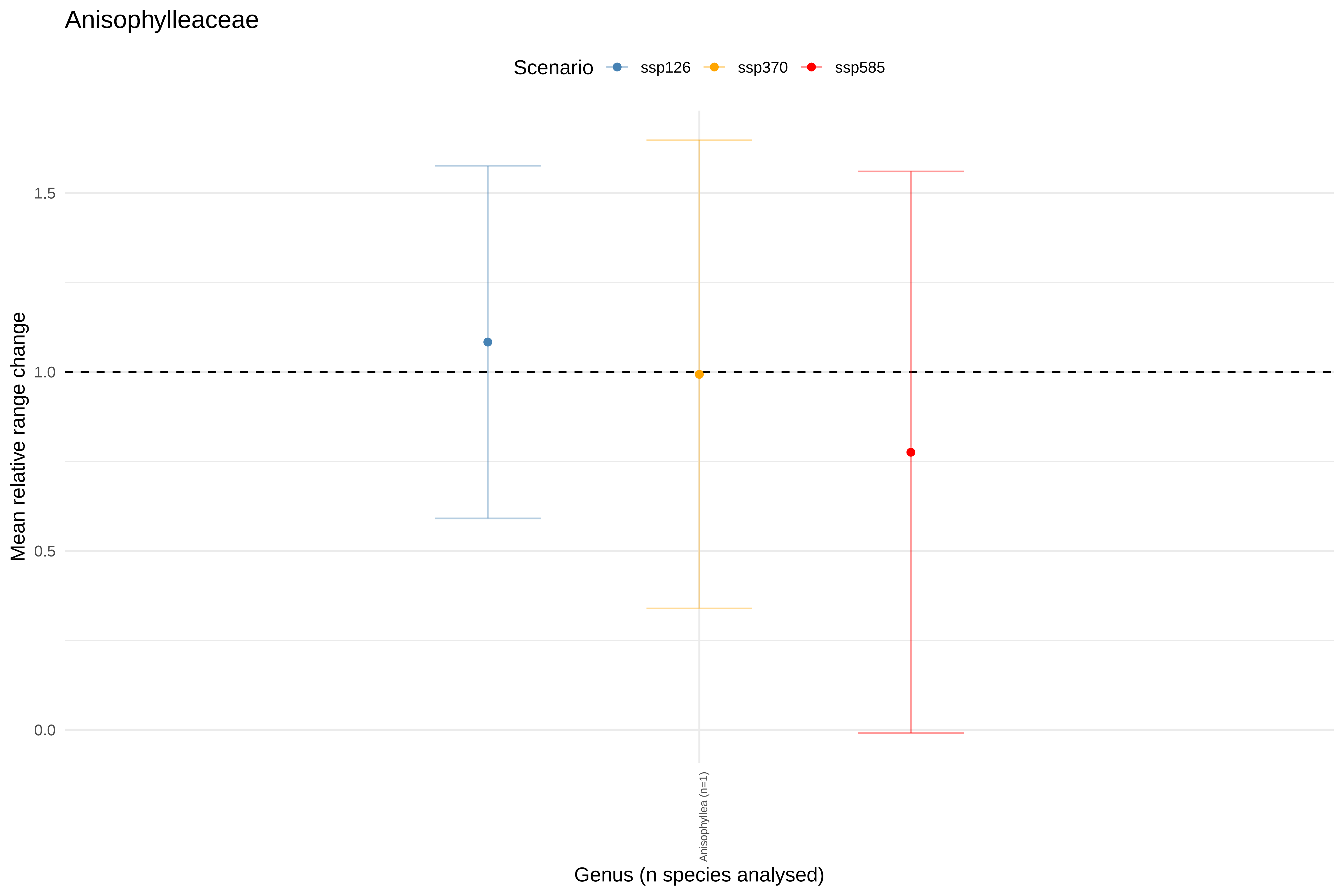
Anisophylleaceae
Scenario
ssp126
ssp370
ssp585
Mean relative range change
1.5
1.0
0.5
0.0
Anisophyllea (n=1)
Genus (n species analysed)

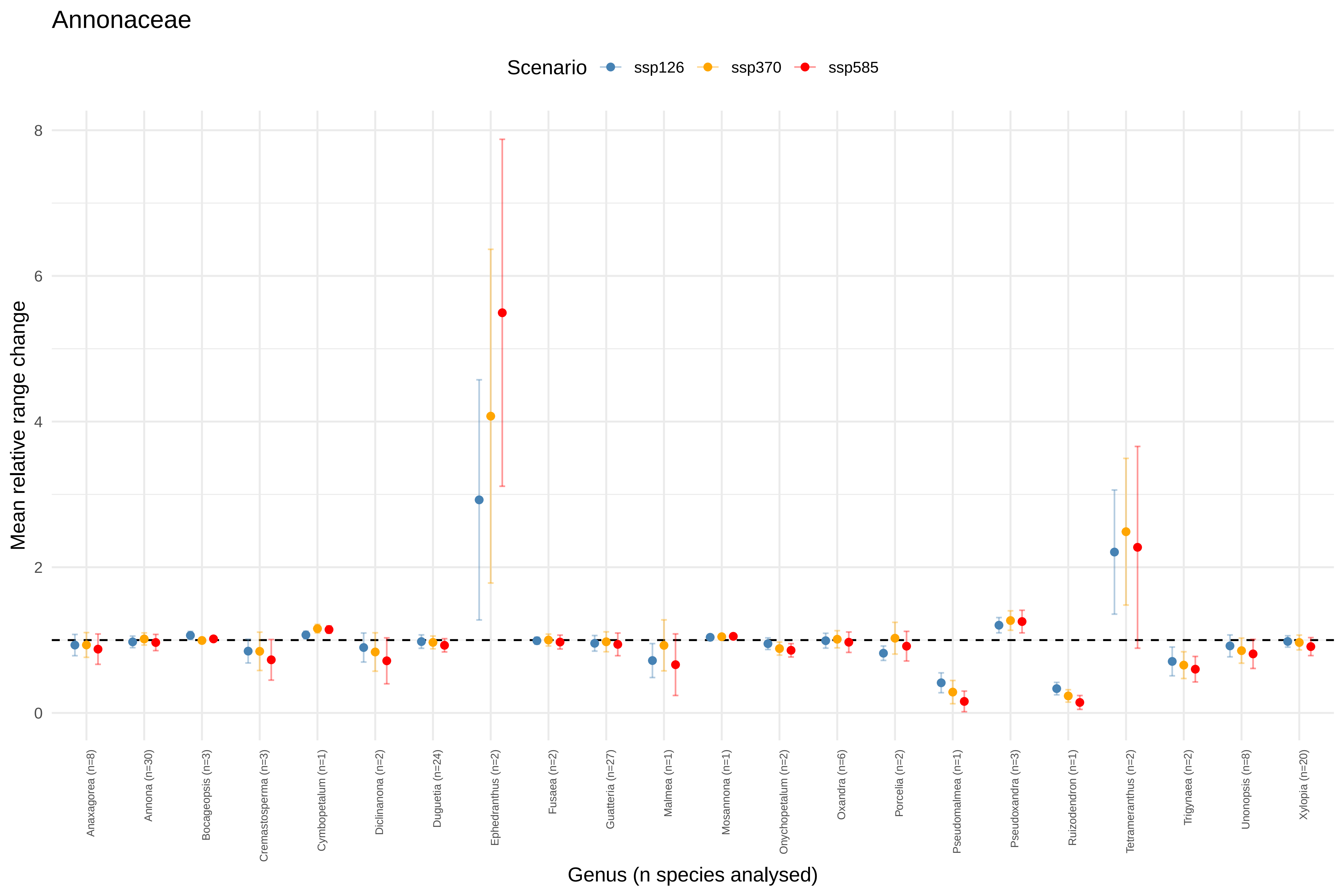

Annonaceae
Scenario
ssp126
ssp370
ssp585
Mean relative range change
0
2
4
6
8
Anaxagorea (n=8)
Annona (n=30)
Bocageopsis (n=3)
Cremastosperma (n=3)
Cymbopetalum (n=1)
Diclinanona (n=2)
Duguetia (n=24)
Ephedranthus (n=2)
Fusaea (n=2)
Guatteria (n=27)
Malmea (n=1)
Mosannona (n=1)
Onychopetalum (n=2)
Oxandra (n=6)
Porcelia (n=2)
Pseudomalmea (n=1)
Pseudoxandra (n=3)
Ruizodendron (n=1)
Tetrameranthus (n=2)
Trigynaea (n=2)
Unonopsis (n=8)
Xylopia (n=20)
Genus (n species analysed)

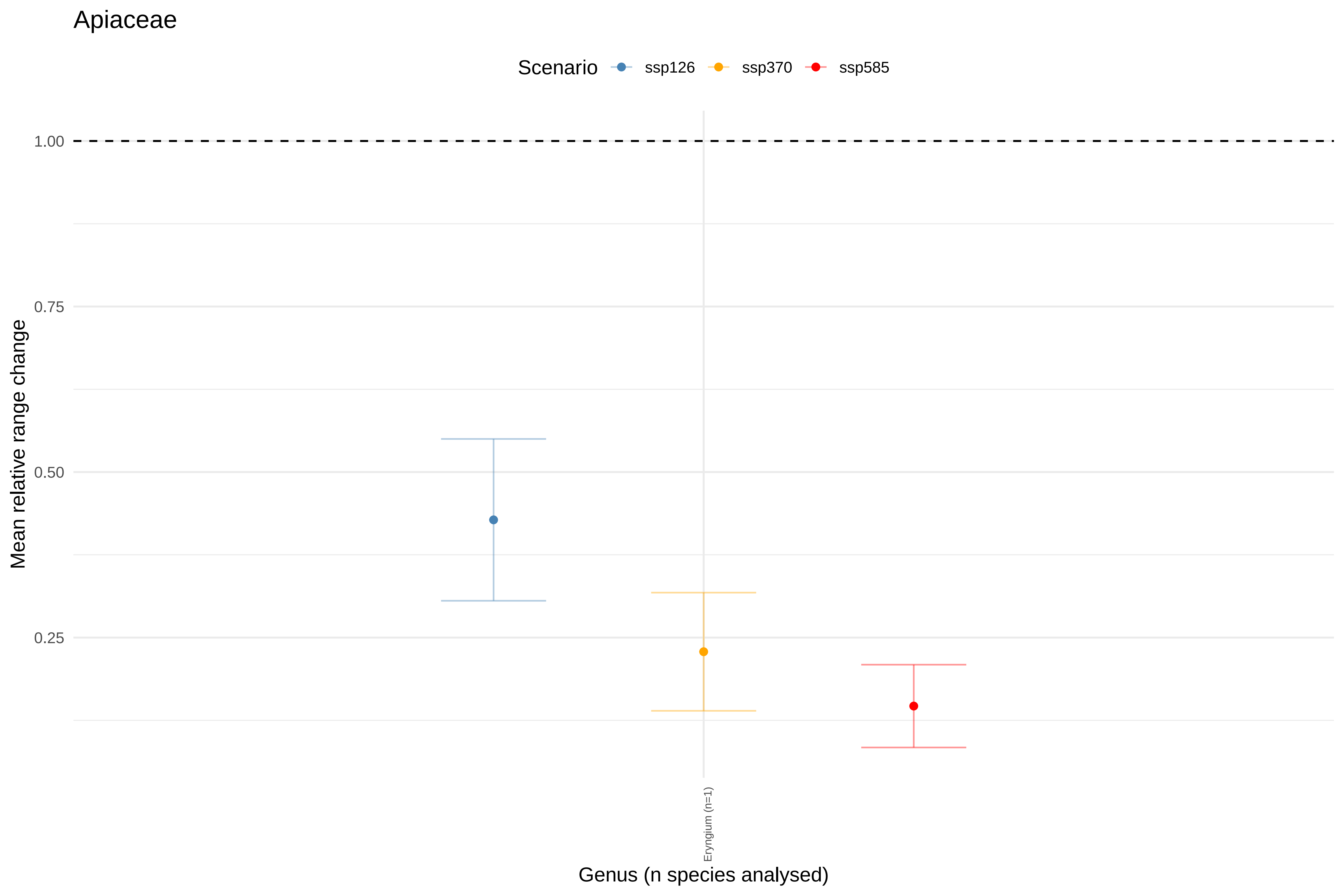

Apiaceae
Scenario
ssp126
ssp370
ssp585
1.00
0.75
0.50
0.25
Mean relative range change
Eryngium (n=1)
Genus (n species analysed)

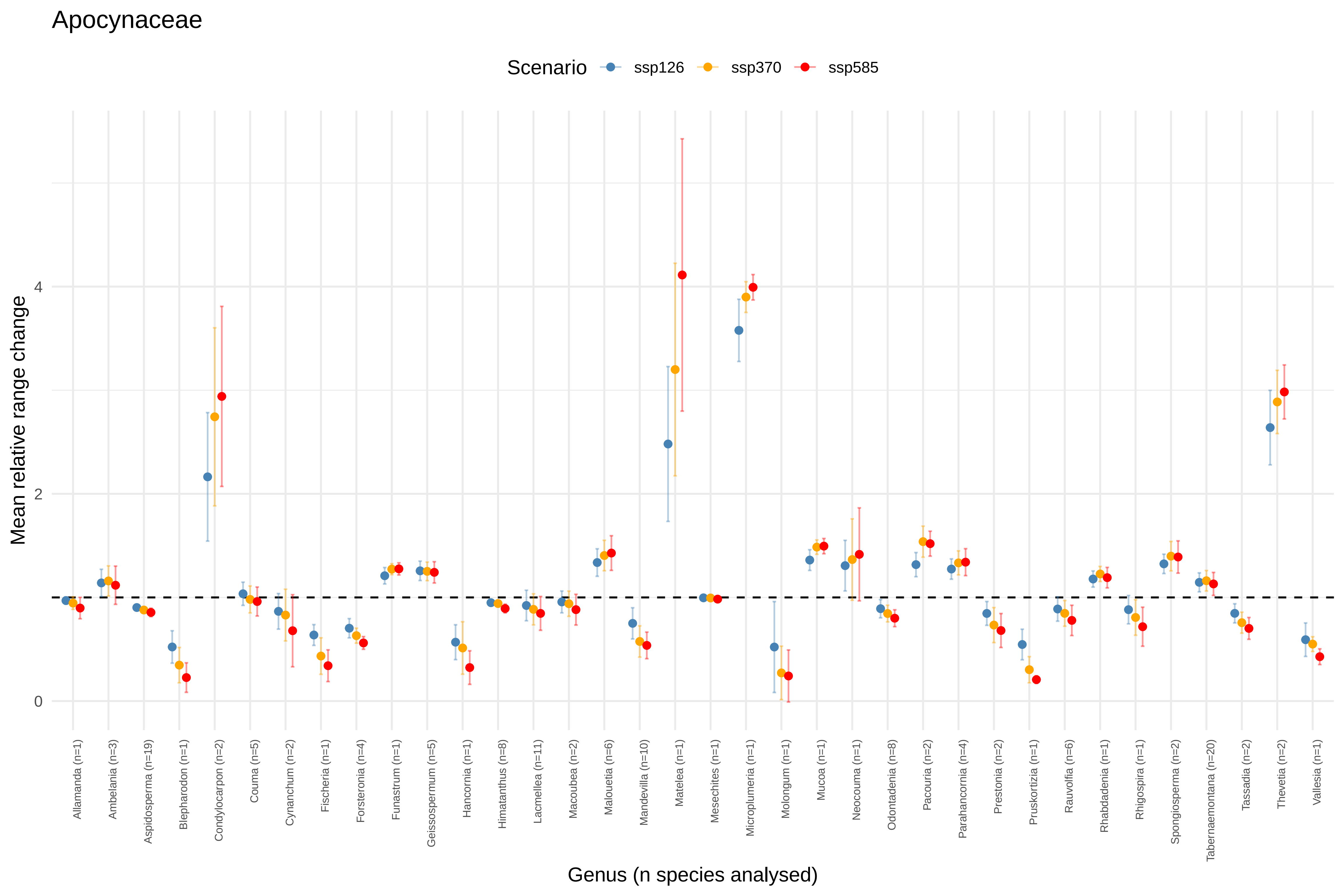

Apocynaceae
Scenario
ssp126
ssp370
ssp585
Mean relative range change
0
2
4
Genus (n species analysed)
Allamanda (n=1)
Ambelania (n=3)
Aspidosperma (n=19)
Blepharodon (n=1)
Condylocarpon (n=2)
Couma (n=5)
Cynanchum (n=2)
Fischeria (n=1)
Forsteronia (n=4)
Funastrum (n=1)
Geissospermum (n=5)
Hancornia (n=1)
Himatanthus (n=8)
Lacmellea (n=11)
Macoubea (n=2)
Malouetia (n=6)
Mandevilla (n=10)
Matelea (n=1)
Mesechites (n=1)
Microplumeria (n=1)
Molongum (n=1)
Mucoa (n=1)
Neocouma (n=1)
Odontadenia (n=8)
Pacouria (n=2)
Parahancornia (n=4)
Prestonia (n=2)
Pruskortizia (n=1)
Rauvolfia (n=6)
Rhabdadenia (n=1)
Rhigospira (n=1)
Spongiosperma (n=2)
Tabernaemontana (n=20)
Tassadia (n=2)
Thevetia (n=2)
Vallesia (n=1)

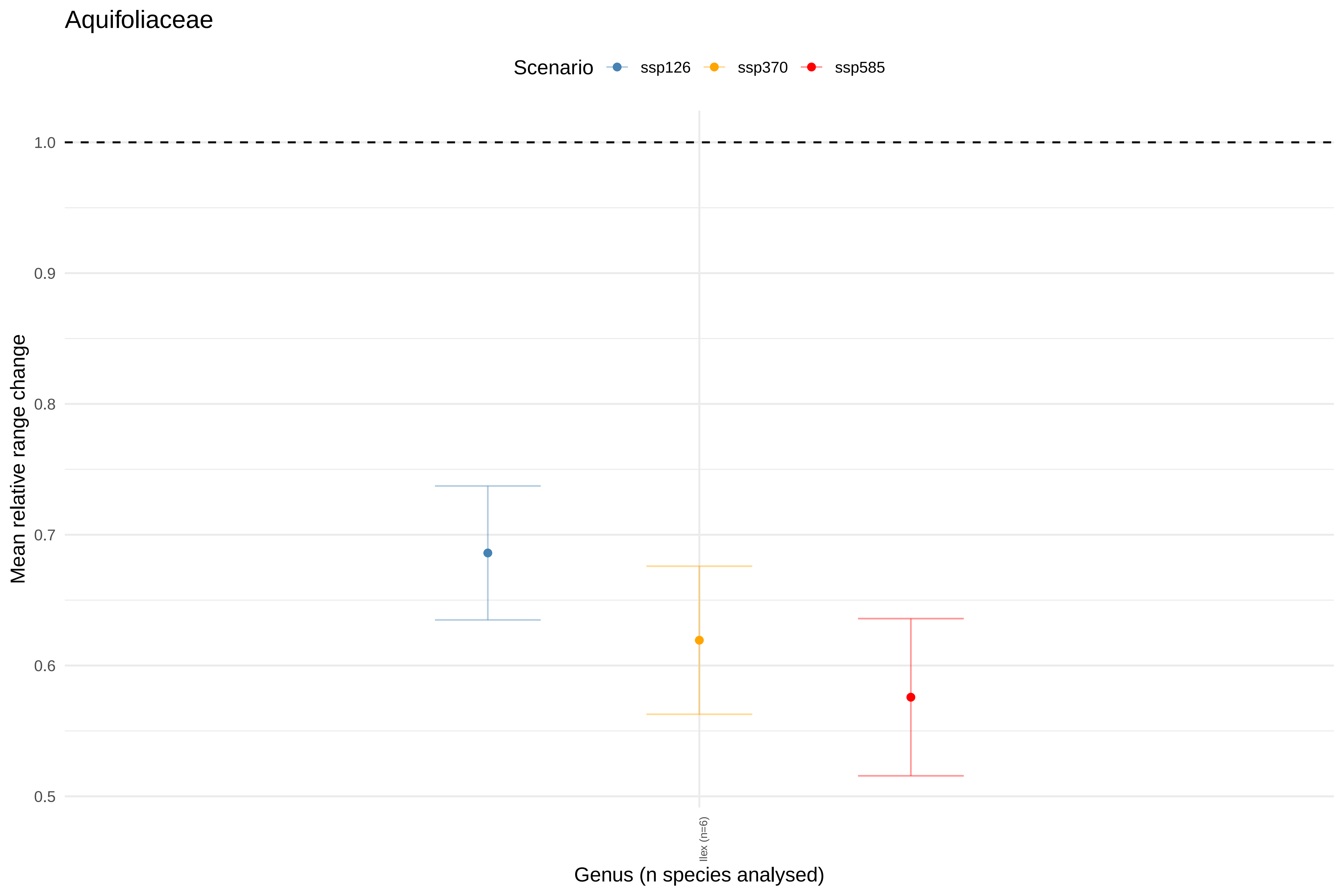

Aquifoliaceae
Scenario
ssp126
ssp370
ssp585
1.0
0.9
0.8
0.7
0.6
0.5
Mean relative range change
Ilex (n=6)
Genus (n species analysed)

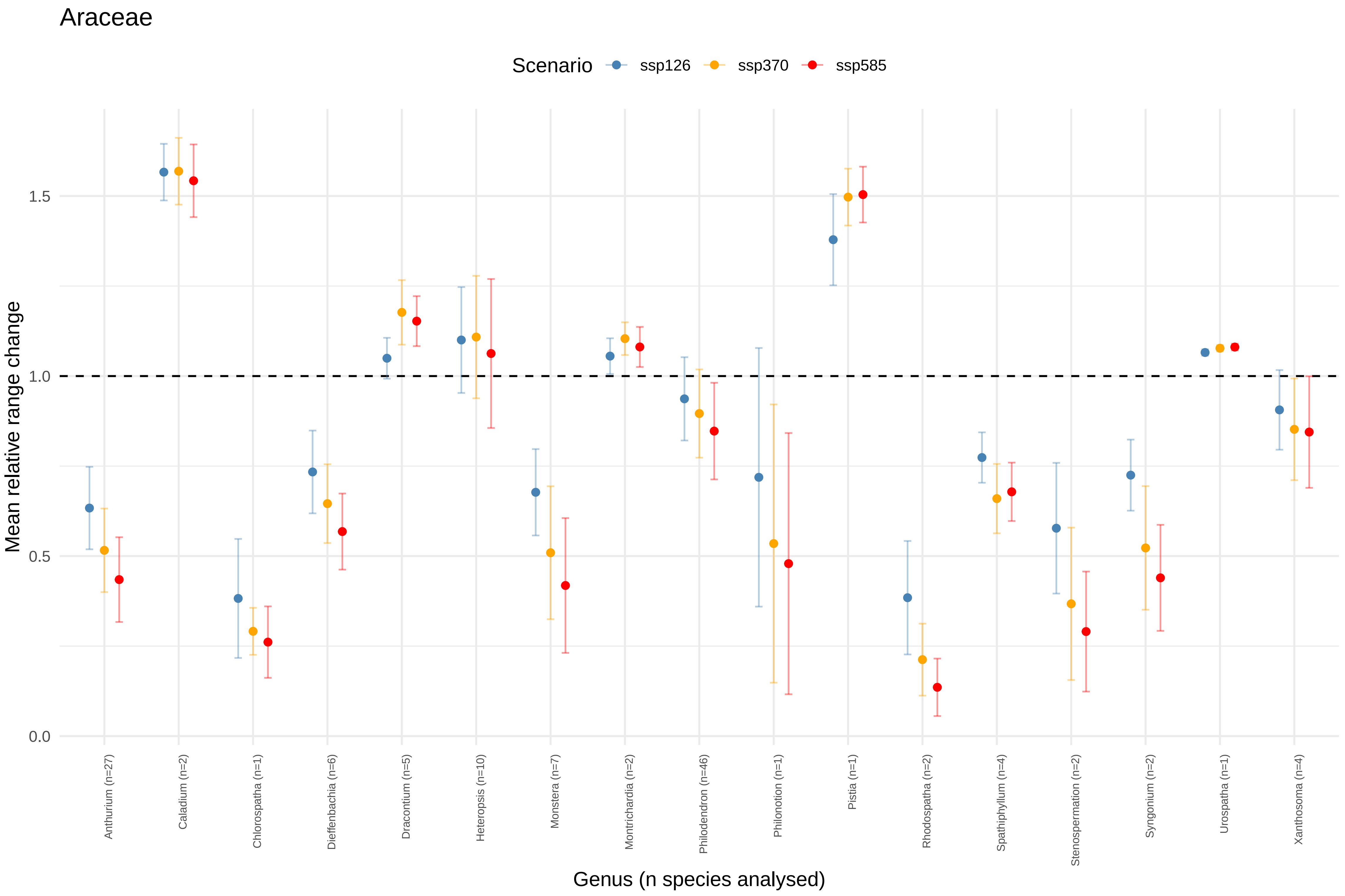

Araceae
Scenario
ssp126
ssp370
ssp585
Mean relative range change
1.5
1.0
0.5
0.0
Anthurium (n=27)
Caladium (n=2)
Chlorospatha (n=1)
Dieffenbachia (n=6)
Dracontium (n=5)
Heteropsis (n=10)
Monstera (n=7)
Montrichardia (n=2)
Philodendron (n=46)
Philonotion (n=1)
Pistia (n=1)
Rhodospatha (n=2)
Spathiphyllum (n=4)
Stenospermation (n=2)
Syngonium (n=2)
Urospatha (n=1)
Xanthosoma (n=4)
Genus (n species analysed)

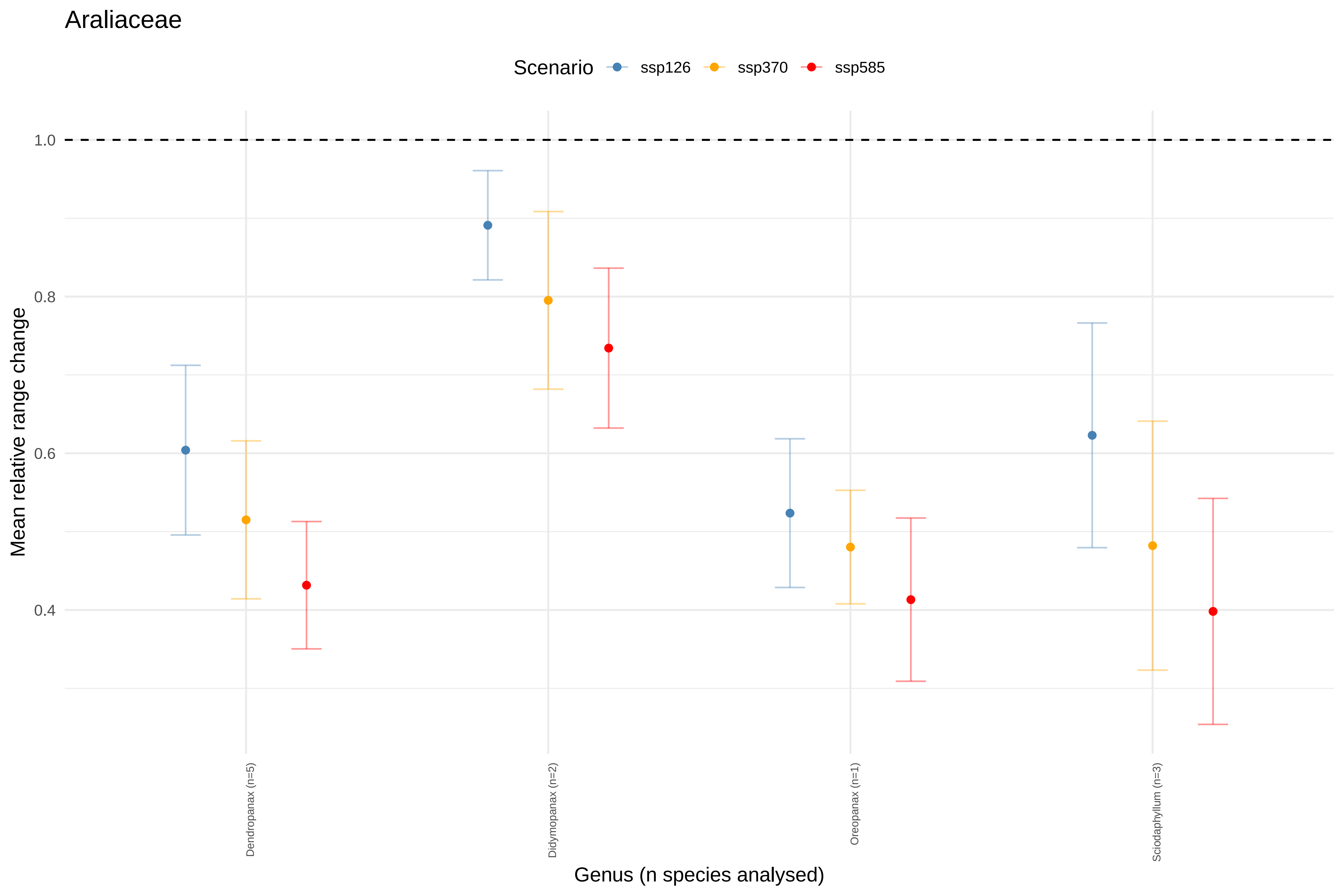
Araliaceae
Scenario
ssp126
ssp370
ssp585
1.0
0.8
0.6
0.4
Mean relative range change
Dendropanax (n=5)
Didymopanax (n=2)
Oreopanax (n=1)
Sciodaphyllum (n=3)
Genus (n species analysed)

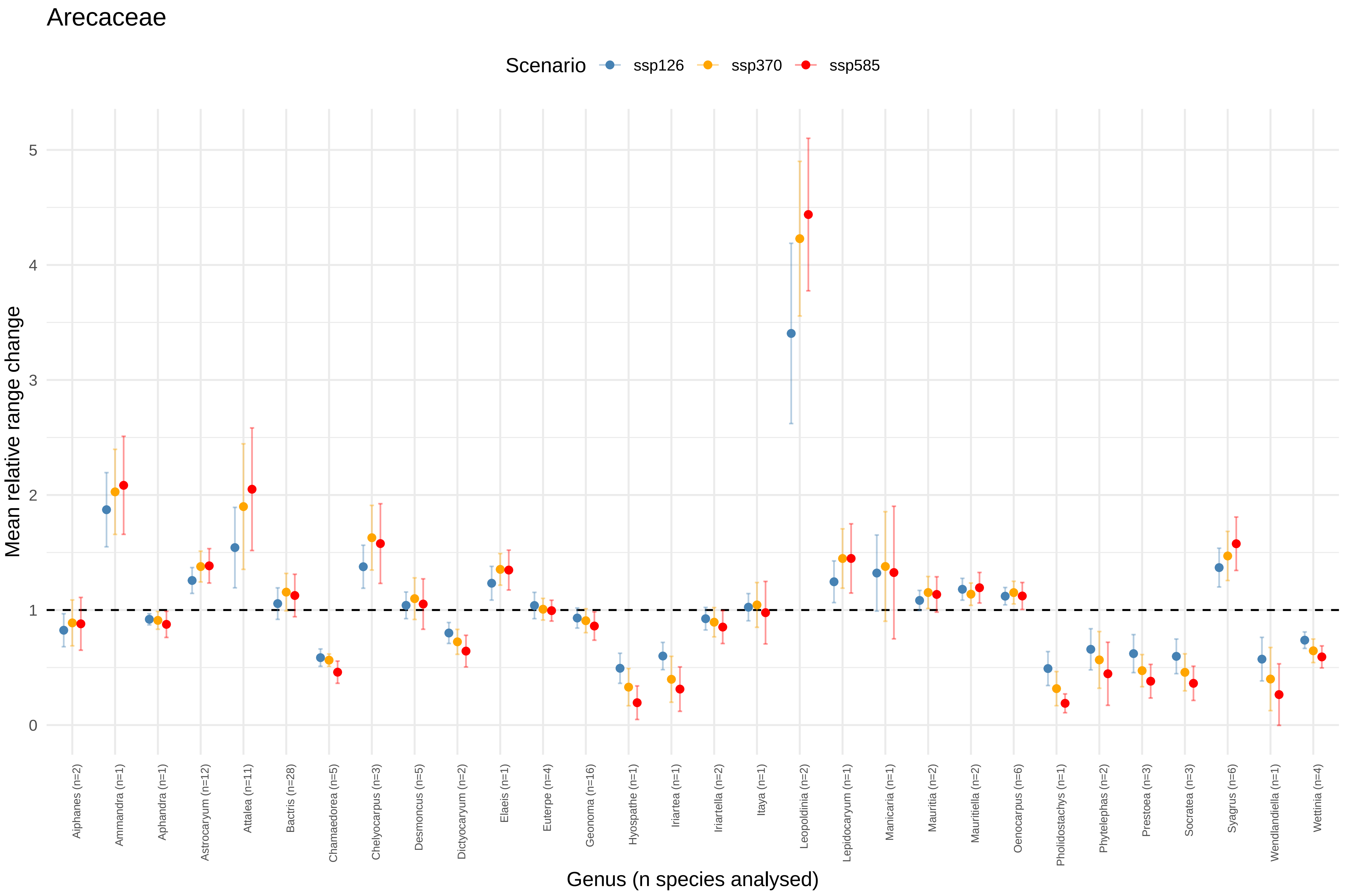

Arecaceae
Scenario
ssp126
ssp370
ssp585
Mean relative range change
0
1
2
3
4
5
Aiphanes (n=2)
Ammandra (n=1)
Aphandra (n=1)
Astrocaryum (n=12)
Attalea (n=11)
Bactris (n=28)
Chamaedorea (n=5)
Chelyocarpus (n=3)
Desmoncus (n=5)
Dictyocaryum (n=2)
Elaeis (n=1)
Euterpe (n=4)
Geonoma (n=16)
Hyospathe (n=1)
Iriartea (n=1)
Iriartella (n=2)
Itaya (n=1)
Leopoldinia (n=2)
Lepidocaryum (n=1)
Manicaria (n=1)
Mauritia (n=2)
Mauritiella (n=2)
Oenocarpus (n=6)
Pholidostachys (n=1)
Phytelephas (n=2)
Prestoea (n=3)
Socratea (n=3)
Syagrus (n=6)
Wendlandiella (n=1)
Wettinia (n=4)
Genus (n species analysed)

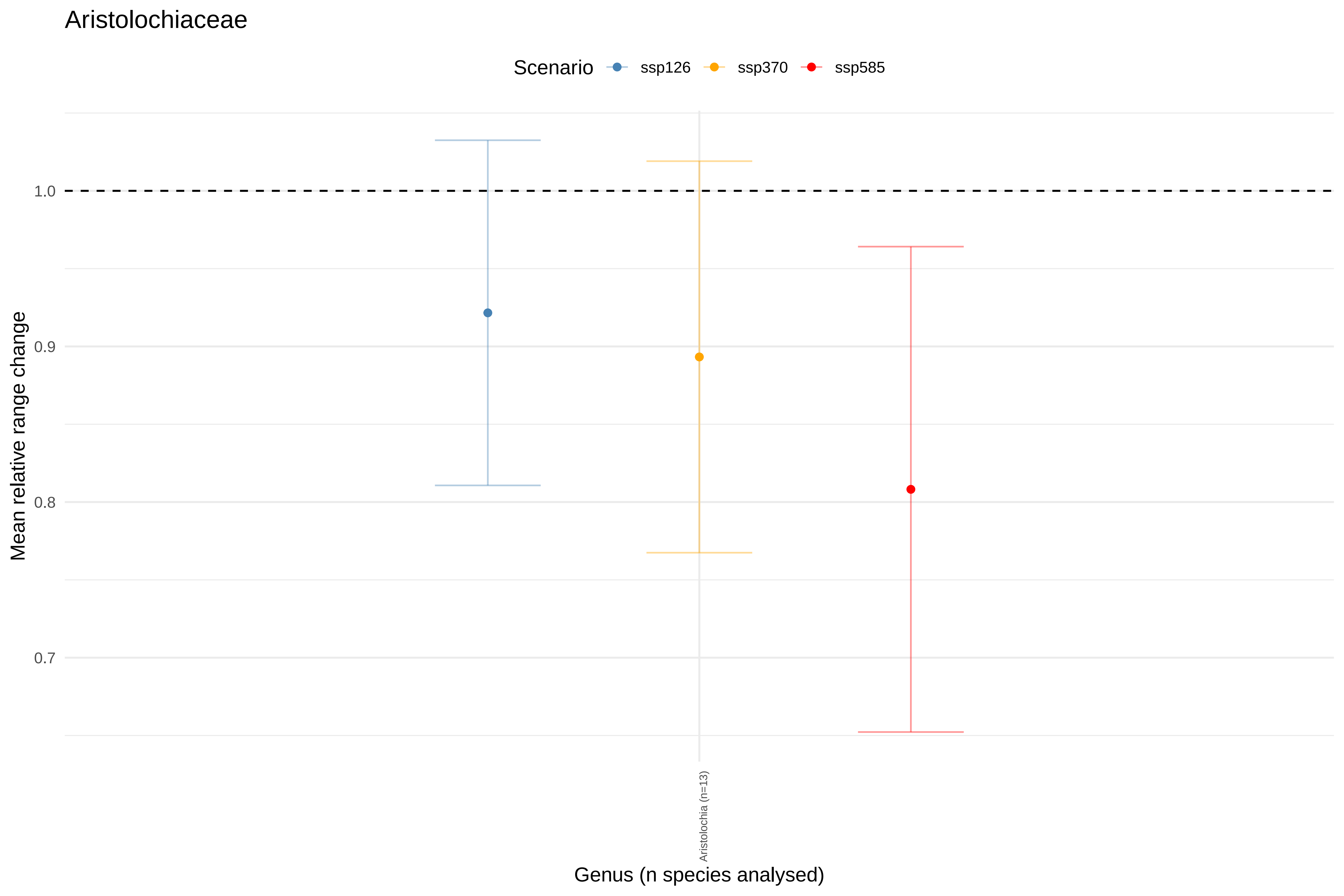

Aristolochiaceae
Scenario
ssp126
ssp370
ssp585
Mean relative range change
1.0
0.9
0.8
0.7
Aristolochia (n=13)
Genus (n species analysed)

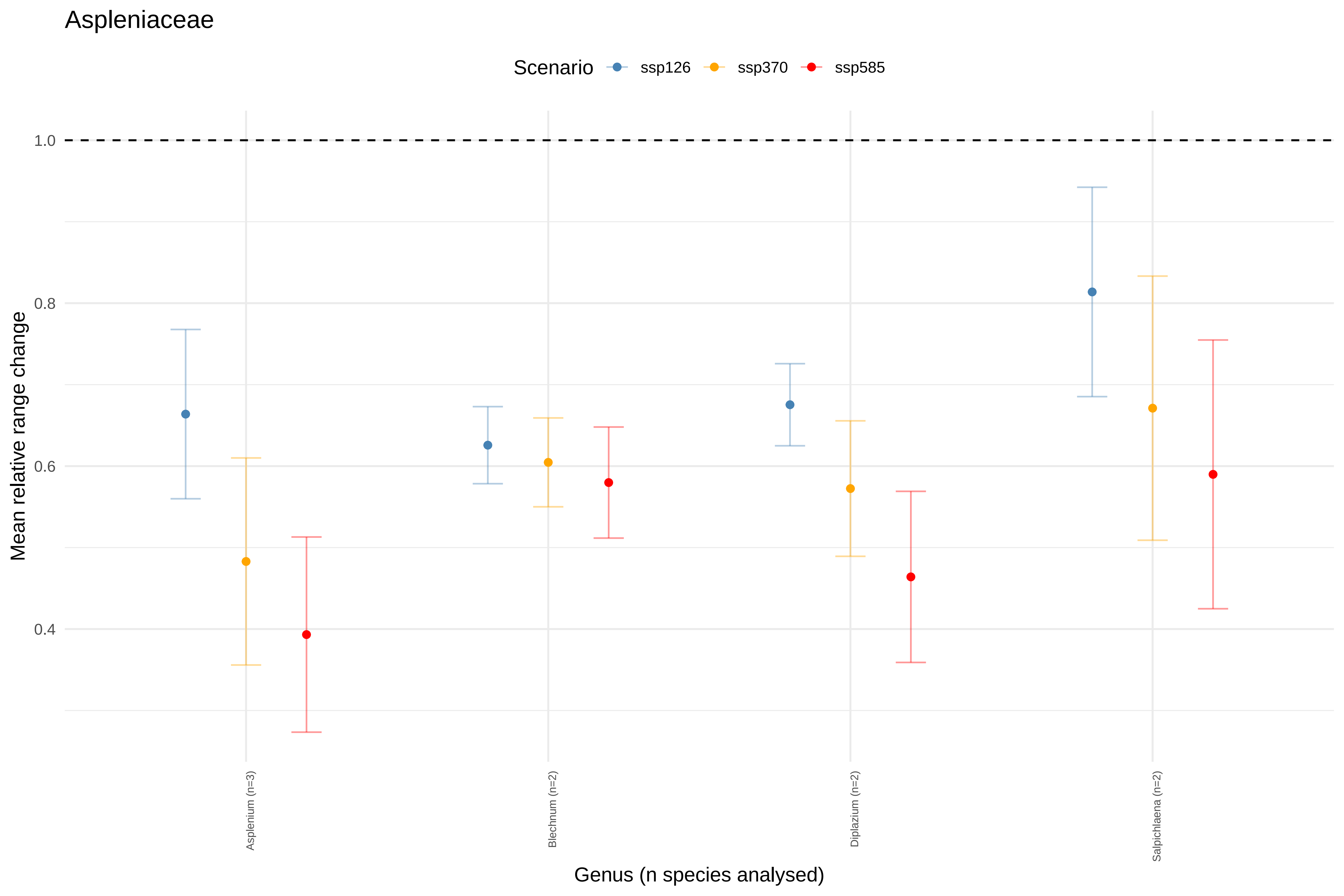

Aspleniaceae
Scenario
ssp126
ssp370
ssp585
1.0
0.8
0.6
0.4
Mean relative range change
Asplenium (n=3)
Blechnum (n=2)
Diplazium (n=2)
Salpichlaena (n=2)
Genus (n species analysed)

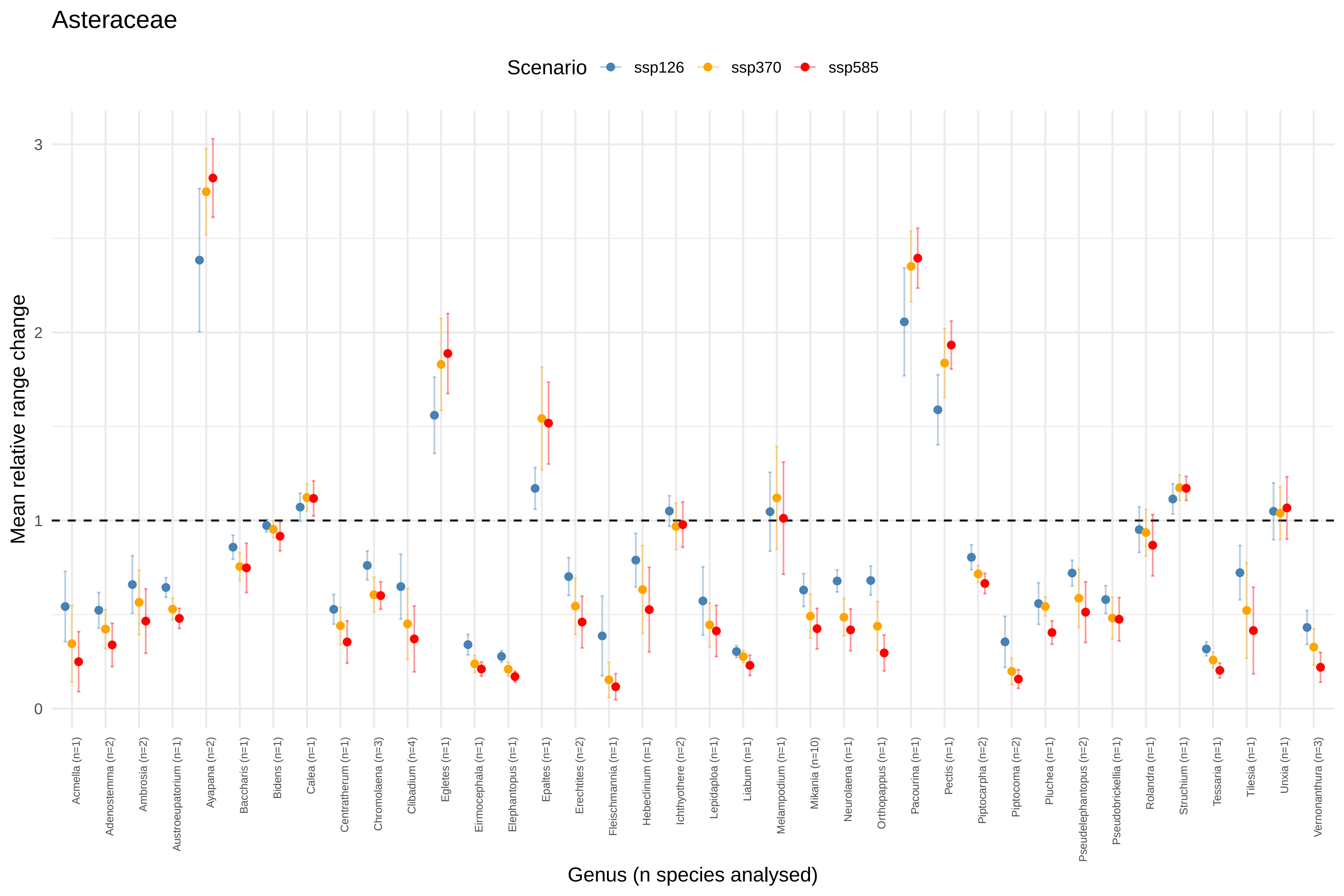

Asteraceae
Scenario
ssp126
ssp370
ssp585
Mean relative range change
0
1
2
3
Genus (n species analysed)
Acmella (n=1)
Adenostemma (n=2)
Ambrosia (n=2)
Austroeupatorium (n=1)
Ayapana (n=2)
Baccharis (n=1)
Bidens (n=1)
Calea (n=1)
Centratherum (n=1)
Chromolaena (n=3)
Clibadium (n=4)
Egletes (n=1)
Eirmocephala (n=1)
Elephantopus (n=1)
Epaltes (n=1)
Erechtites (n=2)
Fleischmannia (n=1)
Hebeclinium (n=1)
Ichthyothere (n=2)
Lepidaploa (n=1)
Liabum (n=1)
Melampodium (n=1)
Mikania (n=10)
Neurolaena (n=1)
Orthopappus (n=1)
Pacourina (n=1)
Pectis (n=1)
Piptocarpha (n=2)
Piptocoma (n=2)
Pluchea (n=1)
Pseudelephantopus (n=2)
Pseudobrickellia (n=1)
Rolandra (n=1)
Struchium (n=1)
Tessaria (n=1)
Tilesia (n=1)
Unxia (n=1)
Vernonanthura (n=3)

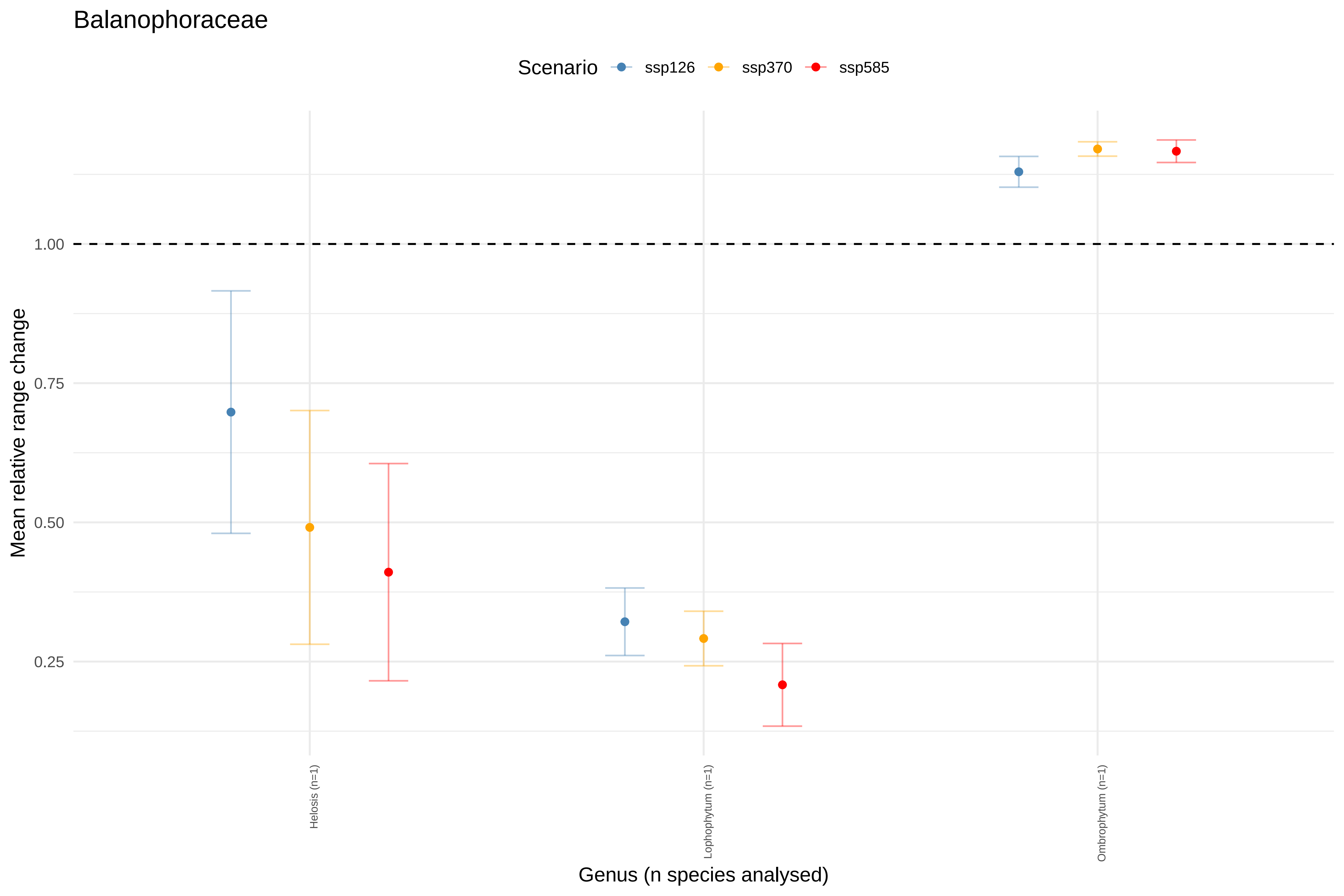

Balanophoraceae
Scenario
ssp126
ssp370
ssp585
Mean relative range change
1.00
0.75
0.50
0.25
Helosis (n=1)
Lophophytum (n=1)
Ombrophytum (n=1)
Genus (n species analysed)

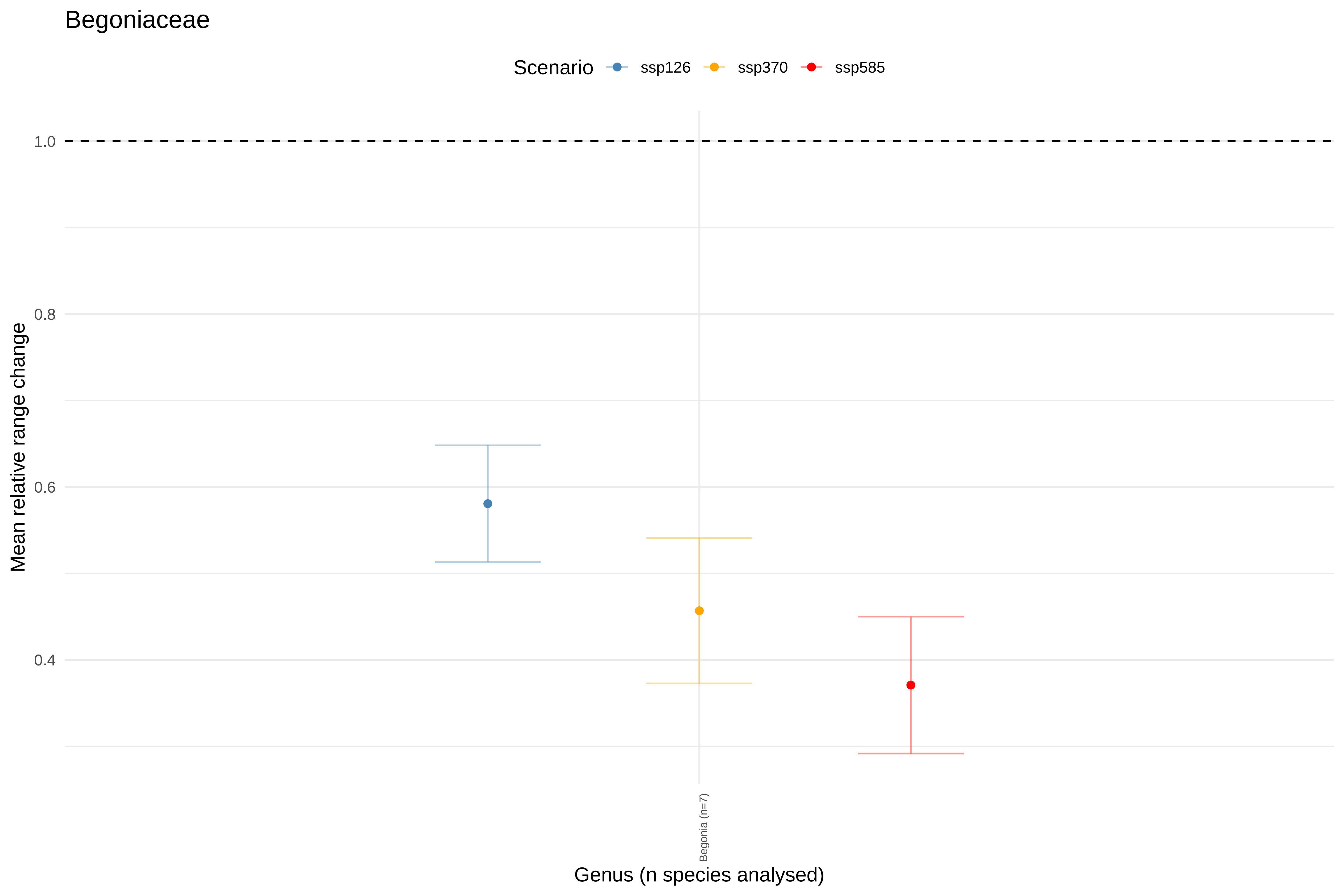

Begoniaceae
Scenario
ssp126
ssp370
ssp585
1.0
0.8
0.6
0.4
Mean relative range change
Begonia (n=7)
Genus (n species analysed)

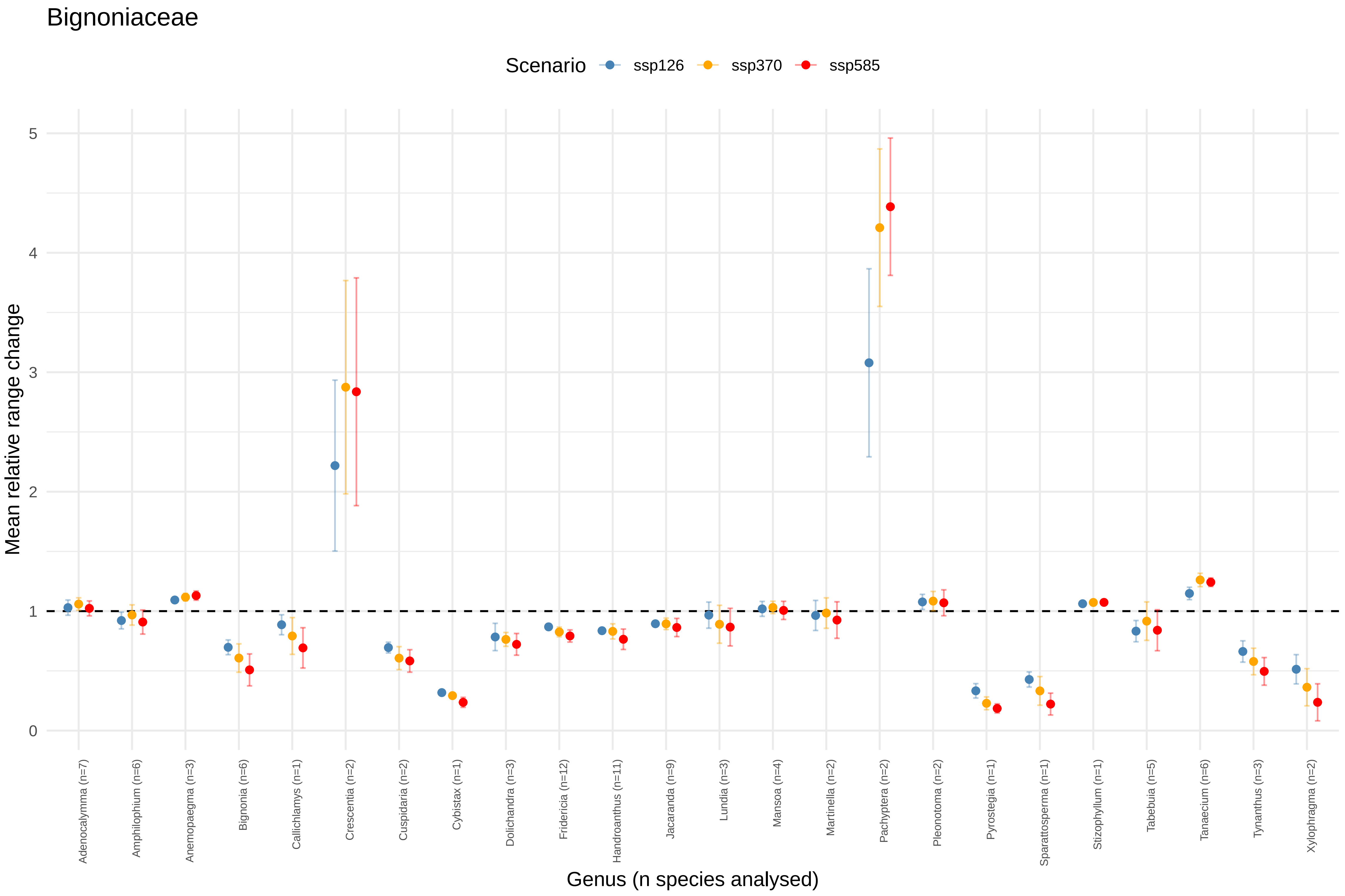

Bignoniaceae
Scenario
ssp126
ssp370
ssp585
Mean relative range change
0
1
2
3
4
5
Adenocalymma (n=7)
Amphilophium (n=6)
Anemopaegma (n=3)
Bignonia (n=6)
Callichlamys (n=1)
Crescentia (n=2)
Cuspidaria (n=2)
Cybistax (n=1)
Dolichandra (n=3)
Fridericia (n=12)
Handroanthus (n=11)
Jacaranda (n=9)
Lundia (n=3)
Mansoa (n=4)
Martinella (n=2)
Pachyptera (n=2)
Pleonotoma (n=2)
Pyrostegia (n=1)
Sparattosperma (n=1)
Stizophyllum (n=1)
Tabebuia (n=5)
Tanaecium (n=6)
Tynanthus (n=3)
Xylophragma (n=2)
Genus (n species analysed)

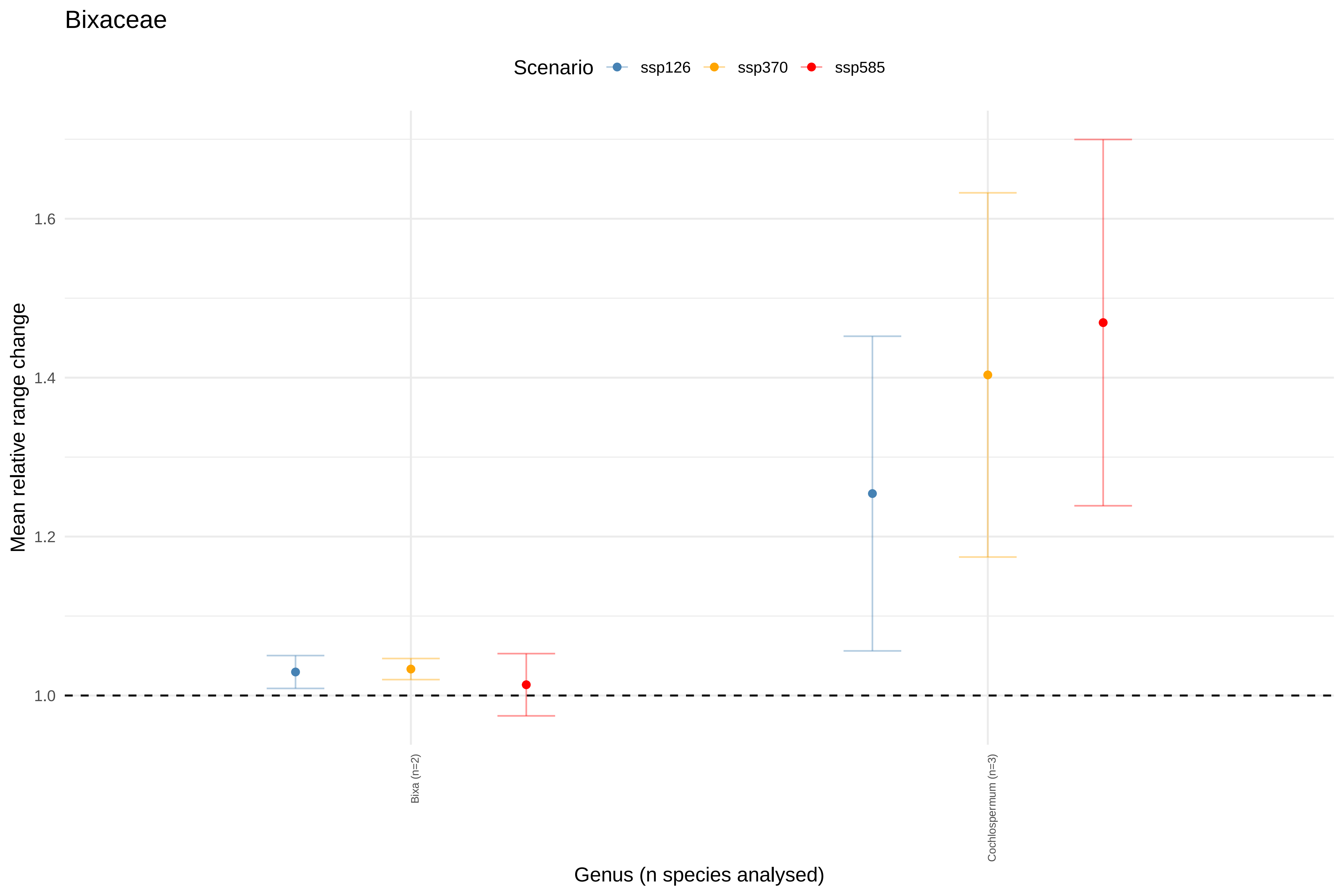

Bixaceae
Scenario
ssp126
ssp370
ssp585
Mean relative range change
1.6
1.4
1.2
1.0
Bixa (n=2)
Cochlospermum (n=3)
Genus (n species analysed)

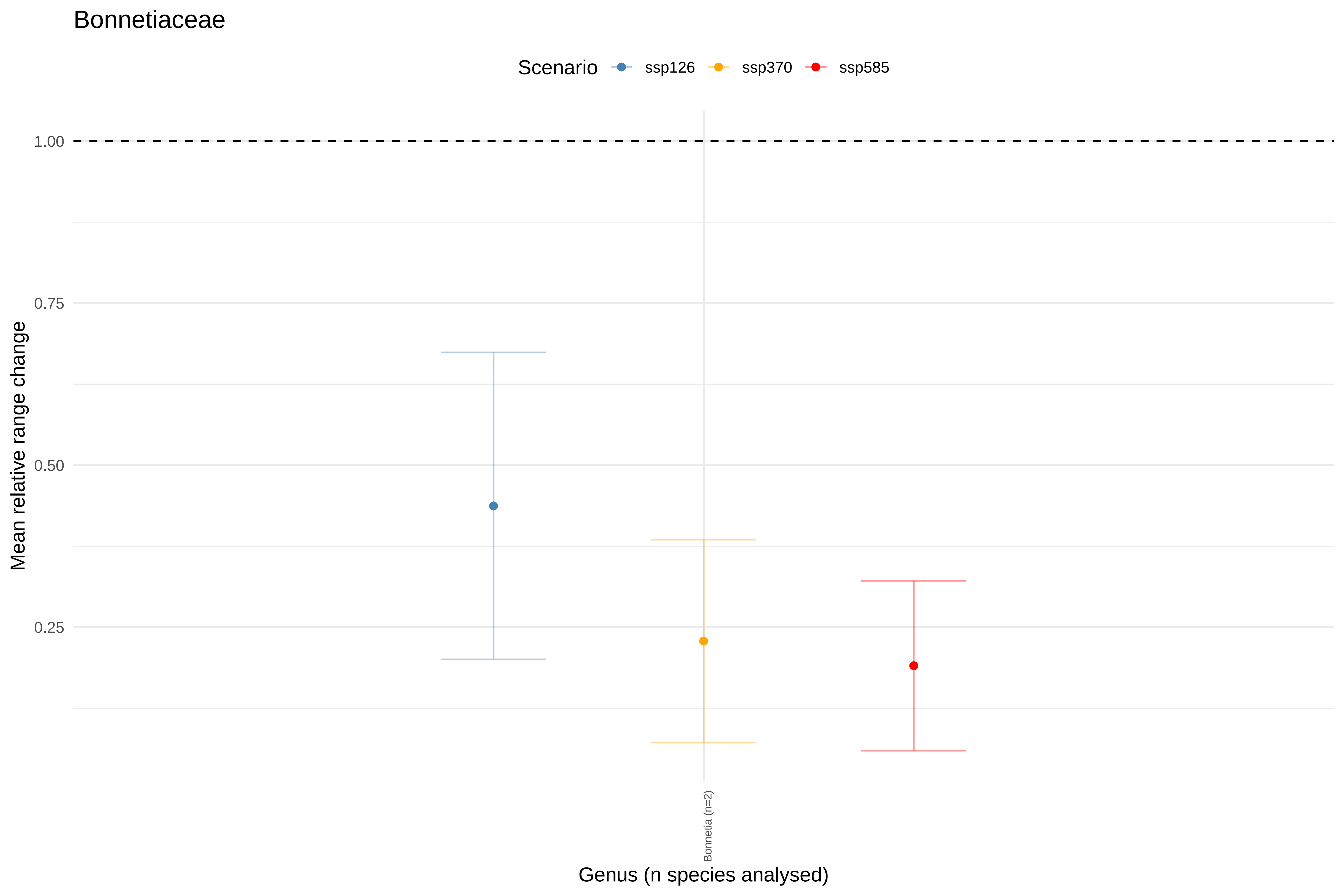
Bonnetiaceae
Scenario
ssp126
ssp370
ssp585
1.00
0.75
0.50
0.25
Mean relative range change
Bonnetia (n=2)
Genus (n species analysed)

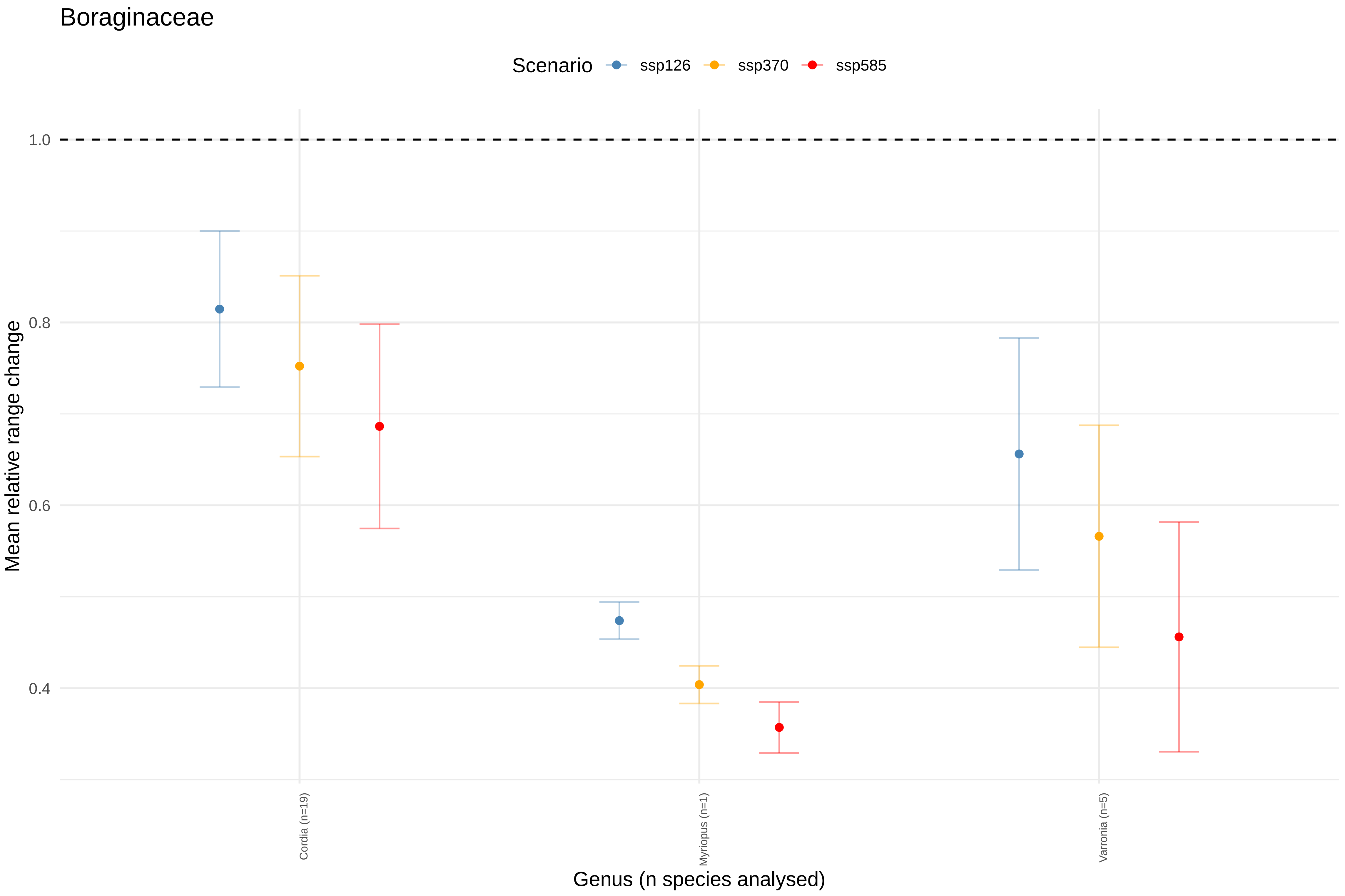

Boraginaceae
Scenario
ssp126
ssp370
ssp585
1.0
0.8
0.6
0.4
Mean relative range change
Cordia (n=19)
Myriopus (n=1)
Varronia (n=5)
Genus (n species analysed)

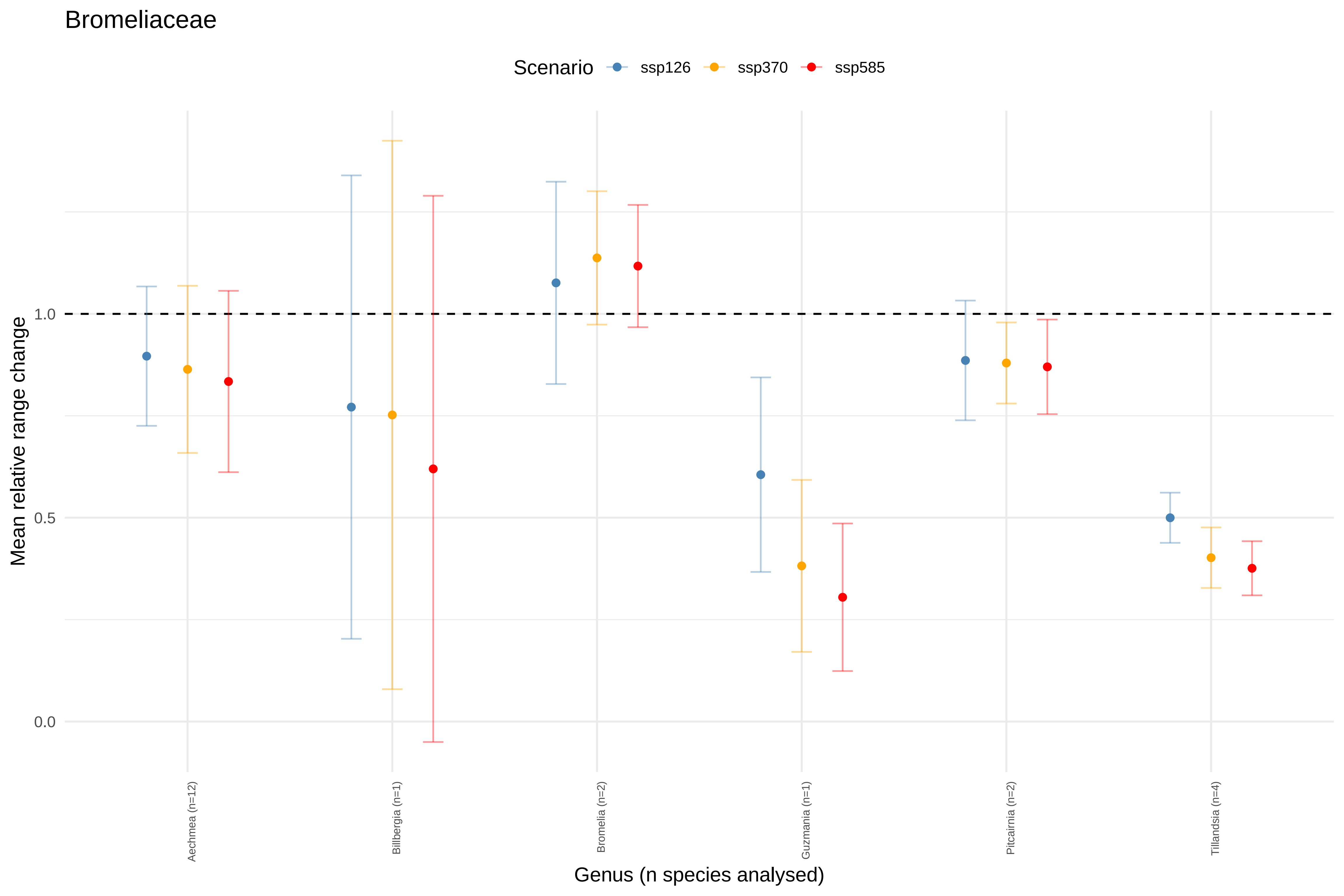

Bromeliaceae
Scenario
ssp126
ssp370
ssp585
Mean relative range change
1.0
0.5
0.0
Aechmea (n=12)
Billbergia (n=1)
Bromelia (n=2)
Guzmania (n=1)
Pitcairnia (n=2)
Tillandsia (n=4)
Genus (n species analysed)

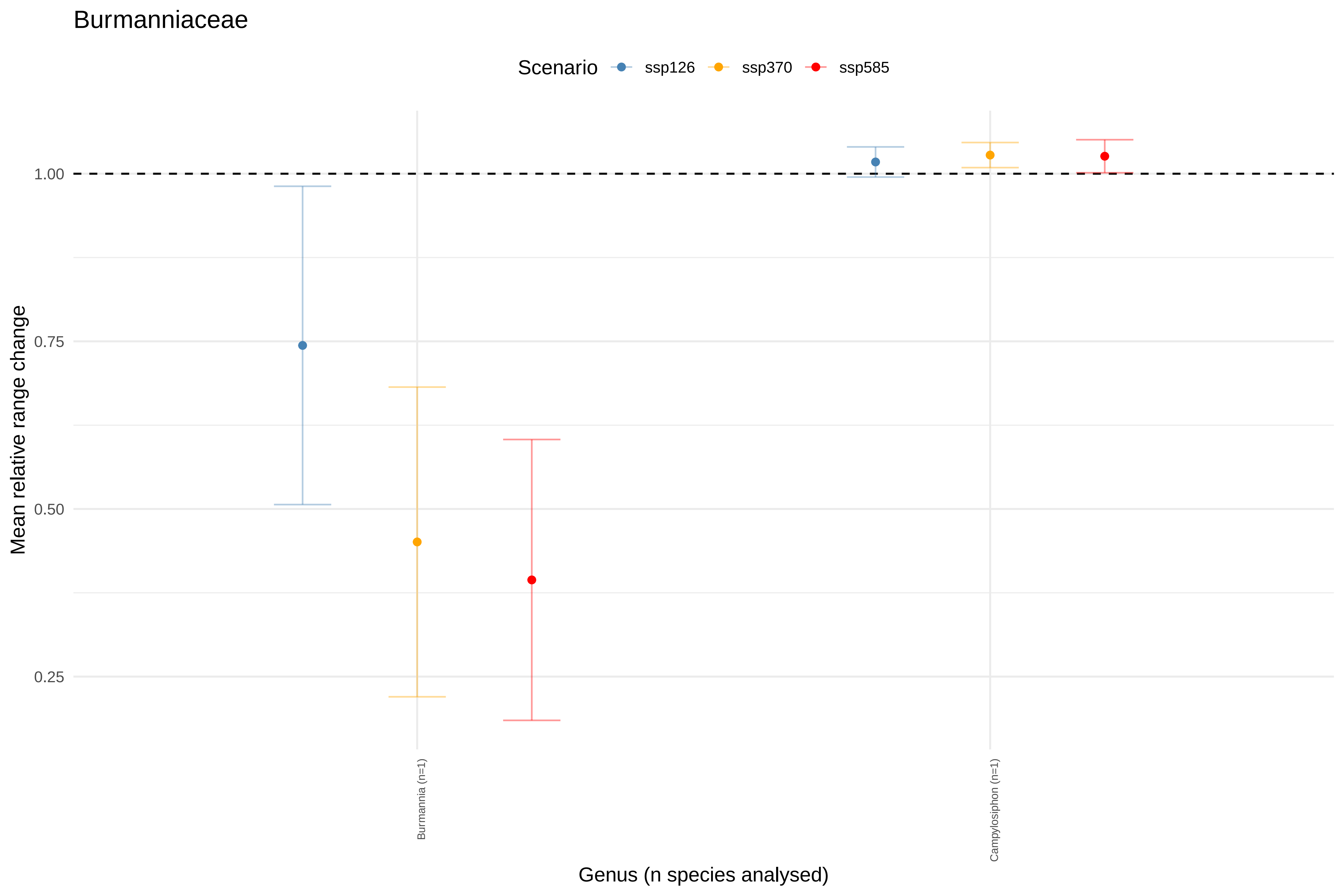
Burmanniaceae
Scenario
ssp126
ssp370
ssp585
Mean relative range change
1.00
0.75
0.50
0.25
Burmannia (n=1)
Campylosiphon (n=1)
Genus (n species analysed)

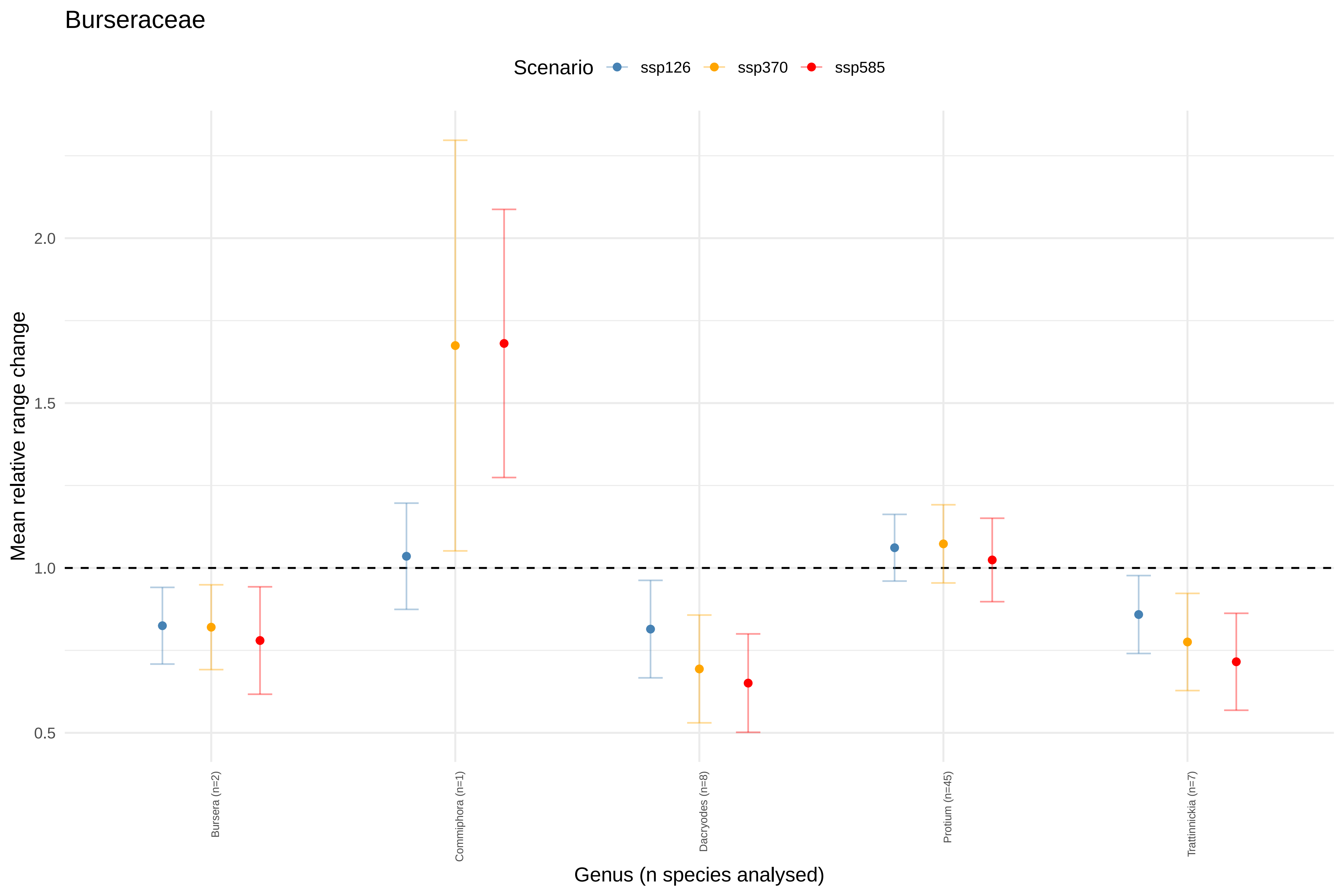

Burseraceae
Scenario
ssp126
ssp370
ssp585
Mean relative range change
2.0
1.5
1.0
0.5
Bursera (n=2)
Commiphora (n=1)
Dacryodes (n=8)
Protium (n=45)
Trattinnickia (n=7)
Genus (n species analysed)

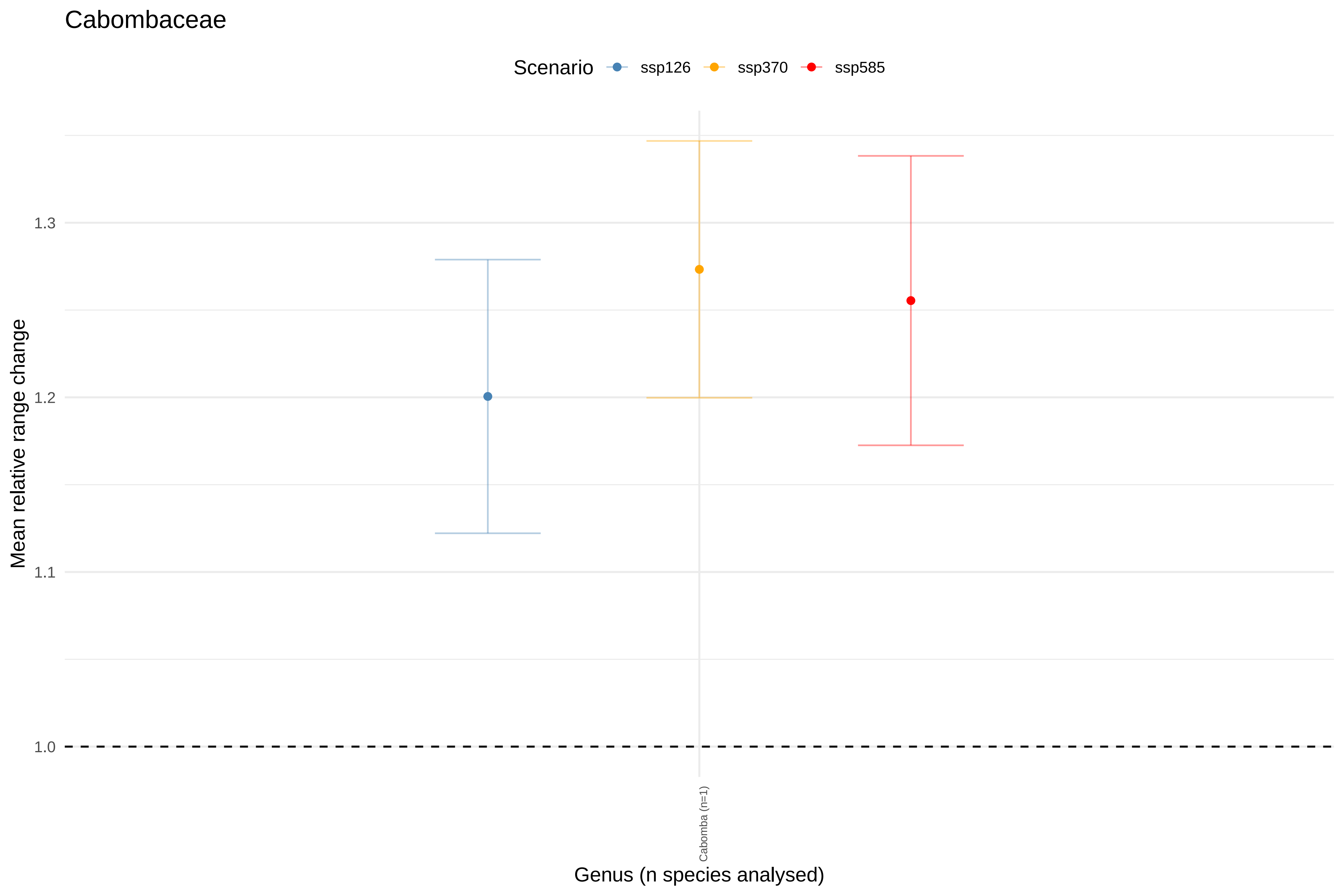

Cabombaceae
Scenario
ssp126
ssp370
ssp585
Mean relative range change
1.3
1.2
1.1
1.0
Cabomba (n=1)
Genus (n species analysed)

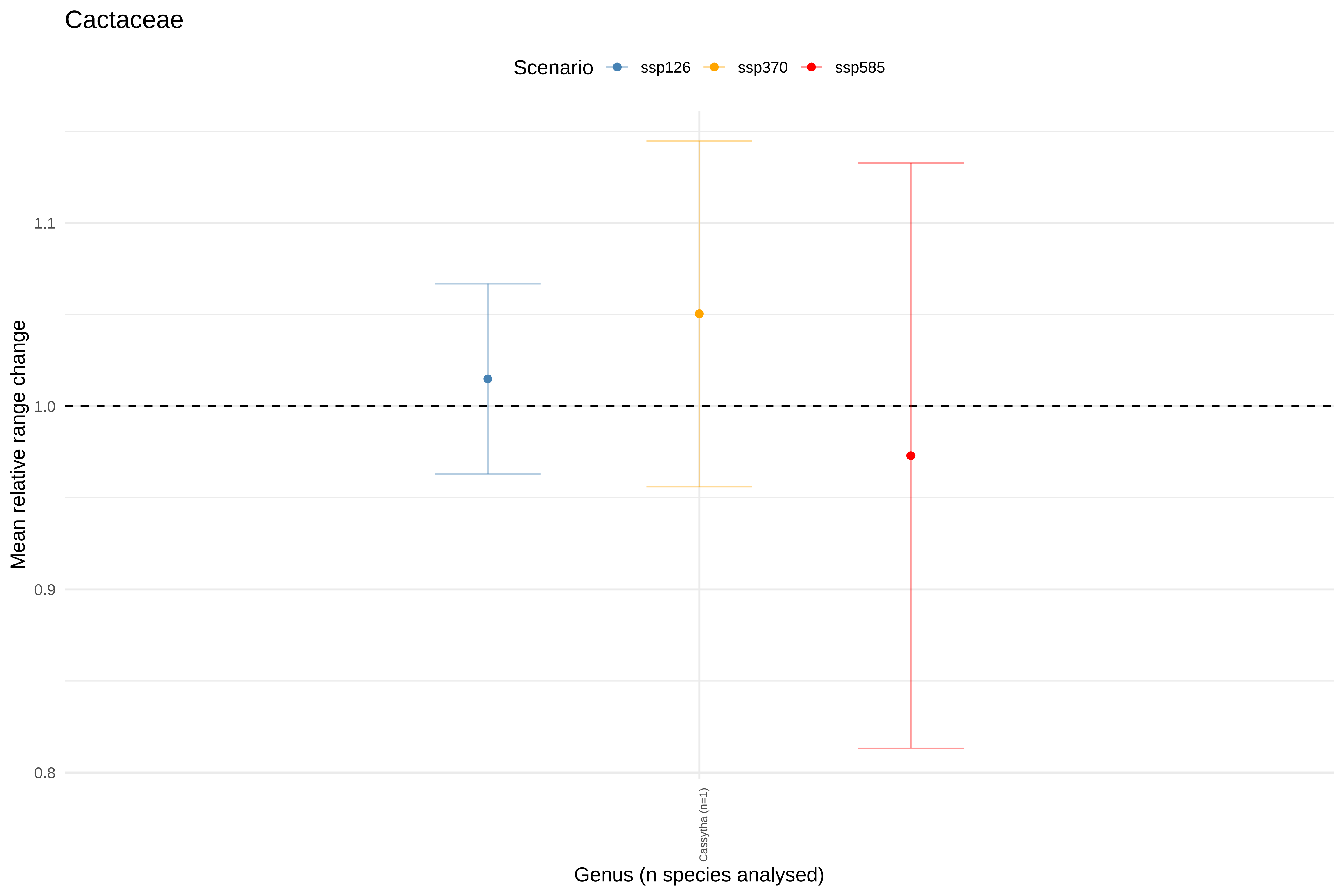
Cactaceae
Scenario
ssp126
ssp370
ssp585
Mean relative range change
1.1
1.0
0.9
0.8
Cassytha (n=1)
Genus (n species analysed)

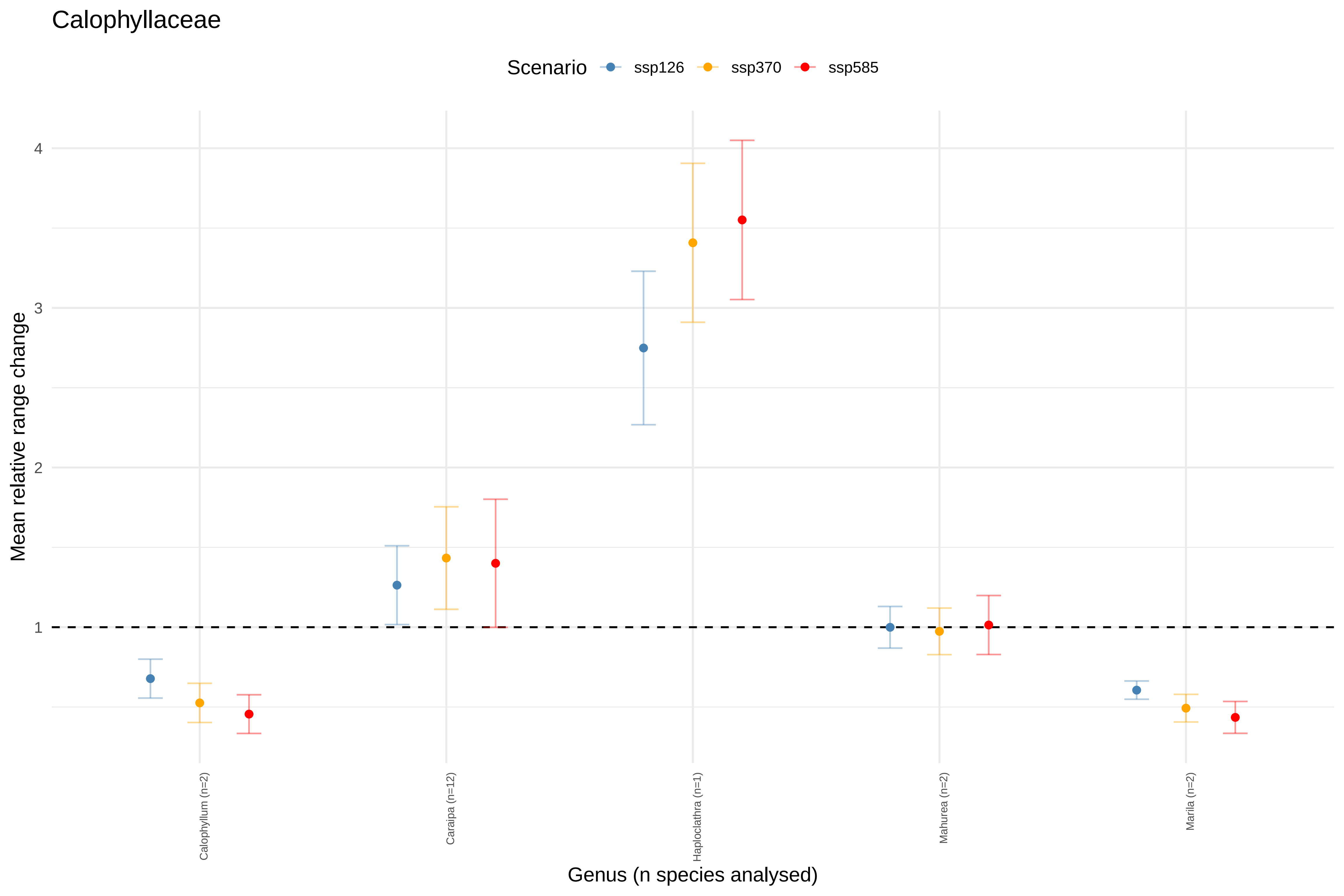

Calophyllaceae
Scenario
ssp126
ssp370
ssp585
Mean relative range change
4
3
2
1
Calophyllum (n=2)
Caraipa (n=12)
Haploclathra (n=1)
Mahurea (n=2)
Marila (n=2)
Genus (n species analysed)

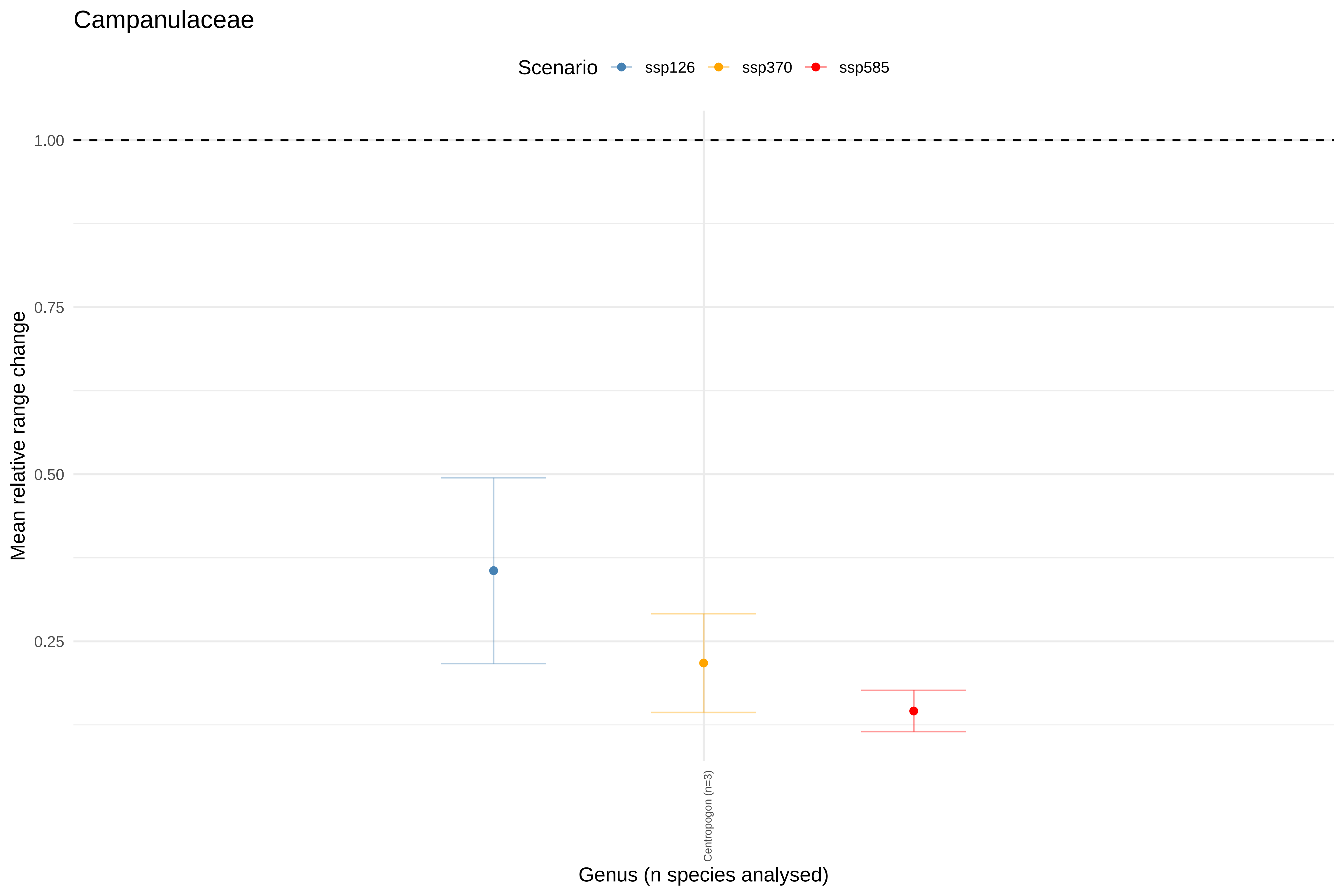
Campanulaceae
Scenario
ssp126
ssp370
ssp585
1.00
0.75
0.50
0.25
Mean relative range change
Centropogon (n=3)
Genus (n species analysed)

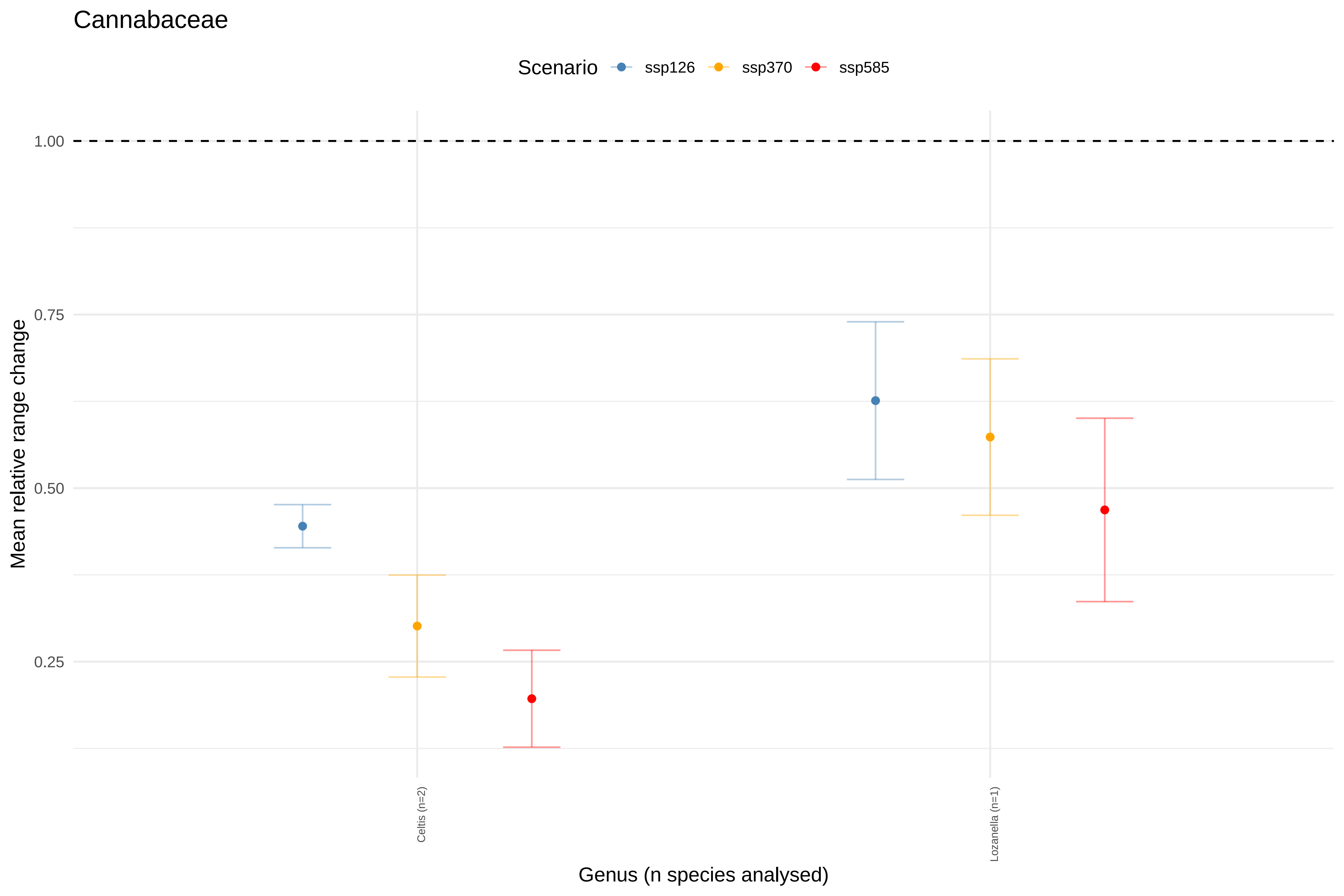

Cannabaceae
Scenario
ssp126
ssp370
ssp585
1.00
0.75
0.50
0.25
Mean relative range change
Celtis (n=2)
Lozanella (n=1)
Genus (n species analysed)

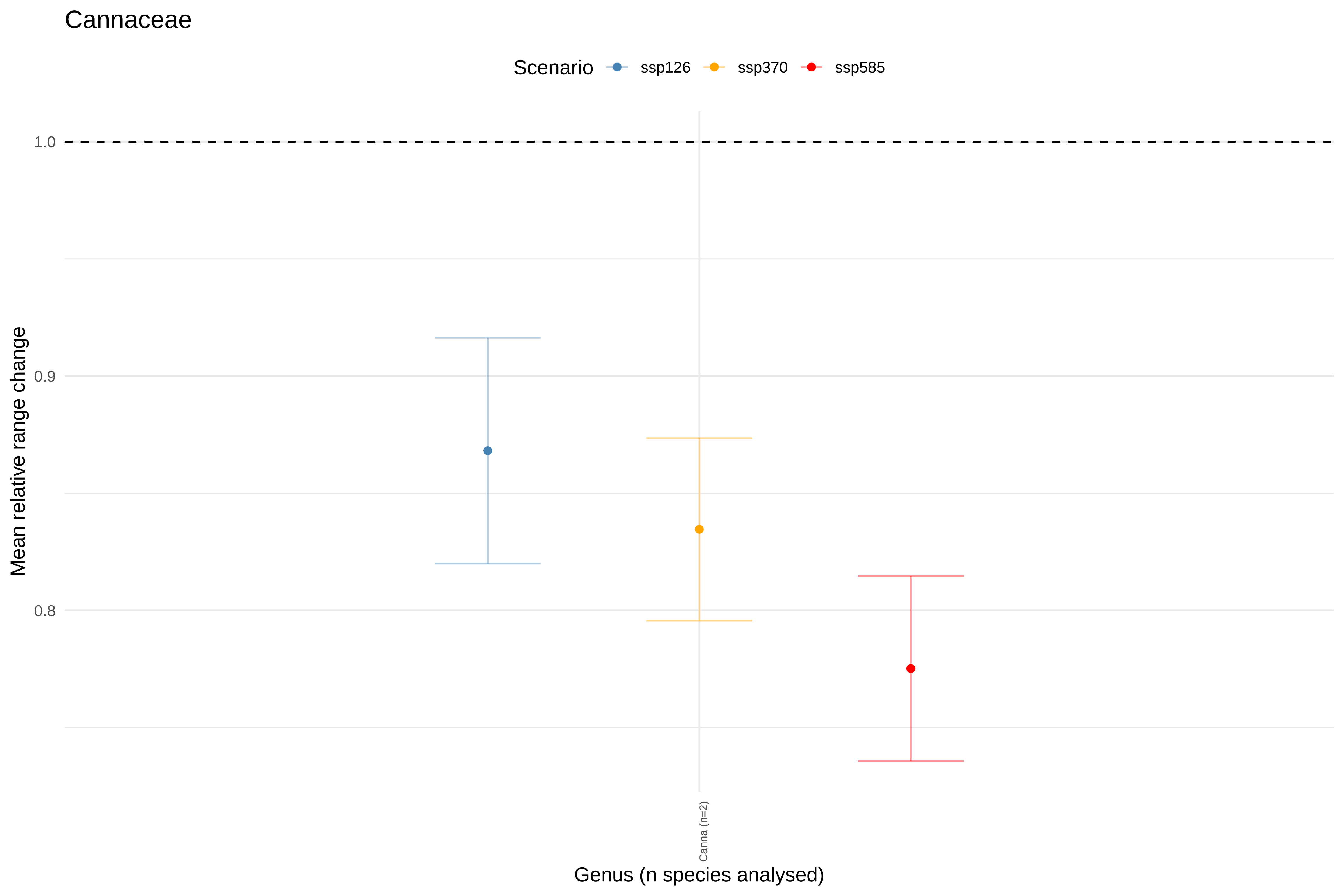

Cannaceae
Scenario
ssp126
ssp370
ssp585
1.0
0.9
0.8
Mean relative range change
Canna (n=2)
Genus (n species analysed)

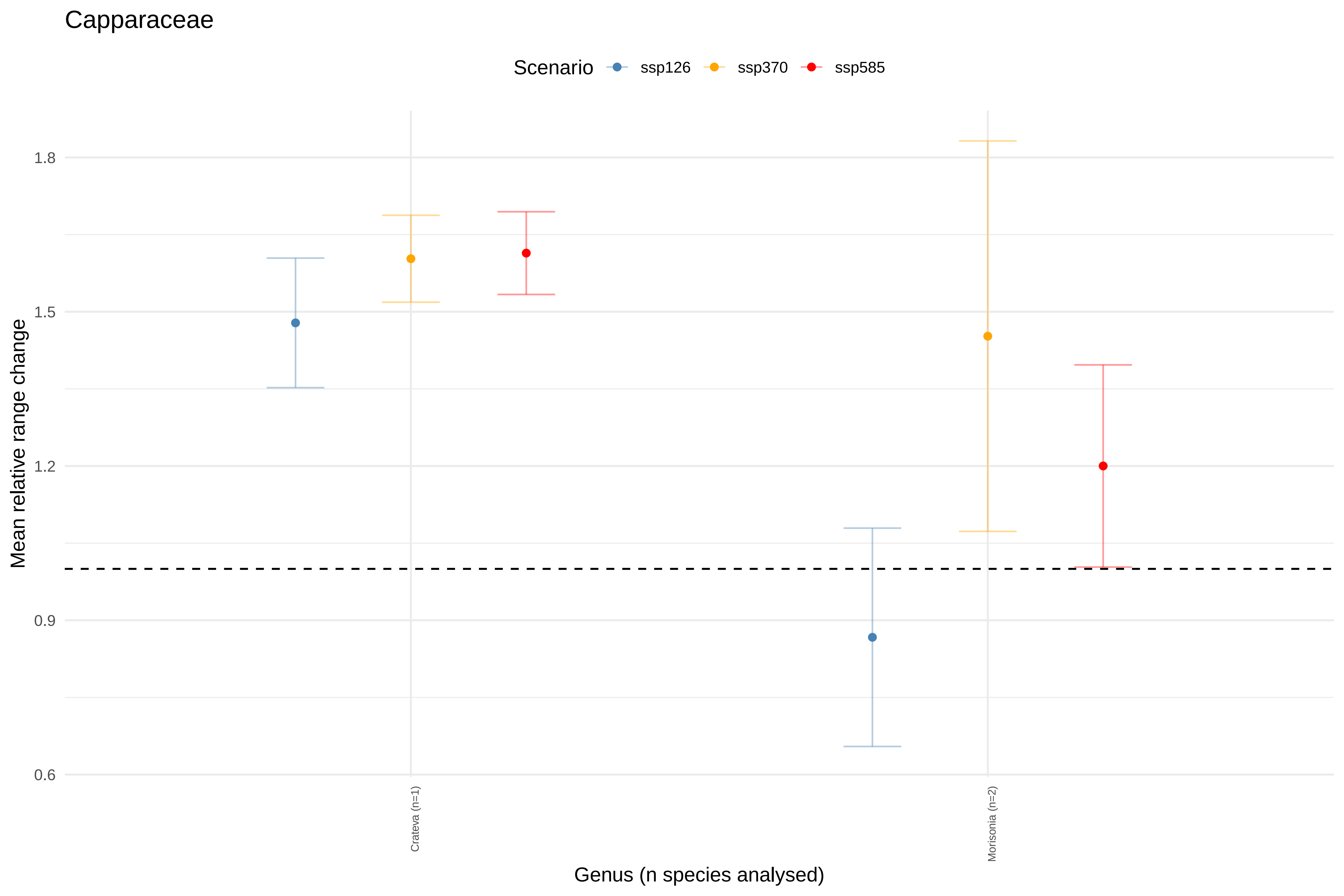

Capparaceae
Scenario
ssp126
ssp370
ssp585
Mean relative range change
1.8
1.5
1.2
0.9
0.6
Crateva (n=1)
Morisonia (n=2)
Genus (n species analysed)

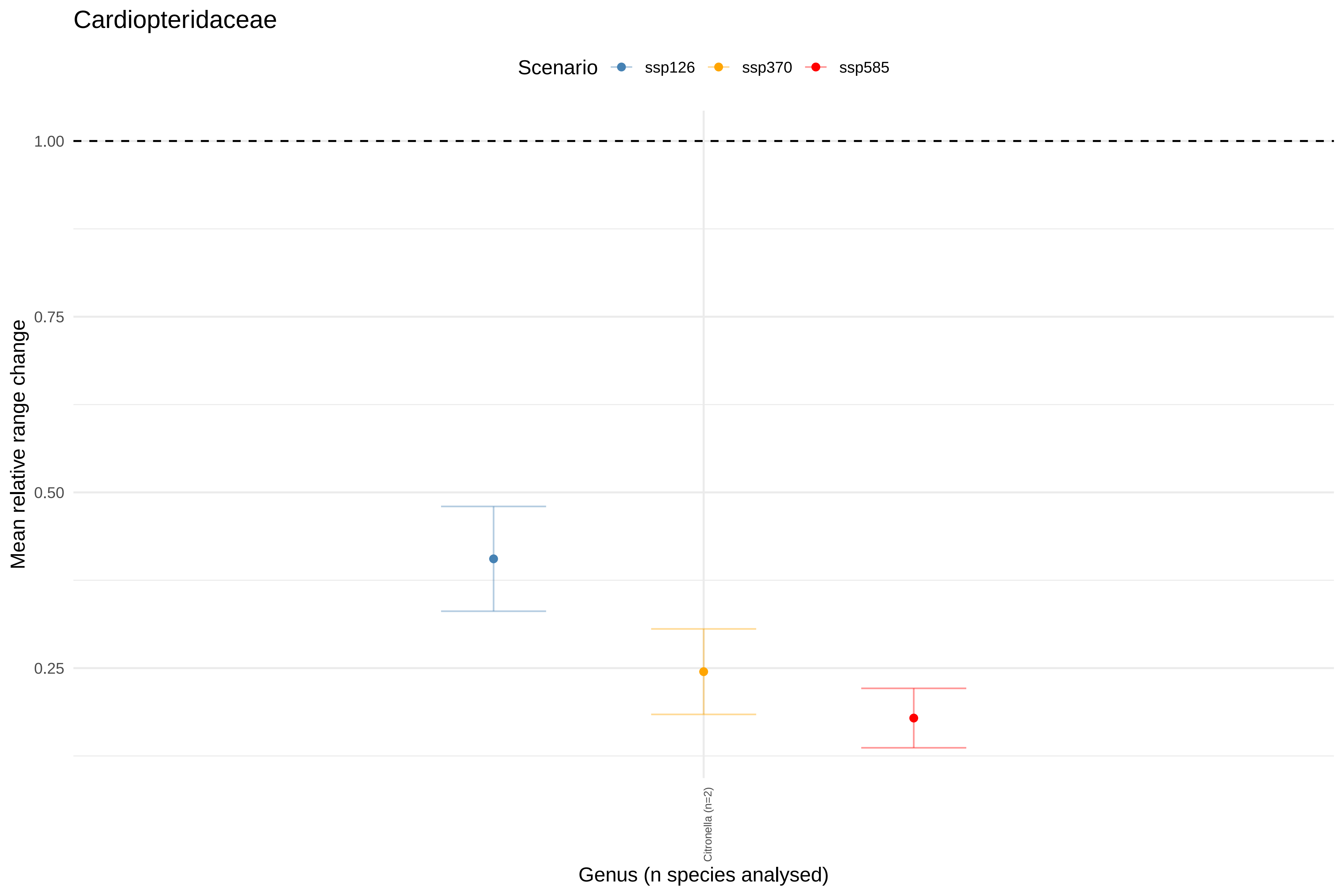

Cardiopteridaceae
Scenario
ssp126
ssp370
ssp585
1.00
0.75
0.50
0.25
Mean relative range change
Citronella (n=2)
Genus (n species analysed)

Caricaceae

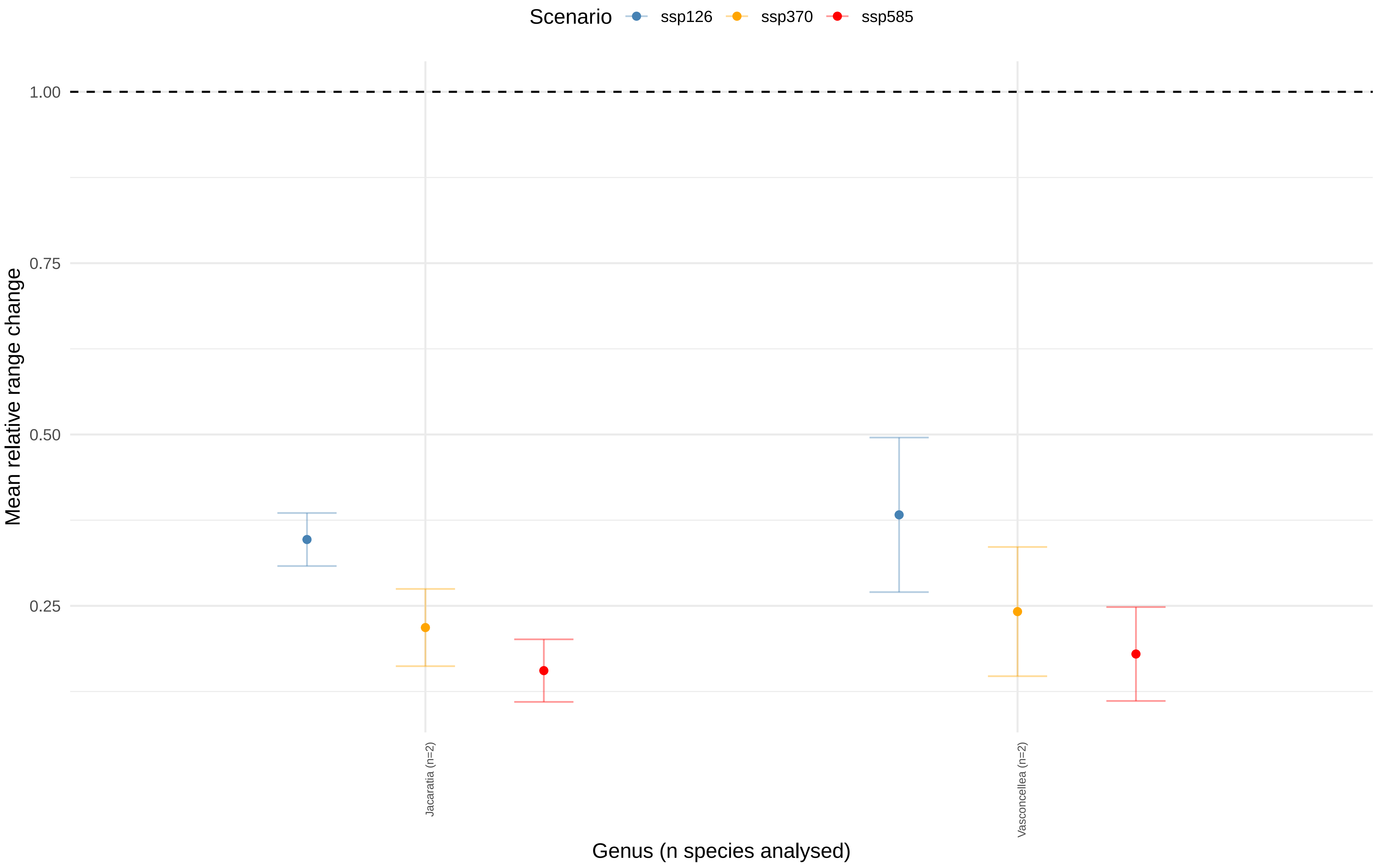
Scenario
ssp126
ssp370
ssp585
Mean relative range change
1.00
0.75
0.50
0.25
Jacaratia (n=2)
Vasconcellea (n=2)
Genus (n species analysed)

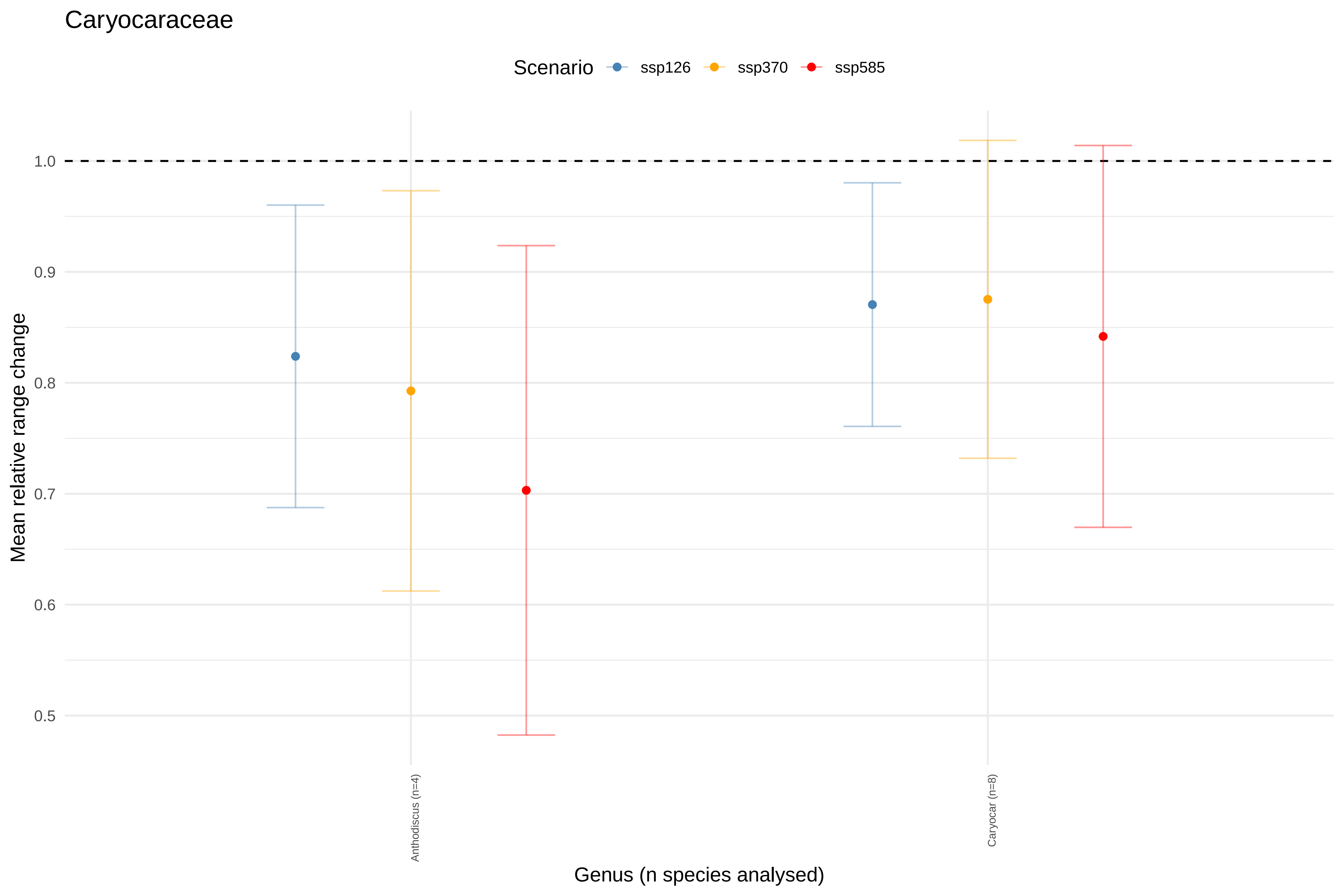

Caryocaraceae
Scenario
ssp126
ssp370
ssp585
Mean relative range change
1.0
0.9
0.8
0.7
0.6
0.5
Anthodiscus (n=4)
Caryocar (n=8)
Genus (n species analysed)

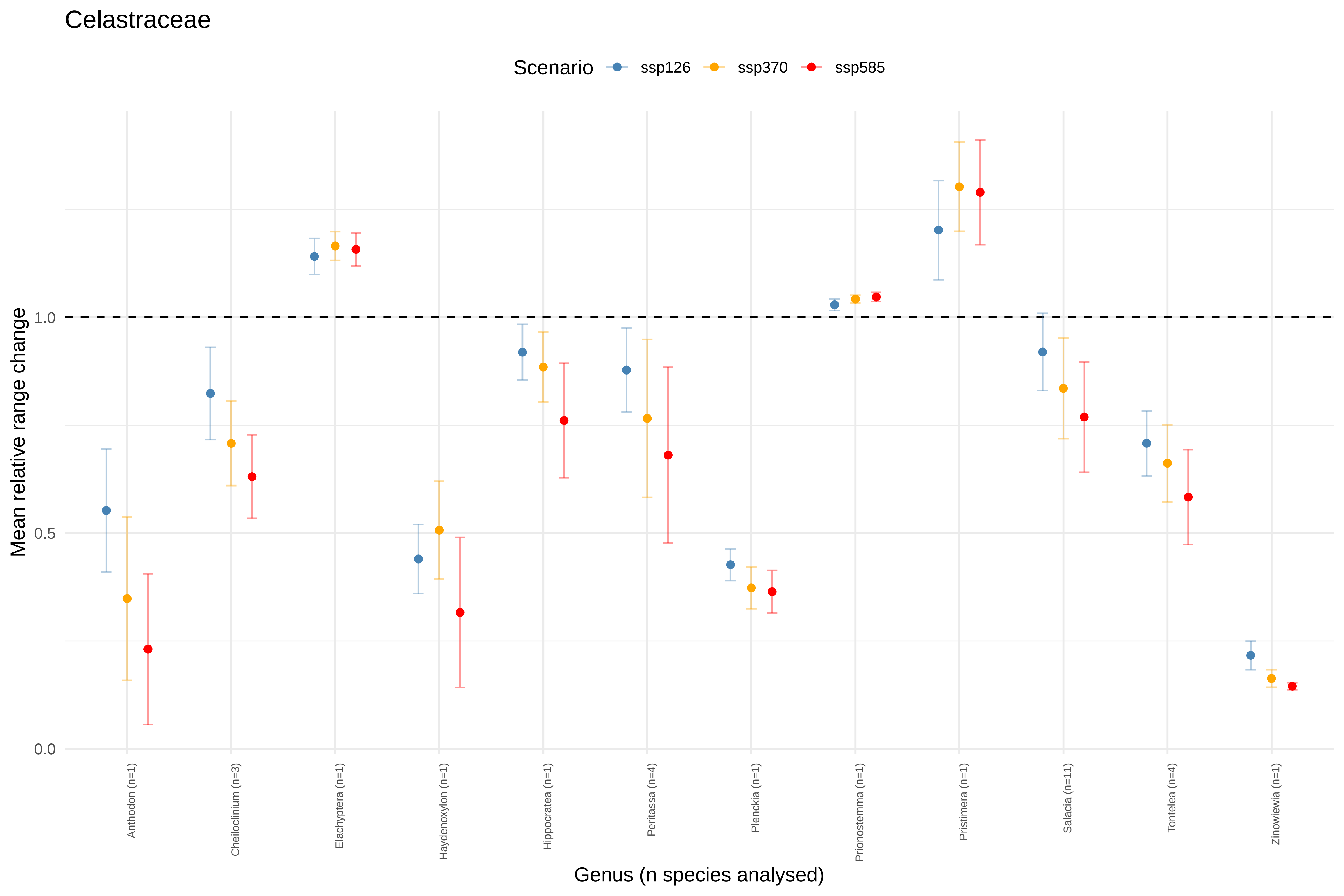

Celastraceae
Scenario
ssp126
ssp370
ssp585
Mean relative range change
1.0
0.5
0.0
Anthodon (n=1)
Cheiloclinium (n=3)
Elachyptera (n=1)
Haydenoxylon (n=1)
Hippocratea (n=1)
Peritassa (n=4)
Plenckia (n=1)
Prionostemma (n=1)
Pristimera (n=1)
Salacia (n=11)
Tontelea (n=4)
Zinowiewia (n=1)
Genus (n species analysed)

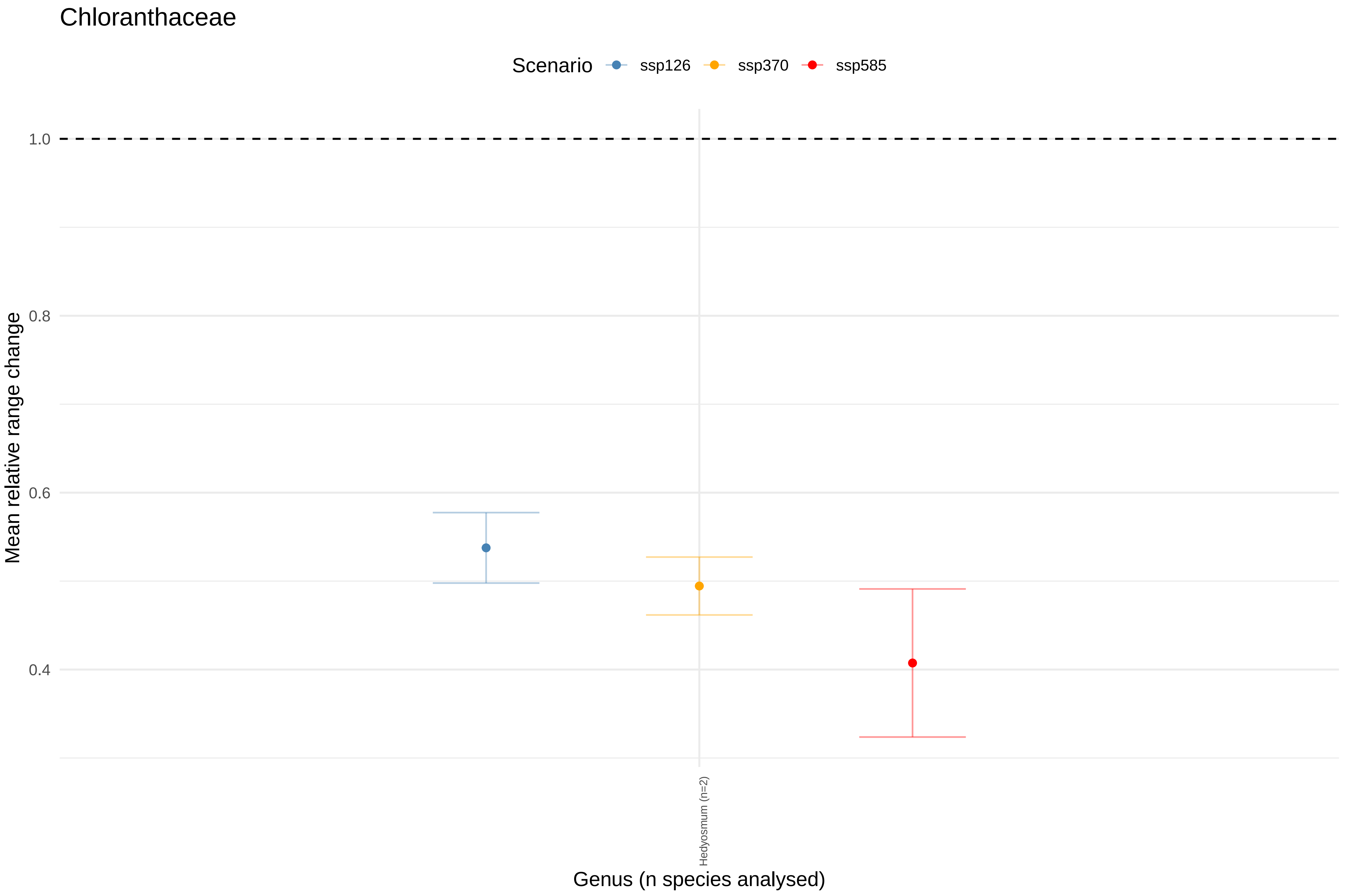
Chloranthaceae
Scenario
ssp126
ssp370
ssp585
1.0
0.8
0.6
0.4
Mean relative range change
Hedyosmum (n=2)
Genus (n species analysed)

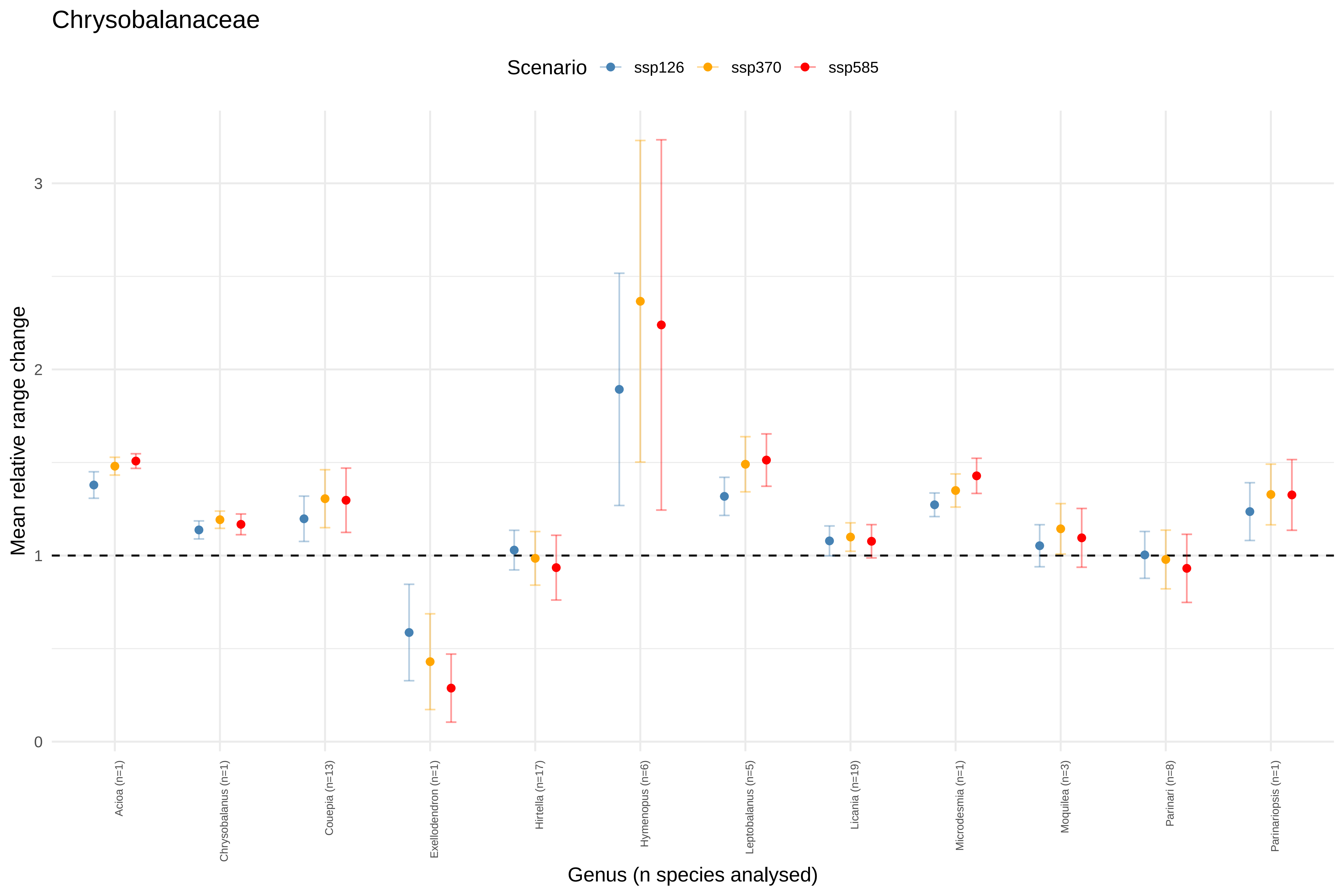

Chrysobalanaceae
Scenario
ssp126
ssp370
ssp585
Mean relative range change
0
1
2
3
Acioa (n=1)
Chrysobalanus (n=1)
Couepia (n=13)
Exellodendron (n=1)
Hirtella (n=17)
Hymenopus (n=6)
Leptobalanus (n=5)
Licania (n=19)
Microdesmia (n=1)
Moquilea (n=3)
Parinari (n=8)
Parinariopsis (n=1)
Genus (n species analysed)

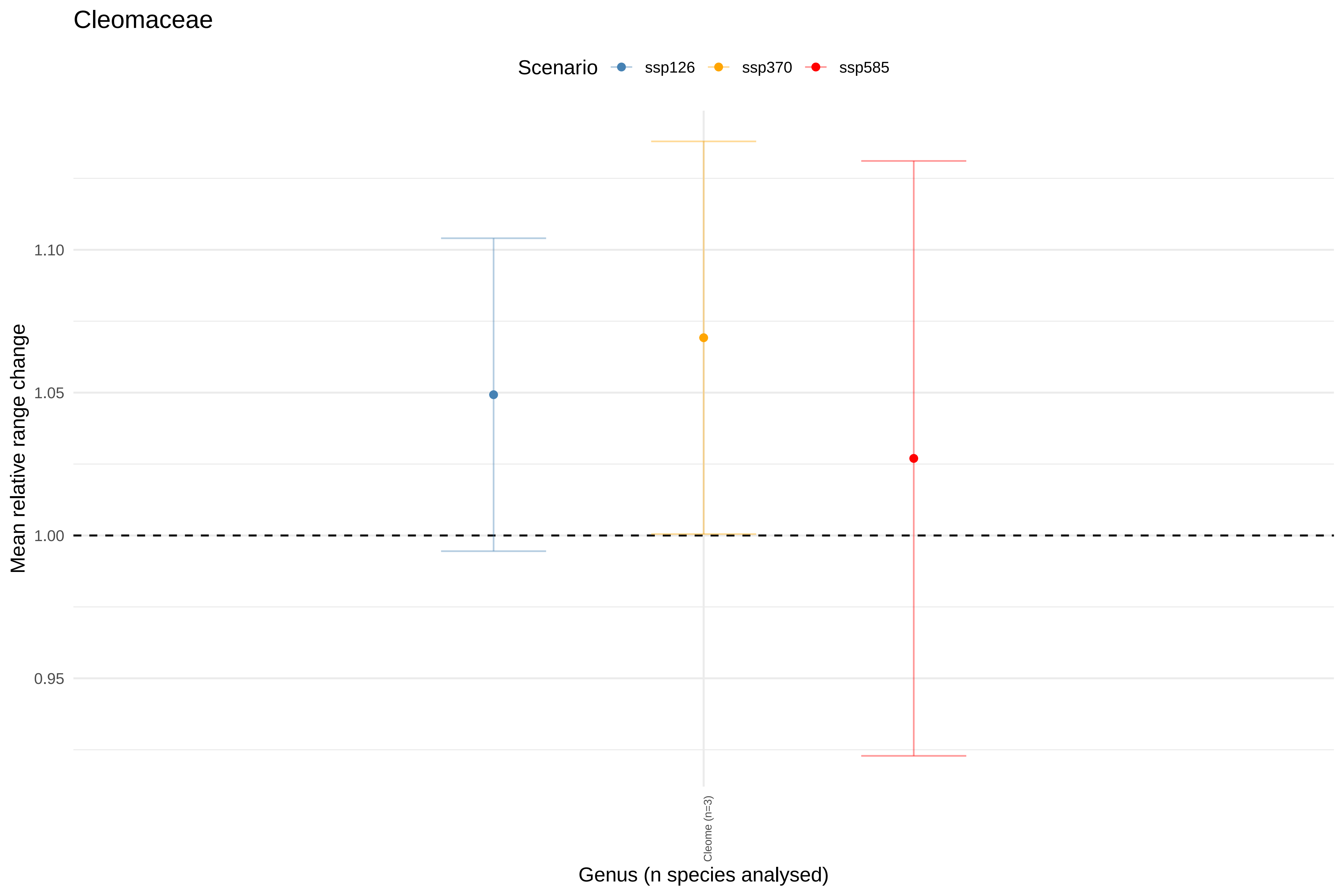

Cleomaceae
Scenario
ssp126
ssp370
ssp585
Mean relative range change
1.10
1.05
1.00
0.95
Cleome (n=3)
Genus (n species analysed)

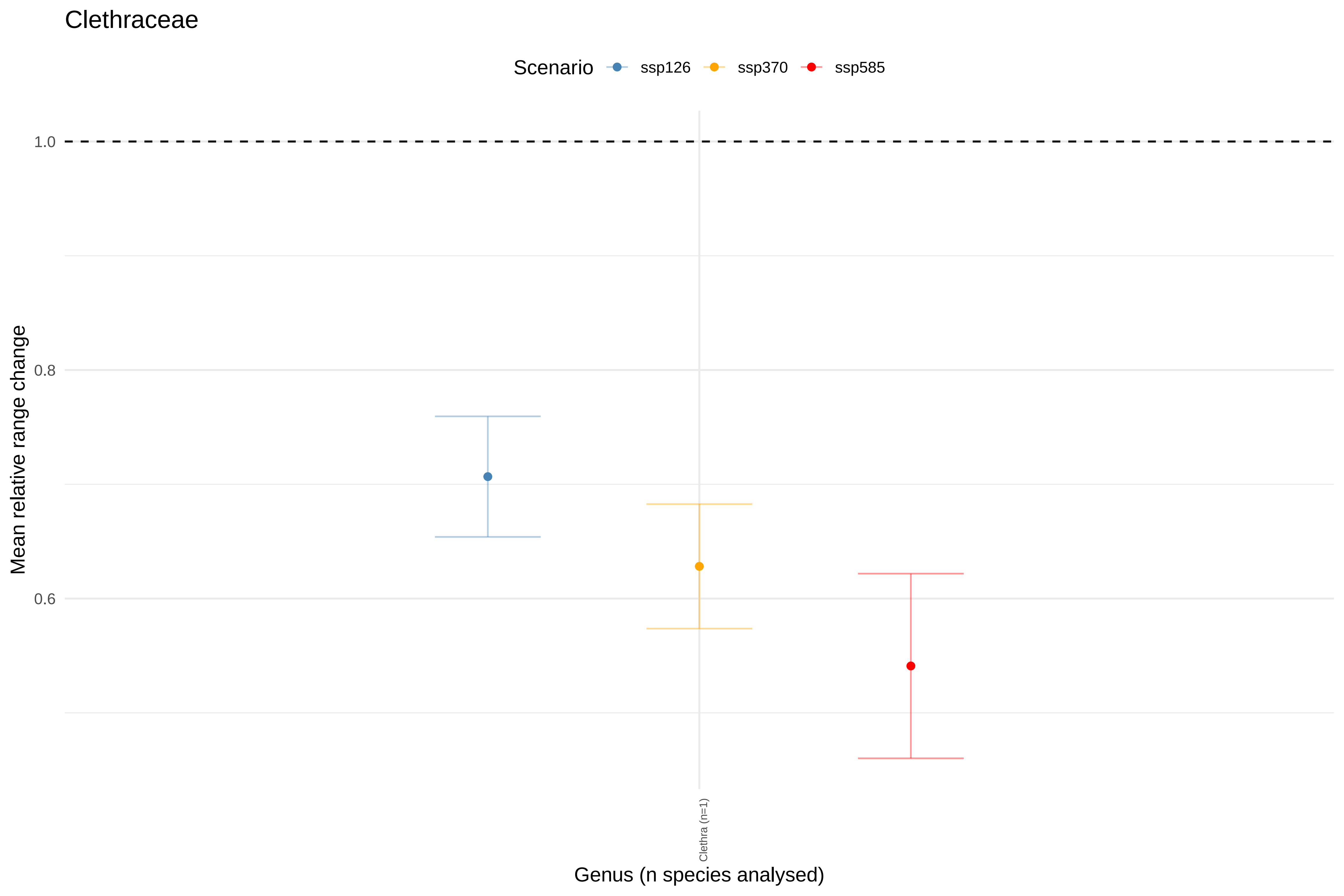

Clethraceae
Scenario
ssp126
ssp370
ssp585
1.0
0.8
0.6
Mean relative range change
Clethra (n=1)
Genus (n species analysed)

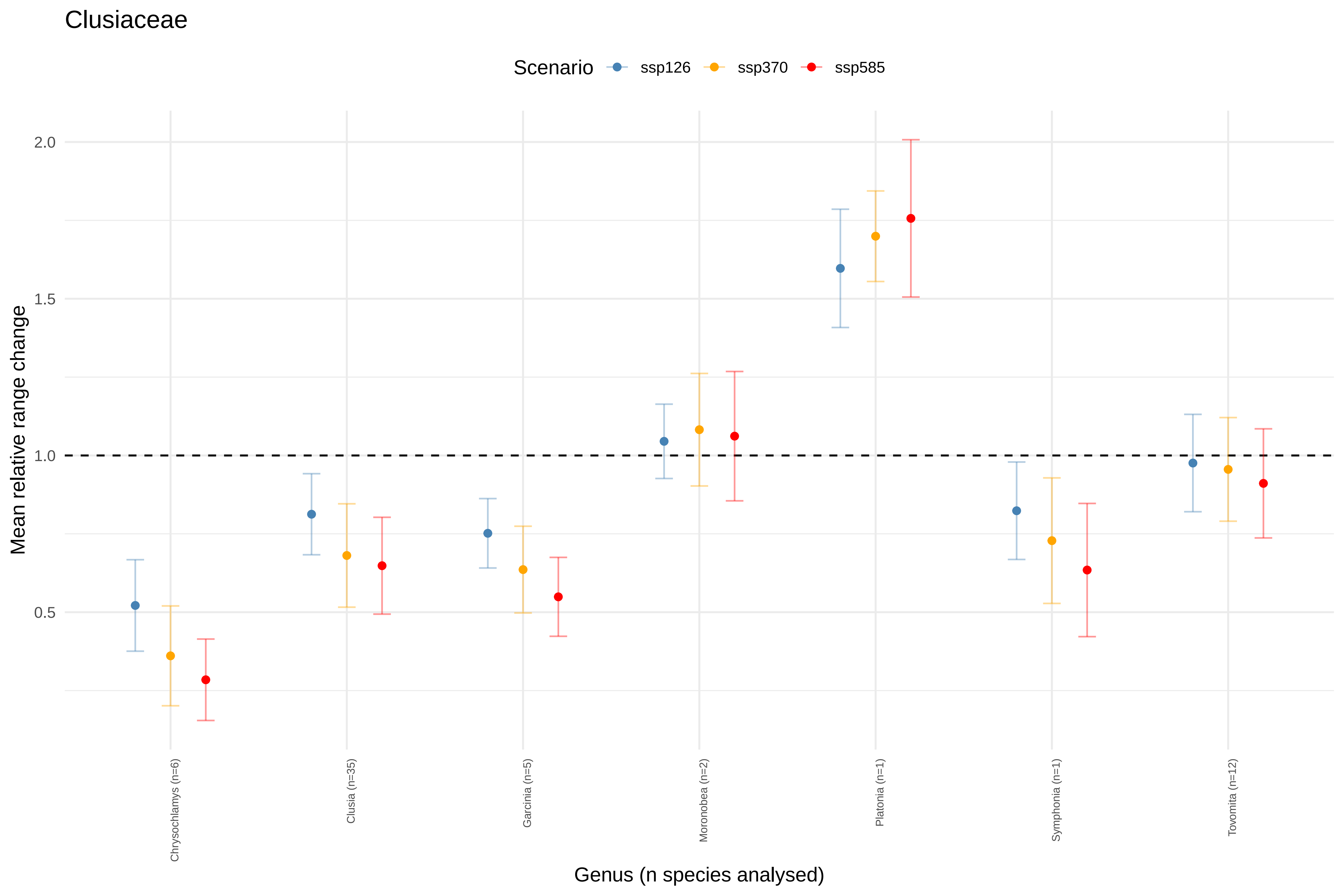
Clusiaceae
Scenario
ssp126
ssp370
ssp585
Mean relative range change
2.0
1.5
1.0
0.5
Chrysochlamys (n=6)
Clusia (n=35)
Garcinia (n=5)
Moronobea (n=2)
Platonia (n=1)
Symphonia (n=1)
Tovomita (n=12)
Genus (n species analysed)

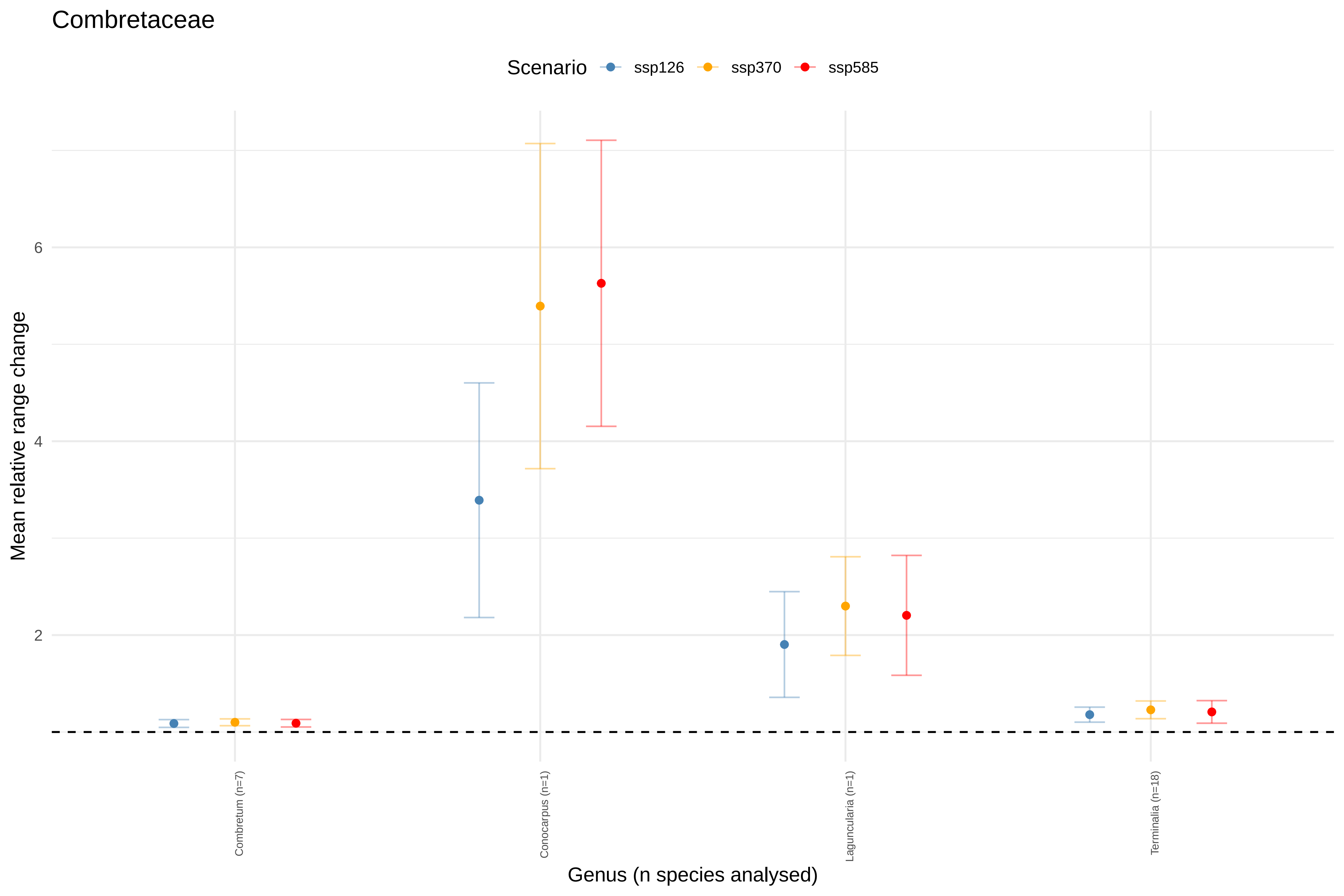

Combretaceae
Scenario
ssp126
ssp370
ssp585
Mean relative range change
6
4
2
Combretum (n=7)
Conocarpus (n=1)
Laguncularia (n=1)
Terminalia (n=18)
Genus (n species analysed)

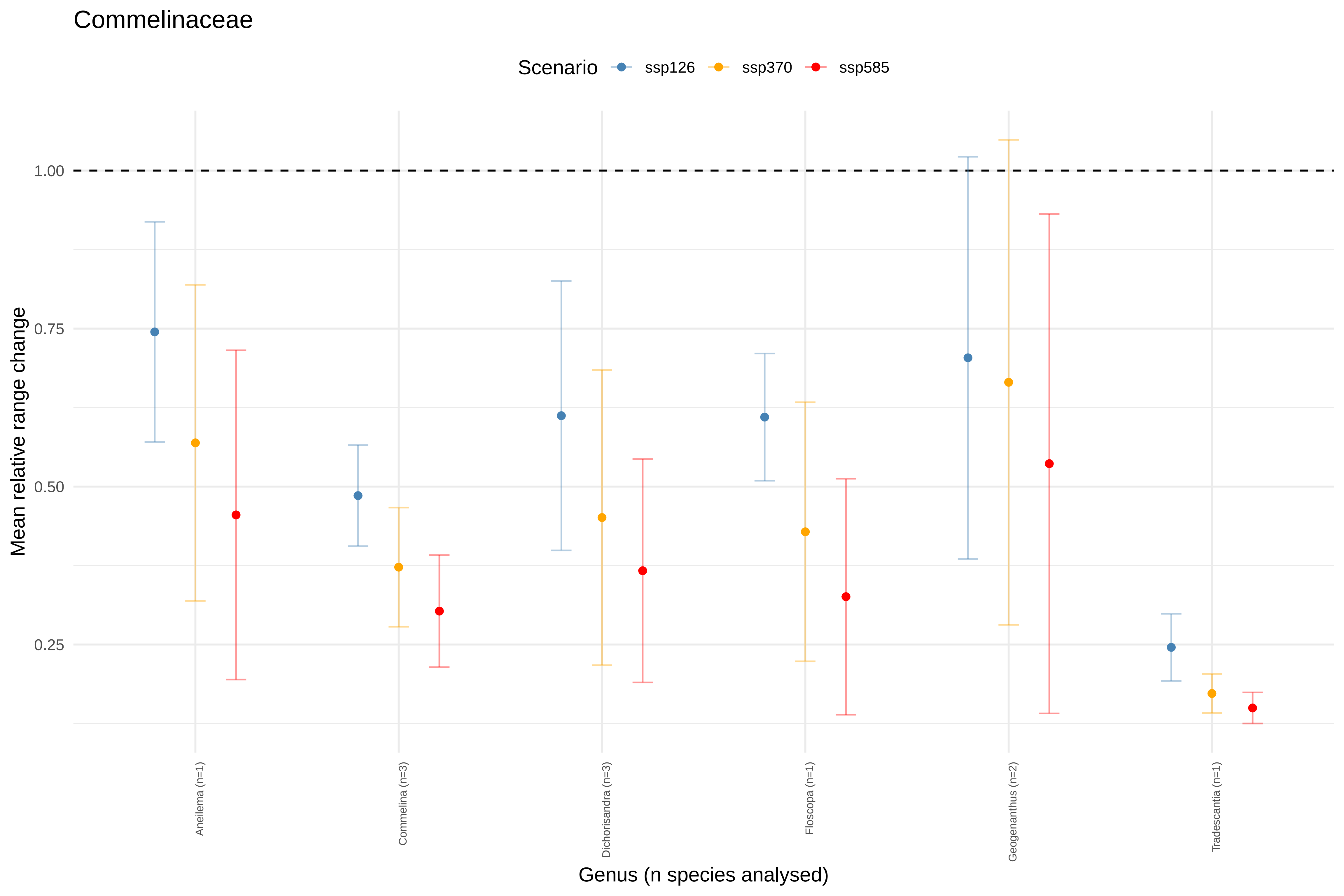
Commelinaceae
Scenario
ssp126
ssp370
ssp585
1.00
0.75
0.50
0.25
Mean relative range change
Aneilema (n=1)
Commelina (n=3)
Dichorisandra (n=3)
Floscopa (n=1)
Geogenanthus (n=2)
Tradescantia (n=1)
Genus (n species analysed)

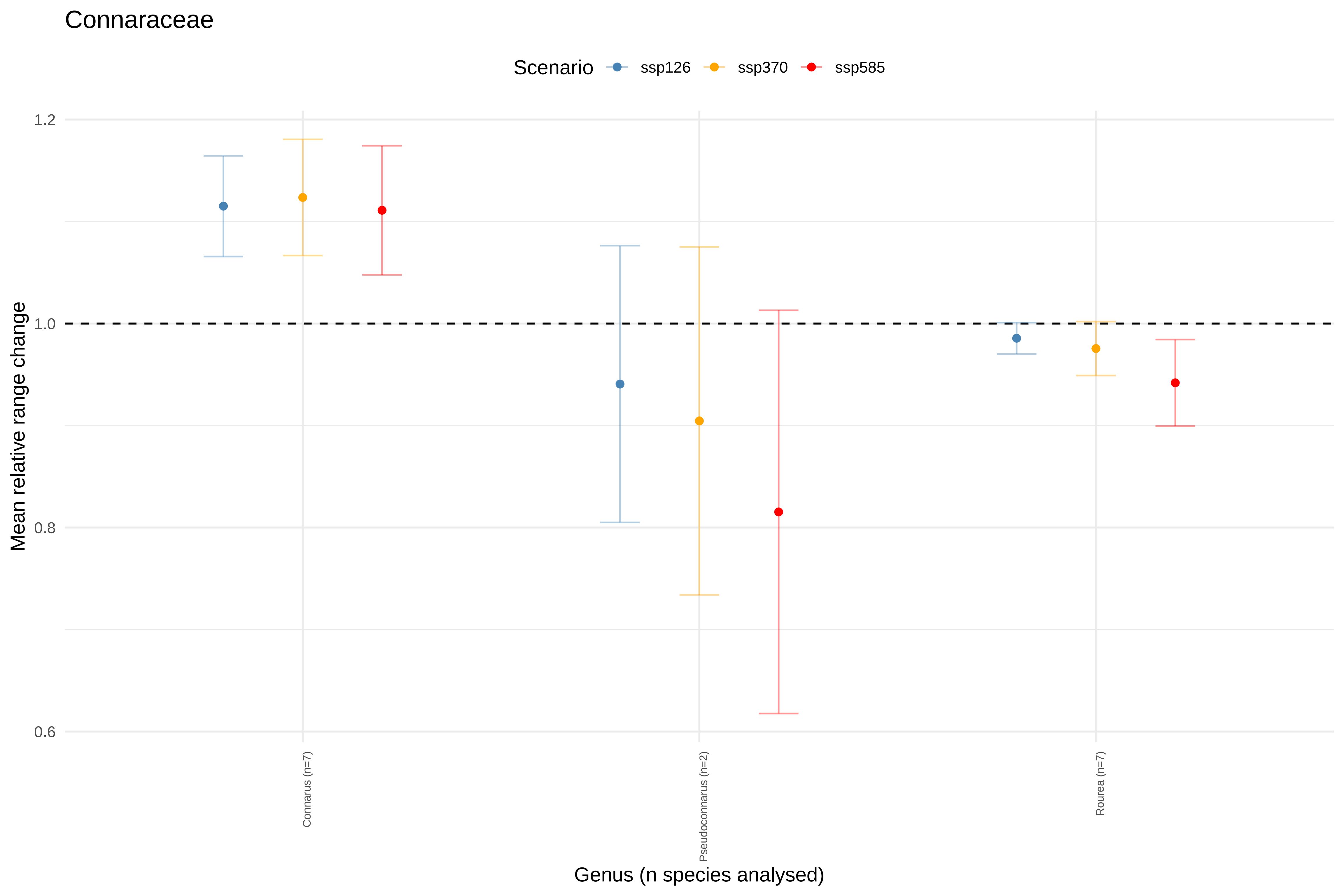

Connaraceae
Scenario
ssp126
ssp370
ssp585
1.2
1.0
0.8
0.6
Mean relative range change
Connarus (n=7)
Pseudoconnarus (n=2)
Rourea (n=7)
Genus (n species analysed)

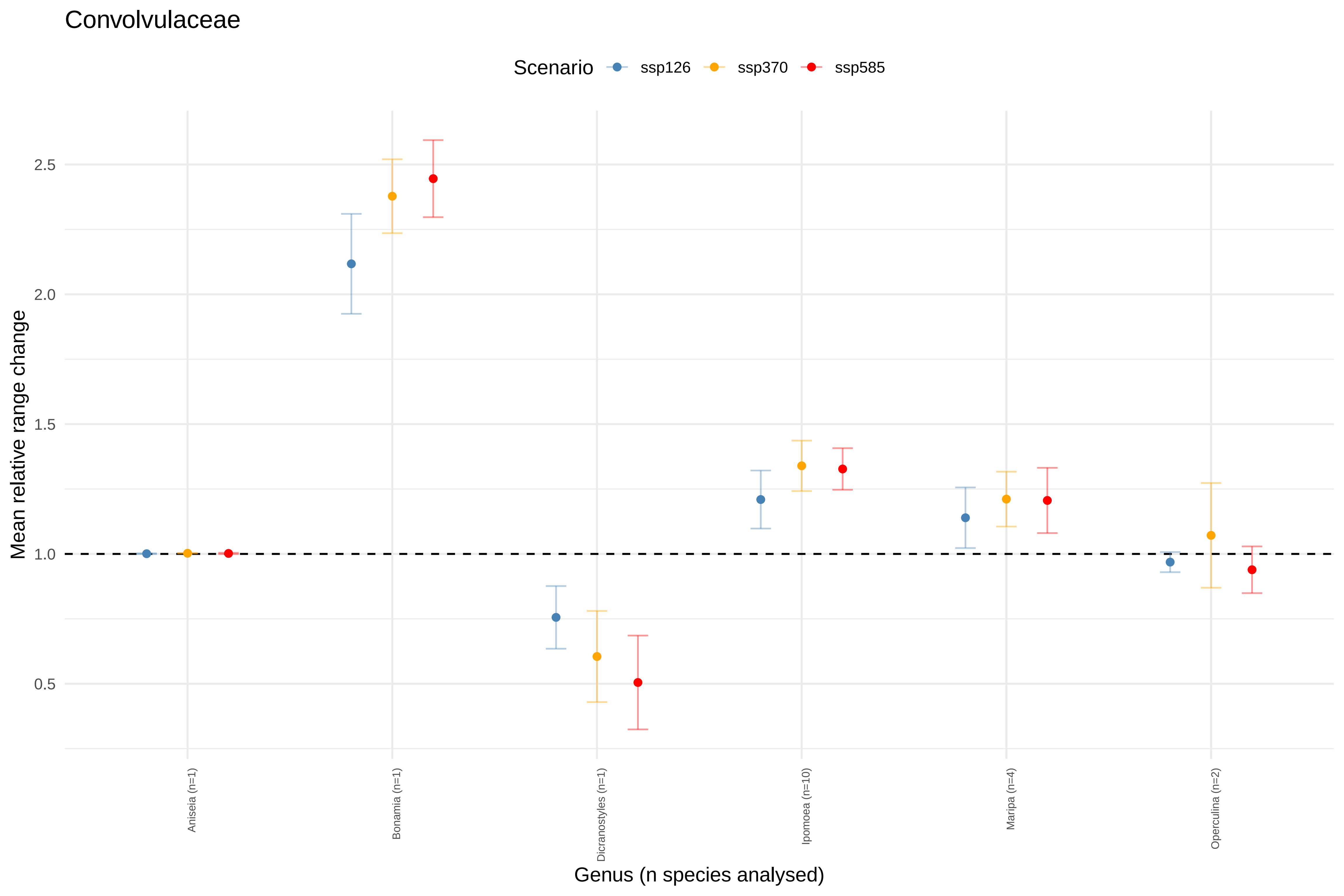

Convolvulaceae
Scenario
ssp126
ssp370
ssp585
Mean relative range change
2.5
2.0
1.5
1.0
0.5
Aniseia (n=1)
Bonamia (n=1)
Dicranostyles (n=1)
Ipomoea (n=10)
Maripa (n=4)
Operculina (n=2)
Genus (n species analysed)

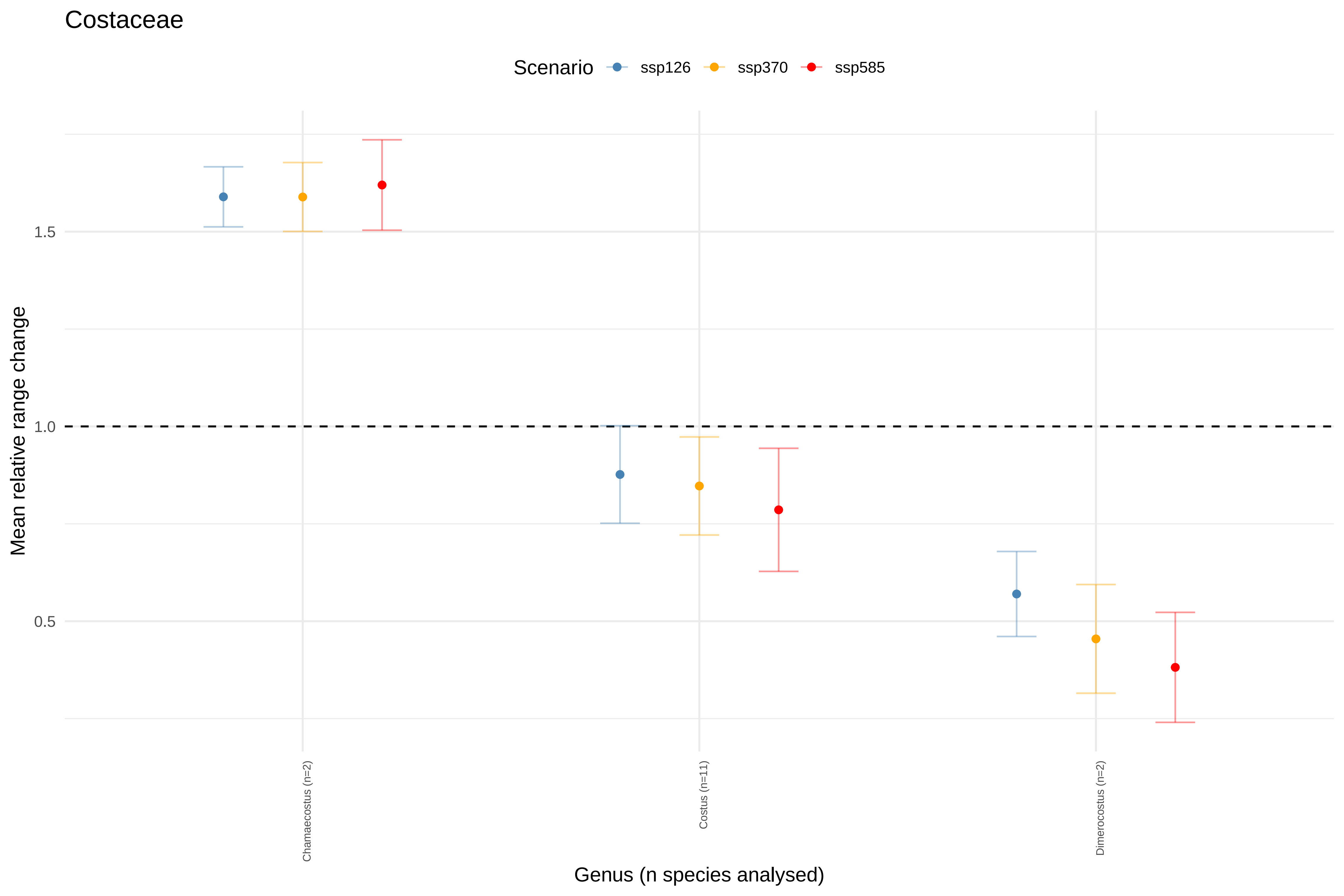

Costaceae
Scenario
ssp126
ssp370
ssp585
Mean relative range change
1.5
1.0
0.5
Chamaecostus (n=2)
Costus (n=11)
Dimerocostus (n=2)
Genus (n species analysed)

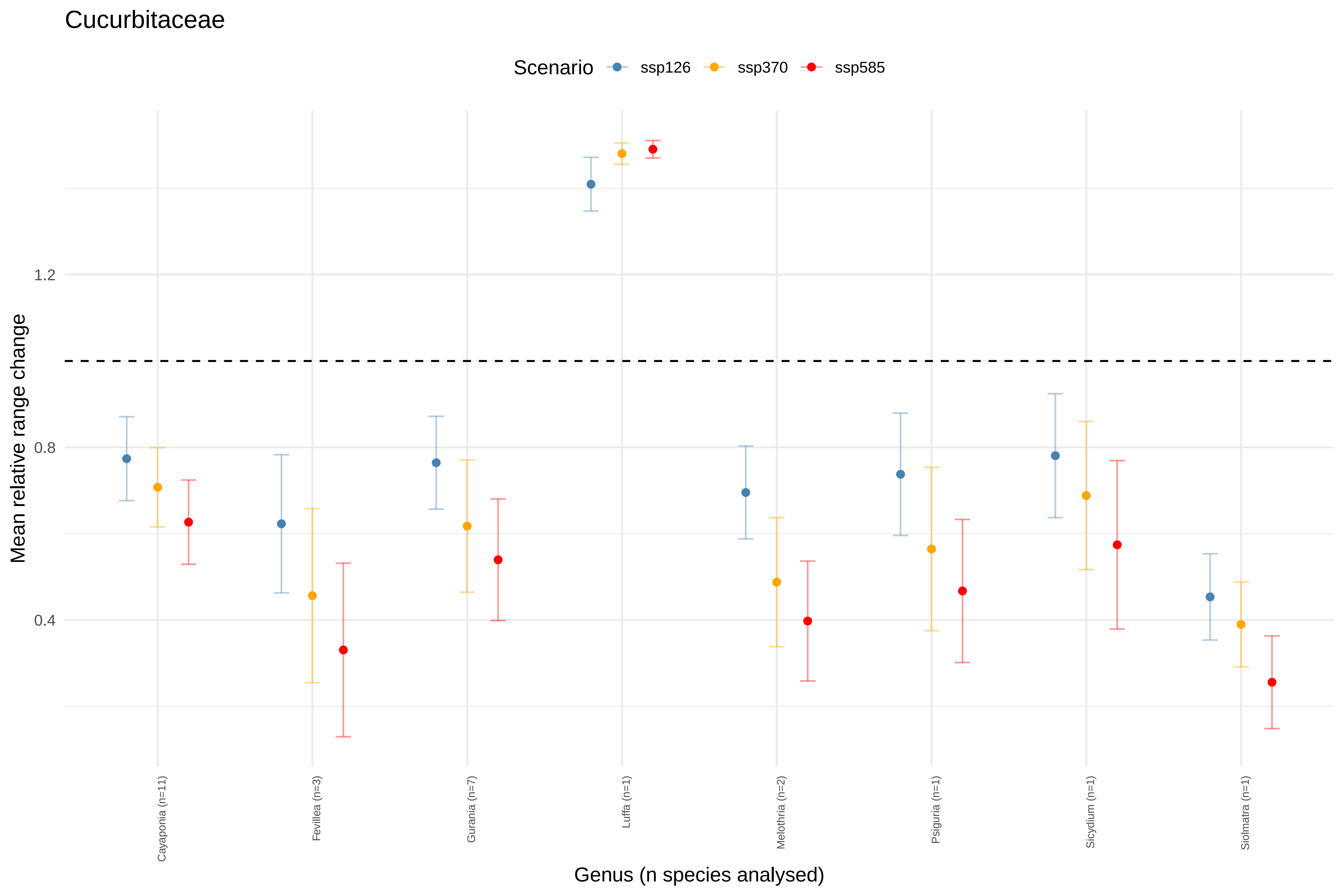

Cucurbitaceae
Scenario
ssp126
ssp370
ssp585
Mean relative range change
1.2
0.8
0.4
Cayaponia (n=11)
Fevillea (n=3)
Gurania (n=7)
Luffa (n=1)
Melothria (n=2)
Psiguria (n=1)
Sicydium (n=1)
Siolmatra (n=1)
Genus (n species analysed)

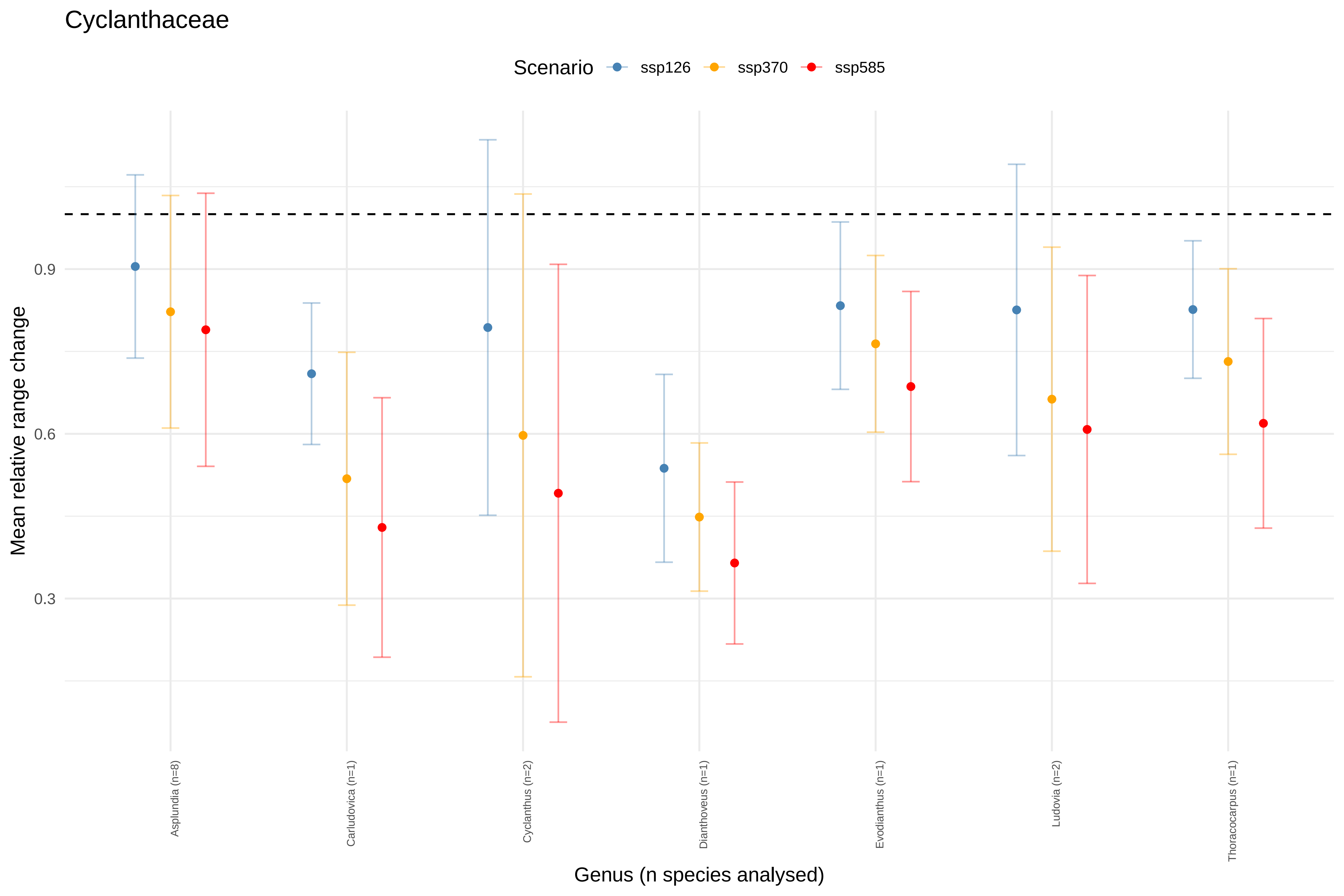

Cyclanthaceae
Scenario
ssp126
ssp370
ssp585
Mean relative range change
0.9
0.6
0.3
Asplundia (n=8)
Carludovica (n=1)
Cyclanthus (n=2)
Dianthoveus (n=1)
Evodianthus (n=1)
Ludovia (n=2)
Thoracocarpus (n=1)
Genus (n species analysed)

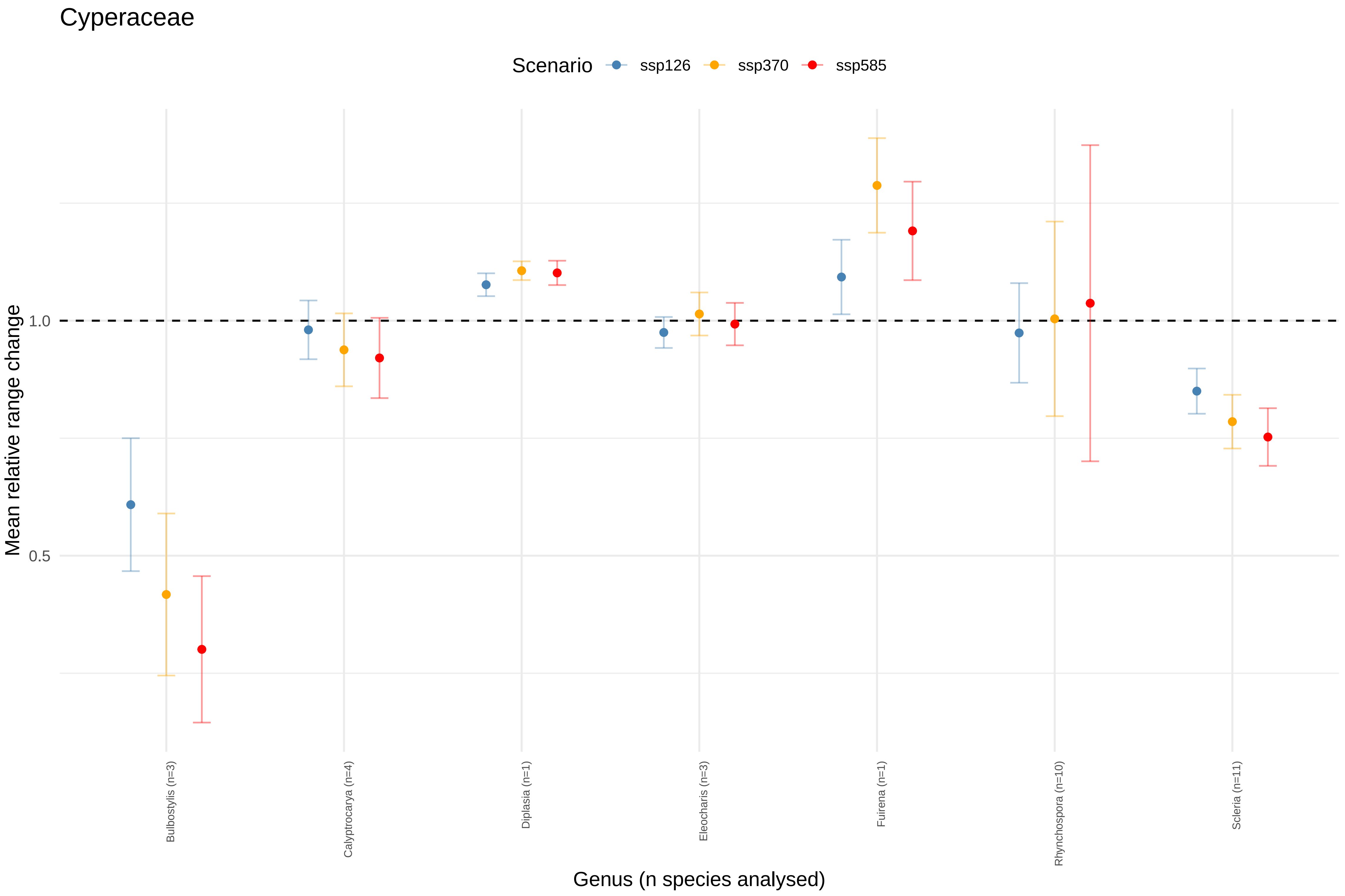

Cyperaceae
Scenario
ssp126
ssp370
ssp585
Mean relative range change
1.0
0.5
Bulbostylis (n=3)
Calyptrocarya (n=4)
Diplasia (n=1)
Eleocharis (n=3)
Fuirena (n=1)
Rhynchospora (n=10)
Scleria (n=11)
Genus (n species analysed)

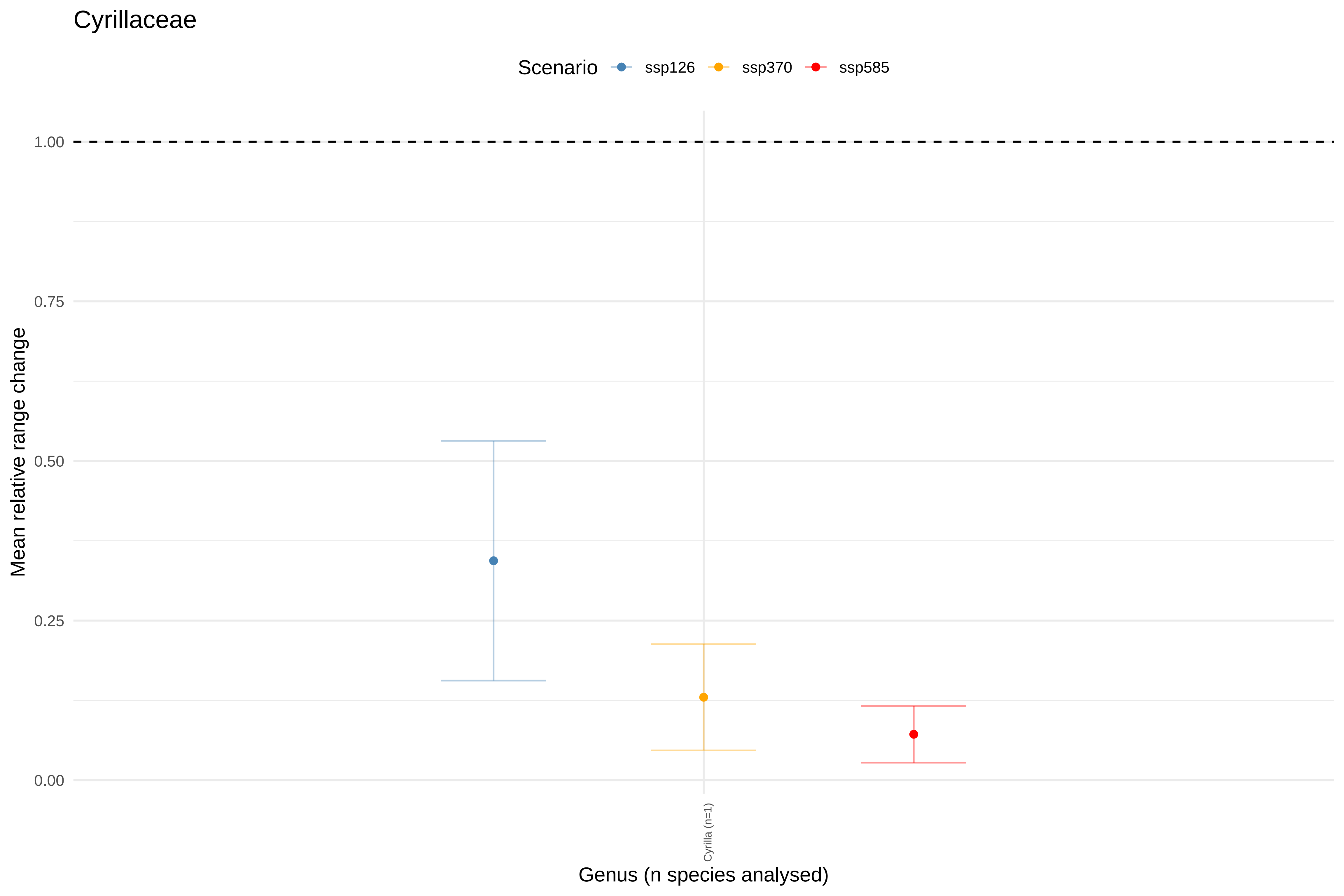
Cyrillaceae
Scenario
ssp126
ssp370
ssp585
1.00
0.75
0.50
0.25
0.00
Mean relative range change
Cyrilla (n=1)
Genus (n species analysed)

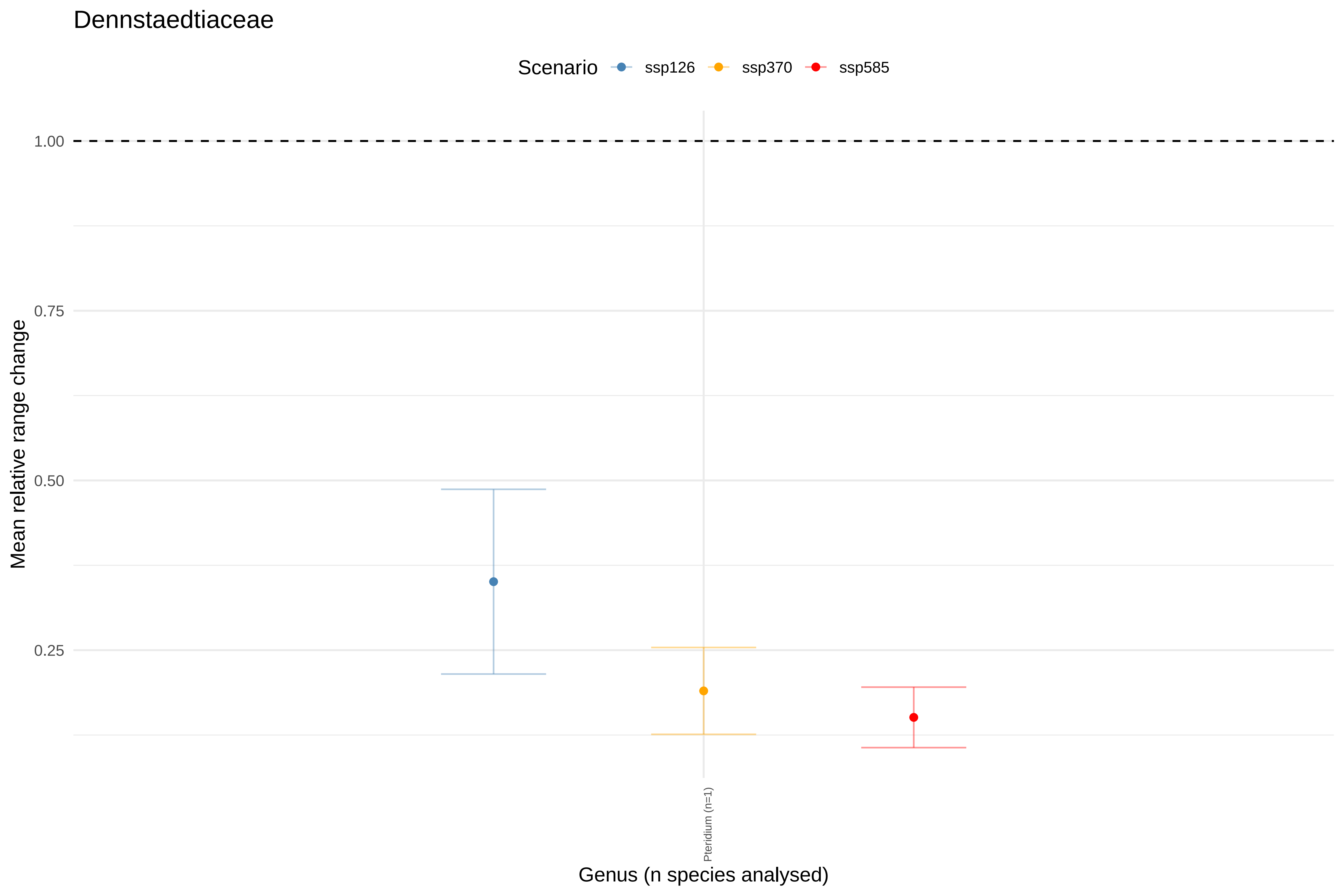

Dennstaedtiaceae
Scenario
ssp126
ssp370
ssp585
1.00
0.75
0.50
0.25
Mean relative range change
Pteridium (n=1)
Genus (n species analysed)

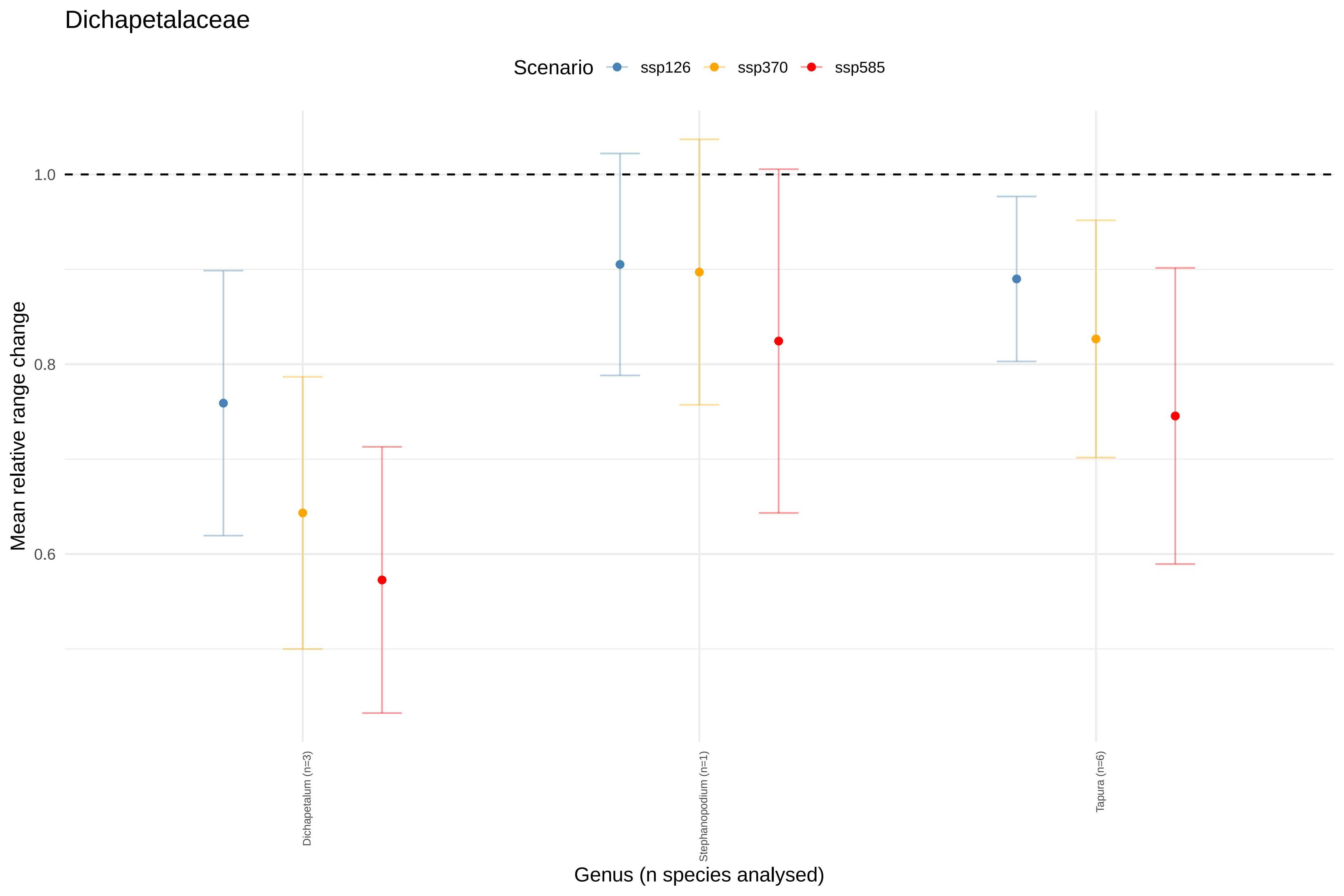

Dichapetalaceae
Scenario
ssp126
ssp370
ssp585
Mean relative range change
1.0
0.8
0.6
Dichapetalum (n=3)
Stephanopodium (n=1)
Tapura (n=6)
Genus (n species analysed)

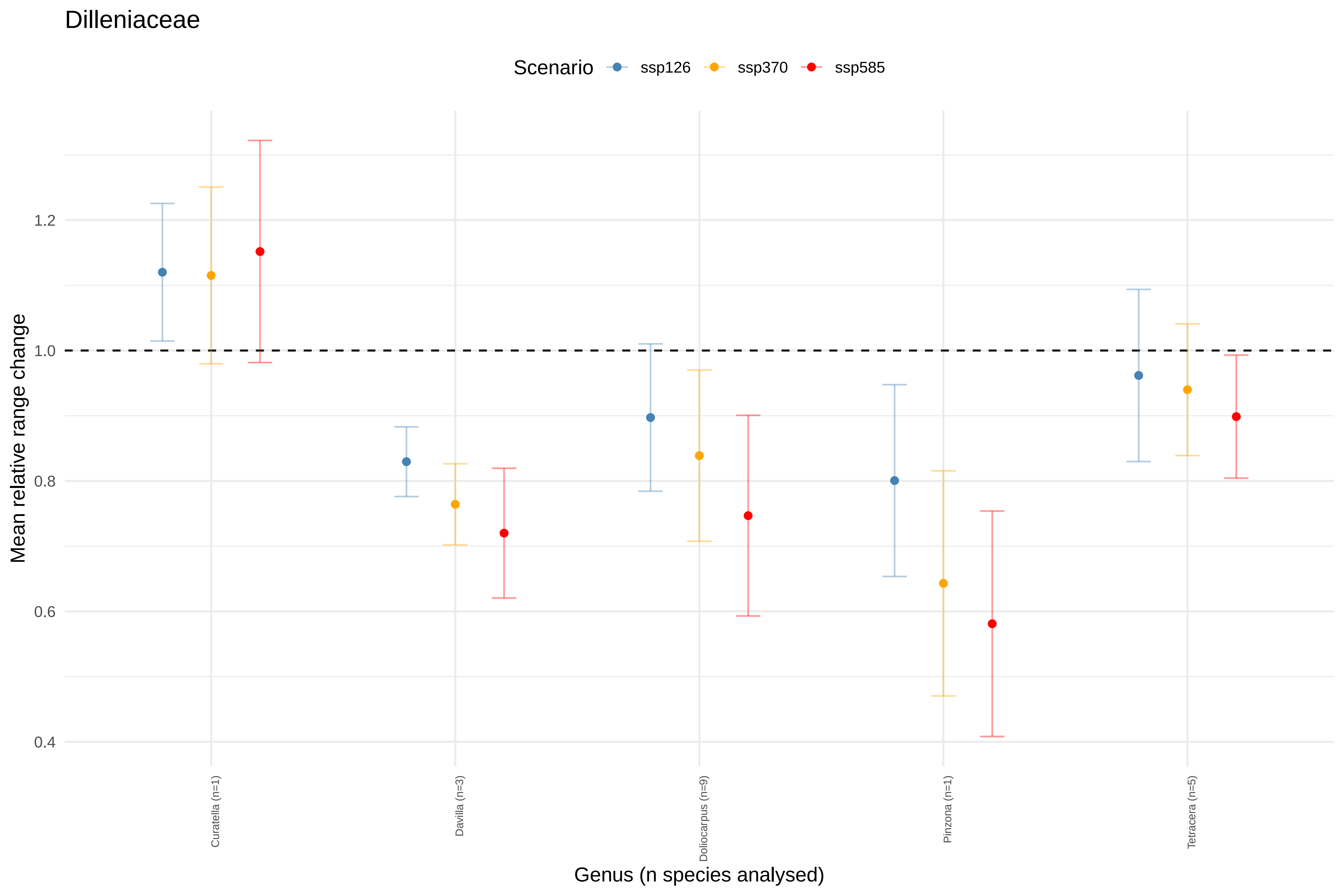

Dilleniaceae
Scenario
ssp126
ssp370
ssp585
Mean relative range change
1.2
1.0
0.8
0.6
0.4
Curatella (n=1)
Davilla (n=3)
Doliocarpus (n=9)
Pinzona (n=1)
Tetracera (n=5)
Genus (n species analysed)

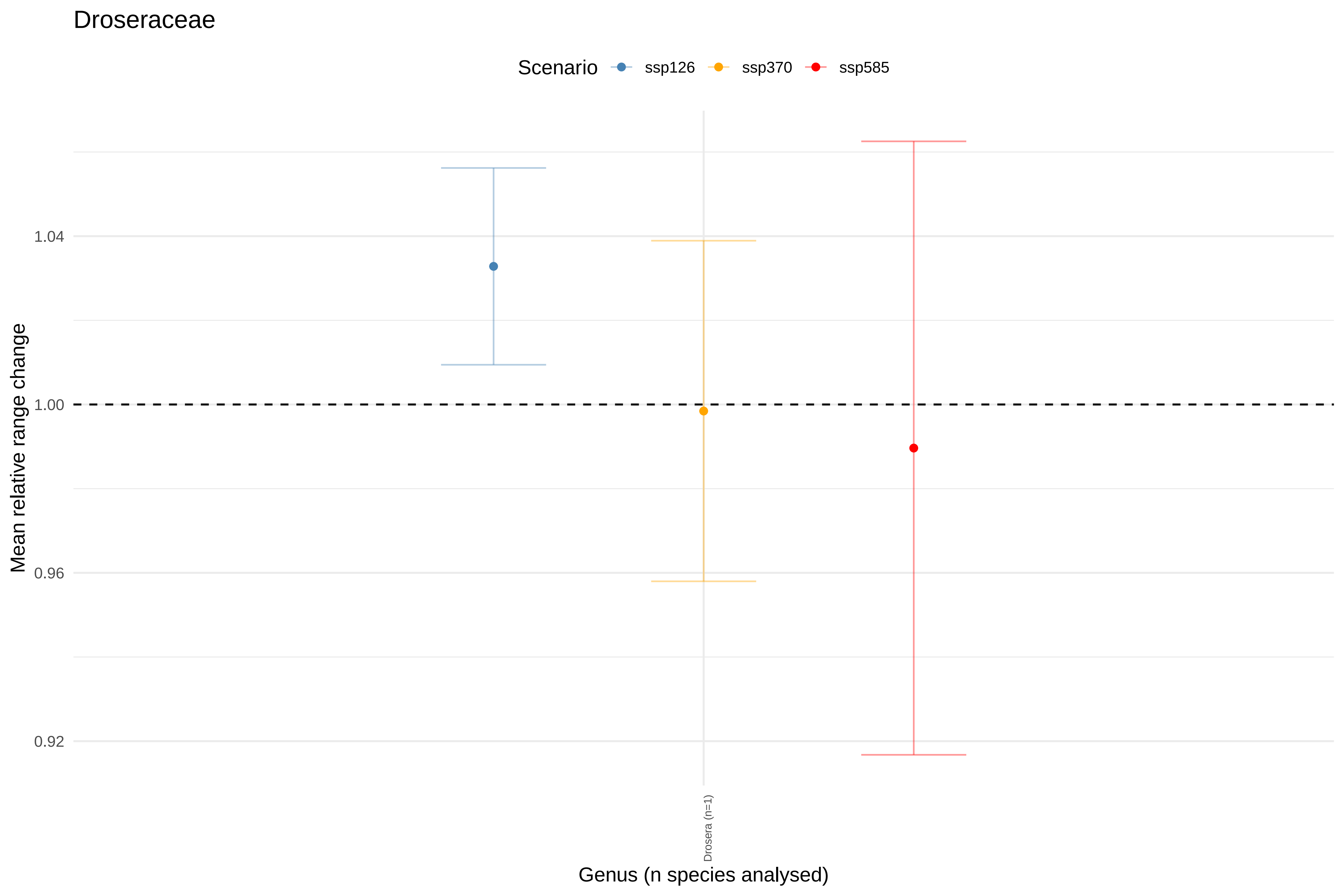
Droseraceae
Scenario
ssp126
ssp370
ssp585
Mean relative range change
1.04
1.00
0.96
0.92
Drosera (n=1)
Genus (n species analysed)

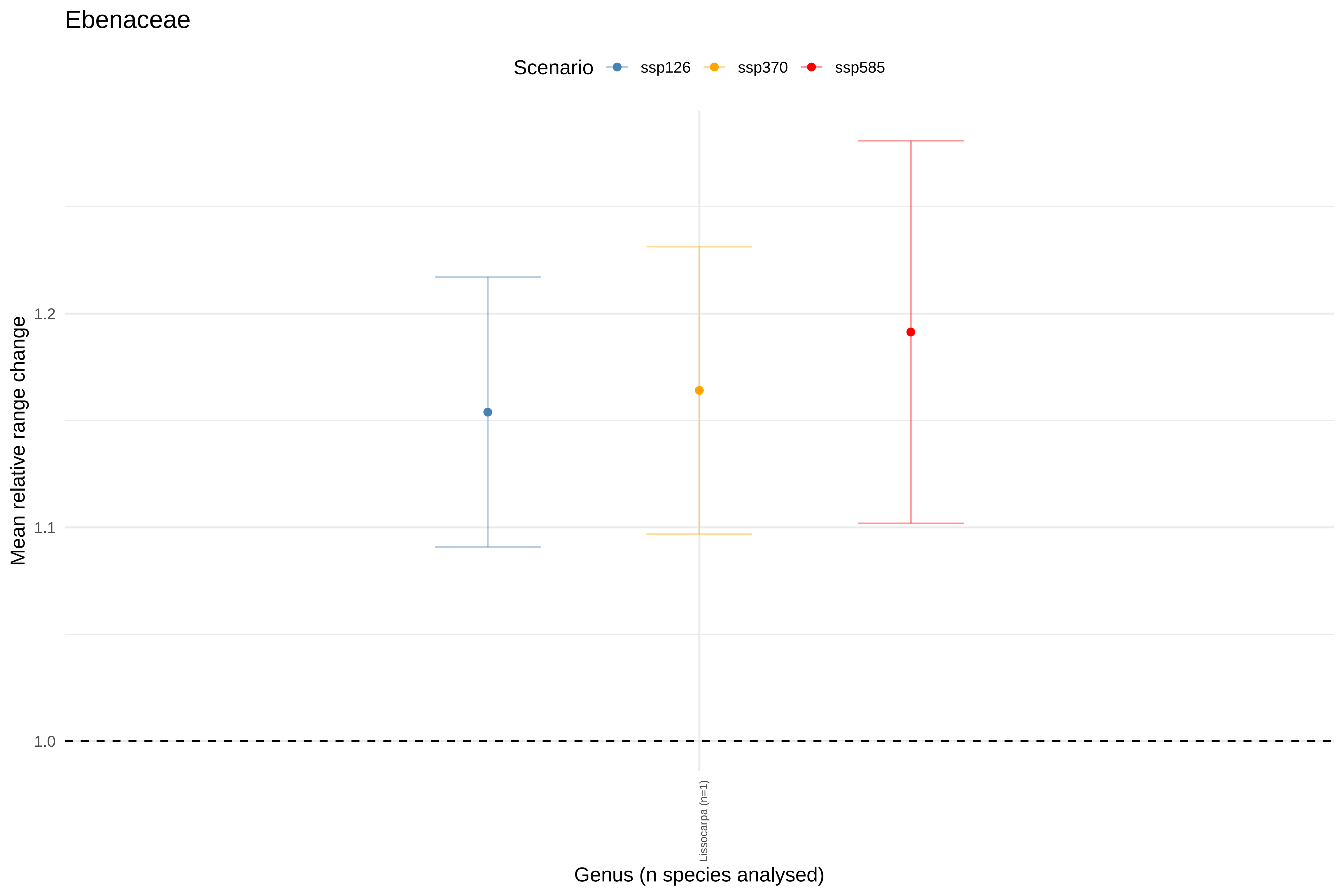

Ebenaceae
Scenario
ssp126
ssp370
ssp585
Mean relative range change
1.2
1.1
1.0
Lissocarpa (n=1)
Genus (n species analysed)

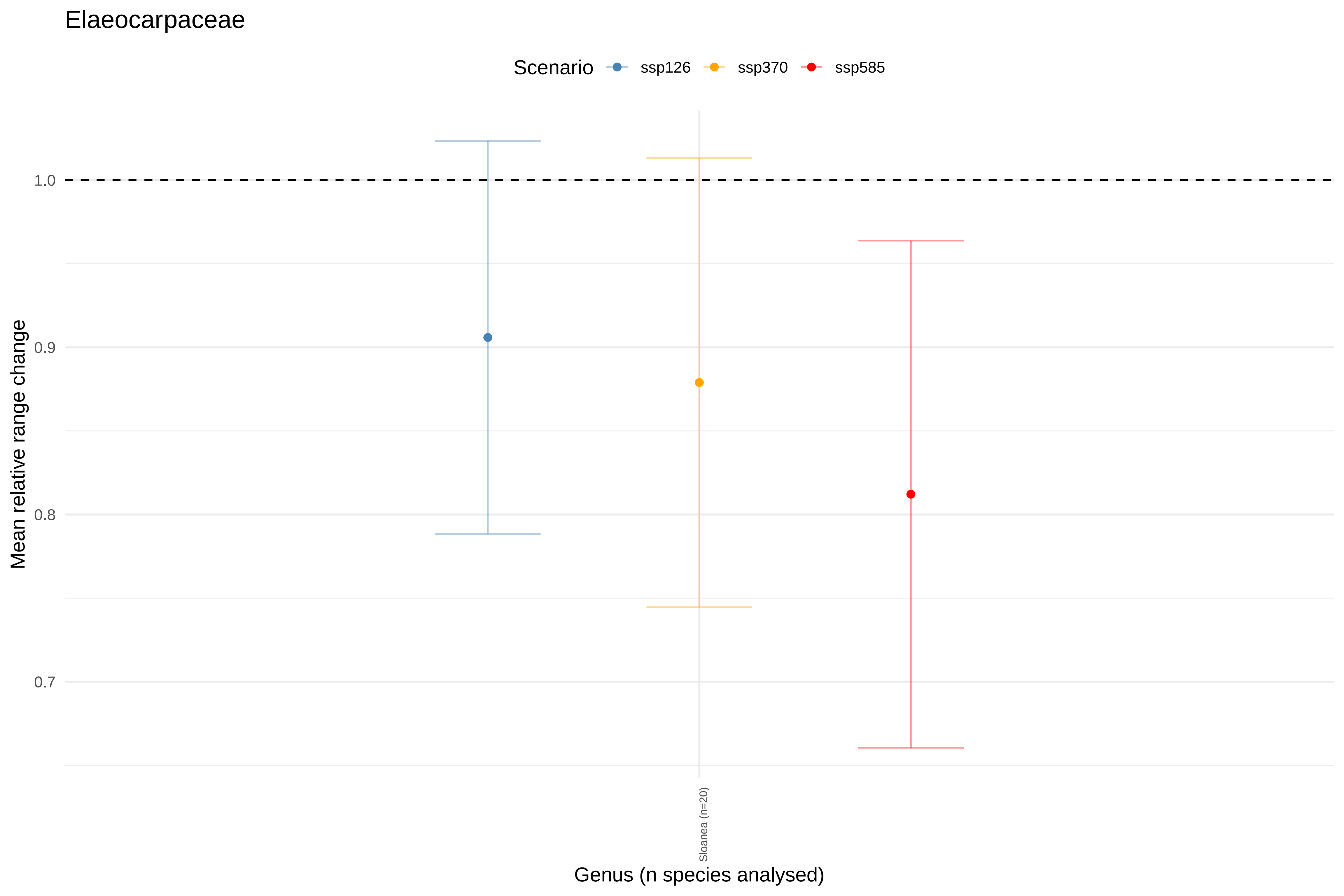

Elaeocarpaceae
Scenario
ssp126
ssp370
ssp585
1.0
0.9
0.8
0.7
Mean relative range change
Sloanea (n=20)
Genus (n species analysed)

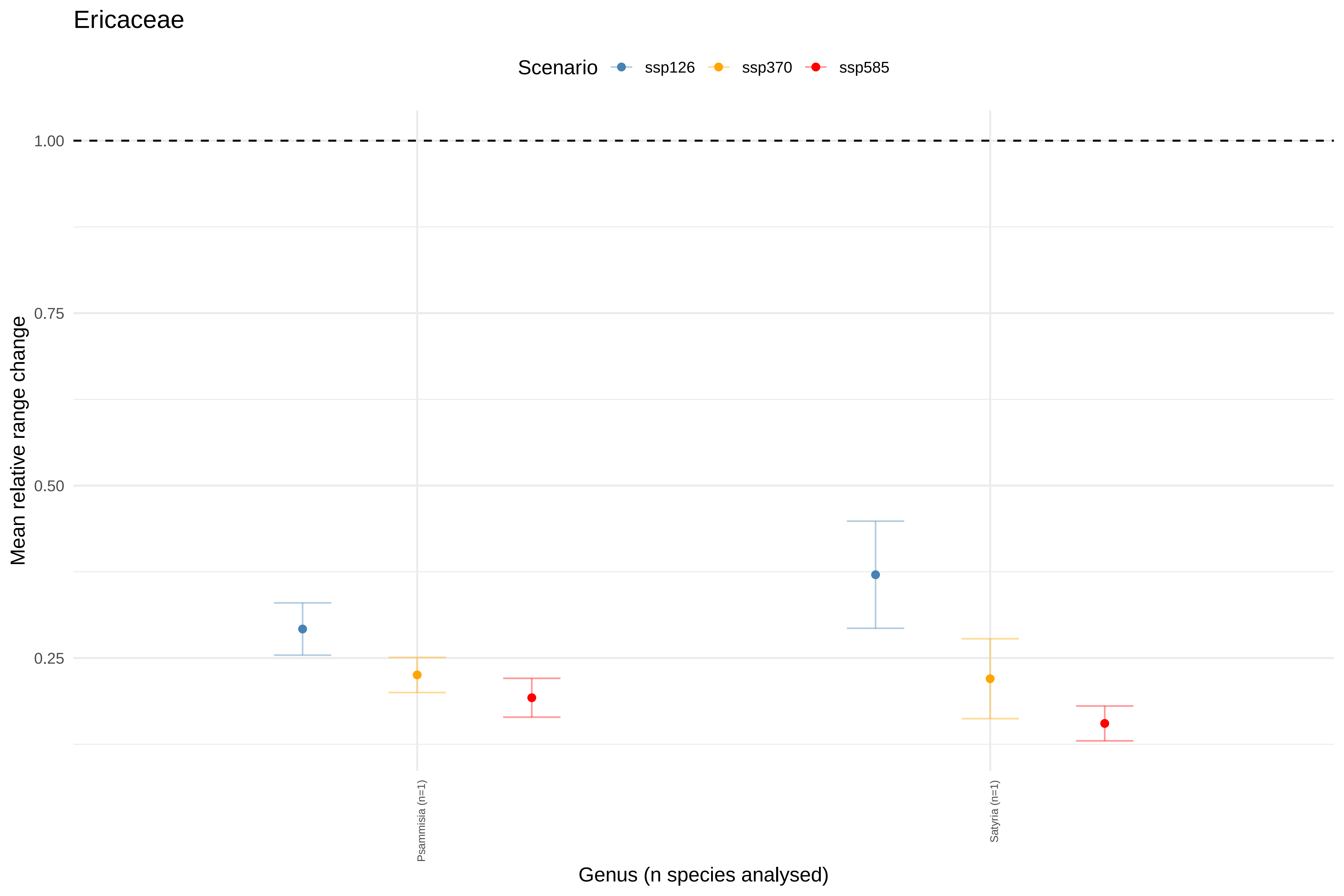

Ericaceae
Scenario
ssp126
ssp370
ssp585
1.00
0.75
0.50
0.25
Mean relative range change
Psammisia (n=1)
Satyria (n=1)
Genus (n species analysed)

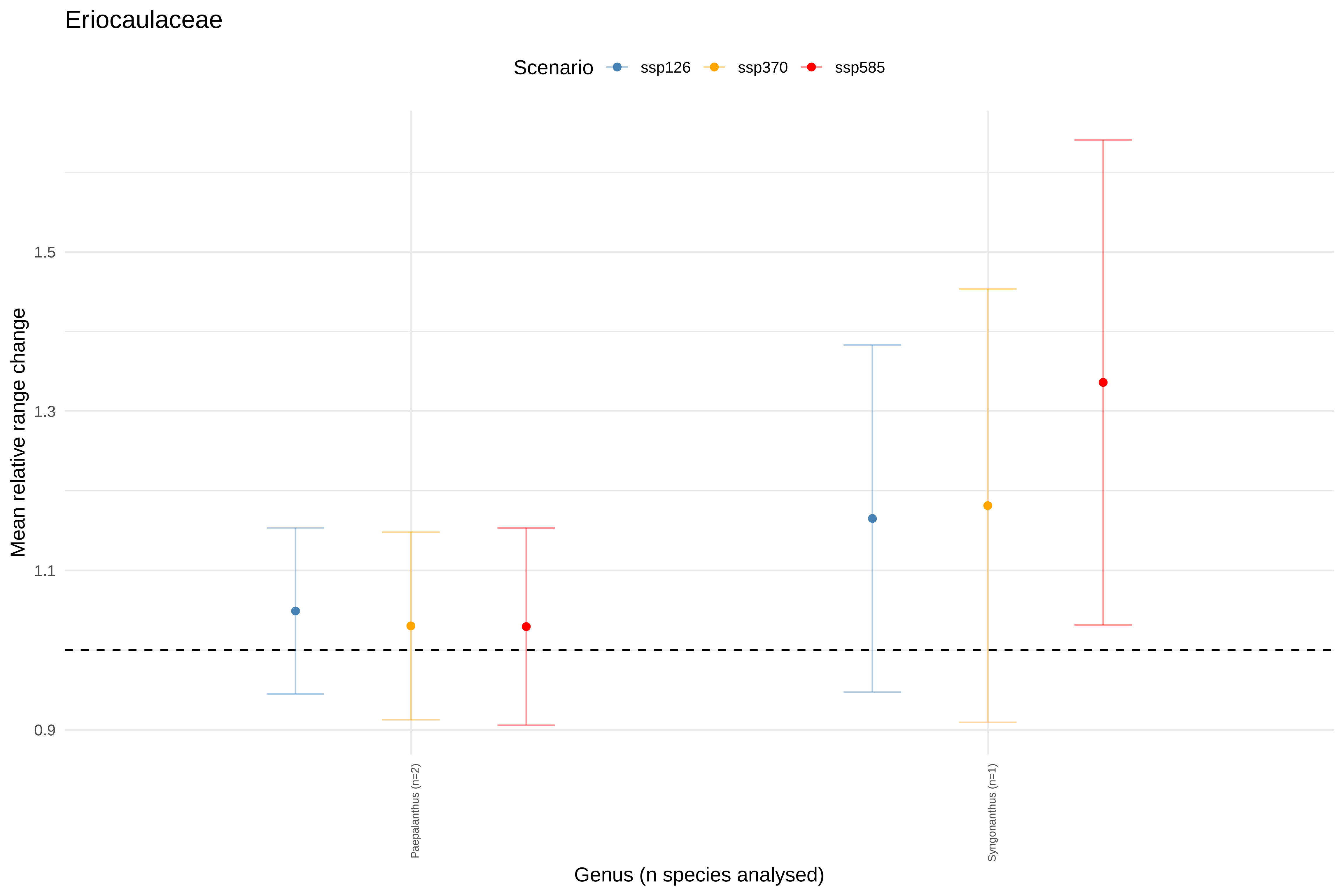

Eriocaulaceae
Scenario
ssp126
ssp370
ssp585
Mean relative range change
1.5
1.3
1.1
0.9
Paepalanthus (n=2)
Syngonanthus (n=1)
Genus (n species analysed)

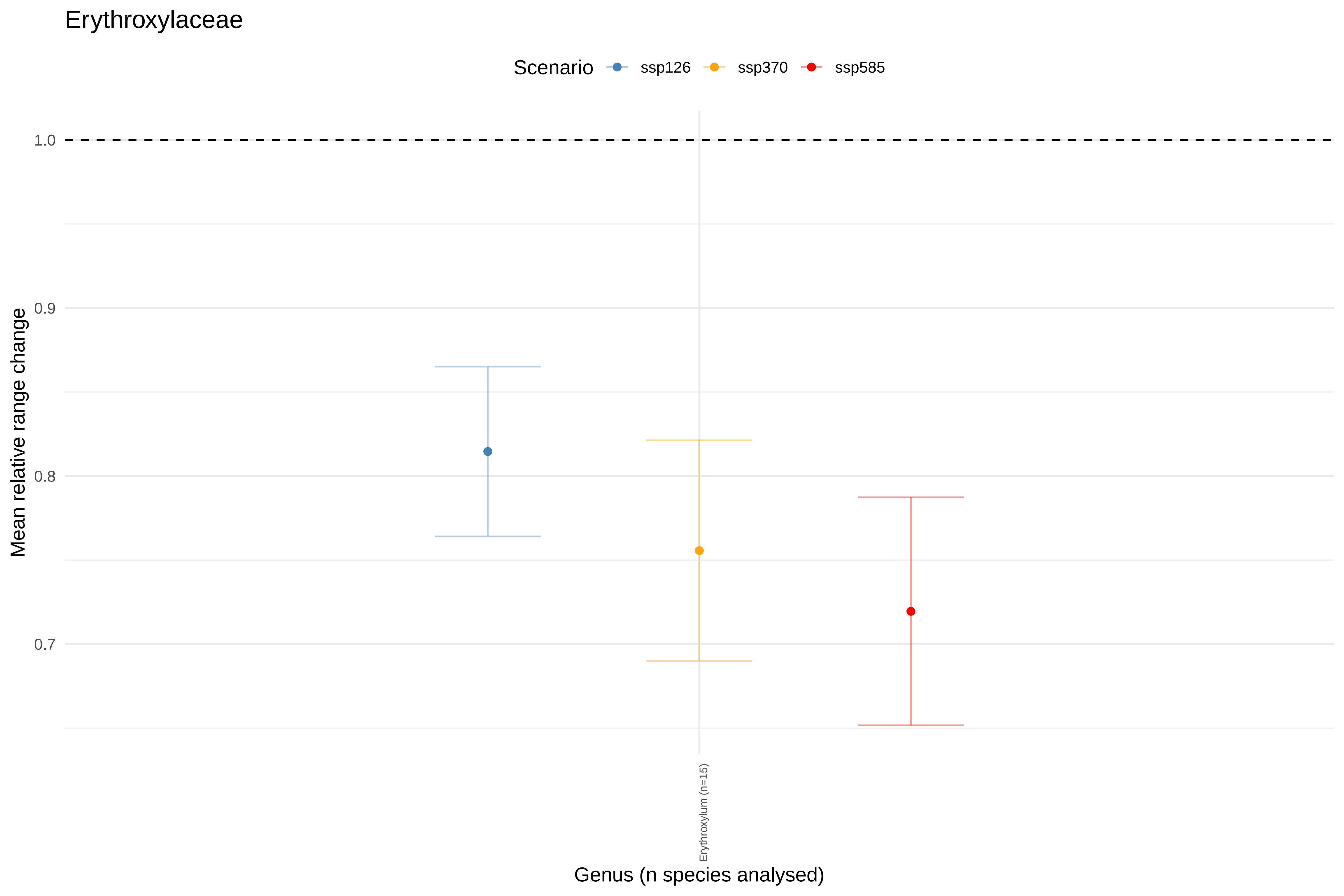

Erythroxylaceae
Scenario
ssp126
ssp370
ssp585
1.0
0.9
0.8
0.7
Mean relative range change
Erythroxylum (n=15)
Genus (n species analysed)

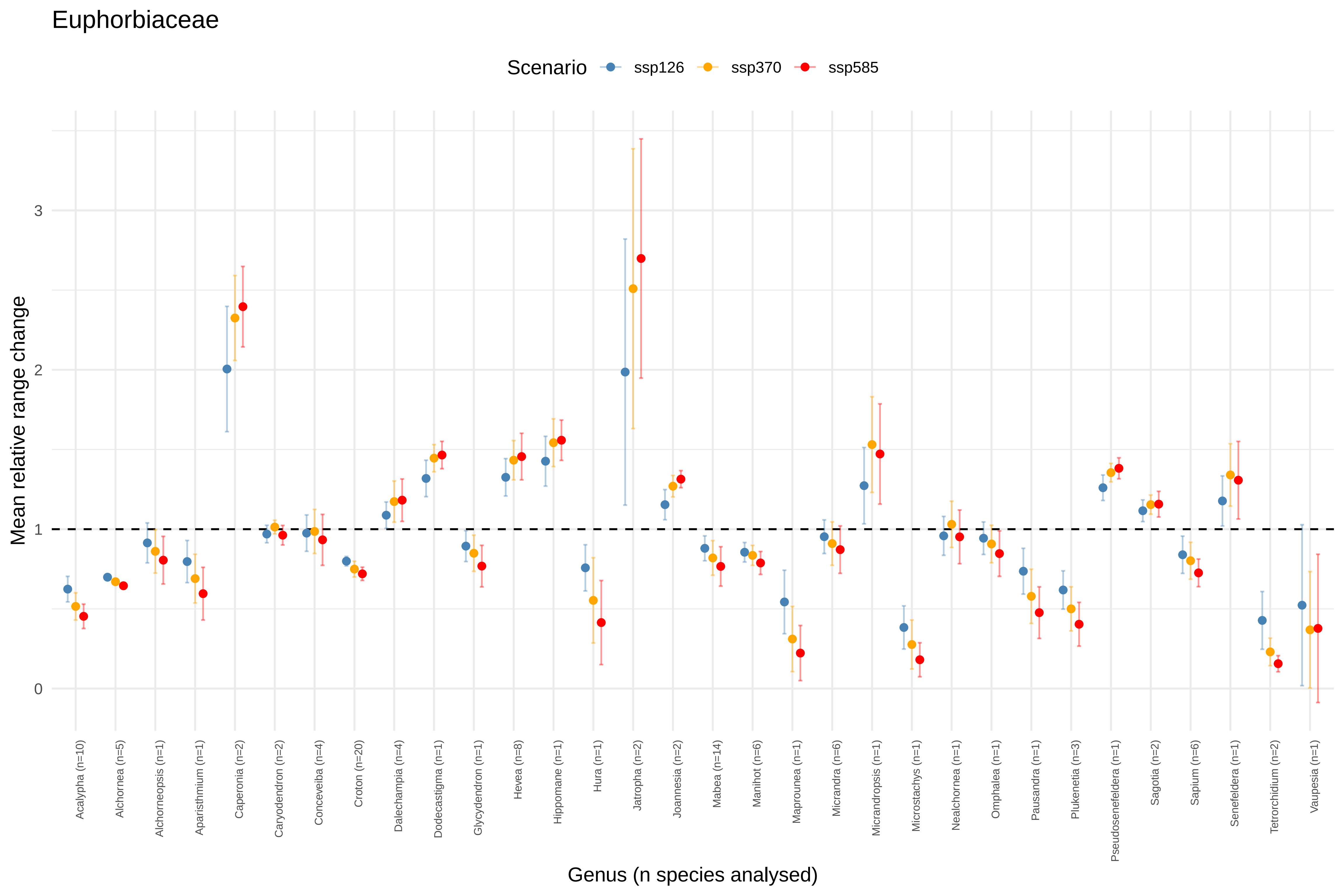

Euphorbiaceae
Scenario
ssp126
ssp370
ssp585
Mean relative range change
0
1
2
3
Acalypha (n=10)
Alchornea (n=5)
Alchorneopsis (n=1)
Aparisthmium (n=1)
Caperonia (n=2)
Caryodendron (n=2)
Conceveiba (n=4)
Croton (n=20)
Dalechampia (n=4)
Dodecastigma (n=1)
Glycydendron (n=1)
Hevea (n=8)
Hippomane (n=1)
Hura (n=1)
Jatropha (n=2)
Joannesia (n=2)
Mabea (n=14)
Manihot (n=6)
Maprounea (n=1)
Micrandra (n=6)
Micrandropsis (n=1)
Microstachys (n=1)
Nealchornea (n=1)
Omphalea (n=1)
Pausandra (n=1)
Plukenetia (n=3)
Pseudosenefeldera (n=1)
Sagotia (n=2)
Sapium (n=6)
Senefeldera (n=1)
Tetrorchidium (n=2)
Vaupesia (n=1)
Genus (n species analysed)

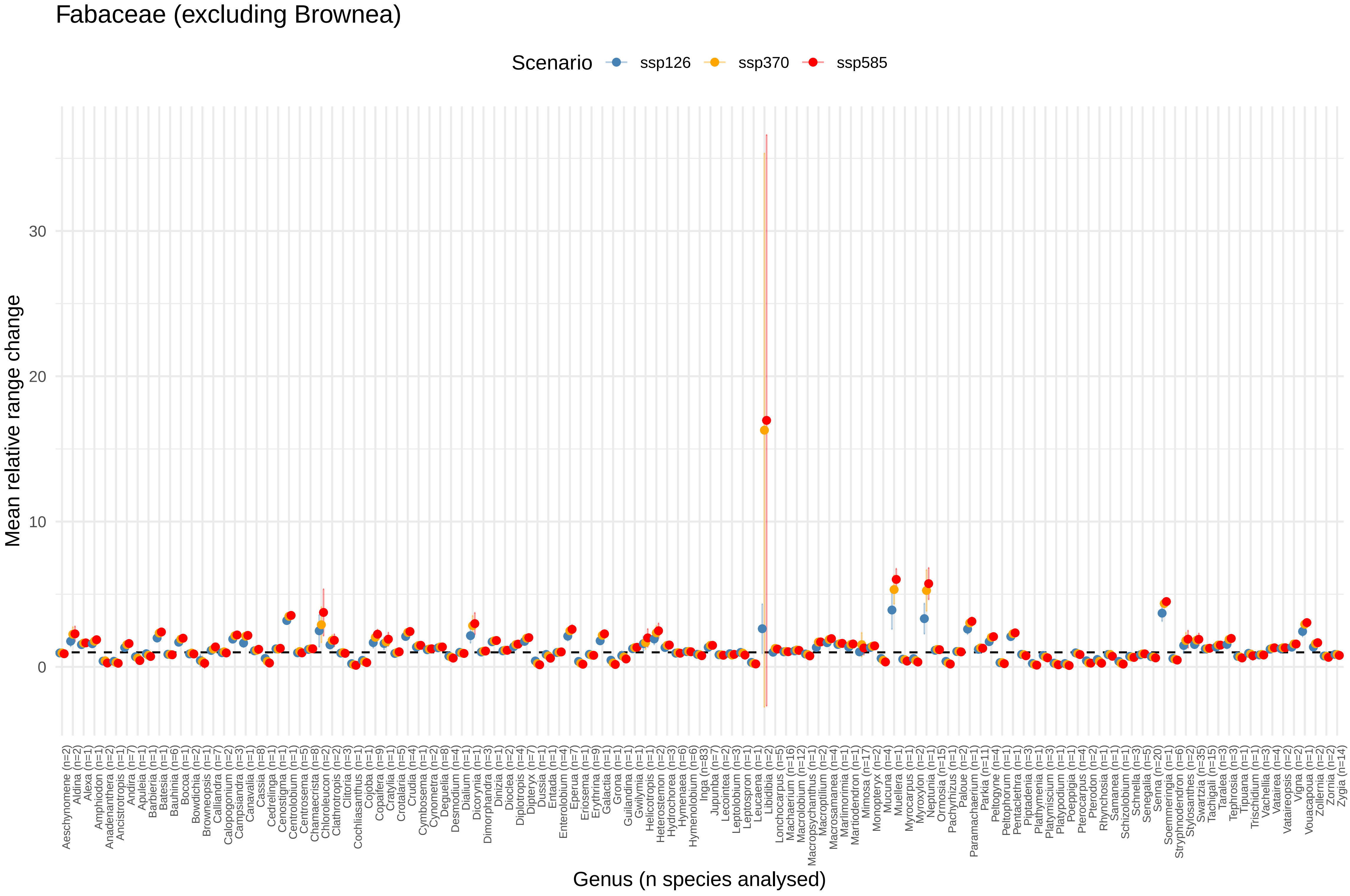

Fabaceae (excluding Brownea)
Scenario
ssp126
ssp370
ssp585
Mean relative range change
0
10
20
30
Aeschynomene (n=2)
Aldina (n=2)
Alexa (n=1)
Amphiodon (n=1)
Anadenanthera (n=2)
Ancistrotropis (n=1)
Andira (n=7)
Apuleia (n=1)
Barbieria (n=1)
Batesia (n=1)
Bauhinia (n=6)
Bocoa (n=1)
Bowdichia (n=2)
Browneopsis (n=1)
Calliandra (n=7)
Calopogonium (n=2)
Campsiandra (n=3)
Canavalia (n=1)
Cassia (n=8)
Cedrelinga (n=1)
Cenostigma (n=1)
Centrolobium (n=1)
Centrosema (n=5)
Chamaecrista (n=8)
Chloroleucon (n=2)
Clathrotropis (n=2)
Clitoria (n=3)
Cochliasanthus (n=1)
Cojoba (n=1)
Copaifera (n=9)
Cratylia (n=1)
Crotalaria (n=5)
Crudia (n=4)
Cymbosema (n=1)
Cynometra (n=2)
Deguelia (n=8)
Desmodium (n=4)
Dialium (n=1)
Dicorynia (n=1)
Dimorphandra (n=3)
Dinizia (n=1)
Dioclea (n=2)
Diplotropis (n=4)
Dipteryx (n=7)
Dussia (n=1)
Entada (n=1)
Enterolobium (n=4)
Eperua (n=7)
Eriosema (n=1)
Erythrina (n=9)
Galactia (n=1)
Grona (n=1)
Guilandina (n=1)
Gwilymia (n=1)
Helicotropis (n=1)
Heterostemon (n=2)
Hydrochorea (n=3)
Hymenaea (n=6)
Hymenolobium (n=6)
Inga (n=83)
Jupunba (n=7)
Lecointea (n=2)
Leptolobium (n=3)
Leptospron (n=1)
Leucaena (n=1)
Libidibia (n=2)
Lonchocarpus (n=5)
Machaerium (n=16)
Macrolobium (n=12)
Macropsychanthus (n=1)
Macroptilium (n=2)
Macrosamanea (n=4)
Marlimorimia (n=1)
Martiodendron (n=1)
Mimosa (n=17)
Monopteryx (n=2)
Mucuna (n=4)
Muellera (n=1)
Myrocarpus (n=1)
Myroxylon (n=2)
Neptunia (n=1)
Ormosia (n=15)
Pachyrhizus (n=1)
Paloue (n=2)
Paramachaerium (n=1)
Parkia (n=11)
Peltogyne (n=4)
Peltophorum (n=1)
Pentaclethra (n=1)
Piptadenia (n=3)
Plathymenia (n=1)
Platymiscium (n=3)
Platypodium (n=1)
Poeppigia (n=1)
Pterocarpus (n=4)
Pterodon (n=2)
Rhynchosia (n=1)
Samanea (n=1)
Schizolobium (n=1)
Schnella (n=3)
Senegalia (n=5)
Senna (n=20)
Soemmeringia (n=1)
Stryphnodendron (n=6)
Stylosanthes (n=2)
Swartzia (n=35)
Tachigali (n=15)
Taralea (n=3)
Tephrosia (n=3)
Tipuana (n=1)
Trischidium (n=1)
Vachellia (n=3)
Vatairea (n=4)
Vataireopsis (n=2)
Vigna (n=2)
Vouacapoua (n=1)
Zollernia (n=2)
Zornia (n=2)
Zygia (n=14)
Genus (n species analysed)

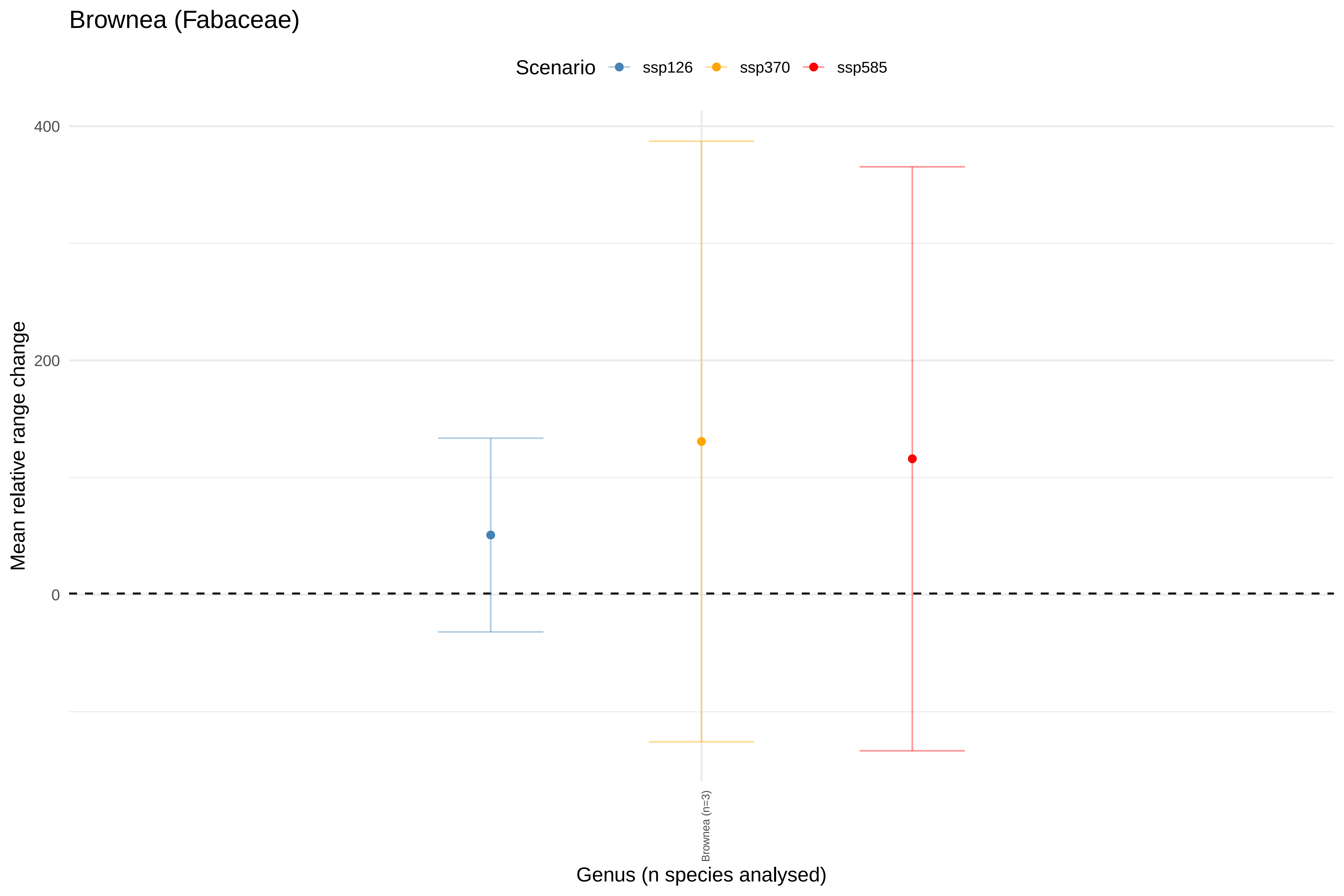

Brownea (Fabaceae)
Scenario
ssp126
ssp370
ssp585
400
200
0
Mean relative range change
Brownea (n=3)
Genus (n species analysed)

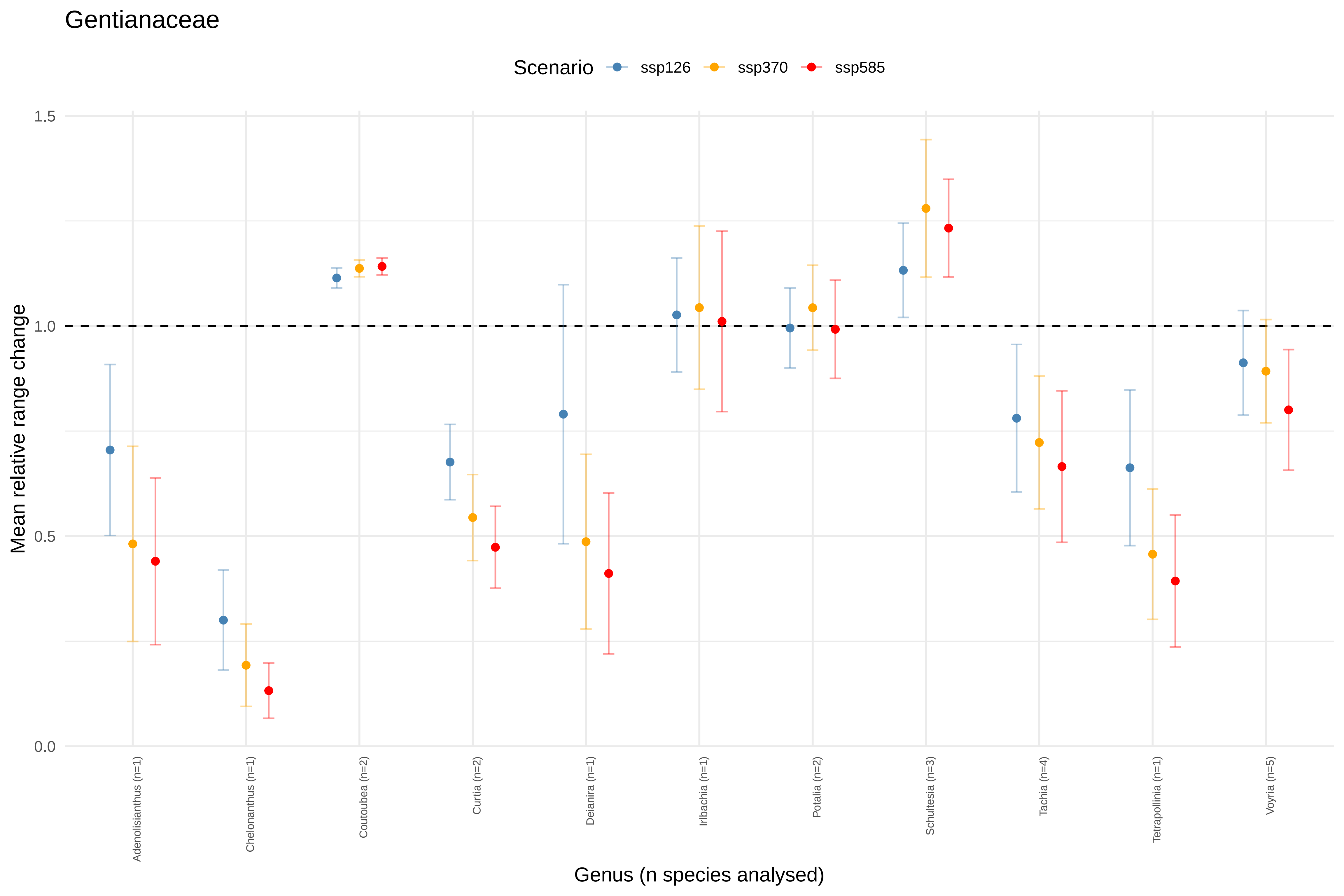

Gentianaceae
Scenario
ssp126
ssp370
ssp585
Mean relative range change
1.5
1.0
0.5
0.0
Adenolisianthus (n=1)
Chelonanthus (n=1)
Coutoubea (n=2)
Curtia (n=2)
Deianira (n=1)
Irlbachia (n=1)
Potalia (n=2)
Schultesia (n=3)
Tachia (n=4)
Tetrapollinia (n=1)
Voyria (n=5)
Genus (n species analysed)

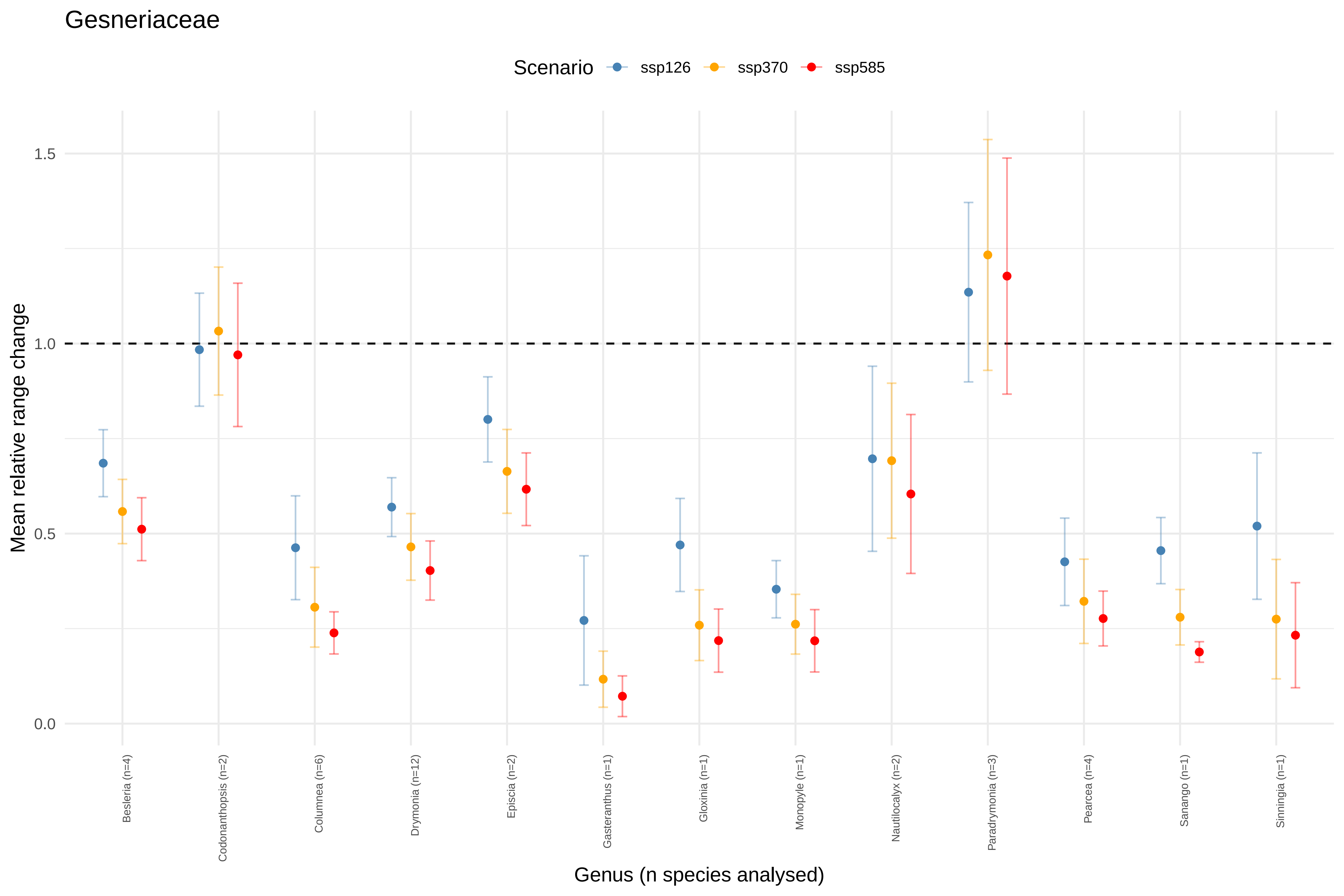

Gesneriaceae
Scenario
ssp126
ssp370
ssp585
Mean relative range change
1.5
1.0
0.5
0.0
Besleria (n=4)
Codonanthopsis (n=2)
Columnea (n=6)
Drymonia (n=12)
Episcia (n=2)
Gasteranthus (n=1)
Gloxinia (n=1)
Monopyle (n=1)
Nautilocalyx (n=2)
Paradrymonia (n=3)
Pearcea (n=4)
Sanango (n=1)
Sinningia (n=1)
Genus (n species analysed)

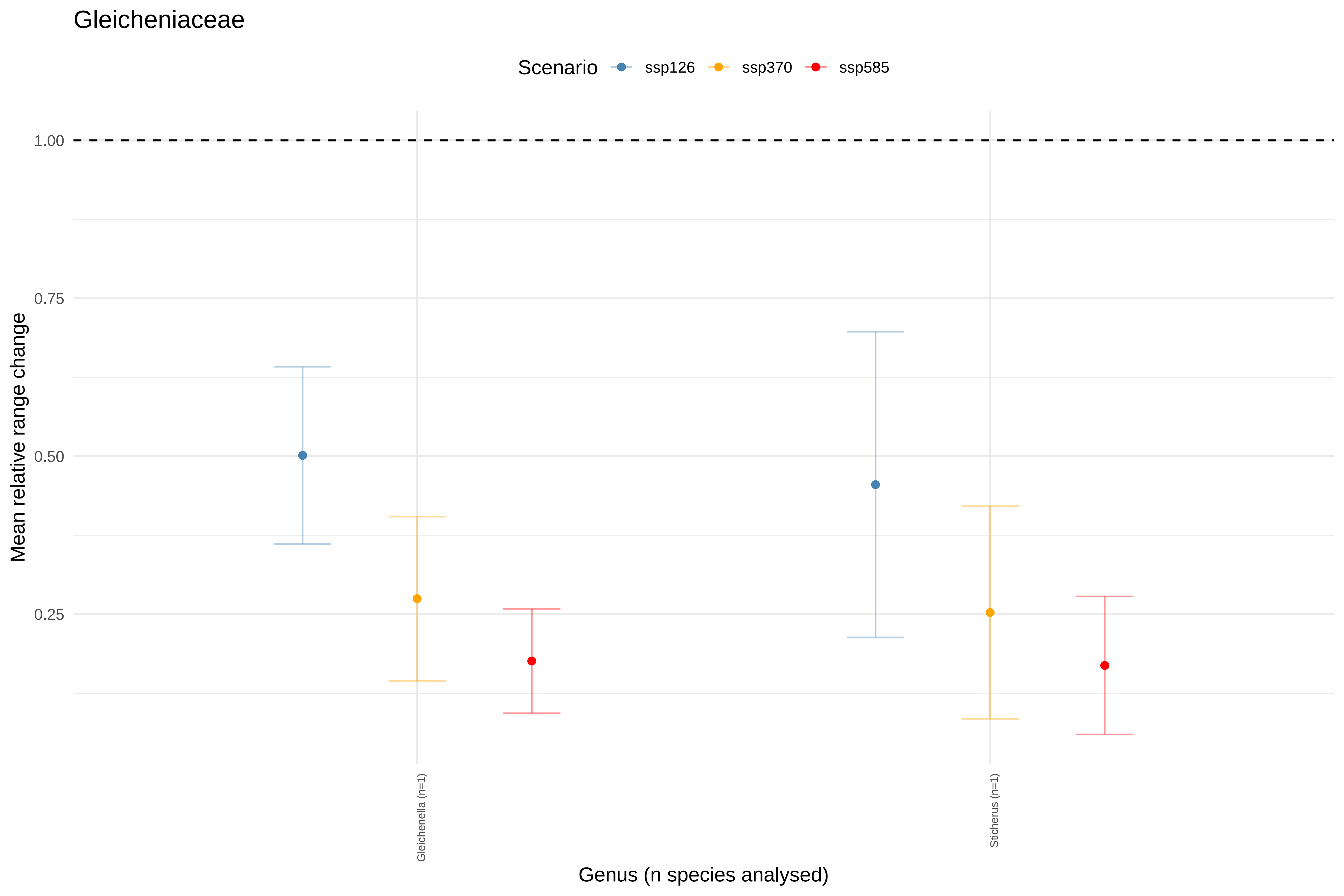

Gleicheniaceae
Scenario
ssp126
ssp370
ssp585
1.00
0.75
0.50
0.25
Mean relative range change
Gleichenella (n=1)
Sticherus (n=1)
Genus (n species analysed)

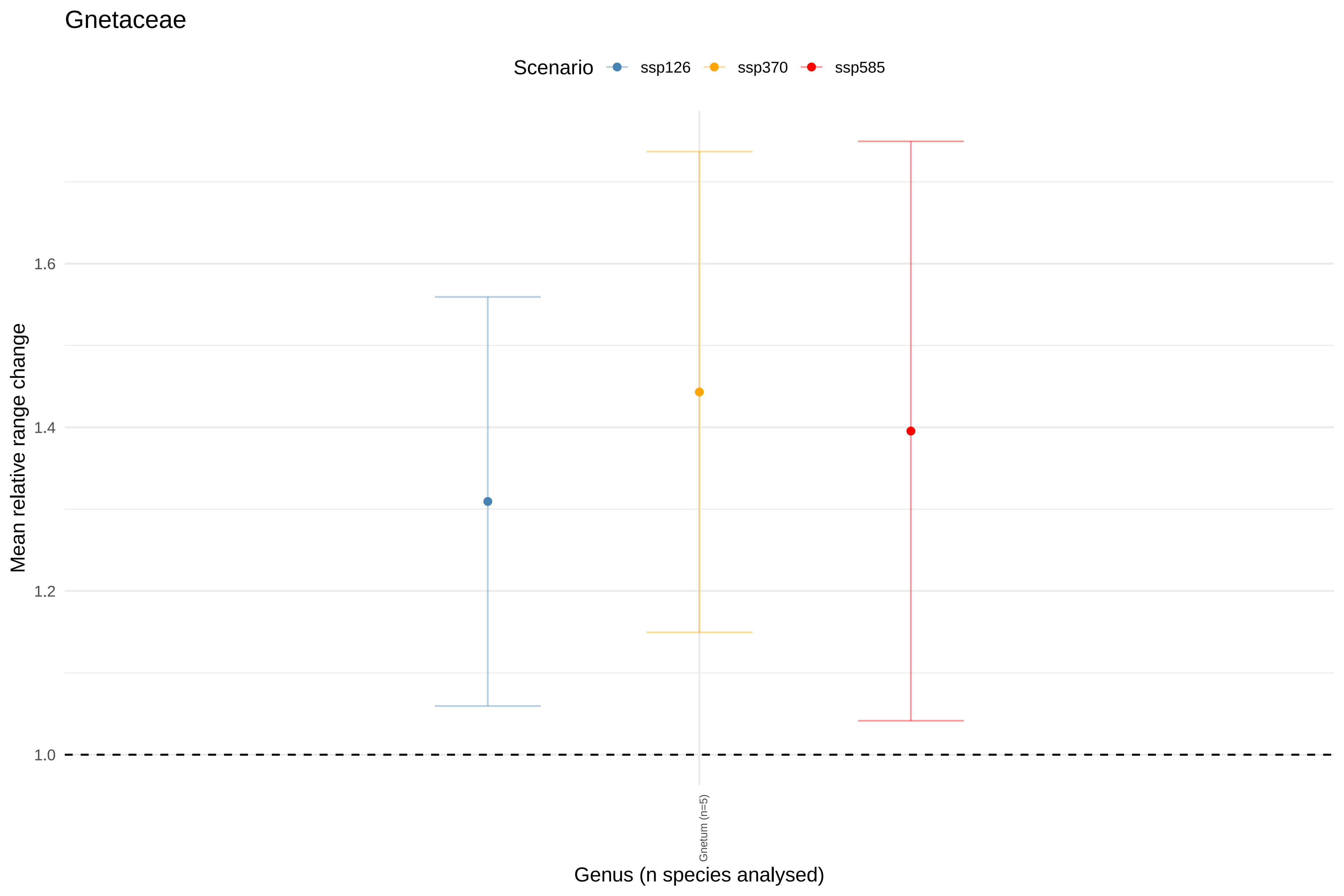
Gnetaceae
Scenario
ssp126
ssp370
ssp585
Mean relative range change
1.6
1.4
1.2
1.0
Gnetum (n=5)
Genus (n species analysed)

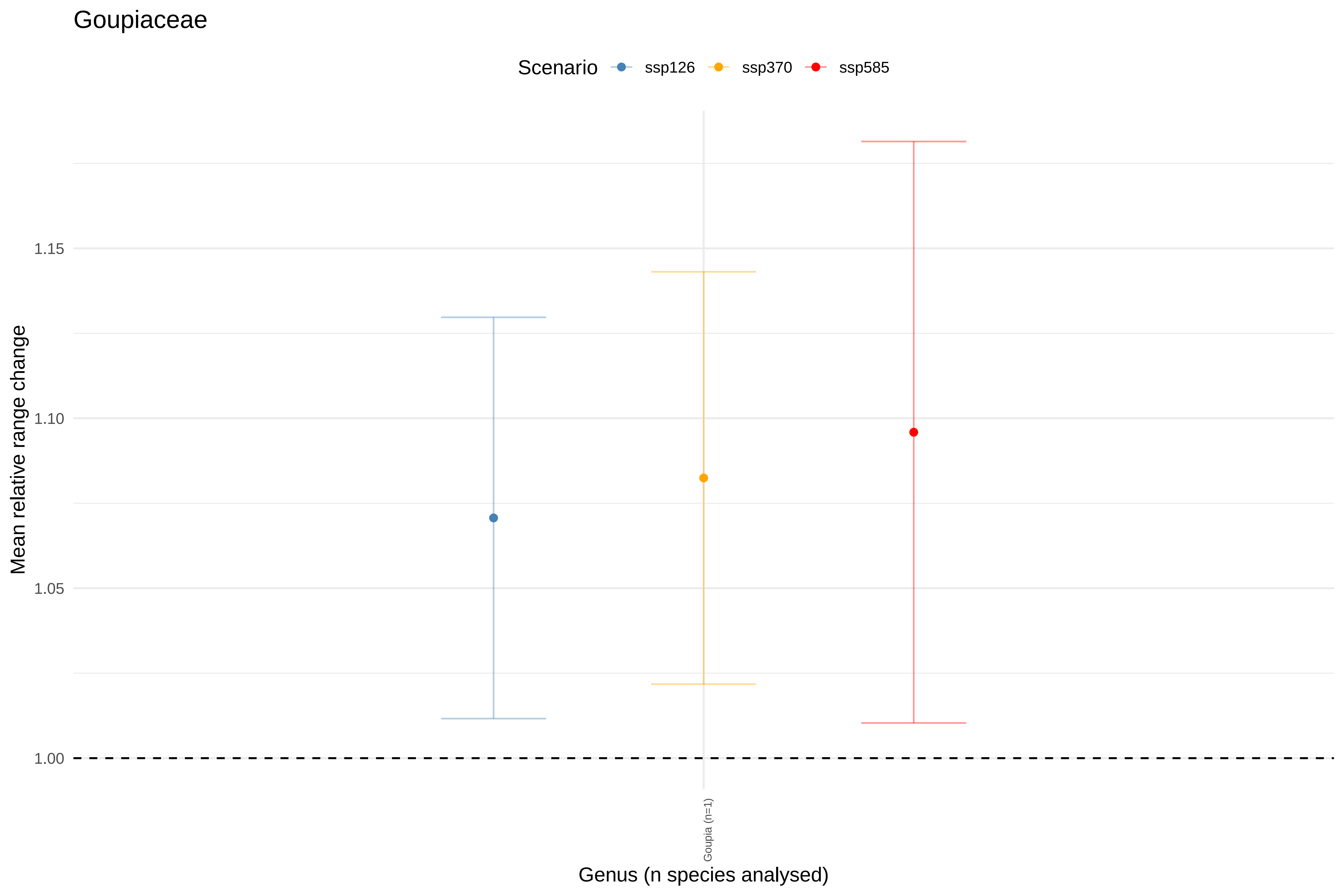

Goupiaceae
Scenario
ssp126
ssp370
ssp585
Mean relative range change
1.15
1.10
1.05
1.00
Goupia (n=1)
Genus (n species analysed)

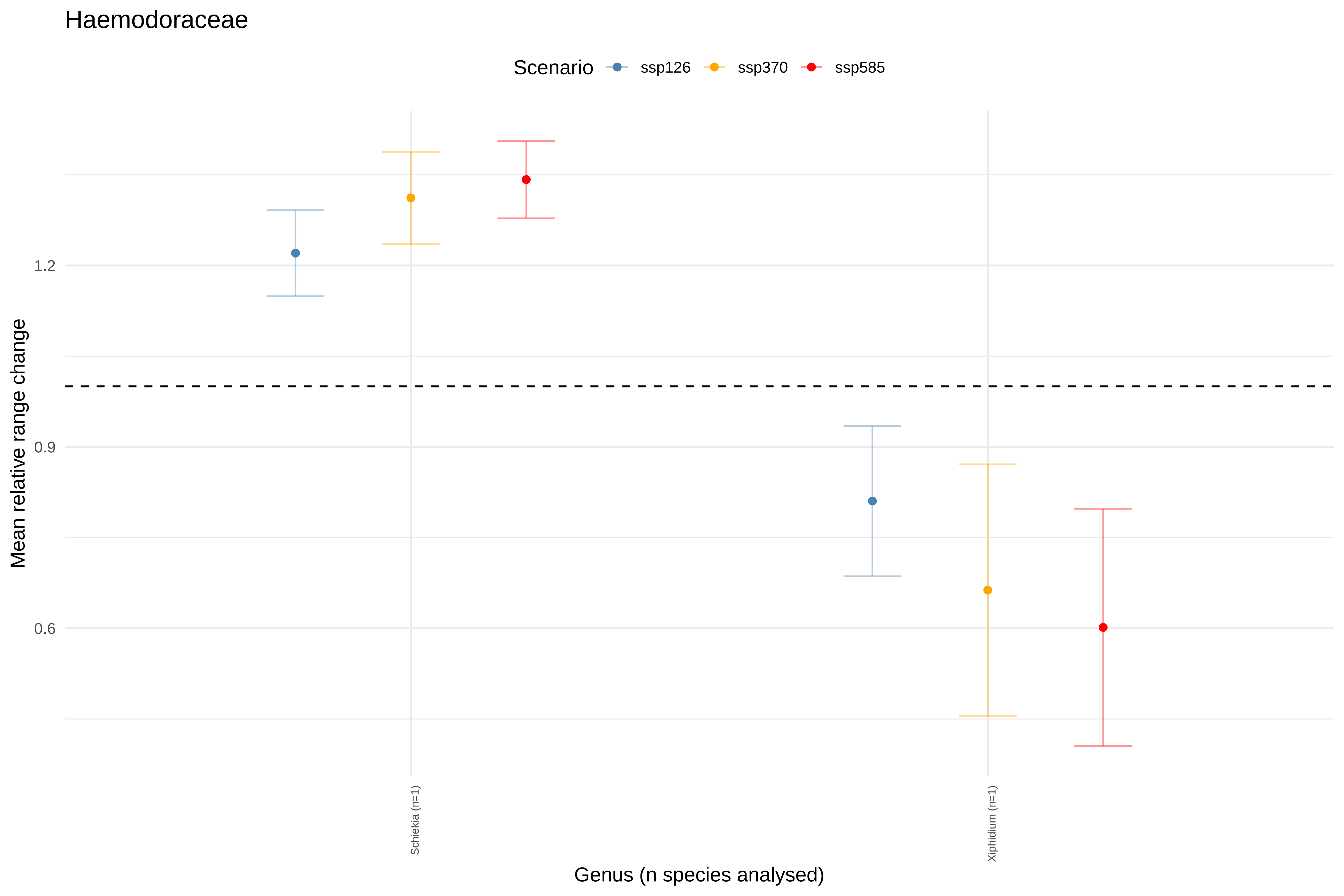

Haemodoraceae
Scenario
ssp126
ssp370
ssp585
Mean relative range change
1.2
0.9
0.6
Schiekia (n=1)
Xiphidium (n=1)
Genus (n species analysed)

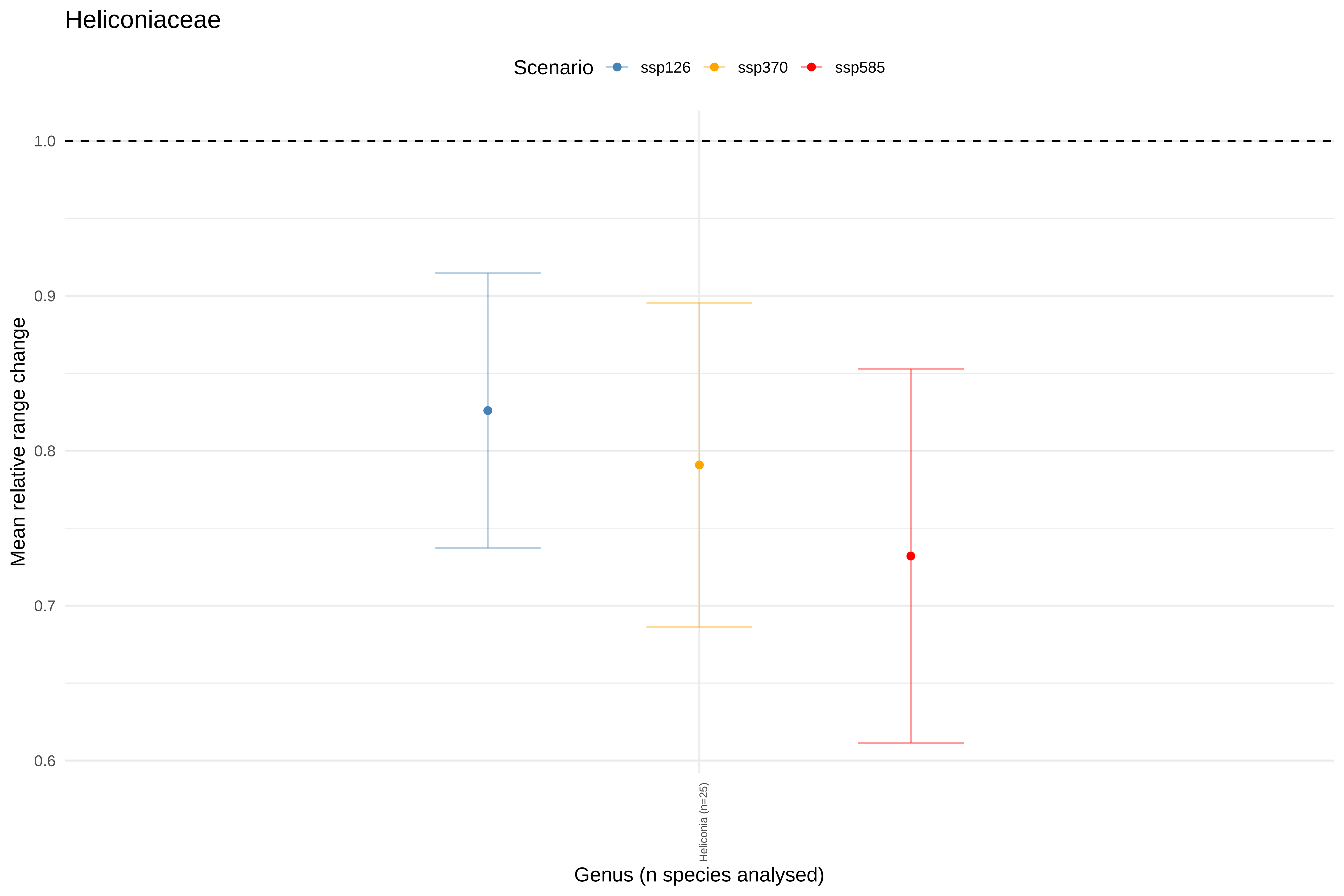

Heliconiaceae
Scenario
ssp126
ssp370
ssp585
Mean relative range change
1.0
0.9
0.8
0.7
0.6
Heliconia (n=25)
Genus (n species analysed)

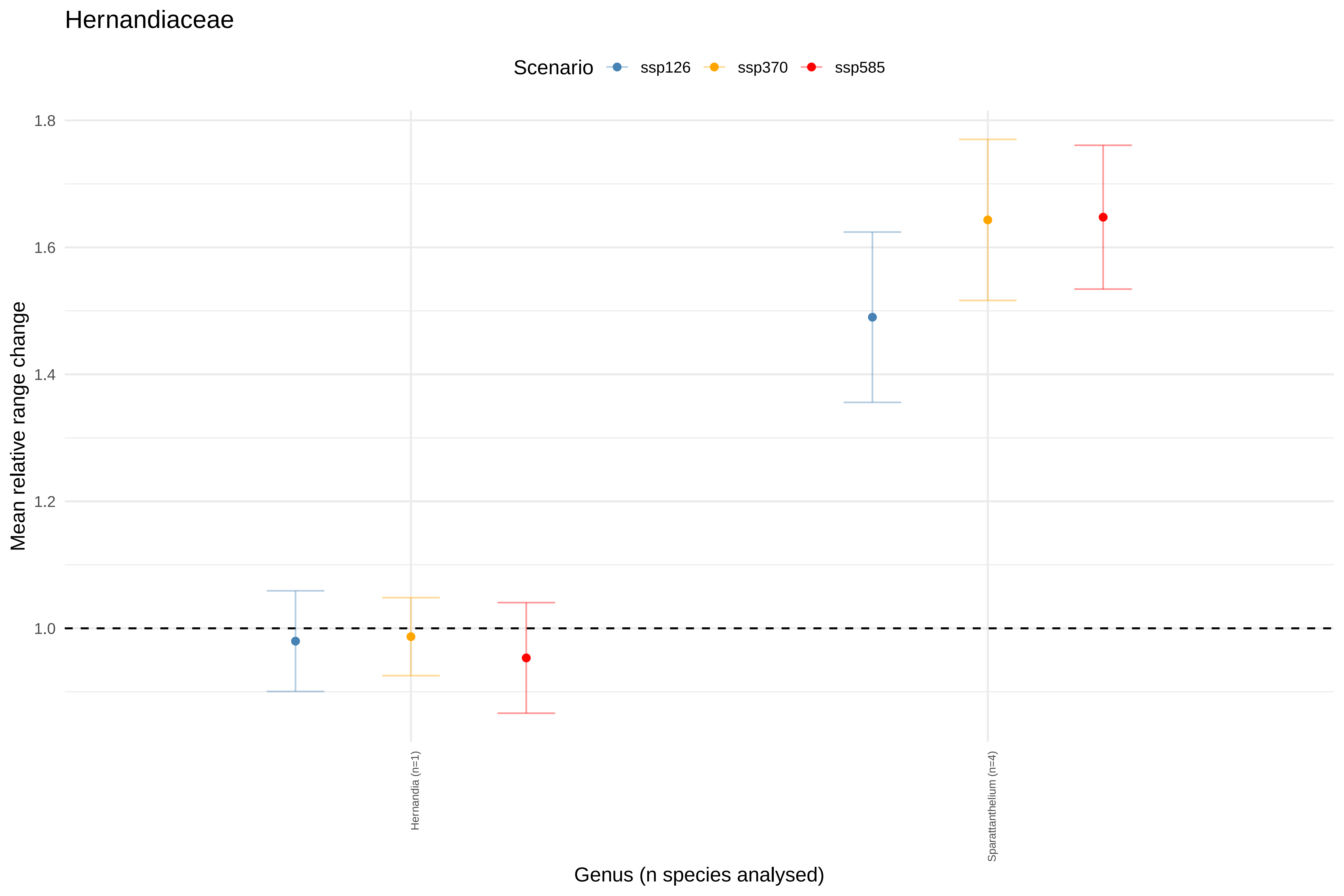

Hernandiaceae
Scenario
ssp126
ssp370
ssp585
1.8
1.6
1.4
1.2
1.0
Mean relative range change
Hernandia (n=1)
Sparattanthelium (n=4)
Genus (n species analysed)

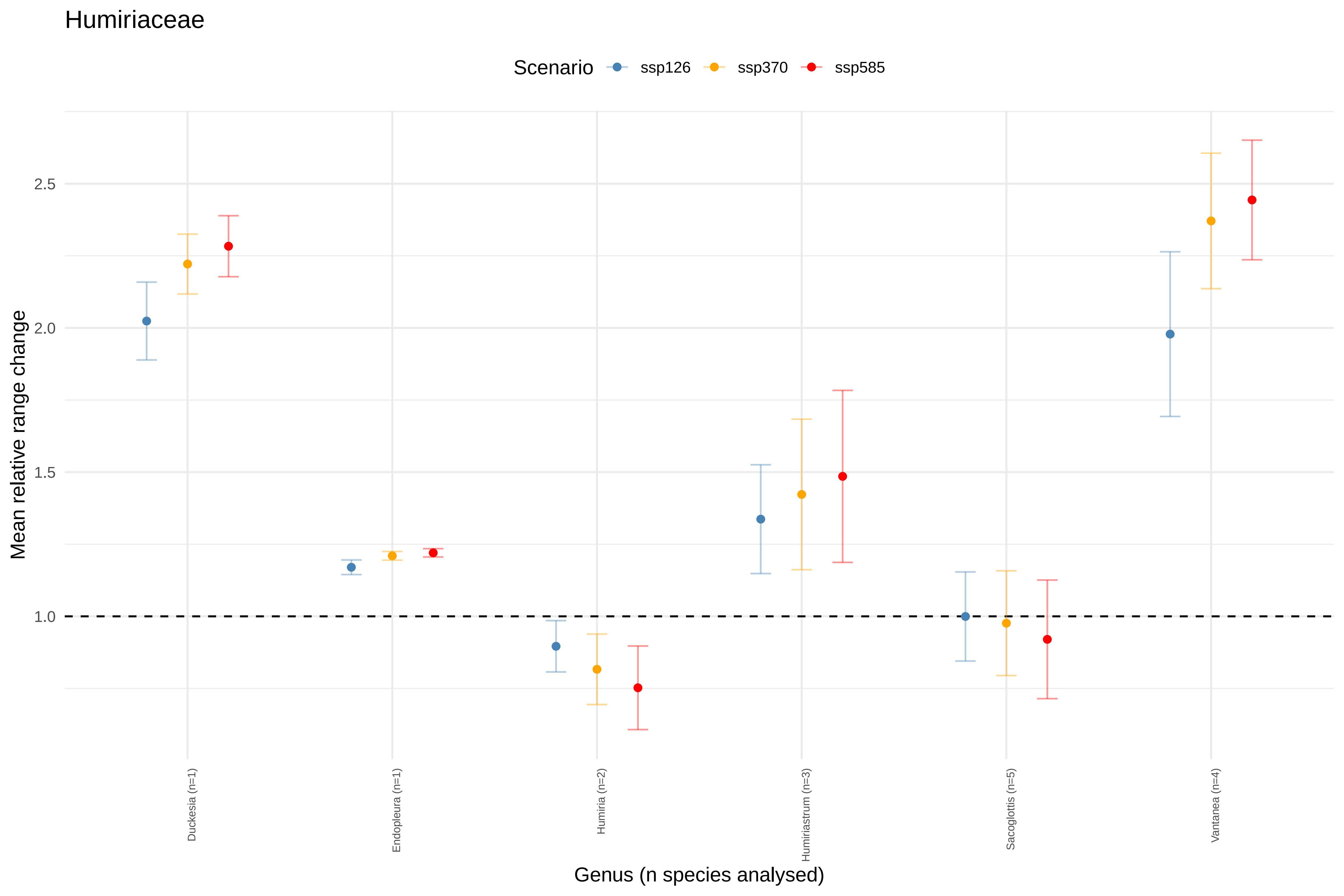
Humiriaceae
Scenario
ssp126
ssp370
ssp585
Mean relative range change
2.5
2.0
1.5
1.0
Duckesia (n=1)
Endopleura (n=1)
Humiria (n=2)
Humiriastrum (n=3)
Sacoglottis (n=5)
Vantanea (n=4)
Genus (n species analysed)

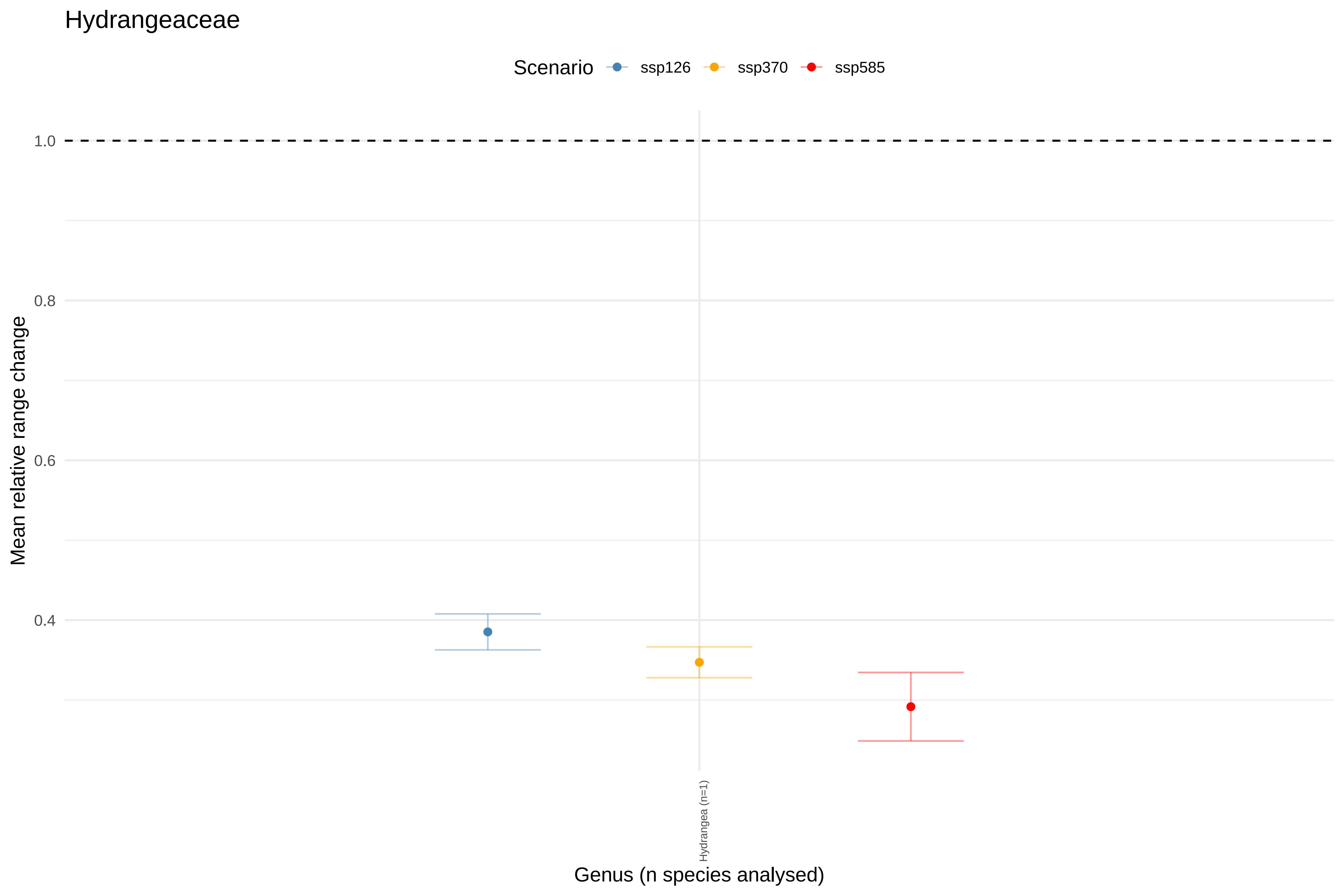
Hydrangeaceae
Scenario
ssp126
ssp370
ssp585
Mean relative range change
1.0
0.8
0.6
0.4
Hydrangea (n=1)
Genus (n species analysed)

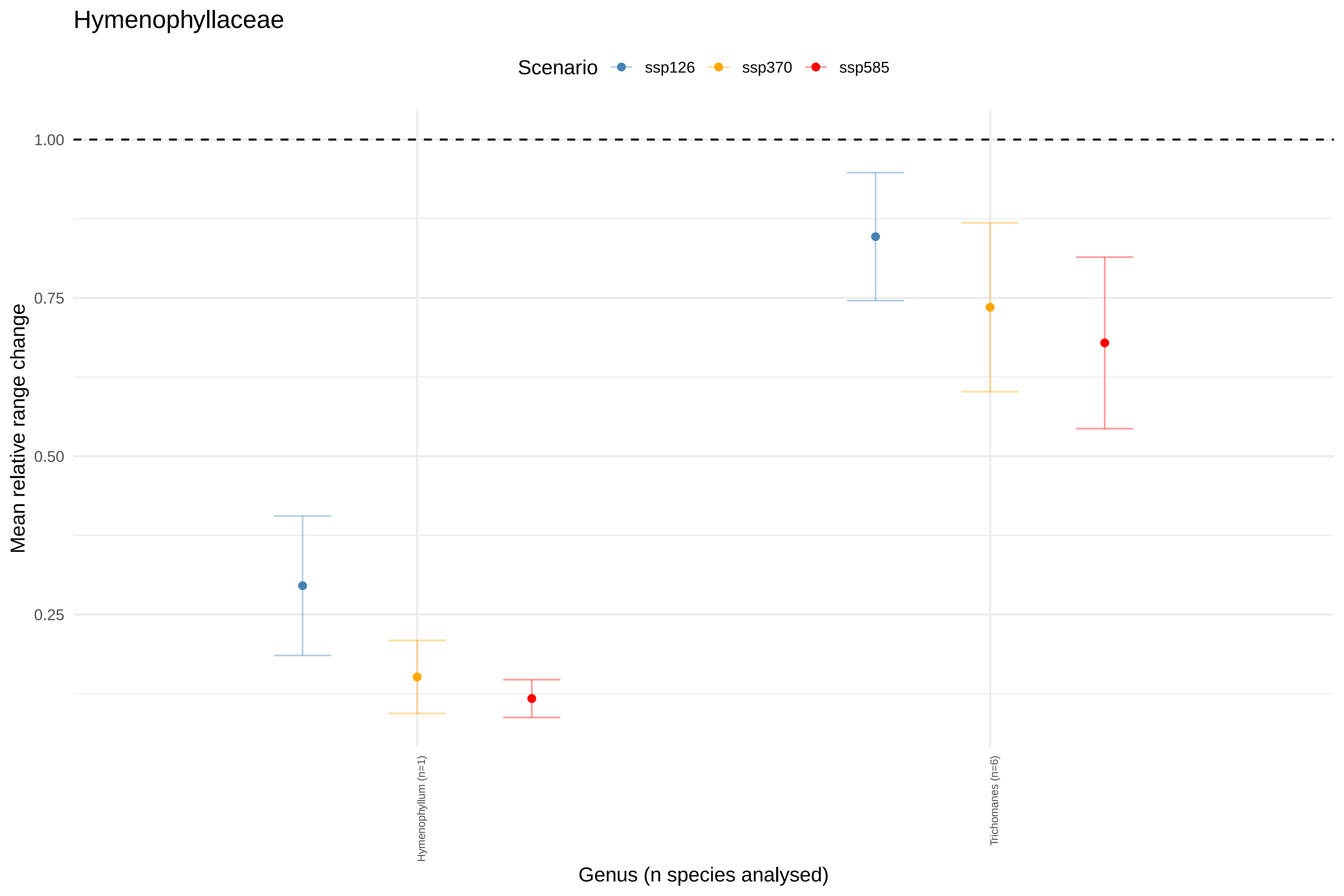

Hymenophyllaceae
Scenario
ssp126
ssp370
ssp585
Mean relative range change
1.00
0.75
0.50
0.25
Hymenophyllum (n=1)
Trichomanes (n=6)
Genus (n species analysed)

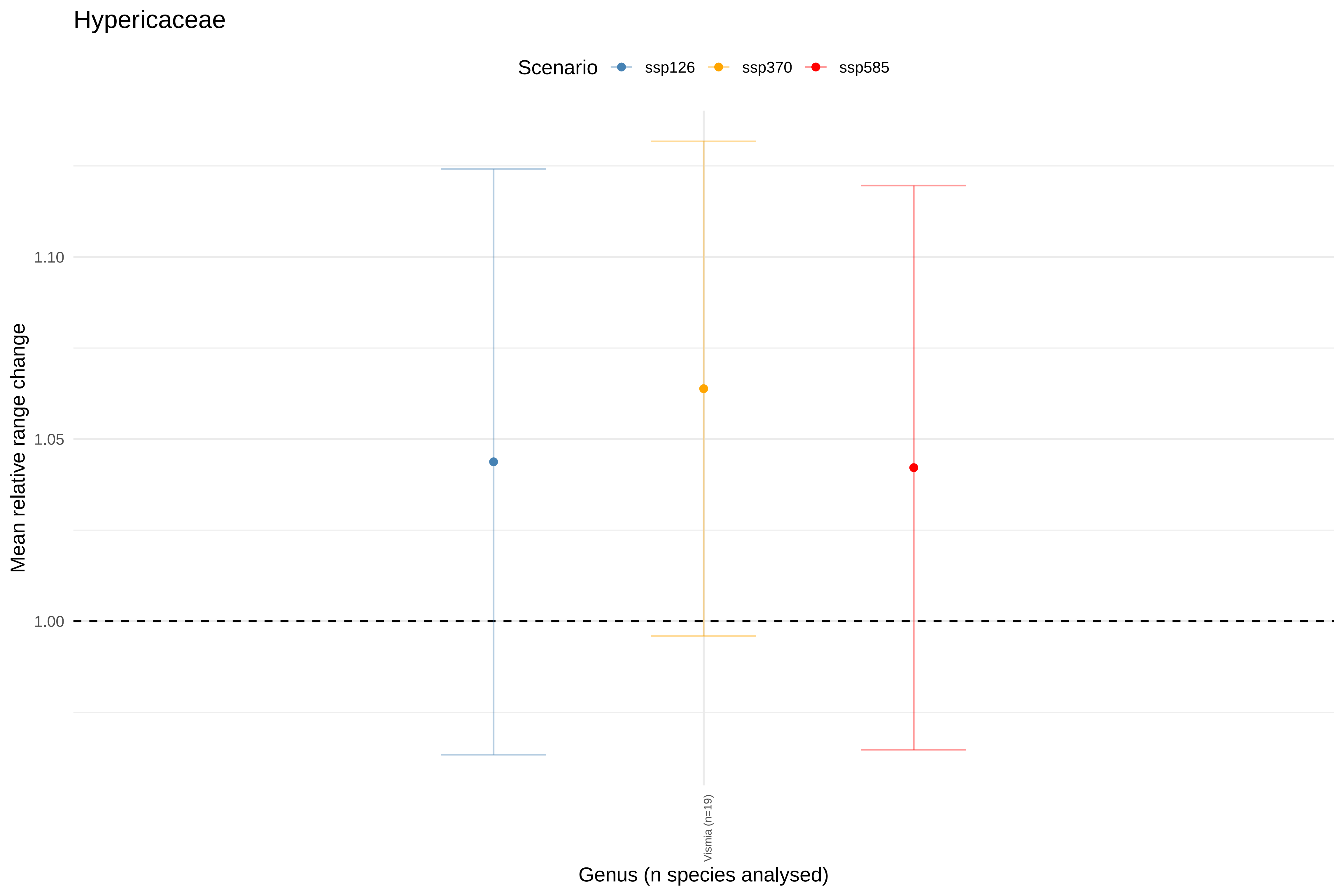

Hypericaceae
Scenario
ssp126
ssp370
ssp585
1.10
1.05
1.00
Mean relative range change
Vismia (n=19)
Genus (n species analysed)

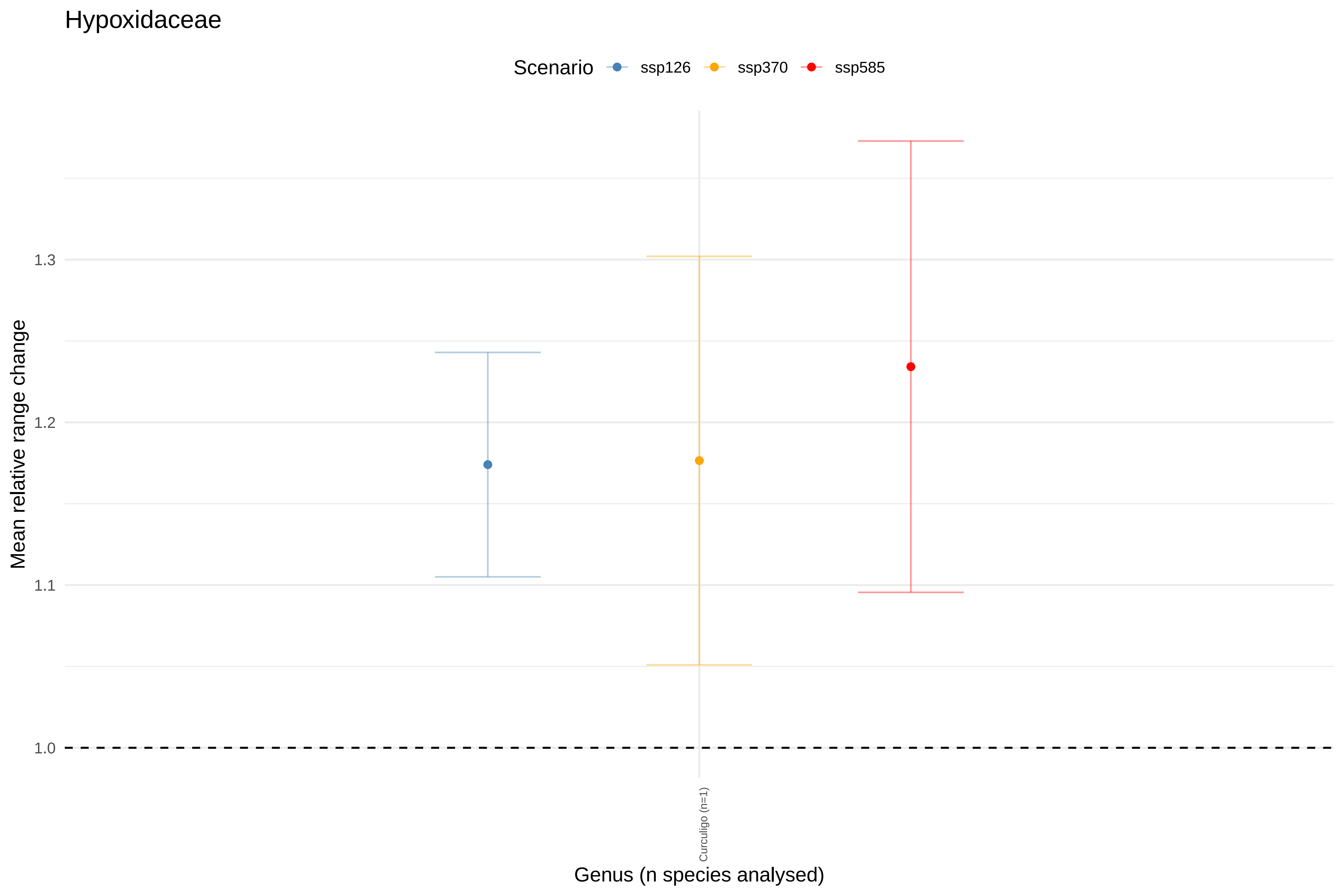

Hypoxidaceae
Scenario
ssp126
ssp370
ssp585
Mean relative range change
1.3
1.2
1.1
1.0
Curculigo (n=1)
Genus (n species analysed)

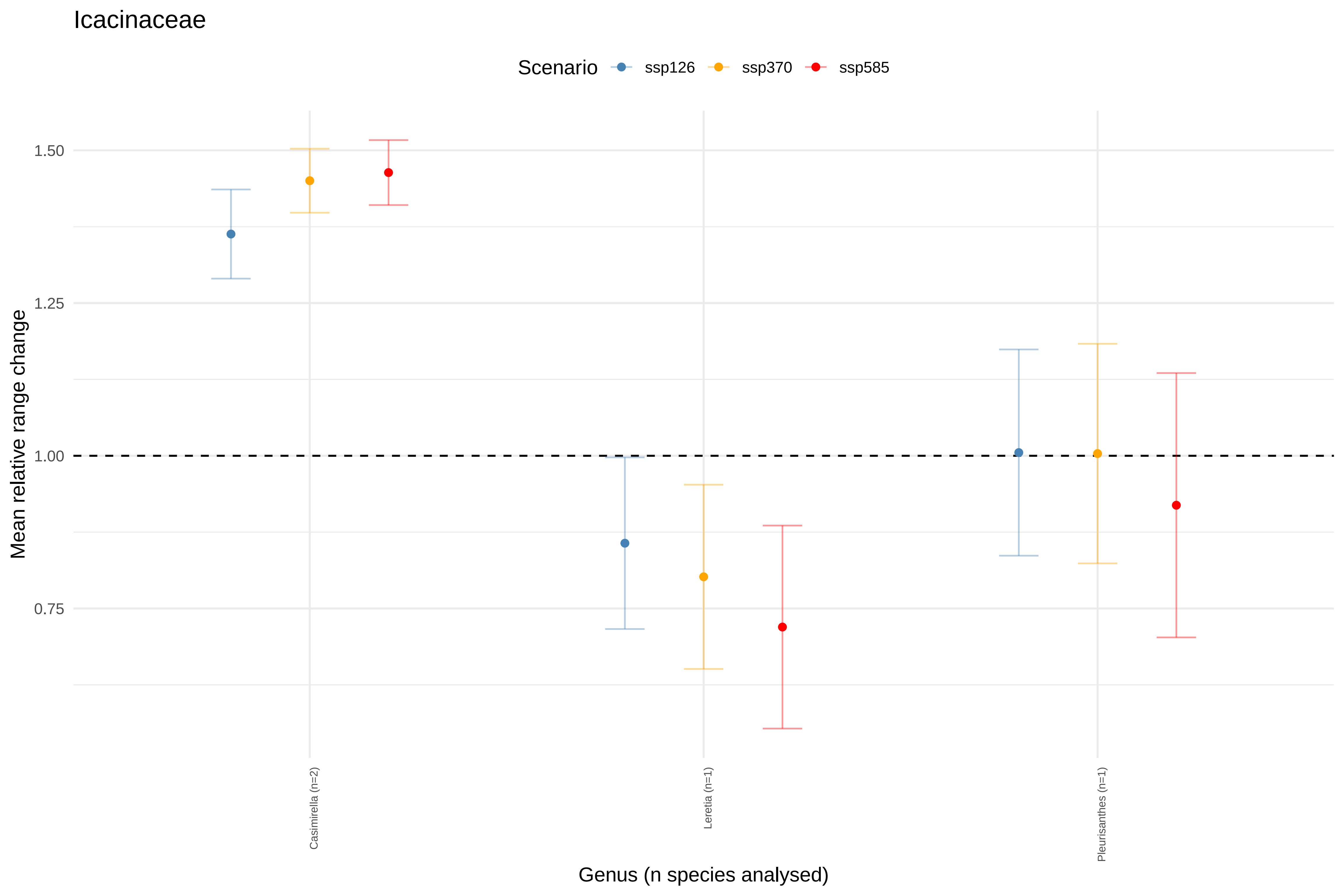

Icacinaceae
Scenario
ssp126
ssp370
ssp585
Mean relative range change
1.50
1.25
1.00
0.75
Casimirella (n=2)
Leretia (n=1)
Pleurisanthes (n=1)
Genus (n species analysed)

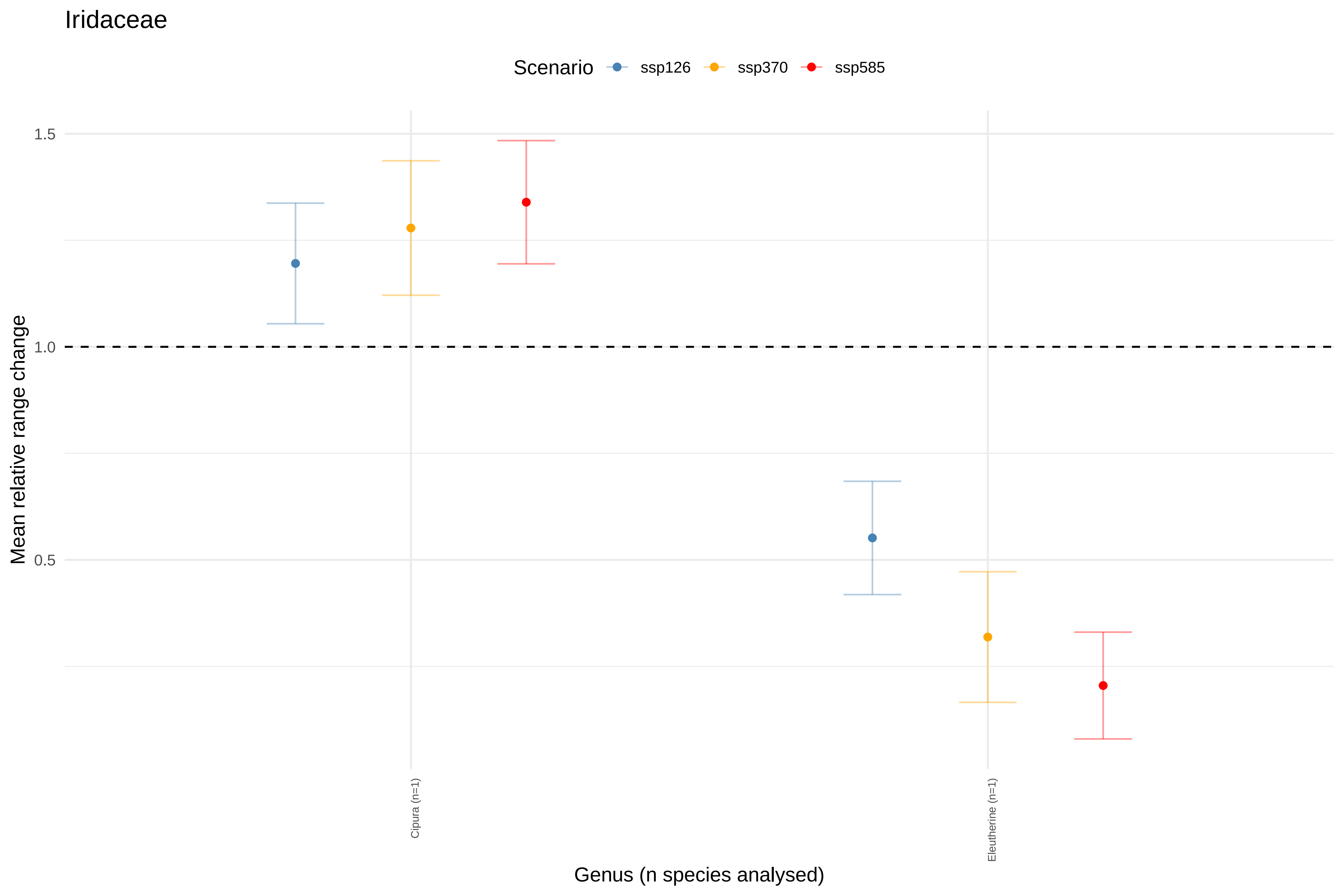
Iridaceae
Scenario
ssp126
ssp370
ssp585
1.5
1.0
0.5
Mean relative range change
Cipura (n=1)
Eleutherine (n=1)
Genus (n species analysed)

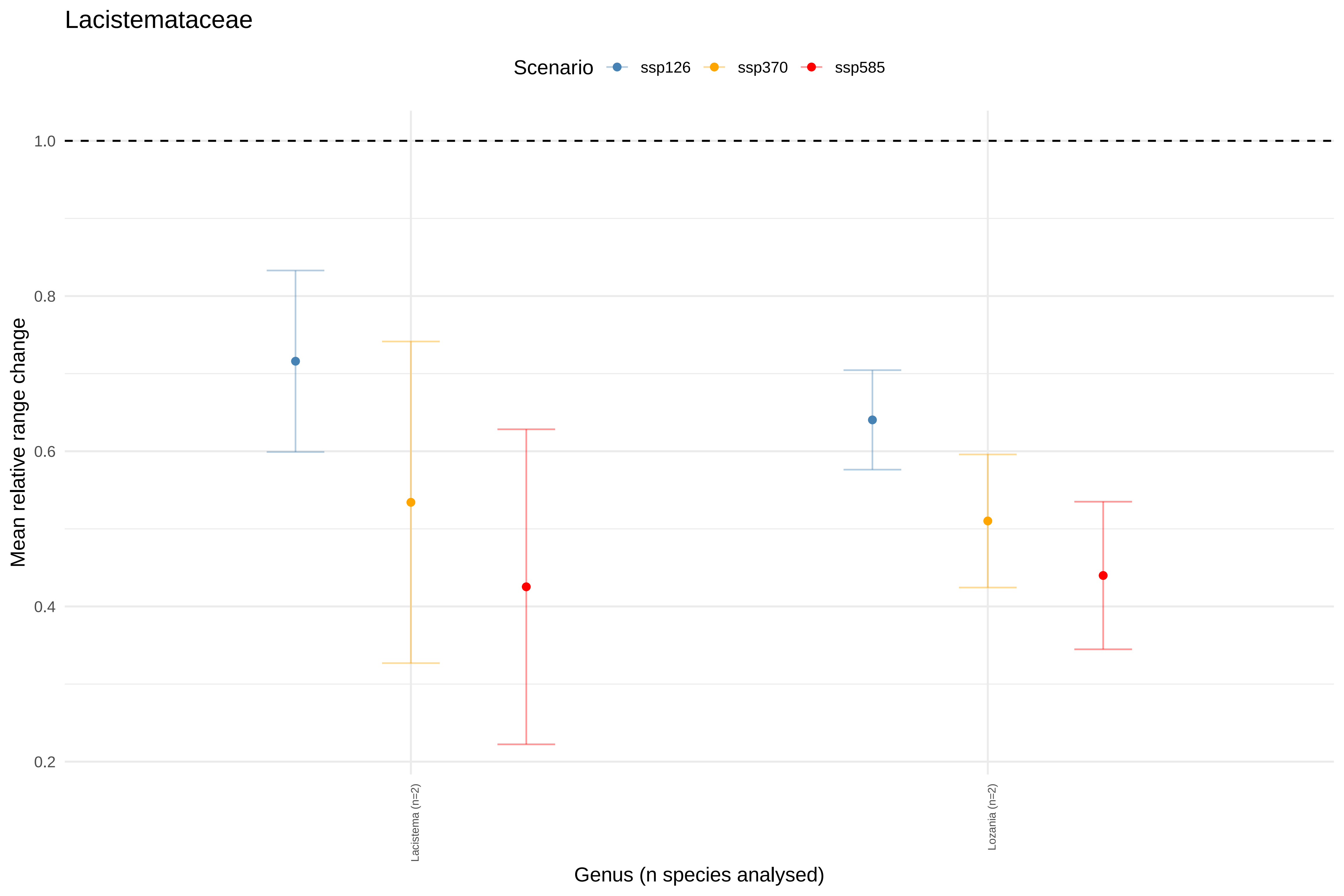

Lacistemataceae
Scenario
ssp126
ssp370
ssp585
Mean relative range change
1.0
0.8
0.6
0.4
0.2
Lacistema (n=2)
Lozania (n=2)
Genus (n species analysed)

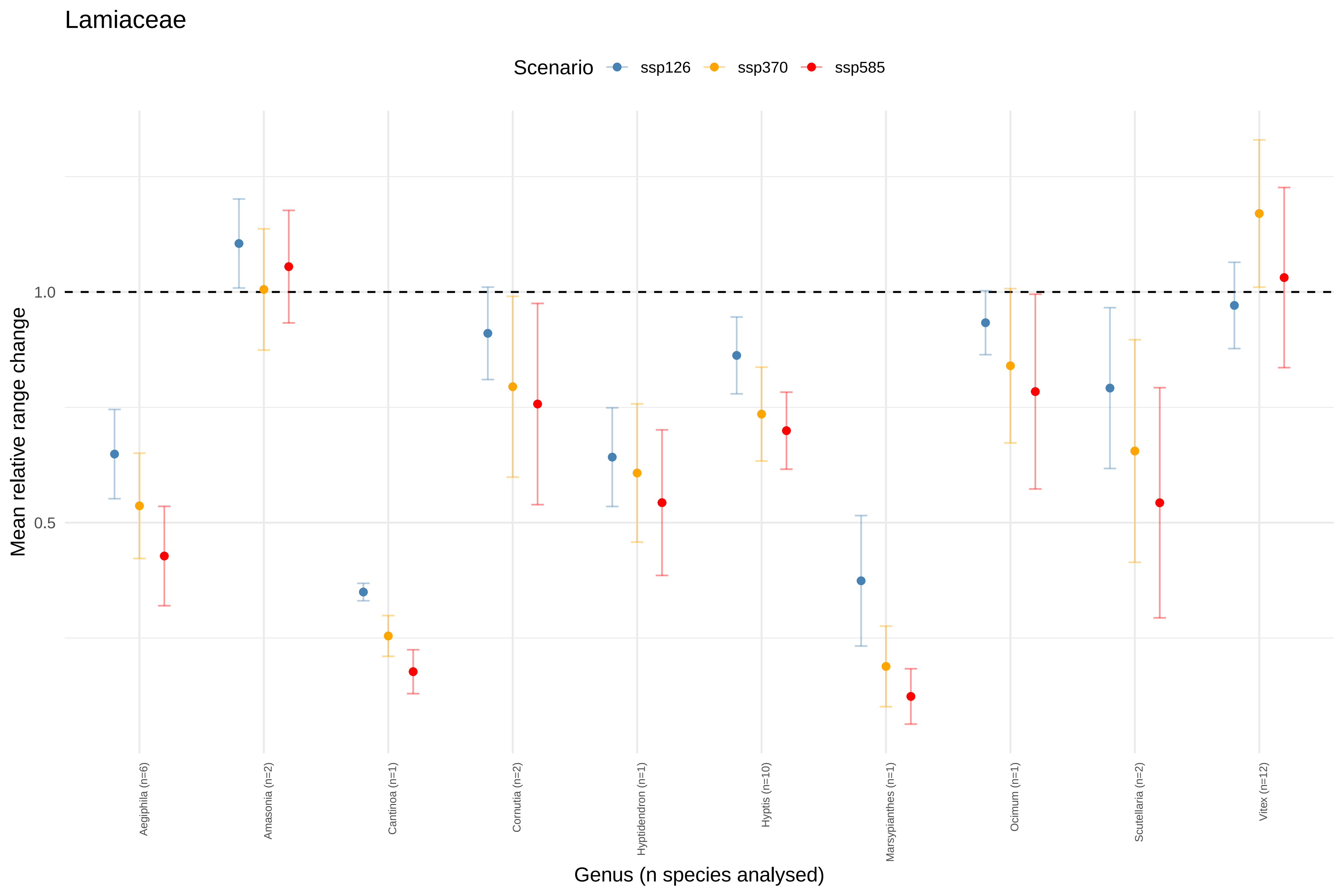
Lamiaceae
Scenario
ssp126
ssp370
ssp585
Mean relative range change
1.0
0.5
Aegiphila (n=6)
Amasonia (n=2)
Cantinoa (n=1)
Cornutia (n=2)
Hyptidendron (n=1)
Hyptis (n=10)
Marsypianthes (n=1)
Ocimum (n=1)
Scutellaria (n=2)
Vitex (n=12)
Genus (n species analysed)

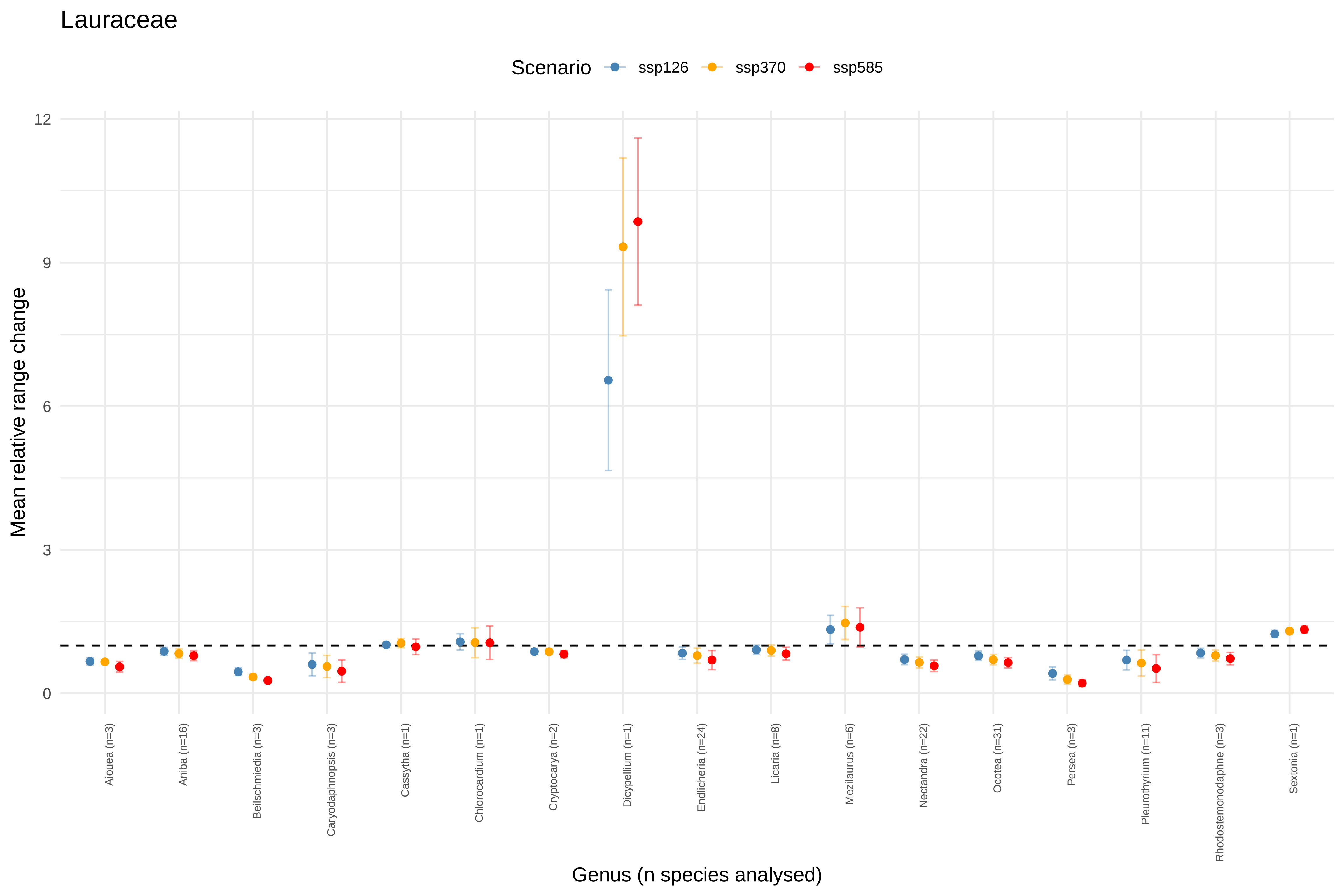

Lauraceae
Scenario
ssp126
ssp370
ssp585
Mean relative range change
0
3
6
9
12
Aiouea (n=3)
Aniba (n=16)
Beilschmiedia (n=3)
Caryodaphnopsis (n=3)
Cassytha (n=1)
Chlorocardium (n=1)
Cryptocarya (n=2)
Dicypellium (n=1)
Endlicheria (n=24)
Licaria (n=8)
Mezilaurus (n=6)
Nectandra (n=22)
Ocotea (n=31)
Persea (n=3)
Pleurothyrium (n=11)
Rhodostemonodaphne (n=3)
Sextonia (n=1)
Genus (n species analysed)

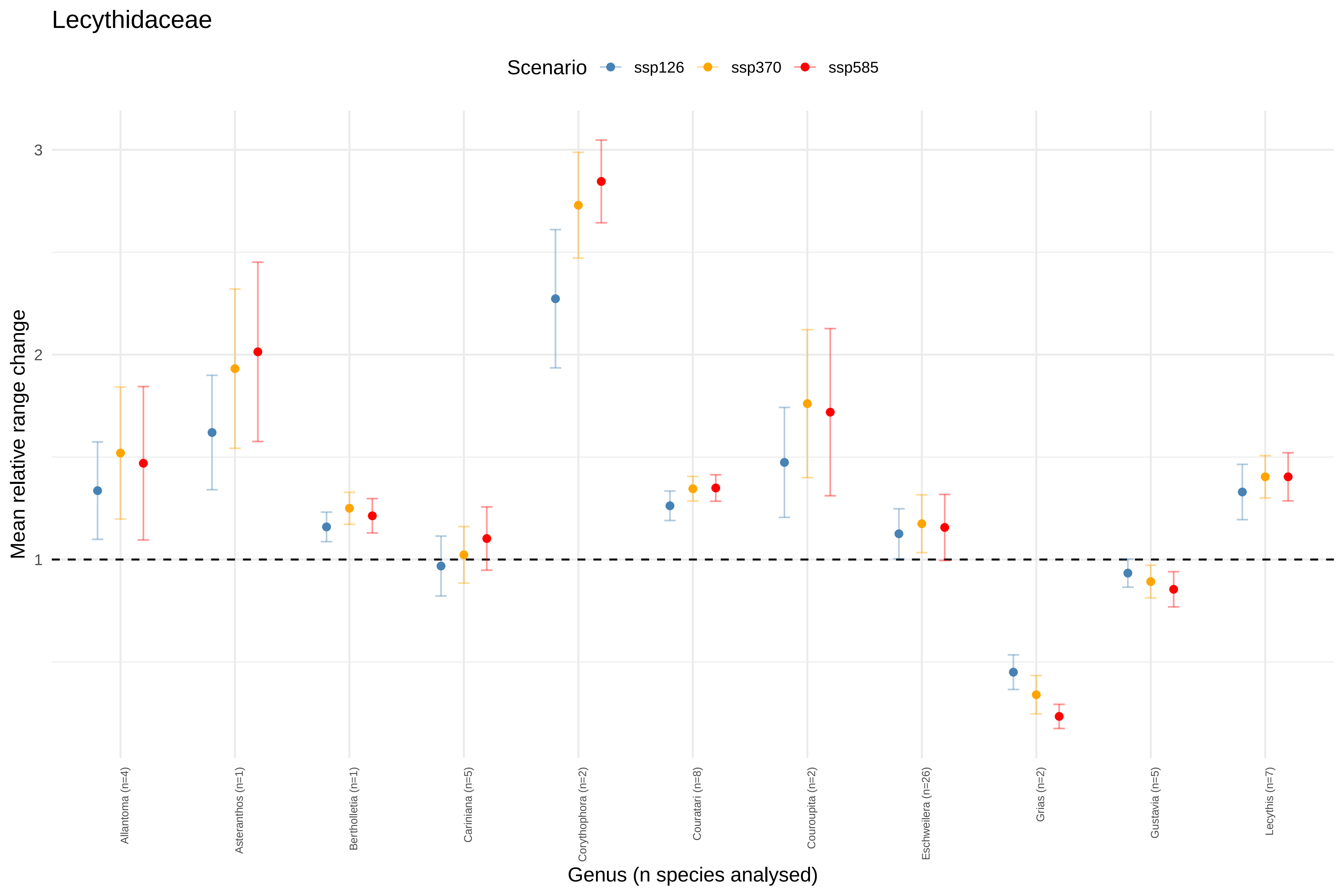

Lecythidaceae
Scenario
ssp126
ssp370
ssp585
Mean relative range change
3
2
1
Allantoma (n=4)
Asteranthos (n=1)
Bertholletia (n=1)
Cariniana (n=5)
Corythophora (n=2)
Couratari (n=8)
Couroupita (n=2)
Eschweilera (n=26)
Grias (n=2)
Gustavia (n=5)
Lecythis (n=7)
Genus (n species analysed)

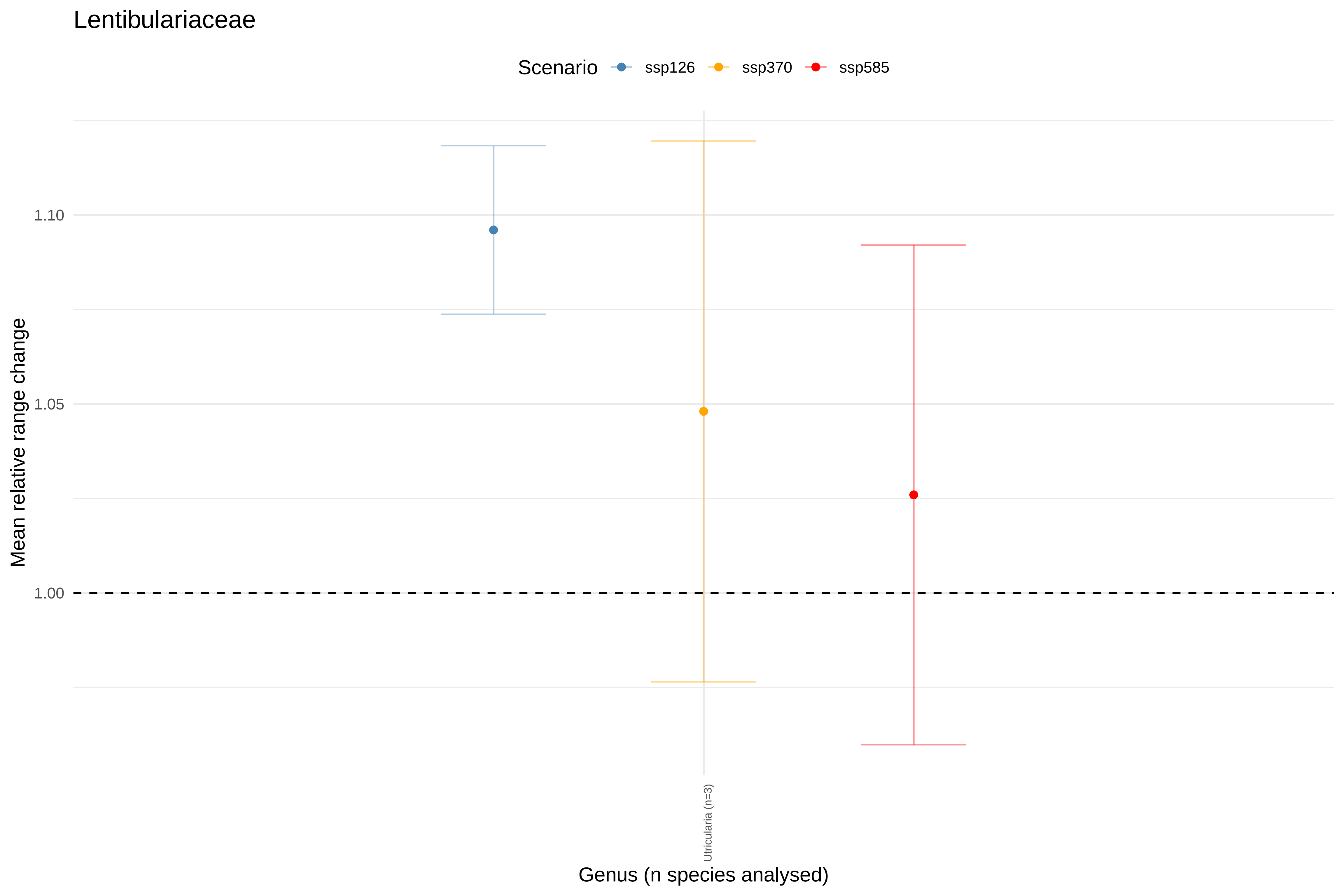

Lentibulariaceae
Scenario
ssp126
ssp370
ssp585
1.10
1.05
1.00
Mean relative range change
Utricularia (n=3)
Genus (n species analysed)

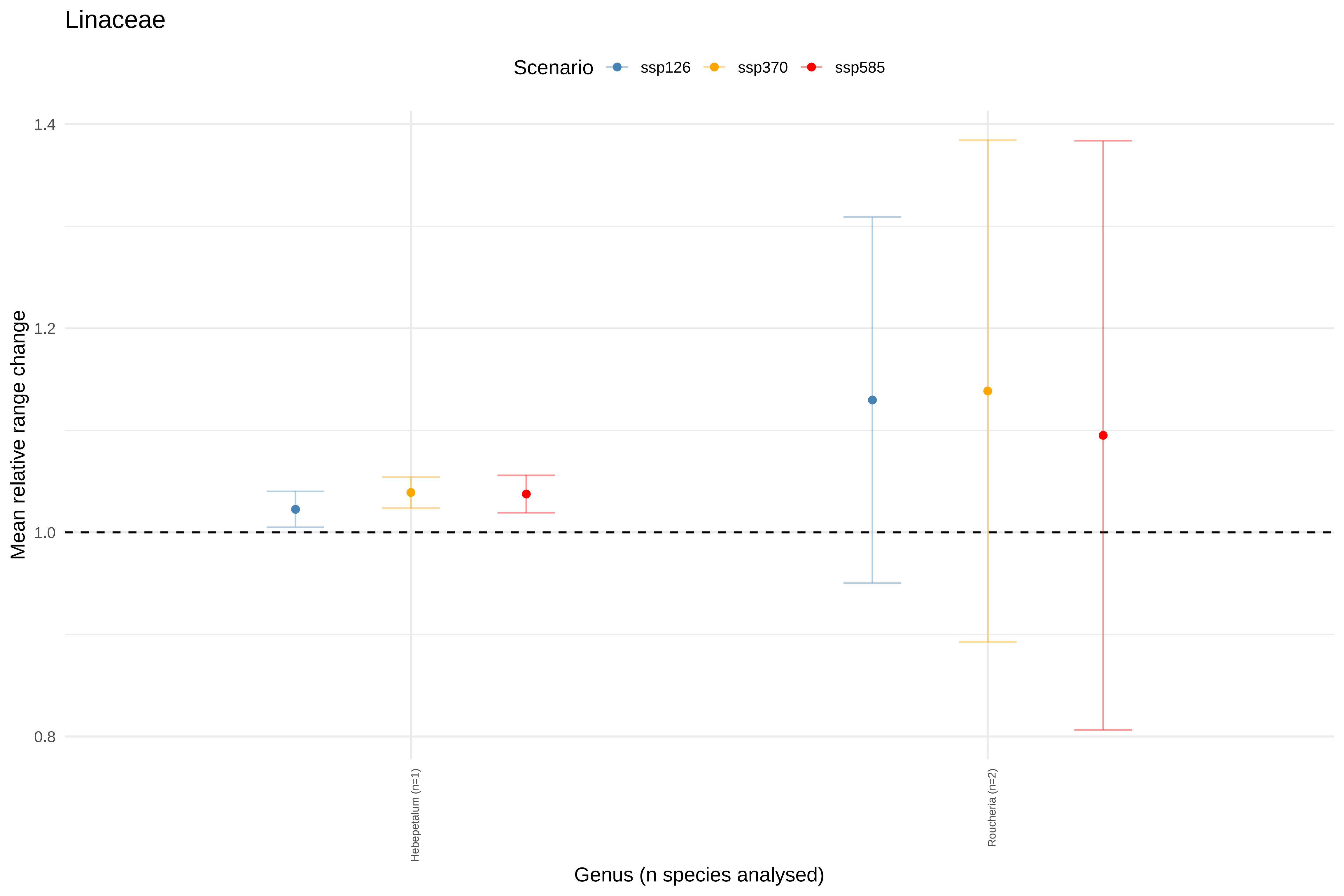

Linaceae
Scenario
ssp126
ssp370
ssp585
Mean relative range change
1.4
1.2
1.0
0.8
Hebepetalum (n=1)
Roucheria (n=2)
Genus (n species analysed)

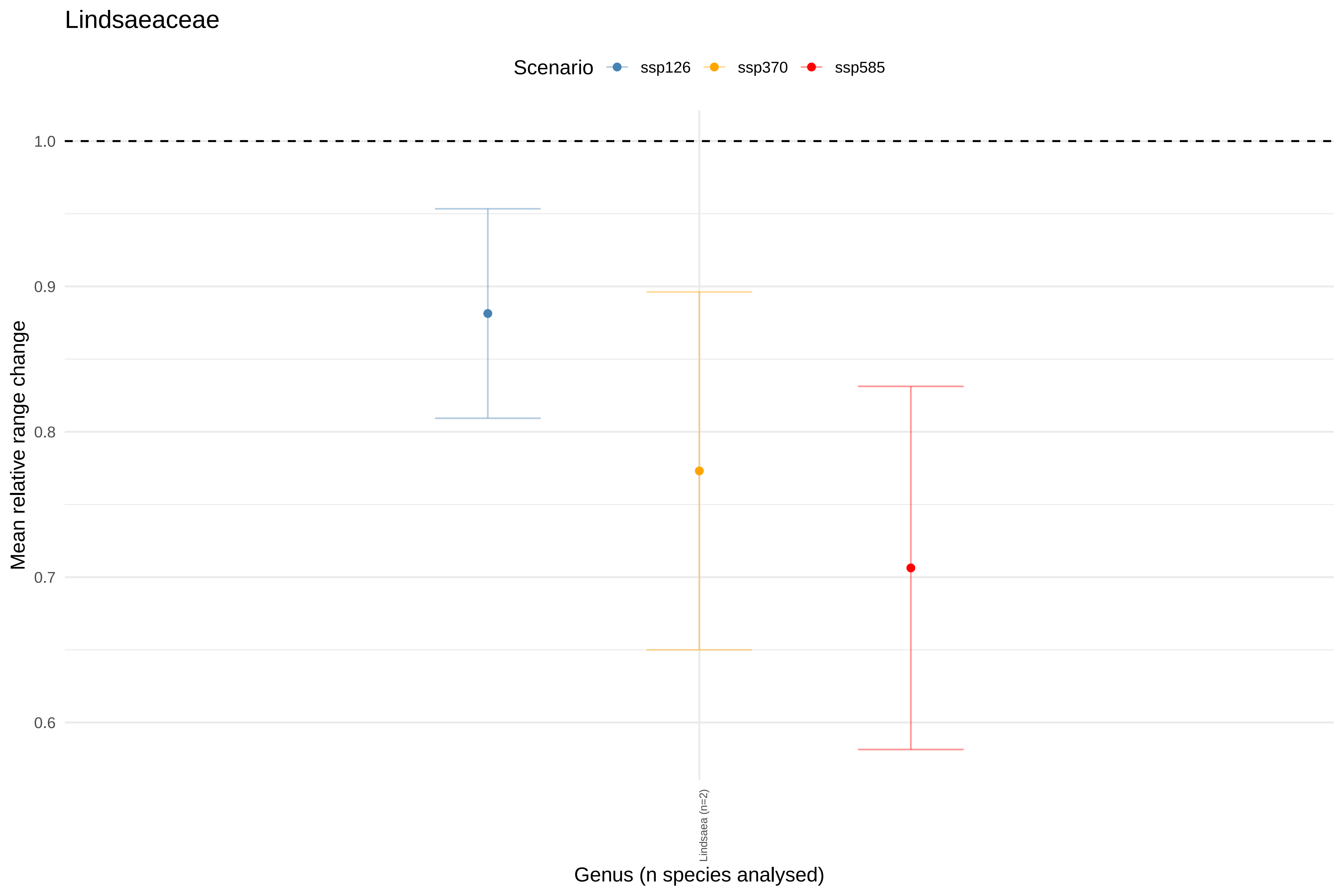

Lindsaeaceae
Scenario
ssp126
ssp370
ssp585
1.0
0.9
0.8
0.7
0.6
Mean relative range change
Lindsaea (n=2)
Genus (n species analysed)

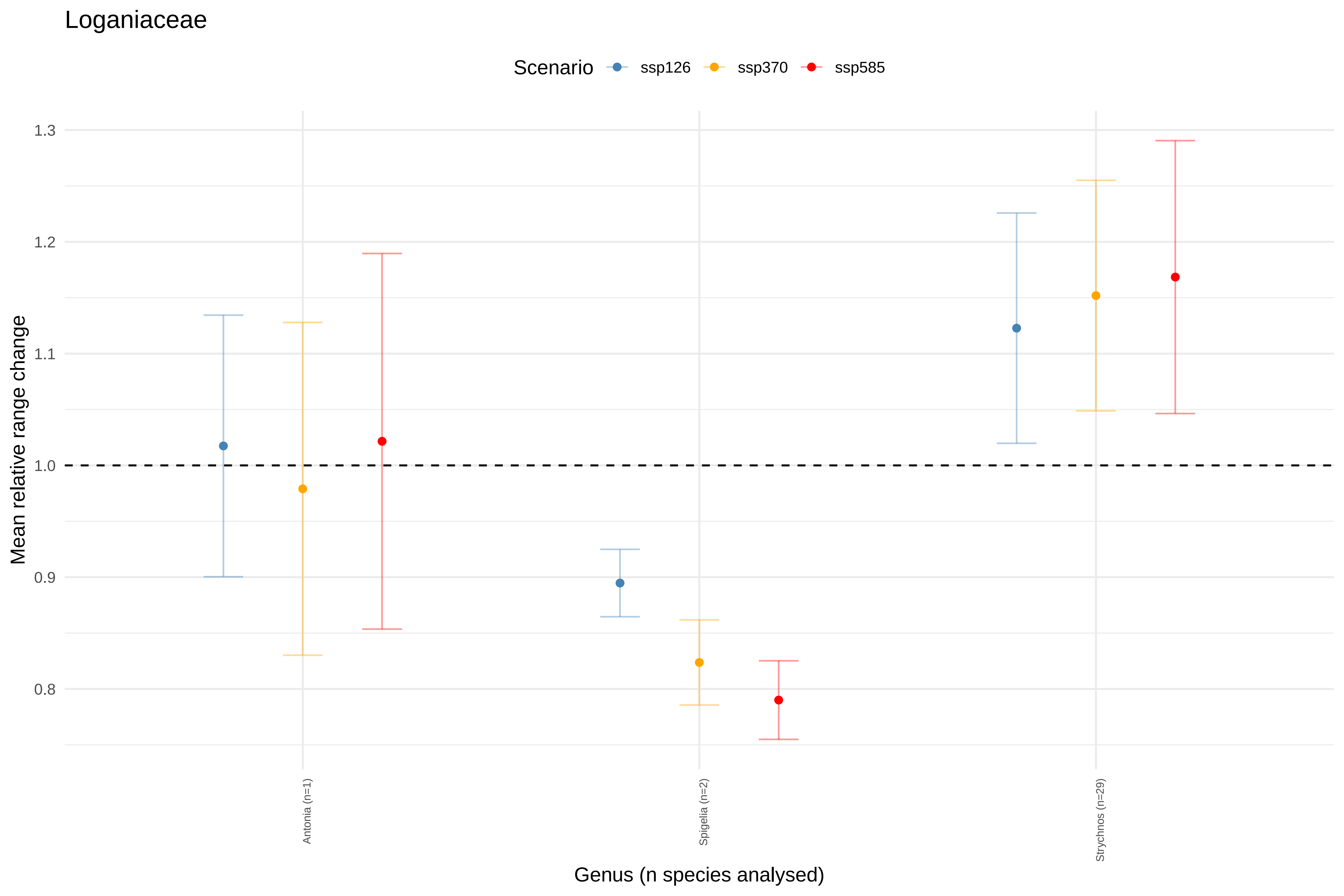

Loganiaceae
Scenario
ssp126
ssp370
ssp585
Mean relative range change
1.3
1.2
1.1
1.0
0.9
0.8
Antonia (n=1)
Spigelia (n=2)
Strychnos (n=29)
Genus (n species analysed)

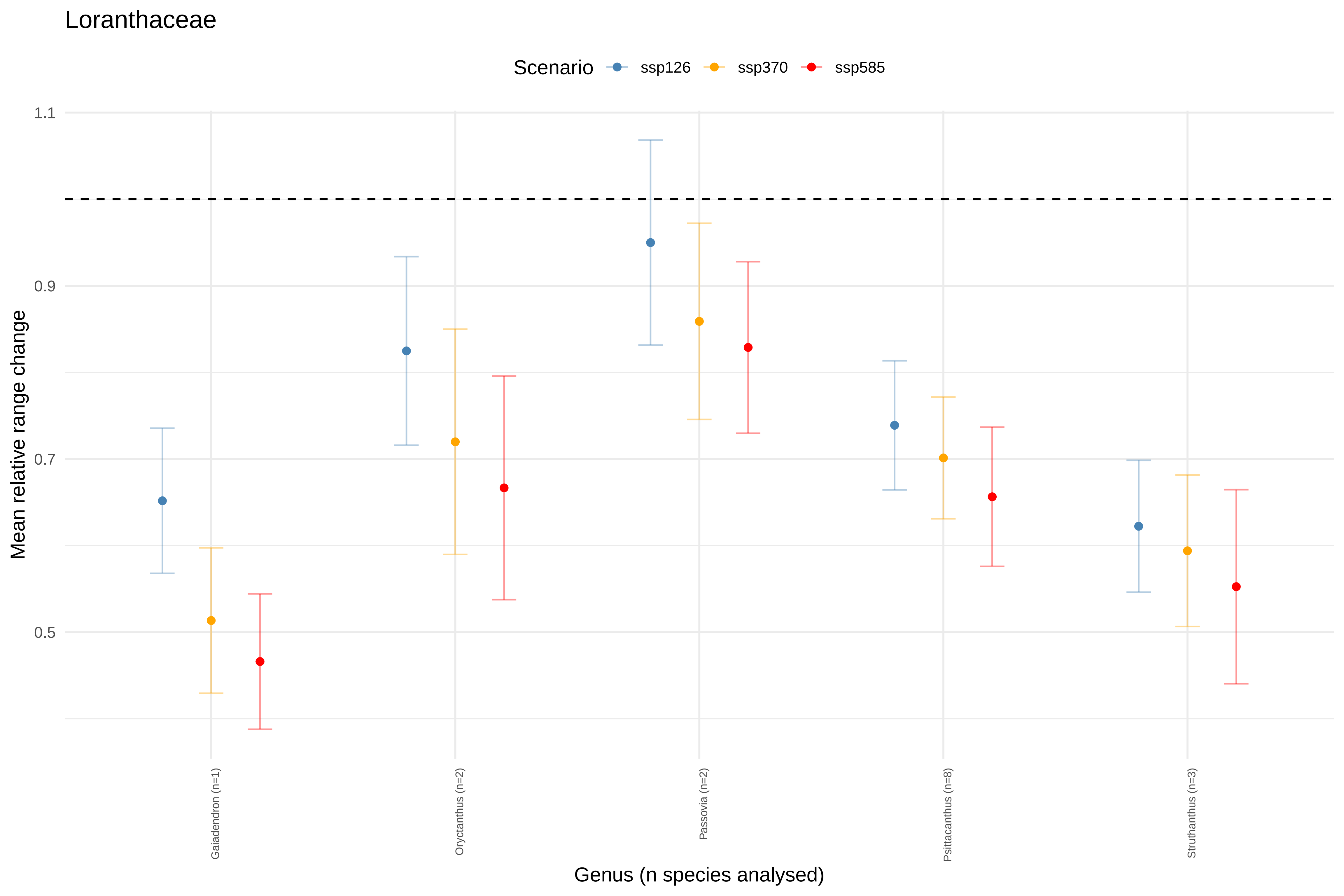

Loranthaceae
Scenario
ssp126
ssp370
ssp585
1.1
0.9
0.7
0.5
Mean relative range change
Gaiadendron (n=1)
Oryctanthus (n=2)
Passovia (n=2)
Psittacanthus (n=8)
Struthanthus (n=3)
Genus (n species analysed)

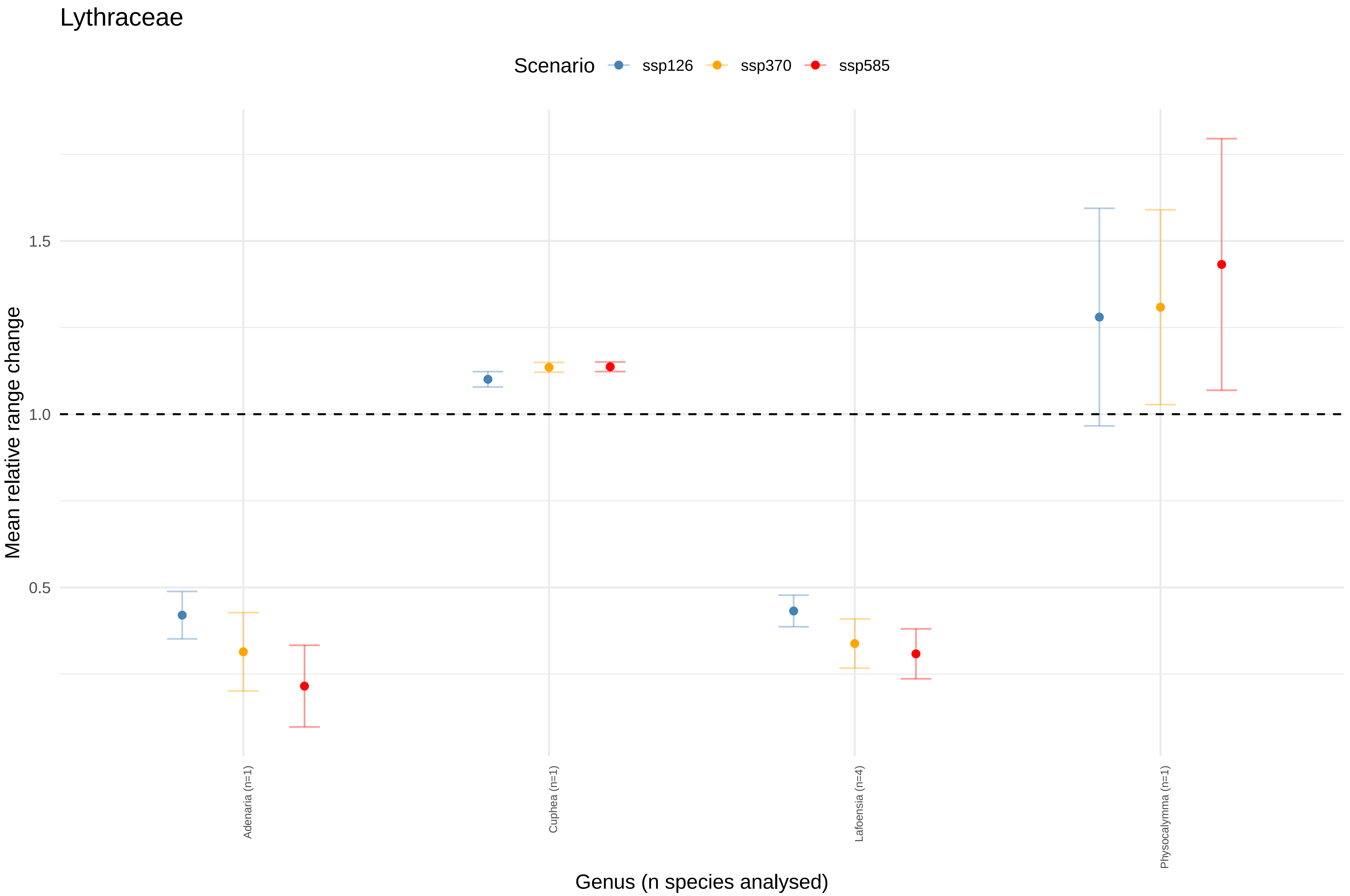

Lythraceae
Scenario
ssp126
ssp370
ssp585
Mean relative range change
1.5
1.0
0.5
Adenaria (n=1)
Cuphea (n=1)
Lafoensia (n=4)
Physocalymma (n=1)
Genus (n species analysed)

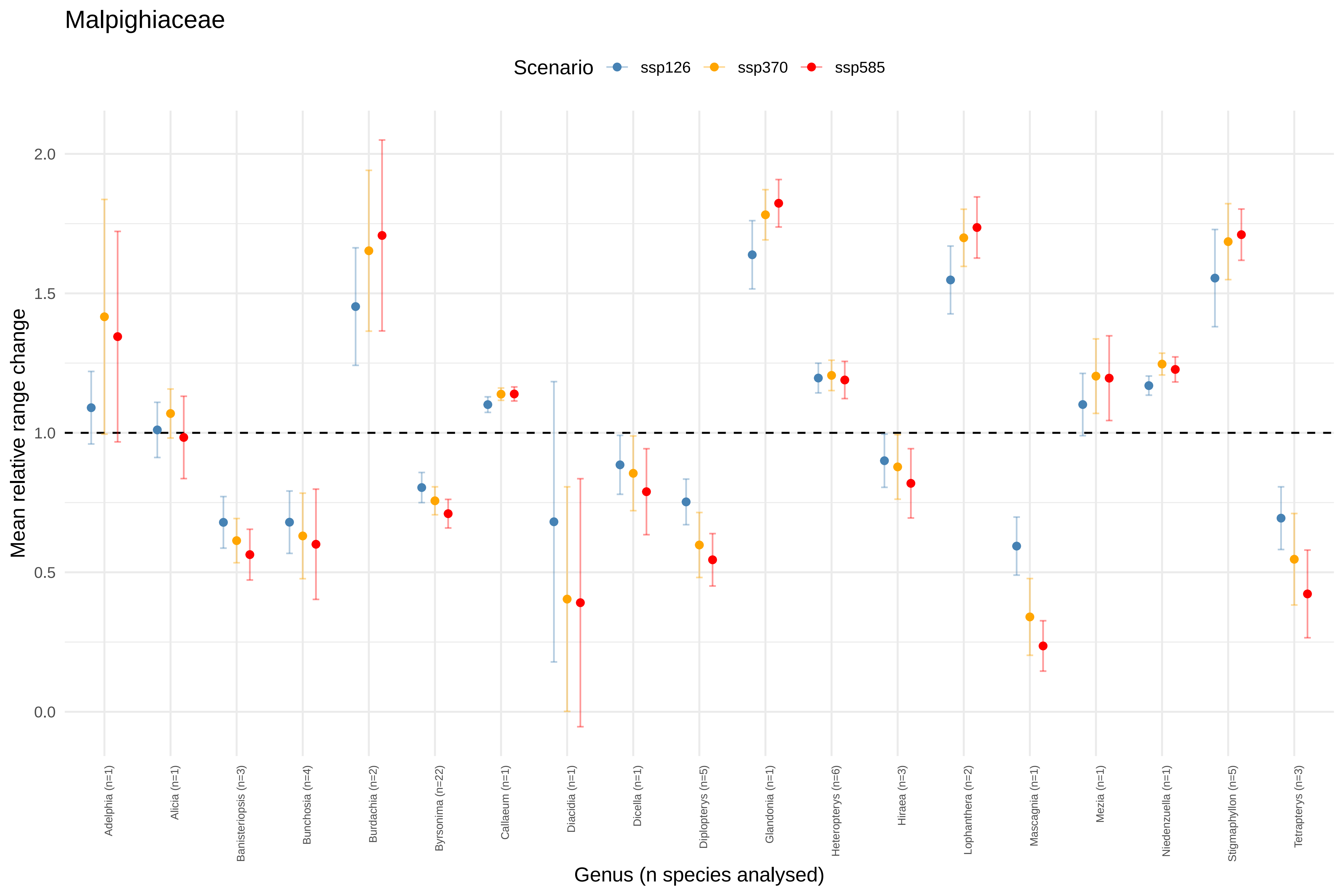

Malpighiaceae
Scenario
ssp126
ssp370
ssp585
Mean relative range change
0.0
0.5
1.0
1.5
2.0
Adelphia (n=1)
Alicia (n=1)
Banisteriopsis (n=3)
Bunchosia (n=4)
Burdachia (n=2)
Byrsonima (n=22)
Callaeum (n=1)
Diacidia (n=1)
Dicella (n=1)
Diplopterys (n=5)
Glandonia (n=1)
Heteropterys (n=6)
Hiraea (n=3)
Lophanthera (n=2)
Mascagnia (n=1)
Mezia (n=1)
Niedenzuella (n=1)
Stigmaphyllon (n=5)
Tetrapterys (n=3)
Genus (n species analysed)

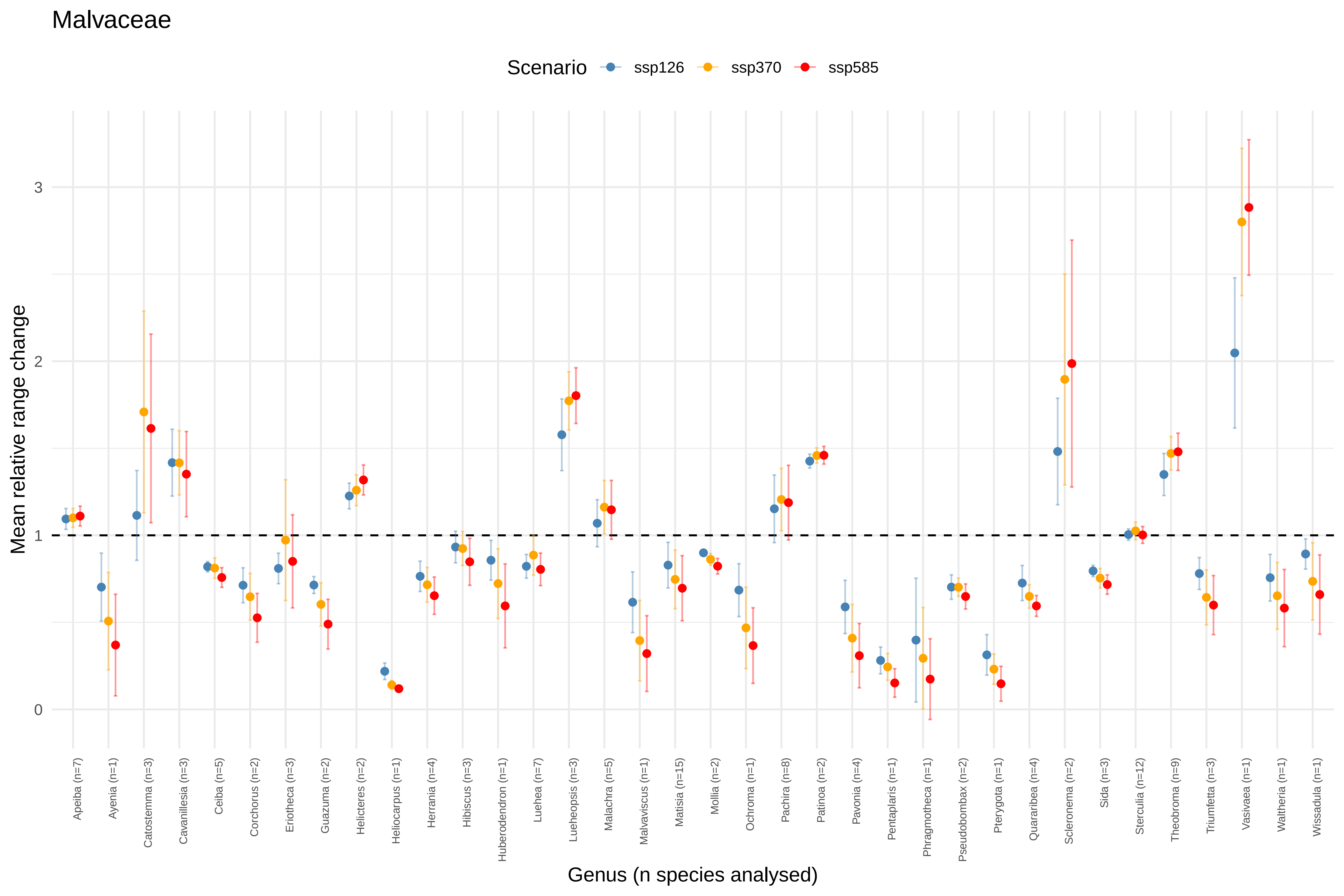

Malvaceae
Scenario
ssp126
ssp370
ssp585
Mean relative range change
0
1
2
3
Genus (n species analysed)
Apeiba (n=7)
Ayenia (n=1)
Catostemma (n=3)
Cavanillesia (n=3)
Ceiba (n=5)
Corchorus (n=2)
Eriotheca (n=3)
Guazuma (n=2)
Helicteres (n=2)
Heliocarpus (n=1)
Herrania (n=4)
Hibiscus (n=3)
Huberodendron (n=1)
Luehea (n=7)
Lueheopsis (n=3)
Malachra (n=5)
Malvaviscus (n=1)
Matisia (n=15)
Mollia (n=2)
Ochroma (n=1)
Pachira (n=8)
Patinoa (n=2)
Pavonia (n=4)
Pentaplaris (n=1)
Phragmotheca (n=1)
Pseudobombax (n=2)
Pterygota (n=1)
Quararibea (n=4)
Scleronema (n=2)
Sida (n=3)
Sterculia (n=12)
Theobroma (n=9)
Triumfetta (n=3)
Vasivaea (n=1)
Waltheria (n=1)
Wissadula (n=1)

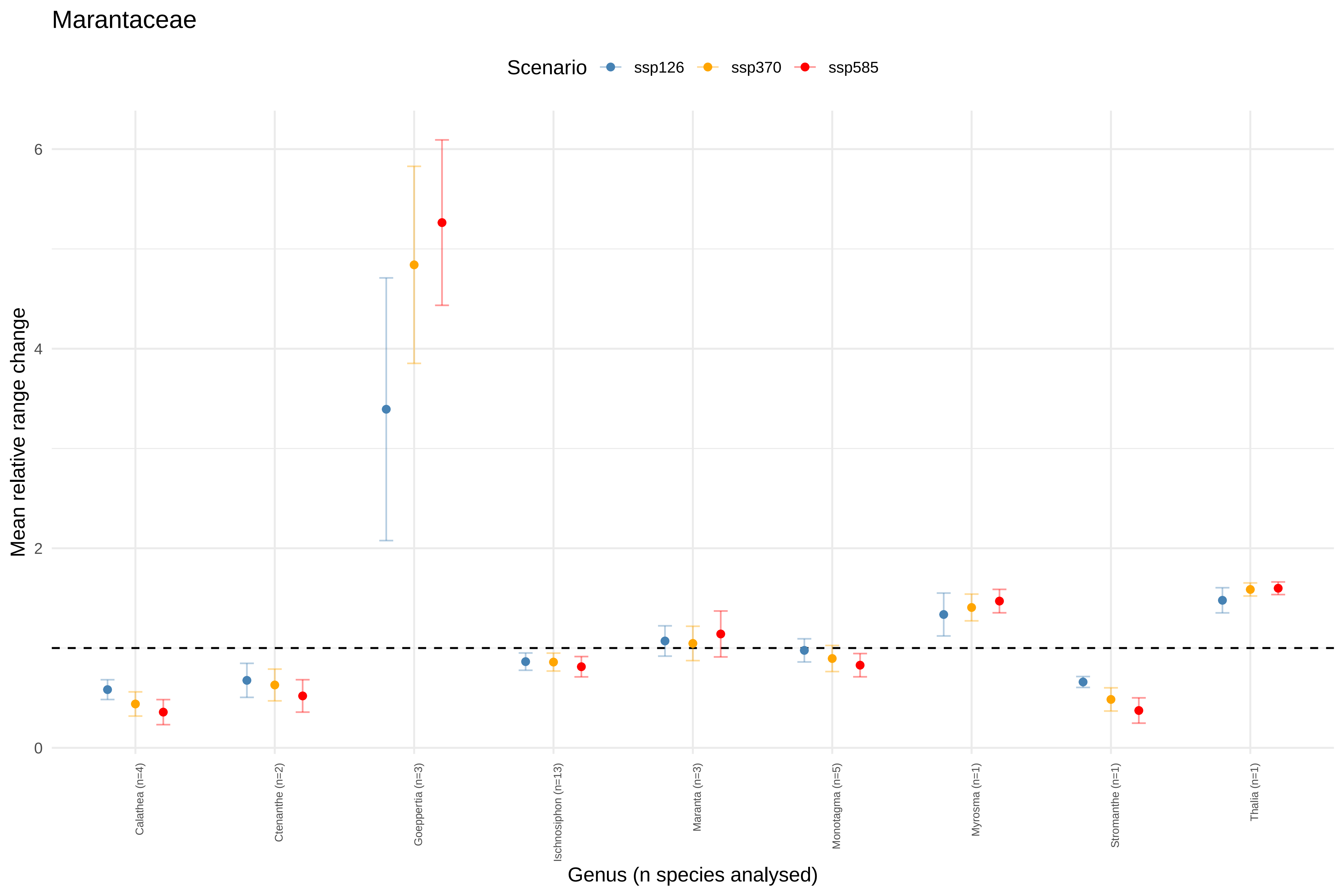

Marantaceae
Scenario
ssp126
ssp370
ssp585
Mean relative range change
0
2
4
6
Calathea (n=4)
Ctenanthe (n=2)
Goepperta (n=3)
Ischnosiphon (n=13)
Maranta (n=3)
Monotagma (n=5)
Myrosma (n=1)
Stromanthe (n=1)
Thalia (n=1)
Genus (n species analysed)

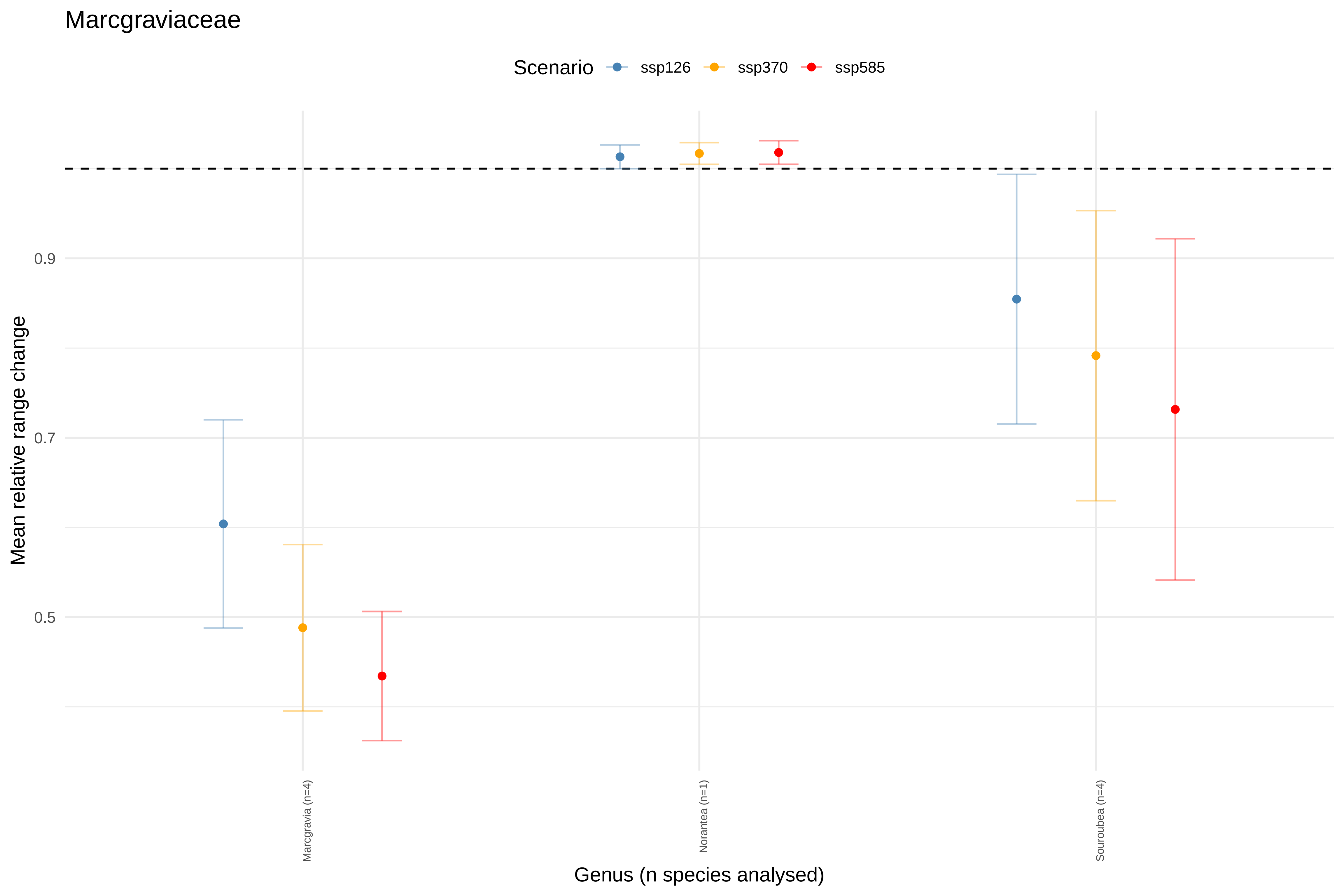
Marcgraviaceae
Scenario
ssp126
ssp370
ssp585
Mean relative range change
0.9
0.7
0.5
Marcgravia (n=4)
Norantea (n=1)
Souroubea (n=4)
Genus (n species analysed)

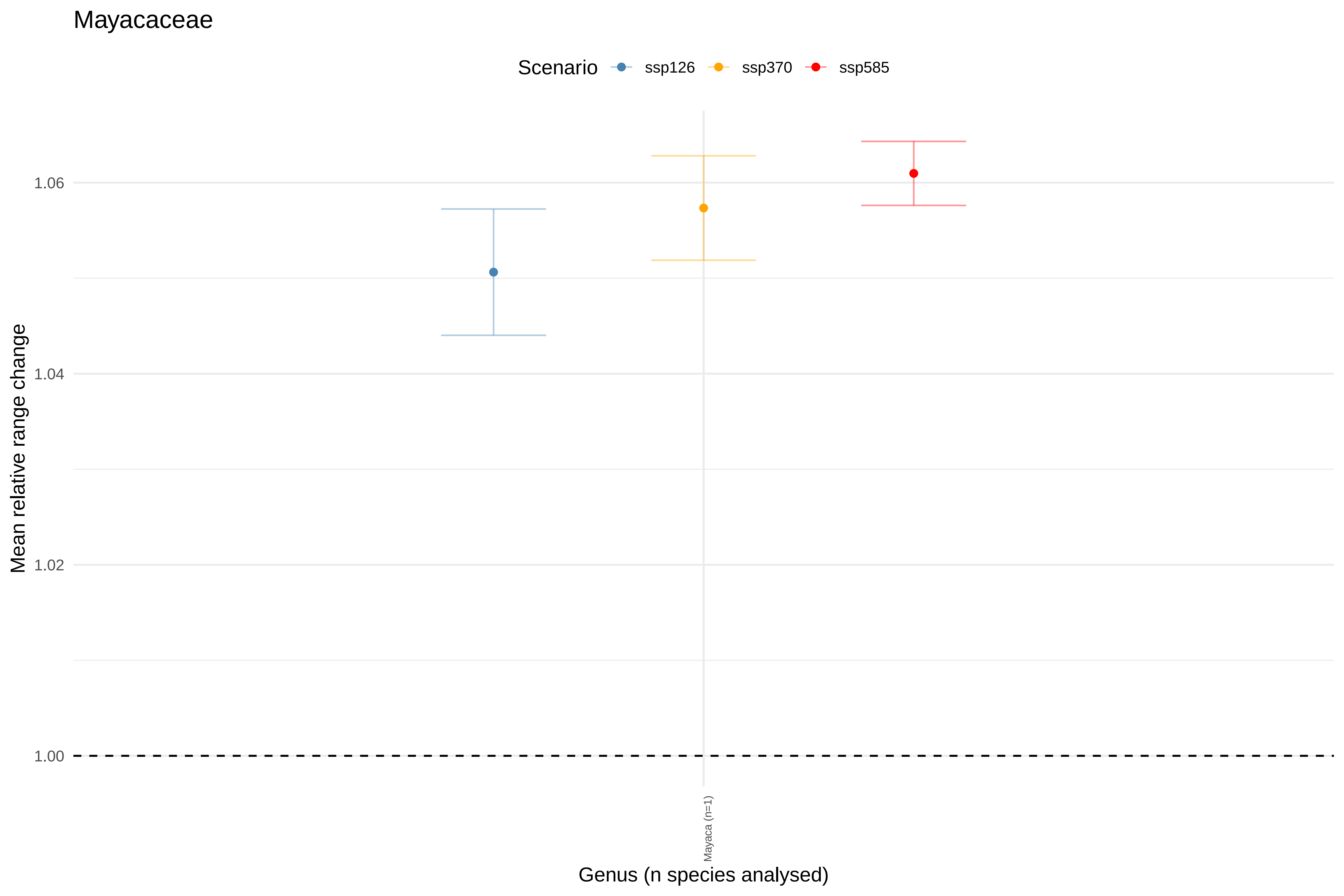

Mayacaceae
Scenario
ssp126
ssp370
ssp585
Mean relative range change
1.06
1.04
1.02
1.00
Mayaca (n=1)
Genus (n species analysed)

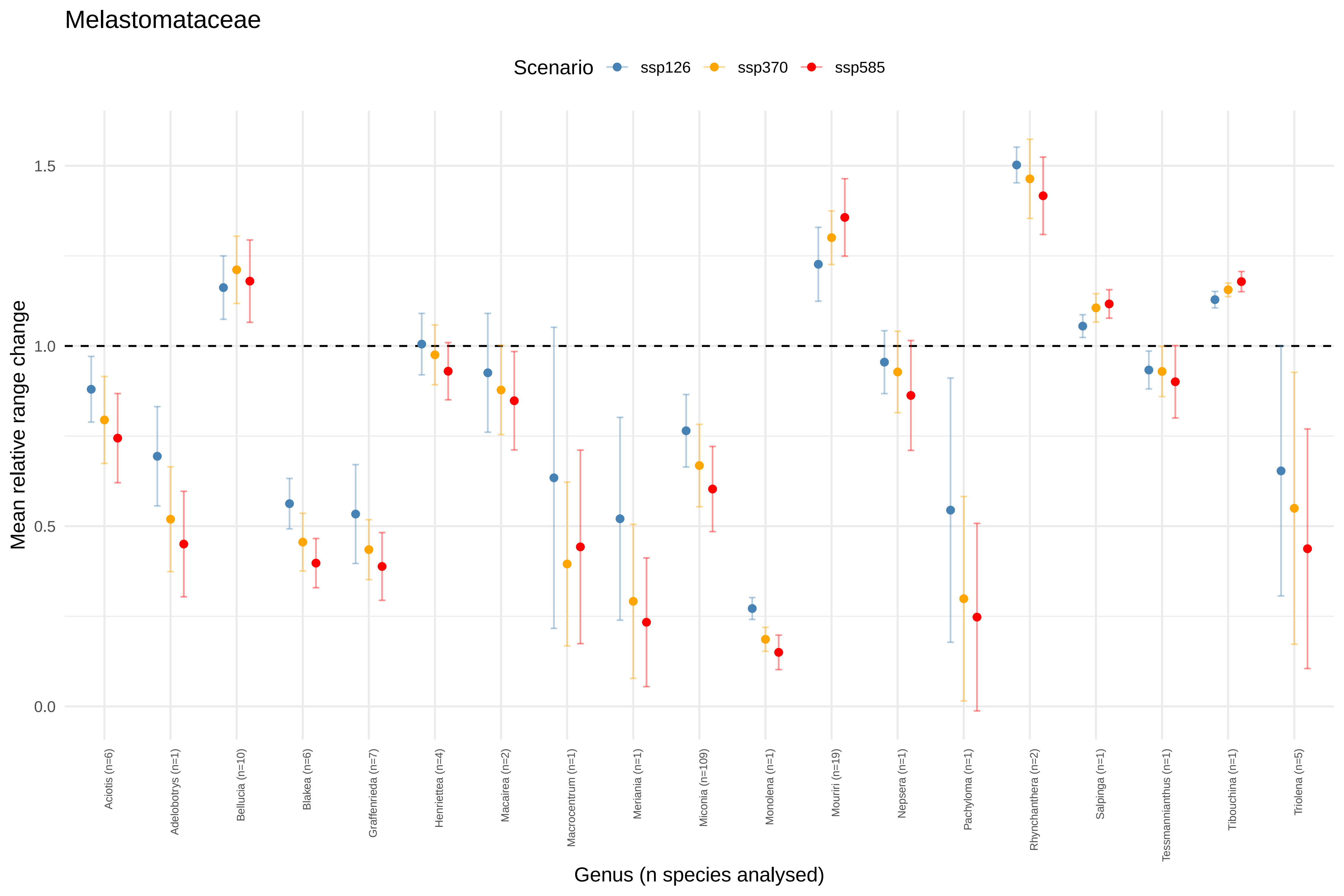
Melastomataceae
Scenario
ssp126
ssp370
ssp585
Mean relative range change
1.5
1.0
0.5
0.0
Aciotis (n=6)
Adelobotrys (n=1)
Bellucia (n=10)
Blakea (n=6)
Graffenrieda (n=7)
Henriettea (n=4)
Macairea (n=2)
Macrocentrum (n=1)
Meriania (n=1)
Miconia (n=109)
Monolena (n=1)
Mouriri (n=19)
Nepsera (n=1)
Pachyloma (n=1)
Rhynchanthera (n=2)
Salpinga (n=1)
Tessmannianthus (n=1)
Tibouchina (n=1)
Triolena (n=5)
Genus (n species analysed)

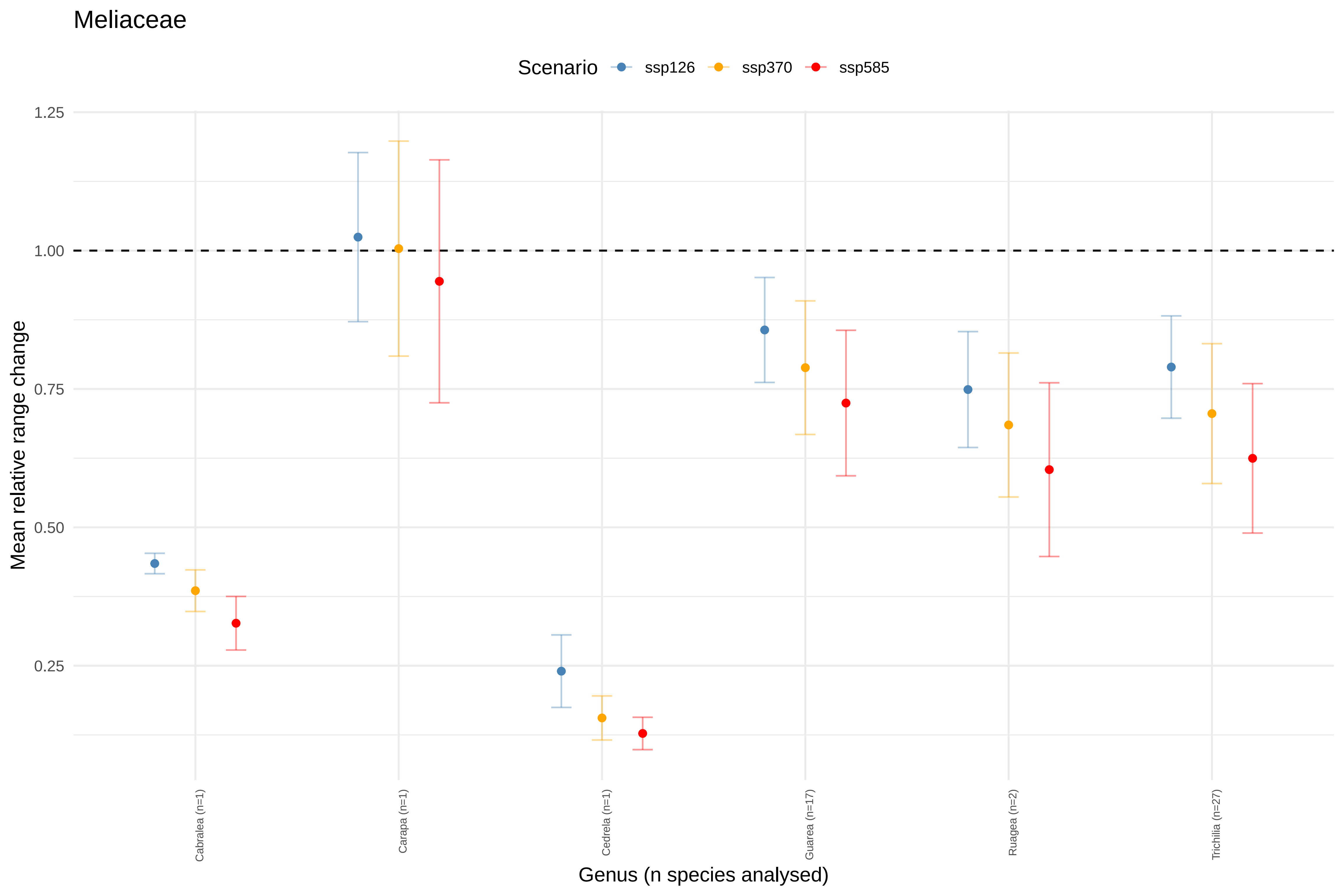

Meliaceae
Scenario
ssp126
ssp370
ssp585
1.25
1.00
0.75
0.50
0.25
Mean relative range change
Cabralea (n=1)
Carapa (n=1)
Cedrela (n=1)
Guarea (n=17)
Ruagea (n=2)
Trichilia (n=27)
Genus (n species analysed)

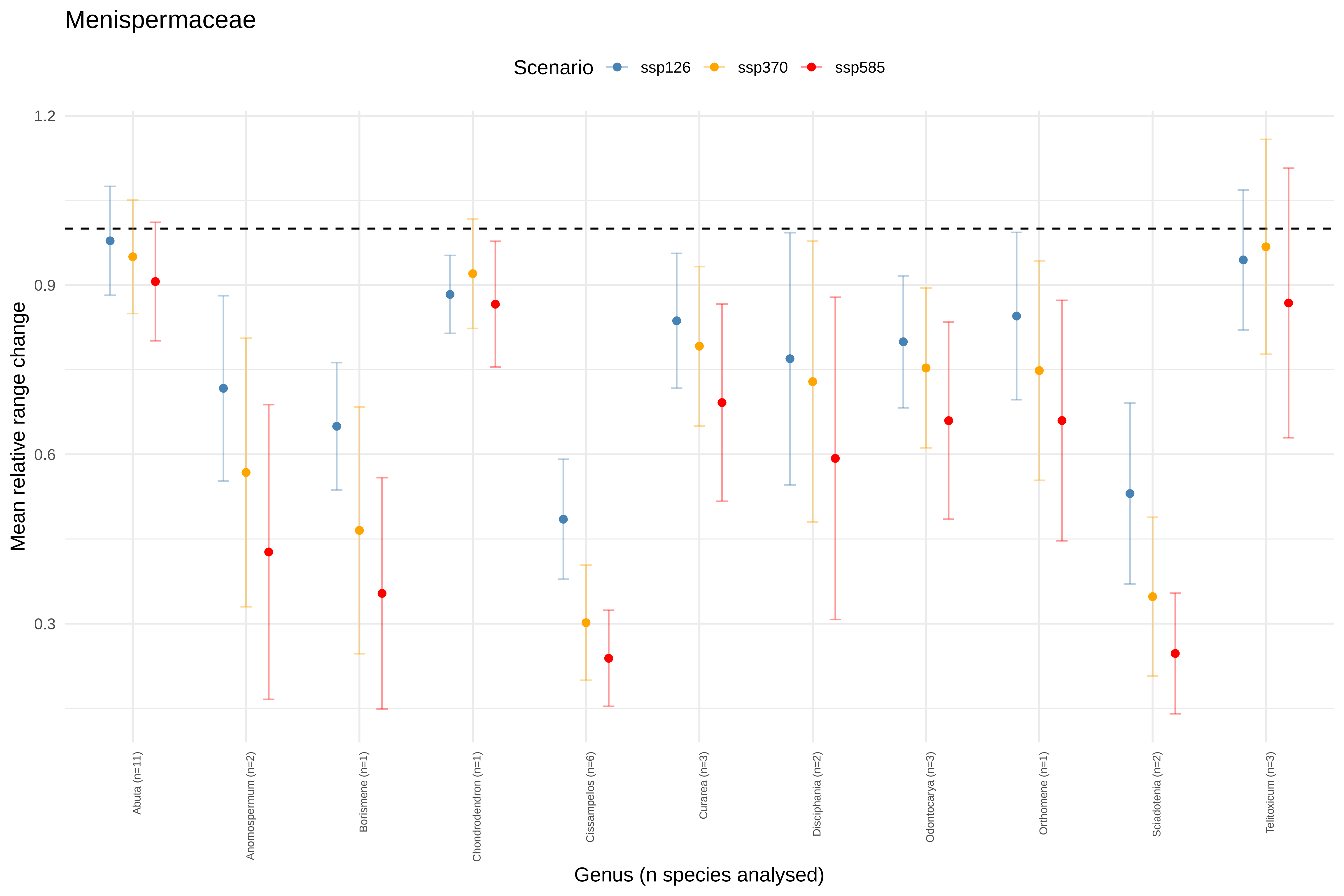

Menispermaceae
Scenario
ssp126
ssp370
ssp585
1.2
0.9
0.6
0.3
Mean relative range change
Abuta (n=11)
Anomospermum (n=2)
Borismene (n=1)
Chondrodendron (n=1)
Cissampelos (n=6)
Curarea (n=3)
Disciphania (n=2)
Odontocarya (n=3)
Orthomene (n=1)
Sciadotenia (n=2)
Telitoxicum (n=3)
Genus (n species analysed)

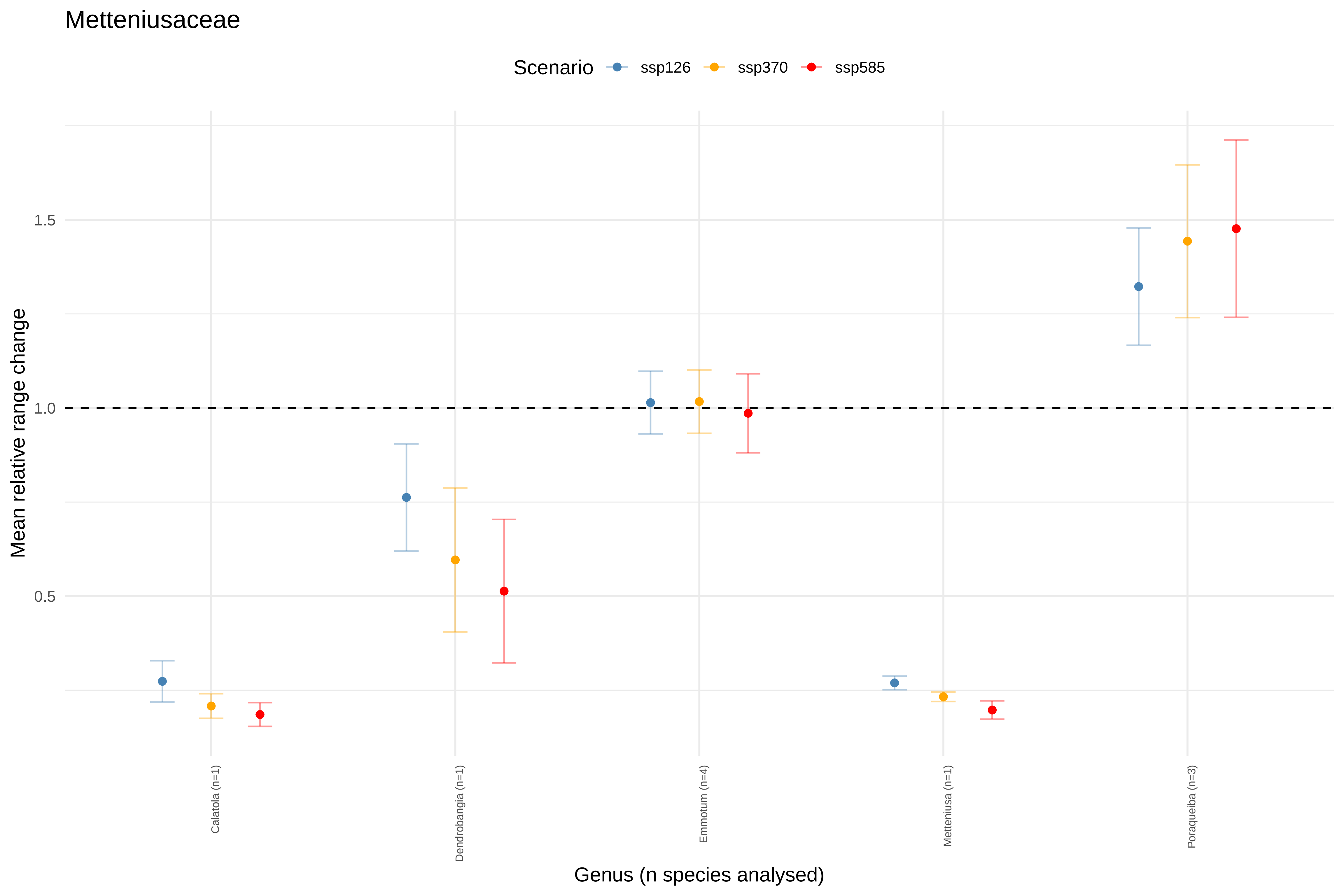
Metteniusaceae
Scenario
ssp126
ssp370
ssp585
Mean relative range change
1.5
1.0
0.5
Calatola (n=1)
Dendrobangia (n=1)
Emmotum (n=4)
Metteniusa (n=1)
Poraqueiba (n=3)
Genus (n species analysed)

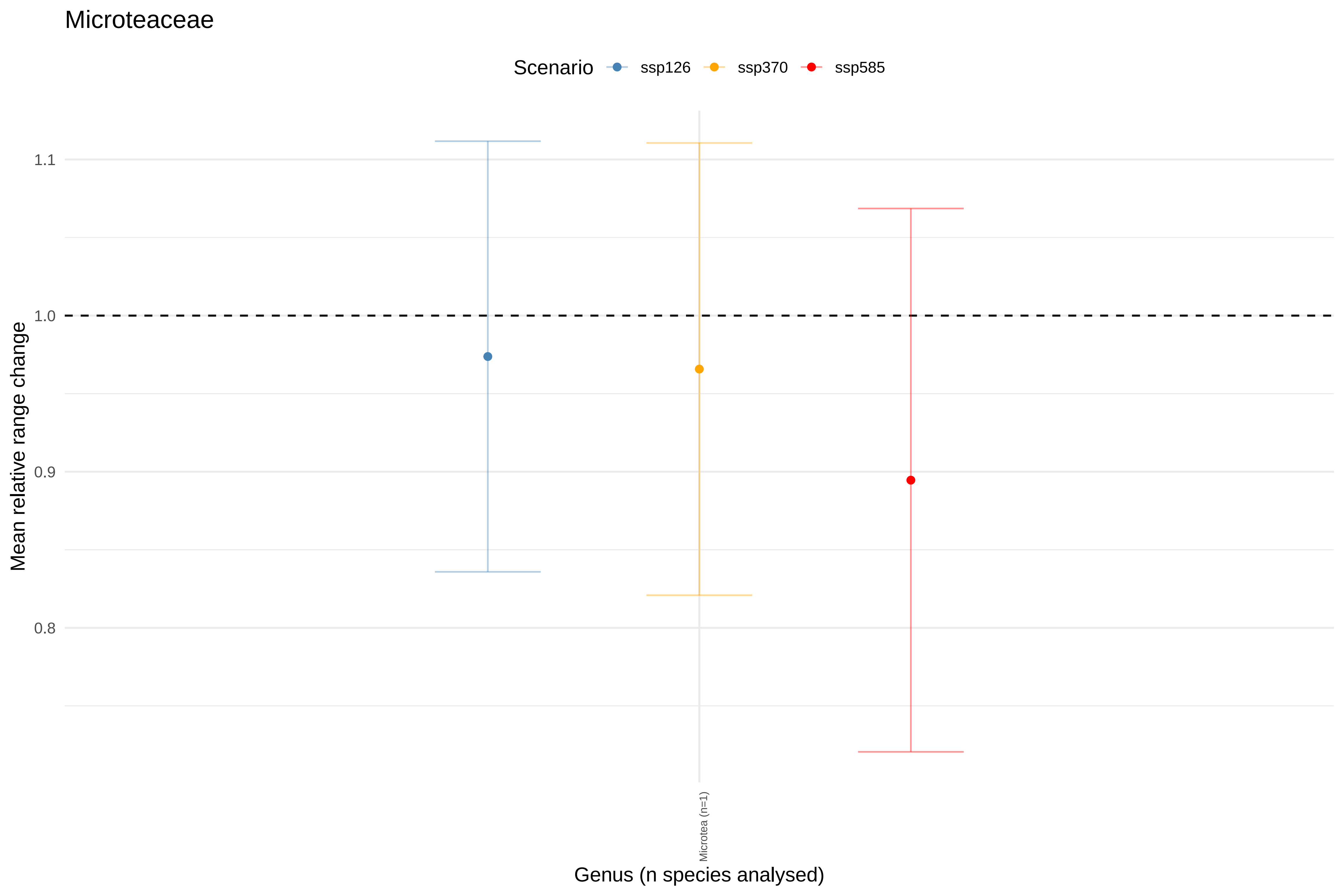

Microteaceae
Scenario
ssp126
ssp370
ssp585
1.1
1.0
0.9
0.8
Mean relative range change
Microtea (n=1)
Genus (n species analysed)

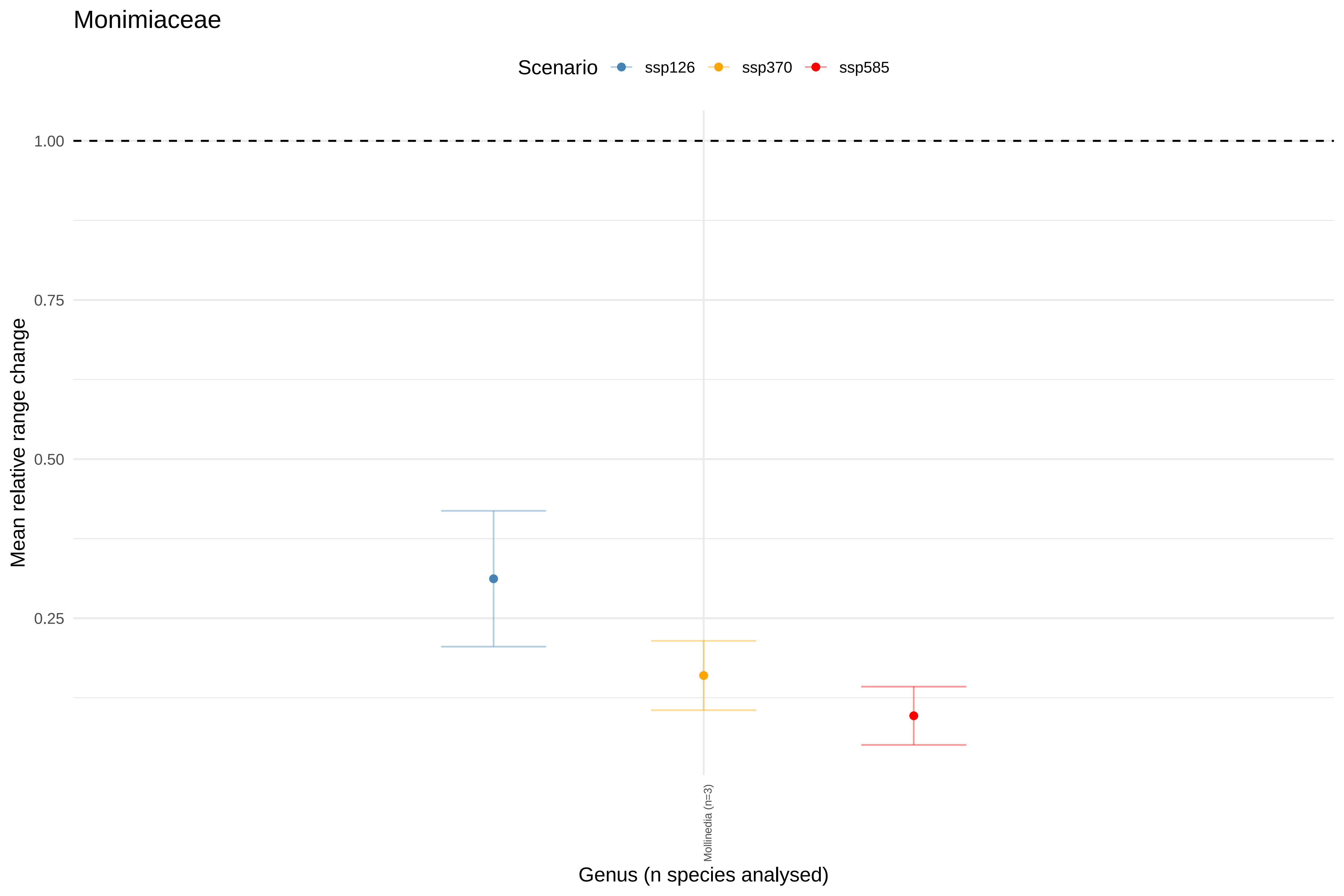

Monimiaceae
Scenario
ssp126
ssp370
ssp585
1.00
0.75
0.50
0.25
Mean relative range change
Mollinedia (n=3)
Genus (n species analysed)

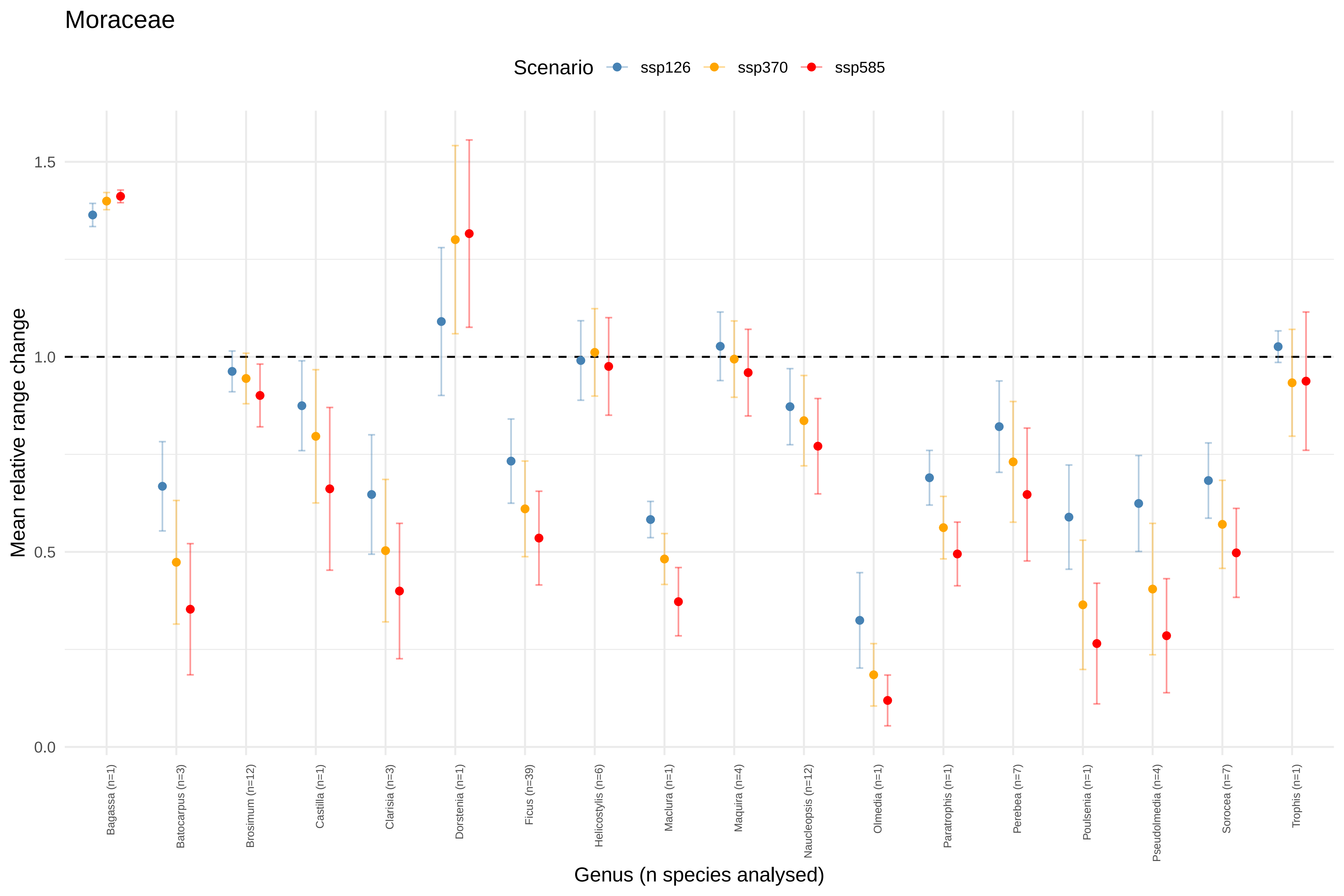

Moraceae
Scenario
ssp126
ssp370
ssp585
Mean relative range change
0.0
0.5
1.0
1.5
Bagassa (n=1)
Batocarpus (n=3)
Brosimum (n=12)
Castilla (n=1)
Clarisia (n=3)
Dorstenia (n=1)
Ficus (n=39)
Helicostylis (n=6)
Maclura (n=1)
Maquira (n=4)
Naucleopsis (n=12)
Olmedia (n=1)
Paratrophis (n=1)
Perebea (n=7)
Poulsenia (n=1)
Pseudolmedia (n=4)
Sorocea (n=7)
Trophis (n=1)
Genus (n species analysed)

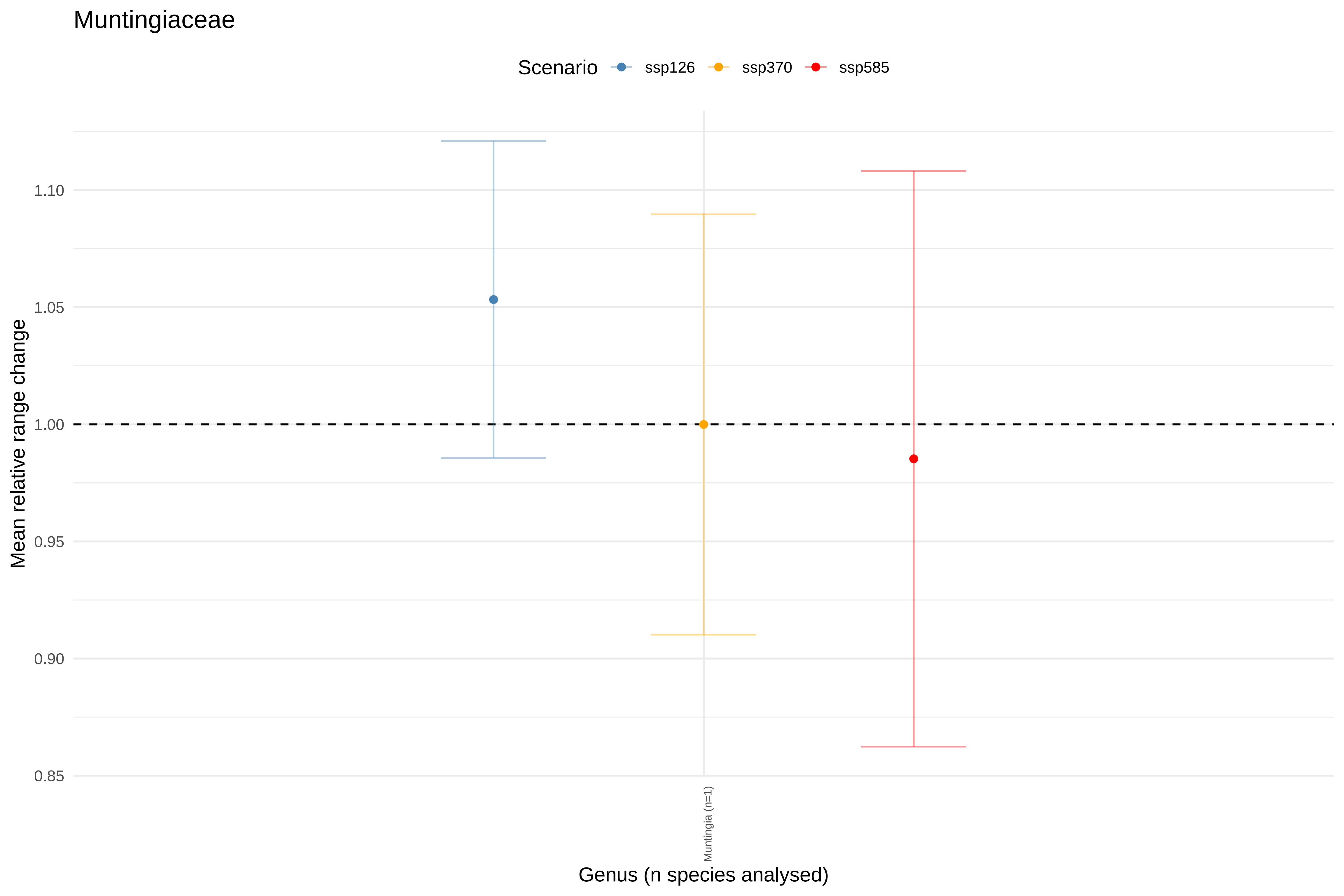

Muntingiaceae
Scenario
ssp126
ssp370
ssp585
Mean relative range change
1.10
1.05
1.00
0.95
0.90
0.85
Muntingia (n=1)
Genus (n species analysed)

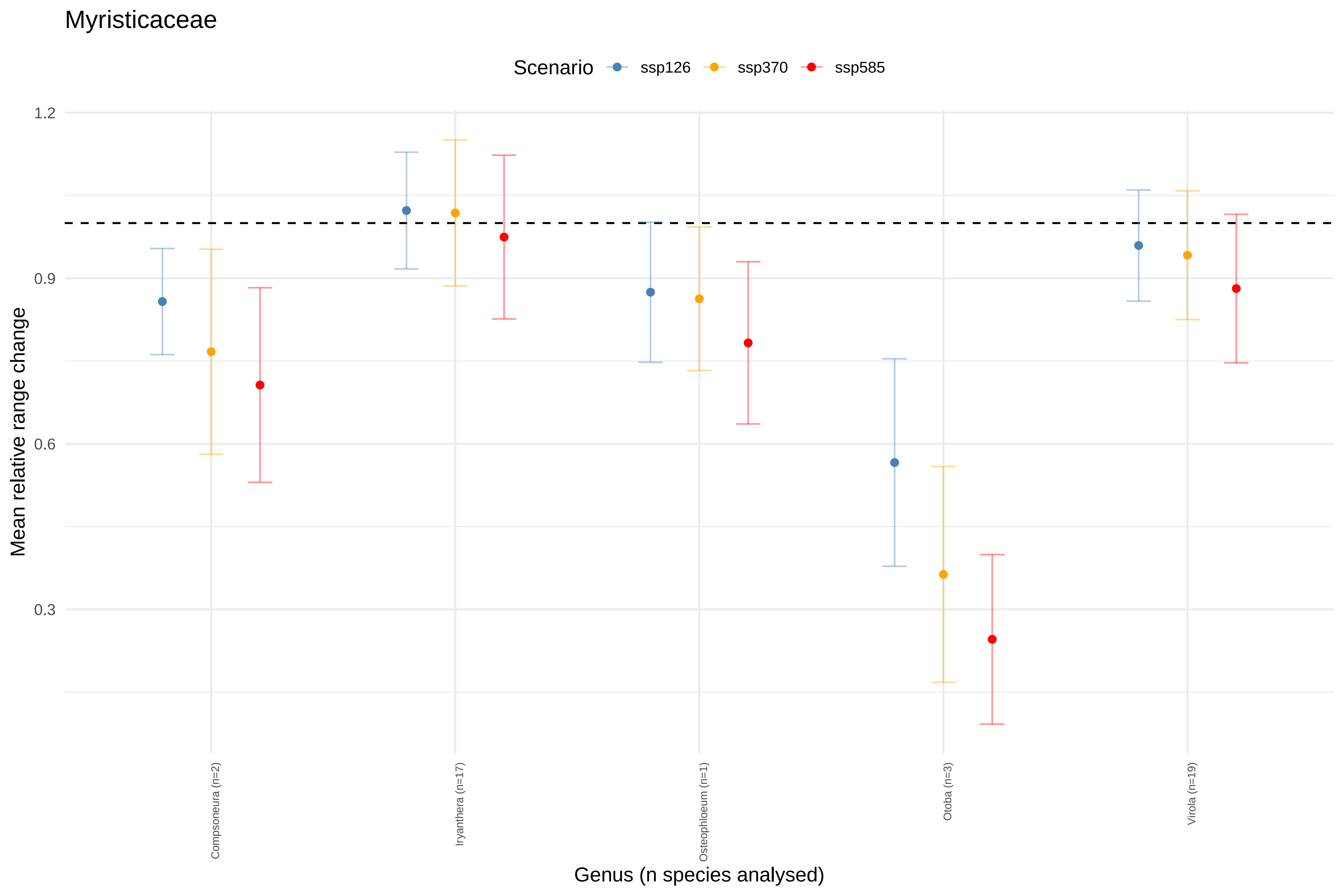

Myristicaceae
Scenario
ssp126
ssp370
ssp585
1.2
0.9
0.6
0.3
Mean relative range change
Compsoneura (n=2)
Iryanthera (n=17)
Osteophloeum (n=1)
Otoba (n=3)
Virola (n=19)
Genus (n species analysed)

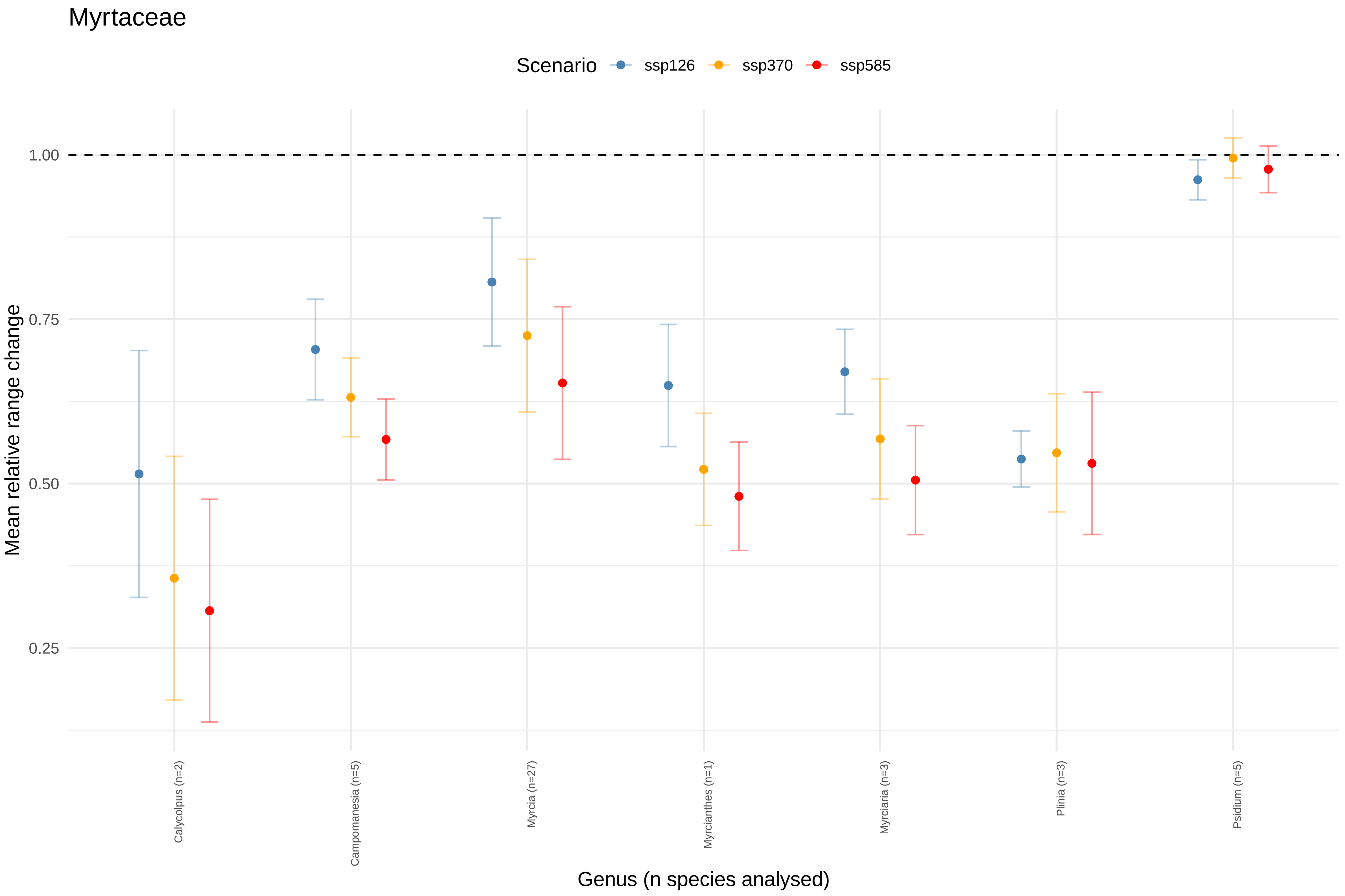

Myrtaceae
Scenario
ssp126
ssp370
ssp585
1.00
0.75
0.50
0.25
Mean relative range change
Calycolpus (n=2)
Campomanesia (n=5)
Myrcia (n=27)
Myrcianthes (n=1)
Myrciaria (n=3)
Plinia (n=3)
Psidium (n=5)
Genus (n species analysed)

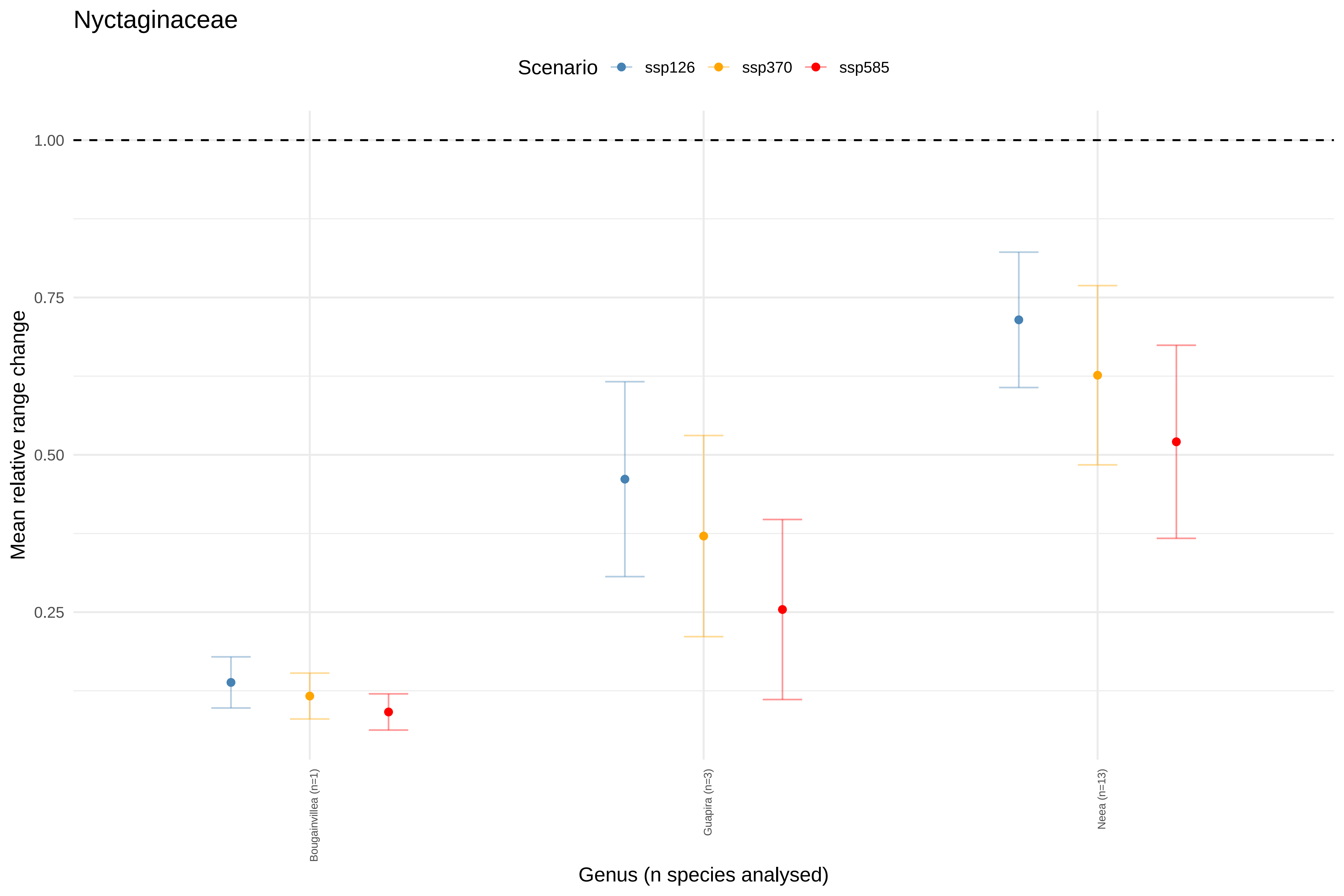

Nyctaginaceae
Scenario
ssp126
ssp370
ssp585
Mean relative range change
1.00
0.75
0.50
0.25
Bougainvillea (n=1)
Guapira (n=3)
Neea (n=13)
Genus (n species analysed)

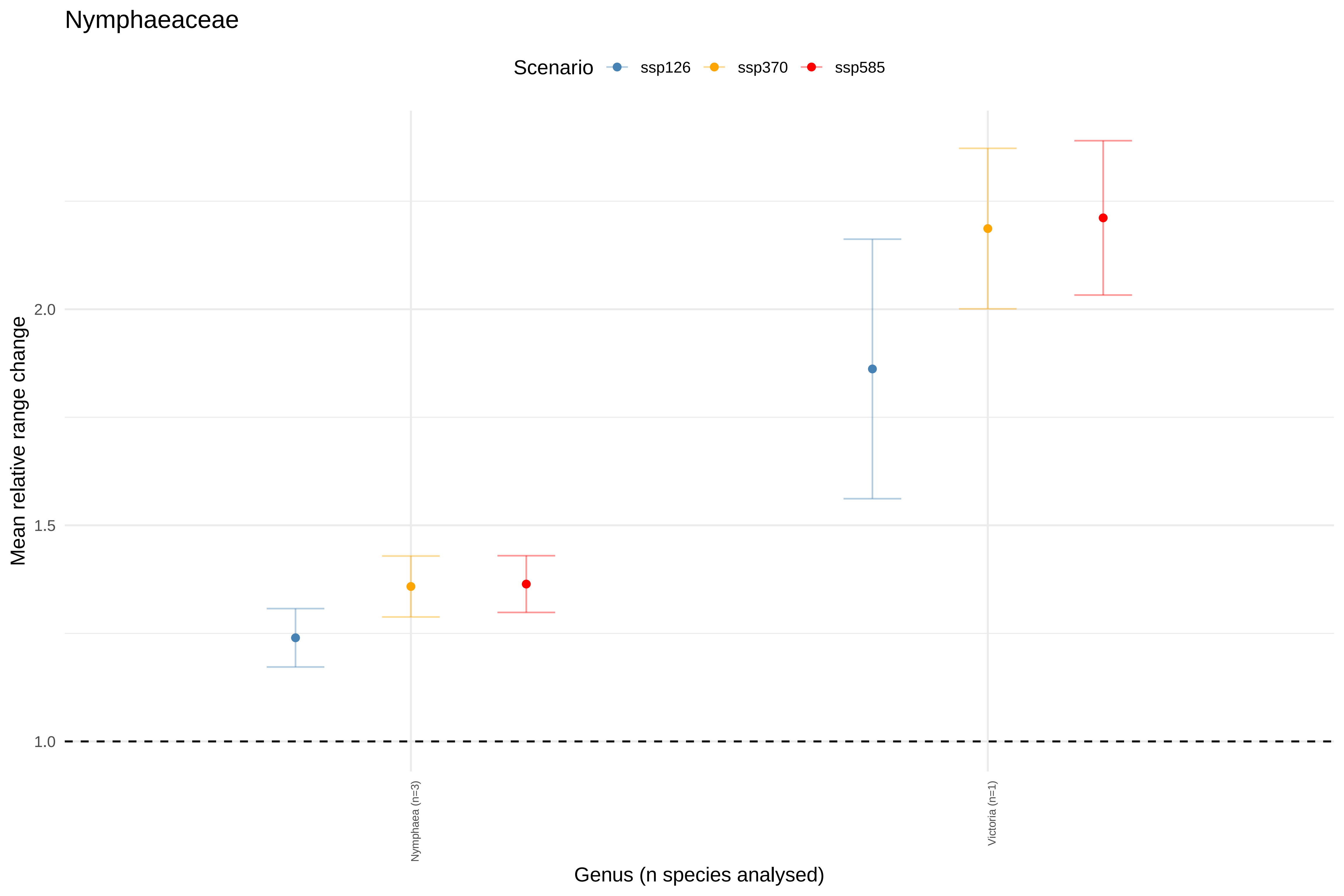

Nymphaeaceae
Scenario
ssp126
ssp370
ssp585
Mean relative range change
2.0
1.5
1.0
Nymphaea (n=3)
Victoria (n=1)
Genus (n species analysed)

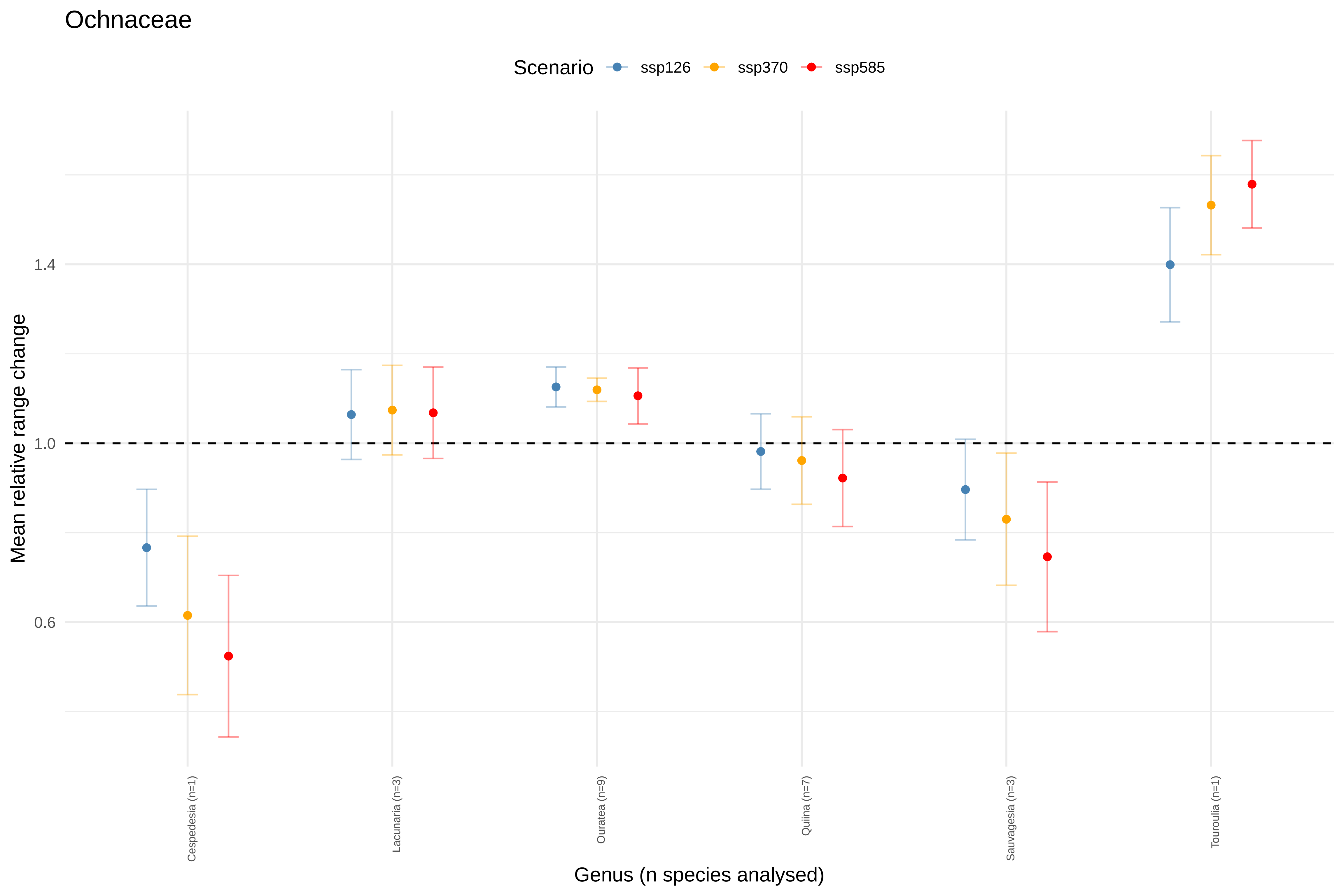

Ochnaceae
Scenario
ssp126
ssp370
ssp585
Mean relative range change
1.4
1.0
0.6
Cespedesia (n=1)
Lacunaria (n=3)
Ouratea (n=9)
Quiina (n=7)
Sauvagesia (n=3)
Touroulia (n=1)
Genus (n species analysed)

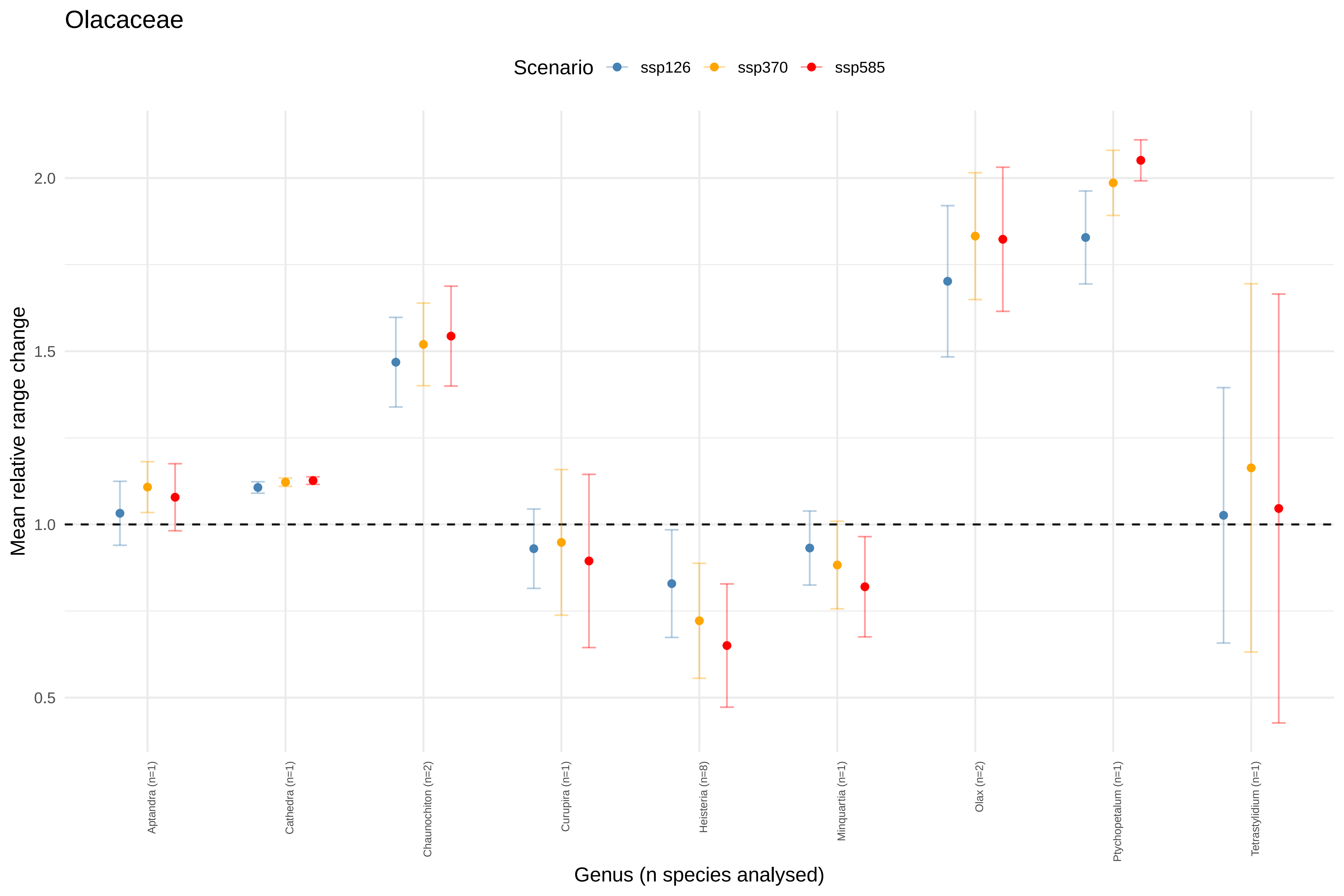

Olacaceae
Scenario
ssp126
ssp370
ssp585
Mean relative range change
2.0
1.5
1.0
0.5
Aptandra (n=1)
Cathedra (n=1)
Chaunochiton (n=2)
Curupira (n=1)
Heisteria (n=8)
Minquartia (n=1)
Olax (n=2)
Ptychopetalum (n=1)
Tetrastylidium (n=1)
Genus (n species analysed)

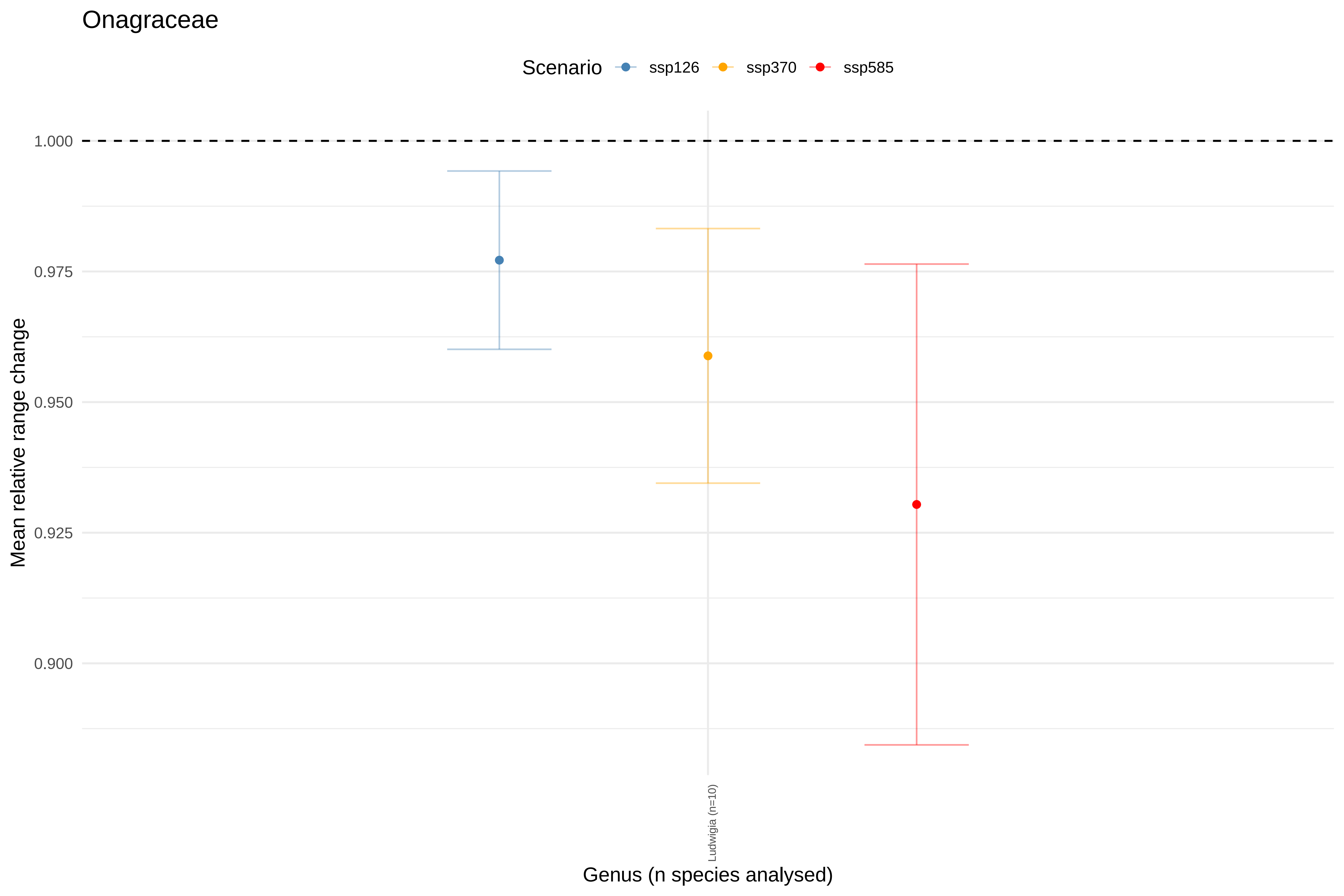
Onagraceae
Scenario
ssp126
ssp370
ssp585
1.000
0.975
0.950
0.925
0.900
Mean relative range change
Ludwigia (n=10)
Genus (n species analysed)

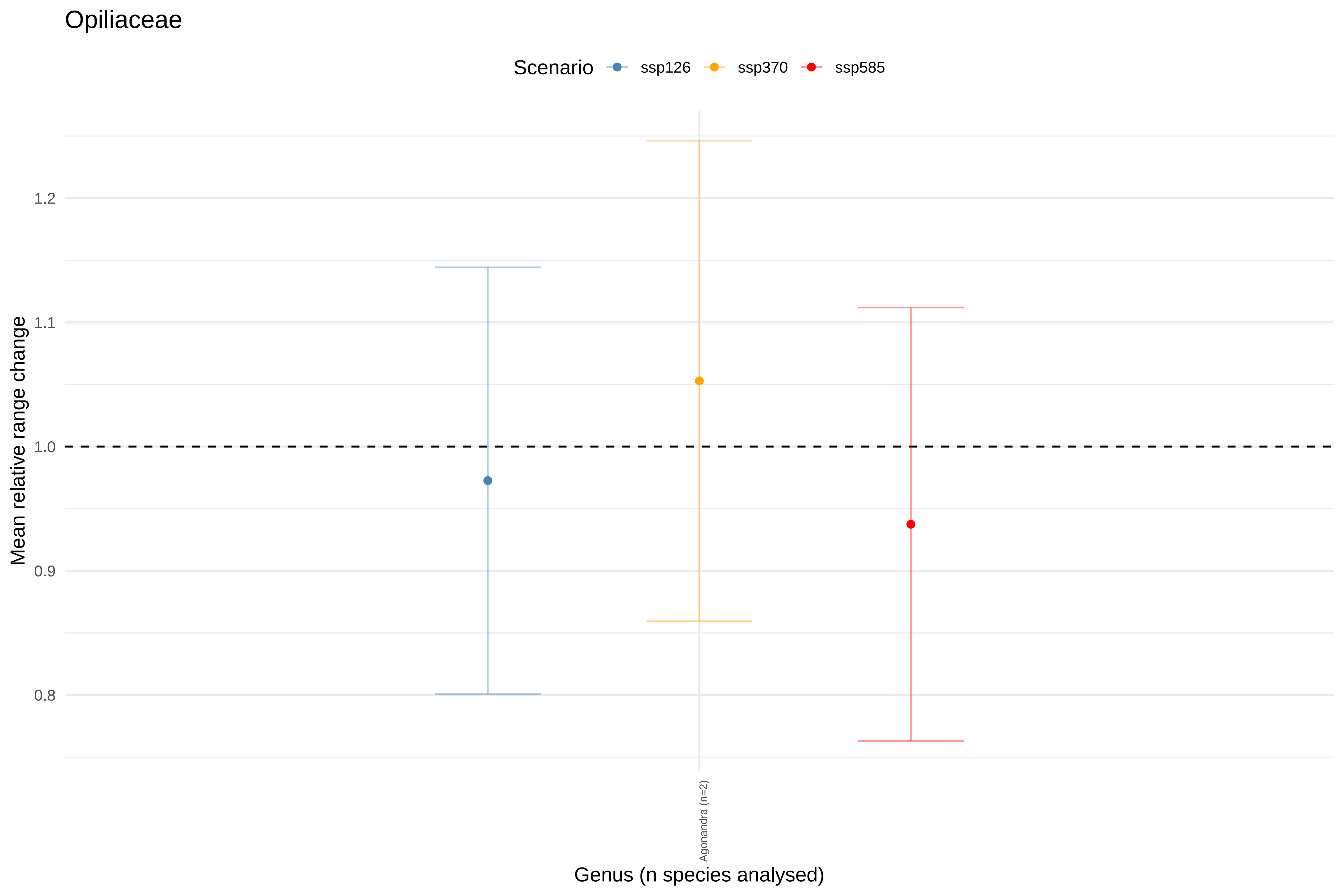
Opiliaceae
Scenario
ssp126
ssp370
ssp585
Mean relative range change
1.2
1.1
1.0
0.9
0.8
Agonandra (n=2)
Genus (n species analysed)

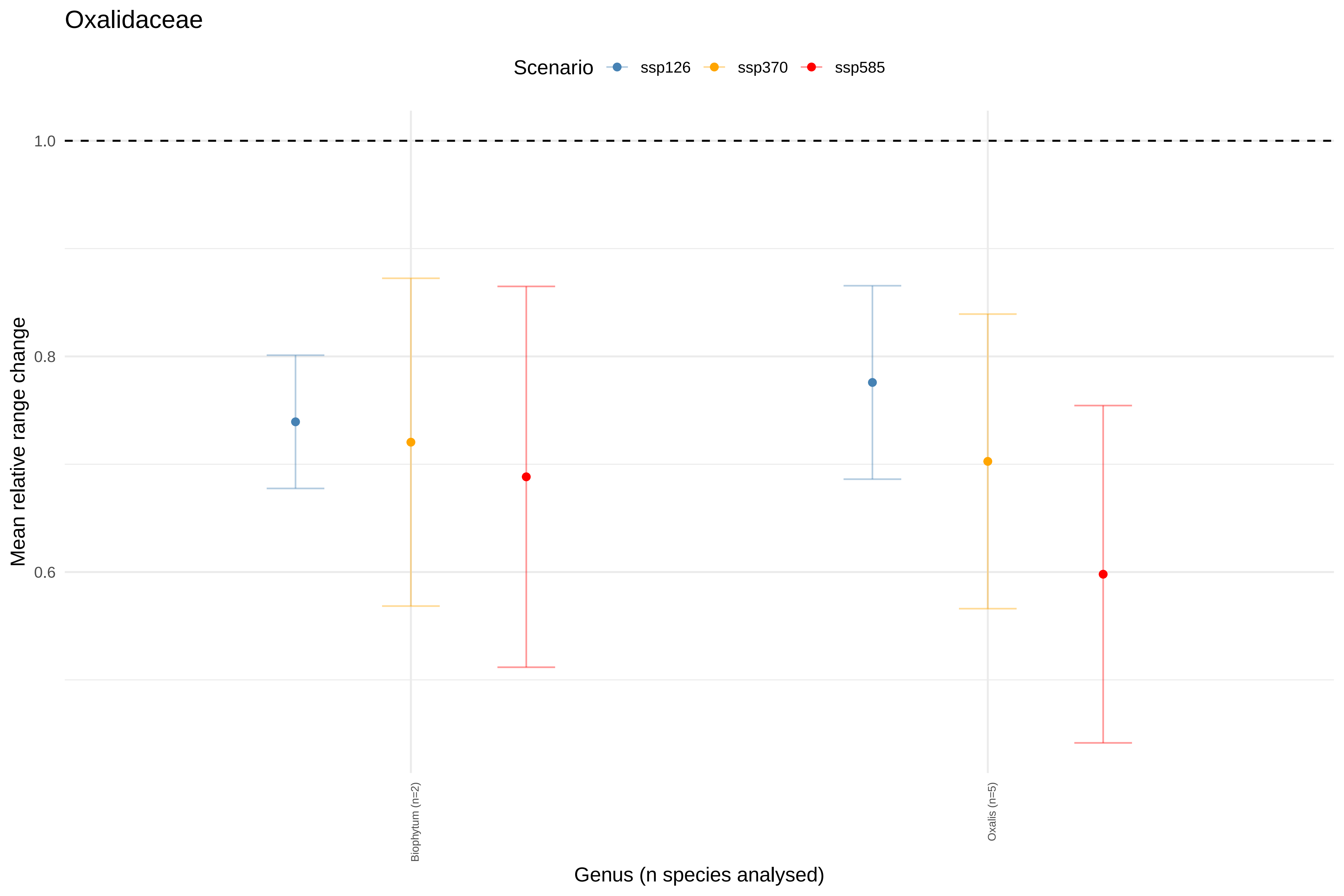

Oxalidaceae
Scenario
ssp126
ssp370
ssp585
1.0
0.8
0.6
Mean relative range change
Biophytum (n=2)
Oxalis (n=5)
Genus (n species analysed)

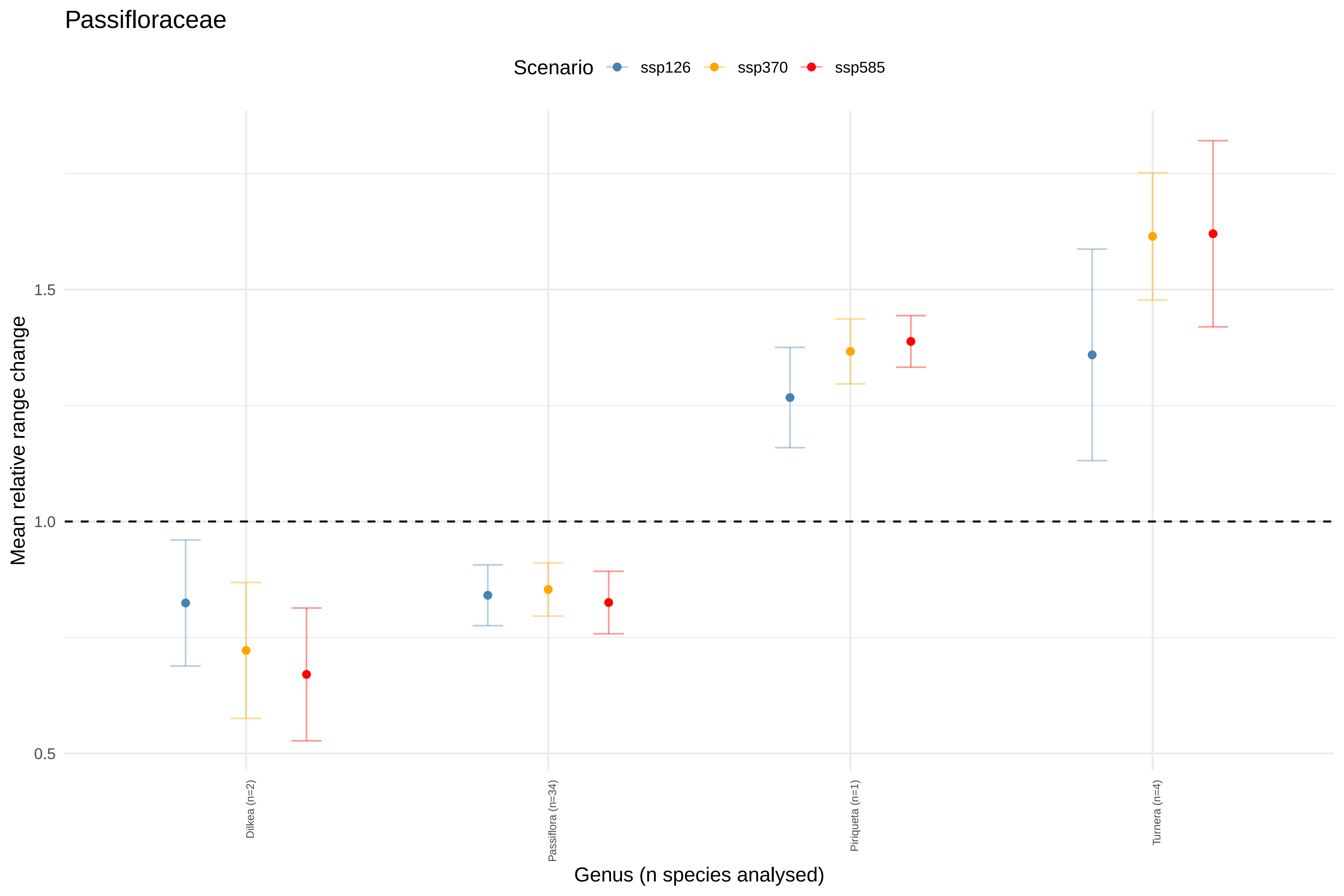

Passifloraceae
Scenario
ssp126
ssp370
ssp585
Mean relative range change
1.5
1.0
0.5
Dilkea (n=2)
Passiflora (n=34)
Piriqueta (n=1)
Turnera (n=4)
Genus (n species analysed)

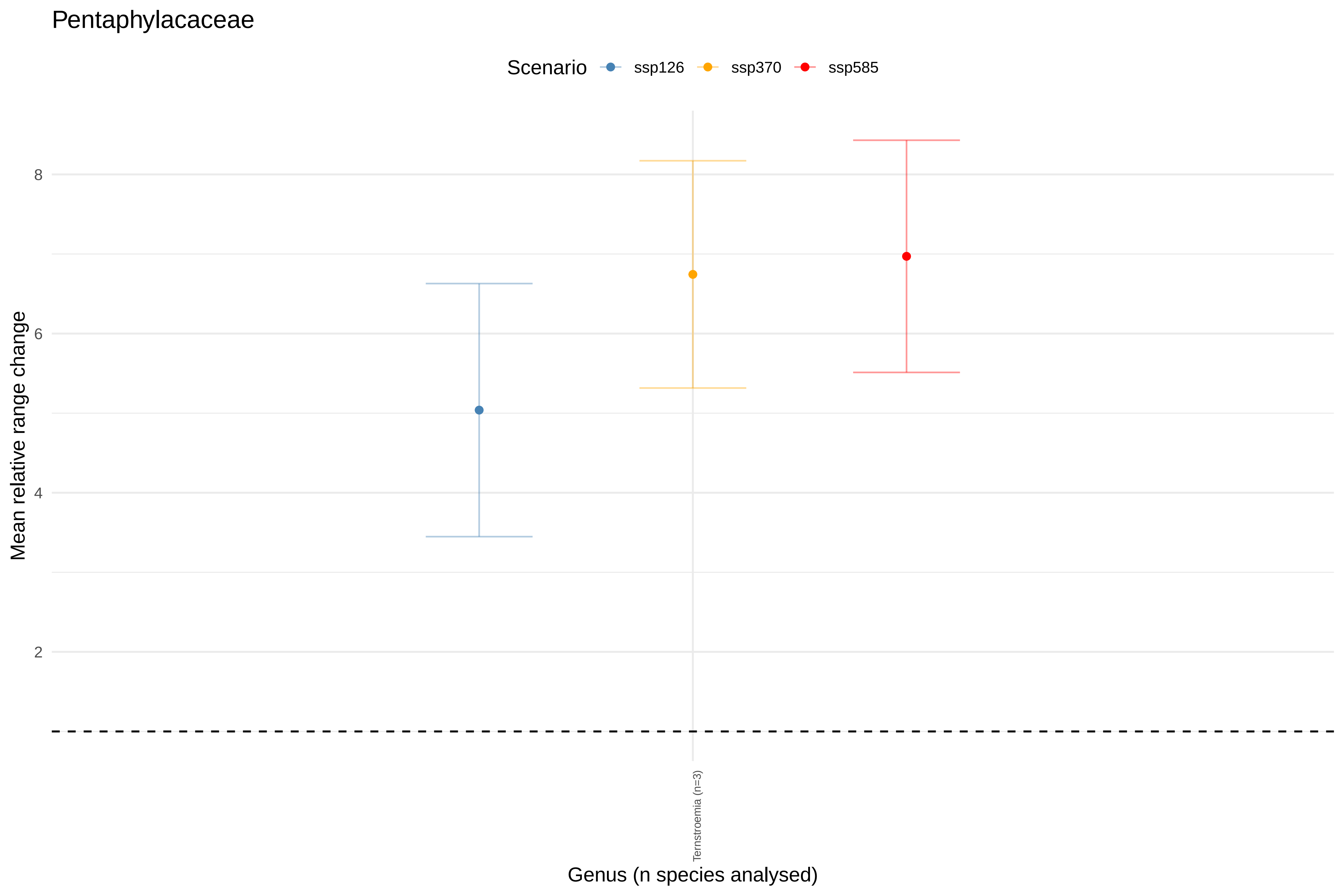

Pentaphylacaceae
Scenario
ssp126
ssp370
ssp585
Mean relative range change
8
6
4
2
Ternstroemia (n=3)
Genus (n species analysed)

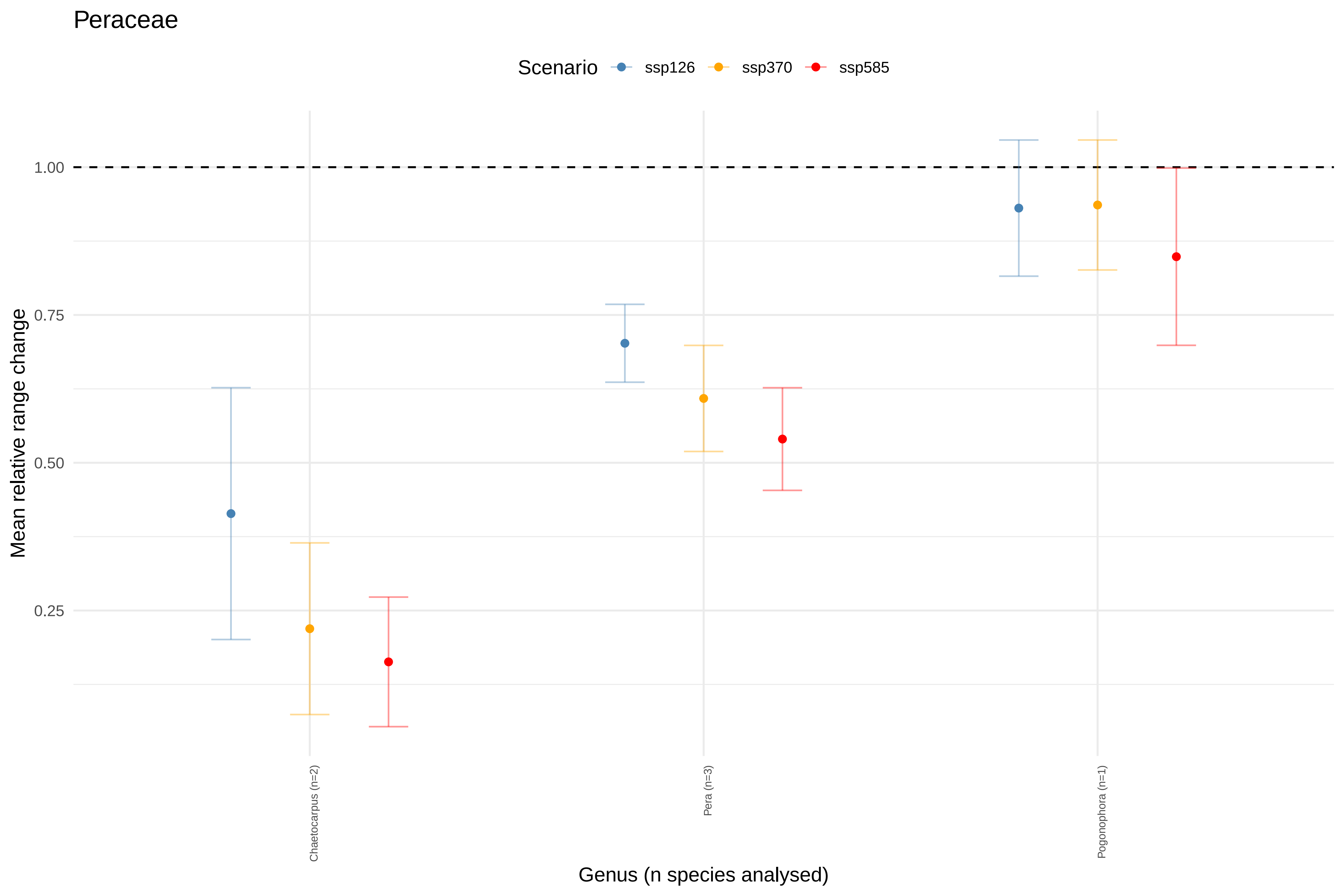

Peraceae
Scenario
ssp126
ssp370
ssp585
Mean relative range change
1.00
0.75
0.50
0.25
Chaetocarpus (n=2)
Pera (n=3)
Pogonophora (n=1)
Genus (n species analysed)

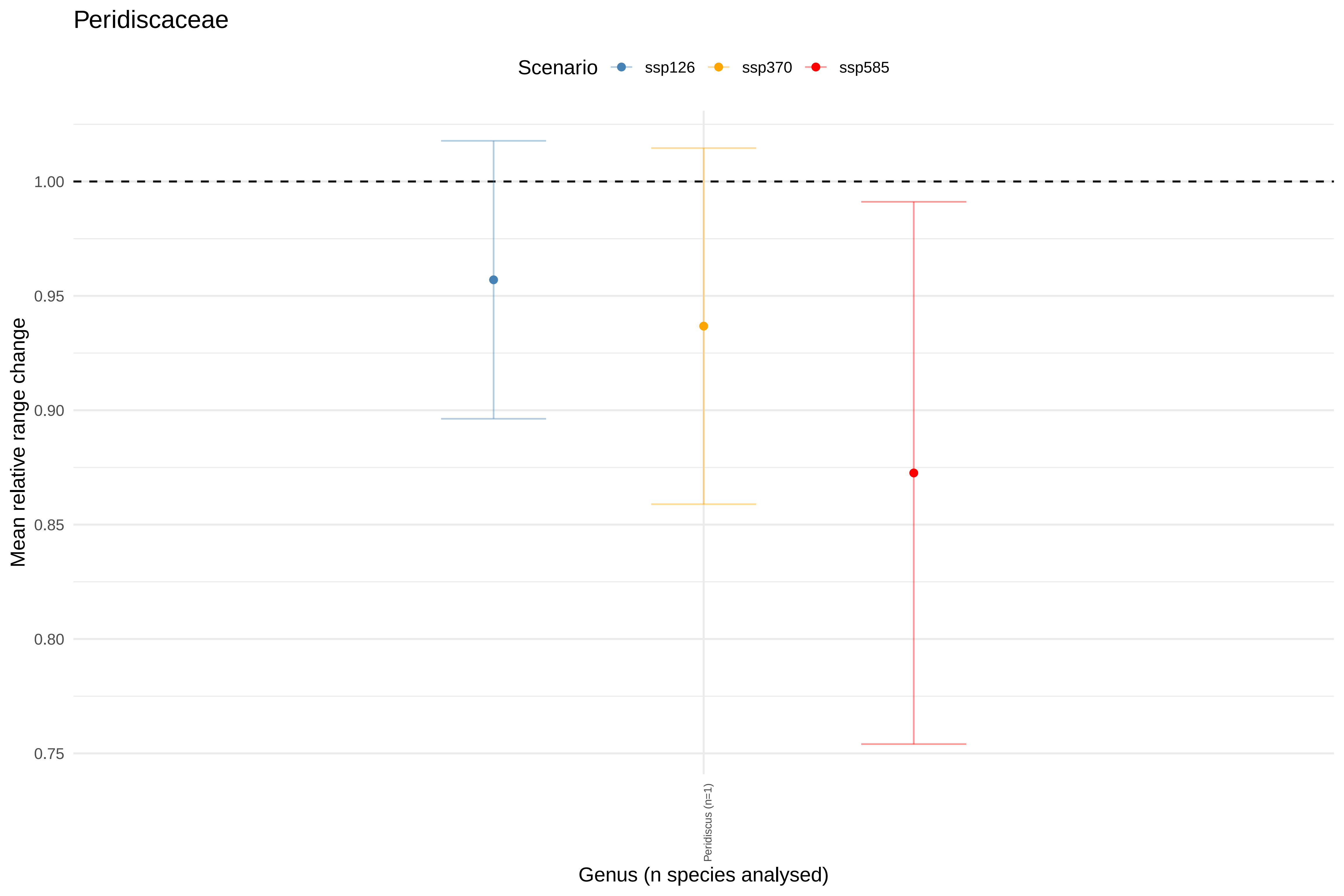

Peridiscaceae
Scenario
ssp126
ssp370
ssp585
Mean relative range change
1.00
0.95
0.90
0.85
0.80
0.75
Peridiscus (n=1)
Genus (n species analysed)

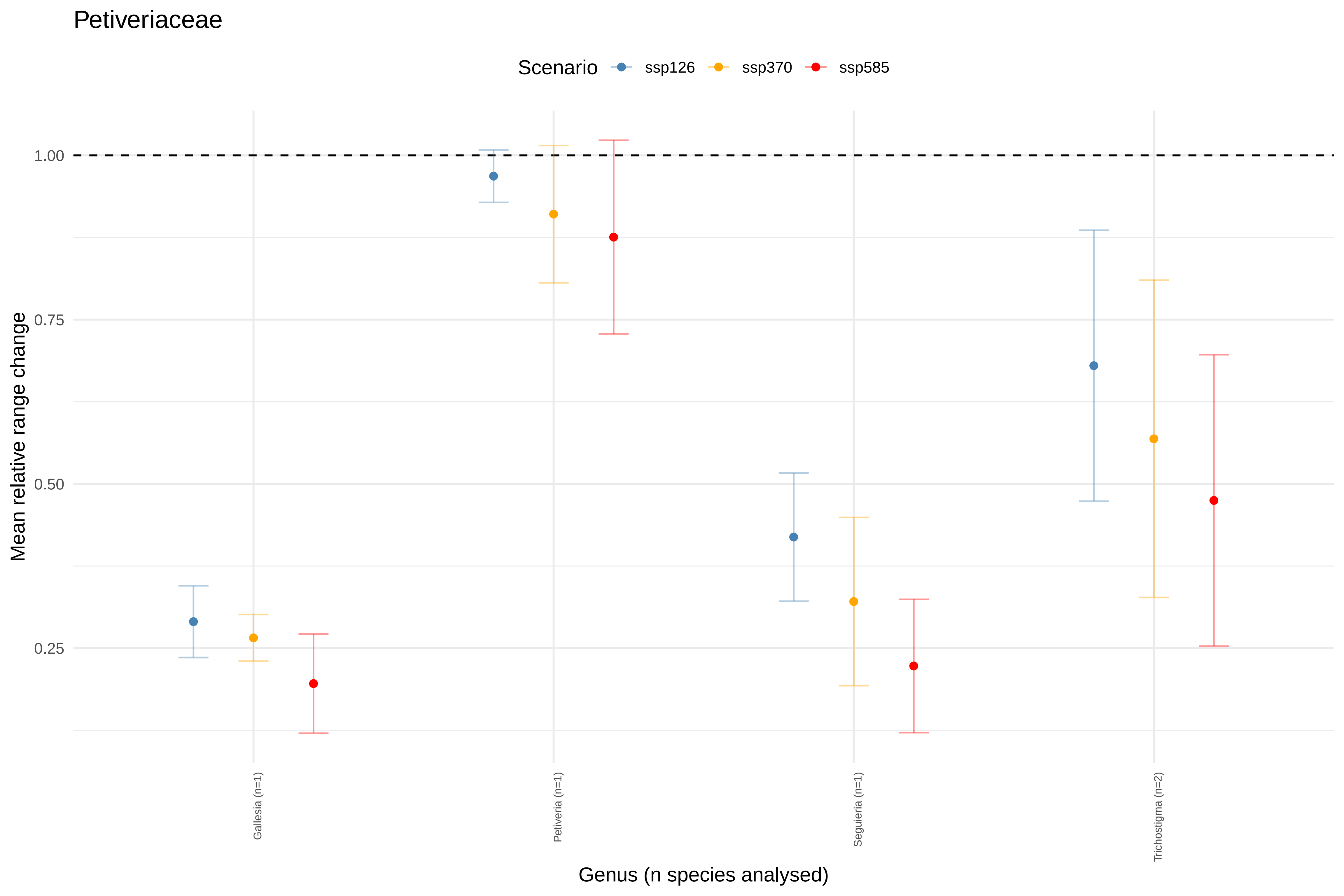

Petiveriaceae
Scenario
ssp126
ssp370
ssp585
Mean relative range change
1.00
0.75
0.50
0.25
Gallesia (n=1)
Petiveria (n=1)
Seguieria (n=1)
Trichostigma (n=2)
Genus (n species analysed)

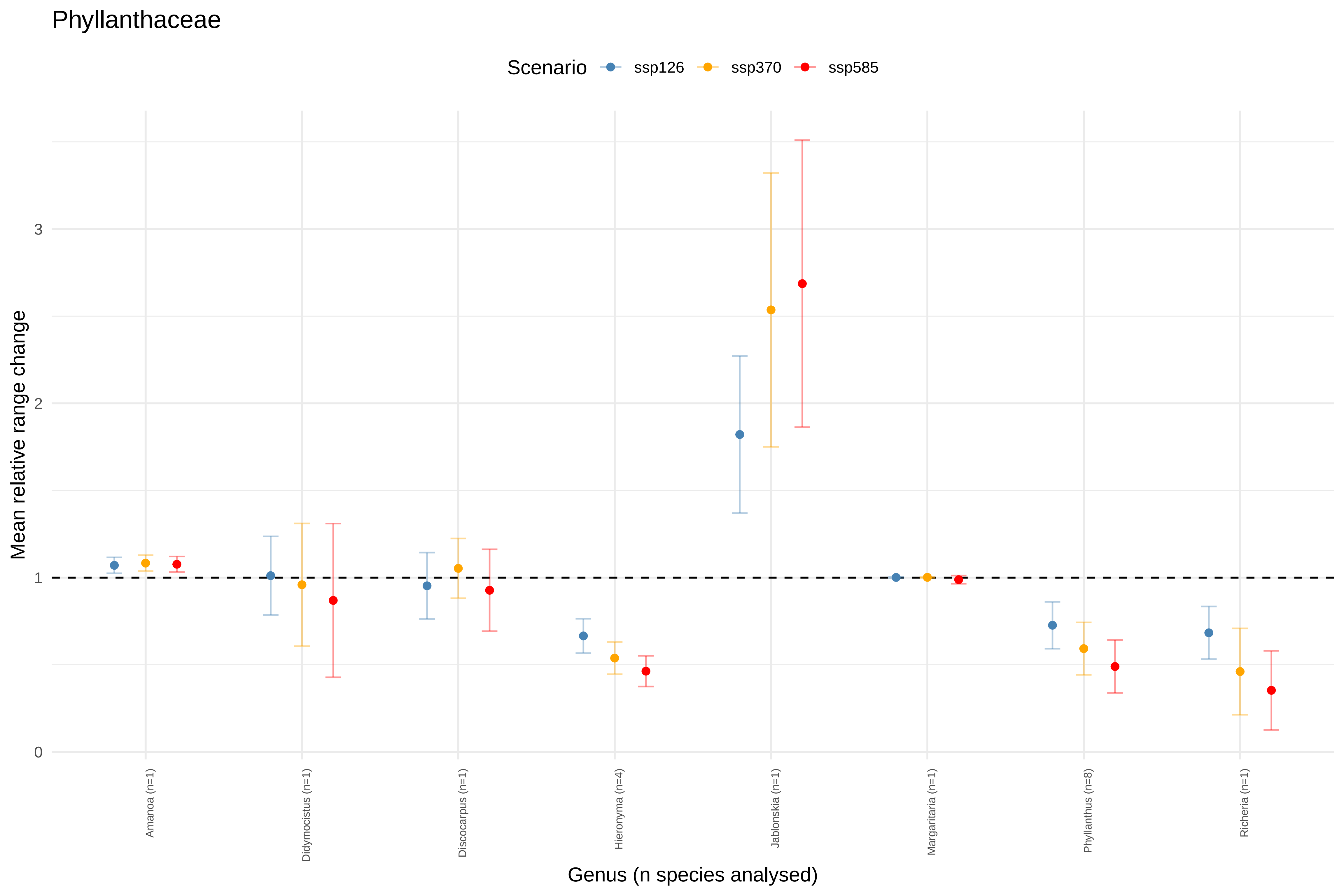

Phyllanthaceae
Scenario
ssp126
ssp370
ssp585
Mean relative range change
0
1
2
3
Amanoa (n=1)
Didymocistus (n=1)
Discocarpus (n=1)
Hieronyma (n=4)
Jablonskia (n=1)
Margaritaria (n=1)
Phyllanthus (n=8)
Richeria (n=1)
Genus (n species analysed)

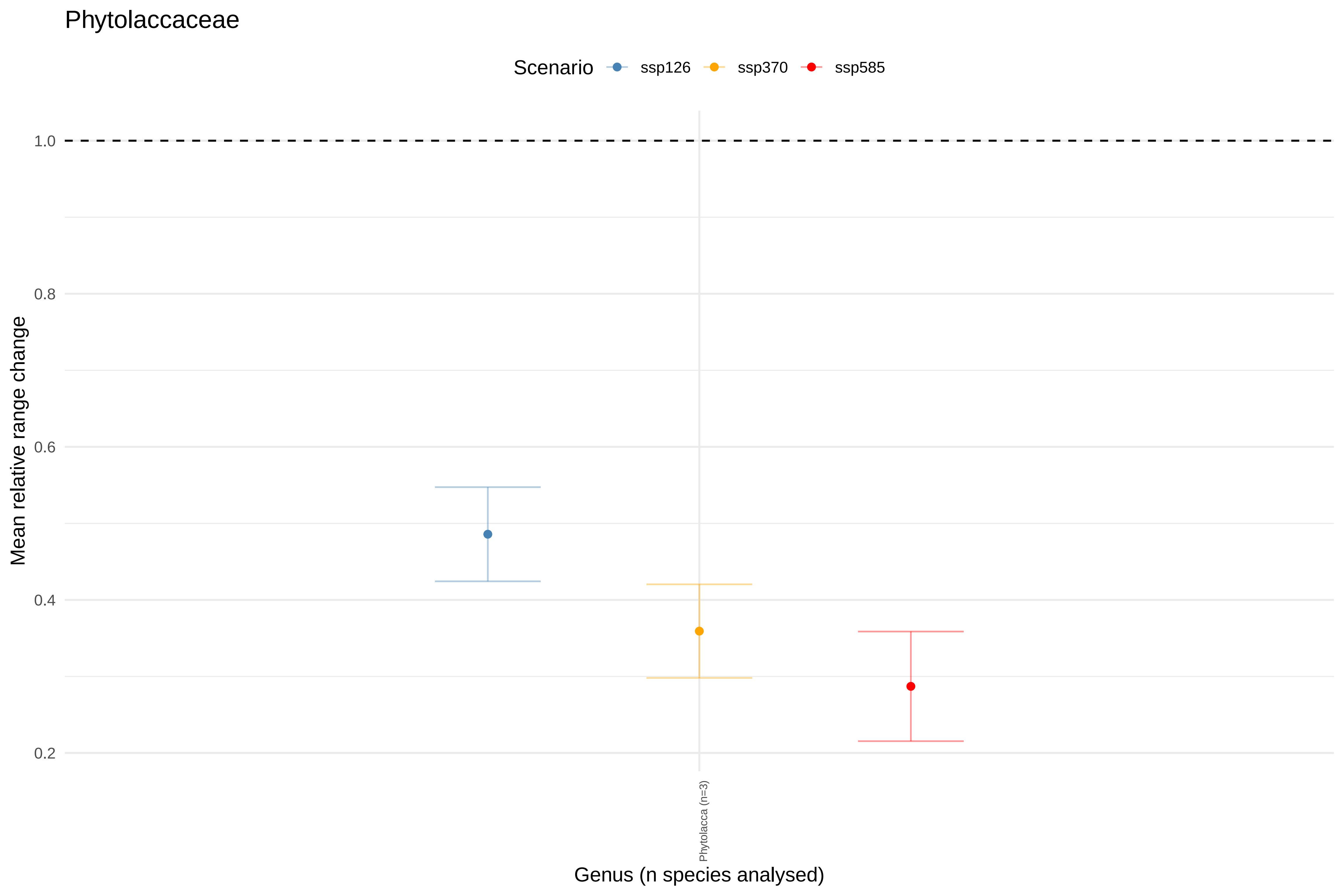

Phytolaccaceae
Scenario
ssp126
ssp370
ssp585
Mean relative range change
1.0
0.8
0.6
0.4
0.2
Phytolacca (n=3)
Genus (n species analysed)

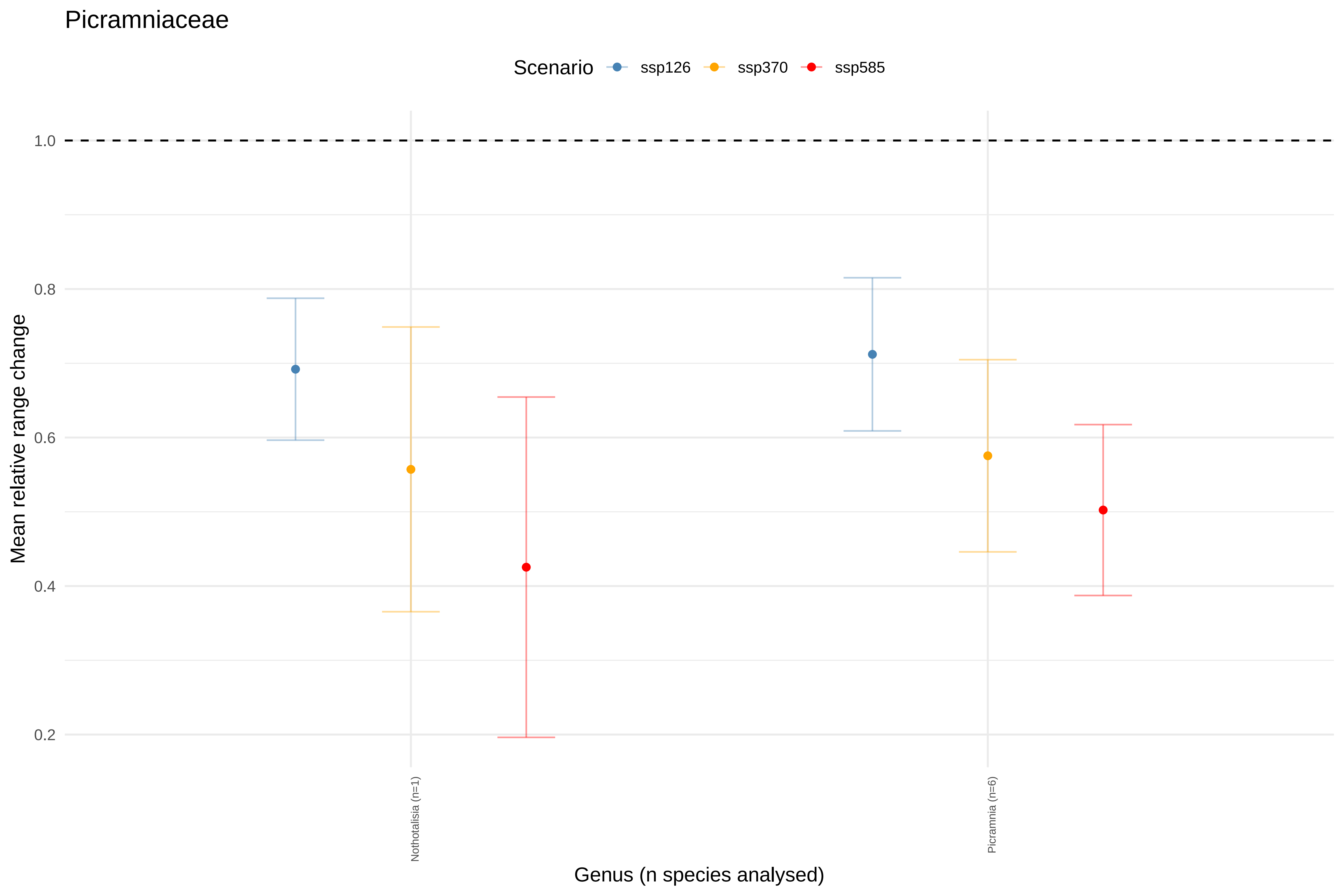

Picramniaceae
Scenario
ssp126
ssp370
ssp585
1.0
0.8
0.6
0.4
0.2
Mean relative range change
Nothotalisia (n=1)
Picramnia (n=6)
Genus (n species analysed)

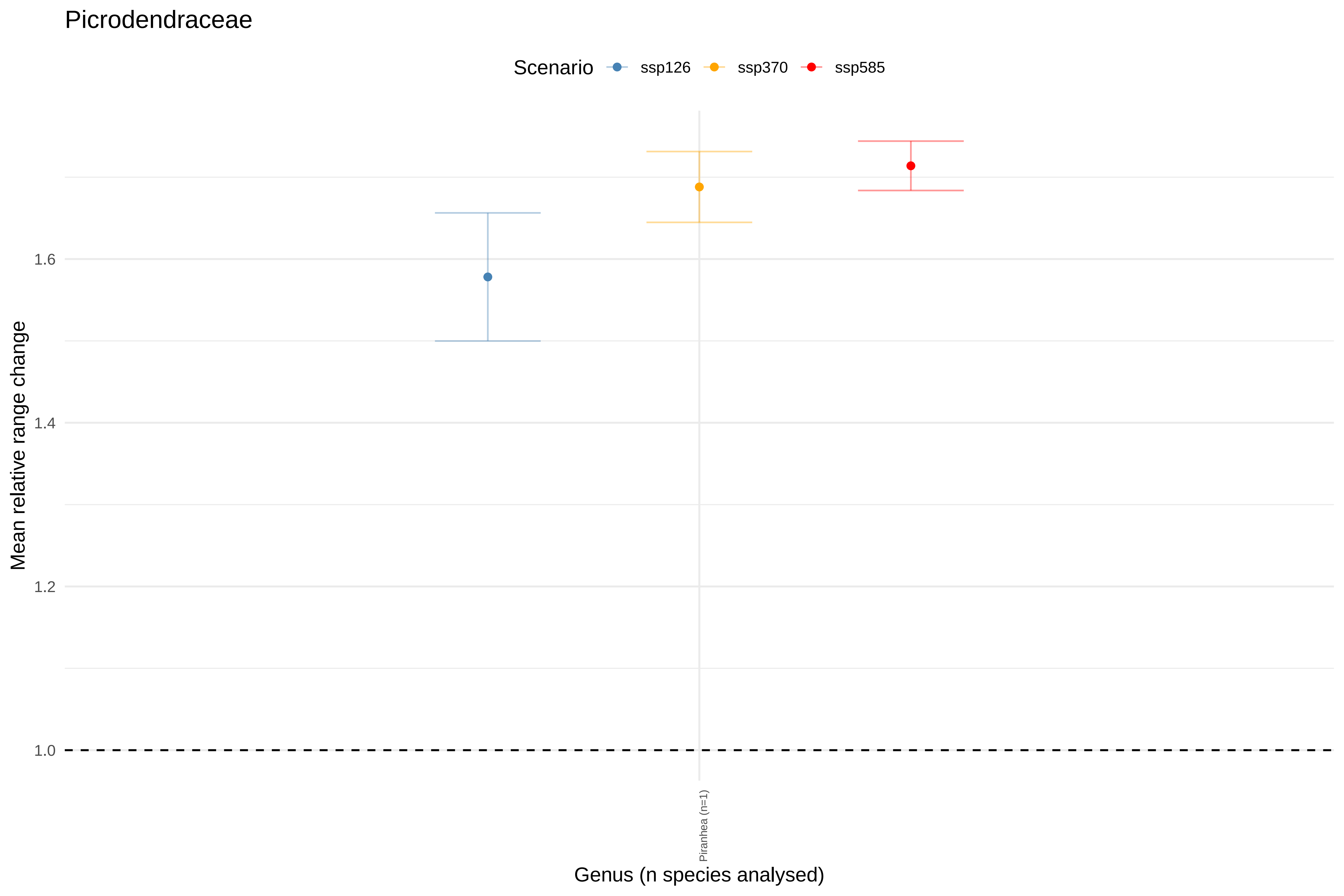
Picrodendraceae
Scenario
ssp126
ssp370
ssp585
Mean relative range change
1.6
1.4
1.2
1.0
Piranhea (n=1)
Genus (n species analysed)

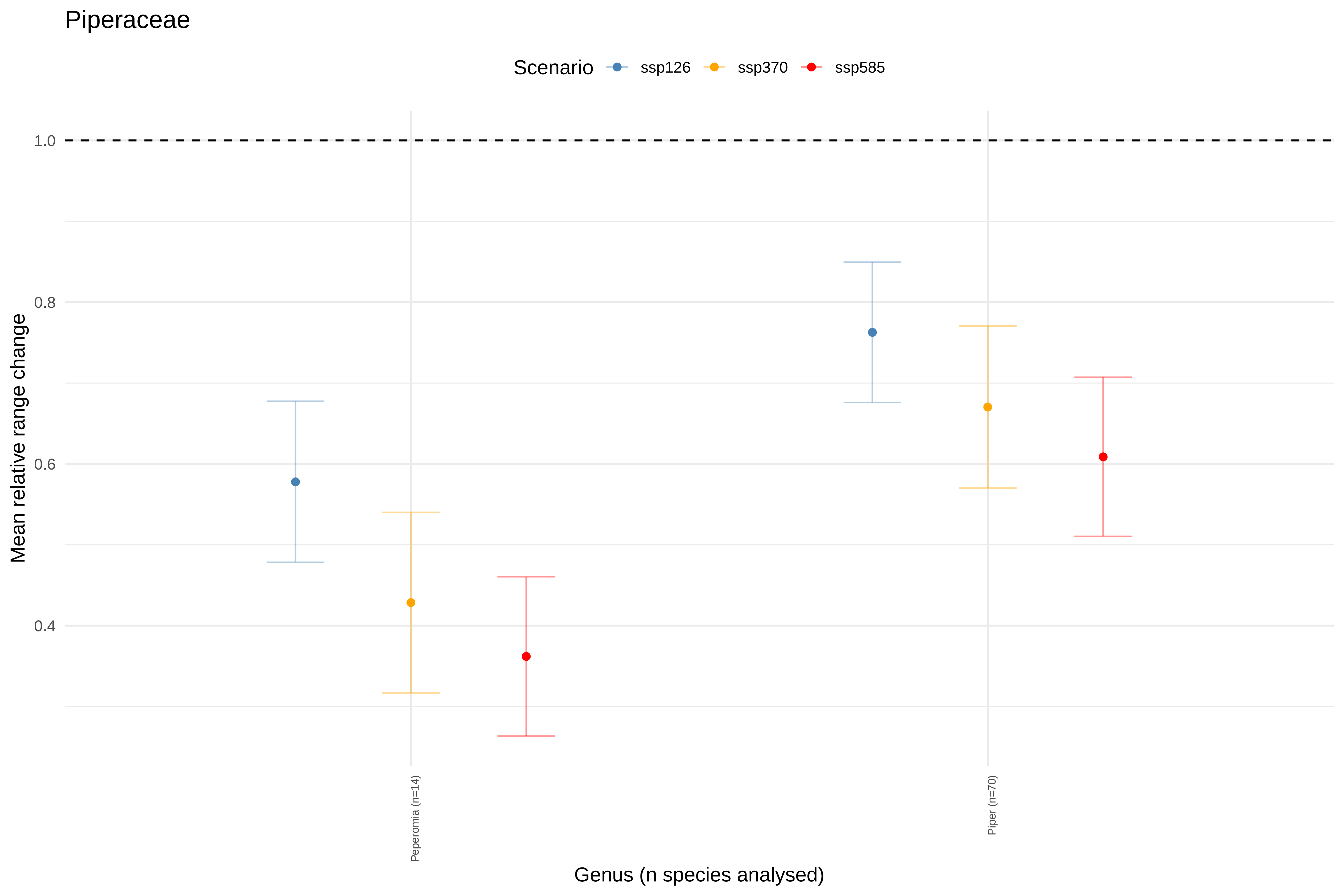

Piperaceae
Scenario
ssp126
ssp370
ssp585
1.0
0.8
0.6
0.4
Mean relative range change
Peperomia (n=14)
Piper (n=70)
Genus (n species analysed)

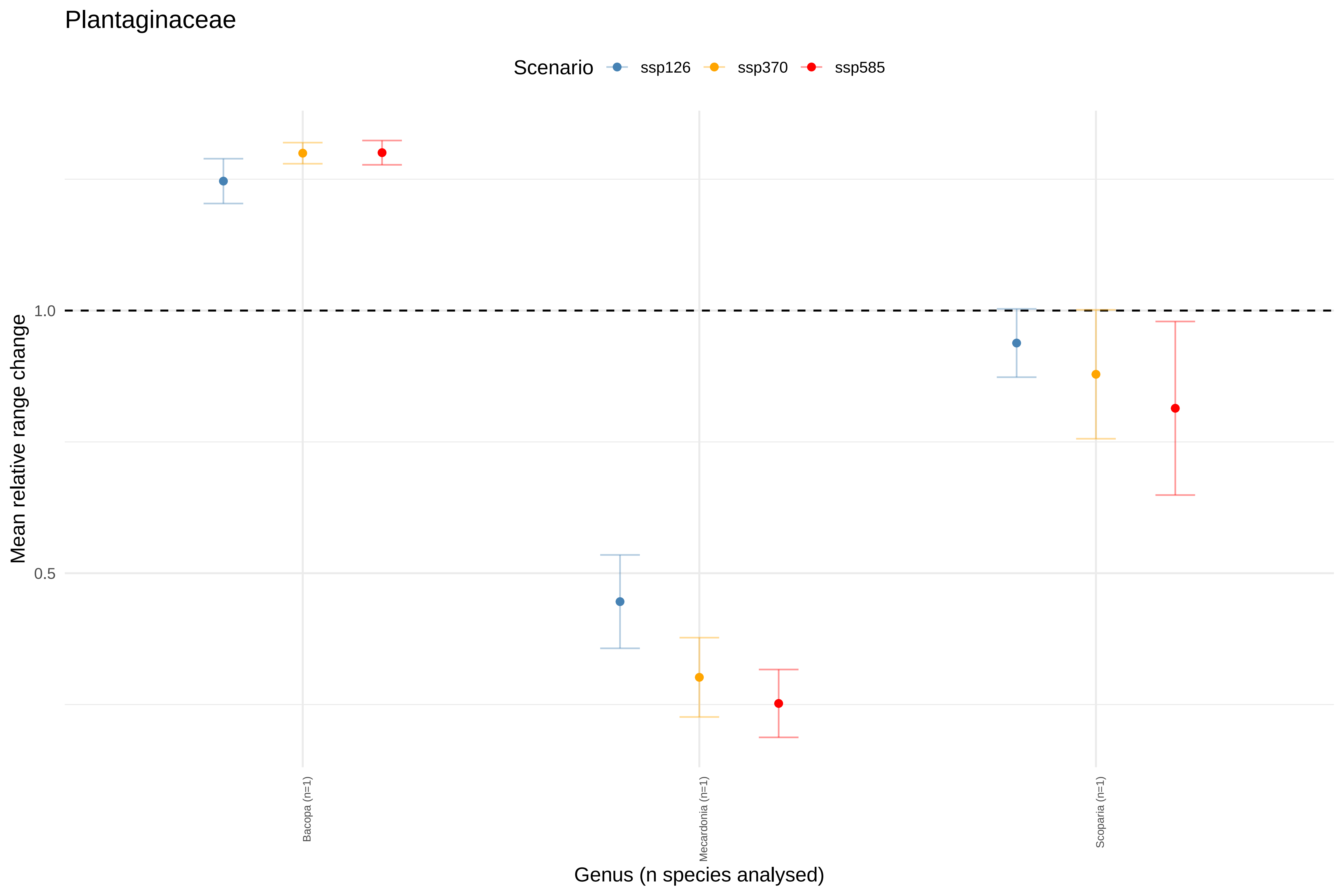
Plantaginaceae
Scenario
ssp126
ssp370
ssp585
Mean relative range change
1.0
0.5
Bacopa (n=1)
Mecardonia (n=1)
Scoparia (n=1)
Genus (n species analysed)

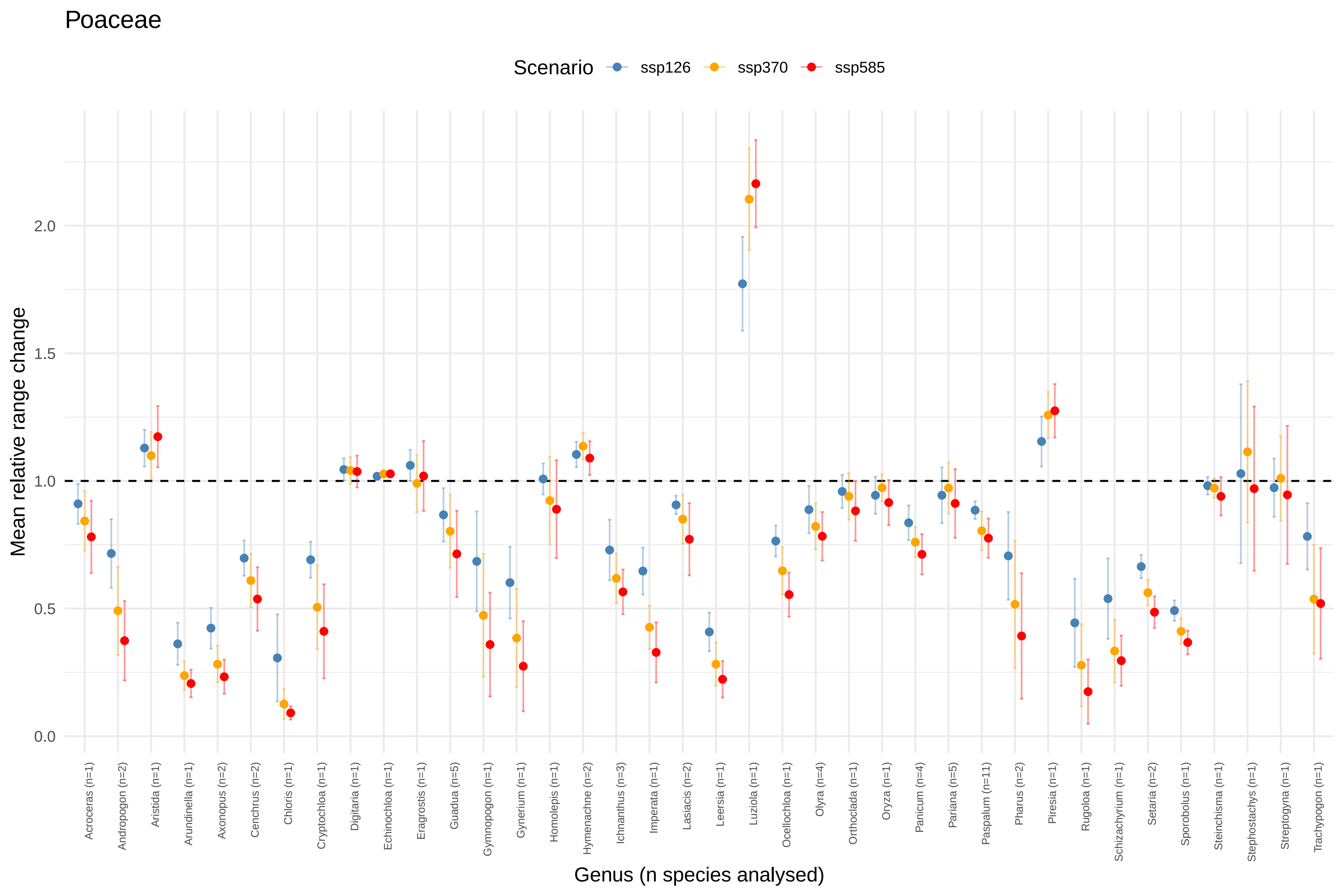

Poaceae
Scenario
ssp126
ssp370
ssp585
Mean relative range change
0.0
0.5
1.0
1.5
2.0
Genus (n species analysed)
Acroceras (n=1)
Andropogon (n=2)
Aristida (n=1)
Arundinella (n=1)
Axonopus (n=2)
Cenchrus (n=2)
Chloris (n=1)
Cryptochloa (n=1)
Digitaria (n=1)
Echinochloa (n=1)
Eragrostis (n=1)
Guadua (n=5)
Gymnopogon (n=1)
Gynerium (n=1)
Homolepis (n=1)
Hymenachne (n=2)
Ichnanthus (n=3)
Imperata (n=1)
Lasiacis (n=2)
Leersia (n=1)
Luziola (n=1)
Ocellochloa (n=1)
Olyra (n=4)
Orthoclada (n=1)
Oryza (n=1)
Panicum (n=4)
Pariana (n=5)
Paspalum (n=11)
Pharus (n=2)
Piresia (n=1)
Rugoloa (n=1)
Schizachyrium (n=1)
Setaria (n=2)
Sporobolus (n=1)
Steinchisma (n=1)
Stephostachys (n=1)
Streptogyna (n=1)
Trachypogon (n=1)

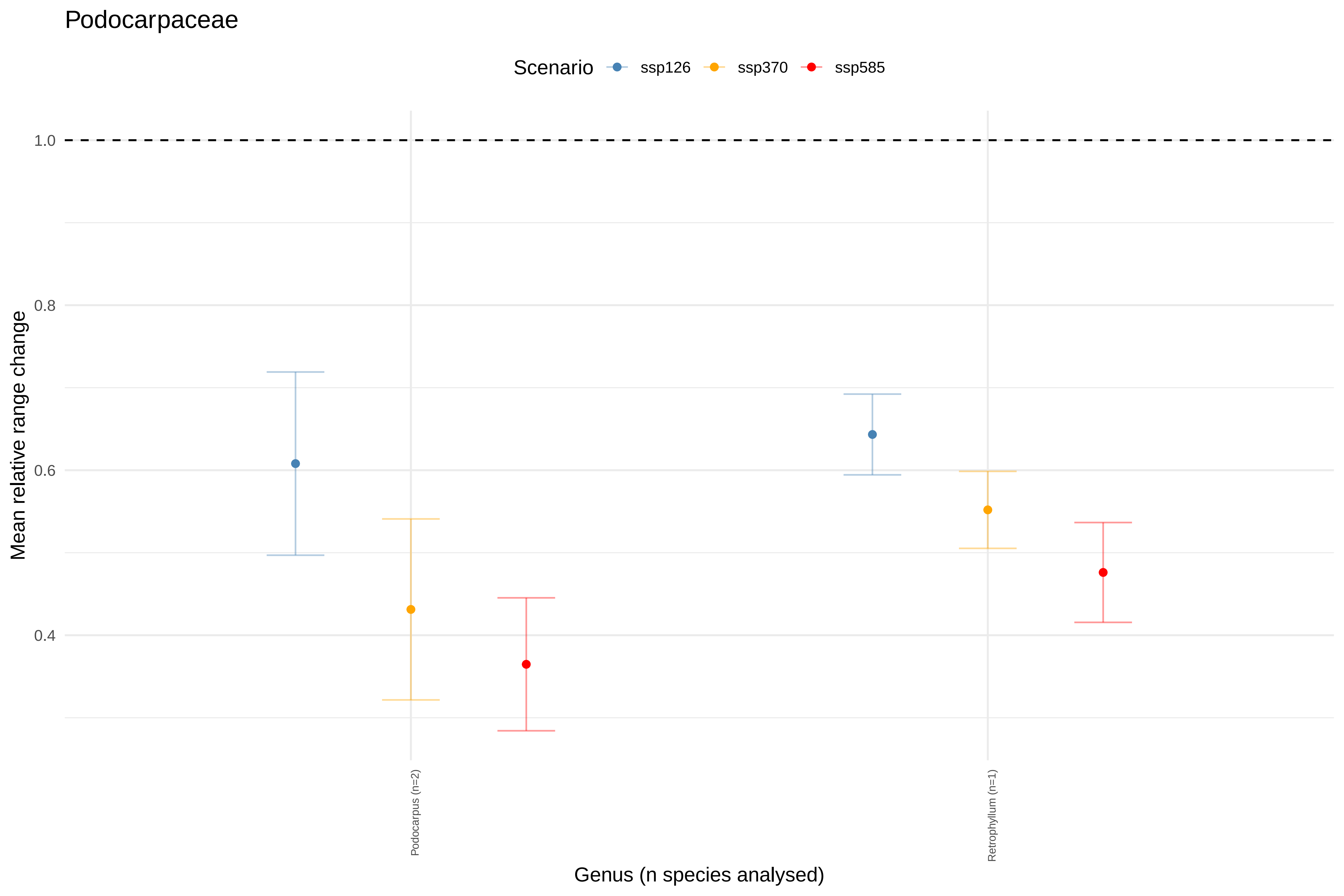
Podocarpaceae
Scenario
ssp126
ssp370
ssp585
1.0
0.8
0.6
0.4
Mean relative range change
Podocarpus (n=2)
Retrophyllum (n=1)
Genus (n species analysed)

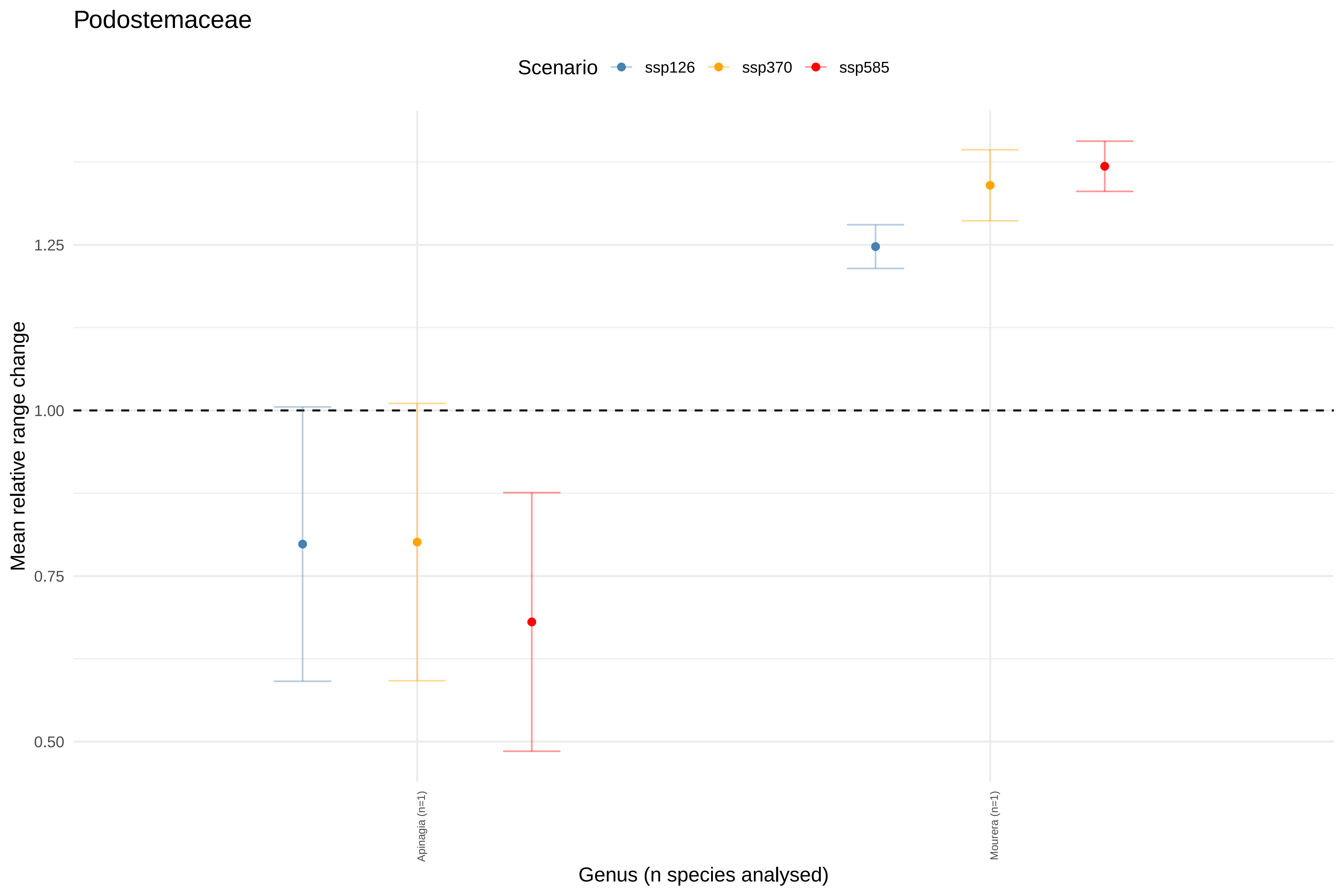

Podostemaceae
Scenario
ssp126
ssp370
ssp585
Mean relative range change
1.25
1.00
0.75
0.50
Apinagia (n=1)
Mourera (n=1)
Genus (n species analysed)

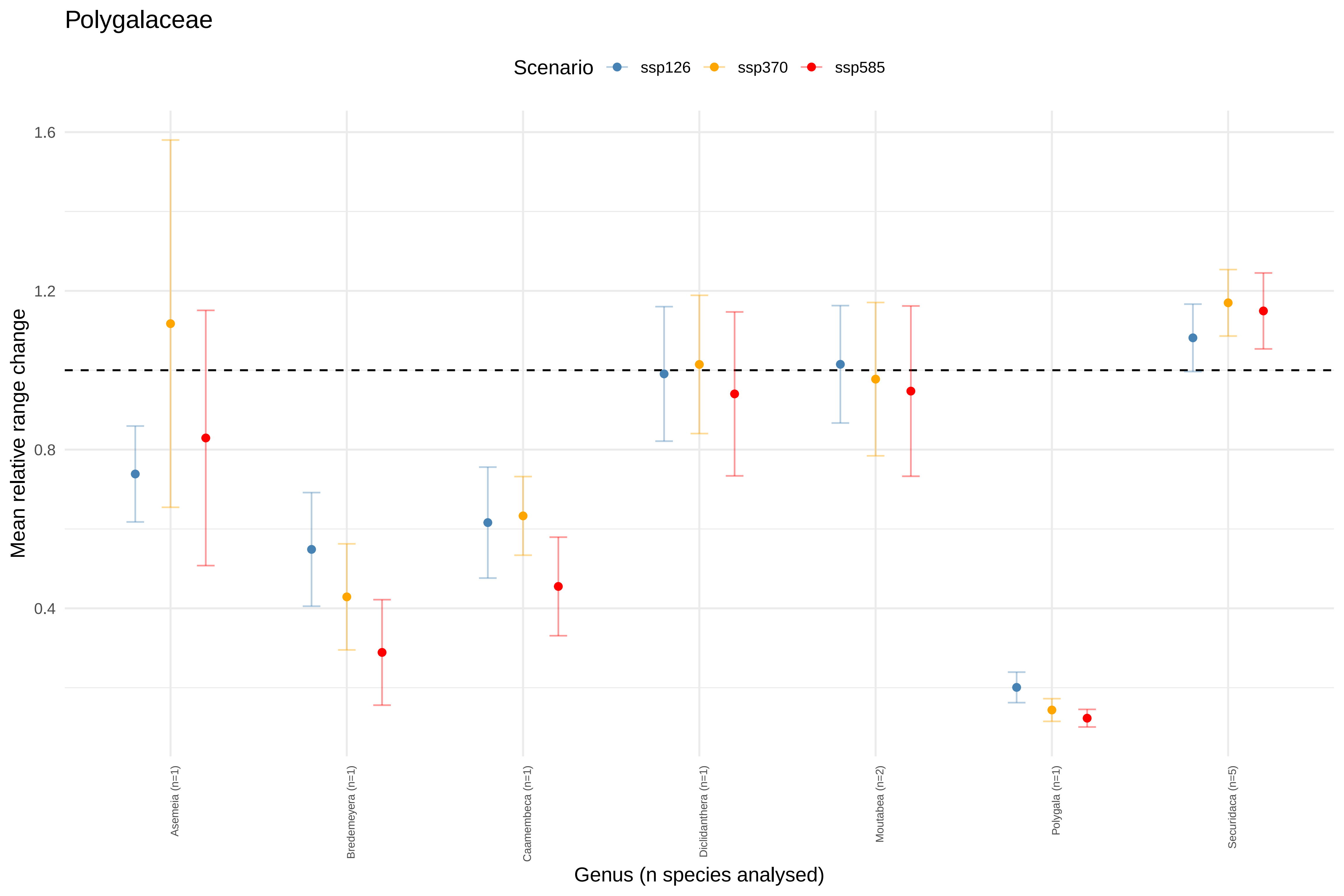
Polygalaceae
Scenario
ssp126
ssp370
ssp585
1.6
1.2
0.8
0.4
Mean relative range change
Asemeia (n=1)
Bredemeyera (n=1)
Caamembeca (n=1)
Diclidanthera (n=1)
Moutabea (n=2)
Polygala (n=1)
Securidaca (n=5)
Genus (n species analysed)

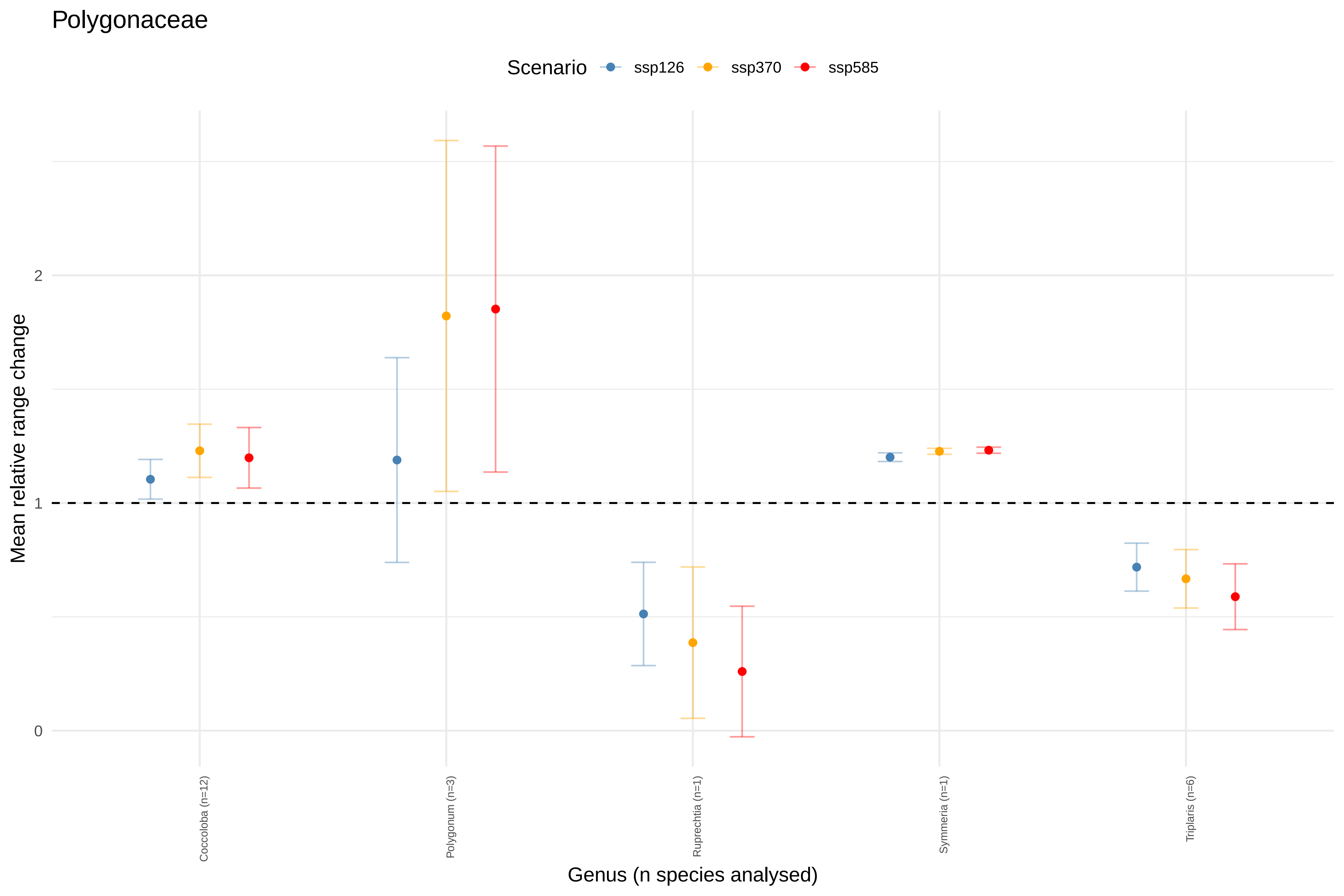
Polygonaceae
Scenario
ssp126
ssp370
ssp585
Mean relative range change
2
1
0
Coccoloba (n=12)
Polygonum (n=3)
Ruprechtia (n=1)
Symmeria (n=1)
Triplaris (n=6)
Genus (n species analysed)

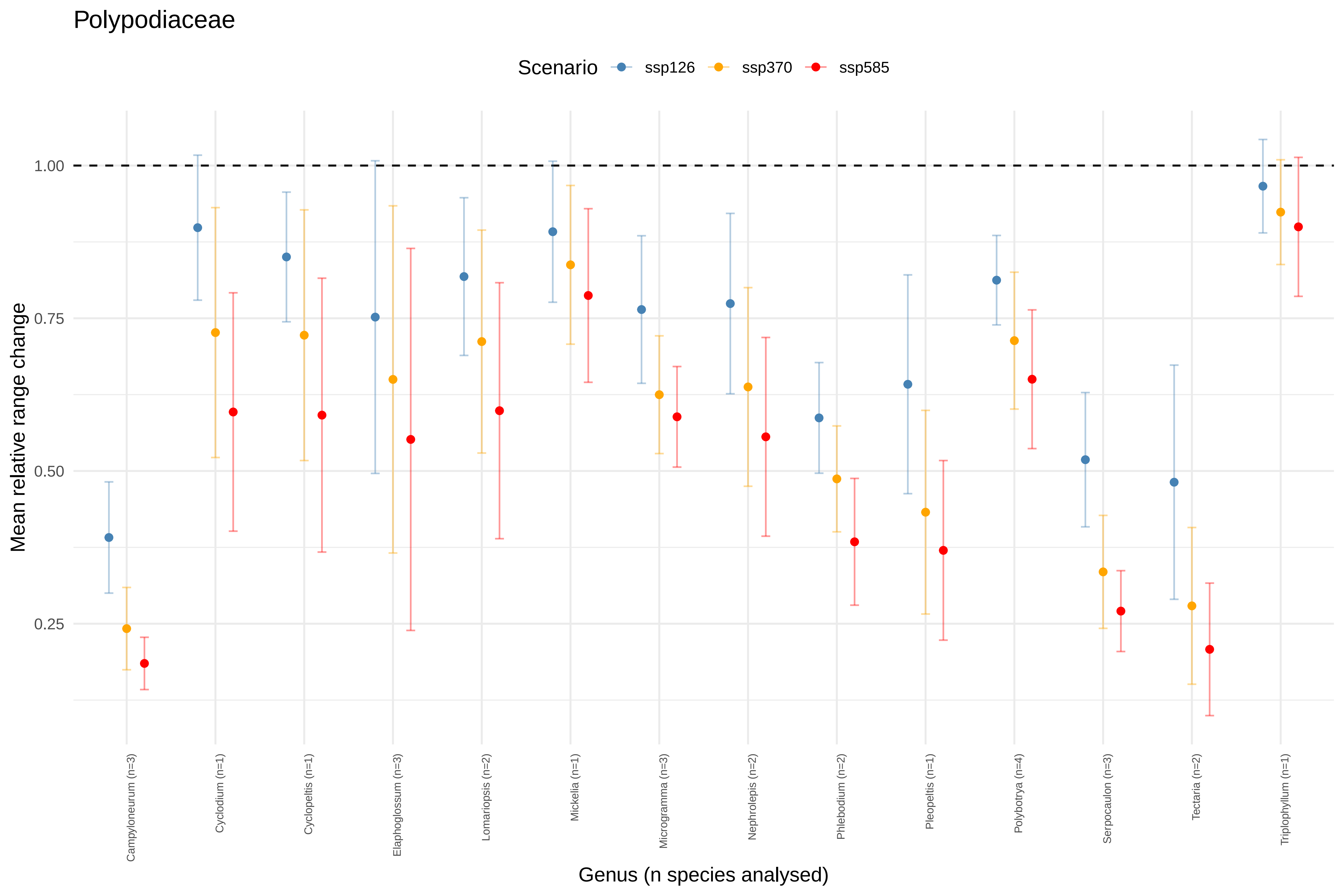

Polypodiaceae
Scenario
ssp126
ssp370
ssp585
Mean relative range change
1.00
0.75
0.50
0.25
Campyloneurum (n=3)
Cyclodium (n=1)
Cyclopeltis (n=1)
Elaphoglossum (n=3)
Lomariopsis (n=2)
Mickelia (n=1)
Microgramma (n=3)
Nephrolepis (n=2)
Phlebodium (n=2)
Pleopeltis (n=1)
Polybotrya (n=4)
Serpocaulon (n=3)
Tectaria (n=2)
Triplophyllum (n=1)
Genus (n species analysed)

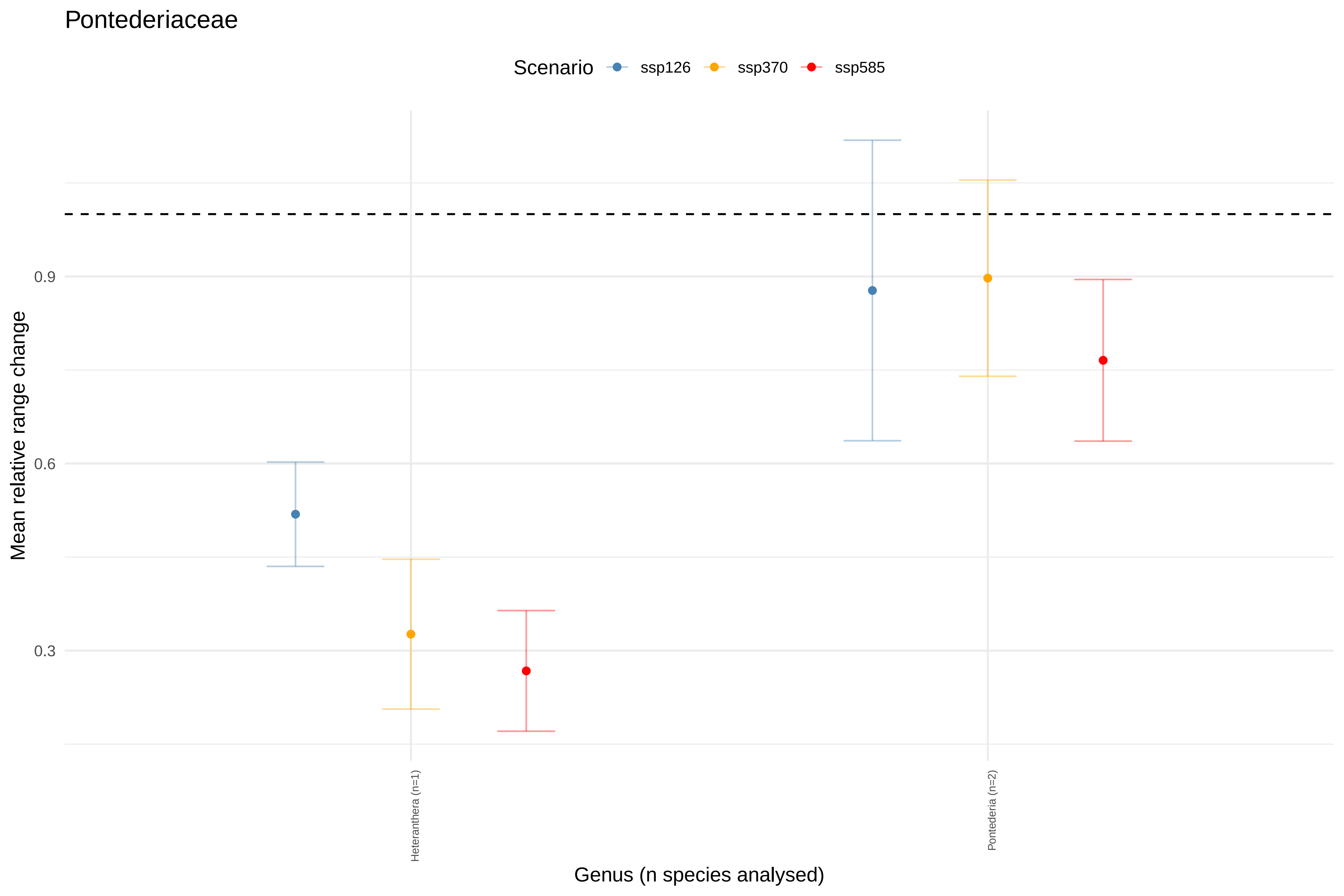

Pontederiaceae
Scenario
ssp126
ssp370
ssp585
Mean relative range change
0.9
0.6
0.3
Heteranthera (n=1)
Pontederia (n=2)
Genus (n species analysed)

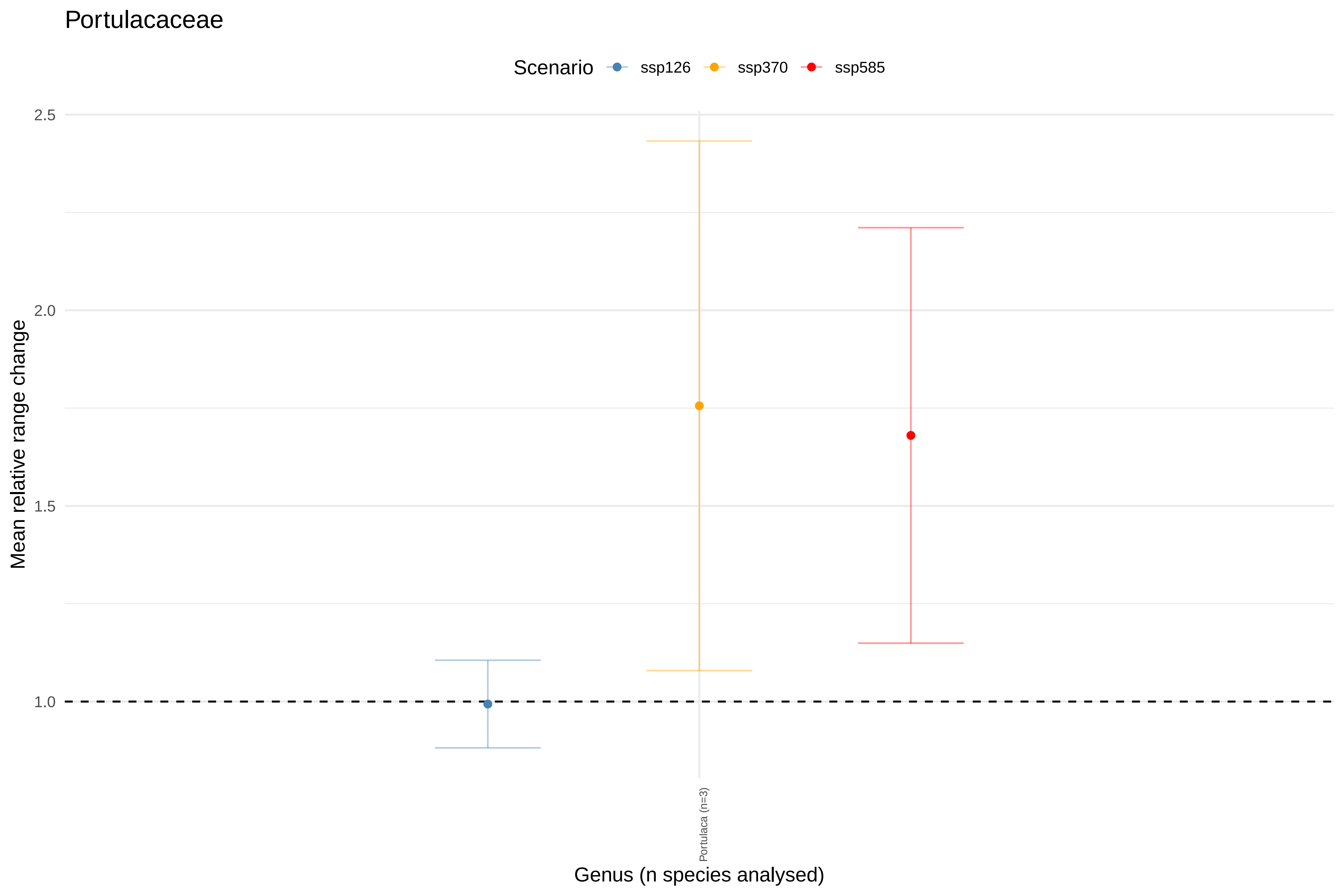

Portulacaceae
Scenario
ssp126
ssp370
ssp585
2.5
2.0
1.5
1.0
Mean relative range change
Portulaca (n=3)
Genus (n species analysed)

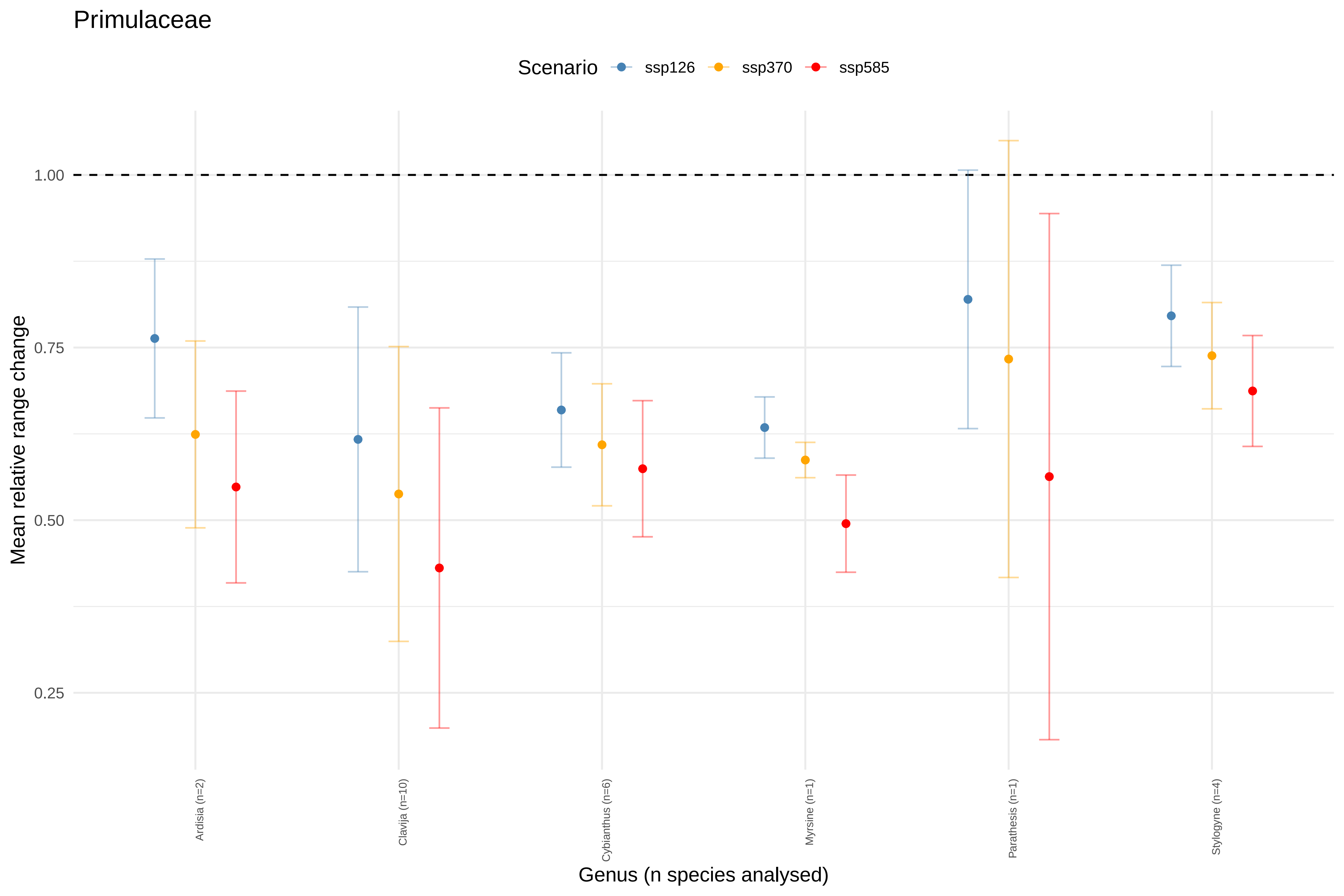

Primulaceae
Scenario
ssp126
ssp370
ssp585
Mean relative range change
1.00
0.75
0.50
0.25
Ardisia (n=2)
Clavija (n=10)
Cybianthus (n=6)
Myrsine (n=1)
Parathesis (n=1)
Stylogyne (n=4)
Genus (n species analysed)

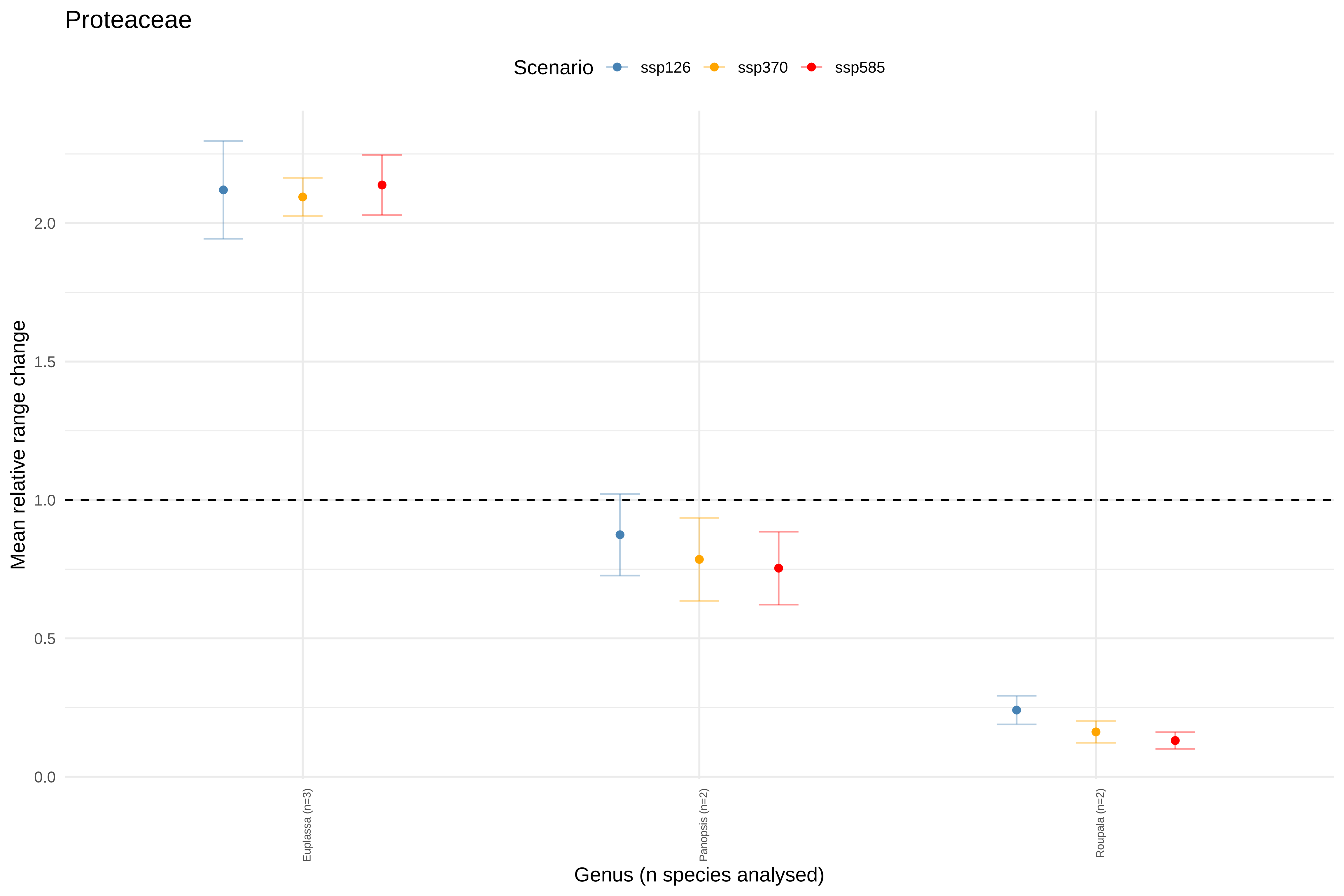

Proteaceae
Scenario
ssp126
ssp370
ssp585
Mean relative range change
2.0
1.5
1.0
0.5
0.0
Euplassa (n=3)
Panopsis (n=2)
Roupala (n=2)
Genus (n species analysed)

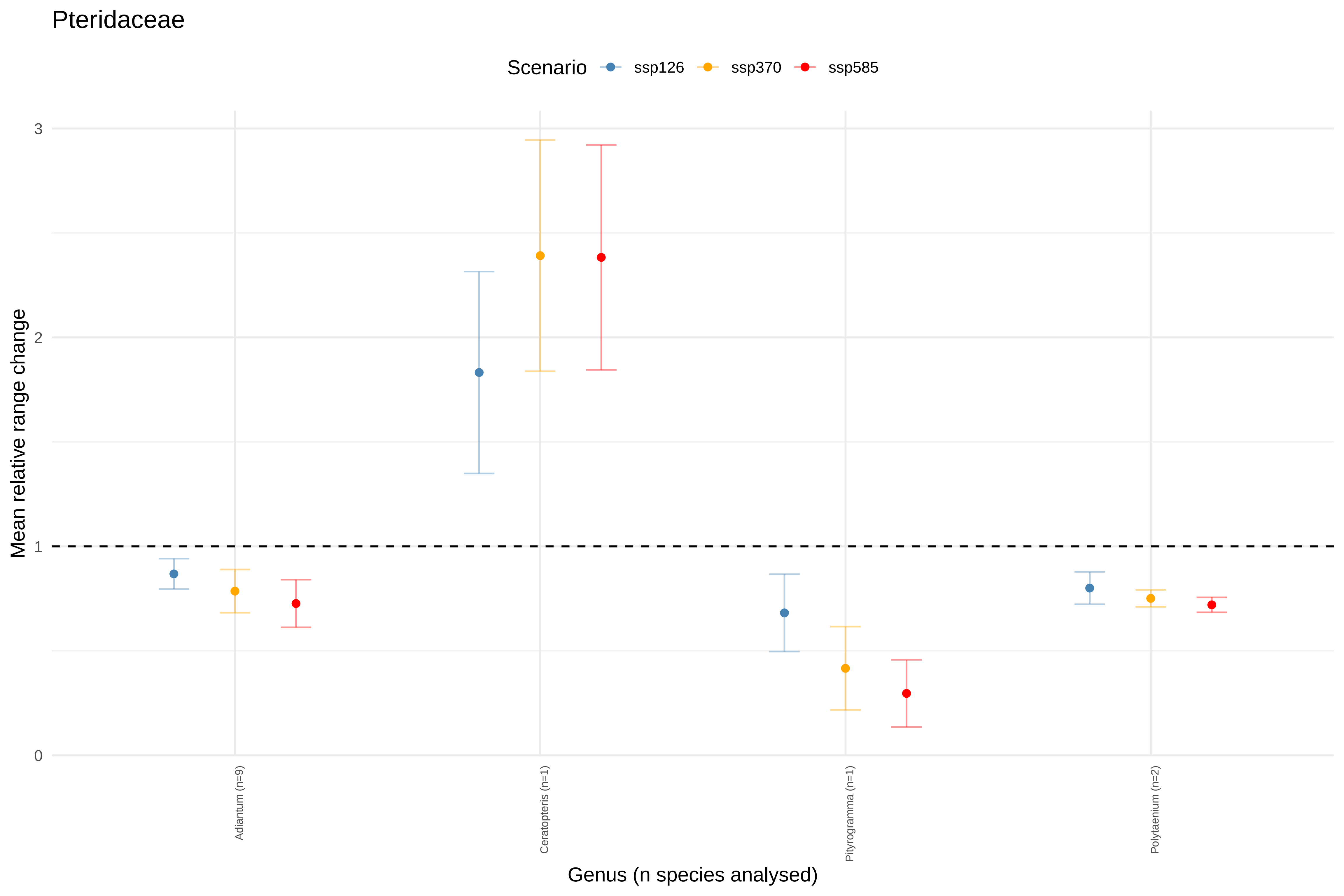
Pteridaceae
Scenario
ssp126
ssp370
ssp585
Mean relative range change
0
1
2
3
Adiantum (n=9)
Ceratopteris (n=1)
Pityrogramma (n=1)
Polytaenium (n=2)
Genus (n species analysed)

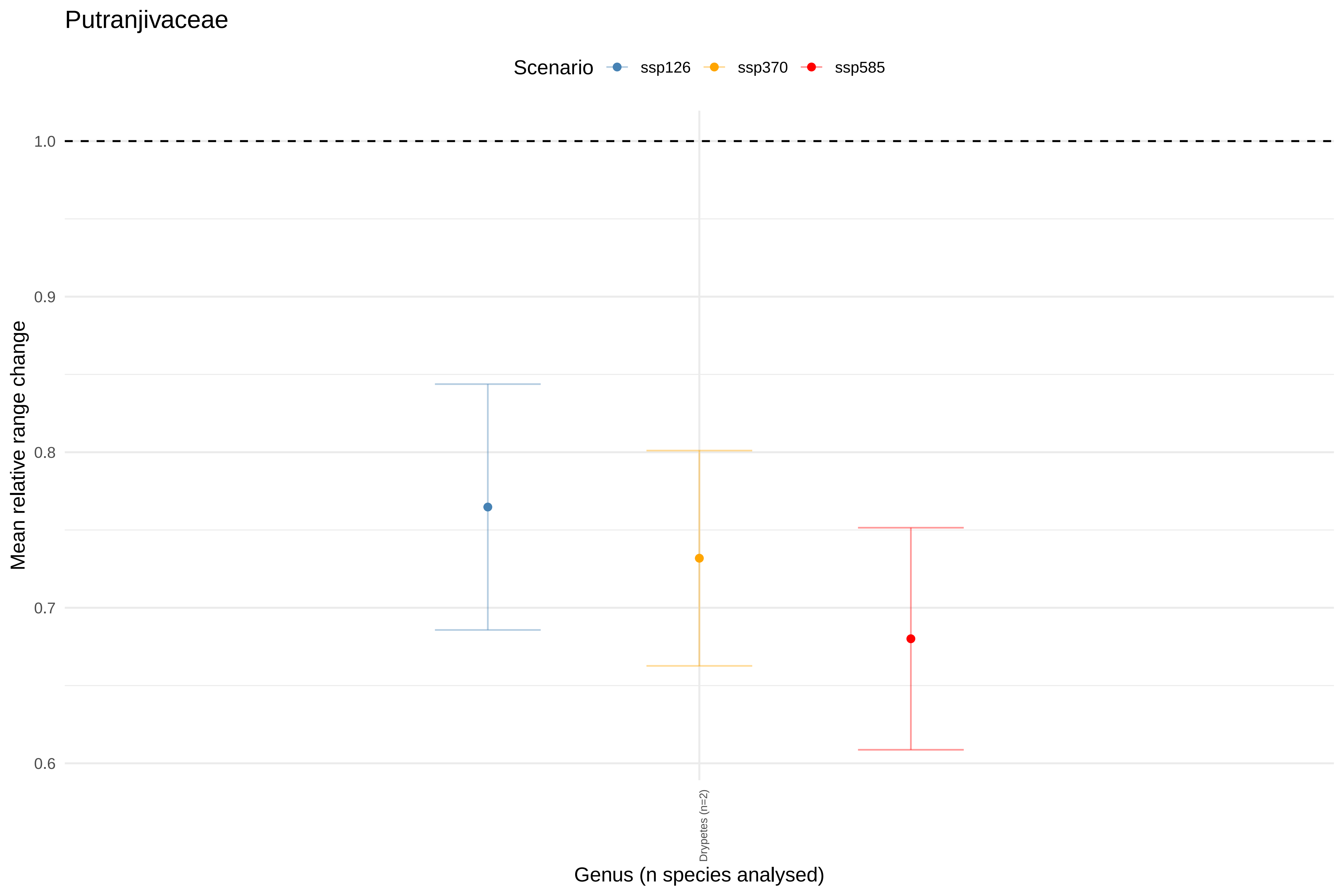

Putranjivaceae
Scenario
ssp126
ssp370
ssp585
Mean relative range change
1.0
0.9
0.8
0.7
0.6
Drypetes (n=2)
Genus (n species analysed)

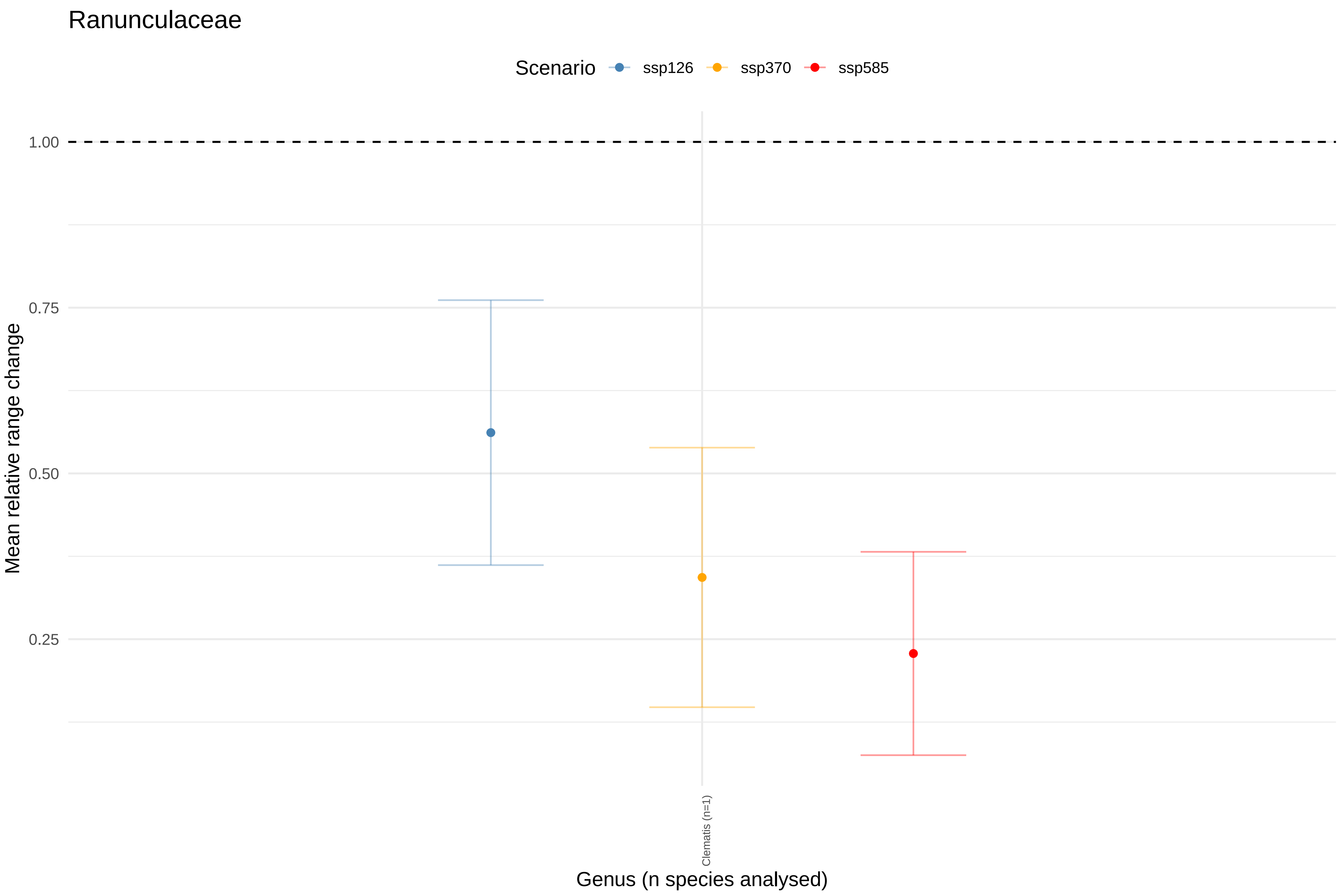

Ranunculaceae
Scenario
ssp126
ssp370
ssp585
1.00
0.75
0.50
0.25
Mean relative range change
Clematis (n=1)
Genus (n species analysed)

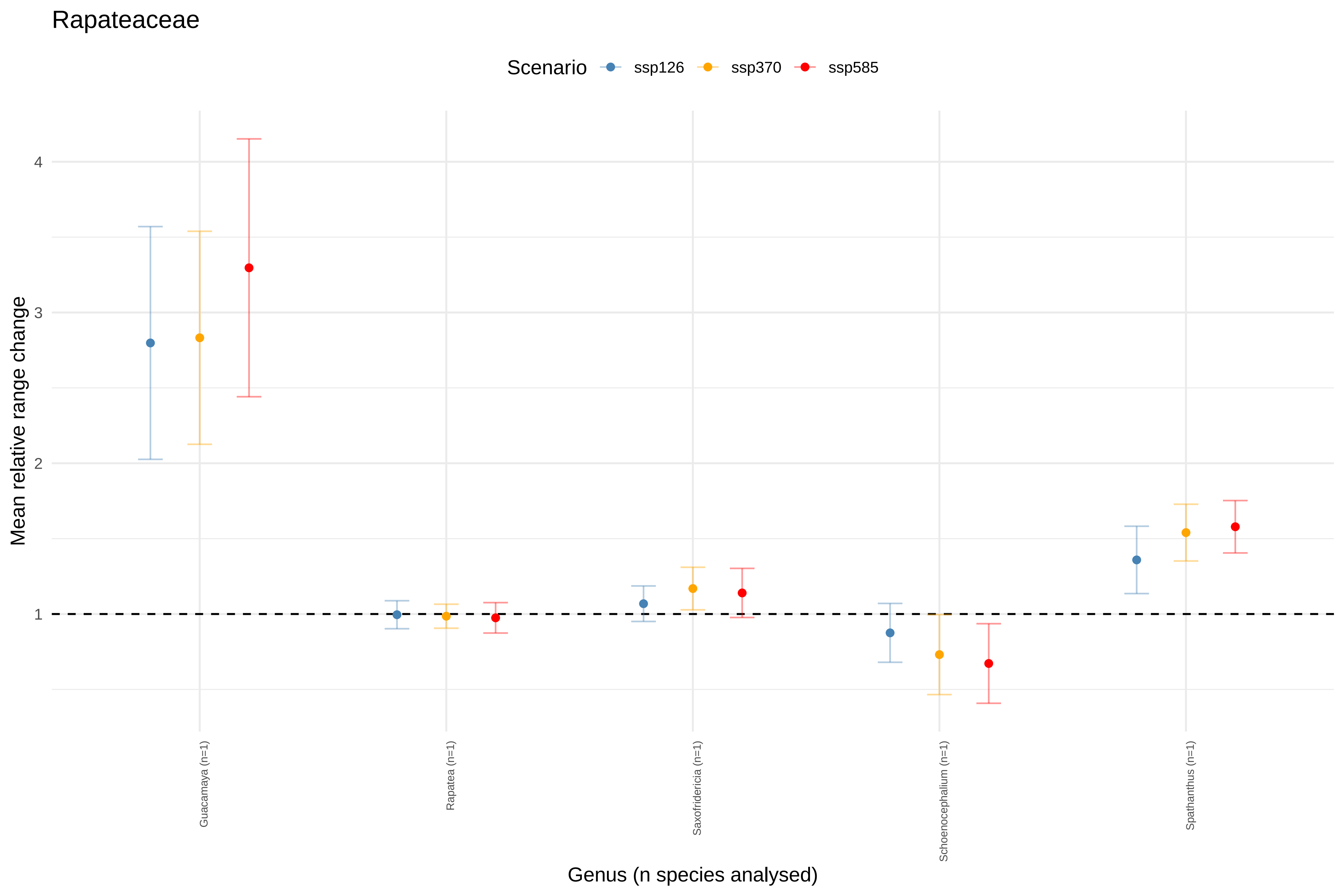

Rapateaceae
Scenario
ssp126
ssp370
ssp585
Mean relative range change
4
3
2
1
Guacamaya (n=1)
Rapatea (n=1)
Saxofridericia (n=1)
Schoenocephalium (n=1)
Spathanthus (n=1)
Genus (n species analysed)

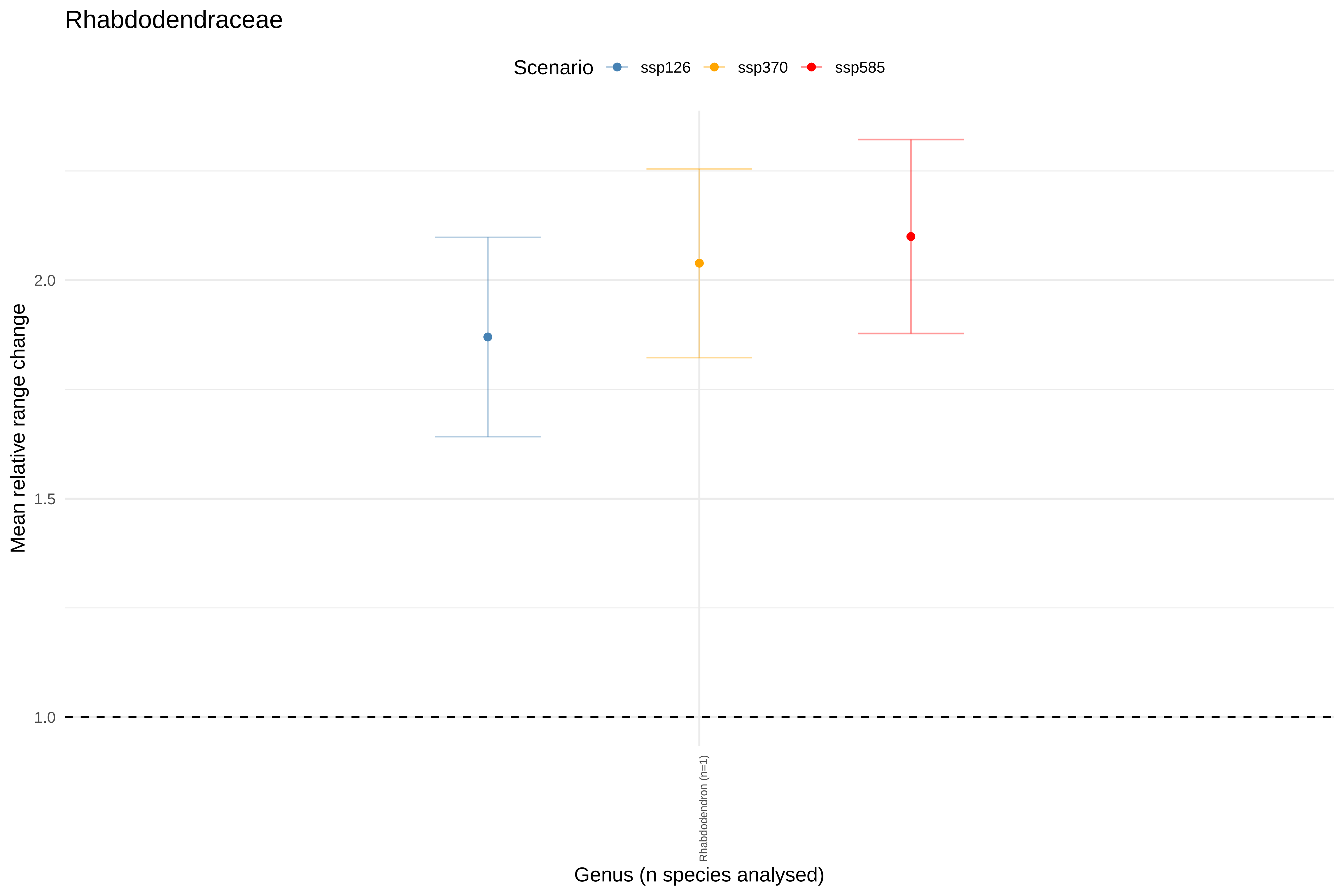

Rhabdodendraceae
Scenario
ssp126
ssp370
ssp585
Mean relative range change
2.0
1.5
1.0
Rhabdodendron (n=1)
Genus (n species analysed)

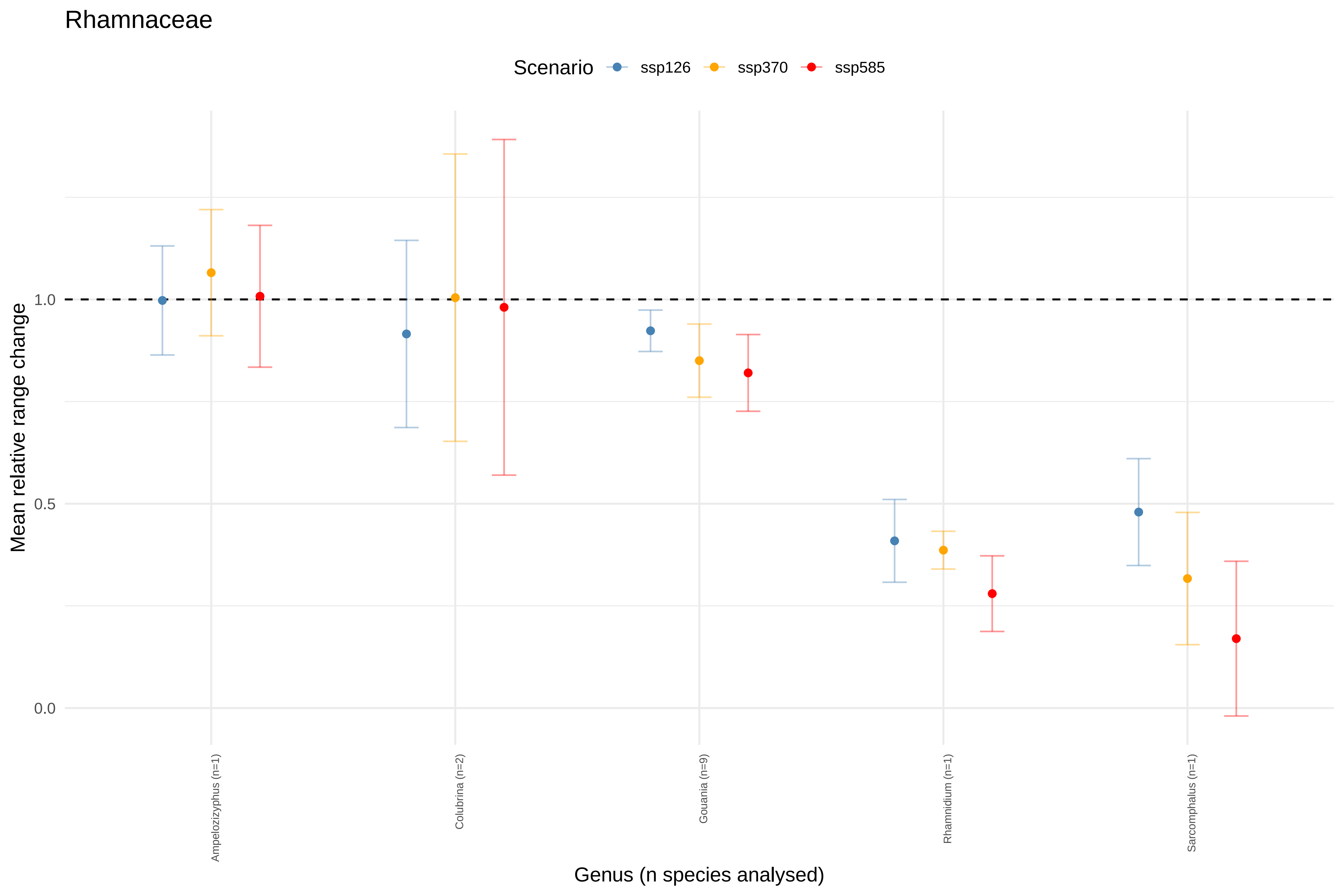

Rhamnaceae
Scenario
ssp126
ssp370
ssp585
Mean relative range change
1.0
0.5
0.0
Ampelozizyphus (n=1)
Colubrina (n=2)
Gouania (n=9)
Rhamnidium (n=1)
Sarcomphalus (n=1)
Genus (n species analysed)

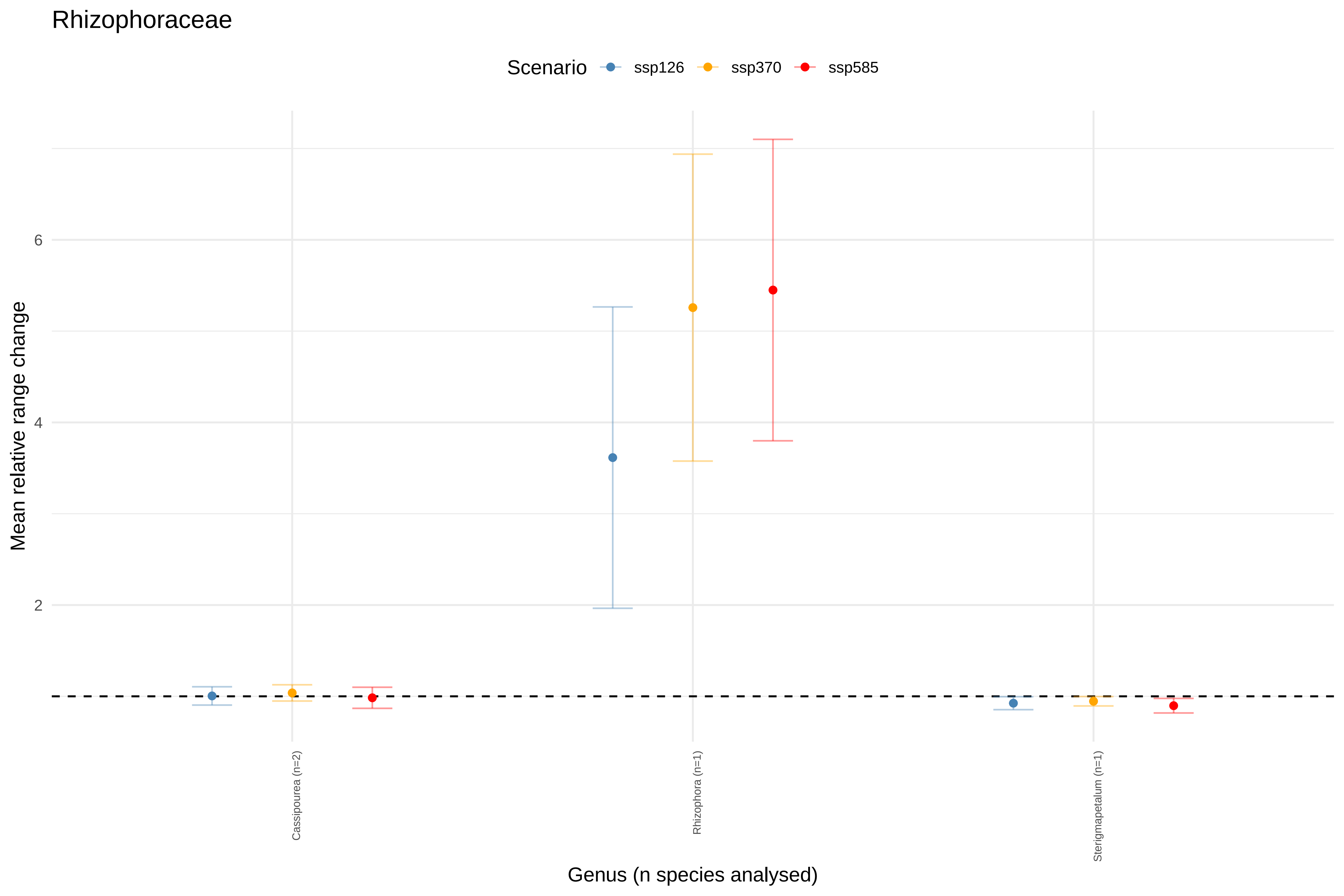
Rhizophoraceae
Scenario
ssp126
ssp370
ssp585
Mean relative range change
6
4
2
Cassipourea (n=2)
Rhizophora (n=1)
Sterigmapetalum (n=1)
Genus (n species analysed)

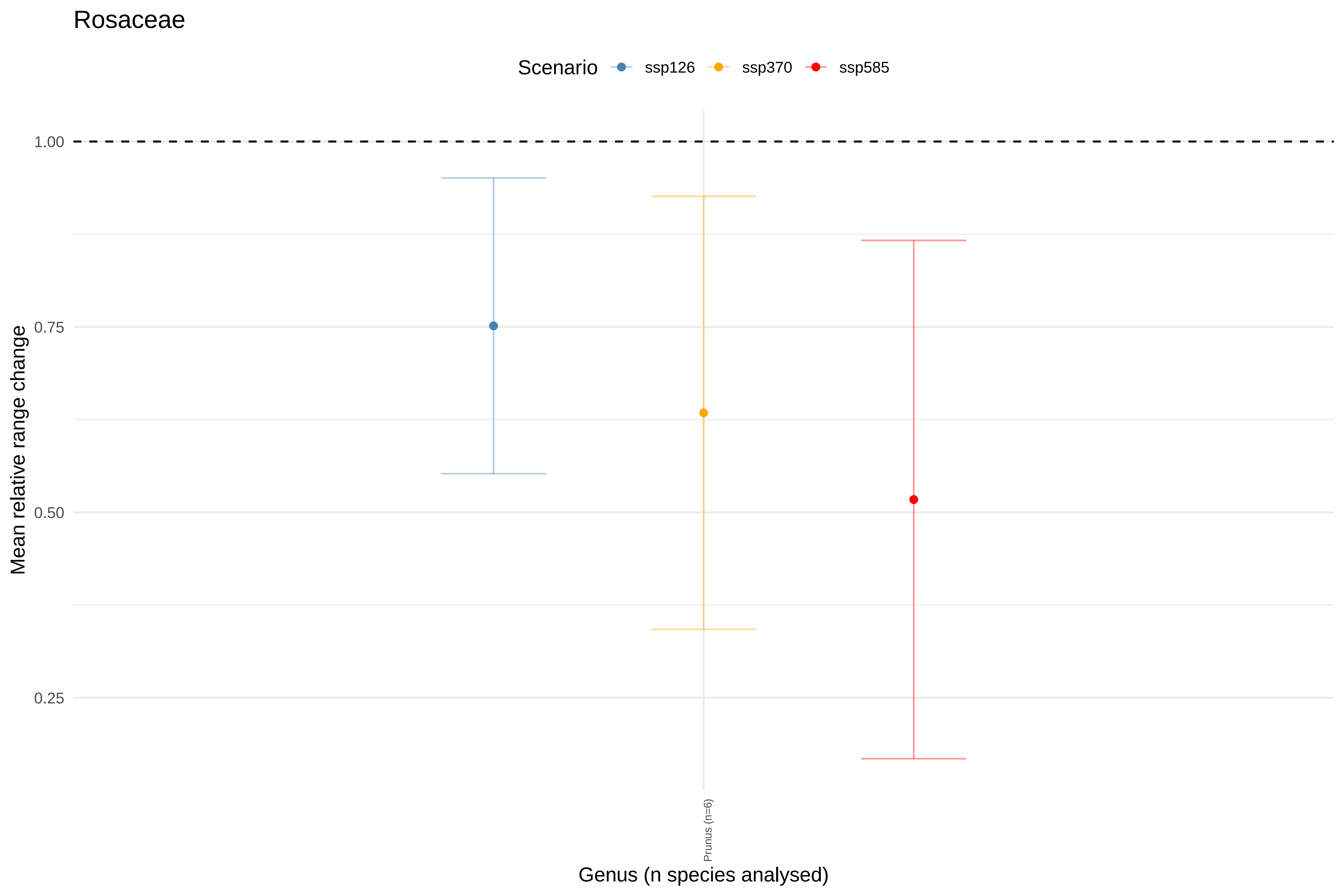
Rosaceae
Scenario
ssp126
ssp370
ssp585
1.00
0.75
0.50
0.25
Mean relative range change
Prunus (n=6)
Genus (n species analysed)

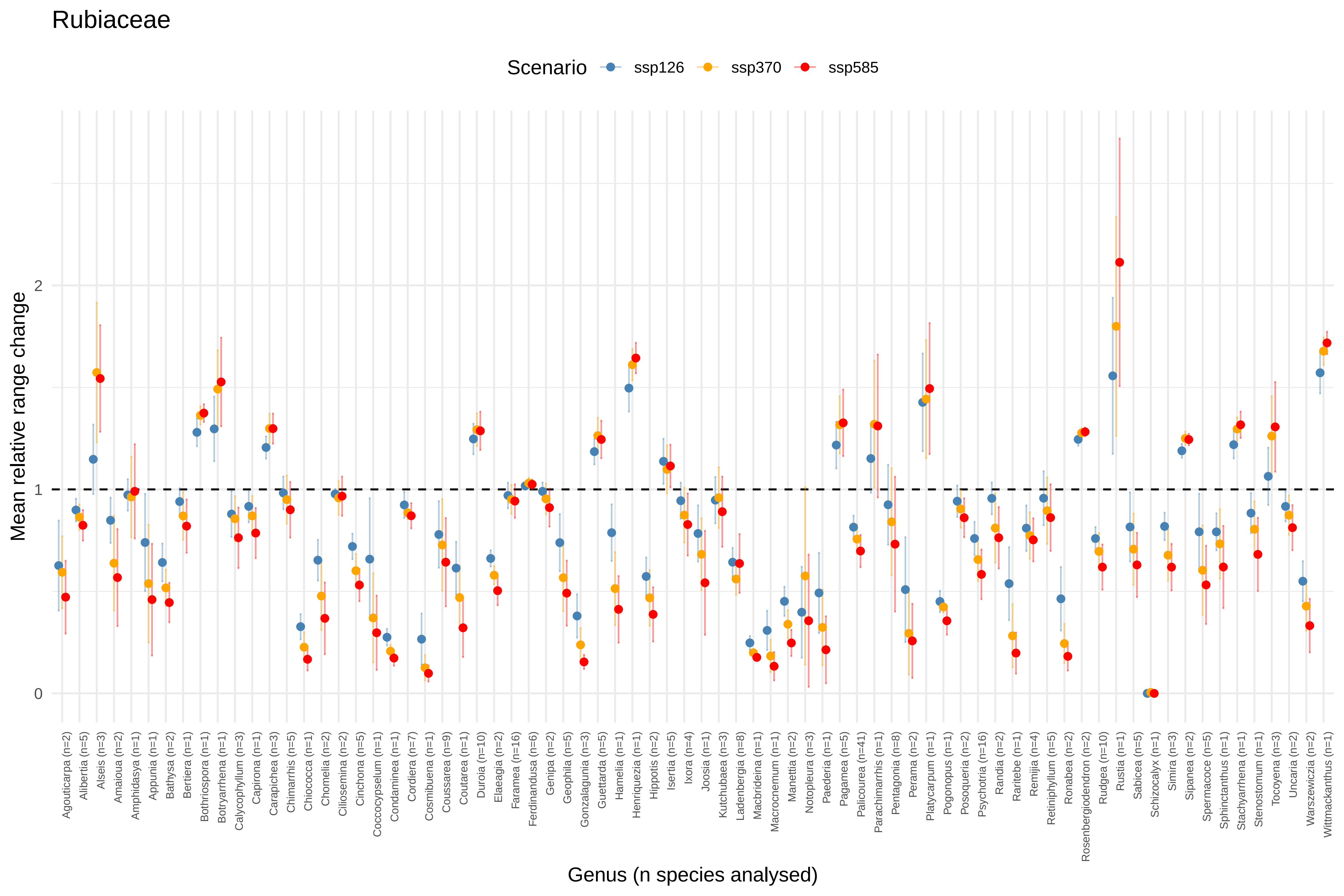
Rubiaceae
Scenario
ssp126
ssp370
ssp585
Mean relative range change
0
1
2
Genus (n species analysed)
Agouticarpa (n=2)
Alibertia (n=5)
Alseis (n=3)
Amaioua (n=2)
Amphidasya (n=1)
Appunia (n=1)
Bathysa (n=2)
Bertiera (n=1)
Bothriospora (n=1)
Botryarrhena (n=1)
Calycophyllum (n=3)
Capirona (n=1)
Carapichea (n=3)
Chimarrhis (n=5)
Chiococca (n=1)
Chomelia (n=2)
Ciliosemina (n=2)
Cinchona (n=5)
Coccocypselum (n=1)
Condaminea (n=1)
Cordiera (n=7)
Cosmibuena (n=1)
Coussarea (n=9)
Coutarea (n=1)
Duroia (n=10)
Elaeagia (n=2)
Faramea (n=16)
Ferdinandusa (n=6)
Genipa (n=2)
Geophila (n=5)
Gonzalagunia (n=3)
Guettarda (n=5)
Hamelia (n=1)
Henriquezia (n=1)
Hippotis (n=2)
Isertia (n=5)
Ixora (n=4)
Joosia (n=1)
Kutchubaea (n=3)
Ladenbergia (n=8)
Macbrideina (n=1)
Macrocnemum (n=1)
Manettia (n=2)
Notopleura (n=3)
Paederia (n=1)
Pagamea (n=5)
Palicourea (n=41)
Parachimarrhis (n=1)
Pentagonia (n=8)
Perama (n=2)
Platycarpum (n=1)
Pogonopus (n=1)
Posoqueria (n=2)
Psychotria (n=16)
Randia (n=2)
Raritebe (n=1)
Remijia (n=4)
Retiniphyllum (n=5)
Ronabea (n=2)
Rosenbergiodendron (n=2)
Rudgea (n=10)
Rustia (n=1)
Sabicea (n=5)
Schizocalyx (n=1)
Simira (n=3)
Sipanea (n=2)
Spermacoce (n=5)
Sphinctanthus (n=1)
Stachyarrhena (n=1)
Stenostomum (n=1)
Tocoyena (n=3)
Uncaria (n=2)
Warszewiczia (n=2)
Wittmackanthus (n=1)

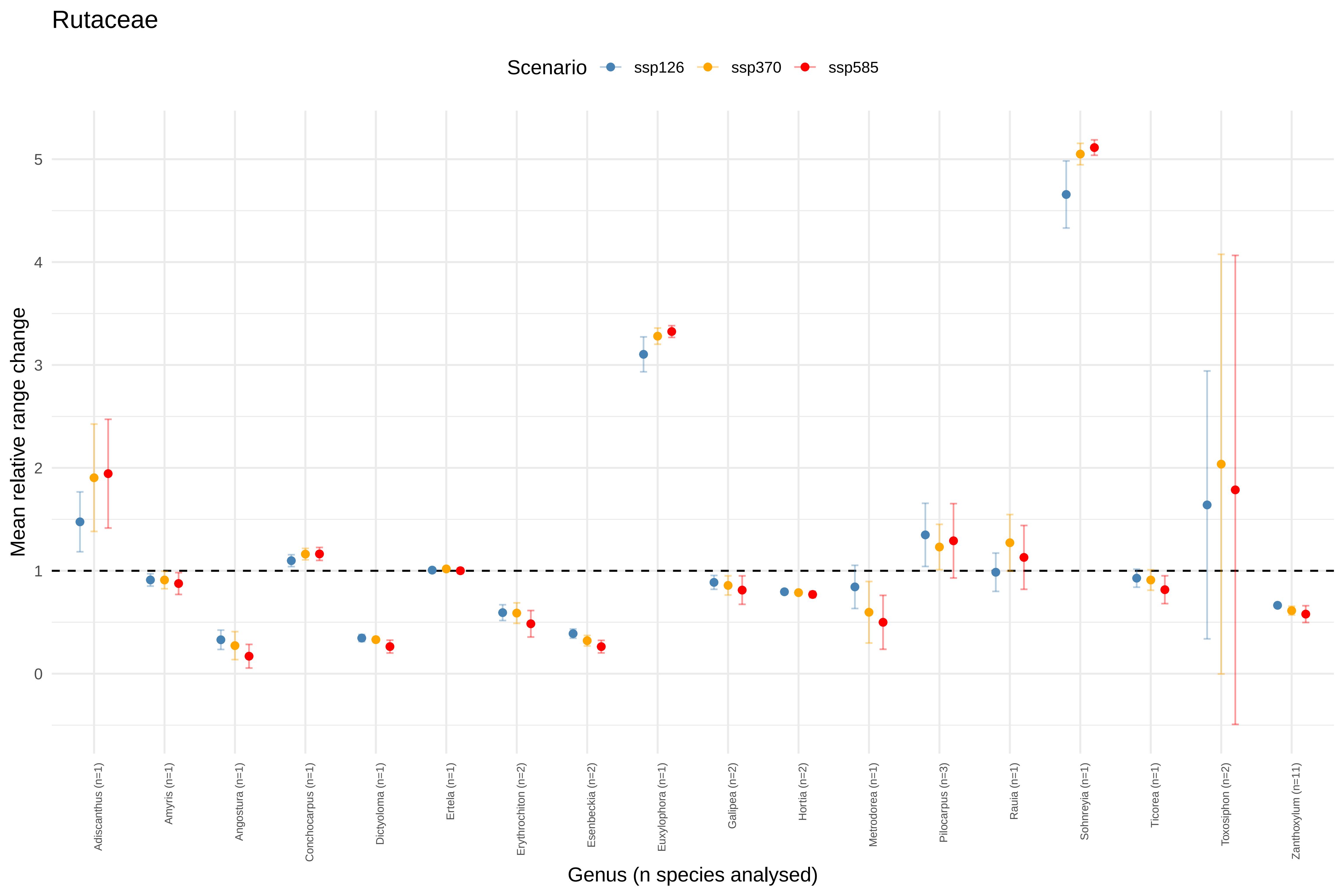

Rutaceae
Scenario
ssp126
ssp370
ssp585
Mean relative range change
0
1
2
3
4
5
Adiscanthus (n=1)
Amyris (n=1)
Angostura (n=1)
Conchocarpus (n=1)
Dictyoloma (n=1)
Ertela (n=1)
Erythrochiton (n=2)
Esenbeckia (n=2)
Euxylophora (n=1)
Galipea (n=2)
Hortia (n=2)
Metrodorea (n=1)
Pilocarpus (n=3)
Rauia (n=1)
Sohnreyia (n=1)
Ticorea (n=1)
Toxosiphon (n=2)
Zanthoxylum (n=11)
Genus (n species analysed)

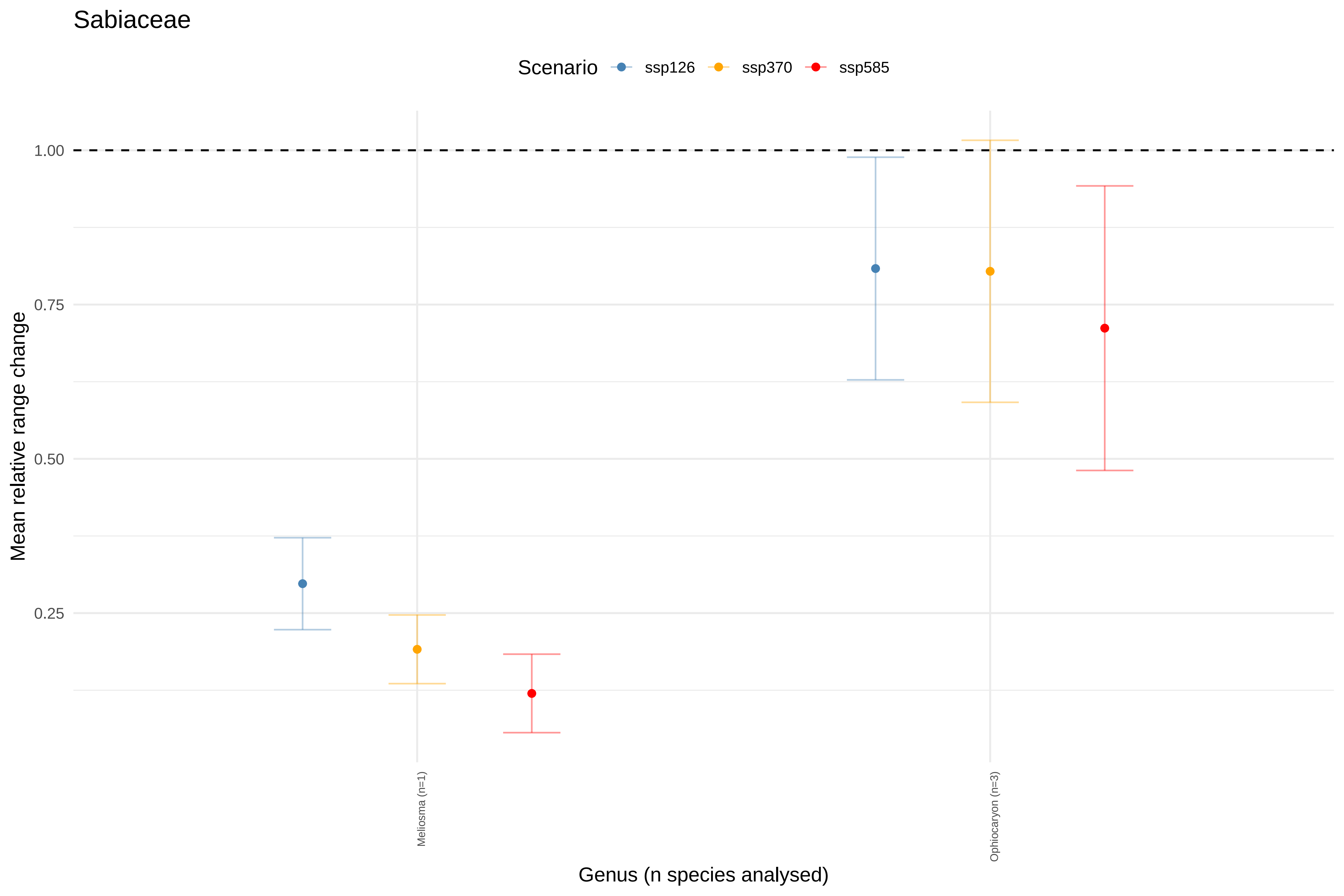

Sabiaceae
Scenario
ssp126
ssp370
ssp585
Mean relative range change
1.00
0.75
0.50
0.25
Meliosma (n=1)
Ophiocaryon (n=3)
Genus (n species analysed)

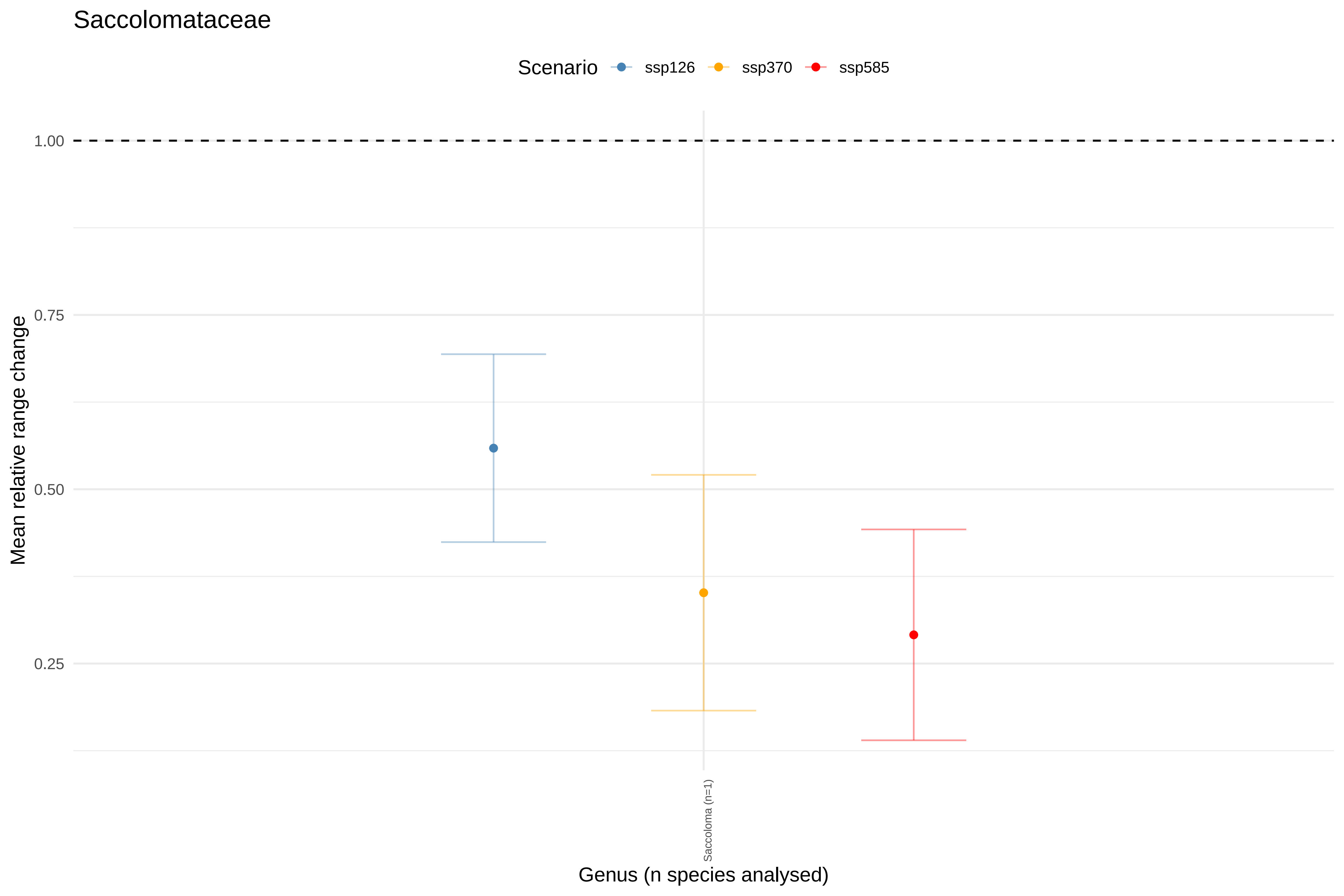
Saccolomataceae
Scenario
ssp126
ssp370
ssp585
1.00
0.75
0.50
0.25
Mean relative range change
Saccoloma (n=1)
Genus (n species analysed)

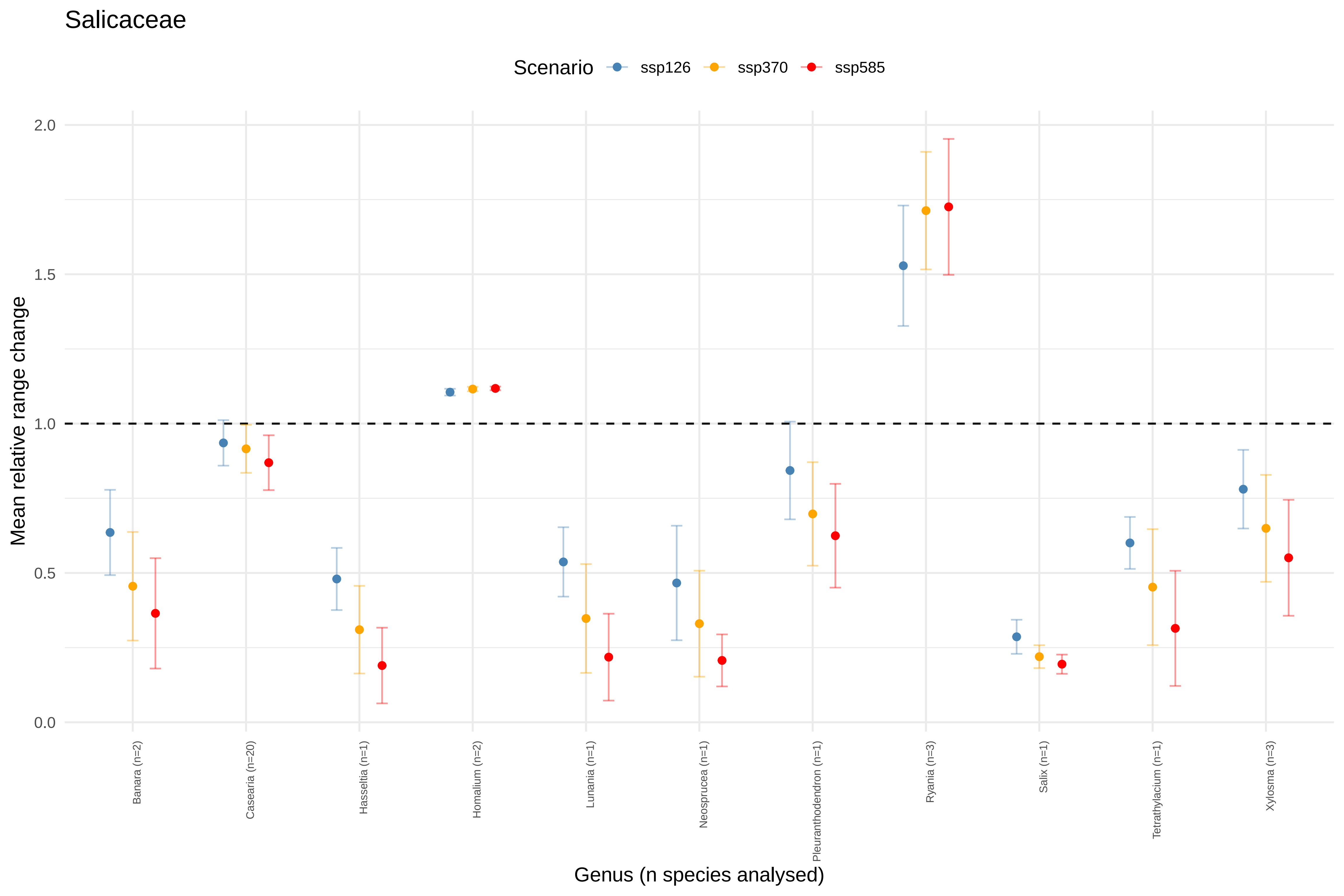

Salicaceae
Scenario
ssp126
ssp370
ssp585
Mean relative range change
2.0
1.5
1.0
0.5
0.0
Banara (n=2)
Casearia (n=20)
Hasseltia (n=1)
Homalium (n=2)
Lunania (n=1)
Neosprucea (n=1)
Pleuranthodendron (n=1)
Ryania (n=3)
Salix (n=1)
Tetrathylacium (n=1)
Xylosma (n=3)
Genus (n species analysed)

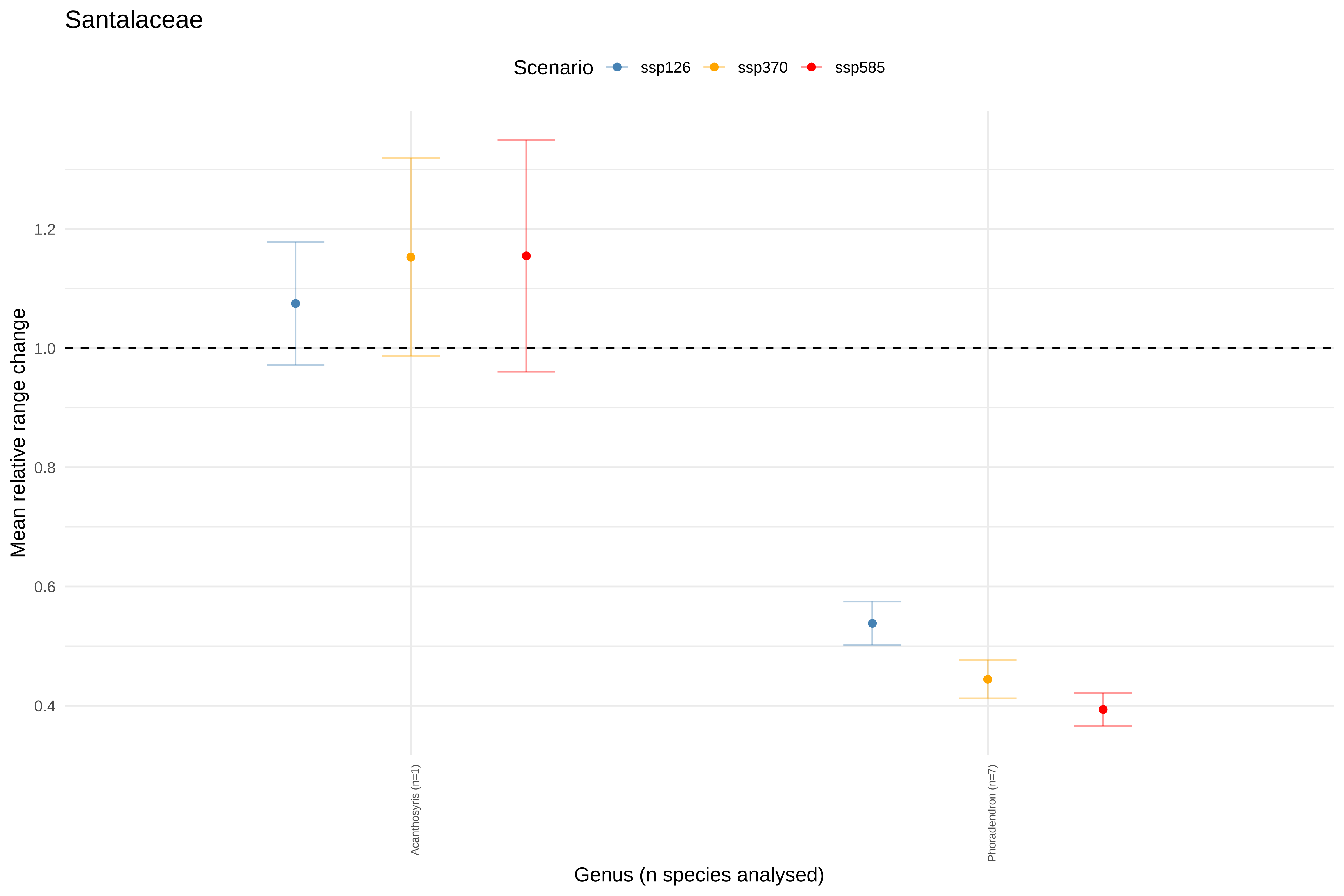

Santalaceae
Scenario
ssp126
ssp370
ssp585
Mean relative range change
1.2
1.0
0.8
0.6
0.4
Acanthosyris (n=1)
Phoradendron (n=7)
Genus (n species analysed)

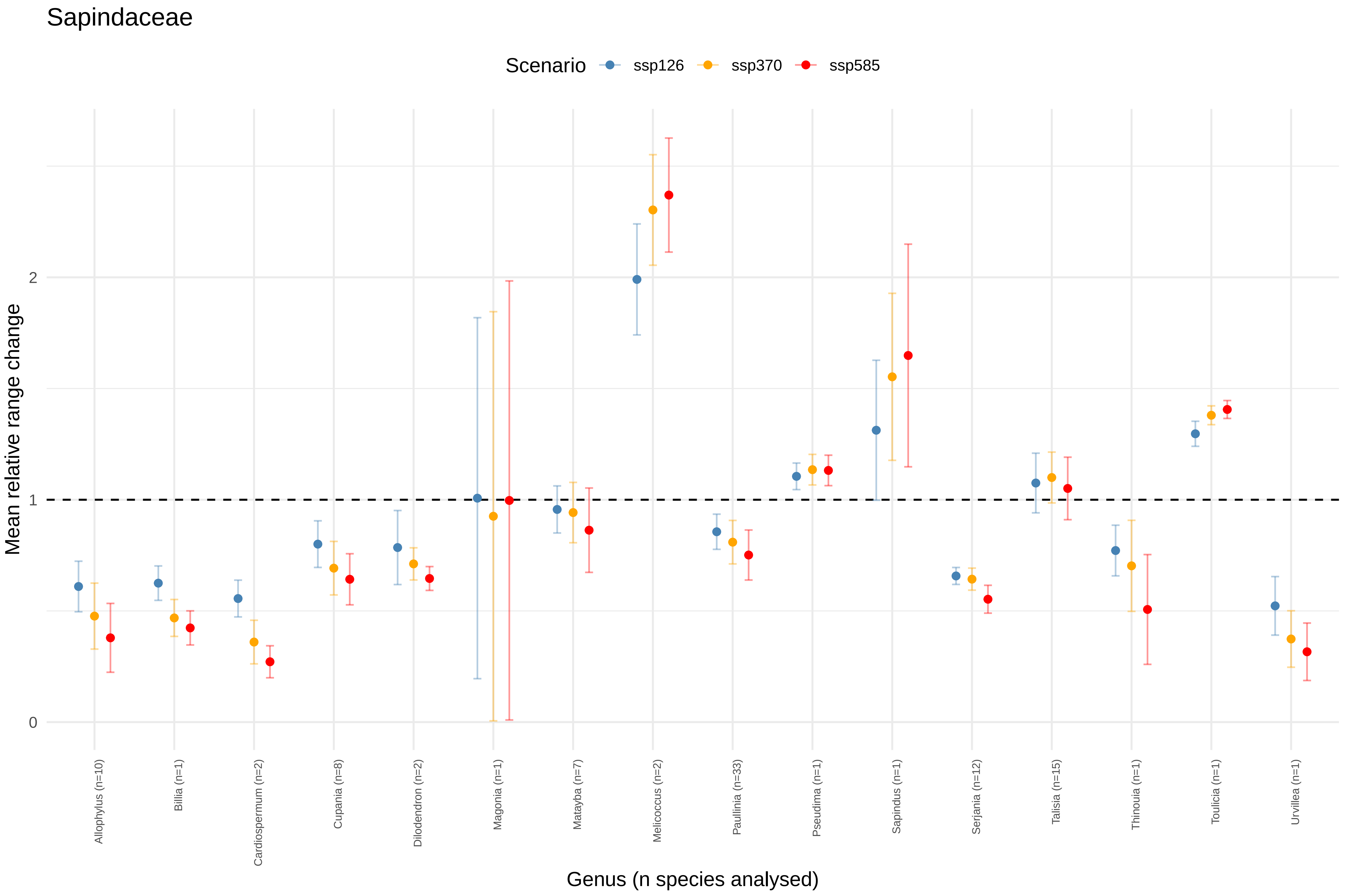

Sapindaceae
Scenario
ssp126
ssp370
ssp585
Mean relative range change
0
1
2
Allophylus (n=10)
Billia (n=1)
Cardiospermum (n=2)
Cupania (n=8)
Dilodendron (n=2)
Magonia (n=1)
Matayba (n=7)
Melicoccus (n=2)
Paullinia (n=33)
Pseudima (n=1)
Sapindus (n=1)
Serjania (n=12)
Talisia (n=15)
Thinouia (n=1)
Toulicia (n=1)
Urvillea (n=1)
Genus (n species analysed)

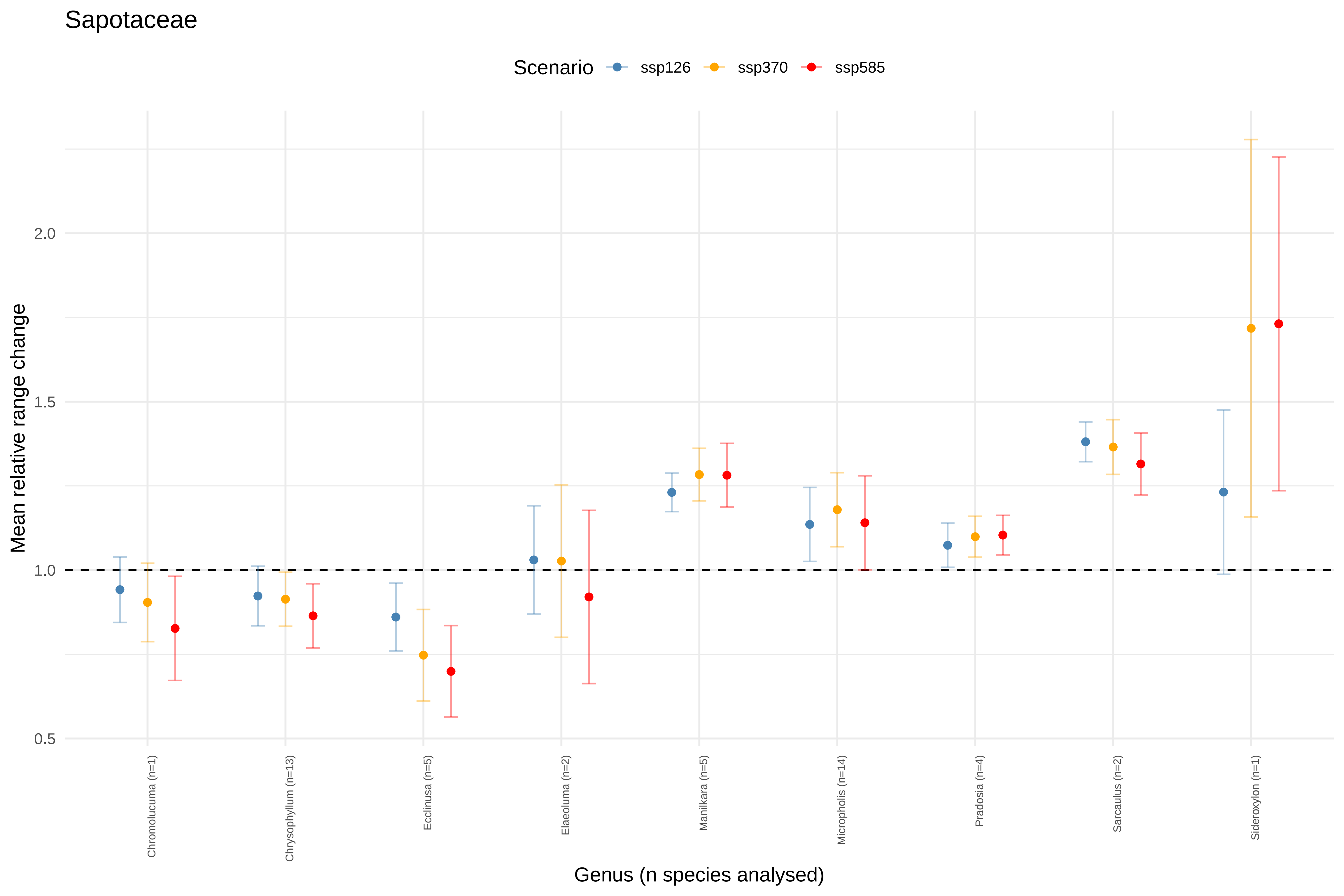
Sapotaceae
Scenario
ssp126
ssp370
ssp585
Mean relative range change
2.0
1.5
1.0
0.5
Chromolucuma (n=1)
Chrysophyllum (n=13)
Ecclinusa (n=5)
Elaeoluma (n=2)
Manilkara (n=5)
Micropholis (n=14)
Pradosia (n=4)
Sarcaulus (n=2)
Sideroxylon (n=1)
Genus (n species analysed)

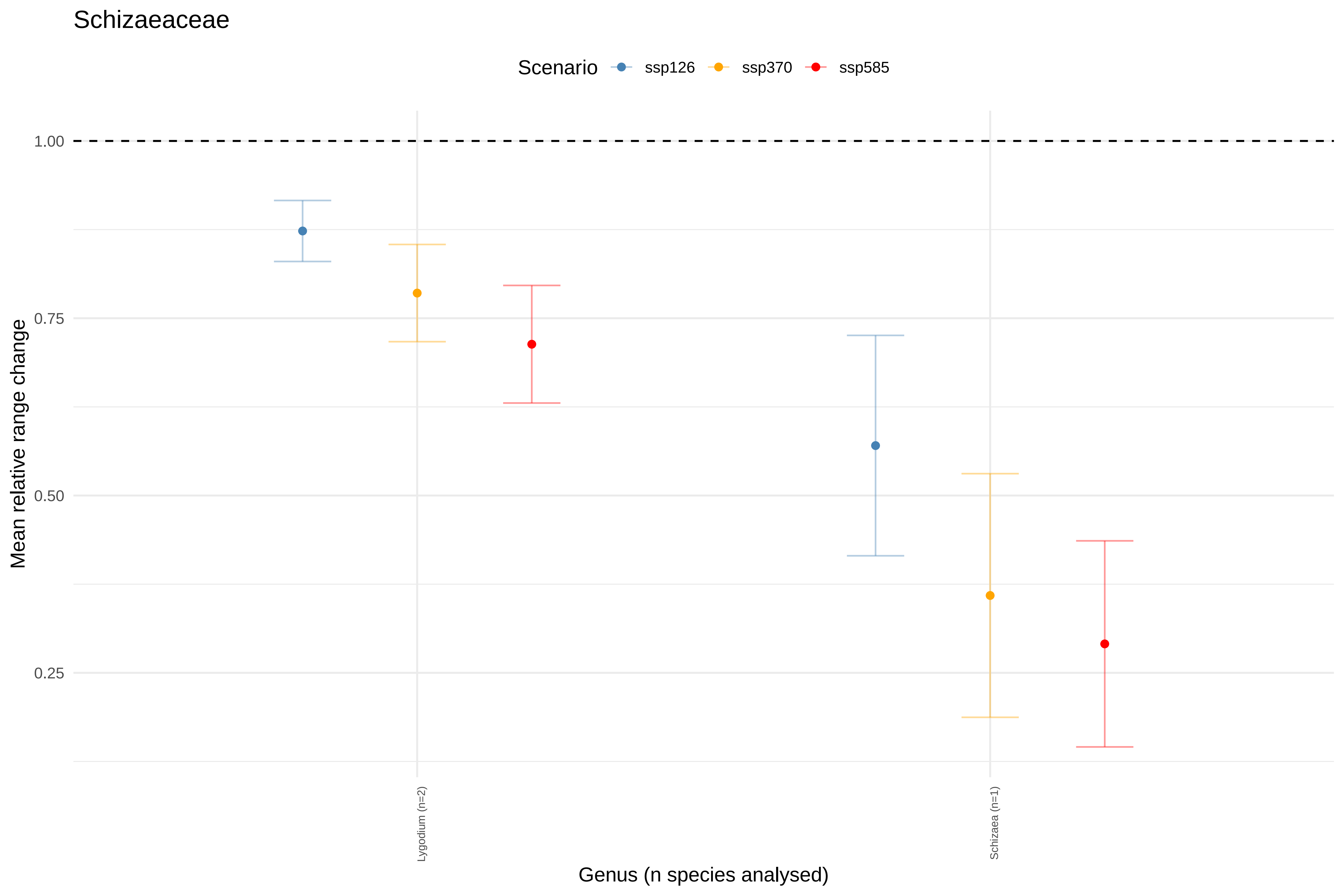

Schizaeaceae
Scenario
ssp126
ssp370
ssp585
1.00
0.75
0.50
0.25
Mean relative range change
Lygodium (n=2)
Schizaea (n=1)
Genus (n species analysed)

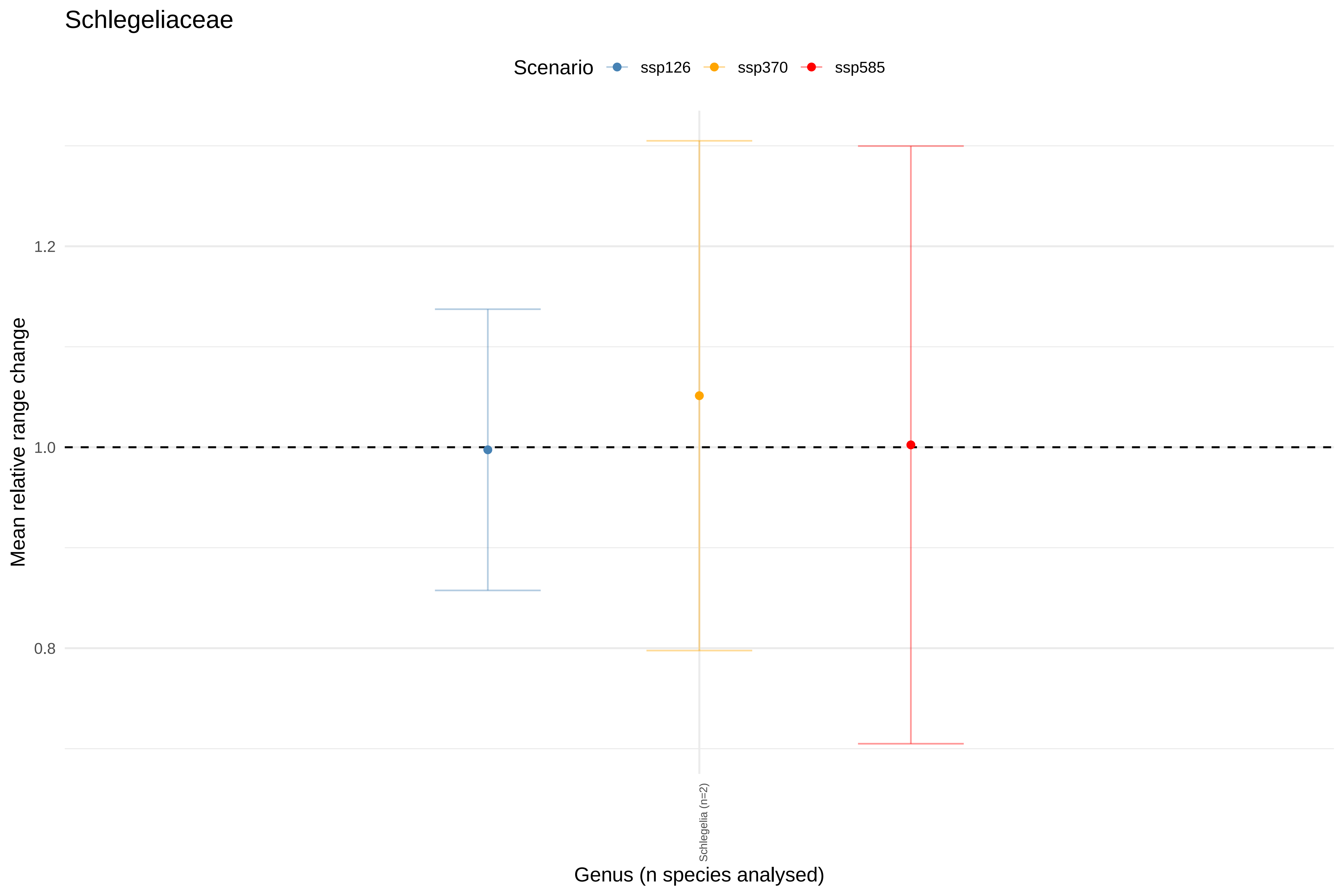

Schlegeliaceae
Scenario
ssp126
ssp370
ssp585
Mean relative range change
1.2
1.0
0.8
Schlegelia (n=2)
Genus (n species analysed)

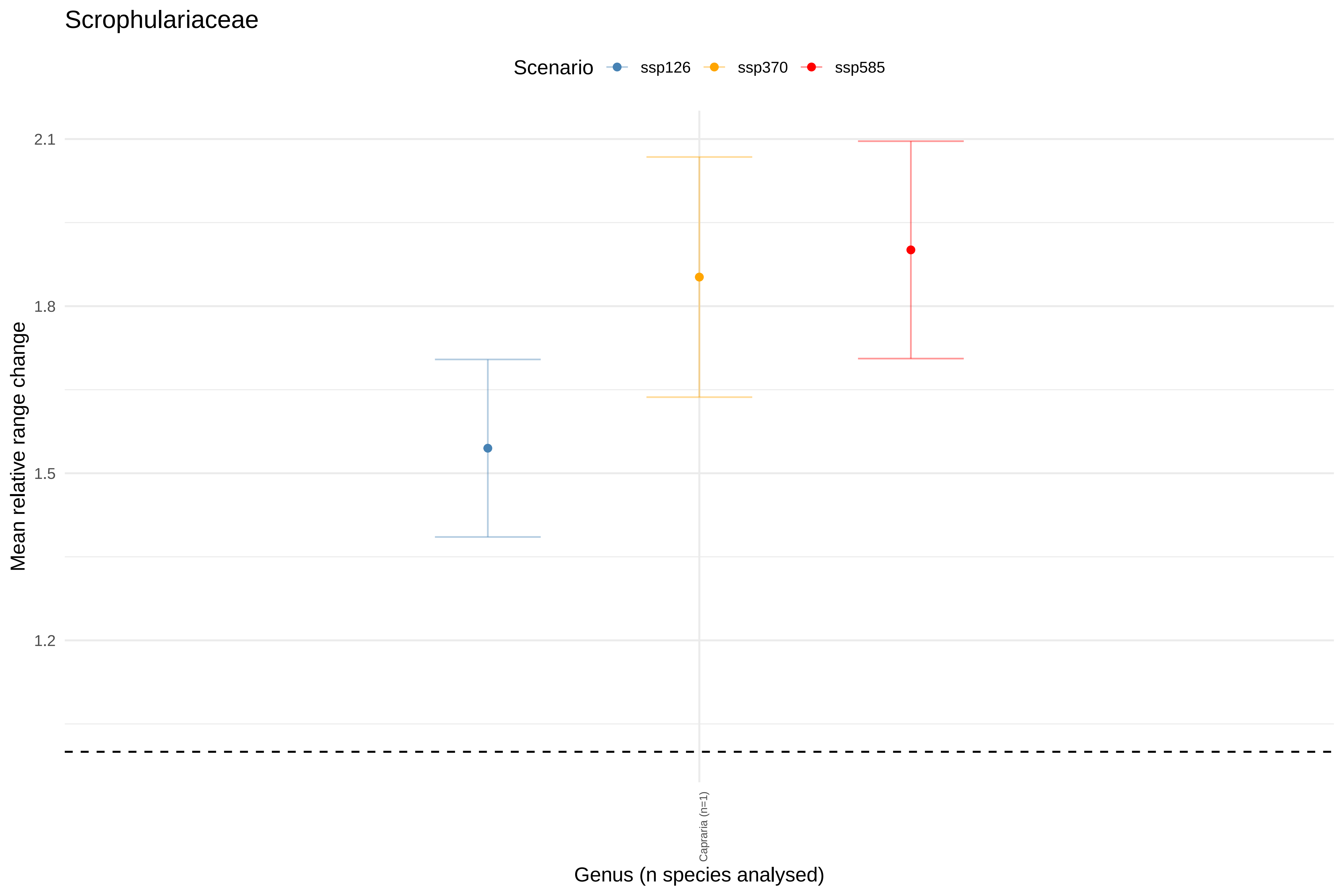

Scrophulariaceae
Scenario
ssp126
ssp370
ssp585
2.1
1.8
1.5
1.2
Mean relative range change
Capraria (n=1)
Genus (n species analysed)

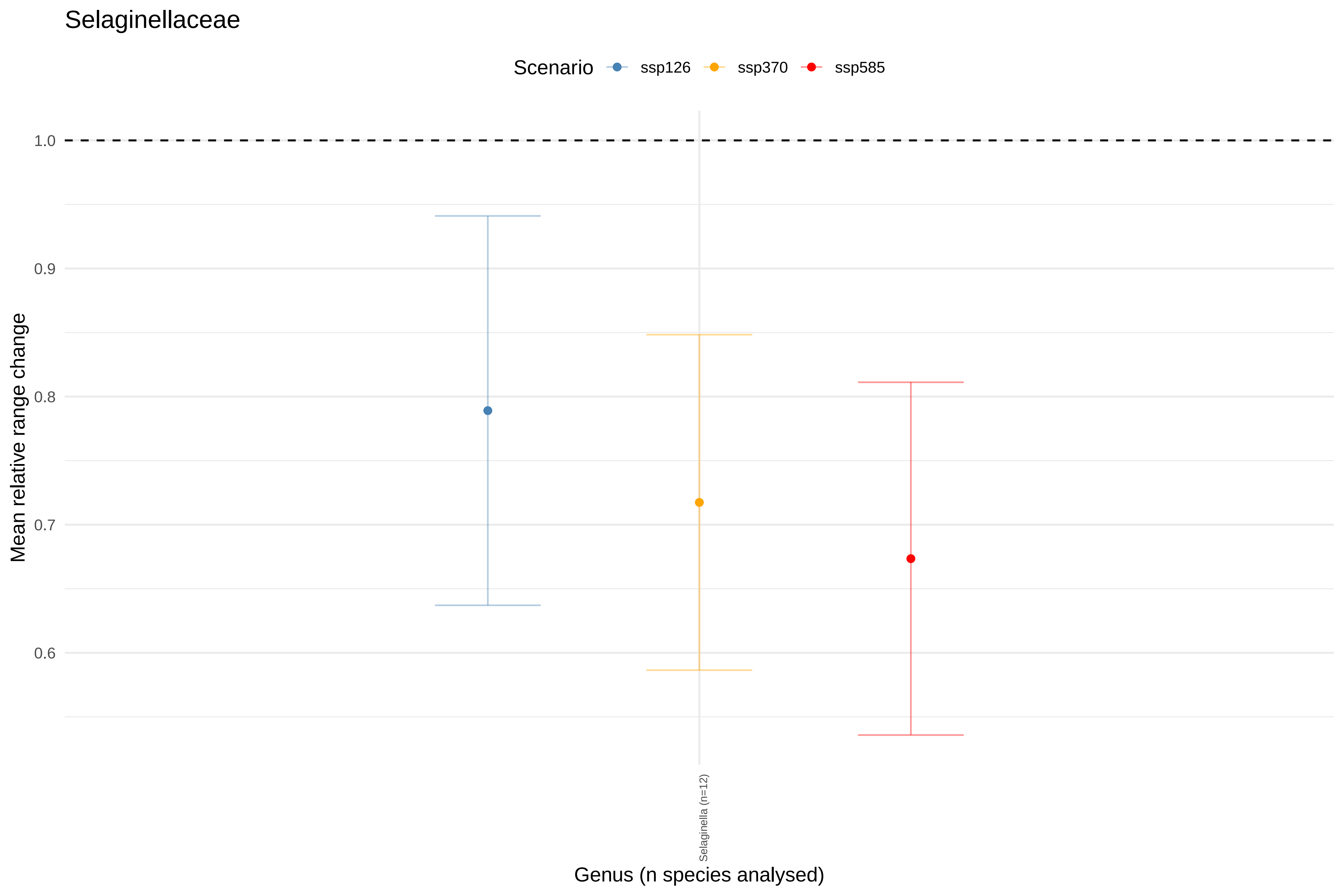
Selaginellaceae
Scenario
ssp126
ssp370
ssp585
1.0
0.9
0.8
0.7
0.6
Mean relative range change
Selaginella (n=12)
Genus (n species analysed)

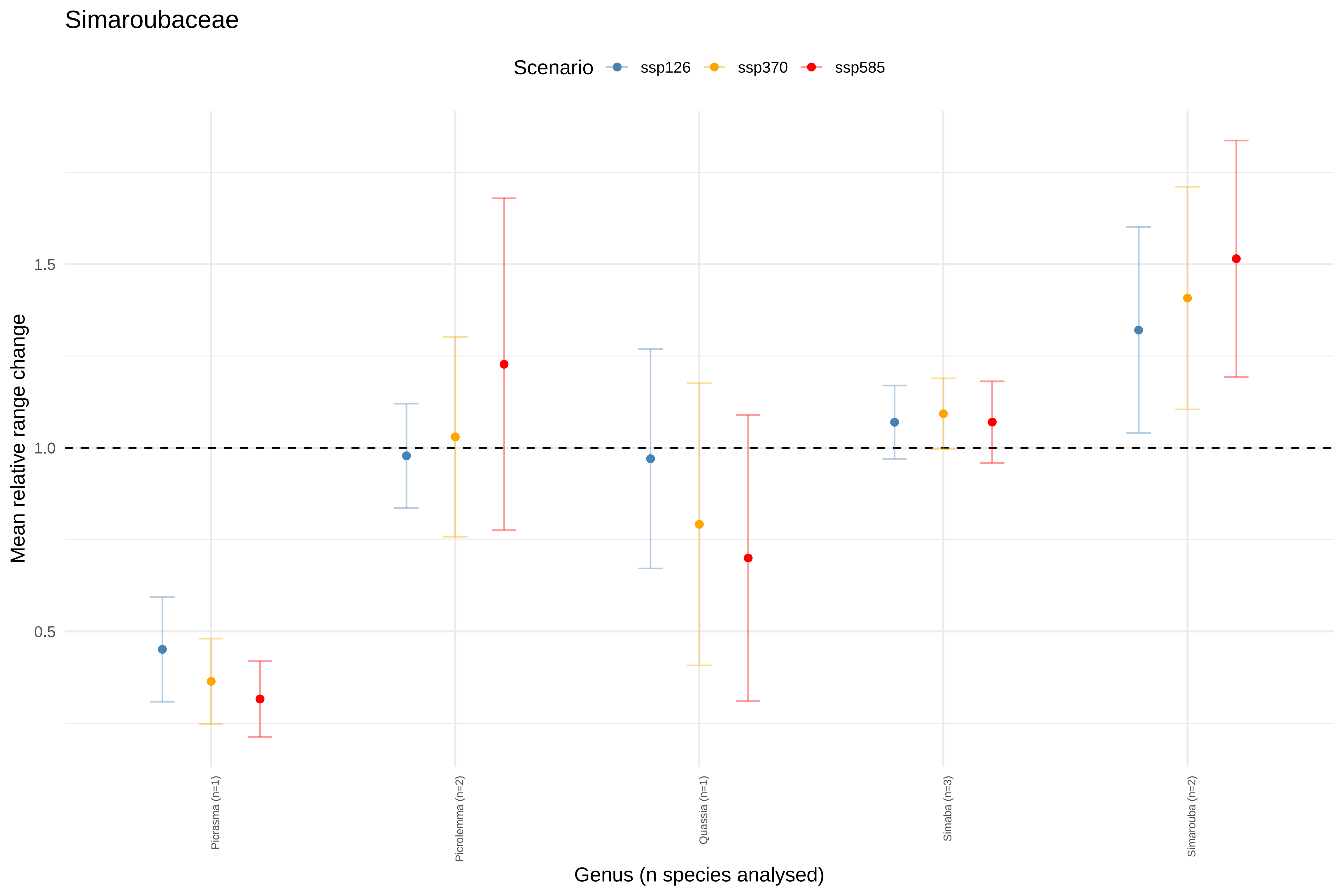

Simaroubaceae
Scenario
ssp126
ssp370
ssp585
Mean relative range change
1.5
1.0
0.5
Picrasma (n=1)
Picrolemma (n=2)
Quassia (n=1)
Simaba (n=3)
Simarouba (n=2)
Genus (n species analysed)

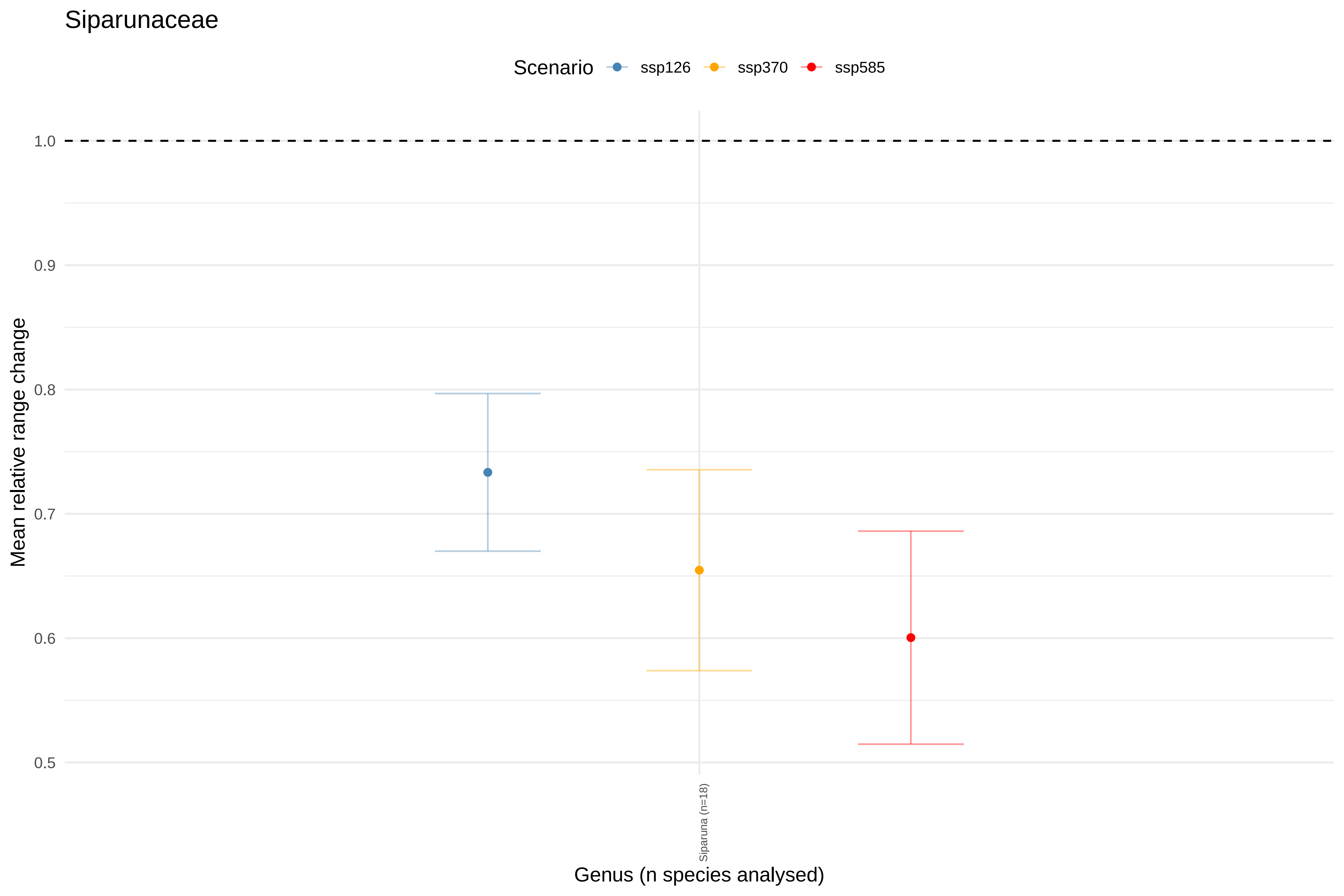
Siparunaceae
Scenario
ssp126
ssp370
ssp585
Mean relative range change
1.0
0.9
0.8
0.7
0.6
0.5
Siparuna (n=18)
Genus (n species analysed)

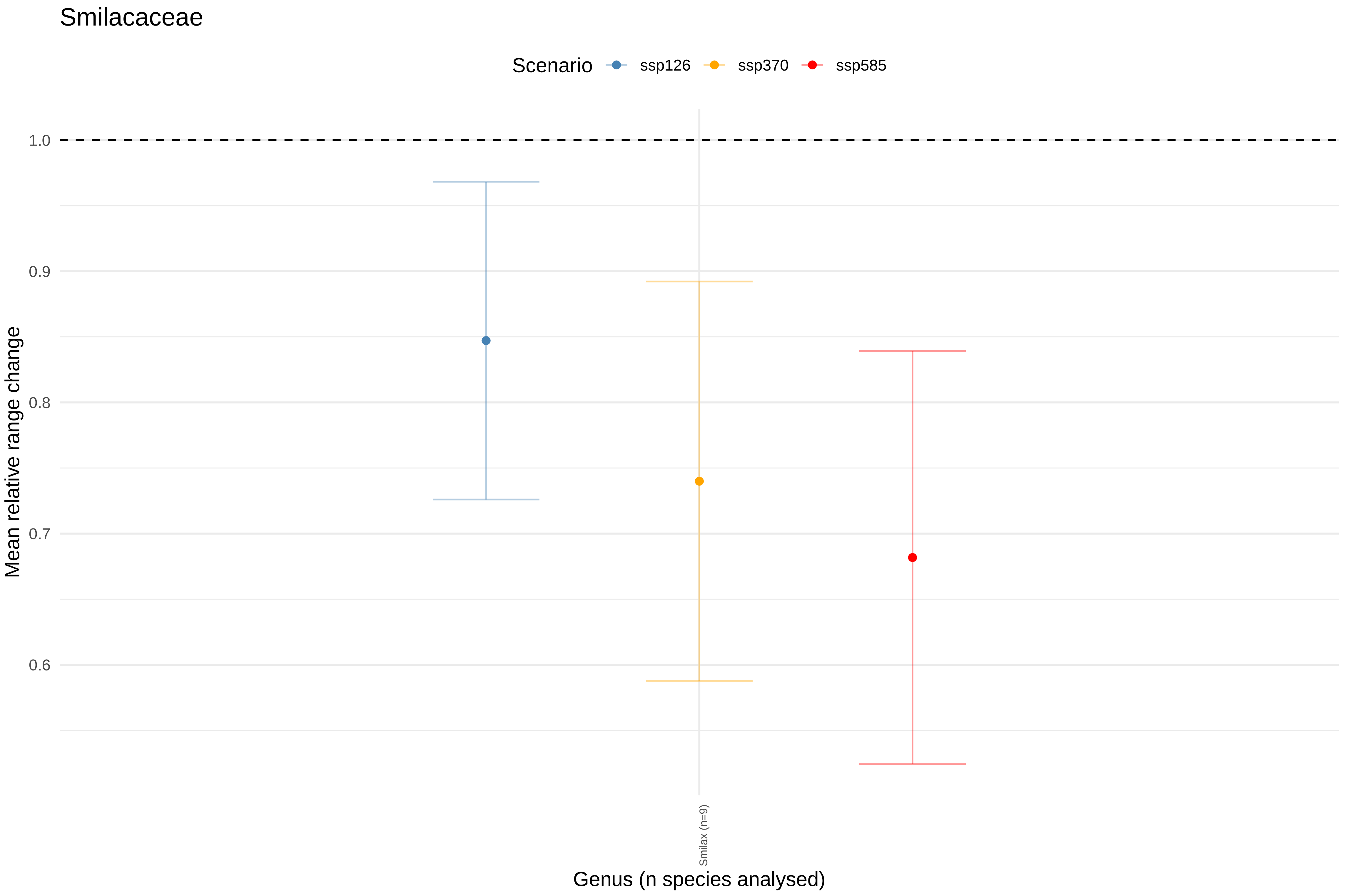

Smilacaceae
Scenario
ssp126
ssp370
ssp585
1.0
0.9
0.8
0.7
0.6
Mean relative range change
Smilax (n=9)
Genus (n species analysed)

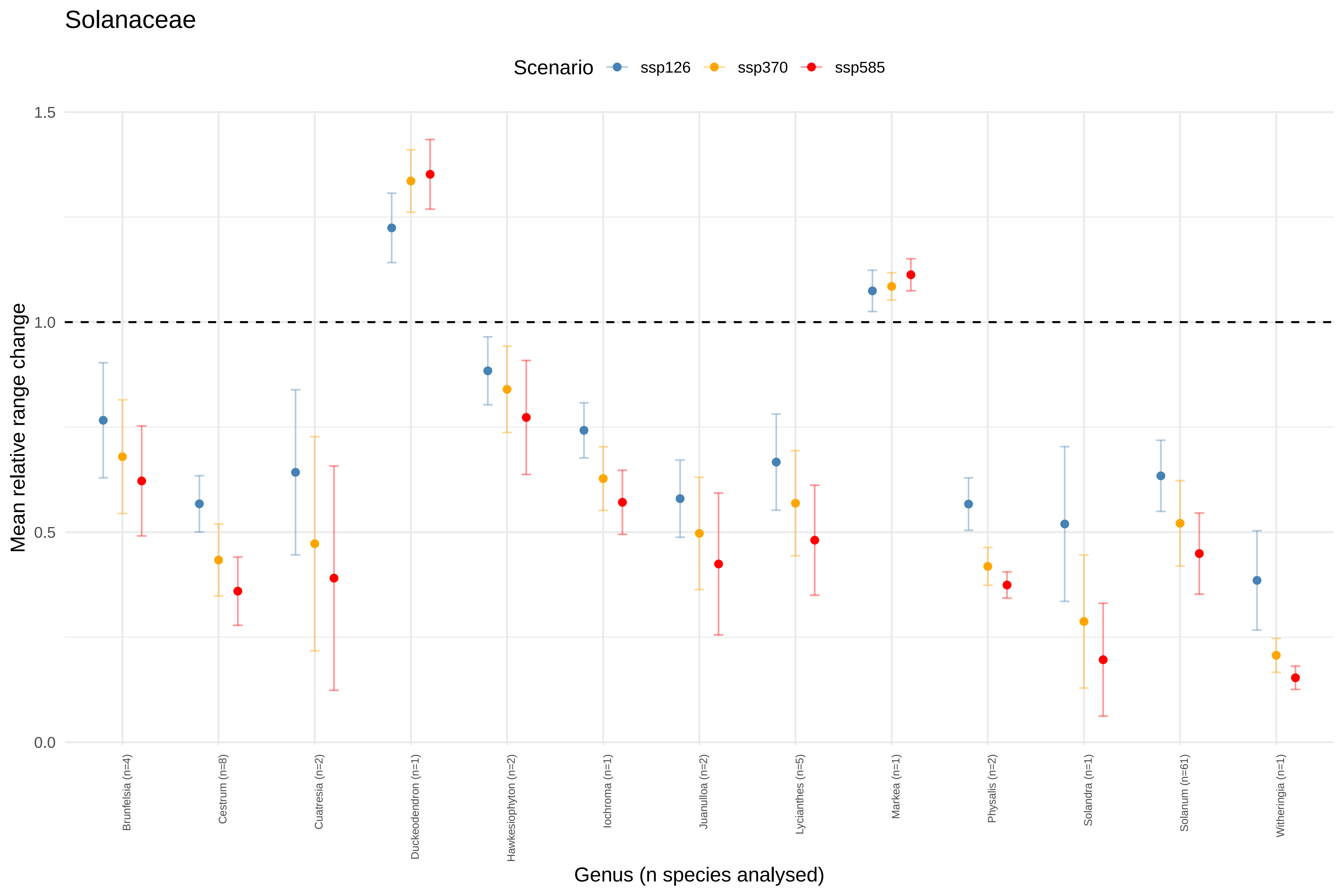

Solanaceae
Scenario
ssp126
ssp370
ssp585
Mean relative range change
1.5
1.0
0.5
0.0
Brunfelsia (n=4)
Cestrum (n=8)
Cuatresia (n=2)
Duckeodendron (n=1)
Hawkesiophyton (n=2)
Iochroma (n=1)
Juanulloa (n=2)
Lycianthes (n=5)
Markea (n=1)
Physalis (n=2)
Solandra (n=1)
Solanum (n=61)
Witheringia (n=1)
Genus (n species analysed)

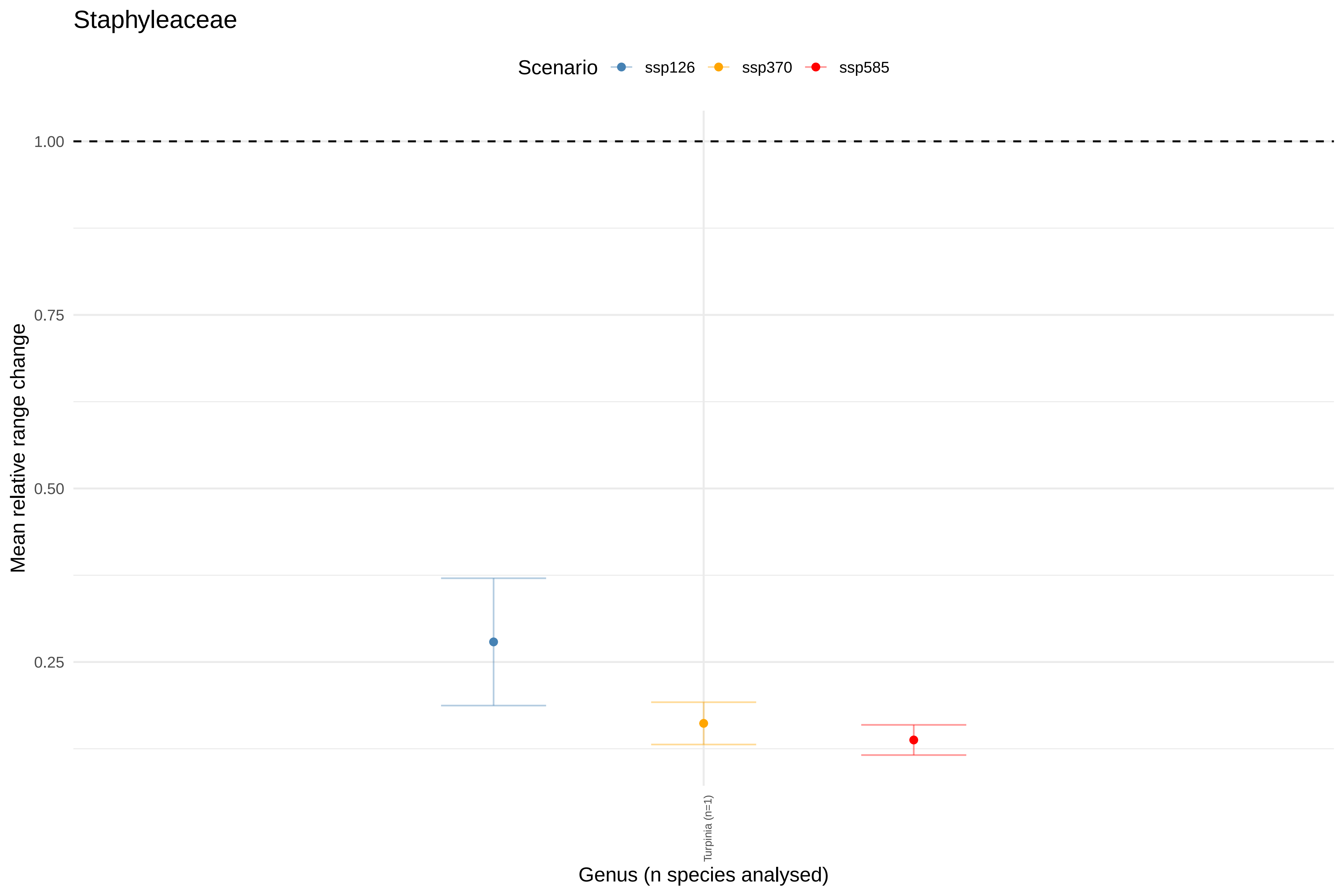

Staphyleaceae
Scenario
ssp126
ssp370
ssp585
1.00
0.75
0.50
0.25
Mean relative range change
Turpinia (n=1)
Genus (n species analysed)

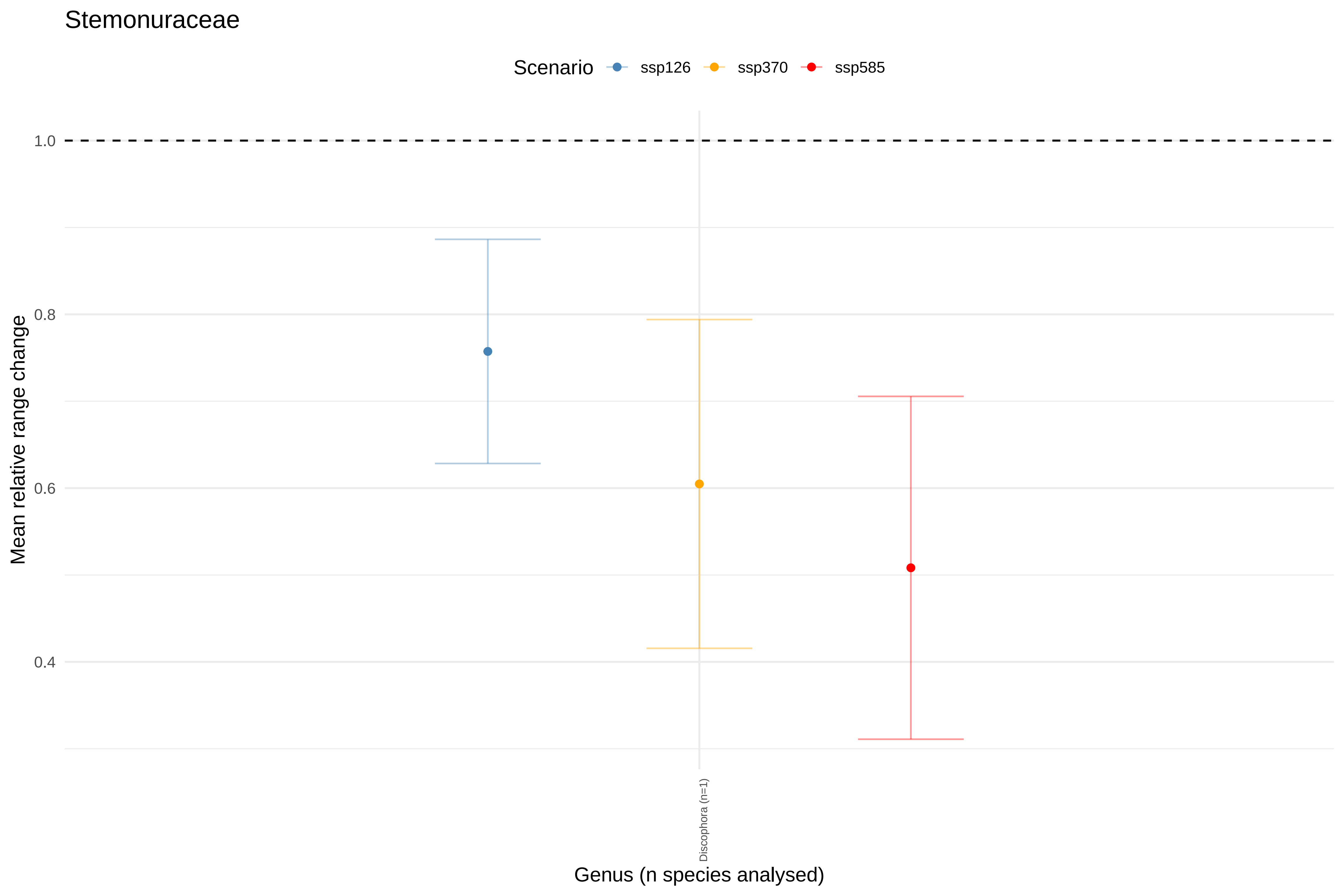

Stemonuraceae
Scenario
ssp126
ssp370
ssp585
1.0
0.8
0.6
0.4
Mean relative range change
Discophora (n=1)
Genus (n species analysed)

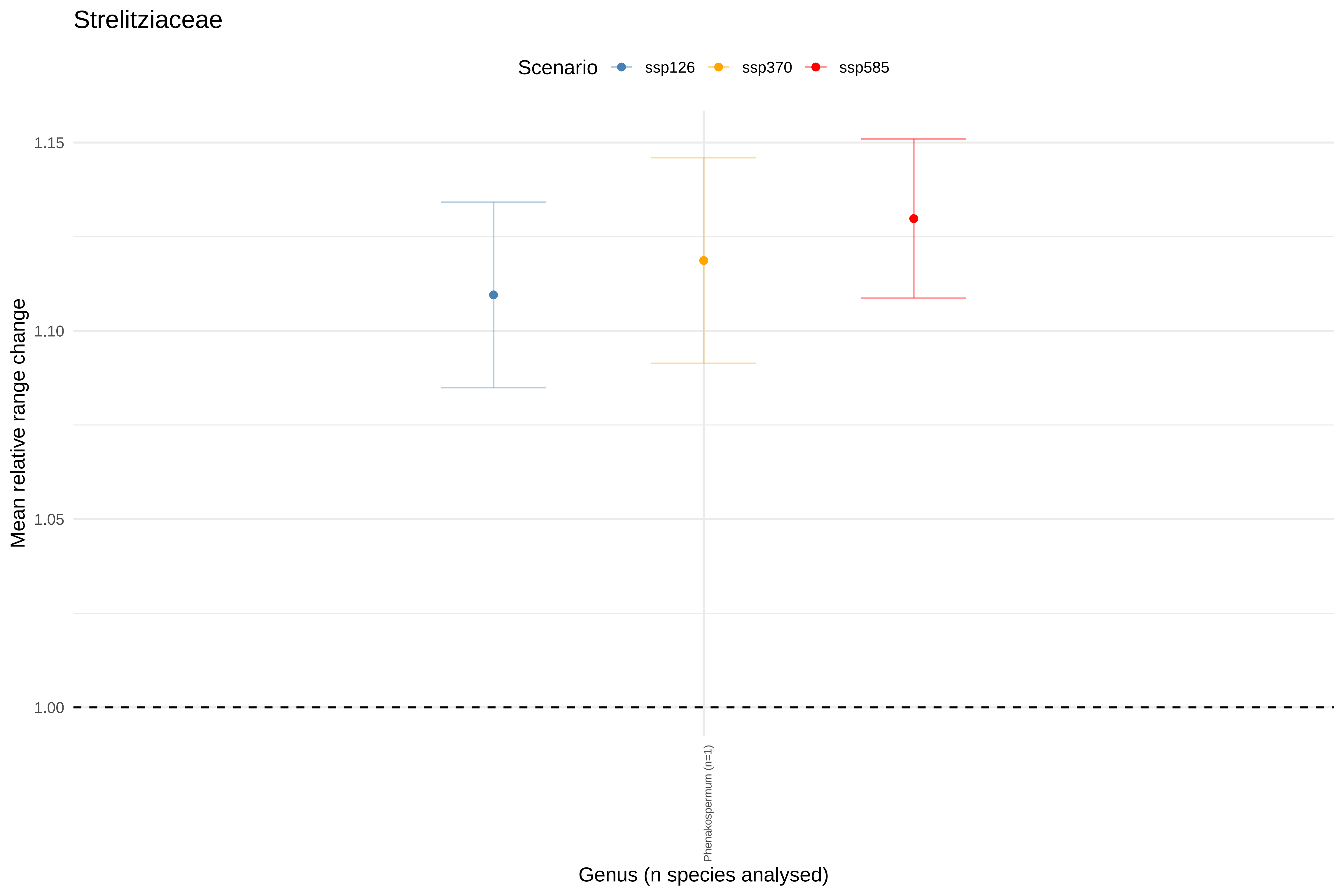

Strelitziaceae
Scenario
ssp126
ssp370
ssp585
Mean relative range change
1.15
1.10
1.05
1.00
Phenakospermum (n=1)
Genus (n species analysed)

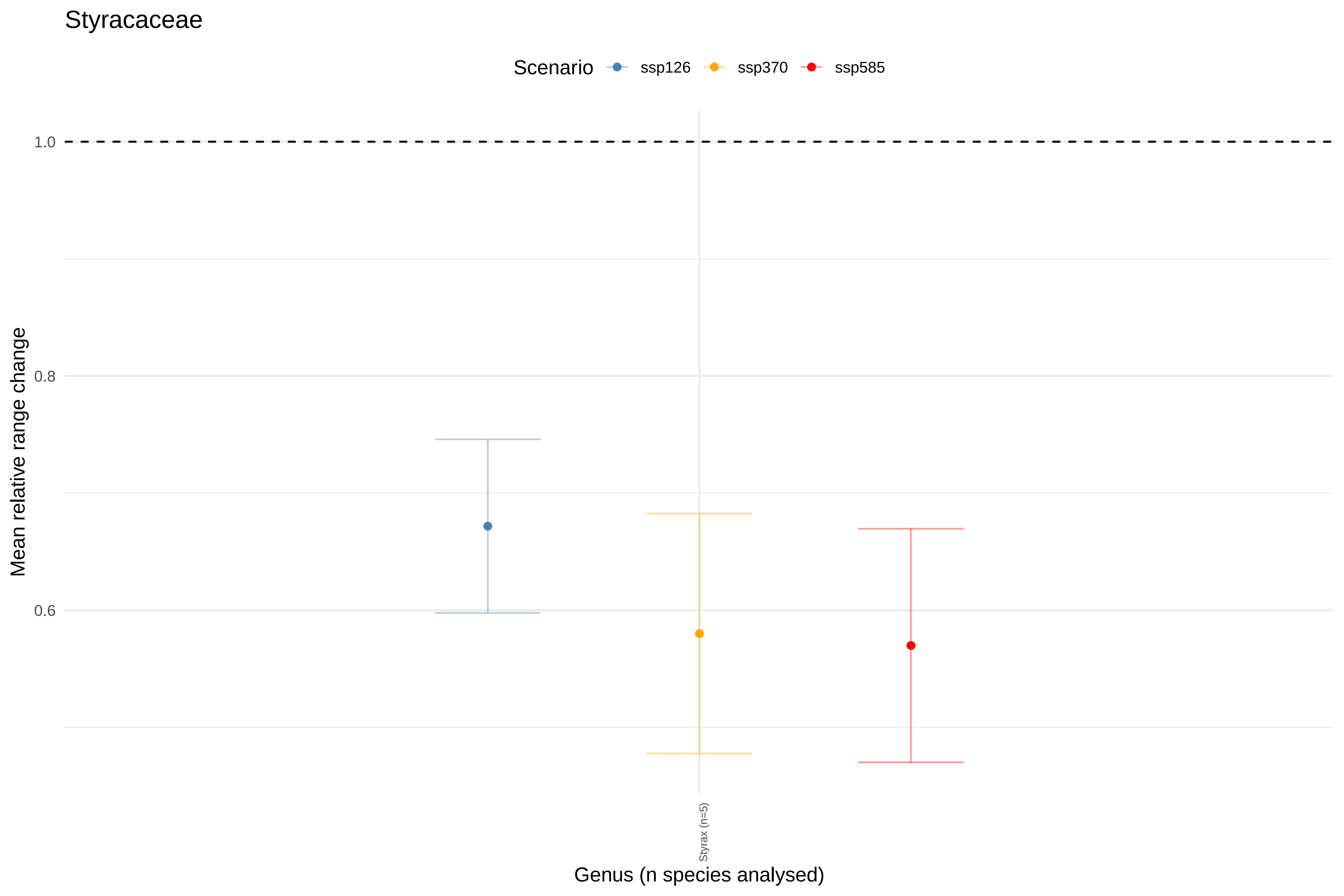

Styracaceae
Scenario
ssp126
ssp370
ssp585
1.0
0.8
0.6
Mean relative range change
Styrax (n=5)
Genus (n species analysed)

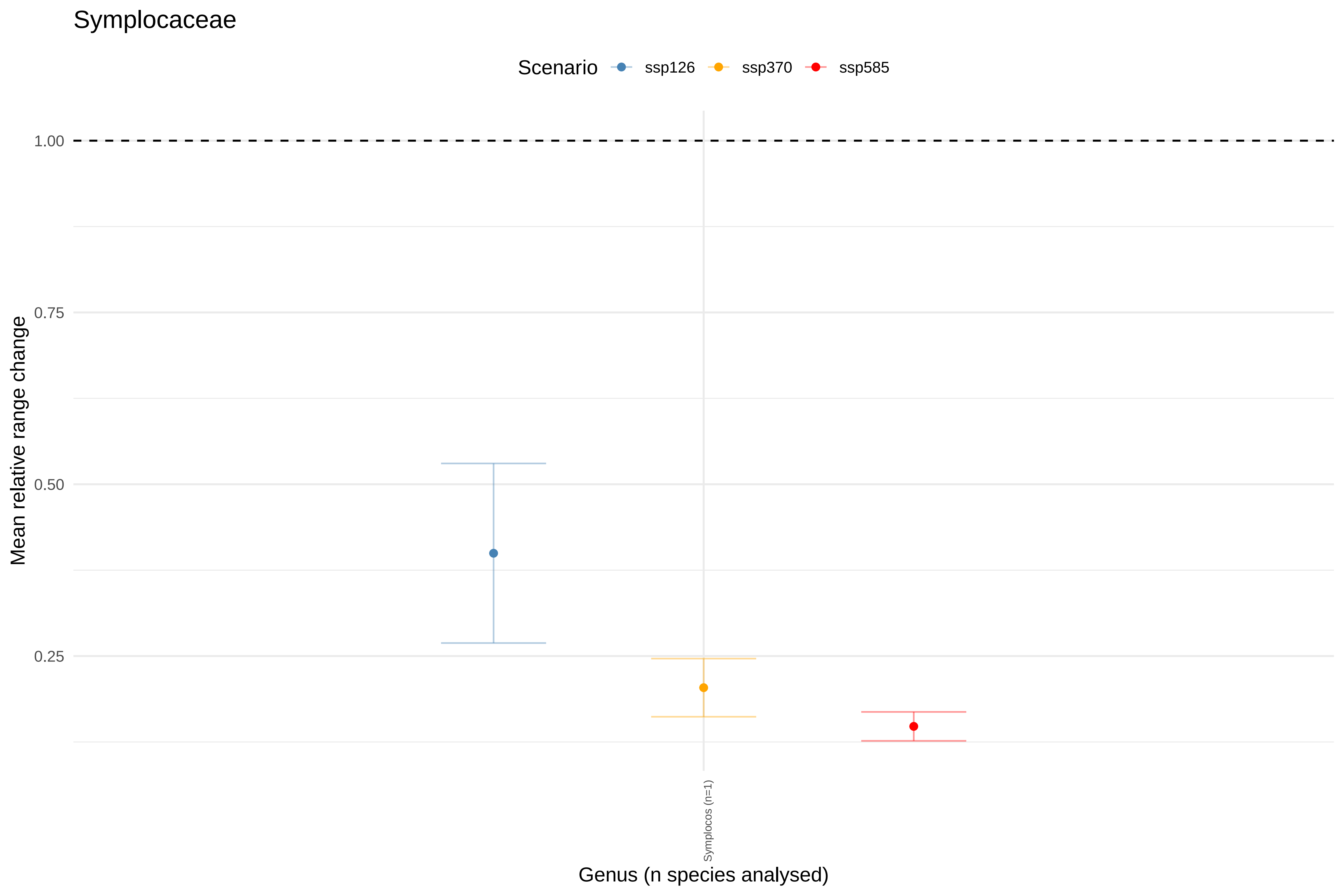

Symplocaceae
Scenario
ssp126
ssp370
ssp585
1.00
0.75
0.50
0.25
Mean relative range change
Symplocos (n=1)
Genus (n species analysed)

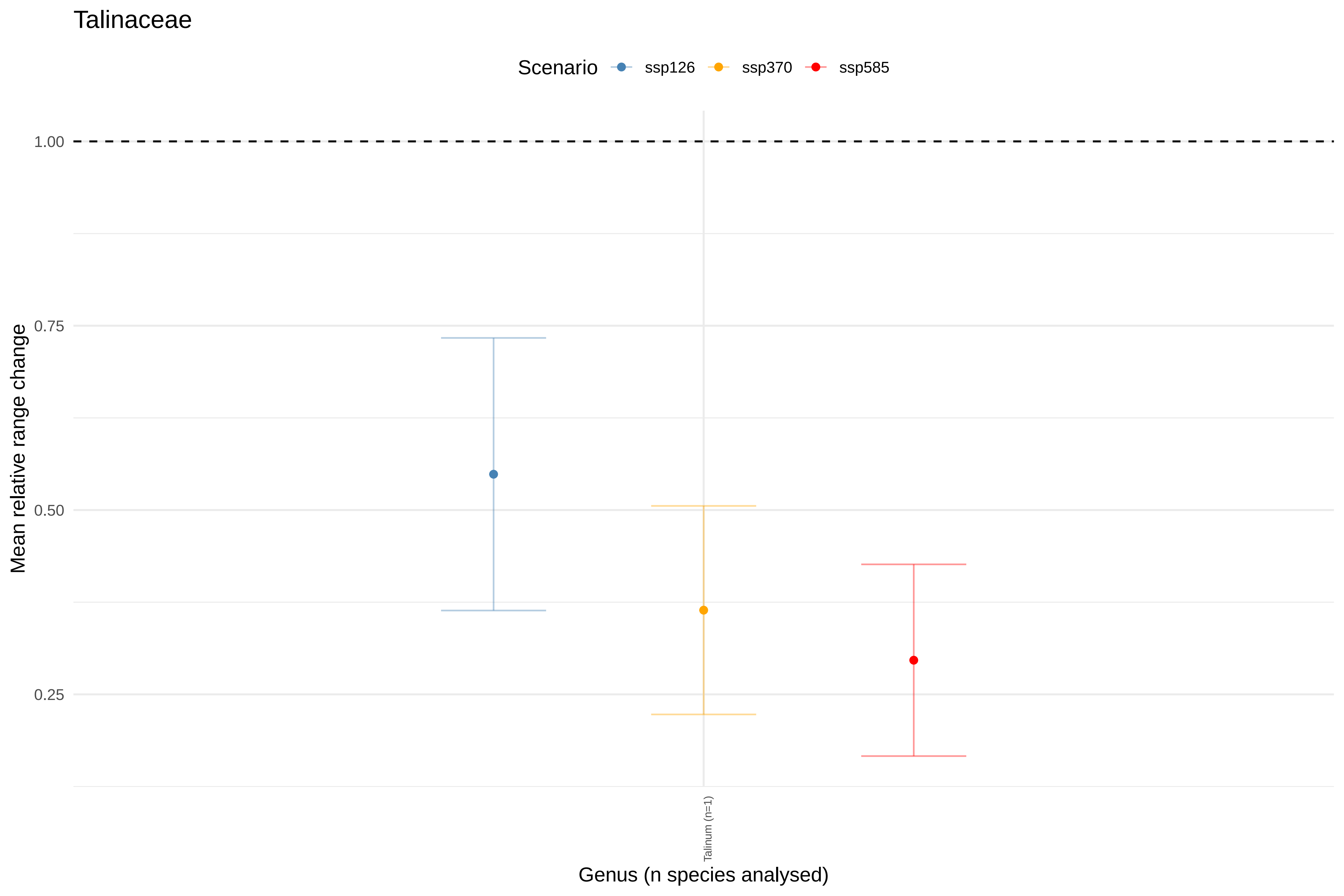
Talinaceae
Scenario
ssp126
ssp370
ssp585
1.00
0.75
0.50
0.25
Mean relative range change
Talinum (n=1)
Genus (n species analysed)

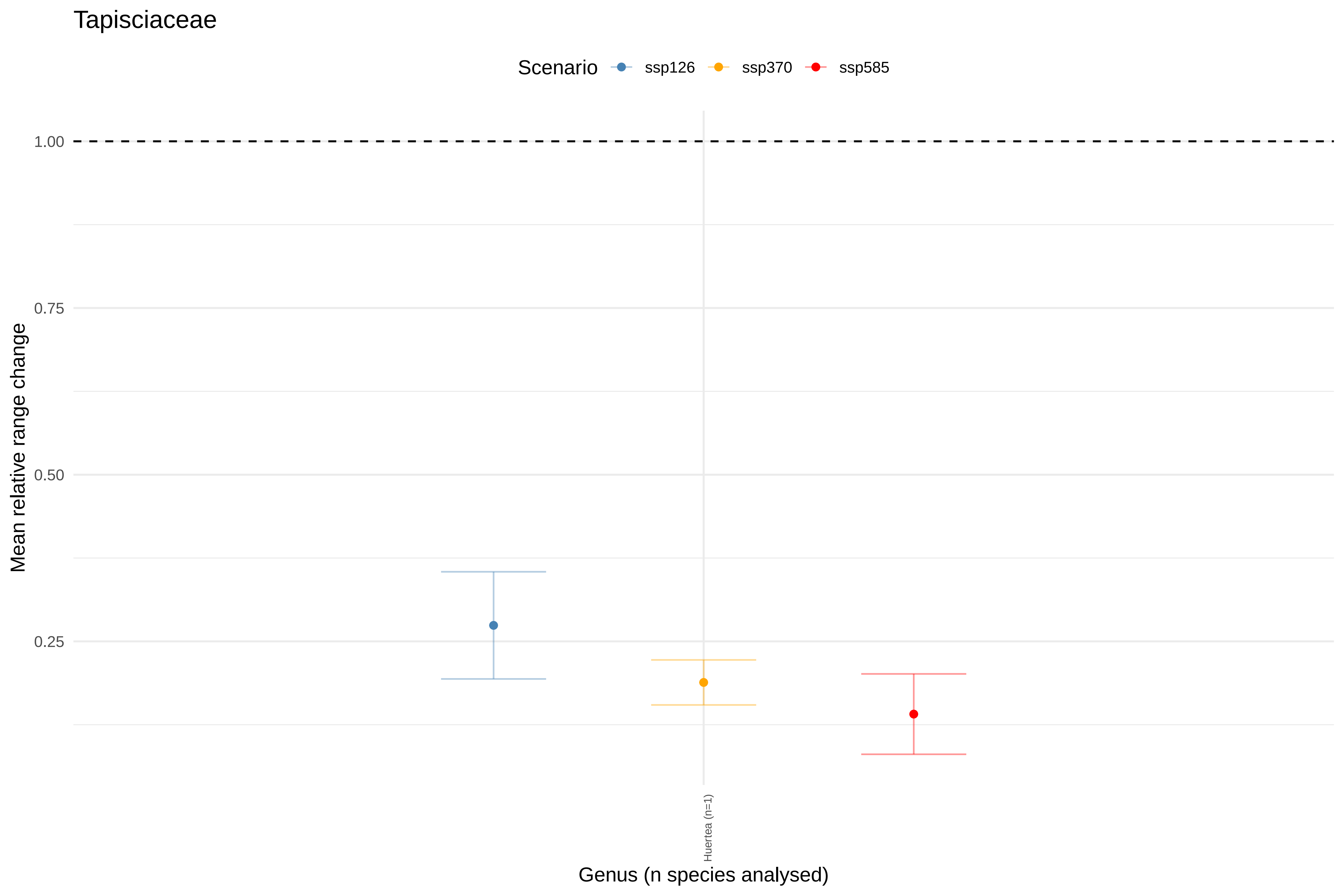

Tapisciaceae
Scenario
ssp126
ssp370
ssp585
1.00
0.75
0.50
0.25
Mean relative range change
Huertea (n=1)
Genus (n species analysed)

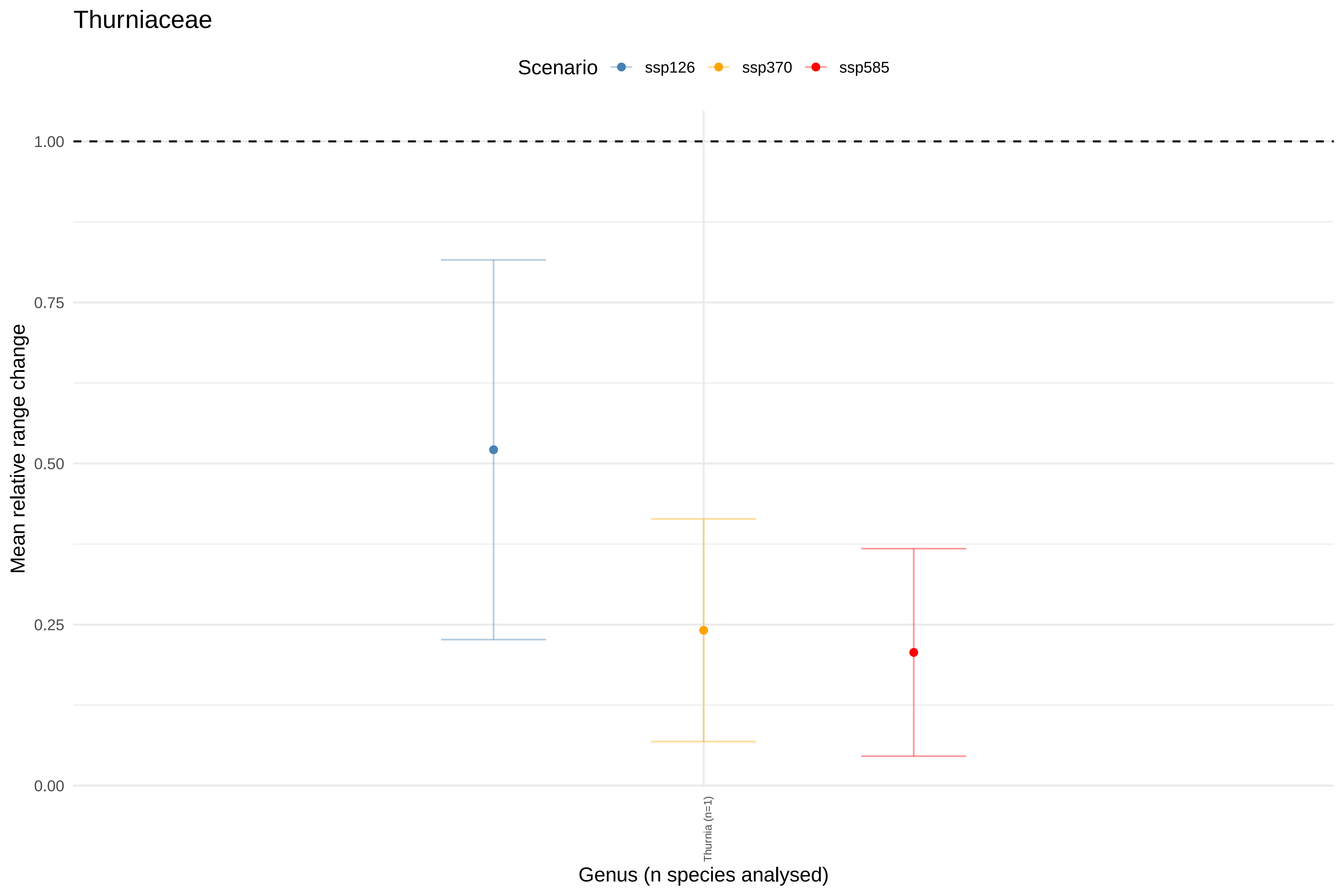
Thurniaceae
Scenario
ssp126
ssp370
ssp585
Mean relative range change
1.00
0.75
0.50
0.25
0.00
Thurnia (n=1)
Genus (n species analysed)

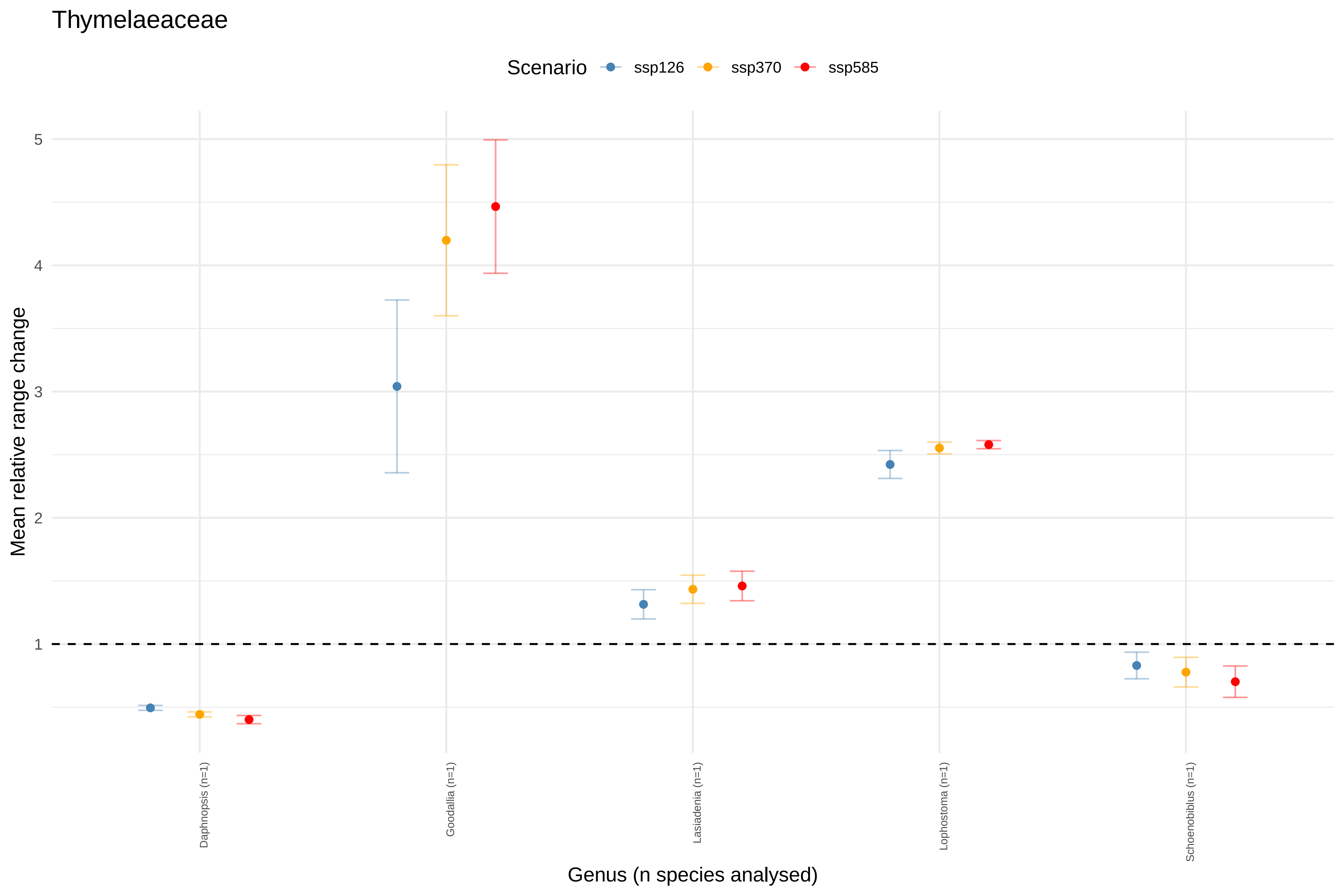

Thymelaeaceae
Scenario
ssp126
ssp370
ssp585
Mean relative range change
5
4
3
2
1
Daphnopsis (n=1)
Goodallia (n=1)
Lasiadenia (n=1)
Lophostoma (n=1)
Schoenobiblus (n=1)
Genus (n species analysed)

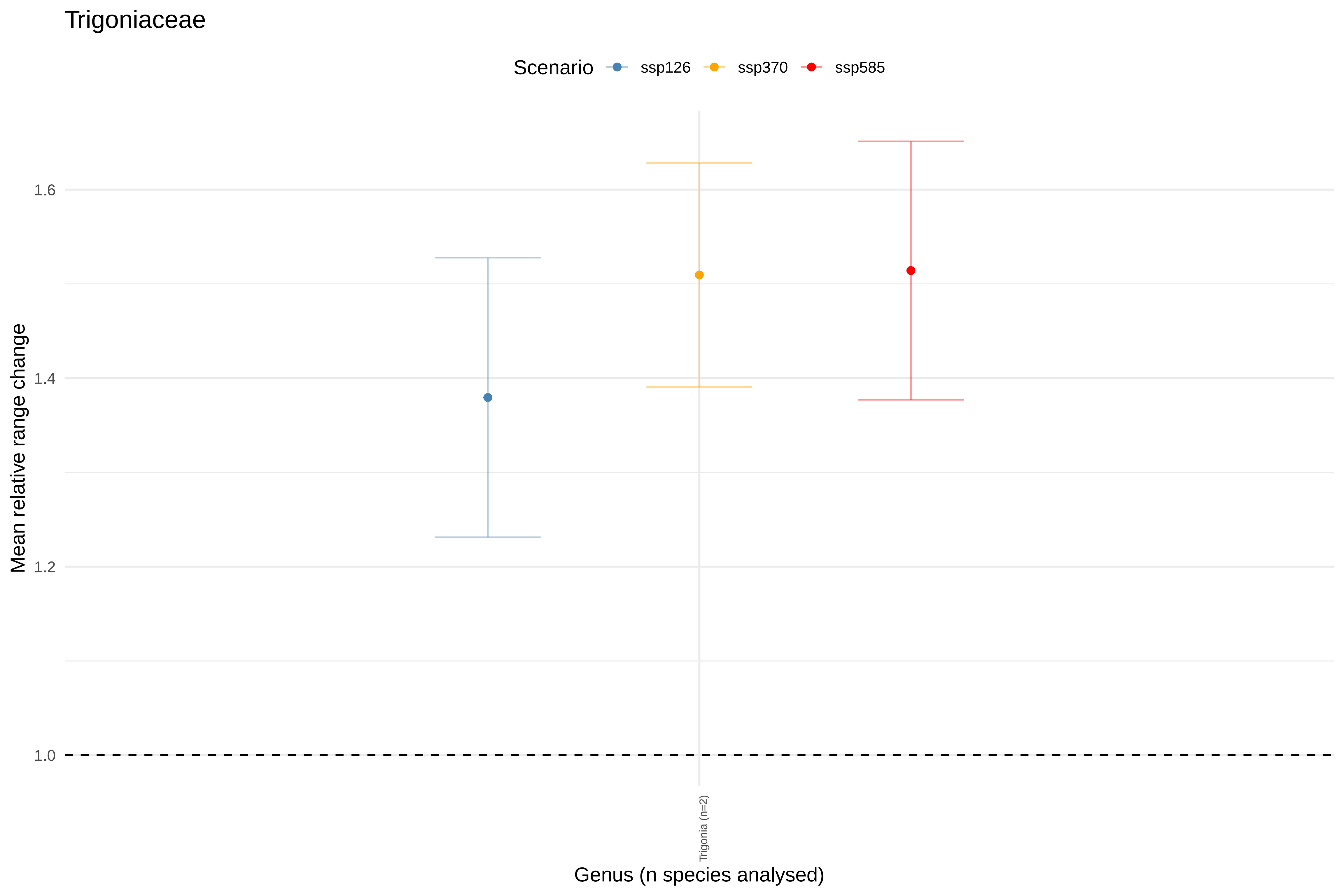

Trigoniaceae
Scenario
ssp126
ssp370
ssp585
Mean relative range change
1.6
1.4
1.2
1.0
Trigonia (n=2)
Genus (n species analysed)

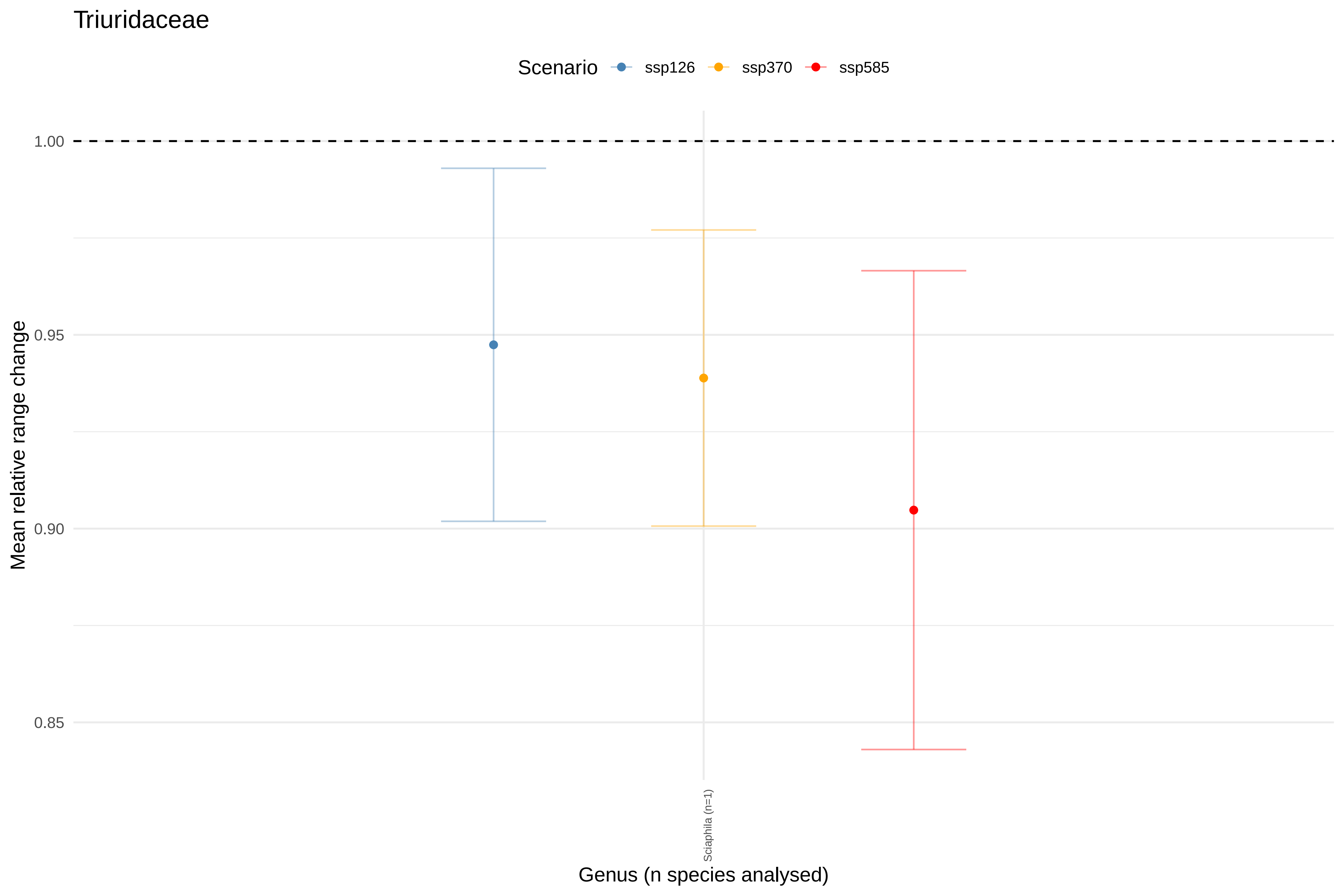

Triuridaceae
Scenario
ssp126
ssp370
ssp585
1.00
0.95
0.90
0.85
Mean relative range change
Sciaphila (n=1)
Genus (n species analysed)

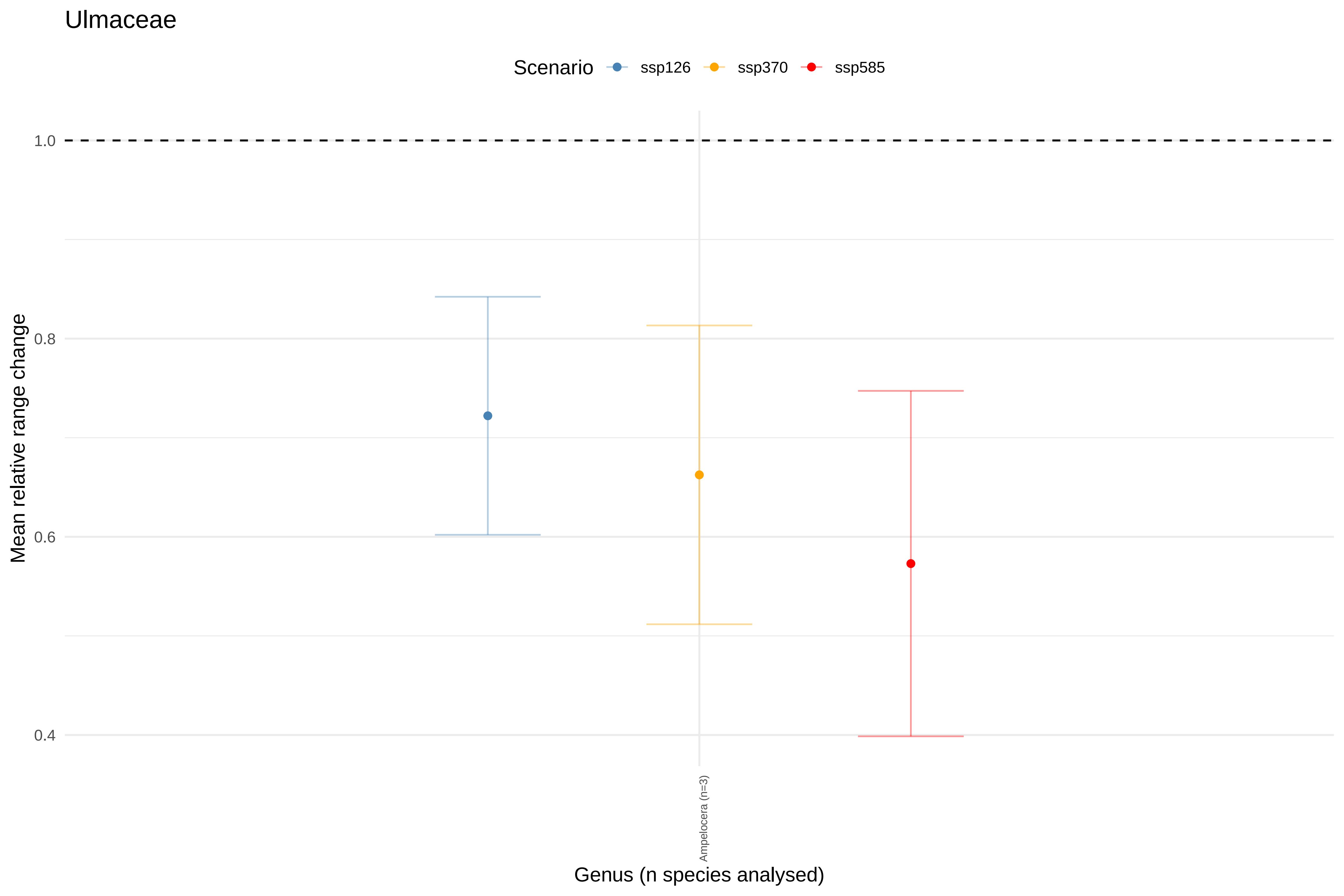

Ulmaceae
Scenario
ssp126
ssp370
ssp585
1.0
0.8
0.6
0.4
Mean relative range change
Ampelocera (n=3)
Genus (n species analysed)

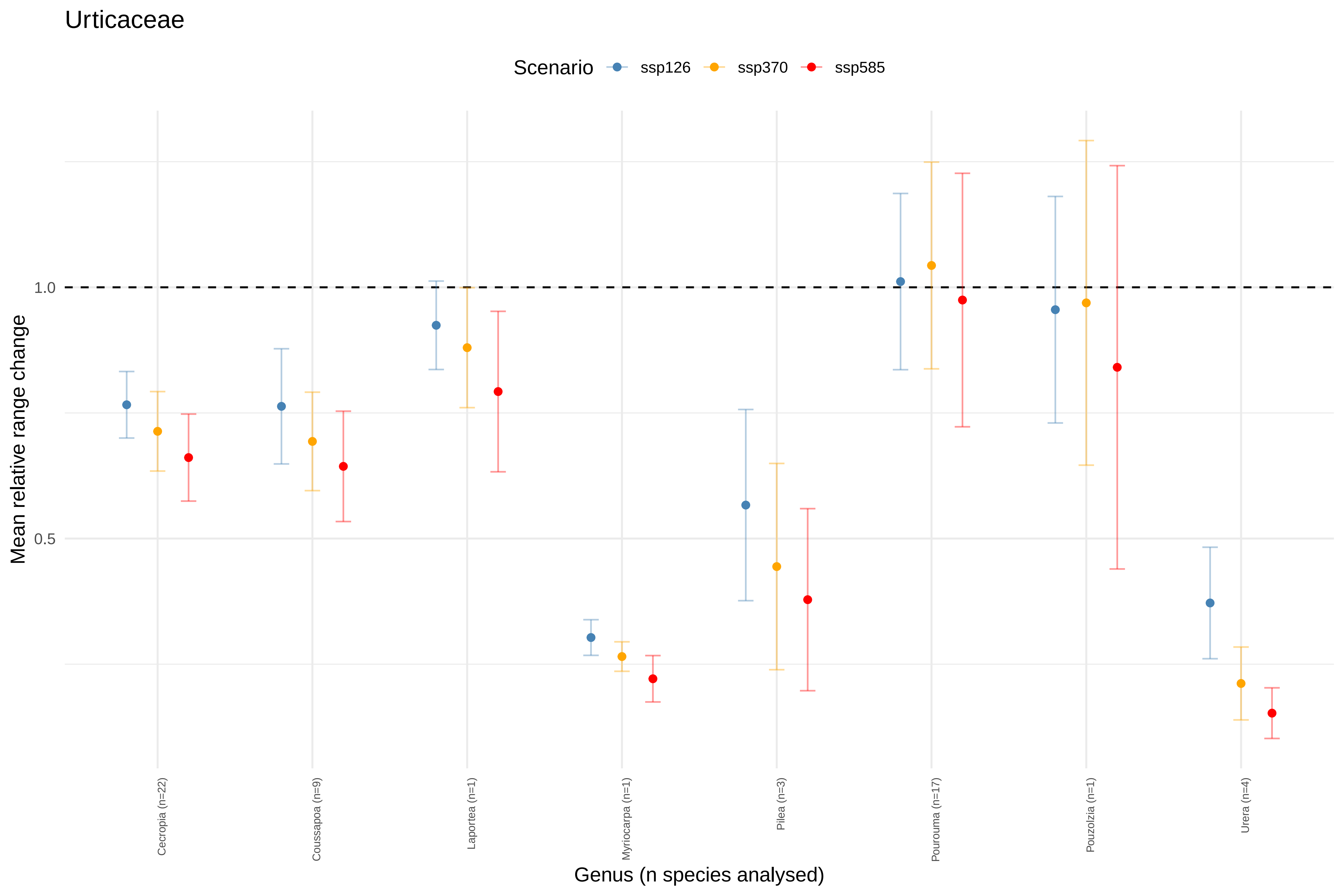

Urticaceae
Scenario
ssp126
ssp370
ssp585
Mean relative range change
1.0
0.5
Cecropia (n=22)
Coussapoa (n=9)
Laportea (n=1)
Myriocarpa (n=1)
Pilea (n=3)
Pourouma (n=17)
Pouzolzia (n=1)
Urera (n=4)
Genus (n species analysed)

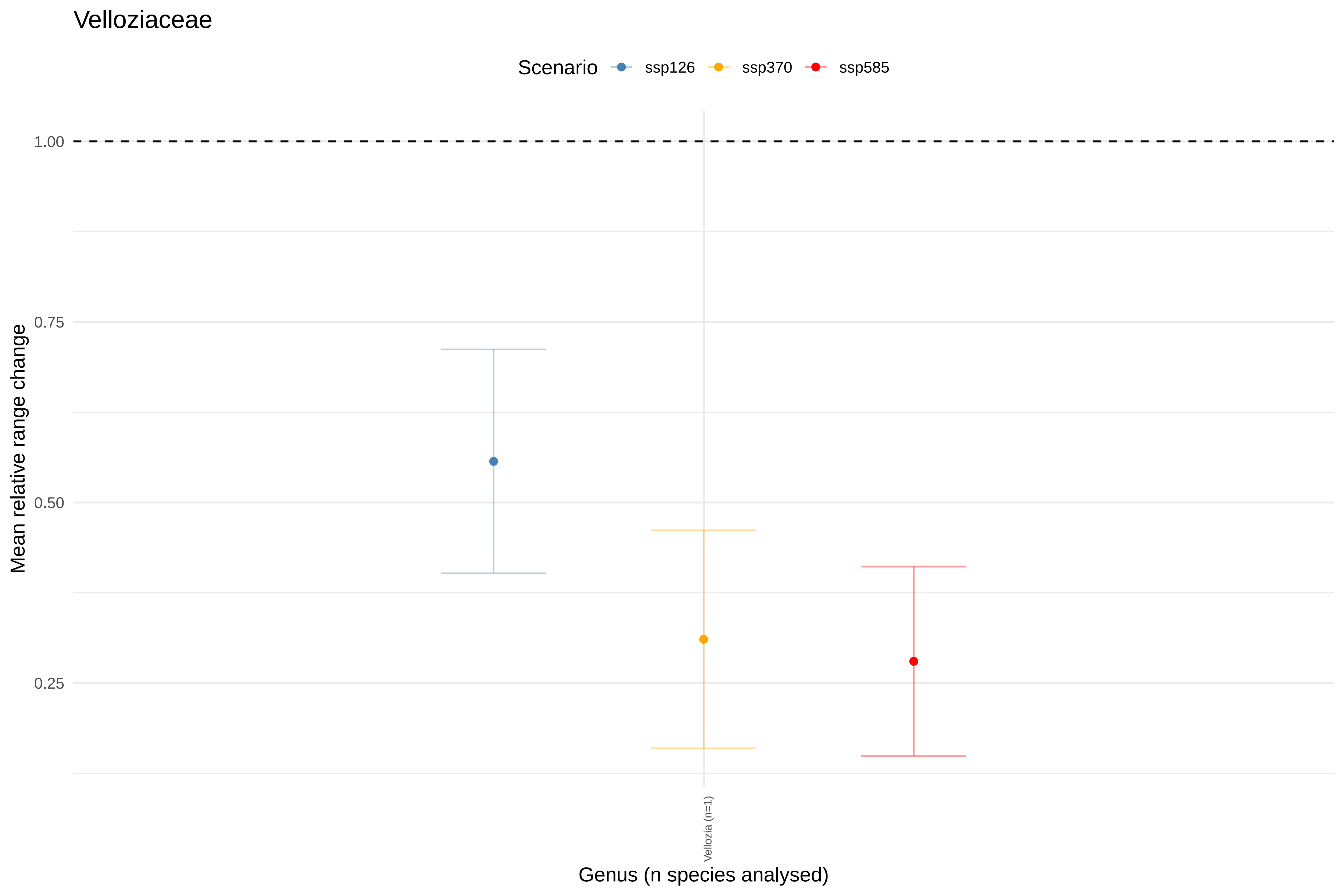
Velloziaceae
Scenario
ssp126
ssp370
ssp585
1.00
0.75
0.50
0.25
Mean relative range change
Vellozia (n=1)
Genus (n species analysed)

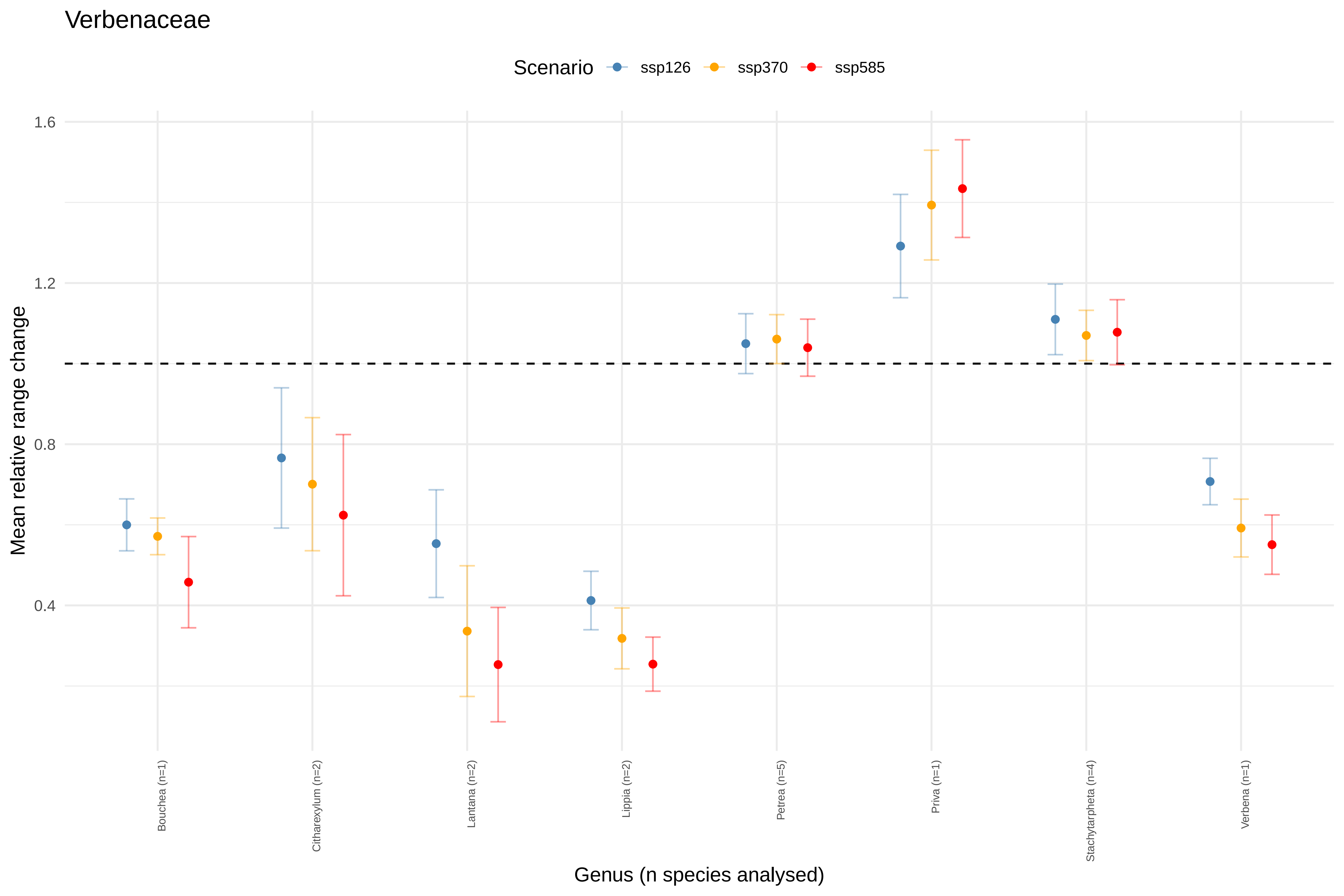
Verbenaceae
Scenario
ssp126
ssp370
ssp585
Mean relative range change
1.6
1.2
0.8
0.4
Bouchea (n=1)
Citharexylum (n=2)
Lantana (n=2)
Lippia (n=2)
Petrea (n=5)
Priva (n=1)
Stachytarpheta (n=4)
Verbena (n=1)
Genus (n species analysed)

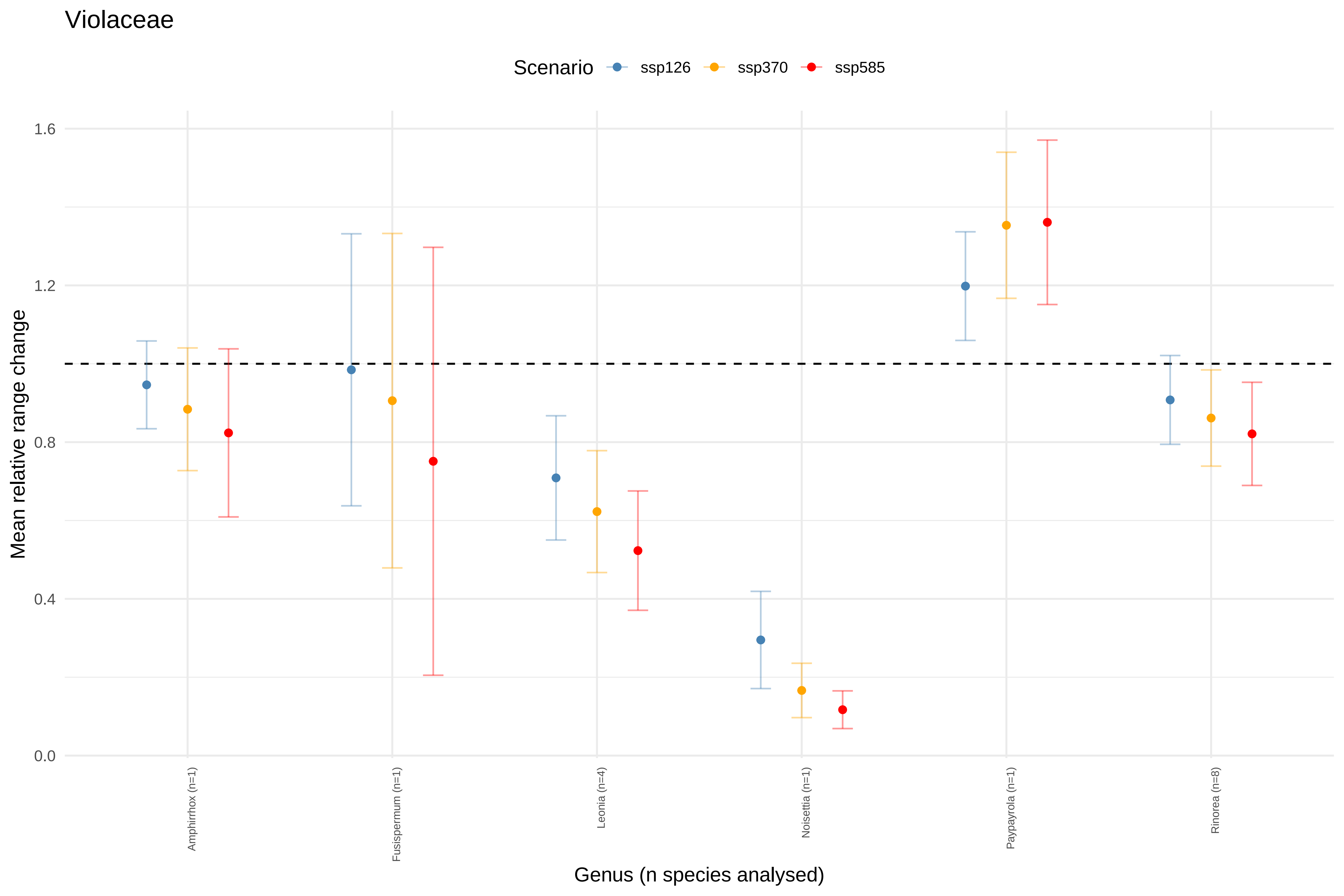
Violaceae
Scenario
ssp126
ssp370
ssp585
Mean relative range change
0.0
0.4
0.8
1.2
1.6
Amphirrhox (n=1)
Fusispermum (n=1)
Leonia (n=4)
Noisettia (n=1)
Paypayrola (n=1)
Rinorea (n=8)
Genus (n species analysed)

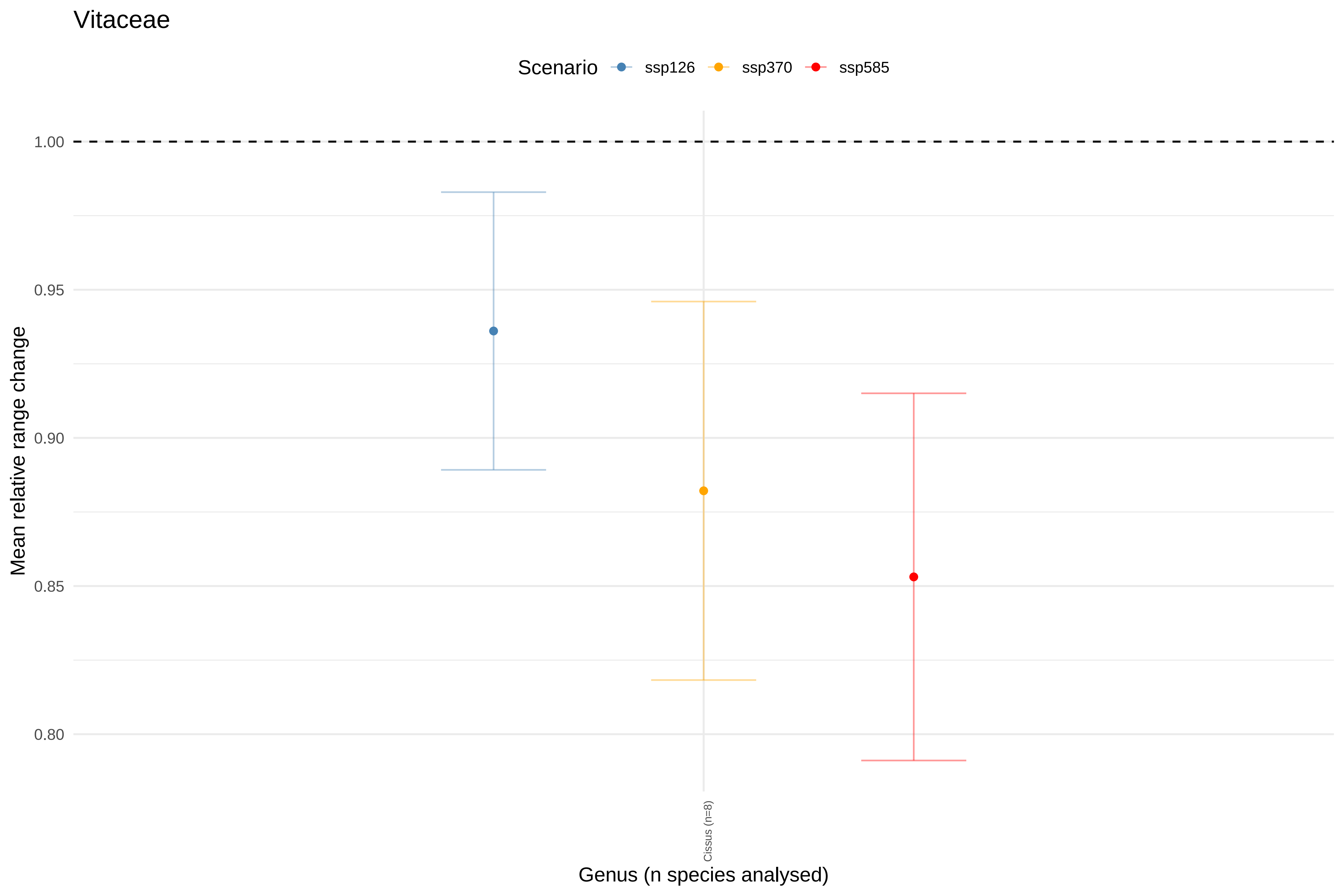
Vitaceae
Scenario
ssp126
ssp370
ssp585
1.00
0.95
0.90
0.85
0.80
Mean relative range change
Cissus (n=8)
Genus (n species analysed)

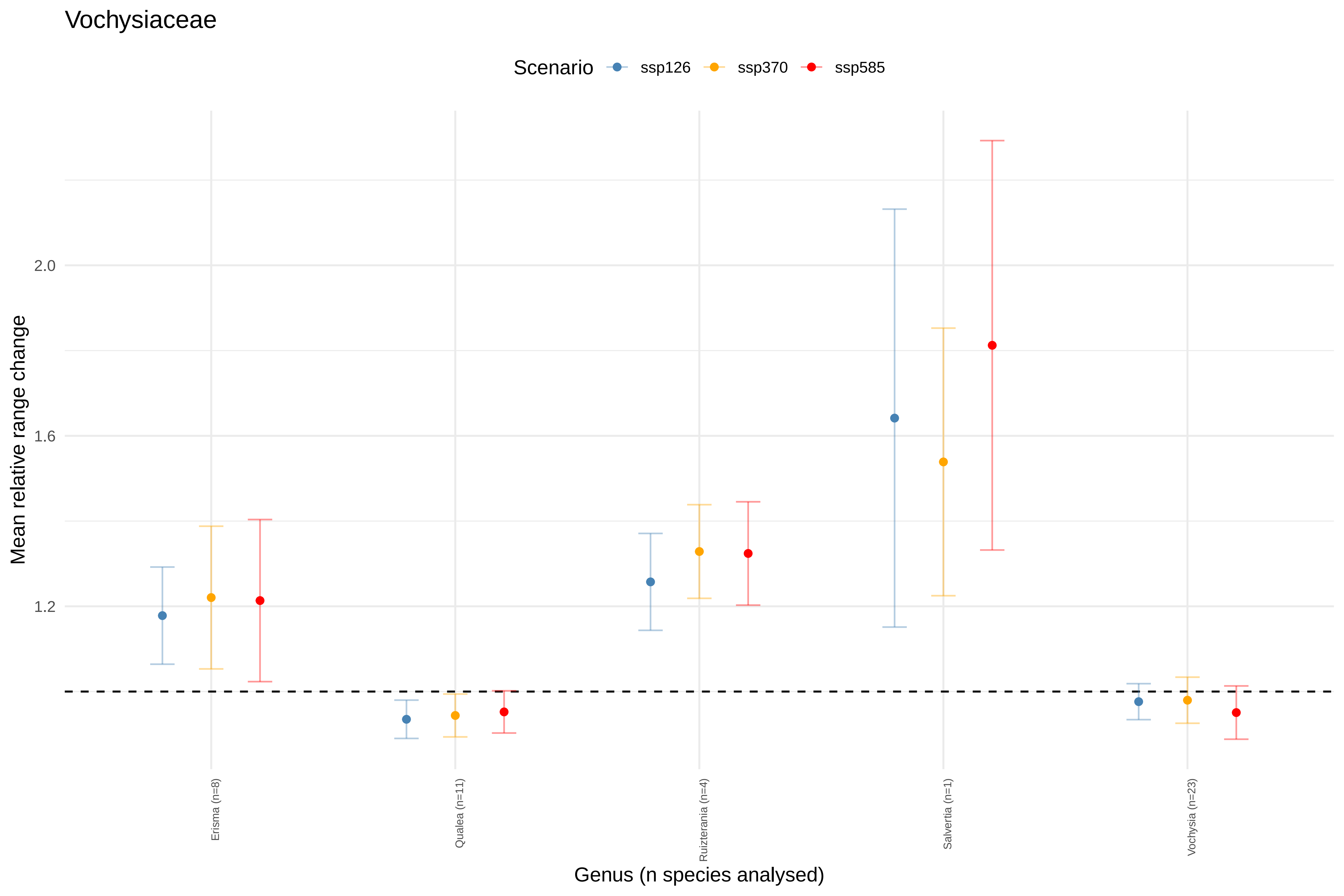

Vochysiaceae
Scenario
ssp126
ssp370
ssp585
Mean relative range change
2.0
1.6
1.2
Erisma (n=8)
Qualea (n=11)
Ruizterania (n=4)
Salvertia (n=1)
Vochysia (n=23)
Genus (n species analysed)

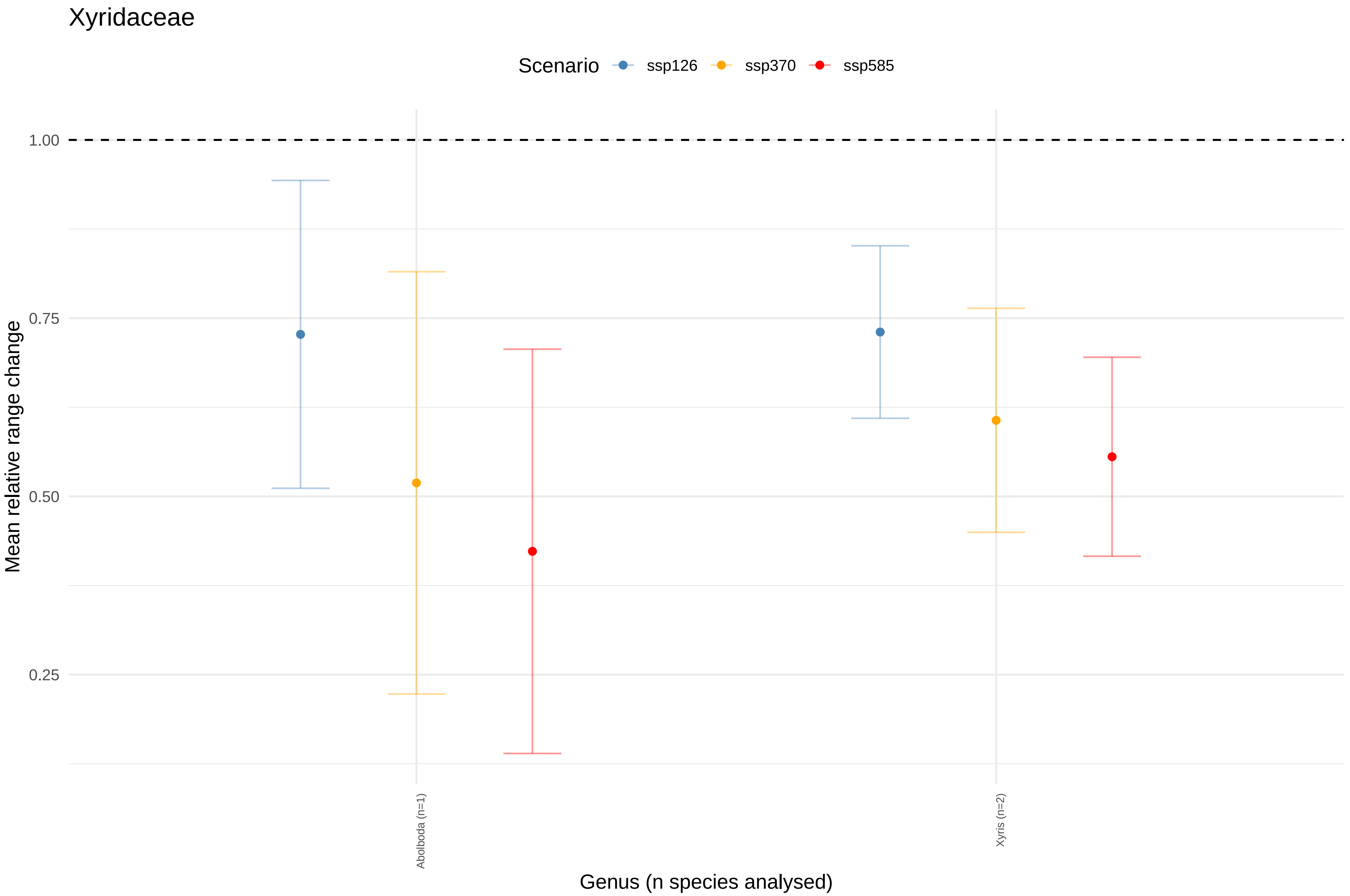

Xyridaceae
Scenario
ssp126
ssp370
ssp585
1.00
0.75
0.50
0.25
Mean relative range change
Abolboda (n=1)
Xyris (n=2)
Genus (n species analysed)

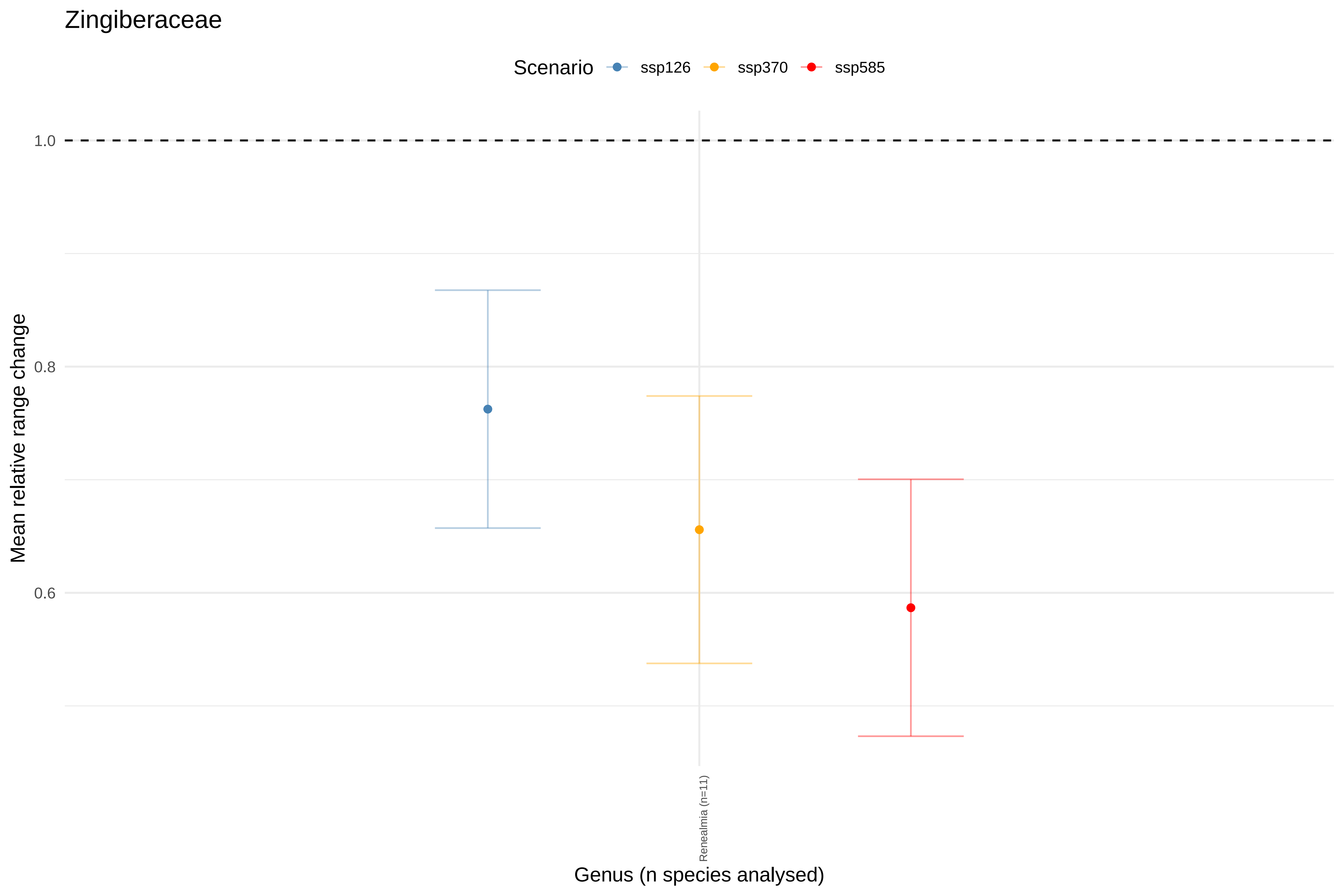
Zingiberaceae
Scenario
ssp126
ssp370
ssp585
1.0
0.8
0.6
Mean relative range change
Renealmia (n=11)
Genus (n species analysed)
