# Supplementary material for: The forest of knowledge under global change
Source: Nature. 2026 Jul 8;655(8125):1212–6. doi: 10.1038/s41586-026-10741-y (PMC13421348; doi:10.1038/s41586-026-10741-y)
Supplement: Supplementary file 4 — List of the 700 references included in the bibliographic review. [file 41586_2026_10741_MOESM4_ESM.pdf]

Supplementary Table 1 I List of the 700 references included in the bibliographic review.

ACEER, cited as source in: Duke, J.A., & Wain, K.K. (1981) Medicinal Plants of the World. Computer index with more than 85,000 entries, 3 vols.

Acero-Duarte, L.E. (1979) Principales Plantas Útiles de la Amazonia Colombiana. Editora Guadalupe, Bogotá.

Acevedo-Rodriguez, P. (1990) The occurrence of piscicides and stupefactants in the plant kingdom. *Advances in Economic Botany* 8: 1-23. https://www.jstor.org/stable/43927563

Acosta-Solis, M. (1952) Las fibras y lanas vegetales en el Ecuador. Contribución No. 21, Instituto Ecuatoriano de Ciencias Naturales. Casa de la Cultura Ecuatoriana, Quito.

Acosta, J. de (1590/1940) Historia natural y moral de las Indias, en que se tratan las cosas notables del cielo y elementos, metales, plantas y animales dellas; y los ritos, ceremonias, leyes y gobierno, y guerras de los indios (Edmundo O'Gorman, Ed.). México: Fondo de Cultura Económica; Imp. Manuel León Sánchez, S.C.L.

Acuña, C.D. (1641) Nuevo descubrimiento del gran río de las Amazonas. Imprenta Real, Madrid.

Aguilar, Z. (2006) Influence of the Huaorani on the conservation of Oenocarpus bataua, Arecaceae in Yasuní National Park and Biosphere Reserve, Amazonian Ecuador. *Lyonia* 10: 83-90. https://lyonia.org/downloadPDF-2.404.pdf

Aguirre, G. (2006) Plantas medicinales utilizadas por los indígenas Mosetén-Tsimane´ de la comunidad Asunción del Quiquibey, en la RB-TCO Pilón Lajas, Beni, Bolivia. Facultad de Ciencias Puras y Naturales. Universidad Mayor de San Andres.

Ahlbrinck, W. (1931) Encyclopaedie der Karaiben, Amsterdam, 1931; traduction française de Doude van Herwijnen, Institut Geographique National, Paris, 1956.

Alama Ybarra, C. (1950) Rio Negro. Caracas: Tipografía Vargas, pp. 53–57.

Alarcón, E.D.B. (2013) Estudio etnobotánico y etnofarmacológico de las especies aromáticas usadas en ceremonias de ayahuasca por la etnia Huarayo (Puerto Maldonado). Tesis. Universidad Nacional de San Antonio Abad del Cusco, Peru.

Alarcón, R. (1988) Etnobotánica de los Quichuas de la Amazonia ecuatoriana. Miscelánea Antropológica Ecuatoriana, Serie Monográfica (Ecuador) 7. Museos del Banco Central del Ecuador. ISSN 0254-7678.

Alarcón, R. (1994) El taller "Etnobotánica y valoración económica de los recursos florísticos silvestres". Etnobotánica, Valoración Económica y Comercialización de Recursos Florísticos Silvestres en el Alto Napo, Ecuador, Ecociencia, Quito.

Albán, J. (1994) La mujer y las plantas útiles silvestres en la comunidad Cocama-Cocamilla de los ríos Samiria y Marañon. Informe de Proyecto. Wild World Life Foundation-Biodiversity Support Program 7560, Lima.

Albarracin, J. & Caycedo, A.O. (2001) La pesca entre los Ticuna: história, técnicas y ecossistemas. *Boletín de Antropología* 13(30): 73-99. https://revistas.udea.edu.co/index.php/boletin/article/view/337509

Albis, M.M. (1936). Memorias de un viajero. Publicadas por José María Vergara i Vergara y Evaristo Delgado (1854). *Revista Popayán* 26(163–165), 28–32.

Alexiades, M.N. (1999) Ethnobotany of the Ese Eja: Plants, health, and change in an Amazonian society (Doctoral dissertation, City University of New York).

Allen, P.H. (1947) Indians of Southeastern Colombia. *Geographical Review* 37: 567-582. https://www.jstor.org/stable/211186

Álvarez Maldonado, J. (1899) Relación de la jornada y descubrimiento del río Manu (hoy Madre de Dios), por Juan Álvarez Maldonado en 1567 (Publícala Luis Ulloa). Sevilla: Imprenta y Litografía de C. Salas. xxiii + 53 pp., 1 mapa.

Alves de Souza, J.M. & Chau Ming, L. (2016) Plantas medicinais utilizadas por seringueiros na Reserva Extrativista São Luiz do Remanso, Acre. Chapter 13. In: Siviero, A. Chau Ming, L., Silveira, M., Daly, D. & Wallace, R. (eds.) Etnobotânica e Botânica Econômica do Acre. Editora da Universidade Federal do Acre-Edufac. ISBN: 978-85-8236-027-9.

Anderson, A.B. (1977) Os nomes e usos de palmeiras entre uma tribo de índios Yanomama. *Acta Amazonica* 7(1): 5-13. https://doi.org/10.1590/1809-43921977071005

Anderson, P.J. (2004) The social context for harvesting *Iriartea deltoidea* (Arecaceae). *Economic Botany* 58: 410-419. https://www.jstor.org/stable/4256835

Andoque, H. et al. (2009) Plantas medicinales de la Gente de Hacha. Universidad Nacional de Colombia-Sede Amazonia. https://repositorio.unal.edu.co/handle/unal/70174

Andrade, A.A. (1926) Estudo das matérias corantes de origem vegetal, em uso entre os índios do Brasil e das plantas de que procedem. *Archivos do Museu Nacional do Rio de Janeiro* 28: 177–199.

Andrade, J.N., Costa Neto, E.M. & Brandao, H. (2015) Using ichthyotoxic plants as bioinsecticide: A literature review. *Revista Brasileira de Plantas Medicinais* 17(4): 649-656. https://doi.org/10.1590/1983-084X/13_105

Angel, L.H. (1976) El nacimiento de los Matapí. *Revista Colombiana de Antropología* XX: 201–280. https://doi.org/10.22380/2539472X.1734

Anonymous (1977) Las plantas. Mundo Shuar 5. Serie A, Fasciculo 5. Ediciones Mundo Shuar, Centro de Documentación, Investigación y Publicaciones, Sucua.

Antezana, L. (1976) Palmeras nativas de Bolivia de valor económico. Pp 87-97. In: Villegas, C. (ed.) Simposio internacional sobre plantas de interés económico de la flora Amazónica, Turrialba, Costa Rica. https://hdl.handle.net/11324/13251

Antolinez, L.D. (1999) La alimentación en la Amazonía: Estudio de caso entre los coreguajes. Graduate thesis. Departamento de Filología e Idiomas, Facultad de Ciencias Humanas. Universidad Nacional de Colombia, Bogotá.

Araujo-Murakami, A. (2019) Barbascos y Curare en Bolivia. *Kempffiana* 15 (1): 57-63. https://museonoelkempff.org/sitio/Informacion/KEMPFFIANA/kempffiana15(1)/5_Araujo-Murakami.pdf

Arcila Robledo, G. (1950) Las misiones franciscanas en Colombia: Estudio documental. Bogotá: Imprenta Nacional.

Armesilla, P.J. (2006) Usos de las palmeras (Arecaceae) en la Reserva de la Biosfera-Tierra Comunitaria de Orígen Pilón Lajas, (Bolivia). Facultad de Ciencias. Universidad Autónoma de Madrid, Madrid.

Arnaud, E. (1975) Os índios Gaviões de Oeste Pacificação e integração. Belem, Museu Paraense Emilio Goeldi. 86 Pg. (Publ. Avulsas, 28). https://repositorio.museu-goeldi.br/handle/mgoeldi/902

Arrais, F.D.C.L. et al. (2017) Levantamento etnobotânico nas margens do córrego Machado-Palmas, Tocantins, Brazil. *FLOVET-Boletim do Grupo de Pesquisa da Flora, Vegetação e Etnobotânica* 1(9): 58-68. https://periodicoscientificos.ufmt.br/ojs/index.php/flovet/article/download/5486/3610

Athias, R. (2015) Hãwäg and b'atìb: The Balance between Health and Disease among the Hupd'äh in the Upper Rio Negro Region, Brazil. *Tipiti: Journal of the Society for the Anthropology of Lowland South America* 13(2): 61-73. https://digitalcommons.trinity.edu/cgi/viewcontent.cgi?article=1207&context=tipiti

Aublet, F. (1775) Histoire des Plantes de la Guaiane Françoise. Paris, Pierre-Francois Didot jeune, Libraire de la Faculté de Médicine. Vol. 2. https://doi.org/10.5962/bhl.title.674

Ayala Flores, F. (1984) Notes on some medicinal and poisonous plants of Amazonian Peru. *Advances in Economic Botany* 1: 1-8. https://www.jstor.org/stable/43931364

Báez, S. (1998) Dictionary of plants used by the Canelos-Quichua. In: People and biodiversity: Two case studies from the Andean foothills of Ecuador. Centre for research on cultural and biological diversity of Andean rainforests. DIVA Technical Report. Link

Báez, S. & Backevall, Å. (1998) Dictionary of plants used by the Shuar of Makuma and Mutints. . In: People and biodiversity: Two case studies from the Andean foothills of Ecuador. Centre for research on cultural and biological diversity of Andean rainforests. DIVA Technical Report. Link

Baillon, H. (1879) Nouvelles observations sur les plantes du curare. *Adansonia* 12: 366. https://www.biodiversitylibrary.org/item/25865#page/378

Baillon, H. E. (1880) *Strychnos melinoniana* (Loganiaceae), nouvelle espèce. *Bulletin mensuel de la Société Linnéenne de Paris* 1: 256.

Balée, W. (1987) A etnobotânica quantitativa dos índios Tembé (Rio Gurupi, Pará). *Boletim do Museu Paraense Emilio Goeldi. Nova serie Botánica* 3(1): 29-50. https://acervo.socioambiental.org/acervo/documentos/etnobotanica-quantitativa-dos-indios-tembe-rio-gurupi-pa

Balée, W. (1989) The culture of Amazonian forests. *Advances in Economic Botany* 7: 1-21. https://www.jstor.org/stable/43927542

Balée, W. (1994) Footprints of the forest: Ka'apor ethnobotany-the historical ecology of plant utilization by an Amazonian people. Columbia University Press. ISBN: 9780231074858. https://cup.columbia.edu/book/footprints-of-the-forest/9780231074858/

Balée, W. & Gély, A. (1989) Managed forest succession in Amazonia: The Ka'apor case. *Advances in Economic Botany* 7: 129-158. https://www.jstor.org/stable/43927549

Balick, M.J. (1985) Useful plants of Amazonia: A resource of global importance. Pg. 339-368 (Chap. 19). In: Prance, G.T. & Lovejoy, T.E. (eds.) Amazonia. Pergamon Press. https://www.nybg.org/files/scientists/mbalick/UsefulPlantsofAmazonia.pdf

Balick, M.J. (1986) Systematics and economic botany of the *Oenocarpus-Jessenia* (Palmae) complex. *Advances in Economic Botany* 3: 1-140. https://www.jstor.org/stable/43927509

Balslev, H. & Barfod, A. (1987) Ecuadorean palms- an overview. *Opera Botanica* 92: 17-35. https://www.researchgate.net/publication/292219379_Ecuadorean_palms_-_an_overview

Balslev, H. & Henderson, A. (1987) A New *Ammandra* (Palmae) from Ecuador. *Systematic Botany* 12: 501-504. https://doi.org/10.2307/2418885

Balslev, H. et al. (1997) Palmas útiles en la cordillera de los Huacamayos. PROBONA, Quito. https://www.researchgate.net/profile/Montserrat-Rios/publication/283122653_Palmas_utiles_en_la_Cordillera_de_los_Huacamayos_provincia_del_Napo_Ecuador/links/562bc9ba08ae04c2aeb35761/Palmas-utiles-en-la-Cordillera-de-los-Huacamayos-provincia-del-Napo-Ecuador.pdf

Balslev, H. et al. (2008) Useful palms (Arecaceae) near Iquitos, Peruvian Amazon. *Revista Peruana de Biología* 15: 121-132. http://www.scielo.org.pe/scielo.php?script=sci_arttext&pid=S1727-99332008000000014

Barandiarán, D. de (1968) El fuego entre los indios Sanema-Yanoama. *Antropológica* 22, 1–64.

Barbosa Rodrigues, J. (1891) Loganiaceae. In Vellosia: Contribuições do Museu Botânico do Amazonas (2nd ed., Vol. 1, pp. 33–44). Imprensa Nacional.

Barbosa Rodrigues, J. (1893) Vocabulario indígena com a orthographia correcta (Complemento da Poranduba Amazonense). Rio de Janeiro: Typ. de C. Leuzinger & Filhos, Publicação da Bibliotheca Nacional.

Barbosa Rodrigues, J. (1903) L'Uiraery ou Curare. Monnom, Bruxelles.

Barker, J. (1953) Memoria sobre la cultura de los Guaika (W. Dupouy, Trans.). *Bol. Indig. Ven.* 1(1): 433-489.

Barneby, R.C., & Krukoff, B.A. (1971) Supplementary notes on American Menispermaceae. VIII. A generic survey of the American Triclisieae and Anomospermeae. *Memoirs of the New York Botanical Garden* 22(2): 1–89.

Barrère, P. (1743) Nouvelle relation de la France équinoxiale, contenant la description des côtes de la Guyane; de l'isle de Cayenne; le commerce de cette colonie; les divers changemens arrivés dans ce pays; & les mœurs & coutumes des différens peuples sauvages qui l'habitent. Avec des figures dessinées sur les lieux. Paris: Moreau.

Barriga, H.G. (1974) Flora Medicinal de Colombia. Instituto de Ciencias Naturales. Universidad Nacional. Vols. 1-3.

Barriga, R. (1994) Plantas útiles de la Amazonia Peruana: características, usos y posibilidades. CONCYTEC, Lima. 261 pg.

Barrire, P. (1743) Nouuelle Relation de la France Equinoxiale, Paris. https://www.biodiversitylibrary.org/item/163112#page/9/mode/1up

Barros, A.P. et al. (2017) Uso medicinal de plantas na comunidade de Santa Helena, Axixá-Tocantins. *Revista Craibeiras de Agroecologia* 1(1): 1-4. https://www.seer.ufal.br/index.php/era/article/download/3833/2919/14539

Bates, H.W. (1863) The Naturalist on the River Amazons. Volume 1. John Murray, London. https://www.biodiversitylibrary.org/page/18556984

Bates, H.W. (1863) The Naturalist on the River Amazons. Volume 2. John Murray, London. https://doi.org/10.5962/bhl.title.103298

Becerra, G.C. (2023) Las plantas y los pueblos indígenas de tradición nómada del noroeste amazónico en Alto Río Negro–Vaupés, frontera de Colombia y Brasil. *Etnobiología* 21(3): 14–36. https://revistaetnobiologia.mx/index.php/etno/article/view/537

Beck, H.T. (1990) A survey of the useful species of *Paullinia* L. (Sapindaceae). *Advances in Economic Botany* 8: 41-56. https://www.jstor.org/stable/43927566

Beltrán Zapata, G.D. (2016) Conocimiento tradicional y los modos de transmisión de saberes alrededor de las plantas medicinales en la comunidad de Macaquiño (zona Aatiam, territorio del Vaupés) (Master dissertation). https://repositorio.unal.edu.co/handle/unal/58071

Bennett, B.C., Baker, M.A. & Gómez-Andrade, P. (2002) Ethnobotany of the Shuar of Eastern Ecuador. *Advances in Economic Botany* 14: 1-299. https://www.jstor.org/stable/43927635

Bentham, G. (1854) On the north Brazilian Euphorbiaceae in the collections of Mr. Spruce. *Hooker. Journ. Bot.* 6: 363-371. https://www.biodiversitylibrary.org/item/6324#page/1/mode/1up

Bergman, R. (1980) Amazon Economics: The Simplicity of Shipibo Indian Wealth. Department of Geography. Syracuse University.

Berkel, A. van (1670–1689/1941) Travels in South America between the Berbice and Essequibo rivers and in Surinam (1670–1689) (W.E. Roth, Trans. & Ed.). Georgetown: Daily Chronicle Ltd. (The Guiana Edition, No. 2, 2nd impression).

Berlin, B. & Berlin, E.A. (1979) Etnobiología subsistencia y nutrición en una sociedad de la selva tropical: los Aguaruna. Pg. 13-47. In: A. Chirif (comp.) Salud y nutrición en comunidades nativas. Lima: CIPA.

Bernal, R. (1992) Colombian palm products. Pg. 158-173. In: Plotkin, M., Famolare, L. Sustainable Harvest and Marketing of Rain Forest Products. Island Press, Washington.

Bernal, R., Galeano, G., García, N., Olivares, I.L., & Cocomá, C. (2010) Uses and commercial prospects for the Wine Palm, *Attalea butyracea*, in Colombia. *Ethnobotany Research and Applications* 8: 255-268.

Bertani, S. et al. (2005) Evaluation of French Guiana traditional antimalarial remedies. *Journal of Ethnopharmacology* 98(1-2): 45-54. https://doi.org/10.1016/j.jep.2004.12.020

Bianchi, C. (1982) Artesanías y técnicas Shuar. Ediciones Mundo Shuar, Quito.

Bieski, I.G.C. et al. (2015) Ethnobotanical study of medicinal plants by population of Valley of Juruena Region, legal Amazon, Mato Grosso, Brazil. *Journal of Ethnopharmacology* 173: 383-423. https://doi.org/10.1016/j.jep.2015.07.025

Bisset, N.G. (1992) Curare. In S.W. Pelletier (Ed.), Alkaloids: Chemical and biological perspectives. Springer-Verlag, New York.

Bisset, N.G. (1992) Uses, chemistry and pharmacology of *Malouetia* (Apocynaceae, subf. Apocynoideae). *Journal of Ethnopharmacology* 36(1): 43-50. https://doi.org/10.1016/0378-8741(92)90059-Z

Bisset, N.G. (1992) War and hunting poisons of the New World. Part 1. Notes on the early history of curare. *Journal of Ethnopharmacology* 36(1): 1-26.

Bloca, E. (1965) Viaggi tra gli Indi: Alto Río Negro, Alto Orinoco, vol. 3. Roma: C.N.R.

Blohm, H. (1962) Poisonous plants of Venezuela. Harvard University Press, Cambridge, Massachusetts. ISBN: 0674681509. https://books.google.ch/books/about/Poisonous_Plants_of_Venezuela.html?id=ZdHNzQEACAAJ&redir_esc=y

Bodley, J.H. & Benson, F.C. (1979) Cultural ecology of Amazonian palms. Reports of investigations, No. 56. Laboratory of Anthropology, Washington State University, Pullman, Washington. https://books.google.ch/books/about/Cultural_Ecology_of_Amazonian_Palms.html?id=PgBCAAAAIAAJ&redir_esc=y

Boll, T. et al. (2005) Spatial distribution and environmental preferences of the piassaba palm *Aphandra natalia* (Arecaceae) along the Pastaza and Urituyacu rivers in Peru. *Forest Ecology and Management* 213: 175-183. https://doi.org/10.1016/j.foreco.2005.03.020

Boom, B.M. (1986) The Chacobo indians and their palms. *Principes* 30(2): 63-70. https://palms.org/wp-content/uploads/2016/05/v30n2p63-70.pdf

Boom, B.M. (1987) The Panare Indians and their forest: Survival of a Venezuelan culture. *Journal of the Washington Academy of Sciences* 77: 178–182.

Boom, B.M. (1990) Useful plants of the Panare Indians of the Venezuelan Guayana. *Advances in Economic Botany* 8: 57-76. https://www.jstor.org/stable/43927567

Boom, B.M. (1996) Ethnobotany of the Chácobo Indians, Beni, Bolivia. *Advances in Economic Botany* 4: 1-74. https://www.jstor.org/stable/43931377

Borchsenius, F., Borgtoft, H. & Balslev, H. (1998) Manual to the Palms of Ecuador. AAU Reports 37: 1-217. ISBN: 8787600536.

Borgtoft, H. (1992) Uses and management of *Aphandra natalia* (Palmae) in Ecuador. *Bulletin de l´Institute Française d´Études Andines* 2: 741-753. https://www.persee.fr/doc/bifea_0303-7495_1992_num_21_2_1085

Borgtoft, H. (1996) Production and harvest of fibers from *Aphandra natalia* (Palmae) in Ecuador. *Forest Ecology and Management* 80: 155-161. https://doi.org/10.1016/0378-1127(95)03632-6

Boso, J.M. (1815, mayo 25) Carta sobre las montañas de Yucarés. In: H. Valdizán & Á. Maldonado (Eds.)(1922) La medicina popular peruana. Contribución al folklore médico del Perú (Tomo II, pp. 348–388). Imprenta Torres Aguirre, Lima.

Bourdy, G. (1999) Conozcan nuestros árboles, nuestras hierbas. UMSA, CIPTA, IRD. La Paz, Bolivia.

https://www.academia.edu/40172433/_CIPTA_et_al_1999_Etnobota_nica_Tacana

Bourdy, G. et al. (2000) Medicinal plants uses of the Tacana, an Amazonian Bolivian ethnic group. *Journal of Ethnopharmacology* 70(2): 87-109. https://doi.org/10.1016/s0378-8741(99)00158-0

Bourdy, G., Valadeau, C. & Albán, J. (2008) Yato' Ramuesh: Plantas Medicinales Yaneshas. PRODAPP-IRD, Lima. https://horizon.documentation.ird.fr/exl-doc/pleins_textes/divers10-04/010047903.pdf

Boussingault, J.-B. & Roulin, F.D. (1849) Viajes científicos a los Andes ecuatoriales, o colección de memorias sobre física, química e historia natural de la Nueva Granada, Ecuador y Venezuela. París: Librería Castellana, Laserre (Ed.), Imprenta de Beau.

Brady, G. & Clauser, H.R. (1977) Materials Handbook. McGraw-Hill, New York. https://construcaoereparacaonaval.wordpress.com/wp-content/uploads/2017/07/materials-handbook-brady.pdf

Branch, L.C. & da Silva, M.F. (1983) Folk medicine of Alter do Chao, Para, Brazil. *Acta Amazonica* 13: 737-797. https://doi.org/10.1590/1809-4392135737

Brandão, M.G. et al. (1992) Survey of medicinal plants used as antimalarials in the Amazon. *Journal of Ethnopharmacology* 36(2): 175-182. https://doi.org/10.1016/0378-8741(92)90018-m

Breton, R. (1666/1900) Dictionaire françois-caraïbe (Éd. fac-simile; J. Platzmann, Repr.). Leipzig: B. G. Teubner. (2) + 415 pp.

Brewer-Carias, C. & Steyermark, J.A. (1976) Hallucinogenic snuff drugs of the Yanomamo Caburiwe-Teri in the Cauaburi river, Brazil. *Economic Botany* 30 (1): 57-66. https://www.jstor.org/stable/4253692

Brücher, H. (2012) Useful Plants of Neotropical Origin and Their Wild Relatives. Springer Science & Business Media. ISBN: 978-3-642-73313-0. https://link.springer.com/book/10.1007/978-3-642-73313-0

Butt Colson, A. (1973) Inter-tribal trade in the Guiana highlands. *Antropológica, ICAS, Fundación La Salle de Ciencias Naturales* 34: 1–70.

Byg, A. & Balslev, H. (2004) Factors affecting local knowledge of palms in Nangaritza valley, Southeastern Ecuador. *Journal of Ethnobiology* 24: 255-27. https://biostor.org/reference/175096

Caballero-Serrano, V. et al. (2019) Traditional ecological knowledge and medicinal plant diversity in Ecuadorian Amazon home gardens. *Global Ecology and Conservation* 17: e00524. https://doi.org/10.1016/j.gecco.2019.e00524

Cabrera Becerra, G., Franky, C. & Mahecha, D. (1999) Los Nukak: nómadas de la Amazonía colombiana. Unibiblos, Universidad Nacional de Colombia, Bogotá. https://www.researchgate.net/publication/336032482_Los_Nukak_Nomadas_de_la_Amazonia_colombiana

Cadena-Vargas, C., Diazgranados-Cadelo, M. & Bernal-Malagón, H. (2007) Plantas útiles para la elaboración de artesanías de la comunidad indígena Monifue Amena (Amazonas, Colombia). *Universitas Scientiarum-Revista de la Facultad de Ciencias* 12: 97-116. https://revistas.javeriana.edu.co/index.php/scientarium/article/view/4876

Calella, P. (1945) Tabaco entrer las tribus Siona. Amazon. Col. American 3: 39.

Califano, M. (1999) Los indios Sirionó de Bolivia oriental. Ciudad Argentina, Buenos Aires. ISBN: 987-507-106-4. https://editorialciudadargentina.com.ar/productos/los-indios-siriono-de-bolivia-oriental/

Califano, M., & Distel, A.F. (1982) The Use of a Hallucinogenous Plant among the Mashco (Southwestern Amazonia, Peru). *Zeitschrift für Ethnologie* 1: 129-143. https://www.jstor.org/stable/25841801

Cámara-Leret, R., Fortuna, M.A. & Bascompte, J. (2019) Indigenous knowledge networks in the face of global change. *Proceedings of the National Academy of Sciences* 116(20): 9913-9918. https://doi.org/10.1073/pnas.1821843116

Cárdenas, D. & Politis, G.G. (2000) Territorio, Movilidad, Etnobotánica y Manejo del Bosque de los Nukak Orientales. Ediciones Uniandes, Santafé de Bogotá. ISBN: 978-958-695-035-2. http://hdl.handle.net/1992/55969

Cárdenas, D. & Ramírez, J.G. (2004) Plantas útiles y su incorporación a los sistemas productivos del Departamento del Guaviare (Amazonia Colombiana). *Cadalsia* 26: 95-110. https://revistas.unal.edu.co/index.php/cal/article/view/39355

Cárdenas, D. et al. (2002) Plantas útiles en dos comunidades del Departamento de Putumayo. Instituto Amazónico de Investigaciones Científicas, SINCHI, Colombia. https://repositorio.minciencias.gov.co/handle/20.500.14143/32294

Cárdenas, D. et al. (2007) Plantas útiles y promisorias en la comunidad de Wacurabá (Caño Cuduyarí) en el departamento de Vaupés. Bogotá, SINCHI. https://www.sinchi.org.co/files/publicaciones/publicaciones/pdf/wacuraba%20grande.pdf

Cárdenas, M. (1989) Manual de Plantas Económicas de Bolivia. Editorial Los Amigos del Libro, La Paz. ISBN: 9788483701553. https://books.google.ch/books/about/Manual_de_plantas_económicas_de_Bolivia.html?id=64lgAAAAMAAJ&redir_esc=y

Carod-Artal, F.J. (2012) Curares y timbós, venenos del Amazonas. *Revista de Neurología* 55 (11): 689-698. https://doi.org/10.33588/rn.5511.2012343

Cartagena, A. de. (1953) Palabras indígenas relacionadas con "Los Apuntes sobre el baile" en algunas tribus de la región Sur-Oriental de Colombia. *Revista Amazonia Colombiana Americanista* 17-19: 26, 29, 31, 33.

Casement, R. (1910) Roger Casement's Diaries: 1910: The Black and the White. Ed. R. Sawyer, R. (2010). Random House.

Casevits F.M. (1976) Notes sur la pharmacopée des Matsiguenga. *Colloque á Anthropologie des populations andinesà, INSERM, Toulouse*: 129-140.

Castaño-Arboleda, N., Cárdenas, D. & Otavo, E. (2007) Ecología, aprovechamiento y manejo sostenible de nueve especies de plantas del departamento del Amazonas, generadoras de productos maderables y no maderables. Instituto Amazónico de Investigaciones Científicas SINCHI. https://www.sinchi.org.co/ecologia-aprovechamiento-y-manejo-sostenible-de-nueve-especies-de-plantas-del-departamento-del-amazonas-generadoras-de-productos-maderables-y-no-maderables

Castellvi, M. (1946–1950) La famosa "planta de la vida" (*Paullinia yoco*): Iniciales, bibliografía y comentarios. *Revista Amazonia Colombiana Americanista* 4(12–16), 21–27.

Castellví, M. de (1941-44) Materiales etnobotánicos sobre el tabaco y la coca. *Revista Amazonia Colombiana Americanista* 2, nos. 4-6.

Castelnau, F. de. (1851) Expedition dans les parlies centrales de l'Amerique du Sud 1843-47. https://doi.org/10.5962/bhl.title.48609

Cavalcante, P.B. (1972) Frutas comestíveis da Amazônia I. Instituto Nacional de Pesquisas Museu Paraense Emílio Goeldi. Publicaçoes Avulas No. 17. https://repositorio.museu-goeldi.br/handle/mgoeldi/896

Cavalcante, P.B. (1974) Frutas comestíveis da Amazônia II. Instituto Nacional de Pesquisas Museu Paraense Emílio Goeldi. Publicaçoes Avulas No. 27. https://repositorio.museu-goeldi.br/handle/mgoeldi/901

Cavalcante, P.B. (1979) Frutas comestíveis da Amazônia III. Instituto Nacional de Pesquisas Museu Paraense Emílio Goeldi. Publicaçoes Avulas No. 33. https://repositorio.museu-goeldi.br/handle/mgoeldi/768

CEATA Trópico & CI-Bolivia (2007) Transformación del fruto del Majo (Oenocarpus bataua). Recomendaciónes para su aprovechamiento sostenible. Guía para técnicos extensionistas. CI-Bolivia, La Paz.

Cerón, C. & Reyes, C. (2007) Aspectos florísticos, ecológicos y etnobotánica de una hectárea de bosque en la comunidad Secoya Sehuaya, Sucumbíos-Ecuador. Pg. 123-164. In: de la Torre, S. & Yépez, P. (eds.) Caminando sobre el sendero: hacia la conservación del ambiente y la cultura Secoya. Fundación VIHOMA, Quito.

Cerón, C., Reyes, C. & Jiménez, E. (2012) Plantas útiles de los Kichwa, centro-norte de la Amazonia ecuatoriana. *Cinchonia* 12(1): 22-202. https://revistadigital.uce.edu.ec/index.php/CINCHONIA/article/view/2365

Cerón, C.E et al. (2011) Mil y más plantas de la Amazonia ecuatoriana utilizadas por los Secoyas. *Cinchonia* 11: 13-205. https://revistadigital.uce.edu.ec/index.php/CINCHONIA/article/view/2363

Cerón, C.E. (1993) Etnobotánica Quichua en la vía Hollín-Loreto, provincia del Napo. *Hombre y Ambiente* 25: 131-171.

Cerón, C.E. (1993) Manejo florístico Shuar-Achuar (Jívaro) del ecosistema amazónico en el Ecuador. *Hombre y Ambiente* 25: 173-197.

Cerón, C.E. (1995) Etnobiología de los Cofanes de Dureno, provincia de Sucumbíos, Ecuador. Museo Ecuatoriano de Ciencias Naturales. Ediciones Abya-Yala, Quito. ISBN: 9978041559. https://books.google.es/books?id=hpyTeT4a9zkC&hl=es

Cerón, C.E. (2003) Etnobotánica Quichua del Río Yasuní. *Cinchonia* 4: 1-20. https://revistadigital.uce.edu.ec/index.php/CINCHONIA/article/view/2314

Cerón, C.E. & Montalvo, C. (1998) Etnobotánica de los Huaorani de Quehueiri-Ono, Napo-Ecuador. Ediciones Abya-Yala, Quito. https://books.google.ch/books/about/Etnobotánica_de_los_huaorani_de_quehuei.html?id=FIQLLm5qkHMC&redir_esc=y

Cerón, C.E. & Montalvo, C. (2000) Reserva Biológica Limoncocha. Formaciones vegetales, Diversidad y Etnobotánica. *Cinchonia* 1: 1-20. https://revistadigital.uce.edu.ec/index.php/CINCHONIA/article/view/2293

Cerón, C.E. & Montalvo, C. (2002) Etnobotánica Huaorani de Tivacuno-Tiputini Parque Nacional Yasuni. *Cinchonia* 3: 64-94. https://revistadigital.uce.edu.ec/index.php/CINCHONIA/article/download/2311/2290

Cerón, C.E. & Reyes, C.I. (2007) Parches de bosque y etnobotánica Shuar en Palora, Morona Santiago-Ecuador. *Cinchonia* 8: 66-83. https://revistadigital.uce.edu.ec/index.php/CINCHONIA/article/download/2344/2323/9176

Cerón, C.E. et al. (1994) Etnobotánica y notas sobre la diversidad vegetal en la comunidad Cofán de Sinangüé, Sucumbíos, Ecuador. Ecociencia, Quito. ISBN: 9978825339. https://books.google.ch/books/about/Etnobotánica_y_notas_sobre_la_diversida.html?id=QFQLPwAACAAJ&redir_esc=y

Cerón, C.E. et al. (2005) Etnobotánica Quichua Limoncocha, Sucumbíos-Ecuador. *Cinchonia* 6: 29-55. https://revistadigital.uce.edu.ec/index.php/CINCHONIA/article/download/2326/2303/9140

Cerón, C.E. et al. (2005) Etnobotánica Secoya. In: Yépez, P.A. (ed.) Al inicio del Sendero: Estudios Etnobotánicos Secoya. Arboleda, Quito. https://books.google.ch/books/about/Al_inicio_del_sendero.html?id=Zq8NAAAAYAAJ&redir_esc=y

Cerón, C.E. et al. (2006) Estructura, composición y etnobotánica del sendero "Ccottacco Shaiqui", Cuyabeno-Ecuador. *Cinchonia* 7: 82-114. https://revistadigital.uce.edu.ec/index.php/CINCHONIA/article/view/2340

Cerón, C.E. et al. (2012) Plantas útiles de los Kichwa, centro-norte de la Amazonia ecuatoriana. *Cinchonia* 12: 22-202. https://revistadigital.uce.edu.ec/index.php/CINCHONIA/article/view/2365

Cerro, W. et al. (2003) Estudio etnobotánico en las cuencas altas de los ríos Tambopata e Inambari. Proyecto Tambopata Inambari. Pro-Naturaleza, Lima.

Chávez, F. (1996) Estudio preliminar de la familia Arecaceae (Palmae) en el Parque Nacional del Manu (Pakitza y Cocha Cashu). Pp. 141-168. In: Wilson, D.E. & Sandoval, A. (eds.) Manu: The Biodiversity of Southeastern Peru. Editorial Horizonte, Lima.

Chicchon, A. (1992) Chimane resource use and market involvement in The Beni Biosphere Reserve, Bolivia. University of Florida. https://archive.org/details/chimaneresourceu00chic

Cieza de León, P. (1877) Guerra de Salinas, Guerras civiles del Perú (Tomo LXVIII). In M. de la Fuensanta del Valle, J. Sancho Rayón, & F. de Zabalburu (Eds.), Colección de documentos inéditos para la historia de España (534 pp.). Madrid.

Civrieux, M. de (1959) Datos antropológicos de los indios Kunu-hana. *Antropológica* 8: 85–146.

Civrieux, M. de (1980) Los cumanagotos y sus vecinos. En W. Coppens & B. Escalante (Eds.), Los aborígenes de Venezuela (pp. 27–239). Caracas: Fundación La Salle de Ciencias Naturales, Instituto Caribe de Antropología y Sociología (Monografía No. 26).

Claes, F. (1932-34) Chez les indiens Correguajes. *Bul. Soc. Belgue de Geogr.* 56-58: 27-51.

Cobo, B. (1891) Historia del Nuevo Mundo (Tomo II). Sevilla: Sociedad de Bibliófilos Andaluces. (Edición anotada por Marcos Jiménez de la Espada)

Cocco, L. (1987) Iyëwei-theri. Quince años entre los Yanomamos. 2nd Edition. Escuela Técnica Don Bosco, Caracas.

Codazzi, A. (1841) Resumen de la geografía de Venezuela: Formado sobre el mismo plan que el de Balbi y según los conocimientos prácticos adquiridos por el autor en el curso de la Comisión Corográfica que puso a su cargo el gobierno de Venezuela. París: Imprenta de H. Fournier y Compañía.

Coelho-Ferreira, M. (2009) Medicinal knowledge and plant utilization in an Amazonian coastal community of Marudá, Pará State (Brazil). *Journal of Ethnopharmacology* 126(1): 159-175. https://doi.org/10.1016/j.jep.2009.07.016

Coimbra Jr, C.E. (1985) Estudos de ecologia humana entre os Suruí do Parque Indígena Aripuanã, Rondônia. Plantas de importância econômica. *Boletim do Museu Paraense Emílio Goeldi* 2: 37-55. https://acervo.socioambiental.org/acervo/documentos/estudos-de-ecologia-humana-entre-os-surui-do-pqi-aripuanaro-plantas-de

Compte, F. M. (1885) Varones ilustres de la orden seráfica en el Ecuador, desde la fundación de Quito hasta nuestros días (2 vols., 2nd ed., corregida y aumentada por el autor). Quito: Imprenta del Clero.

Condamine, C.M. de la (1745) Relation abrégée d’un voyage fait dans l’intérieur de l’Amérique méridionale. Paris: Veuve Pissot.

Condamine, C.M. de la (1749) *Mém. de l'Acad. Roy. des Sciences, Ann* 430-431.

Coomes, O.T. (2004) Rain forest 'conservation-through-use'? Chambira palm fibre extraction and handicraft production in a a land-constrained community, Peruvian Amazon. *Biodiversity & Conservation* 13: 351-360. https://link.springer.com/article/10.1023/B:BIOC.0000006503.90980.e8

Coomes, O.T. & Ban, N. (2004) Cultivated plant species diversity in home gardens of an Amazonian peasant village in northeastern Peru. *Economic Botany* 58: 420-434. https://doi.org/10.1663/0013-0001(2004)058[0420:CPSDIH]2.0.CO;2

Coomes, O.T. & Burt, G.J. (1997) Indigenous market-oriented agroforestry: dissecting local diversity in western Amazonia. *Agroforestry Systems* 37: 27-44. https://doi.org/10.1023/A:1005834816188

Copeticona, R.C. (2002) Actividad de recolección de frutos silvestres en las comunidades de San Antonio de Matty y Abaroa (Puerto Rico-Pando). Facultad de Ciencias Puras y Naturales. Universidad Mayor de San Andres, La Paz.

Cornejo, M. (1998) Ver, Saber, Poder. Chamanismo de los Yagua de la Amazonía Peruana. IFEA/CAAAP/CAEA-CONICET, Lima. ISBN: 282184591X. https://books.google.ch/books/about/Ver_saber_poder.html?id=MsEWCwAAQBAJ&redir_esc=y

Correa, M.P. (1926) Diccionário das Plantas Úteis do Brasil. Vols. II & VI. Rio de Janeiro. Inst. Bras. De Desenvolvimento Florestal. https://bd.camara.leg.br/bd/items/2f1913cc-6b6c-45c5-a6ee-8697d449f810/full

Costa, R.A. (2013) A identidade e o conhecimento etnobotânico dos moradores da Floresta Nacional do Amapá. Dissertação Mestrado em Biodiversidade Tropical. Universidade Federal do Amapá, Macapá. http://www.iepa.ap.gov.br/biblioteca/tese/2023/RaullyanBorja.Pos.Dr.Relatorio-Final.pdf

Crevaux, J.N. (1883) Voyages dans l'Amerique du Sud. Librairie Hachette et C. https://archive.org/details/voyagesdanslam00crevuoft

Crizón, I. (2001) Por los territorios de la marama. Extracción de la fibra de chiqui chiqui en la amazonía colombiana. Instituto de Estudios Ambientalespara el Desarrollo -IDEADE-, Facultad de Estudios Ambientales y Rurales, Pontificia Universidad

Javeriana, Bogotá. ISBN: 9586833798. https://books.google.co.ve/books?id=Gj5E5-oPSm0C&printsec=copyright#v=onepage&q&f=false

Cuatrecasas, J. (1957) The American Species of *Dacryodes*. *Trop. Woods* 106: 46-65.

Cuervo, A. B. (1894) Colección de documentos inéditos sobre la geografía y la historia de Colombia (Tomo IV, recopilados por Francisco Javier Vergara y V.). Bogotá: Imprenta de Vapor de Zalame.

D'Abbeville, C. (1614) Histoire de la mission des Pères Capucins en l'isle de Maragnan et terres circonvoisines : où est traicté des singularitez admirables & des mœurs merveilleuses des Indiens habitans de ce pais, avec les missives & advis qui ont esté envoyez de nouveau (F. Huby, Ed.). Paris: Imprimerie de François Huby.

d'Orbigny, A.D. (1847) Voyage dans l'Amérique méridionale 1826-1833: (Le Brésil, la République orientale de l'Uruguay, la République argentine, la Patagonie, la République du Chili, la République de Bolivia, la République du Pérou). Paris: P. Bertrand. https://doi.org/10.5962/bhl.title.110540

da Conceição Sena, C. et al. (2019) Análise da comercialização de plantas medicinais no município de Laranjal do Jari-Amapá-Brasil. *Revista Eletrônica Casa de Makunaima* 2(4): 105-110. https://doi.org/10.24979/makunaima.v2i4.687

da Costa, I.B. et al. (2017) Ethnobotanical survey of medicinal flora in the rural community Rio dos Couros, state of Mato Grosso, Brazil. *Boletín Latinoamericano y del Caribe de Plantas Medicinales y Aromáticas* 16(1): 53-67. https://www.redalyc.org/pdf/856/85649119005.pdf

da Silva Braga, M.D.N. et al. (2022) Estudo Etnobotânico De Plantas Medicinais Da Família Fabaceae Na Comunidade Cristolândia, Humaitá-Am. *Biodiversidade* 21(2): 14-26. https://periodicoscientificos.ufmt.br/ojs/index.php/biodiversidade/article/view/14139

da Silva, A.F. et al. (2021) Etnobotânica de plantas medicinais aromáticas: preparações e usos da flora local em cinco comunidades rurais localizadas na região do Baixo Tocantins, Pará, Brasil. *Research, Society and Development* 10(1): e9510111284-e9510111284. https://rsdjournal.org/index.php/rsd/article/download/11284/10294/152904

Dantas, A.R. et al. (2014) Produção de frutos de urucurizeiros, *Attalea excelsa* Mart. (Arecaceae), em floresta de várzea no estuário do rio Amazonas. *Biota Amazonia* (4): 108-114. https://www.alice.cnptia.embrapa.br/alice/bitstream/doc/1006101/1/CPAFAP2014Producaodefrutosurucurizeiros.pdf

Davis, E.W. & Yost, J.A. (1983) The ethnobotany of the Waorani of eastern Ecuador. *Botanical Museum Leaflets* 29: 159-217. https://www.jstor.org/stable/41762848

Davis, W. (1983) The ethnobotany of Chamairo: *Mussatia hyacinthina*. *Journal of Ethnopharmacology* 9: 225-236. https://doi.org/10.1016/0378-8741(83)90033-8

De Alviano, F. (1953) Noticia Etnográfica de los Indios Ticunas. Miscelánea Padre Castellví, 129.

de Athayde, S.F. et al. (2006) Participatory research and management of Arumã (*Ischnosiphon gracilis* [Rudge [Köern., Marantaceae) by the Kaiabi People in the Brazilian Amazon. *Journal of Ethnobiology* 26(1): 36-59. https://doi.org/10.2993/0278-0771(2006)26[36:PRAMOA]2.0.CO;2

De Feo, V. (1991) Uso di piante ad azione antinfiammatoria nell'Alto Ucayali, Perù orientale. *Fitoterapia* 62: 481-494.

De Feo, V. (1992) Medicinal and magical plants in the northern Peruvian Andes. *Fitoterapia* 63: 417-440. https://www.samorini.it/doc1/alt_aut/ad/defeo2.pdf

De Jong, W. (2001) Tree and forest management in the floodplains of the Peruvian Amazon. *Forest Ecology and Management* 150: 125-134. https://doi.org/10.1016/S0378-1127(00)00686-1

De la Torre, L. et al. (2008) Enciclopedia de las Plantas Utiles del Ecuador. Herbario QCA de la Escuela de Ciencias Biológicas de la Pontificia Universidad Católica del Ecuador & Herbario AAU del Departamento de Ciencias Biológicas de la Universidad de Aarhus. https://ddrn.dk/wp-content/uploads/2018/01/Enciclopedia_de_Plantas_U_tiles_del_Ecua.pdf

de Lima Silva, W. et al. (2019) Guia etnobotânico de plantas em comunidades Desano (Tukano-oriental) no rio Tiquié-Brasil. *Cadernos de Etnolingüística* 7(1): 1-42. http://www.etnolinguistica.org/article:vol7n1p1-42

de Menezes Ramos, C. (2018) Segurança alimentar, preservação e conservação ambiental na terra indígena Tenharim do marmelos-amazonas, Brasil: as plantas e suas utilidades. REDE-*Revista Eletrônica do PRODEMA* 11(2): 108-120.

de Oliveira, E.P.B. et al. (2016) Uso, diversidade e conhecimento etnobotânico de plantas medicinais utilizadas para o tratamento da malária no município de Nova Santa Helena-MT. *Boletim do Grupo de Pesquisa da Flora, Vegetação e Etnobotânica* 1(8): 89-108. https://periodicoscientificos.ufmt.br/ojs/index.php/flovet/article/view/4033/2810

de Oliveira, P.C. (2020) Traditional knowledge of forest medicinal plants of Munduruku indigenous people-Ipaupixuna. *European Journal of Medicinal Plants* 31(13): 20-35. https://doi.org/10.9734/ejmp/2020/v31i1330309

de Paula Filho, G.X. et al. (2020) Ethnobotanical knowledge on non-conventional food and medicinal plants in Rio Cajari Extractivist Reserve, Amazon, Brazil. *Research Square*: https://doi.org/10.21203/rs.3.rs-35316/v3

De Pinell, G. (1924). Un viaje por el Putumayo y el Amazonas: ensayo de navegacion. Imprenta Nacional.

DeFilipps, R.A. et al. (2004) Medicinal Plants of the Guianas (Guyana, Surinam, French Guiana). Smithsonian Institution Press, Washington, D.C. https://naturalhistory.si.edu/media/1868

Denevan, W. & Treacy, J.M. (1987) Young managed Fallows at Brillo Nuevo. *Advances in Economic Botany* 5: 8-49. https://www.jstor.org/stable/43919697

Der Marderosian, A.H., Kensinger, K.M., Chao, J.-M. & Goldstein, F.J. (1970) The use and hallucinatory principles of a psychoactive beverage of the Cashinahua tribe (Amazon Basin). Drug Dependence, October 1970, Issue 5, pp. 7–8. National Institute of Mental Health, National Clearing House for Mental Health Information, Chevy Chase, MD.

Descola, P. (1989) La Selva Culta: Simbolismo y praxis en la ecología de los Achuar. Ediciones Abya-Yala y MLAL, Quito. https://doi.org/10.4000/books.ifea.1600

Desmarchelier, C. et al. (1996) Ritual and medicinal plants of the Ese'ejas of the Amazonian rainforest (Madre de Dios, Peru). *Journal of Ethnopharmacology* 52: 45-51. https://doi.org/10.1016/0378-8741(96)01390-6

DeWalt, S.J. et al. (1999) Ethnobotany of the Tacana: quantitative inventories of two permanent plots of northwestern Bolivia. *Economic Botany* 53(3): 237-260. https://doi.org/10.1007/BF02866635

Díaz Piedrahita, S. (1981) Las hojas de las plantas como envoltura de alimentos. Ediciones CIEC, Bogotá.

do Nascimento Martinez, L. et al. (2018) Avaliação etnobotânica de plantas utilizadas como potenciais antimaláricos na região da Amazônia ocidental brasileira. *Interfaces Científicas-Saúde e Ambiente* 6(2): 9-20. https://doi.org/10.17564/2316-3798.2018v6n2p9-20

Dos Santos Silva, J.P.G. & Chaves de Oliveira, P. (2016) Etnobotânica de plantas medicinais na comunidade de Várzea Igarapé do Costa, Santarém-Pará, Brasil. *Ambiente e Sostenibilidad* 6: 136-151. https://doi.org/10.25100/ays.v0i0.4295

dos Santos, J.X. et al. (2016) Caracterizao etnobotãnica de essencias florestais com fins medicinais utilizadas pela etnia Xipaya, no Municipio de Altamira-PA. *Biota Amazonia* 6(2): 1-8. https://periodicos.unifap.br/index.php/biota/article/view/1277

dos Santos, M.R.A. & de Lima, M.R. (2009) Levantamento dos recursos vegetais utilizados como fitoterápicos no Município de Cujubim, Rondônia, Brasil. *Boletim de Pesquisa e Desenvolvimento* 62: 7-17. https://www.infoteca.cnptia.embrapa.br/infoteca/bitstream/doc/710896/1/62recursosvegetaisfitoterapia.pdf

dos Santos, M.R.A., Lima, M.R.D. & Ferreira, M.D.G.R. (2008) Uso de plantas medicinais pela população de Ariquemes, em Rondônia. *Horticultura Brasileira* 26: 244-250. https://doi.org/10.1590/S0102-05362008000200023

Ducke, A. (1938) Plantes nouvelles ou peu connues de la région Amazonienne. *Archivos do Instituto de Biología Vegetal* 4(1). https://www.biodiversitylibrary.org/page/50304923#page/9/mode/1up

Ducke, A. (1945) O gênero *Strychnos* L. na Amazônia brasileira, com a descrição de uma espécie nova: Strychnos pachycarpa n. sp. *Boletim Técnico, Instituto Agronômico do Norte* (3), 1–23.

Ducke, A. (1950) Plantas novas ou pouco conhecidas das Amazônia. *Boletim Técnico, Instituto Agronômico do Norte* 19: 20 (1950).

Ducke, A. (1955) O gênero *Strychnos* no Brasil. *Boletim Técnico, Instituto Agronômico do Norte* 30: 1–64.

Dufour, D.L. & Zarucchi, J.L. (1979) *Monopteryx angustifolia* and *Erisma japura*: Their Use by Indigenous Peoples in the Northwestern Amazon. *Botanical Museum* Leaflets 27(3/4): 69-91. https://www.jstor.org/stable/41762815

Dugand, A. (1961) Palms of Colombia. *Principes* 5: 135-144.

Duke, J.A. & Vasquez, R. (1994) Amazonian Ethnobotanical Dictionary. CRC Press, Florida. https://archive.org/details/amazonianethnobo0000duke

Duke, J.A. & Wain, K.K. (1981) Medicinal Plants of the World. Computer index with more than 85,000 entries, 3 vols.

Echeverri, J.A. & ‘Enokakuiodo’ Román-Jitdutjaaño, O. (2011) Witoto ash salts from the Amazon. *Journal of Ethnopharmacology* 138(2): 492-502. https://doi.org/10.1016/j.jep.2011.09.047

Eder, F.J. (1791/1888) Descripción de la provincia de los Mojos en el Reino del Perú [Original work Descriptio provinciae Moxitarum in Regno Peruano, 1791]

Edwards, W. H. (1847) A voyage up the River Amazon: including a residence at Pará (Vol. 48). J. Murray.

Einzmann, H. (1988) Artesanía indígena del Ecuador: los Cofanes. Revista del Centro Interamericano de Artesanías y Artes Populares 26: 19-88.

Elisabetsky, E. & Posey, D.A. (1989) Use of contraceptive and related plants by the Kayapo Indians (Brazil). *Journal of Ethnopharmacology* 26(3): 299-316. https://doi.org/10.1016/0378-8741(89)90103-7

Ernst, A. (1888) Fischvergiftende Pflanzen. Gesellschaft naturforschender Freunde, Sitzung vom 19. Juni: 111-118. https://www.biodiversitylibrary.org/page/8788864#page/29/mode/1up

Etter, A. (2001) Puinawai y Nukak. Caracterización Ecológica General de dos Reservas Nacionales Naturales de la Amazonía Colombiana. Instituto de Estudios Ambientales para el Desarrollo (IDEAE), Bogotá. https://www.researchgate.net/publication/232080081_Puinawai_y_Nukak_Caracterizacion_ecologica_de_dos_Reservas_Nacionales_Naturales_de_la_Amazonia_colombiana

Fanshawe, D. B. (1954) The genus *Strychnos* in British Guiana. *Brittonia* 8: 65–68.

Fanshawe, D.B. (1953) Fish poisons of British Guiana. *Kew Bulletin* 8(2): 239-240. https://www.jstor.org/stable/4109302

Farabee, W. C. (1918) The Central Arawaks. University of Pennsylvania, The University Museum. (Anthropological Publications, Vol. IX). Philadelphia, PA: University Museum.

Farias, J.E.S. (2012) Manejo de açaizais, riqueza florística e uso tradicional de espécies de várzeas do Estuário Amazônico. Dissertação Mestrado em Biodiversidade Tropical. Universidade Federal do Amapá, Macapá. https://ainfo.cnptia.embrapa.br/digital/bitstream/item/179996/1/CPAF-AP-2012-Dissertacao-Manejo-acaizais-riqueza-floristica.pdf

Feisal, E. (2009) PFNM en seis comunidades campesinas del norte amazónico boliviano: Causas de éxito o fracaso de comercialización. PROMAB, Riberalta.

Fermin, P. (1769). Description générale, historique, géographique et physique de la colonie de Surinam (Tome I, xxiv + 252 pp.; Tome II, 2 + 352 pp.). Amsterdam: E. van Harrevelt.

Fernandes, P. (1996) O uso das plantas psicoativas entre os Hupda. https://acervo.socioambiental.org/sites/default/files/documents/HMD00002.pdf

Ferreira, A.B. et al. (2015) Plants used to treat malaria in the regions of Rio Branco-Acre State and Southern Amazonas State-Brazil. *International Journal of Phytocosmetics and Natural Ingredients*: 2-9. http://dx.doi.org/10.15171/ijpni.2015.09

Ferrero, P.A. (1967) Los Machiguengas. Editorial OPE: Villava-Pamplona. Lima, Peru. https://books.google.ch/books/about/Los_machiguengas.html?id=CCh9AAAAMAAJ&redir_esc=y

Ferreyra, R. (1970) Flora Invasora de los Cultivos de Pucallpa y Tingo Maria. Universidad Nacional Mayor de San Marcos, Lima, Perú.

Figueroa, F. de P. (1659-1661) Relación de las misiones de la Compañía de Jesús en el país de los Maynas (Vol. 1)(1904). Madrid.

Finkers, J. (1986) Los Yanomami y su sistema alimentício. Edición 2 de Monografía / Vicariato Apostólico de Puerto Ayacucho. ISBN: 980265373X. https://books.google.ch/books/about/Los_yanomami_y_su_sistema_alimenticio.html?id=RPErAAAAYAAJ&redir_esc=y

Flores Paitán, S. (1987) Old managed fallows at Brillo Nuevo. *Advances in Economic Botany* 5: 53-66. https://www.jstor.org/stable/43919697

Flores Paitán, S. (1998) Agroforestería amazónica: una alternativa a la agricultura migratoria. Pp. 417-440. In: Kalliola, R. Geoecología y desarrollo Amazónico: estudio integrado en la zona de Iquitos, Perú. *Annales Universitatis Turkuensis Ser. A II.* http://www.iiap.org.pe/upload/publicacion/CDinvestigacion/unap/unap7/UNAP7_PORTADA.HTM

Flores, C.F. & Ashton, P.M.S. (2000) Harvesting impact and economic value of Geonoma deversa, Arecaceae, an understory palm used for roof thatching in the Peruvian amazon. *Economic Botany* 54: 267-277. https://www.jstor.org/stable/4256321

Folkers, K. & Unna, K. (1939) *Archives Internationales de Pharmacodynamie et de Thérapie* 61: 370.

Forero, M.C. (2005) Aspectos etnobotánicos de uso y manejo de la familia Arecaceae (palmas) en la comunidad indígena Ticuna de Santa Clara de Tarapoto, del resguardo Ticoya del municipio de Puerto Nariño, Amazonas, Colombia. Facultad de Estudios Ambientales y Rurales. Pontificia Universidad Javeriana, Bogotá.

Franco, J.W. (2002) Etnobotánica de la yanchama (*Ficus* spp: MORACEAE) Amazonas, Colombia. Trabajo de grado. Carrera de Biología. Facultad de Ciencias. Pontificia Universidad Javeriana. Bogota. https://apidspace.javeriana.edu.co/server/api/core/bitstreams/d2a9cc54-ef5e-464e-b63b-1e6c5ee1422c/content

Freire, B.H. (2006) Ethnobotany of the Huaorani communities in the Ecuadorian Northwest. *Lyonia* 10(2): 7-17. https://www.lyonia.org/downloadPDF-2.390.pdf?pdfID=2.390.

Fritz, S. (1686-1723) Journal of the travels and labours of Father Samuel Fritz in the River of the Amazons between 1686 and 1723: Translated from the Evora MS (G. Edmundson, Trans. & Ed.). Hakluyt Society.

Fróes, R. L. (1959). Les strychnées sud-américaines et leur utilisation dans la préparation des curares par les Indiens. In D. Bovet, F. Bovet-Nitti, & G. B. Marini-Bettòlo (Eds.), Curare and curare-like agents (pp. 83–112). Elsevier.

Fuentes, E. (1980) Los Yanomami y las plantas silvestres. *Antropológica* 54: 3-138. http://biblioteca.funai.gov.br/media/pdf/REVISTAS/antropologica/MFN-11270.pdf

Furtado Santos, J.J., Coelho-Ferreira, M. & Costa-Lima, P.G. (2018) Etnobotanica de plantas medicinais em mercados publicos da regiã o metropolitana de Belem do Parã, Brasil. *Biota Amazonia* 8(1): 1-9. https://core.ac.uk/download/pdf/233922111.pdf

Galeano, G. (1992) Las Palmas de la Región de Araracuara. Tropenbos-Colombia, Bogotá. https://www.tropenbos.org/file.php/1628/tbi-col-1.pdf

Galeano, G. & Bernal, R. (2010) Palmas de Colombia. Guía de Campo. Editorial Universidad Nacional de Colombia. Instituto de Ciencias Naturales, Facultad de Ciencias-Universidad Nacional de Colombia, Bogotá. ISBN:978-958-719-501-9.

García Barriga, H. (1974) Flora Medicinal de Colombia. Botánica Médica. Instituto de Ciencias Naturales, Universidad Nacional, Bogotá. 3 vols.

García, L.J. et al. (1996) Principales especies nativas de fauna y flora del Caquetá, usos actuales y potenciales. PNR/CORPOICA, Regional Amazonía, Florencia. http://hdl.handle.net/20.500.12324/17135

Garzón, C. & Macuritofe, V. (1992) La noche, las plantas y sus sueños: Aproximación al conocimiento botánico en una cultura amazónica. Corporación Colombiana para la Amazonia, Bogotá. ISBN: 958953791X.

Garzón, N.C. (1985) Aproximación etnobotánica en la comunidad Guayabero de Barrancion-Guaviare. Facultad de Ciencias Humanas. Tesis de Grado de Antropología. Universidad Nacional de Colombia, Bogotá.

Gaspar de Carvajal (1542) Descubrimiento del río de las Amazonas según la relación hasta ahora inédita de Fr. Gaspar de Carvajal con otros documentos referentes á Francisco de Orellana y sus compañeros. Imprenta de E. RASCO, Bustos Tavera, núm. I. MDCCCXCIV.

Gentry, A.H., & Wettach, R.H. (1986) *Fevillea*-a new oil seed from Amazonian Peru. *Economic Botany* 40(2): 177-185. https://doi.org/10.1007/BF02859141

Gilmore, M.P., Eshbaugh, W.H. & Greenberg, A.M. (2002) The use, construction, and importance of canoes among the Maijuna of the Peruvian Amazon. *Economic Botany* 56: 10-26. https://www.jstor.org/stable/4256516

Giovannini, P. (2015) Medicinal plants of the Achuar (Jivaro) of Amazonian Ecuador: Ethnobotanical survey and comparison with other Amazonian pharmacopoeias. *Journal of Ethnopharmacology* 164: 78-88. https://doi.org/10.1016/j.jep.2015.01.038

Girard, R. (1958) Indios Selváticos de la Amazonia Peruana. Libro Mex, México. https://books.google.ch/books/about/Indios_selváticos_de_la_Amazonía_perua.html?id=VvMXAAAAIAAJ&redir_esc=y

Glenboski, L.L. (1983) The Ethnobotany of the Tikuna Indians. Universidad Nacional de Colombia, Bogota.

Gomez, D. et al. (1996) Palmas útiles en la provincia de Pastaza. Amazonia ecuatoriana. Manual práctico. Serie Manuales de plantas útiles amazónicas 1. Fundación Omaere. Quito

González-Pérez, S.E. et al. (2012) Conhecimento e usos do babaçu (*Attalea speciosa* Mart. e *Attalea eichleri* (Drude) AJ Hend.) entre os Mebêngôkre-Kayapó da Terra Indígena Las Casas, estado do Pará, Brasil. *Acta Botanica Brasilica* 26(2): 295-308. https://doi.org/10.1590/S0102-33062012000200007

Grández, C.A. & Henderson, A. (1993) A new record of Manicaria for Peru. *Principes* 37: 159-160. https://palms.org/wp-content/uploads/2016/07/vol37n3.pdf

Grenand, P. (1980) Introduction d l'étude de l'univers Wayapi. Ethnoécologie des Indiens du Haut-Oyapock (Guyane française). SELAF, Paris. https://www.persee.fr/doc/hom_0439-4216_1983_num_23_2_368392

Grenand, P., Moretti, C., & Jacquemin, H. (1987) Pharmacopées traditionnelles en Guyane (Mémoire No. 108). ORSTOM.

Guallart, J.M. (1968) Nomenclatura Jibaro-Aguaruna de Palmeras en el Distrito de Cenepa. *Biota* 7: 230-251.

Guèze, M. et al. (2014) Are ecologically important tree species the most useful? A case study from indigenous people in the Bolivian Amazon. *Economic Botany* 68: 1-15. https://doi.org/10.1007/s12231-014-9257-8

Gumilla, J. (1791) Historia natural, civil y geografica de las naciones situadas en las riveras del rio Orinoco. 2 Vols. https://babel.banrepcultural.org/digital/collection/p17054coll10/id/3325/

Guppy, N. (1958) Wai-Wai: Through the forests north of the Amazon. John Murray.

Gutiérrez-Vásquez, C.A. & Peralta, R. (2001) Palmas comunes de Pando, Santa Cruz de la Sierra, Bolivia. PANFOR/OIMT/BOLFOR. https://www.itto.int/files/user/pdf/publications/PD%2024%2097/pd24-97-1%20rev1(F)%20s.pdf

Gutierrez, G. (1943) Estudio sobre los principales barbascos colombianos. *Revista Fc. Nac. Agron. Bogotá* 7: 77-93.

Gutiérrez, J.M.B. et al. (2014) Wild medicinal plants used by Colombian Kofan Indians to treat cutaneous leishmaniasis. *Revista Cubana de Plantas Medicinales* 19(4): 407-420. http://scielo.sld.cu/scielo.php?script=sci_arttext&pid=S1028-47962014000400012

Guyot, M. (1972) La maison des Indiens Bora et Miraña. *Journal de la Société des Americanistes* 61: 141-176. https://www.persee.fr/doc/jsa_0037-9174_1972_num_61_1_2116

Guyot, M. (1979) La historia del mar de danta, el Caqueta: una fase de la evolucion cultural en el noroeste amazonico. *Journal de la Société des Americanistes* 66: 99-123. https://www.persee.fr/doc/jsa_0037-9174_1979_num_66_1_2173

Hajdu, Z., & Hohmann, J. (2012) An ethnopharmacological survey of the traditional medicine utilized in the community of Porvenir, Bajo Paraguá Indian Reservation, Bolivia. *Journal of Ethnopharmacology* 139(3): 838-857. https://doi.org/10.1016/j.jep.2011.12.029

Hamlin, C.C. & Salick, J. (2003) Yanesha agriculture in the upper Peruvian Amazon: Persistence and change fifteen years down the 'road'. *Economic Botany* 57: 163-180. https://www.jstor.org/stable/4256676

Hansson, A. et al. (1986) Preclinical and clinical studies with latex from *Ficus glabrata* HBK, a traditional intestinal anthelminthic in the Amazonian area. *Journal of Ethnopharmacology* 17(2): 105-138. https://doi.org/10.1016/0378-8741(86)90053-x

Hardenburg, W.E. (1912) The Putumayo: The devil's paradise; travels in the Peruvian Amazon region and an account of the atrocities committed upon the Indians therein. T. Fisher Unwin, London.

Harner, M.J. (1984) The Jívaro. People of the Sacred Waterfalls. University of California Press, Berkeley. https://books.google.ch/books/about/The_Jivaro.html?id=APziu1G7TtoC&redir_esc=y

Harner, M.J., 1968. The sound of rushing water: A hallucinogenic drug gives the Jivaro shaman entrance to the 'real world' and gives him the power to cure or bewitch. *Natural History Magazine*, June–July 1968, pp. 28–29. © The American Museum of Natural History

Hartwell, J.L. (1982) Plants used against cancer: a survey. Quarterman Publications, Inc. Lawrence, MA.

Haverroth, M., Negreiros, P.R.M. & Barros, L.C.P. (2010) Ethnobiology and health among the Kulina people from the Upper Envira River, State of Acre, Brazil. *The Open Complementary Medicine Journal* 2: 43-57. https://benthamopen.com/contents/pdf/TOALTMEDJ/TOALTMEDJ-2-42.pdf

Hegnauer, R. (1964) Chemotaxonomie der Pflanzen-Birkhauser Verlag. Basel. Vols. 1-6. https://link.springer.com/book/10.1007/978-3-0348-7985-9

Heizer, R.F. (1953) Aboriginal fish poisons. *Bureau of American Ethnology Bulletin* 151(38): 231-283. https://repository.si.edu/handle/10088/22079

Henderson, A. & Chávez, F. (1993) *Desmoncus* as a useful palm in the western Amazon basin. *Principes* 37: 184-186.

Henkemans, A. (2001) Tranquilidad and Hardship in the Forest: Livelihoods and Perceptions of Camba Forest dwellers in the northern Bolivian Amazon. PROMAB Scientific Series, volume 5. Riberalta, Bolivia. https://dspace.library.uu.nl/handle/1874/810

Heriarte, M. de. (1874). Descrição do Estado do Maranhão, Pará, Corupá e rio das Amazonas: Feita por ... Ouvidor-geral, Provedor-mor e Auditor, que foi, pelo Governador D. Pedro de Mello, no anno de 1662. Por mandado do Governador-geral Diogo Vaz de Sequeira. Dada à luz pela primeira vez. Viena d'Áustria: Imprensa do Filho de Carlos Gerold.

Hibert, F. et al. (2011) Botany, genetics and ethnobotany: a crossed investigation on the elusive tapir's diet in French Guiana. *PLoS One* 6(10): e25850. https://doi.org/10.1371/journal.pone.0025850

Hinojosa, I. (1991) Plantas utilizadas por los Mosetenes de Santa Ana (Alto Beni, Depto. La Paz). Tesis de Licenciatura en Biología. Universidad Mayor de San Andres, La Paz. 88 pp.

Hinojosa, I. et al. (2001) Los Yuracaré: su conocimiento, experiencia y la utilización de recursos vegetales en el río Chapare. Instituto de Ecología, Universidad Mayor de San Andrés, La Paz.

Hissink, K. & Hahn, A. (2000) Los Tacana. Datos sobre la historia de su civilización. Plural Editores, La Paz.

Hoehne, F. C. (1910). História natural: Botânica. Parte I (Anexo No. 5). In Comissão de Linhas Telegráficas Estratégicas de Matto Grosso ao Amazonas. Rio de Janeiro. https://www.biodiversitylibrary.org/item/113619

Hoehne, F. C. (1951) Índice bibliográfico e numérico das plantas colhidas pela Comissão Rondon ou Comissão de Linhas Telegráficas Estratégicas de Mato-Grosso ao Amazonas, de 1908 até 1923. Instituto de Botânica, São Paulo.

Hoehne, F.C. (1937) Botanica e Agricultura no Brasil no seculo XVI. *Brasiliana* 71: 1-410. http://etnolinguistica.wdfiles.com/local--files/biblio%3Ahoehne-1937-botanica/Hoehne_1937_Botanica_e_agricultura_Brasil_sec_XVI.pdf

Hoffman, B. & Ruysschaert, S. (2017) Lianas of the Guianas: a field guide to woody climbers in the tropical forests of Guyana, Suriname and French Guiana. LM Publishers.

Holm-Jensen, O. & Balslev, H. (1995) Ethnobotany of the fiber palm *Astrocaryum chambira* (Arecaceae) in Amazonian Ecuador. *Economic Botany* 49(3): 309-319. https://www.jstor.org/stable/4255747

Holmberg, A.R. (1978) Nómadas del arco largo: los Sirionó del oriente boliviano. Instituto Indigenista Interamericano, México.

Horackova, J. et al. (2023) Ethnobotanical inventory of medicinal plants used by Cashinahua (Huni Kuin) herbalists in Purus Province, Peruvian Amazon. *Journal of Ethnobiology and Ethnomedicine* 19(1): 16. https://doi.org/10.1186/s13002-023-00586-4

Howes, F.N. (1930) Fish-poison plants. *Kew Bulletin* 4: 129-153. https://doi.org/10.2307/4107559

Huanca, T. (1999) Tsimane Indigenous Knowledge, Swidden Fallow Management and Conservation. PhD Thesis. University of Florida, Gainesville. https://ufdcimages.uflib.ufl.edu/AA/00/04/86/35/00001/tsimaneindigenou00huan.pdf

Huber, J. (1900) Arboretum Amazonicum: Iconographia dos mais importantes vegetaes espontaneos e cultivados da região Amazonica / Iconographie des plantes spontanées et cultivées les plus importantes de la région Amazonienne. 1a Decada. Pará: Museu Paraense de História Natural e Ethnographia.

Huber, J. (1900) Arboretum Amazonicum: Iconographia dos mais importantes vegetaes espontaneos e cultivados da região Amazonica / Iconographie des plantes spontanées et cultivées les plus importantes de la région Amazonienne. 2a Decada. Pará: Museu Paraense de História Natural e Ethnographia.

Huber, J. (1906) Arboretum Amazonicum: Iconographia dos mais importantes vegetaes espontaneos e cultivados da região Amazonica / Iconographie des plantes spontanées et cultivées les plus importantes de la région Amazonienne. 3a Decada. Pará: Museu Paraense de História Natural e Ethnographia.

Huber, J. (1906) Arboretum Amazonicum: Iconographia dos mais importantes vegetaes espontaneos e cultivados da região Amazonica / Iconographie des plantes spontanées et cultivées les plus importantes de la région Amazonienne. 4a Decada. Pará: Museu Paraense de História Natural e Ethnographia.

Hübschmann, L.K. et al. (2007) Uses of Vara Casha - a Neotropical Liana Palm, Desmoncus polyacanthos - in Iquitos, Peru. *Palms* 51: 167-176. https://palms.org/wp-content/uploads/2016/05/v51n4p167-176-1.pdf

Huertas Castillo, B. (2007) Kamoua Nupanempua Yaimirute. Nuestro territorio Kampu Piyawi-Shawi. Terra Nuova, Lima.

Humboldt, A. von, & Bonpland, A. (1818) Personal narrative of travels to the equinoctial regions of the New Continent, during the years 1799–1804 (H. M. Williams, Trans.; Vol. 3). London: Longman, Hurst, Rees, Orme, and Brown.

Humboldt, A. von, & Bonpland, A. (1819) Personal narrative of travels to the equinoctial regions of the New Continent, during the years 1799–1804 (H. M. Williams, Trans.; Vol. 4). London: Longman, Hurst, Rees, Orme, and Brown.

Humboldt, A. von, & Bonpland, A. (1821) Personal narrative of travels to the equinoctial regions of the New Continent, during the years 1799–1804 (H. M. Williams, Trans.; Vol. 5). London: Longman, Hurst, Rees, Orme, and Brown.

Iglesias, G. (1987) Hierbas medicinales de los Quichua del Napo. Abya-Yala, Quito.

Iglesias, G. (1989) Hierbas medicinales de los Quichua del Napo. Enfermedades femeninas y enfermedades del "susto". Abya-Yala, Quito.

Iglesias, G. (1989) Sacha Jambi: El uso de las plantas en la medicina tradicional de los Quichuas del Napo. Ediciones Abya-Yala, Quito.

Igualada, B. de (1938) Descubrimientos de nuevas tribus indígenas. Tres emocionantes exploraciones misionales en el río Cahuinari. *Boletín de Estudios Históricos* VII (83).

Im Thurn, E.F. (1883) Among the Indians of Guiana: being sketches chiefly anthropologic from the interior of British Guiana. K. Paul, Trench & Company.

Irvine, D. (1989) Succesion Management and Resource Distribution in an Amazonian Rain Forest. *Advances in Economic Botany* 7: 223-237. https://www.jstor.org/stable/43927554

Játiva, V. & Alarcón, R. (1994) Sobre la etnobotánica y la comercialización de la ungurahua, Oenocarpus bataua (Arecaceae), en la zona del Alto Napo, Ecuador. Pp. 53-89. In: Alarcón, R., Mena, P.A. & Soldi, A. (eds.) Etnobotánica, valoración económica y comercialización de recursos florísticos silvestres en el Alto Napo. Ecuador. Ecociencia, Quito.

Jernigan, K.A. (2009) Barking up the same tree: a comparison of ethnomedicine and canine ethnoveterinary medicine among the Aguaruna. *Journal of Ethnobiology and Ethnomedicine* 5(1): 1-9. https://doi.org/10.1186/1746-4269-5-33

Jiménez de la Espada, M. (1889) Noticias auténticas del famoso río Marañón y misión apostólica de la Compañía de Jesús de la provincia de Quito en los dilatados bosques de dicho río: Escribíalas por los años de 1738 un misionero de la misma Compañía, y las publica ahora por primera vez. Madrid: Establecimiento tipográfico de Fontanet; Impresor de la Real Academia de la Historia.

Jiménez de la Espada, M. (1904) Vocabulario de la lengua general de los indios del Putumayo y Caquetá, publicado con una introducción por el autor (Revista de Archivos, Bibliotecas y Museos, año 1898). Madrid: [s.n.].

Jiménez de la Espada, M. (Ed.)(1895) Relaciones geográficas de Indias: Perú (Tomo II). Madrid: Ministerio de Fomento.

Jiménez de la Espada, M. (Ed.)(1897) Relaciones geográficas de Indias: Perú (Tomo IV). Madrid: Ministerio de Fomento.

Jitdutjaaño, R., Romualdo, O., Román Sánchez, S., & Echeverri, J.A. (2020) ɨairue nagɨni Aiñɨko urukɨ nagɨni Aiñɨra urukɨ nagɨni Halogeno–Halofita Sal de vida.

Jobert, C. (1878) Sur la preparation du Curare. *Comptes rendus de l'Académie des Sciences* 86: 121-122.

Jobert, C. (1879) Sur l'action physiologique des Strychnées de l'Amérique du Nord. *Comptes Rendus de l'Académie des Sciences* 89: 646

Johnson, D. (1975) Some palm products of the Peruvian Amazon. *Principes* 19: 78-79.

Johnson, D. & Mejía, K. (1998) The making of a dugout canoe from the trunk of the palm *Iriartea deltoidea*. *Principes* 42: 201-205, 208. https://palms.org/wp-content/uploads/2016/05/vol42n4p201-205208.pdf

Jordan, C.B. (1970) A study of germination and use in twelve palms of Northeastern Peru. Principes 14: 26-32. https://palms.org/wp-content/uploads/2016/05/v14n1p26-32.pdf

Kahn, F. & Mejía, K. (1987) Notes on the biology, ecology, and use of a small Amazonian palm: *Lepidocaryum tessmannii*. *Principes* 31: 14-19.

Kainer, K.A. & Duryea, M.L. (1992) Tapping women's knowledge: Plant resource use in extractive reserves, Acre, Brazil. *Economic Botany* 46(4): 408-425. https://doi.org/10.1007/BF02866513

Kamen-Kaye, D. (1977) Ichthyotoxic plants and the term" barbasco". *Botanical Museum Leaflets* 25(2): 71-90. https://www.jstor.org/stable/41762777

Karsten, R. (1935) The head-hunters of Western Amazonas: The life and culture of the Jíbaro Indians of Eastern Ecuador and Peru. *Commentationes Humanarum Litterarum, Societas Scientiarum Fennica* VII(1), pp. 124, 343–345, 438-41.

Karsten, R. (1988) La vida y la cultura de los Shuar. Tomo I. Abya-Yala, Quito. https://abyayala.org.ec/producto/la-vida-y-cultura-de-los-shuar/?srsltid=AfmBOooXQY0EgnB2Ek_ViBzWoX2qd4czevNm7q36VXoPi_8suhtoOt8g

Keller, F. (1875) The Amazon and Madeira rivers: sketches and descriptions from the note-book of an explorer. Chapman and Hall.

Kerharo, H., Guichard, F. & Bouquet, A. (1959-1961) Les végétaux ichtyotoxiques (poison de peche). In: Kerharo, J. (ed.) Biolog Biologie Végétale et Matière Médicale. *Bulletins et Mémoires de l'Ecole Nationale de Médecine et de Pharmacie de Dakar* 8: 314-329: 9: 355-386: 10: 223-242. https://horizon.documentation.ird.fr/exl-doc/pleins_textes/pleins_textes_5/b_fdi_16-17/22355.pdf

Kermath, B.M., Bennett, B.C., & Pulsipher, L.M. (2018) Food Plants in the Americas: A Survey of the Domesticated, Cultivated, and Wild Plants Used for Human Food in North, Central, and South America and the Caribbean. https://www.researchgate.net/publication/263888295_Food_Plants_in_the_Americas_A_Survey_of_the_Domesticated_Cultivated_and_Wild_Plants_Used_for_Human_Food_in_North_Central_and_South_America_and_the_Caribbean

Kerr, W.E., Posey, D.A. & Wolter Filho, W. (1978) Cupá, ou cipó-babão, alimento de alguns índios amazônicos. *Acta Amazonica* 8: 702-705. https://www.scielo.br/j/aa/a/4cRNsyvfMkr7PjfBVVXNF8R/?format=pdf

Kffuri, C.W. (2014) Etnobotânica de plantas antimaláricas em comunidades indígenas da região do Alto Rio Negro-Amazonas-Brasil. PhD Thesis. Universidade Estadual Paulista. https://repositorio.unesp.br/entities/publication/ec7b7536-0f66-4550-abef-7492a30404af

Killip, E.P. & Smith, A.C. (1935) Some American plants used as fish poisons. Bureau of Plant Industry. USDA, Pp. 1-27.

Koch-Grünberg, T. (1906) Die Makú. *Anthropos* 1 (4): 877-906. https://www.jstor.org/stable/40442165

Koch-Grünberg, T. (1906) Les Indiens Ouitotos: étude linguistique. *Journal de la Société des Américanistes* 3(2): 157–189.

Koch-Grünberg, T. (1909–1910) Zwei Jahre unter den Indianern: Reisen in Nord-West-Brasilien, 1903–1905. 2 vols. Berlin: Ernst Wasmuth.

Koch-Grünberg, T. (1917–1928) Vom Roroima zum Orinoco: Ergebnisse einer Reise in Nordbrasilien und Venezuela in den Jahren 1911–1913. 5 vols. Stuttgart: Strecker & Schroeder, vol. III, p. 386.

Kronik, J. et al. (1999) Fééjahisuu. Palmas de los Nietos de la Tierra y Montaña Verde del Centro. Centro de Investigación y Desarrollo, Copenhague. ISBN: 8788467414.

Krukoff, B.A. & Monachino, J. (1946) Supplementary notes on the American species of *Strychnos* II. *Lloydia* 9: 62–72.

Krukoff, B.A. (1965) Supplementary notes on the American species of *Strychnos* VII. *Memoirs of the New York Botanical Garden* 12(2): 7, 42-43. https://www.biodiversitylibrary.org/part/264187

Krukoff, B.A. (1972) American species of *Strychnos*. *Lloydia* 35: 193–271.

Krukoff, B.A. (1976). Supplementary notes on American Menispermaceae. XI. Neotropical Triclisieae and Anomospermeae. *Phytologia* 33: 323–341.

Krukoff, B.A. (1977) Supplementary notes on the American species of Strychnos XV. *Phytologia* 36: 17–22.

Krukoff, B.A. (1979) Supplementary notes on American Menispermaceae — XIV. Neotropical Triclisieae and Anomospermeae. *Phytologia* 41: 239-255.

Krukoff, B.A. & Barneby, R.C. (1969) Supplementary notes on the American species of *Strychnos* VIII. *Memoirs of the New York Botanical Garden* 20(1): 1–93.

Krukoff, B.A. & Barneby, R.C. (1969) Supplementary notes on the American species of Strychnos X. *Phytologia* 19: 176-185.

Krukoff, B.A. & Barneby, R.C. (1970) Supplementary notes on American Menispermaceae VI. *Memoirs of the New York Botanical Garden* 20(2): 1–70.

Krukoff, B.A. & Barneby, R.C. (1970) Supplementary notes on American Menispermaceae. VIII. *Memoirs of the New York Botanical Garden* 20(2): 71–146.

Krukoff, B.A. & Barneby, R.C. (1972) Supplementary notes on American Menispermaceae IX. *Phytologia* 25: 32–48.

Krukoff, B.A. & Barneby, R.C. (1973) Supplementary notes on the American species of *Strychnos* XIII. *Phytologia* 27: 97–107.

Krukoff, B.A. & Moldenke, H.N. (1938) Studies of American Menispermaceae, with special reference to species used in preparation of arrow-poisons. *Brittonia* 3(1): 1-74. https://doi.org/10.2307/2804992

Krukoff, B.A. & Moldenke, H.N. (1951) Supplementary notes on American Menispermaceae V. *Bulletin of the Torrey Botanical Club* 78(3): 258–272.

Krukoff, B.A. & Monachino, J. (1942) The American species of *Strychnos*. *Brittonia* 4: 248-332. https://doi.org/10.2307/2804715

Krukoff, B.A. & Monachino, J. (1946) The genus *Strychnos* in Venezuela. *Darwiniana* 7(2): 185-193. https://www.jstor.org/stable/23211624

Krukoff, B.A. & Monachino, J. (1947) Supplementary Notes on the American Species of *Strychnos* IV. *Bol. Tec. Inst. Agron. Belem* 11: 3-15. http://www.alice.cnptia.embrapa.br/alice/handle/doc/381775

Krukoff, B.A. & Monachino, J. (1947) Supplementary notes on the American species of *Strychnos* V. *Boletim Técnico, Instituto Agronômico do Norte* (12): 5–16.

Krukoff, B.A. & Monachino, J.V. (1950) Supplementary notes on the American species of *Strychnos* VI. *Boletim Técnico, Instituto Agronômico do Norte* (20): 3.

Krukoff, B.A. & Smith, A.C. (1937) Notes on the botanical components of curare. *Bulletin of the Torrey Botanical Club* 64(6): 401-409. https://doi.org/10.2307/2481123

Krukoff, B.A. & Smith, A.C. (1937) Rotenone-yielding plants of South America. American *Journal of Botany* 24(9): 573-587. https://doi.org/10.1002/j.1537-2197.1937.tb09149.x

Krukoff, B.A. & Smith, A.C. (1939) Notes on the botanical components of curare-II. *Bulletin of the Torrey Botanical Club* 66(5): 305-314. https://doi.org/10.2307/2480853

Kvist, L. et al. (2001) Extraction from woody plants in flood plain communities in Amazonian Peru: use, choice, evaluation and conservation status of resources. *Forest Ecology and Management* 150: 147-174. https://doi.org/10.1016/S0378-1127(00)00688-5

Kvist, L.P. & Holm-Nielsen, L.B. (1987) Ethnobotanical aspects of lowland Ecuador. *Opera Botanica 92*: 83–107.

Kvist, L.P., Oré-Balbín, I.C. & Llapapasca-Samaniego, D.C. (1998) Plantas utilizadas en trastornos ginecológicos, parto y control de natalidad en mujeres de la parte baja del río Ucayali, Amazonas Peruana. *Folia Amazonica* 9(1-2): 115-141. https://doi.org/10.24841/fa.v9i1-2.171

La Rotta, C. (1983) Observaciones etnobotánicas sobre algunas especies utilizadas por la comunidad indígena Andoque (Amazonas, Colombia). Universidad de Colombia, Bogotá.

La Rotta, C. et al. (1987) Estudio etnobotánico sobre las especies utilizadas por la comunidad indígena Miraña, Amazonas, Colombia. World Wildlife Fundation, Bogotá.

La Rotta, C. et al. (1989) Especies utilizadas por la Comunidad Miraña. Estudio Etnobotánico. Fondo para la protección del medio ambiente José Celestino Mutis, FEN Colombia. Bogotá.

Labesse, P. (1905) Le curare. *Mémoires de la Société d'Agriculture, Sciences et Arts d'Angers* (7): 231-240.

Lambda, S.S. (1970) Indian piscidal plants. *Economic Botany* 24: 134-136. https://doi.org/10.1007/BF02860591

Langevin, M. (2002) Mapajo. Nuestra selva y cultura. PRAIA (FIDA/CAF), La Paz.

Latcham, R. E. (1936) La agricultura precolombina en Chile y los países vecinos. Santiago: Ediciones de la Universidad de Chile. viii + 336 pp.

Laureano de la Cruz, Fray. (1651) Nuevo descubrimiento del río de las Amazonas, hecho por los misioneros de la Provincia de San Francisco de Quito, el año 1651 (Biblioteca Amazonas, Vol. VII)(1941). Imprenta del Ministerio de Gobierno, Quito.

Le Cointe, P. (1947) Ávores e plantas úteis:(indígenas e aclimadas). *Brasiliana* 251: 1-506. http://bdor.sibi.ufrj.br/handle/doc/337

Lévi-Strauss, C. (1952) The use of wild plants in tropical South America. *Economic Botany* 6(3): 252-270. https://doi.org/10.1007/BF02985068

Lewis, W.H. & Elvin-Lewis, M.P. (1977) Medical Botany: Plants Affecting Human Health. John Wiley & Sons, NY. 515 pp.

Lima, R.B., de Medeiros, F.A. & Souto, R.N.P. (2019) Espécies vegetais usadas como repelentes e inseticidas no estado do Amapá, BR. *Revista Brasileira de Agroecologia* 14(3): 40-53. https://doi.org/10.33240/rba.v14i3.22799

Lira, J.A. (1945) Diccionario K-kechuwa–Español. Tucumán: Universidad Nacional de Tucumán, Departamento de Lingüística y Folklore; Talleres Gráficos Miguel Violetto.

Lizot, J. (1984) Les Yanõmami Centraux. Éditions de l'École des hautes Études en Sciences sociales, Paris. 266 pp.

López Camacho, R. (2006) Manual de identificación de especies no maderables del corregimiento de Tarapacá, Colombia. Bogota, Sinchi. https://sinchi.org.co/manual-de-identificacion-de-especies-no-maderables-del-corregimiento-de-tarapaca

López Ruiz, S. J. (1783/1883). Relación del viaje que de la capital de Santafé de Bogotá hizo a las montañas de los Andaquíes y misiones de los ríos Caquetá y Putumayo (AIP, Bogotá, junio de 1883).

López-Parodi, J. (1988) The use of palms and other native plants in non-conventional, low cost rural housing in the Peruvian Amazon. *Advances in Economic Botany* 6: 119-129. https://www.jstor.org/stable/43927523

López, R., Cárdenas, D. & Marín, C. (1998) Plantas de Uso Potencial (no maderable) en el Norte del Departamento del Guaviare, Amazonía Colombiana. The Field Museum and The Andrew Mellon Foundation, Chicago.

Lozano Balcázar, A. (2005) Los barbascos utilizados por los Ticuna del PNN Amacayacu (Bachelor's thesis, Uniandes). http://hdl.handle.net/1992/22199

Luna, L.E. (1982) El concepto de plantas que enseñan, entre los cuatro Shamanes mestizos de Iquitos nordeste del Perú. Revista Colombiana de Antropología 24: 45-66. https://doi.org/10.22380/2539472X.1518

Luziatelli, G. et al. (2010) Asháninka medicinal plants: a case study from the native community of Bajo Quimiriki, Junín, Peru. *Journal of Ethnobiology and Ethnomedicine* 6: 21. https://doi.org/10.1186/1746-4269-6-21

Macbride, J.F. (1936-) Flora of Perú. Field Museum of Natural History, Botanical Services, Chicago.

Macbride, J.F. (1956) Flora of Peru. Vol. 13 (3a) No. 2: 291-744. Field Museum of Natural History, Chicago.

Macbride, J.F. (1960) Flora of Peru. Vol. 13 (1) No. 2. Field Museum of Natural History, Chicago.

Macía, M.J. (2004) A comparison of useful pteridophytes between two Amerindian groups from Amazonian Bolivia and Ecuador. *American Fern Journal* 94(1): 39-46. https://www.jstor.org/stable/1547253

Macía, M.J. (2004) Multiplicity in palm uses by the Huaorani of Amazonian Ecuador. *Botanical Journal of the Linnean Society* 144: 149-159. https://doi.org/10.1111/j.1095-8339.2003.00248.x

Macía, M.J. unpubl., cited in: Macía, M.J. et al. (2011) Palm uses in northwestern South America: a quantitative review. *The Botanical Review* 77: 462-570. https://doi.org/10.1007/s12229-011-9086-8

Magnin, J. (1740) Breve Descripción de la Provincia de Quito (1740). In: Bayle, C. (1940) Descubridores Jesuitas del Amazonas. Instituto Gonzalo Fernandez de Oviedo, Madrid.

Magnin, J. (1740/1940). Breve descripción de la Provincia de Quito y de sus misiones de Sucumbíos de religiosos de San Francisco y de Maynas de padres de la Compañía de Jesús a las orillas del gran río Marañón, hecha para el mapa que se hizo el año 1740. Revista del Instituto Gonzalo Fernández de Oviedo, 1, 151–185. Madrid.

Maia, A.C. et al. (2020) Fitoterapia familiar no assentamento Madre Cristina (Ariquemes, Rondônia). *Brazilian Journal of Development* 6(11): 89780-89798. https://doi.org/10.34117/bjdv6n11-406

Mansutti Rodríguez, A. (1986) Hierro, barro cocido, curare y cerbatanas: El comercio intra e interétnico entre los Uwotjuja. *Antropológica* 65: 3–75.

Marcgrave, G. & Piso, W. (1648) Historia naturalis Brasiliae: in qua non tantum plantæ et animalia, sed et indigenarum morbi, ingenia et mores describuntur et iconibus supra quingentas illustrantur. Elsevier, Amsterdam.

Marcoy, P. (1873) A journey across South America from the Pacific Ocean to the Atlantic Ocean (Vol. 1). Blackie and Son.

Marles, R.J., Neill, D.A. & Farnsworth, N.R. (1988) A contribution to the ethnopharmacology of the lowland Quichua people of Amazonian Ecuador. *Revista de la Academia Colombiana de Ciencias Exactas, Físicas y Naturales* 16: 111-120. https://www.accefyn.com/revista/Volumen_16/63/111-120.pdf

Maroni, P. (1738). Noticias auténticas del famoso río Marañón y misión apostólica de la Compañía de Jesús de la Provincia de Quito en los dilatados bosques de dicho río, escribíalas por los años de 1738 (J. P. Chaumeil, ed. 1988. Iquitos: IIAP-CETA.

Marques, W.P.G., dos Anjos, T.O., & da Costa, M.N.R.F. (2020) Plantas medicinais usadas por comunidades ribeirinhas do Estuário Amazônico. *Brazilian Journal of Development* 6(10): 74242-74261. https://doi.org/10.34117/bjdv6n10-013

Martin, F.W., Campbell, C.W. & Ruberte, R.M. (1987) Perennial Edible Fruits of the Tropics. Agriculture Handbook #642. USDA, Washington, D.C. https://assets-global.echocommunity.org/books/9678939f-cd82-4a10-b757-771c7fdbe13e/en/en_perennial-edible_print.pdf

Martins, J.E.C. (1989) Plantas Medicinais de Uso na Amazônia. 2a ediçao.

Martius, C.F.P. von (1830) Repertorium Pharmaceuticum, 36, 337.

Martius, C.F.P. von (1830) Ueber die Bereitung des Pfeilgiftes Urari bei den Indianern Juris am Rio Yupura in Nordbrasilien. *Buehner, Repert. Pharmacie* 36: 340-349. https://doi.org/10.5962/bhl.title.4399

Martius, C.F.P. von (1843) Beitrage zur Kenntniss der Gattung Erythroxylon. *Abhandlungen der mathematisch-physikalischen Classe der koniglich bayerischen Akademie der Wissenschaften, Munich* 3: 367-369. https://gallica.bnf.fr/ark:/12148/bpt6k97920m.image

Martius, C.F.P. von (1843) Systema Materiae Medicae Vegetabilis Brasiliensis. F. Fleischer. Leipzig. https://doi.org/10.5962/bhl.title.9541

Martius, C.F.P. von (1868) Flora Brasiliensis : Enumeratio plantarum in Brasilia hactenus detectarum (Vol. 6, Part 1, col. 295). München & Leipzig: F. Fleischer.

Martius, C.F.P. von & Spix, J.B. von (1823-31) Reise in Brasilien in den Jahren 1817 bis 1820. Volumes 3. M. Lindauer. Munich.

Martyn, E.B. & Follett Smith, R.R. (1936) The fish poison plants of British Guiana, with special reference to the genera *Tephrosia* and *Lonchocarpus*. *Agric. J. British Guiana* 7(3): 154-159.

Mata, N.D.S. (2009) Participação da mulher Waiãpi no uso tradicional de plantas medicinais. Dissertação Mestrado em Desenvolvimento Regional. Universidade Federal do Amapá, Macapá. https://www2.unifap.br/ppgdas/files/2022/10/DISSERTACAO-NELY-DAYSE-SANTOS-DA-MATA.pdf

Maugin De Lincourt, L. (1854) Fragments of travels from Itaituba to the cataracts of the Tapajos, and among the Mundrucus and Maues Indians. In: W.L. Herndon, ed. Exploration of the Valley of the Amazon, made under direction of the Navy Department. 2 vols. Washington: Robert Armstrong, vol. 1, pp. 314–315.

Maxwell, N. (1990) Witch Doctor's Apprentice: Hunting for Medicinal Plants in the Amazon. 3rd edition. Citadel Press, New York. 391 pp.

Mayer, W. (2006) The piassaba palm: conservation and development in the buffer zone of Peru's Cordillera Azul National Park. Nicholas School of the Environment and Earth Sciences, Duke University.

Mejía, K. (1988) Utilization of palms in eleven Mestizo villages of the Peruvian Amazon (Ucayali river, department of Loreto). *Advances in Economic Botany* 6: 130-136. https://www.jstor.org/stable/43927524

Mejía, K. (1992) Las palmeras en los mercados de Iquitos. *Bulletin de l´Institute Française d´Études Andines* 21: 755-769. https://www.persee.fr/doc/bifea_0303-7495_1992_num_21_2_1086

Mejía, K. & Rengifo, E. (2000) Plantas Medicinales de Uso Popular en la Amazonía Peruana. Agencia Española De Cooperación Internacional (AECI) Y El Instituto De Investigaciones De La Amazonía Peruana (IIAP), Lima. http://www.iiap.org.pe/upload/publicacion/l017.pdf

Mejía, K.M. (1983) Palmeras y el selvícola amazónico. Museo Historia Natural, Universidad Nacional Mayor de San Marcos, Lima.

Mejía, L.E. & Turbay, S. (2009) Los venenos de cacería en la Amazonia colombiana:¿sustancias letales o fuente de vitalidad? *Boletín de Antropología Universidad de Antioquia* 23(40): 129-153. https://www.redalyc.org/pdf/557/55715428007.pdf

Melo, N.C. (2015) Avaliação da atividade protetora solar in vitro das espécies pau-mulato (*Calycophyllum spruceanum* (Benth.) Hook. f. ex K. Schum) e ipê-amarelo (*Tabebuia aurea* (Silva Manso) Benth. & Hook. f. ex S. Moore). Dissertação Mestrado em Ciências da Saúde. Universidade Federal do Amapá, Macapá. http://repositorio.unifap.br:80/jspui/handle/123456789/128

Mendoza, D.E. & Panduro, A. (2005) El tejido de las hojas de palmera en la vivienda amazónica. Proyecto Araucaria Amazonas Nauta/ Agencia Española de Cooperación internacional - Gobierno Regional de Loreto - Iquitos, Perú.

Mendoza, P. (1994) Identificación de los frutos comestibles silvestres recolectados por los indígenas Huaorani de la comunidad de Toñiampari en la Amazonía del Ecuador. Pontificia Universidad Católica del Ecuador, Quito

Meneguelli, A.Z. (2020) Ethnopharmacological and botanical evaluation of medicinal plants used by Brazilian Amazon Indian community. *Interacoes* 31(3): 633-645. https://doi.org/10.20435/inter.v21i3.2926

Mesa, C.L. (2011) Etnobotánica de Palmas en la Amazonia Colombiana: Comunidades Indígenas Piapocos del río Guaviare, como estudio de caso. Master thesis. Línea Manejo y Conservación de Vida Silvestre. Universidad Nacional de Colombia, Facultad de Ciencias, Departamento de Biología. Bogotá. https://repositorio.unal.edu.co/handle/unal/8504

Mesa, L. & Galeano, G. (2013) Palms uses in the Colombian Amazon. *Caldasia* 35(2): 351-369. http://www.scielo.org.co/scielo.php?script=sci_arttext&pid=S0366-52322013000200011

Michelena y Rojas, F. (1867) Exploración oficial por la primera vez desde el norte de la América del Sur [...], en los años de 1855 hasta 1859. Bruselas: A. Lacroix, Verboeckhoven y Cía., impresores y editores.

Miller, C. (2002) Fruit production of the ungurahua palm (Oenocarpus bataua subsp. bataua, Arecaceae) in an indigenous managed reserve. *Economic Botany* 56: 165-176. https://www.jstor.org/stable/4256550

Miller, R.P., Wandelli, E.V. & Grenand, P. (1989) Conhecimento e utilização da floresta pelos índios Waimiri-Atroari do Rio Camanau-Amazonas. *Acta Botanica Brasilica* 3(2 suppl 1): 47-56. https://doi.org/10.1590/S0102-33061989000300005

Milliken, W. (2021) Traditional medicines amongst indigenous groups in Roraima, Brazil: A retrospective. *Ethnoscientia* 6(3): 116-139. https://periodicos.ufpa.br/index.php/ethnoscientia/article/view/10503

Milliken, W. et al. (2021) Plants used traditionally as antimalarials in Latin America: mining the Tree of Life for potential new medicines. *Journal of Ethnopharmacology* 279: 114221. https://doi.org/10.1016/j.jep.2021.114221

Milliken, W., Albert, B., Gomez, G.G. (1999) Yanomami: a forest people. Vol. 701. Royal Botanic Gardens, Kew.

Mitchell, J.D. (1992) Additions to *Anacardium* (Anacardiaceae). *Anacardium amapaënse*, a new species from French Guiana and eastern Amazonian Brazil. *Brittonia* 44(3): 331-338. https://www.jstor.org/stable/2806935

Mollinedo, L.G. (2000) Motacú (*Attalea phalerata*) en la Comunidad Leco Irimo. Publicaciones Proyecto de Investigación CIDOB-DFID, Santa Cruz.

Monachino, J. (1949) A Revision of *Ryania* (Flacourtiaceae). *Lloydia* 12(1): 1-29.

Monconill, G. M. (1945) Manera como preparan la coca los indios Uitoto en general. *Revista Amazonia* 1(9–10): 41–42.

Monconill, G. M. (1945) Preparación del tabaco entre los Witoto. *Revista Amazonia* 3: nos. 9-10.

Mondragón, M.L. & Smith, R. (1997) Bete Quiwiguimamo. Salvando el bosque para vivir sano. Ediciones Abya-Yala, Quito. https://digitalrepository.unm.edu/cgi/viewcontent.cgi?article=1544&context=abya_yala

Monteiro de Noronha, J. (1862) Roteiro da viagem da Cidade do Pará até as últimas colônias do sertão da Província, escrito na Villa de Barcelos pelo Vigário Geral do Rio Negro o Padre Dr. [...] no anno de 1768. Pará: Typographie de Santos & Irmãos.

Moore, S.J. et al. (2007) Field evaluation of traditionally used plant-based insect repellents and fumigants against the malaria vector *Anopheles darlingi* in Riberalta, Bolivian Amazon. *Journal of Medical Entomology* 44(4): 624-630. https://doi.org/10.1603/0022-2585(2007)44[624:feotup]2.0.co;2

Moraes, L.L.C. et al. (2019) A Ethno-knowledge of medicinal plants in a community in the eastern Amazon. *Revista de Ciências Agrárias* 42(2): 565-573. https://revistas.rcaap.pt/rca/article/download/15625/14181/

Moraes, M. (2004) Flora de Palmeras de Bolivia. Plural Editores, La Paz.

Moraes, M. & Sarmiento, J. (1999) La jatata (*Geonoma deversa* (Poit.) Kunth, Palmae)- un ejemplo de producto forestal forestal no maderable en Bolivia: uso tradicional en el este del departamento de La Paz. *Revista de la Sociedad Boliviana de Botánica* 2: 183-196.

Moraes, M. et al. (1996) Notes on the biology and uses of the Motacú palm (*Attalea phalerata*, Arecaceae) from Bolivia. *Economic Botany* 50: 423-428. https://doi.org/10.1007/BF02866525

Moraes, M., Sarmiento, J. & Oviedo, E. (1995) Richness and uses in a diverse palm site in Bolivia. *Biodiversity and Conservation* 4: 719-727. https://doi.org/10.1007/BF00158865

Morcote-Ríos, G. et al. (1998) Las palmas entre los grupos cazadores-recolectores de la Amazonia colombiana. *Cadalsia* 20: 57-74. https://revistas.unal.edu.co/index.php/cal/article/view/17470

Moreno Suárez, L. & Moreno Suárez, O.I. (2006) Colecciones de las palmeras de Bolivia. Editorial FAN, Santa Cruz de la Sierra.

Moretti, C. & Grenand, P. (1982) Les Nivrées ou plantes ichtyotoxiques de la Guyane française. *Journal of Ethnopharmacology* 6(2): 139-160. https://doi.org/10.1016/0378-8741(82)90002-2

Mori, S.A. (1979) In: G.T. Prance & S.A. Mori, Lecythidaceae. I. Fl. Neotrop. Monogr. 21: 128-197. New York Botanical Garden.

Mors, W.B. et al. (1973) Ichthyotoxic activity of plants of the genus *Derris* and compounds isolated therefrom. *Cienc. e Cult.* 25: 647-648.

Morton, C.V. (1931) Notes on Yage, a drug plant of southeastern Colombia. *Proceedings of the Academy of Sciences, Washington* 21(20): 487–488.

Muñoz, V. et al. (2000) A search for natural bioactive compounds in Bolivia through a multidisciplinary approach: Part I. Evaluation of the antimalarial activity of plants used by the Chacobo Indians. *Journal of Ethnopharmacology* 69(2): 127-137. https://doi.org/10.1016/s0378-8741(99)00148-8

Murdock, G.P. (1945) Nuestros contemporáneos primitivos. México: Fondo de Cultura Económica.

Naranjo, P. (1974) El cocaísmo entre los aborígenes de Sud América: su difusión y extinción en el Ecuador. *América Indígena* 34(3): 605-628.

Naranjo, P. (1981) Social function of coca in pre-Columbian America. *Journal of Ethnopharmacology* 3: 161–172.

Nascimento, E.S. (2011) Levantamento dos conhecimentos etnobotânicos de comunidades ribeirinhos do estuário amapaense. Trabalho de Conclusão de Curso (Graduação em Engenharia Florestal) - Universidade do Estado do Amapá, Macapá. http://www.iepa.ap.gov.br/biblioteca/tese/2023/RaullyanBorja.Pos.Dr.Relatorio-Final.pdf

Nimuendajú, C. (1952) The Tukuna. University of California Publications in American Archaeology and Ethnology, 45: 1–209.

Nimuendaju, C. (1956) Os Apinayé. *Boletim do Museu Paraense Emilio Goeldi* 12: 1-150. https://repositorio.museu-goeldi.br/handle/mgoeldi/865

Odonne, G. et al. (2011) Treatment of leishmaniasis in the Oyapock basin (French Guiana): A KAP survey and analysis of the evolution of phytotherapy knowledge amongst Wayãpi Indians. *Journal of Ethnopharmacology* 137(3): 1228-1239. https://doi.org/10.1016/j.jep.2011.07.044

Odonne, G. et al. (2013) Medical ethnobotany of the Chayahuita of the Paranapura basin (Peruvian Amazon). *Journal of Ethnopharmacology* 146(1): 127-153. https://doi.org/10.1016/j.jep.2012.12.014

Ojeda, P. (1994) Diagnóstico etnobotánico y comercialización del morete, Mauritia flexuosa (Arecaceae), en la zona del Alto Napo, Ecuador. Etnobotánica, valoración económica y comercialización de recursos florísticos silvestres en el Alto Napo, Ecuador. Ecociencia. Quito.

Oliveira, S.K.S. (2016) Etnobotânica em duas comunidades da Terra Indígena São Marcos. Roraima, Brasil (Doctoral dissertation, Tese (Doutorado em Biodiversidade e Conservação)-Instituto de Ciências Biológicas, Universidade Federal do Pará. Belém, 2016, 113 pp.).

Ordinaire, O. (1892) Du Pacifique a l'Atlantique par les Andes Peruviennes et l'Amazone. Plon, Nourrit & Co., Paris, Pp. 131-133.

Oré, I. & Llapapasca, D. (1996) Huertas domesticas como sistema tradicional de cultivo en Moena Caño, Rio Amazonas, Iquitos - Peru. *Folia Amazonica* 8(1): 91-110. http://www.iiap.org.pe/upload/publicacion/PUBL645.pdf

Ortiguera, T. de (1909) Jornada del río Marañón, con todo lo acaecido en ella y otras cosas notables dignas de ser sabidas, acaecidas en las Indias Occidentales. In M. Serrano & M. Sanz (Eds.), Historiadores de Indias (Tomo II, pp. 305–422). Madrid: Nueva Biblioteca de Autores Españoles.

Ortiz, R. (1994) Uso, conocimiento y manejo de lagunos recursos naturales en el mundo Yucuna (Mirití-Paraná, Amazonas, Colombia). Ediciones Abya-Yala, Quito. ISBN: 9978040846.

Ortiz, S.E. (1954) Estudios sobre lingüística aborigen de Colombia. Bogotá: Editorial Kelly / Ministerio de Educación Nacional, Revista Bolívar.

Otterburg, M. & Mamani, M. (2008) Buenas prácticas de aprovechamiento de Jatata. Tropico, La Paz. http://hdl.handle.net/20.500.12324/11671

Pacheco, T. et al. (1998) Evaluación de bosques secundarios de la zona de Iquitos. In: Kalliola, R. Geoecología y desarrollo Amazónico: estudio integrado en la zona de Iquitos, Perú. Annales Universitatis Turkuensis Ser. A II. http://www.iiap.org.pe/upload/publicacion/CDinvestigacion/unap/unap7/UNAP7_PORTADA.HTM

Padoch, C. (1988) Aguaje (*Mauritia flexuosa* L.f.) in the economy of Iquitos, Peru. *Advances in Economic Botany* 6: 214-224. https://www.jstor.org/stable/43927531

Padoch, C. & De Jong, W. (1989) Production and profit in agroforestry: an example from the Peruvian Amazon. In: Browder, J.O. Fragile Lands of Latin America. Strategies for Sustainable Development. Westview Press, London. https://www.taylorfrancis.com/chapters/edit/10.4324/9780429042805-7/production-profit-agroforestry-example-peruvian-amazon-christine-padoch-wil-de-jong

Padoch, C. et al. (1985) Amazonian agroforestry: a market-oriented system in Peru. *Agroforestry Systems* 3: 47-58. https://link.springer.com/article/10.1007/BF00045738

Padoch, C. et al. (1987) Market-oriented agroforestry at Tamshiyacu. *Advances in Economic Botany* 5: 90-96. https://www.jstor.org/stable/43919697

Paniagua Zambrana et al., unpubl., cited in: Paniagua-Zambrana, N.Y., Cámara-Leret, R., & Macía, M.J. (2015) Patterns of medicinal use of palms across northwestern South America. *The Botanical Review* 81: 317-415. https://doi.org/10.1007/s12229-015-9155-5

Paniagua Zambrana, N.Y. (1998) Estudio comparativo de la densidad y los niveles de producción de hojas, frutos y semilas en poblaciones naturales del *Attalea phalerata* (Palmae) sometidas a diferente intensidad de extracción (Riberalta, Depto. Beni, Bolivia). Universidad Mayor de San Andrés.

Paniagua Zambrana, N.Y. (2001) Guía de plantas útiles de la comunidad de San José de Uchupiamonas. FUND-ECO/LIDEMA/Herbario Nacional de Bolivia. La Paz

Paniagua Zambrana, N.Y. (2005) Diversidad, densidad, distribución y uso de las palmas en la región del Madidi, noreste del departamento de La Paz (Bolivia). *Ecología en Bolivia* 40: 265-280. https://www.mobot.org/MOBOT/Research/madidi/pdf/08Palmeras40-3.pdf

Paniagua-Zambrana, N.Y. (2005) Conocimiento y uso de las palmas del Abanico del río Pastaza en la Amazonía Peruana. Projekt Arbejde. Dept. of Systematic Botany, Institute of Biology, University of Aarhus, Aarhus.

Paniagua-Zambrana, N.Y., Cámara-Leret, R., & Macía, M.J. (2015) Patterns of medicinal use of palms across northwestern South America. *The Botanical Review* 81: 317-415. https://doi.org/10.1007/s12229-015-9155-5

Pardal, R. (ca. 1937). Medicina aborigen americana (Humanior. Biblioteca del americanista moderno, Sección C: Patrimonio cultural indiano, 3; dirigida por José Imbelloni). Buenos Aires: José Anesi.

Parrotta, J.A., Francis, J.K. & de Almeida, R.R. (1995) Trees of the Tapajós: a photographic field guide. https://doi.org/10.2737/IITF-GTR-1

Patiño, V.M. (1963) Plantas cultivadas y animales domésticos en América equinoccial II: Plantas alimenticias. Imprenta Departamental, Cali.

Patiño, V.M. (1963) Plantas cultivadas y animales domésticos en América equinoccial. I: Frutales. Imprenta Departamental, Cali.

Patiño, V.M. (1967) Plantas cultivadas y animales domésticos en América equinoccial III: Fibras, medicinas, misceláneas. Imprenta Departamental, Cali.

Patiño, V.M. (1990) Historia de la cultura material en la América equinoccial. Tomo 1: Alimentación y alimentos. Instituto Caro & Cuervo, Bogotá.

Patiño, V.M. (1990) Historia de la cultura material en la América equinoccial. Tomo 2: Vivienda y menaje. Instituto Caro & Cuervo, Bogotá.

Patiño, V.M. (1991) Historia de la cultura material en la América equinoccial. Tomo 3: Vías, transportes, comunicaciones. Instituto Caro & Cuervo, Bogotá.

Patiño, V.M. (1992) Historia de la cultura material en la América equinoccial. Tomo 4: Vestidos, adornos y vida social. Instituto Caro & Cuervo, Bogotá.

Patiño, V.M. (1992) Historia de la cultura material en la América equinoccial. Tomo 5: Teconología. Instituto Caro & Cuervo, Bogotá.

Patiño, V.M. (1993) Historia de la cultura material en la América equinoccial. Tomo 6: Comercio. Instituto Caro & Cuervo, Bogotá.

Peckolt, T. (1910) Heil- und Nutzpflanzen Brasiliens. *Berichfe der Deuischen Pharmazeutischen Geseiischaft* 20: 36-58, 43.

Pedrollo, C.T. et al. (2016) Medicinal plants at Rio Jauaperi, Brazilian Amazon: Ethnobotanical survey and environmental conservation. *Journal of Ethnopharmacology* 186: 111-124. https://doi.org/10.1016/j.jep.2016.03.055

Peña-Claros, M. (1996) Ecology and socioeconomics of palm heart extraction from wild populations of Euterpe precatoria Mar. in eastern Bolivia. University of Florida, Gainesville.

Pennington, T.D. (1990) Sapotaceae. Fl. Neotrop. Monogr. 52. New York Botanical Garden.

Peñuela Salazar, M.L. (2002) Estudio etnobotánico del género *Brosimum* sp. (Moraceae) su potencial de uso en Leticia y Araracuara (Amazonia colombiana). Trabajo de Grado. Departamento de Biología, Facultad de Ciencias, Universidad Nacional de Colombia, Bogotá. 155 pp.

Pereira, L.A. et al. (2007) Plantas medicinais de uma comunidade quilombola na Amazônia Oriental: aspectos utilitários de espécies das famílias Piperaceae e Solanaceae. *Rev. Bras. de Agroecologia* 2(2): 1385-1388. https://www.aba-agroecologia.org.br/revista/cad/article/view/2759

Pérez Arbeláez, E. (1956) Plantas útiles de Colombia: ensayo de botánica colombiana aplicada. Librería Colombiana, Bogotá.

Pérez, D. (2002) Etnobotánica medicinal y biocidas para malaria en la Región de Ucayali. *Folia Amazonica* 13(1-2): 87-108. http://www.iiap.org.pe/upload/publicacion/PUBL718.pdf

Pérez, F. (1862) Jeografía física del Estado del Cauca. Imprenta de la Nación, Bogotá.

Pérez, H., Bucheli, P.E. & Benavides, B.G. (2006) La agroforestería en Guainía: Una alternativa sostenible. Instituto amazónico de investigaciones científicas-Sinchi, Bogotá. https://www.sinchi.org.co/files/publicaciones/publicaciones/pdf/la%20agroforisteria%20en%20guainía%20una%20alternativa%20sosteniblewweb.pdf

Peters, C.M., Gentry, A.H. & Mendelsohn, R. (1989) Valuation of an Amazonian rainforest. *Nature* 339: 655-656. https://www.nature.com/articles/339655a0.pdf

Pezzuti, J. & Chaves, R.P. (2009) Etnografia e manejo de recursos naturais pelos índios Deni, Amazonas, Brasil. *Acta Amazonica* 39: 121-138. https://doi.org/10.1590/S0044-59672009000100013

Phillips, O.L. (1993) The potential for harvesting fruits in tropical rainforests: new data from Amazonian Peru. *Biodiversity and Conservation* 2: 18-38. https://doi.org/10.1007/BF00055100

Pilnik, M.S. et al. (2023) Traditional botanical knowledge: food plants from the Huni Kui indigenous people, Acre, western Brazilian Amazon. *Rodriguésia* 74: e00482021. https://doi.org/10.1590/2175-7860202374016

Pinedo-Vasquez, M. et al. (1990) Use-Values of tree species in a communal forest reserve in northeast Peru. *Conservation Biology* 4: 405-416. https://www.jstor.org/stable/2385934

Pinkley, H.V. (1969) Plant admixtures to ayahuasca, the South American hallucinogenic drink. *Lloydia* 32(3): 305-314.

Pinkley, H.V. (1973) The ethno-ecology of the Kofan Indians (Doctoral dissertation, Harvard University).

Pinto, A.A.C. & Maduro, C.B. (2003) Produtos e subprodutos da medicina popular comercializados na cidade de Boa Vista, Roraima. *Acta Amazonica* 33: 281-290. https://doi.org/10.1590/1809-4392200332290

Pipoly, J.J. (1983) Contributions toward a monograph of Cybianthus (Myrsinaceae): III. A revision of subgenus Laxiflorus. *Brittonia* 35: 61-80. https://doi.org/10.2307/2806053

Pires, J.M. (1978) Plantas ictiotoxicas: Aspecto da botânica systematica. *Ciencia e Cult.* 32: 37-41.

Piso, W. (1648) História natural do Brasil. Imprensa Oficial do Estado, São Paulo.

Piso, W. (1658) De India Utriusque Re Naturali et Medica. Elsevier, Amsterdam.

Pittier, H. (1926) Manual de las Plantas Usuales de Venezuela. Litografia del comercio. https://dn720305.ca.archive.org/0/items/manualdelasplant00pitt/manualdelasplant00pitt.pdf

Planchon, G. (1880) Études sur les Strychnos. VII. Nouvelles notes sur les Strychnos qui fournissent le curare de l'Orénoque. *Journal de Pharmacie et de Chimie* 5e série (1): 380–384, 488–493.

Plotkin, M.J. (1994) Tales of a Shaman's Apprentice: An Ethnobotanist Searches for New Medicines in the Rain Forest. Viking Press, NY. 318 pp.

Plowman, T. (1977) *Brunfelsia* in ethnomedicine. *Botanical Museum Leaflets* 25(10): 289-320. https://www.jstor.org/stable/41762775

Plowman, T. (1980) Chamairo: *Mussatia hyacinthina* - an admixture to coca from Amazonian Peru and Bolivia. *Botanical Museum Leaflets* 28(3): 253-261. https://www.jstor.org/stable/41762833

Plowman, T. (1981) Amazonian coca. *Journal of Ethnopharmacology* 3: 195-225. https://doi.org/10.1016/0378-8741(81)90054-4

Plowman, T. et al. (1990) Significance of the fungus *Balansia cyperi* infecting medicinal species of Cyperus (Cyperaceae) from Amazonia. *Economic Botany* 44: 452-462. https://doi.org/10.1007/BF02859780

Polesna, L. et al. (2011) Ethnopharmacological inventory of plants used in coronel Portillo province of Ucayali department, Peru. *Pharmaceutical Biology* 49(2): 125-136. https://doi.org/10.3109/13880209.2010.504927

Ponce, M. (1992) Etnobotánica de palmas de Jatun Sacha. Pp. 43-51. In: Anonymous (ed.) Memorias del Tercer Simposio Colombiano de Etnobotánica. INCIVA.

Posey, D.A. (1984) A preliminary report on diversified management of tropical forest by the Kayapo Indians of the Brazilian Amazon. *Advances in Economic Botany* 1: 112-126. https://www.jstor.org/stable/43931371

Posey, D.A. (2002) Kayapó Ethnoecology and Culture (Vol. 6). Routledge. https://doi.org/10.4324/9780203220191

Poveda, L.J. (1985-86) Marvels of our Medicinal Flora (in Spanish). *Biocenosis* Vols. 1 and 2.

Prada Pedreros, S. (1987) Acercamiento etnopiscicolas con los indios Ticunas del Parque Nacional Natural Amacayacu, Amazonas (Colombia). Tesis de pregrado, Facultad de Ciencias, Universidad Nacional de Colombia, Bogotá.

Prado, M.L. (2008) Las palmas en la comunidad Tikuna de San Martín de Amacayacu: Conocimiento y Uso. Tesis de Maestría en Estudios Amazónicos. Universidad Nacional de Colombia. Sede Amazonia. Leticia, Colombia.

Prance, G.T. (1972) An ethnobotanical comparison of four tribes of Amazonian Indians. *Acta Amazonica* 2(2): 7-27. https://doi.org/10.1590/1809-43921972022007

Prance, G.T. (1972) Chrysobalanaceae (Flora Neotropica, Monograph No. 9). Hafner Publishing Co., New York.

Prance, G.T. (1972) Ethnobotanical notes from Amazonian Brazil. *Economic Botany* 26(3): 221-237. https://www.jstor.org/stable/4253351

Prance, G.T. (1978) The poisons and narcotics of the Dení, Paumarí, Jamamadí and Jarawara Indians of the Purus River Region. *Revista Brasileira de Botânica* 1: 71–82.

Prance, G.T., Campbell, D.G. & Nelson, B.W. (1977) The ethnobotany of the Paumarí Indians. *Economic Botany* 31(2): 129-139. https://www.jstor.org/stable/4253820

Proctor, P. et al. (1992) Expedición de la Universidad de Oxford a Bolivia. Investigación etnobotánica de las Palmae en el noroeste del departamento de Pando. 25 junio-7 de septiembre de 1992. Informe final: Sección B. Universidad de Oxford.

Pupiales, M. (1945) La coca entre la tribu Uitoto de Tarapacá y Piuña Negro. *Revista Amazonia Americanista* 2 (9-10).

Quintana Arias, R.F. (2012) Estudio de plantas medicinales usadas en la comunidad indígena Tikuna del alto Amazonas, Macedonia. *Nova* 10(18): 181-193. http://www.scielo.org.co/scielo.php?pid=S1794-24702012000200005&script=sci_abstract&tlng=es

Quintana, G. & Vargas, L. (1995) Guia popular de plantas utilizadas por los Mosetenes de Covendo, Santa Ana y Muchanes (Alto Beni, Bolivia). FONAMA, La Paz.

Ramos, R.S. (2014) Estudo fitoquímico da atividade microbiológica de citoxicidade e larvicida dos óleos essenciais de espécies da família Lamiacea (LAMIALES). Dissertação Mestrado em Ciências Farmacêuticas. Universidade Federal do Amapá, Macapá.

Ramos, R.S. et al. (2015) Estudo físico-químico e avaliação do potencial larvicida do extrato etanólico das cascas do caule de *Licania macrophylla* Benth. *Biota Amazonia* 5(1): 74-78. http://dx.doi.org/10.18561/2179-5746/biotaamazonia.v5n1p74-78

Reichel-Dolmatoff, G. (1944) La cultura de los Indios Gauhibo. *Rev. Inst. Etnol. Nal.*: 435-454.

Reichel-Dolmatoff, G. (1968) Amazonian Cosmos: The Sexual and Religious Symbolism of the Tukano Indians. Chicago & London: University of Chicago Press, pp. 15f., 27–28, 36f., 43, 45, 77, 82, 126, 127, 267.

Reichel-Dolmatoff, G. (1970) Notes on the cultural extent of the use of Yaje (*Banisteriopsis caapi*) among the Indians of the Vaupés, Colombia. *Economic Botany* 24: 33.

Reinburg, P. (1921) Contribution à l'étude des boissons toxiques des Indiens du nord-ouest de l'Amazone: L'ayahuasca – Le yagé – Le huánto. Étude comparative toxico-physiologique d'une expérience personnelle. *Journal de la Société des Américanistes de Paris* 13: 25–54, 197–216.

Remy, F.E. (1908) Apuntes sobre el Clima y Flora de la Region del Pichis. Pp. 328-351. In: Carlos Larrabure y Correa (Eds.) Coleccion de Leyes, Decretos y Resoluciones Lima, Perú.

Rengifo-Salgado, E. et al. (2017) Saberes ancestrales sobre el uso de flora y fauna en la comunidad indígena Tikuna de Cushillo Cocha, zona fronteriza Perú-Colombia-Brasil. *Revista Peruana de Biología* 24(1): 67-78. http://dx.doi.org/10.15381/rpb.v24i1.13108

Renner, S.S., Balslev, H. & Holm-Nielsen, L.B. (1990) Flowering Plants of Amazonian Ecuador: A Checklist. AAU Reports #24, Botanical Institute, University of Aarhus, Denmark.

Revilla, J. (2002) Plantas Uteis da Bacia Amazonica. Manaus. SEBRAE, INPA. 2 Vols.

Reyes-Garcia, V.E. (2001) Indigenous people, ethnobotanical knowledge, and market economy: a case study of the Tsimane Amerindians in lowland Bolivia. University of Florida.

Reyes, C. (2017) Conocimiento y uso de plantas en tres comunidades Kichwas: Yana Yaku, Loro Cachi y Nina Amarun, Pastaza-Ecuador. *Cinchonia* 15(1): 164-256.

Ribeiro Magno-Silva, E., Teixeira Rocha, T. & Caldeira Tavares-Martins, A.C. (2020) Ethnobotany and ethnopharmacology of medicinal plants used in communities of the Soure Marine Extractive Reserve, Pará State, Brazil. *Boletín Latinoamericano y del Caribe de Plantas Medicinales y Aromáticas* 19(1): 29-64. https://doi.org/10.37360/blacpma.20.19.1.3

Ribeiro, R.V. et al. (2017) Ethnobotanical study of medicinal plants used by Ribeirinhos in the North Araguaia microregion, Mato Grosso, Brazil. *Journal of Ethnopharmacology* 205: 69-102. https://doi.org/10.1016/j.jep.2017.04.023

Rice, H. (1910) The River Uaupés. *Geographical Journal* 35(6): 682-700.

Rios, M. & Caballero, J. (1997) Las plantas en la alimentación de la comunidad Ahuano, Amazonía ecuatoriana. Uso y manejo de recursos vegetales-Memorias del segundo simposio ecuatoriano de etnobotánica y botánica económica. Ediciones Abya-Yala, Quito.

Ritter, R.A. et al. (2012) Ethnoveterinary knowledge and practices at Colares island, Pará state, eastern Amazon, Brazil. *Journal of Ethnopharmacology* 144: 346-352. https://doi.org/10.1016/j.jep.2012.09.018

Rizzini, G.T. & Mors, W. (1976) Botânica Econômica Brasileira. São Paulo, EPU, Ed. da Universidade do São Paulo. ISBN: 978-8586742187.

Robineau, L. Ed. (1991) Towards a Caribbean Pharmacopoeia. TRAMIL-4 WORKSHOP, UNAH, Enda Caribe, Santo Domingo.

Rocha, J. (1905) Memorándum de viaje (Regiones amazónicas). Bogotá: Casa Editorial El Mercurio.

Rodrigues de Freitas, R., Haverroth, M. & Siviero, A. (2016) Educação agroflorestal para resiliência socioecológica de Reservas Extrativistas da Amazônia. In: Siviero, A. Chau Ming, L., Silveira, M., Daly, D. & Wallace, R. (eds.) Etnobotânica e Botânica Econômica do Acre. Editora da Universidade Federal do Acre-Edufac. ISBN: 978-85-8236-027-9.

Rodrigues, E. (2006) Plants and animals utilized as medicines in the Jaú National Park (JNP), Brazilian Amazon. *Phytotherapy Research* 20(5): 378-391. https://doi.org/10.1002/ptr.1866

Rodrigues, E., Duarte-Almeida, J.M. & Pires, J.M. (2010) Perfil farmacológico e fitoquímico de plantas indicadas pelos caboclos do Parque Nacional do Jaú (AM) como potenciais analgésicas: parte I. *Revista Brasileira de Farmacognosia* 20: 981-991. https://doi.org/10.1590/S0102-695X2010005000008

Rodrigues, E., Mendes, F.R. & Negri, G. (2006) Plants indicated by brazilian indians for disturbances of the central nervous system: A bibliographical survey. *Central Nervous System Agents in Medicinal Chemistry* 6(3): 211-244. https://doi.org/10.2174/187152406778226725

Rodrigues, I. & Oliveira, A.E.D. (1977) Alguns aspectos da ergologia Mura-Pirahã. *Boletim do Museu Paraense Emilio Goeldi* 65: 1-54. http://repositorio.museu-goeldi.br/handle/mgoeldi/437

Rodrigues, S., Caetano N., D.G. & Caetano, C.M. (2007) Espécies frutíferas do centro-sul do Estado de Rondônia, Amazônia brasileira. *Acta Agronómica* 56(2): 69-74. http://www.scielo.org.co/scielo.php?script=sci_arttext&pid=S0120-28122007000200003

Rodriquez, F. (1996) Waorani hunting and harvesting practices in Ecuador. CTFS/STRI.

Rojas, R. et al. (2001) Comercialización de masa y fruto verde de aguaje (*Mauritia flexuosa* L.F.) en Iquitos (Perú). *Folia Amazonica* 12: 15-38. https://doi.org/10.24841/fa.v12i1-2.123

Román, F.J. (2002) Especies forestales utilizadas en la construcción de la vivienda tradicional Asháninka en el ámbito del Río Perené (Junín, Perú). Facultad de Ciencias Forestales.

Universidad Nacional Agraria La Molina, Lima.

Romanoff, S. et al. (2004) La Vida Tradicional de los Matsés. CAAAP, Lima.

Roth, W.E. (1924) An introductory study of the arts, crafts and customs of the Guiana Indians. In: Thirty-eighth Annual Report of the Bureau of American Ethnology, 1916-1917: 25-745. https://repository.si.edu/handle/10088/91764

Ruiz, H. (1952) Relación histórica del viage que hizo a los reynos del Perú y Chile el botánico don Hipólito Ruiz en el año de 1777 hasta el de 1788, en cuya época regresó a Madrid (Tomo I, texto). Madrid: Real Academia de Ciencias Exactas, Físicas y Naturales / Talleres Gráficos de Cándido Bermejo. XLIV + 526 págs.

Ruiz, L. et al. (2011) Plants used by native Amazonian groups from the Nanay River (Peru) for the treatment of malaria. *Journal of Ethnopharmacology* 133(2): 917-921. https://doi.org/10.1016/j.jep.2010.10.039

Rusby, H.H. (1924) Miré. *Journal of the American Pharmaceutical Association* 13: 101-102.

Rutter, R.A. (1990) Catalogo de Plantas Útiles de la Amazonia Peruana. Comunidades y Culturas Peruanas No. 22. Ministerio de Edudación, Instituo Linguistico de Verano. http://repositorio.cultura.gob.pe/handle/CULTURA/645

Safford, W.E. (1916) Identity of cohoba, the narcotic snuff of ancient Haiti. *Journal of the Washington Academy of Sciences* 6(15): 547-562. https://www.jstor.org/stable/24521298

Storey, C. & Salem, J.I. (1997) Lay use of Amazonian plants for the treatment of tuberculosis. *Acta Amazónica* 27(3): 175-182.

Saltos, R.V.A. et al. (2016) The use of medicinal plants by rural populations of the Pastaza province in the Ecuadorian Amazon. *Acta Amazonica* 46: 355-366. https://doi.org/10.1590/1809-4392201600305

Sampaio, A.J. de (1916) A Flora de Matto Grosso. Archivos do Museu Nacional do Rio de Janeiro 19: 1-125.

Sampaio, F.R. de (1774) Diario da Viagem ... da Capitania da Rio Negro no Anno de (1774-1775) Typografia da Academia. Lisboa. (1825).

San Sebastián, M. (1995) Ñucanchic Janpi: Tratamientos con plantas medicinales de los Naporunas. CICAME- SANDI YURA, Coca.

Sánchez, M. (1997) Catálogo Preliminar Comentado de la Flora del Medio Caquetá. Estudios en la Amazonia colombiana, 12.

Sánchez, M. (2005) Use of tropical rain forest biodiversity by indigenous communities in northwestern Amazonia. PhD Thesis. Universiteit van Amsterdam/COLCIENCIAS, Bogotá.

Sánchez, M. & Miraña, P. (1991) Utilización de la vegetación arbórea en el Medio Caquetá: 1. El árbol dentro de las unidades de la tierra, un recurso para la comunidad Miraña. *Colombia Amazonica* 5: 69-98.

Santos, M., Pantoja, T.R. & Oliveira, A.M. (2022) Uso popular de plantas medicinais no tratamento do diabetes mellitus no Estado do Amapá, Brasil. Resumos XXVI SPMB. https://resumos.sbpmed.org.br/index.php/spmb/article/view/27

Santos, M.R.A., Lima, M.R. & Oliveira, C.L.L.G. (2014) Medicinal plants used in Rondônia, Western amazon, Brazil. *Revista Brasileira de Plantas Medicinais* 16(3): 707-720. https://doi.org/10.1590/1983-084x/13_102

Sanz-Biset, J. et al. (2009) A first survey on the medicinal plants of the Chazuta valley (Peruvian Amazon). *Journal of Ethnopharmacology* 122(2): 333-362. https://doi.org/10.1016/j.jep.2008.12.009

Schomburgk, R. (1847) Reisen in Britisch Guiana 1840-1844. Vol. 1. Lepizig.

Schomburgk, R. (1922–1923). Richard Schomburgk's travels in British Guiana, 1840–1844 (Vol. 2). Georgetown: "Daily Chronicle" Office.

Schultes, R.E. (1951) Plantae Austro-Americanae VII. *Botanical Museum Leaflets* 15(2): 29-78. https://doi.org/10.5962/p.168473

Schultes, R.E. (1955) A new generic concept in the Euphorbiaccae. *Botanical Museum Leaflets* 17(1): 27-36. https://www.jstor.org/stable/41762939

Schultes, R.E. (1955) Pitch-yielding trees of the Colombian Amazonia. *Botanical Museum Leaflets* 17(1): 12-24.

Schultes, R.E. (1956) The Amazon Indian and evolution in Hevea and related genera. *Journal of the Arnold Arboretum 37*(2): 123-152. https://www.jstor.org/stable/43790885

Schultes, R.E. (1974) Palms and religion in the Northwest Amazon. *Principes* 18: 3-21.

Schultes, R.E. (1976) Plantae Colombianae XIX. E partibus amazonicis Witotorum plantae fructuariae sativae novae. *Botanical Museum Leaflets* 24(8): 193-204. https://www.jstor.org/stable/41762766

Schultes, R.E. (1977) Diversas plantas comestíveis nativas do noroeste da Amazônia. *Acta Amazonica* 7: 317-327. https://doi.org/10.1590/1809-43921977073317

Schultes, R.E. (1978) Evolution of the identification of the major South American narcotic plants. *Botanical Museum Leaflets* 26(9-10): 311-337. https://www.biodiversitylibrary.org/page/7466330#page/327/mode/1up

Schultes, R.E. & Raffauf, R.F. (1990) Field notes on curare constituents in the Northwest Amazonia. *Curare* 13(2): 105-120. https://www.digi-hub.de/viewer/fullscreen/1688641822626/113/LOG_0053/

Schultes, R.E. & Raffauf, R.F. (1990) The Healing Forest: Medicinal and Toxic Plants of the Northwest Amazonia (Dioscorides Press, Portland, OR).

Schultes, R.E. & Raffauf, R.F. (1992) A rare report of an intoxicating snuff from the Amazon. *Kew Bulletin* 47(4): 743-744. https://doi.org/10.2307/4110719

Schultz, H. (1959) Ligeiras notas sobre os Makú do Paraná Boá-Boá. *Revista do Museu Paulista, Nova Serie* 11: 109-132.

Schwacke, W. (1884) Bereitung des Curare-Pfeilgiftes bei den Tecuna-Indianern. *Jahrb. Bot. Gart. Berlin 3*: 220-223. https://www.zobodat.at/pdf/Jb-kgl-bot-Garten-bot-Mus-Berlin_3_0220-0223.pdf

Seitz, G.J. (1967) 'Epeéna, the intoxicating snuff powder of the Waika Indians and the Tukano medicine man, Agostino', in Efron, D.H. (ed.) Ethnopharmacologic search for psychoactive drugs. Washington, D.C.: U.S. Government Printing Office (Public Health Service Publication no. 1645), p. 334.

Seoane, E. & Soplín, S. (1999) Plantas medicinales utilizadas en la regulación de la fertilidad. *Biota* 17: 82-99.

Serra, J. de Santa Gertrudis (Fray)(1757-1767/1956) Maravillas de la naturaleza (Vol. 1). Editorial Argra, Bogotá.

Shepard, G.H. & van der Veld, P. (2004) Arte Baniwa: Sustentabilidade socioambiental de aruma no Alto Rio Negro. Pp. 129-143. In: Ricardo, F. (ed.) Terras indígenas e unidades de conservacao danatureza: O desafio das sobreposicoes. Instituto Socioambiental, Sao Paulo.

Shepard, G.H. et al. (2001) Rain forest habitat classification among the Matsigenka of the Peruvian Amazon. *Journal of Ethnobiology* 21: 1-38. https://ethnobiology.org/sites/default/files/pdfs/JoE/21-1/Shepard-etal.pdf

Shrestha, T., Kopp, B. & Bisset, N.G. (1992) The Moraceae-based dart poisons of South America. Cardiac glycosides of *Maquira* and *Naucleopsis* species. *Journal of Ethnopharmacology* 37(2): 129-143. https://doi.org/10.1016/0378-8741(92)90071-x

Silva Castro, F. da (1868) *Gazeta Medica de Bahia* (39 & 46).

Silva Farias, M. et al. (2016) Diversidade e uso de palmeiras da mata ciliar do rio Acre. Chapter 7. In: Siviero, A. Chau Ming, L., Silveira, M., Daly, D. & Wallace, R. (eds.) Etnobotânica e Botânica Econômica do Acre. Editora da Universidade Federal do Acre-Edufac. ISBN: 978-85-8236-027-9.

Silva, H. & García, J. (1997) La Medicina Tradicional en Loreto. Instituto Peruano de Seguridad Social / Instituto de Medicina Tradicional, Iquitos.

Silva, R.B.L. (2002) A etnobotânica de plantas medicinais da comunidade quilombola de Curiaú, Macapá-AP, Brasil. Dissertação Mestrado em Agronomia. Departamento de Biologia Vegetal, Faculdade de Ciências Agrárias do Pará, Belém.

Silva, R.B.L. (2010) Diversidade, uso e manejo de quintais agroflorestais no Distrito do Carvão, Mazagão-AP, Brasil. Tese Doutorado em Desenvolvimento Sustentável do Trópico Úmido-Universidade Federal do Pará/Núcleo de Altos Estudos Amazônicos, Belém. https://repositorio.ufpa.br/jspui/handle/2011/11107

Silva, R.B.L. et al. (2013) Caracterização agroecológica e socioeconômica dos moradores da comunidade quilombola do Curiaú, Macapá-AP, Brasil. *Biota Amazônia* 3(3): 113-138. http://www.iepa.ap.gov.br/biblioteca/artigo/2015/caracterizacao-agroecol-sociocon-quilombola-curiau.pdf

Silva, W. et al. (2019) Guia etnobotânico de plantas em comunidades Desano (Tukano-oriental) no rio Tiquié - Brasil. *Cadernos de Etnolingüística* 7(1): 1-42. http://www.etnolinguistica.org/article:vol7n1p1-42

Silverwood-Cope, P.L. (1990). Os Makú: povo caçador do noroeste da Amazônia.

Simón, P. (1627) Noticias historiales de las conquistas de Tierra Firme en las Indias Occidentales. Editorial Kelly (1953), Bogotá.

Simson, A. (1886) Travels in the Wilds of Ecuador and Exploration of the Putumayo River.

Skov, F. & Balslev, H. (1989) A revision of *Hyospathe* (Arecaceae). *Nordic Journal of Botany* 9: 189-202. https://doi.org/10.1111/j.1756-1051.1989.tb02114.x

Smith, A.C. (1939) Notes on a collection of plants from British Guiana. *Lloydia* 2: 161-218.

Smith, N. (1979) A Pesca no Rio Amazonas. INPA, Manaus.

Smith, N., Vásquez, R. & Wust, W.H. (2007) Amazon River Fruits. Flavors for Conservation. Amazon Conservation Association (ACA)/ Missouri Botanical Garden Press, Lima.

Soares Machado, F. (2016) Etnobotânica de espécies florestais não madeireiras em comunidades locais do Vale do Juruá, Acre. Chapter 2. In: Siviero, A. Chau Ming, L.,

Silveira, M., Daly, D. & Wallace, R. (eds.) Etnobotânica e Botânica Econômica do Acre. Editora da Universidade Federal do Acre-Edufac. ISBN: 978-85-8236-027-9.

Soriano Lleras, A. (1968). Itinerario de la Comisión Corográfica y otros escritos. Bogotá: Imprenta Nacional, Universidad Nacional de Colombia

Sosnowska, J, Ramírez, D. & Millán, B. (2010) Palmeras usadas por los indígenas Asháninkas en la Amazonía Peruana. *Revista Peruana de Biología* 17(3): 347-352. http://www.scielo.org.pe/scielo.php?script=sci_arttext&pid=S1727-99332010000300009

Soukup, J. (1970) Vocabulary of the Common Names of the Peruvian Flora and Catalog of the Genera. Editorial Salesiano, Lima.

Sousa, G.S. de (1938) Tratado descritivo do Brasil em 1587 (3.ª ed.). São Paulo: Empresa Gráfica da “Revista dos Tribunais”

Southey, R. (1819) History of Brazil 3: 722-723.

Souto, R.N.P.S. et al. (2011) Estudos preliminares da atividade inseticida de óleos essenciais de espécies de *Piper linneus* (Piperaceae) em operárias de *Solenopis saevissima* f Smith (Hymenoptera: formicidae), em laboratório. *Biota Amazonia* 1(1): 42-48. http://dx.doi.org/10.18561/2179-5746/biotaamazonia.v1n1p42-48

Souza, B. (1956) O cipó-babão (*Cissus gongylodes* Baker). Um agente coagulante do látex de *Hevea. Bol. Técnico do Instituto Agronômico do Norte* (31): 163-186. http://www.alice.cnptia.embrapa.br/alice/handle/doc/375650

Spruce, R. (1853) Notes of a Botanist on the Amazon and Andes. Vol. 1. (A.R. Wallace, Ed. 1908). Macmillan.

Spruce, R. (1853) Notes of a Botanist on the Amazon and Andes, Vol. 2. (A.R. Wallace, Ed. 1908). Macmillan.

Spruce, R. (1854) MSS I Uses of American Plants pp. 31-End & C Journal 1854, no. 5 January 4 to June 18, pp. 128-156 (renumbered 106-130), section 190 pp. 136-136* (renumbered 113-114), Archives, Royal Botanic Gardens, Kew.

Spruce, R. (1873) On some remarkable narcotics of the Amazon Valley and Orinoco. *Geographical Review* 5(1): 184-193.

Spruce, R. (1873) Personal experiences on venomous reptiles and insects in South America. *Geographical Review* 4(1): 135-146.

Stagegaard, J., Sørensen, M. & Kvist, L.P. (2002) Estimations of the importance of plant resources extracted by inhabitants of the Peruvian Amazon flood plains. *Perspectives in Plant Ecology, Evolution and Systematics* 5: 103-122. https://doi.org/10.1078/1433-8319-00026

Steward, J.H. (Ed.)(1950). Handbook of South American Indians: Volume 6, Physical anthropology, linguistics and cultural geography of South American Indians. Washington, DC: Government Printing Office.

Steward, J.H. & Metraux, A. (1948) Tribes of the Peruvian and Ecuadorian Montaña. In: Steward, H.J. (ed.) Handbook of South American Indians. Vol 3: The Tropical Forest Tribes. Bureau of American Ethnology. Bulletin 143, Washington DC. p. 594, 605.

Steyemark, J.A. (1984) Flora de Venezuela. Piperaceae. Ediciones Fundacion Ambiental, Venezuela.

Steyermark, J. (1964) Herbarium specimen. Steyermark 60'758a

Svenning, J.C. & Macía, M.J. (2002) Harvesting of *Geonoma macrostachys* Mart. leaves for thatch: an exploration of sustainability. *Forest Ecology and Management* 167: 251-262. https://doi.org/10.1016/S0378-1127(01)00699-5

Tessmann, G. (1930) Die Indianer Nordost-Perus: grundlegende Forschungen für eine systematische Kulturkunde. Veröffentlichung der Harvey-Bassler-Stiftung, Bd. 2. Hamburg: Friederichsen, De Gruyter & Co.

Thevet, A. (1557) Les singularites de la France antarctique, autrement nommee Amerique; et Isles decouvertes de nostre temps. Nouvelle edition avec notes et commentaires par Paul Gaffarel. Paris. (Paris ed. 1557; Anvers ed. 1558).

Thomas, E. (2008) Quantitative ethnobotanical research on knowledge and use of plants for livelihood among Quechua, Yuracaré and Trinitario communities in the Andes and Amazon regions of Bolivia. Faculty of Bioscience Engineering. Ghent University, Belgium.

Thomas, E. & Vandebroek, I. (2006) Guía de Plantas Medicinales de los Yuracarés y Trinitarios del Territorio Indígena Parque Nacional Isiboro-Sécure, Bolivia. Industrias gráficas Sirena, Santa Cruz.

Ticona, J.P. (2001) Los chimane: conocimiento y uso de plantas medicinales en la comunidad Tacuaral del Matos (Provincia Ballivián, Departamento del Beni). Facultad de Ciencias Puras y Naturales. Universidad Mayor de San Andres, La Paz.

Tomchinsky, B. (2014) Etnobotânica de plantas antimaláricas em Barcelos, Amazonas. Dissertação Mestrado Faculdade de Ciências Agronômica. Universidade Estadual Paulista. https://repositorio.unesp.br/entities/publication/1d702d17-8e0d-413d-9597-174737f4fa2a

Tomchinsky, B. et al. (2017) Ethnobotanical study of antimalarial plants in the middle region of the Negro River, Amazonas, Brazil. *Acta Amazonica* 47(3): 203-212. https://doi.org/10.1590/1809-4392201701191

Tournon, J. (2006) Las Plantas, los Rao y sus espíritus (Etnobotánica del Ucayali). Gobierno Regional de Ucayali, Pucallpa.

Tournon, J., Raynal-Roques, A. & Zambettakis, C. (1986) Les Cyperacees medicinales et magiques de L'Ucayali. *Journal d'Agriculture Traditionnelle et de Botanique Appliquée* 33(1): 213-224. https://www.persee.fr/doc/jatba_0183-5173_1986_num_33_1_3952

Tovar, E. D. (1966) Vocabulario del oriente peruano (M. Hildebrandt, Prólogo). Lima: Universidad Mayor de San Marcos, Imprenta Universidad Mayor de San Marcos.

Townsend, W.R. (1996) Nyao Itõ: caza y pesca de los Sirionó. Instituto de Ecología, Universidad Mayor de San Andrés, La Paz.

Triana, G. (1985) Los Puinaves del Inirida. Formas de subsistencia y mecanismos de adaptación. Instituto de Ciencias Naturales-Museo de Historia Natural. Universidad Nacional de Colombia, Bogotá.

Trujillo, W. & Correa-Munera, M. (2010) Plants used by a Coreguaje indigenous community in the Colombian Amazon. *Caldasia* 32(1): 1-20. http://www.scielo.org.co/scielo.php?pid=S0366-52322010000100001&script=sci_abstract

Tudela-Talavera, P. & La Torre, M.D.L.A. (2015) Cultural importance and use of medicinal plants in the Shipibo-Conibo native community of Vencedor (Loreto) Peru. *Ethnobotany Research and Applications* 14: 533-548. https://ethnobotanyjournal.org/index.php/era/article/view/1101

Uscategui M., N. (1954) Contribución al estudio de la masticación de las hojas de coca. *Revista Colombiana de Antropología* 3: 209-289.

Uscategui M., N. (1954) El tabaco entre las tribus indígenas de Colombia. *Revista Colombiana de Antropología* 5: 12, 52.

Uscategui M., N. (1959) The present distribution of narcotics and stimulants amongst the Indian tribes of Colombia. *Botanical Museum Leaflets* 18(6): 273-304.

Uscategui M., N. (1961) Algunos colorantes vegetales usados por las tribus indígenas de Colombia. *Revista Colombiana de Antropología* 10: 333-340. https://doi.org/10.22380/2539472X.1645

Valadeau, C. et al. (2009) Medicinal plants from the Yanesha (Peru): Evaluation of the leishmanicidal and antimalarial activity of selected extracts. *Journal of Ethnopharmacology* 123: 413-422. https://doi.org/10.1016/j.jep.2009.03.041

Valadeau, C. et al. (2010) The rainbow hurts my skin: Medicinal concepts and plants uses among the Yanesha (Amuesha), an Amazonian Peruvian ethnic group. *Journal of Ethnopharmacology* 127: 175-192. https://doi.org/10.1016/j.jep.2009.09.024

Valdizán, H. & Maldonado, Á. (Eds.)(1922) La medicina popular peruana: Contribución al folklore médico del Perú (Tomo II). Lima: Imprenta Torres Aguirre.

Valero, H. (1969) Yanoáma: the story of a woman abducted by Brazilian Indians. Allen & Unwin, London.

Valle, J.R. & Silva, N.P (1973) Ichthyotoxicity of cannabinoids. *Cienc. e Cult.* 25: 647.

van Andel, T.R. (2000) Non-timber forest products of the North-West District of Guyana. Utrecht University.

van den Berg, M.E. (1984) Ver-o-Peso: the ethnobotany of an Amazonian market. *Advances in Economic Botany* 1: 140-149. https://www.jstor.org/stable/43931373

van den Berg, M.E. (1993) Plantas medicinais na Amazônia(contribuição ao seu conhecimento sistemático). Coleção Adolpho Ducke. Belém, Museu Paranaense Emilio Goeldi. ISBN: 85-7098-041-8.

van den Berg, M.E. & Silva, M.H.L.D. (1988) Contribuição ao conhecimento da flora medicinal de Roraima. *Acta Amazonica* 18: 23-35. https://doi.org/10.1590/1809-43921988185035

van den Eynden, V., Cueva, E. & Cabrera, O. (2004) Edible palms of Southern Ecuador. *Palms* 48: 141-157.

van der Linden, M. & López, R. (1990) Utilización de palmeras amazónicas en el nororiente peruano. *Revista Forestal del Perú* 17: 65-74.

Vargas, G. (2006) Transformación y elaboración de alimentos con especies vegetales y animales por las comunidades Cubeas del Cuduyari. SINCHI, Bogotá. http://hdl.handle.net/20.500.12324/2182

Vargas, L. (1997) Vida y medicina tradicional de los Mosetenes de Muchanes. *Ecología en Bolivia* 29: 19-44.

Vásquez, M. & Vásquez, J.B. (1998) La extraccíon de productos forestales diferentes de la madera en el ámbito de Iquitos-Perú. *Folia Amazonica* 9: 69-84. https://revistas.iiap.gob.pe/index.php/foliaamazonica/article/download/155/144/

Vásquez, R. (1992) Sistemática de las plantas medicinales de uso frecuente en el área de Iquitos. *Folia Amazonica* 4: 65-80. http://www.iiap.org.pe/upload/Publicacion/PUBL735.pdf

Vasquez, R. & Gentry, A.H. (1989) Use and misuse of forest-harvested fruits in the Iquitos area. *Conservation Biology* 3: 350-361. https://www.jstor.org/stable/2386216

Vasquez, R. & Gentry, A.H. (s.d.) cited in Duke's Amazonian Ethnobotanical Dictionary.

Vásquez, S.P.F., Mendonça, M.S.D., & Noda, S.D.N. (2014) Etnobotânica de plantas medicinais em comunidades ribeirinhas do Município de Manacapuru, Amazonas, Brasil. *Acta Amazonica* 44: 457-472. https://doi.org/10.1590/1809-4392201400423

Vázquez de Espinosa, A. (c. 1600–1620/1948) Compendio y descripción de las Indias Occidentales (C. U. Clark, Ed.). Smithsonian Miscellaneous Collections, 108, xii + 801 pp. Washington, D.C.: Smithsonian Institution.

Vázquez, M.R. (1990) Useful Plants of Amazonian Peru. Spanish Typescript. Second Draft. Filed with USDA's National Agricultural Library.

Veiga, J.B.D. (2011) Etnobotânica e etnomedicina na Reserva de Desenvolvimento Sustentável do Tupé, baixo rio Negro: plantas antimaláricas, conhecimentos e percepções associadas ao uso e à doença. Tese de Doutourado. Instituto Nacional de Pesquisas da Amazônia. https://repositorio.inpa.gov.br/handle/1/12845

Velasco, J. de (1927). Historia del Reino de Quito en la América meridional (Tomo I, Parte 1: Historia Natural; obra original de 1789). Quito: Imprenta Nacional.

Vélez, G.A. & Vélez, A.J. (1999) Sistema agroforestal de las chagras indígenas del Medio Caquetá. TROPENBOS.

Vespucci, A. (1504) Las cuatro navegaciones: Carta al Illustrísimo Renato, Rey de Jerusalén y de Sicilia. In: Colección de los viajes y descubrimientos que hicieron por mar los españoles... con varios Documentos inéditos, ed. M. de Fernández. (5 vols). Madrid: 1825–1837.

Vickers, W.T. & Plowman, T. (1984) Useful plants of the Siona and Secoya indians of Eastern Ecuador. *Fieldiana* 15: 1-63. https://doi.org/10.5962/bhl.title.2600

Villa Muñoz, G. et al. (2016) Common Trees of Yasuní. A guide for identifying the common trees of the Ecuadorian Amazon. Centro del Publicaciones de la PUCE, Quito.

Villavicencio, M. (1858) Geografía de la República del Ecuador. Imprenta de R. Craighead, New York.

von Hildebrand, M. (1975) Origen del mundo según los Ufaina. *Revista Colombiana de Antropología* XVIII: 321–382. https://doi.org/10.22380/2539472X.1609

Von Reis, S. (1973) Drugs & Foods from Little-known plants. Notes in Harvard University Herbaria. Harvard University Press, Cambridge, Massachusetts.

Von Reis, S. & Lipp, F.J. (1982) New Plant Sources for Drugs and Foods from the New York Botanical Garden Herbarium. Harvard University Press. Cambridge. Massachusetts.

Vormisto, J. (2002) Making and marketing chambira hammocks and bags in the village of Brillo Nuevo, northeastern Peru. *Economic Botany* 56: 27-40. https://www.jstor.org/stable/4256517

Wallace, A.R. (1853) A Narrative of Travels on the Amazon and Rio Negro. Reeve and Co., London.

Wallace, A.R. (1853) Palm Trees of the Amazon and their Uses. J. Van Voorst, London. DOI: 10.5962/bhl.title.11168

Waller, J. & Aoreana. (1979) Guayabero (C. Morales, Trans.). In I. L. V. (Vol. II, pp. 229–250).

Waterton, C. (1879) Wanderings in South America. Macmillan and Company, London.

Wavrin, Marquis de (1937) Mœurs et coutumes des Indiens sauvages de l'Amérique du Sud. Paris: Payot.

Webster, L.J. (1970) Letter to R.E. Schultes. February 5, 1970.

Wheeler, M.A. (1970) Siona use of chambira palm fiber. *Economic Botany* 24: 180-181. https://doi.org/10.1007/BF02860598

Whiffen, T. (1915) The north-west Amazons: notes of some months spent among cannibal tribes. Constable and Co., London.

Wilbert, J. (1977). Navigators of the winter sun. In E. P. Benson (Ed.), The sea in the pre-Columbian world: A conference at Dumbarton Oaks, October 26th, 1974 (pp. 16–46). Washington, DC: Trustees for Harvard University.

Williams, E.P. (1962) Algunos datos sobre el barbasco. *Boletín de la Sociedad Venezolana de Ciencias Naturales* 6: 21- 34.

Woodroffe, J.F. (1914). The Upper Reaches of the Amazon. Methuen & Company, Limited.

Wurdack, J.J. (1958) Indian narcotics in southern Venezuela. *The Garden Journal* 8: 116-118.

Xavier, W.K.S. & Cunha, E.D.S. (2015) Comercialização de produtos naturais medicinais oriundos do Estado do Amapá. *Biota Amazonia* 5(2): 23-25. https://doi.org/10.18561/2179-5746/biotaamazonia.v5n2p23-25

Yanomami, M.I. et al. (2015) Hwërimamotima thë pë ã oni: Manual dos remédios tradicionais Yanomami. São Paulo: Boa Vista, Brazil. https://acervo.socioambiental.org/acervo/publicacoes-isa/manual-dos-remedios-tradicionais-yanomami

Yde, J. (1965) Material culture of the Waiwái. *Nationalmuseets skrifter, Etnografisk række* 10: 70–113. Copenhagen: Nationalmuseet.

Zambrana, N.Y.P. & Bussmann, R.W. (2018) La etnobotánica de los Chácobo en el siglo XXI. *Ethnobotany Research and Applications* 16: 1-149. https://ethnobotanyjournal.org/index.php/era/article/view/1289

Zarucchi, J.L. (1980) Ibapichuna: an edible *Dacryodes* (Burseraceae) from the northwest Amazon. *Botanical Museum Leaflets* 28(1): 81-85. https://www.jstor.org/stable/41762827

Zawadzky, A. (1947) Viajes misioneros del R. P. Fr. Fernando de Jesús Larrea, franciscano, 1700–1773. Cali: Imprenta Bolivariana. VII + 326 pp.

Zent, E.M.L. (1999) Hoti Ethnobotany: Exploring the interactions between plants and people in the Venezuelan Amazon (Doctoral dissertation, University of Georgia).

Zerries, O. (1960) Medizinmannwesen und Geisterglaube der Waika-Indianer des Oberen Orinoco. Ethnologica, N. F., vol. 2. Cologne: E.J. Brill G.m.b.H.
