# Supplementary material for: The forest of knowledge under global change
Source: Nature. 2026 Jul 8;655(8125):1212–6. doi: 10.1038/s41586-026-10741-y (PMC13421348; doi:10.1038/s41586-026-10741-y)
Supplement: Supplementary file 6 — Pairwise Wilcoxon rank-sum tests comparing projected relative range change among culturally widespread, shared, and unique plant species across scenarios, occurrence thresholds (occ), and Continuous Boyce Index (CBI) thresholds. Reported p-values indicate no significant differences (all p > 0.05). [file 41586_2026_10741_MOESM6_ESM.pdf]

Supplementary Table 3 I Pairwise Wilcoxon rank-sum tests comparing projected relative range change among culturally widespread, shared, and unique plant species across scenarios, occurrence thresholds (occ), and Continuous Boyce Index (CBI) thresholds. Reported p-values indicate no significant differences (all $p > 0.05$).

Scenario	CBI	occ	Widespread vs Shared	Widespread vs Unique	Shared vs Widespread	Shared vs Unique	Unique vs Widespread	Unique vs Shared
ssp1-2.6	0.5	5	0.69	0.548	0.69	0.31	0.548	0.31
ssp1-2.6	0.5	10	0.69	0.548	0.69	0.31	0.548	0.31
ssp1-2.6	0.5	20	0.841	0.841	0.841	0.548	0.841	0.548
ssp1-2.6	0.7	5	0.548	0.841	0.548	0.421	0.841	0.421
ssp1-2.6	0.7	10	0.421	1	0.421	0.421	1	0.421
ssp1-2.6	0.7	20	0.421	0.841	0.421	0.548	0.841	0.548
ssp1-2.6	0.9	5	0.222	0.548	0.222	0.222	0.548	0.222
ssp1-2.6	0.9	10	0.222	0.548	0.222	0.222	0.548	0.222
ssp1-2.6	0.9	20	0.222	0.548	0.222	0.222	0.548	0.222
ssp3-7.0	0.5	5	0.548	0.421	0.548	0.151	0.421	0.151
ssp3-7.0	0.5	10	0.548	0.421	0.548	0.31	0.421	0.31
ssp3-7.0	0.5	20	0.548	0.841	0.548	0.421	0.841	0.421
ssp3-7.0	0.7	5	0.421	0.69	0.421	0.421	0.69	0.421
ssp3-7.0	0.7	10	0.421	0.841	0.421	0.421	0.841	0.421
ssp3-7.0	0.7	20	0.421	0.548	0.421	0.421	0.548	0.421
ssp3-7.0	0.9	5	0.151	0.841	0.151	0.151	0.841	0.151
ssp3-7.0	0.9	10	0.151	0.841	0.151	0.151	0.841	0.151
ssp3-7.0	0.9	20	0.151	0.841	0.151	0.151	0.841	0.151
ssp5-8.5	0.5	5	0.548	0.31	0.548	0.222	0.31	0.222
ssp5-8.5	0.5	10	0.548	0.31	0.548	0.31	0.31	0.31
ssp5-8.5	0.5	20	0.548	0.548	0.548	0.421	0.548	0.421
ssp5-8.5	0.7	5	0.31	0.69	0.31	0.222	0.69	0.222
ssp5-8.5	0.7	10	0.31	0.841	0.31	0.222	0.841	0.222
ssp5-8.5	0.7	20	0.222	0.421	0.222	0.31	0.421	0.31
ssp5-8.5	0.9	5	0.056	0.421	0.056	0.095	0.421	0.095
ssp5-8.5	0.9	10	0.056	0.421	0.056	0.095	0.421	0.095
ssp5-8.5	0.9	20	0.056	0.421	0.056	0.095	0.421	0.095
